# Metal ions affect abiotic sugar oligomerization reactions

**DOI:** 10.1038/s42004-026-02129-w

**Published:** 2026-07-13

**Authors:** Daniil Akulenko, Rotem Edri, Moran Frenkel-Pinter

**Affiliations:** 1 Institute of Chemistry, The Hebrew University of Jerusalem, Jerusalem, Israel; 2 The Center for Nanoscience and Nanotechnology, The Hebrew University of Jerusalem, Jerusalem, Israel; 3 Casali Center of Applied Chemistry, The Hebrew University of Jerusalem, Jerusalem, Israel

**Keywords:** Origin of life, Monosaccharides

## Abstract

Numerous long-standing questions in origins-of-life research center on the origin of today’s biopolymers. The dependence of biochemistry on metals appears to be ancient and rooted in chemical evolution. While the effect of metal ions on the abiotic polymerization of peptides and nucleic acids has been investigated, their effect on sugar oligomerization reactions has not been established. Here, we have performed drying reactions of glucose as a model saccharide in the presence or absence of various metal ions and characterized the resulting products using a variety of analytical techniques. We found that while metal ions typically inhibited the overall conversion of glucose into oligomers, some ions led to an apparent shift in oligomeric distribution, in a metal-specific manner. Our results are suggestive of metal-sugar co-evolution during the early stages of prebiotic evolution.

## Abstract (web summary)

Although the effect of metal ions on the abiotic polymerization of peptides and nucleic acids has been investigated before, their effect on sugar oligomerization reactions remains underexplored. Here, the authors report on glucose drying reactions in the presence or absence of metal ions and show that, while metal ions typically inhibit the overall conversion of glucose into oligomers, some ions lead to a metal-specific apparent shift in oligomeric distribution.

## Introduction

The origin of life is one of the biggest mysteries in the field of chemistry and science in general^1–5^. While it is well known that Earth formed around 4.54 billion years ago^6^, it is unknown how and when the first biopolymers arose and under which environmental conditions^7–9^. Biology is dominated by highly evolved biopolymers, namely polysaccharides, proteins, and nucleic acids^10–12^. It is known that the building blocks of today’s biopolymers, such as sugars, amino acids, and nucleobases, existed on the prebiotic Earth. Sugars are highly abundant in meteorites^13–15^ and in model prebiotic reactions^16–20^. For example, in 1861, Butlerov successfully synthesized glycolaldehyde from formaldehyde, along with longer C3-C6 carbohydrates^21^. This reaction, known as the “formose reaction,” is considered a pivotal prebiotic reaction leading to various sugar-related building blocks^22^, some of which have also been detected in meteorites^23,24^, although recent NMR-based mechanistic studies have prompted reconsideration of its capacity to generate biologically relevant linear aldoses^22^.

While abiotic polymerization of peptides and nucleic acid polymers has been studied extensively^25–41^, abiotic oligosaccharide formation has been reported only twice, in drying reactions of glucose^42^ and in the formation of disaccharides from various monosaccharides at aqueous microdroplet interfaces^19^. For example, glucose oligomerization was studied under acidic conditions at 50 °C and during wet-dry cycling for 10 weeks. It was found that glucose can easily oligomerize into long oligomers such as octamers and nonamers, and that a steady state was reached after ~5 cycles. The authors observed favorable formation of 1,6-bonds, followed by 1,4-, 1,3-, and 1,2-bonds, while 1,1-bonds were seen very rarely. In addition, hydration-dehydration cycles that enable hydrolysis and acetal bond formation of different glucose dimers were performed, and it was found that selective formation of 1,6-acetal bonds occurs, independently of the starting regioselectivity of the disaccharide. Together, these findings suggest that sugars can undergo selective abiotic oligomerization under plausible prebiotic conditions. Further research is needed to establish the robustness of abiotic oligomerization reactions of sugars, particularly because polysaccharides are the largest class of biopolymers by mass in today’s biology and yet remain understudied in the context of prebiotic chemistry. Carbohydrates play an important role in biology, for example, as an energy source in nutrition, in metabolism, in cell recognition and communication, and as structural elements. While it is now known that oligosaccharides can form abiotically^42^, it is unknown whether metal ions have any effect on the polymerization of sugars.

Metal ions were highly abundant on prebiotic Earth and likely participated in various prebiotic pathways^43–49^. The role of metal ions in today’s biology is well established^50–55^. Metal ions mediate catalysis, stabilize protein structure, and participate in ligand transport as well as signaling. Around half of the known proteins associate with metal ions or with organometallic cofactors, and about one-third of all enzymes are metalloenzymes. For instance, hemoglobin, which is one of the most essential proteins in the bloodstream responsible for transferring molecular oxygen, contains Fe^2+^, which directly facilitates oxygen binding^50^. In the context of catalysis, a wide variety of enzymes, such as SOD and PHD, contain various metal ions that serve as the catalytic moiety^50^. Metal ions participate in cellular processes, such as peptide bond formation^56–58^ and oxidative phosphorylation^59^. Metal ions can also serve as cofactors; for example, Mg^2+^ ions are essential for RNA folding and catalysis^60^. This role might have been fulfilled by Fe^2+^ in the prebiotic era^60^.

Consistent with their central biological roles, in the context of prebiotic chemistry, it has been demonstrated that peptide and RNA formation can be catalyzed by metal ions^25,56–58,61–70^. For example, on the surface of octacyanomolybdate (IV) metal complexes, such as those involving Zn^2+^ and Cu^2+^ ions, the oligomerization of glycine and alanine into dimers occurs under relatively mild conditions (60 °C) within 7 days^57^. In addition, Mg^2+^ acts as a cocatalyst for the oligomerization of mononucleotides in the presence of montmorillonite^69^. Moreover, the effect of metals and minerals on the formose reaction has been investigated^17,45,71–75^. For instance, olivine silicates, which are minerals that contain Mg^2+^ and Fe^2+^, catalyze glyceraldehyde formation from formaldehyde and subsequently the formose reaction^24^. The mineral provides a stable alkaline environment that promotes this base-catalyzed reaction, stabilizes the enediolate intermediate, and promotes the autocatalytic cycle. Recent studies have argued that peptides and other organic molecules that solubilize metals for redox chemistry could have emerged in the Archean ocean as a central mechanism for structural diversification^43,46^. Notably, minerals were also abundant on the ancient Earth and likely catalyzed various reactions^76–85^.

If metal ions influenced the abiotic formation of peptides and nucleic acids, they may also have affected the abiotic formation of oligosaccharides. Sugar-metal ion interactions are abundant in nature^86–91^. These interactions are pivotal in processes such as the transport and storage of metal ions, binding of glycoproteins to cell surfaces, and stabilization of membrane structures^86,87^. It is known that metal ions can coordinate with sugars via their hydroxyl groups^89–92^. For instance, glucose can coordinate with alkaline and alkaline-earth metals via O–2 and O–1^90–92^. Cations such as Zn^2+^ and Mg^2+^ bind one molecule of glucose and four H_2_O molecules in these coordinated complexes^90^. It is likely that sugars and metal ions co-evolved throughout chemical evolution. Hence, it may be possible to answer long-standing questions about the origin of life by understanding how metals interact with ancestral biopolymers, such as oligosaccharides.

In this paper, we tested the hypothesis that metal ions affect oligomerization reactions of sugars. To test this hypothesis, we performed abiotic oligomerization reactions of glucose as a model sugar in the presence or absence of various metal ions. The tested metals included Li^+^, Na^+^, K^+^, Mg^2+^, Ca^2+^, Mn^2+^, Fe^2+^, Zn^2+^, and Al^3+^. The reaction products were chemically analysed by ESI-MS, LC-MS, NMR, and FTIR. We found that while metal ions typically inhibited the overall conversion of glucose, some metal ions, such as Al^3+^, Ca^2+^, and Fe^2+^, affected the oligomeric distribution. Our results suggest that interactions of primordial sugars with metal ions could have had dramatic effects on the course of early chemical evolution.

## Results and discussion

### Oligomerization of glucose via dry-down reactions in the presence or absence of metal ions

**Fig. 1:**
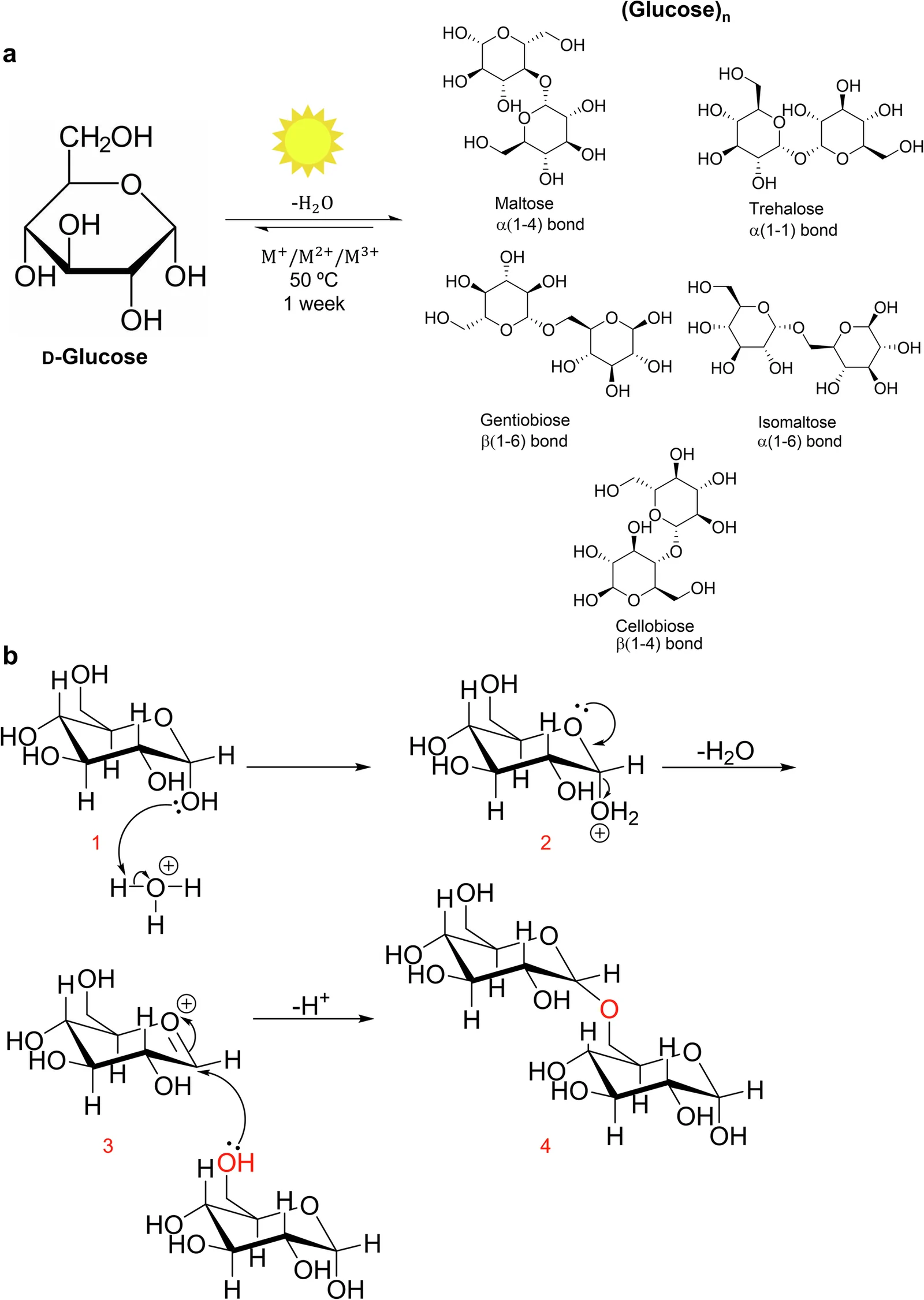
General experimental scheme and acid-catalyzed acetal bond formation. **a**_D_-Glucose was dried down for 1 week at 50 °C in the presence or absence of various metal ions. The resulting products were characterized by LC-MS, FTIR, and NMR. **b** The mechanism for acid-catalyzed acetal bond formation is shown (in red).

_D_-Glucose (Glu) was dried at 50 °C for 1 week in the presence or absence of 1 eq. of metal ions under unbuffered conditions adjusted by the addition of HCl or NaOH (Fig. 1). HCl was added to promote oligomerization, as acetal bond formation is acid-catalyzed. NaOH was added to examine the effects of metal ions under alternative conditions, even though this environment is not expected to favor sugar oligomerization. The following metal ions were used: Li^+^, Na^+^, K^+^, Mg^2+^, Ca^2+^, Mn^2+^, Fe^2+^, Zn^2+^, and Al^3+^. The product mixtures were characterized by various analytical methods, such as nuclear magnetic resonance (NMR), Fourier transform infrared spectroscopy (FTIR), electrospray ionization mass spectrometry (ESI-MS), and liquid chromatography mass spectrometry (LC-MS), in accordance with prior investigation that identified the various reaction products^42^. The reaction involving Fe^2+^ was carried out under anaerobic conditions to minimize oxidation of Fe^2+^ to Fe^3+^. To verify that the atmosphere does not impact glucose oligomerization reactions for other metal ions, we performed a reaction in which glucose was oligomerized in the presence or absence of Zn^2+^ under anaerobic conditions, and indeed, no effect of the atmosphere was observed (Supplementary Fig. S1).

**Fig. 2:**
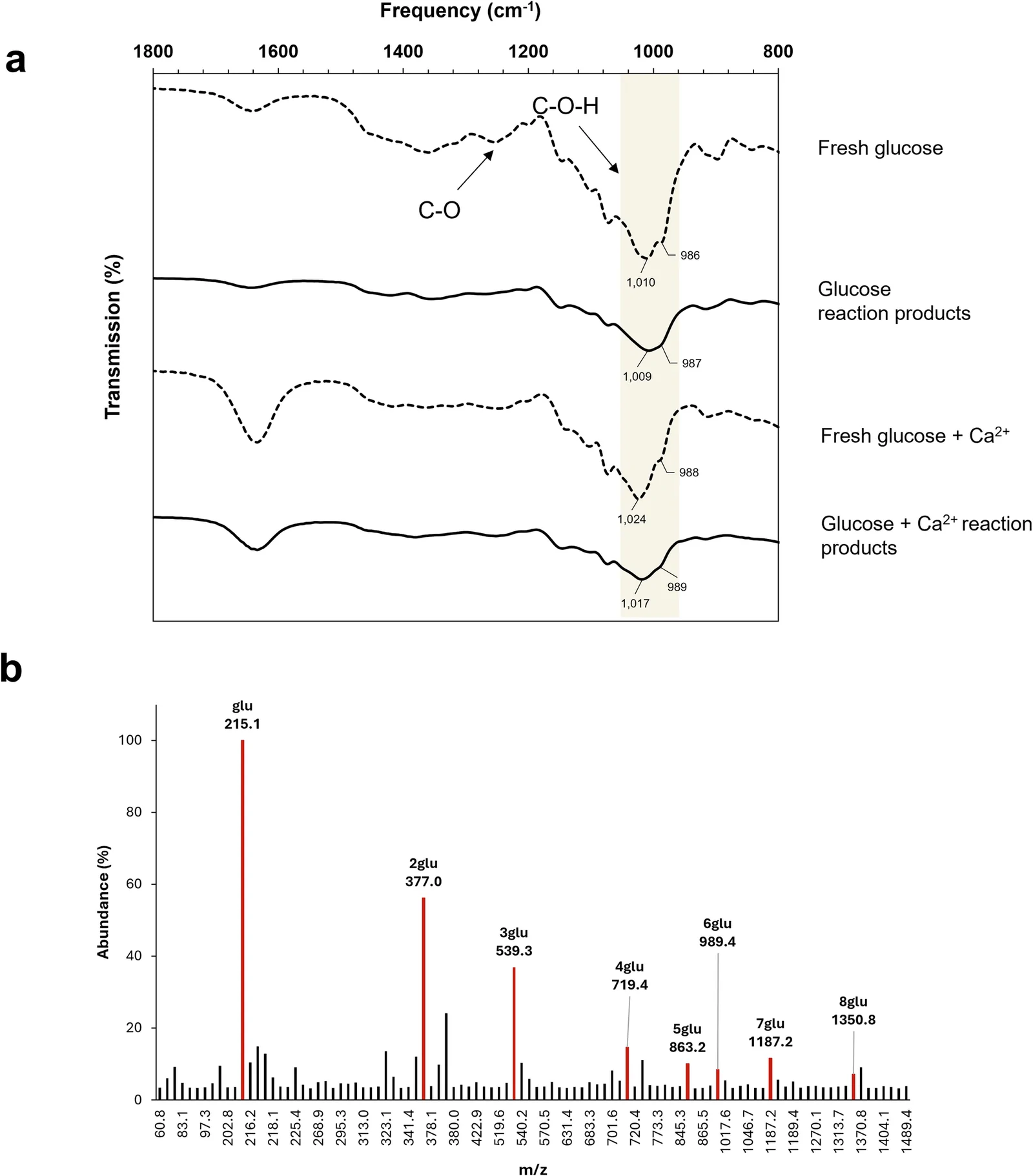
Characterization of dry-down reactions of _D_-Glucose under acidified conditions via FTIR and MS analysis in the absence of metal ions. **a** An FTIR spectra stack of _D_-Glucose in the absence or presence of Ca^2+^ before (dashed lines) and after drying for 1 week at 50 °C (solid lines). **b** MS negative mode analysis of _D_-Glucose that was dried for 1 week. Labeled species correspond to [M + Cl]^−^ ions.

FTIR can be indicative of the formation of new glycosidic bonds at ~1050 cm^−1^ as well as complexation with metal ions^88,92–94^. An indication for the formation of new glycosidic bonds was observed via broadening of the C-O-H peak (~1050–1000 cm^−1^) upon drying reactions (Fig. 2a and Supplementary Figs. S2–S20)^93^. An indication for interactions between glucose saccharides and metal ions, which might be indicative of complexation, was observed in frequencies in the range of ~1300–800 cm^−1^. For example, such differences were noted in the presence of Ca^2+^ under acidified conditions. The C-O-H peak positions for fresh and dried samples in the presence of Ca^2+^ were 1024 and 1017 cm^−1^, respectively. In contrast, the C-O-H peak positions for the fresh and dried samples in the absence of Ca^2+^ were 1010 and 1009 cm^−1^, respectively.

MS analysis confirmed the presence of glucose oligomers in both modes (negative and positive, Supplementary Figs. S21–S60), as previously reported^42^. We note that the negative mode was more informative due to better ionization of the sugar molecules with Cl^−^ adducts (Fig. 2b). For acidified dry-down reactions, we observed the formation of glucose oligomers of up to octamers. On the other hand, upon addition of NaOH to the dry-down reaction mixtures, we observed the formation of only glucose dimers in most cases. In the samples that contained glucose and either Mn^2+^, Zn^2+^, or Fe^2+^, trimers and tetramers were also observed, which might be explained by the lower pH (~5–6) of these product mixtures following 1 week of dry-down (Supplementary Fig. S61 and Table S1). The decrease in pH levels can be attributed to the formation of insoluble hydroxides in the mixture. This observation is explained mechanistically by the fact that acidic pH drives protonation of the anomeric oxygen and hence increases the rate of the reaction (Fig. 1b). Indeed, the pH of the glucose sample in the presence of Al^3+^ was higher than that of the other samples (~4 instead of ~2), and the oligomerization extent in its presence was lower, likely due to the distinct Al^3+^-dependent hydrolysis/speciation environment. The pH is not the only factor, as product mixtures involving glucose and either Mn^2+^ or Ca^2+^, upon addition of NaOH, also had lower pH, but did not result in trimer formation. It is worth noting that in the presence of NaOH, the reaction involves deprotonation of the anomeric hydroxyl and ring-opening. This mechanism probably favors reactions other than oligomerization, such as isomerization^17,95–97^.

In the MS-positive mode, we observed adducts involving sugars (monomers and oligomers) and cations at a 1:1 molar ratio (e.g., [Glu + Li]^+^, [Glu + Mg]^2+^, Table S2), in accordance with previous studies that investigated sugar-metal ion interactions^90,91,93^. For instance, Mg^2+^ forms a stable complex with one glucose molecule and four water molecules^92^. On the other hand, it is known that at a 1:1 molar ratio of Ca^2+^ and glucose, a 7-coordinated complex is observed, whereas for a 1:2 molar ratio (Ca^2+^: glucose), the coordination number is eight, in which each metal ion is bound to two units of monosaccharide^93^. We also detected sugar-metal ion adducts via MS in fresh, non-dried samples. These observations are consistent with sugar-metal ion association, although we note that adducts detected by ESI-MS do not necessarily correspond to pre-existing complexes in solution or in the dry-down reaction mixture, since some adduct formation may also occur during ionization in the instrument. We further note that no major qualitative differences in positive-mode adduct formation were observed between fresh samples adjusted with HCl or NaOH (e.g., Figs. S43 and S53).

### Conversion of glucose into products in the presence or absence of various metal ions

In order to quantify the conversion of glucose into other products in the presence or absence of various metal ions, we integrated ^1^H-NMR resonances of the anomeric protons before and after the dry-down reactions (Fig. 3 and Supplementary Figs. S62–S123). Spiking with standards of glucose dimers (cellobiose, isomaltose) and trimers (maltotriose, isomaltotriose) confirmed the presence of various glucose oligomers following drying reactions (Supplementary Figs. S124–S131). Notably, not all products correspond to oligomers, as elimination or isomerization side products may form, especially under basic conditions. It is well established that the generation of new glycosidic bonds involves one anomeric carbon, and that the formation of this linkage between two anomeric carbons is negligible^42,98^. Therefore, glucose oligomerization reactions result in a shift in the anomeric proton on the non-reducing end and allow us to estimate the extent of glucose conversion. Notably, we acknowledge that integration of the anomeric protons is an underestimation of glucose conversion due to a partial overlap between the signals of the reducing end of glucose monomers and glucose oligomers.

**Fig. 3:**
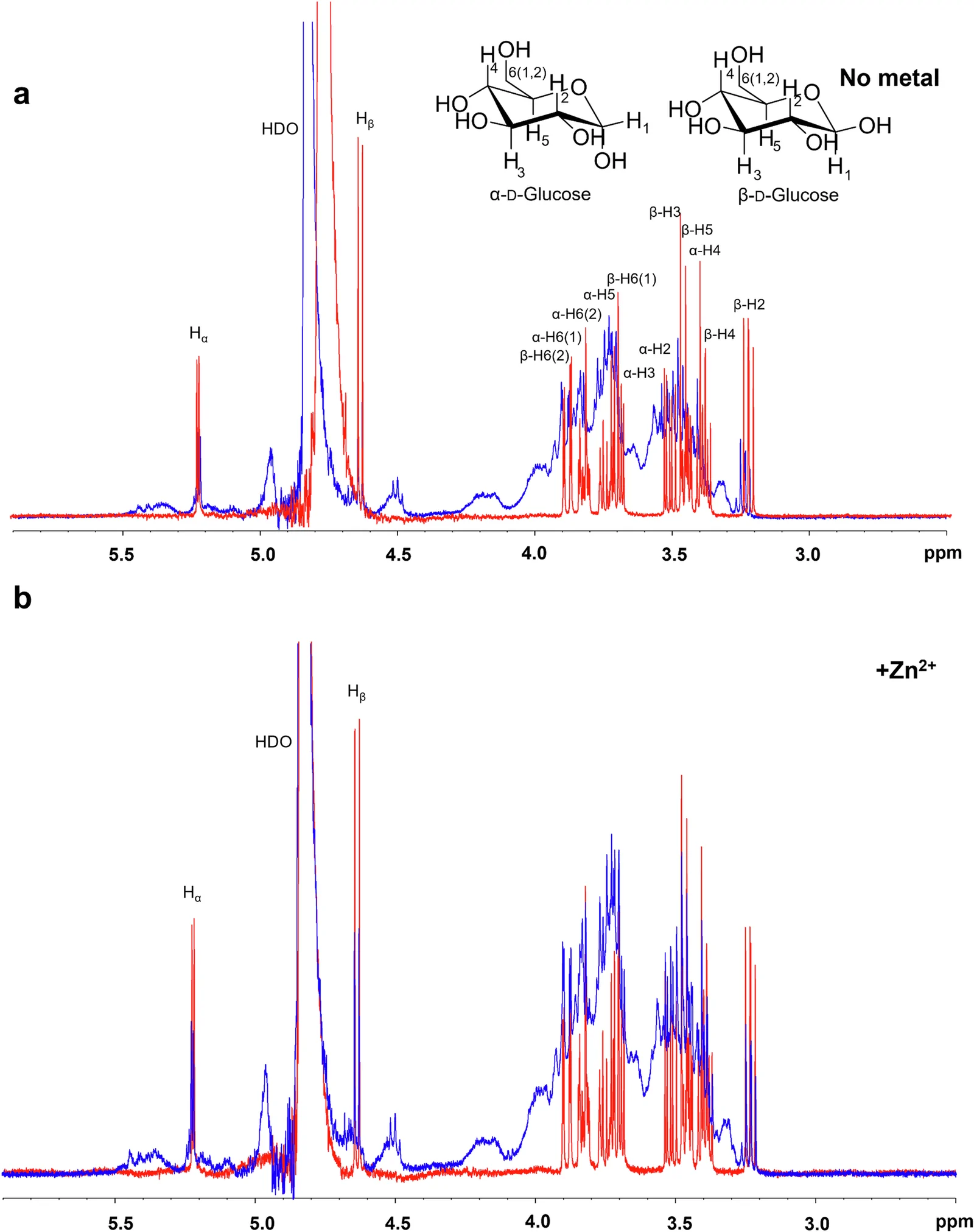
^1^H-NMR spectra of _D_-Glucose. Overlay of ^1^H-NMR spectra of a fresh (red) and a dry-down reaction at 50 °C for 7 days under acidified conditions (blue) of _D_-Glucose in the absence (**a**) or presence of Zn^2+^ (**b**). Samples were dissolved in D_2_O buffer (pH = 2.66).

We noticed, as previously reported, that the anomeric ratio is distinct in the presence of Ca^2+^ ions^99^. We studied changes in the anomeric ratio through a kinetics analysis of acidified fresh solutions of glucose to which 1 eq. of Ca^2+^ was added and observed that the ratio reached an equilibrium after ~240 min (Supplementary Fig. S132). pH is also known to affect the α-β anomeric ratio equilibrium, with the beta anomers becoming more predominant as pH increases^99^. The anomeric ratio is also expected to affect the sugar’s reactivity, probably at the protonation/deprotonation step.

**Fig. 4:**
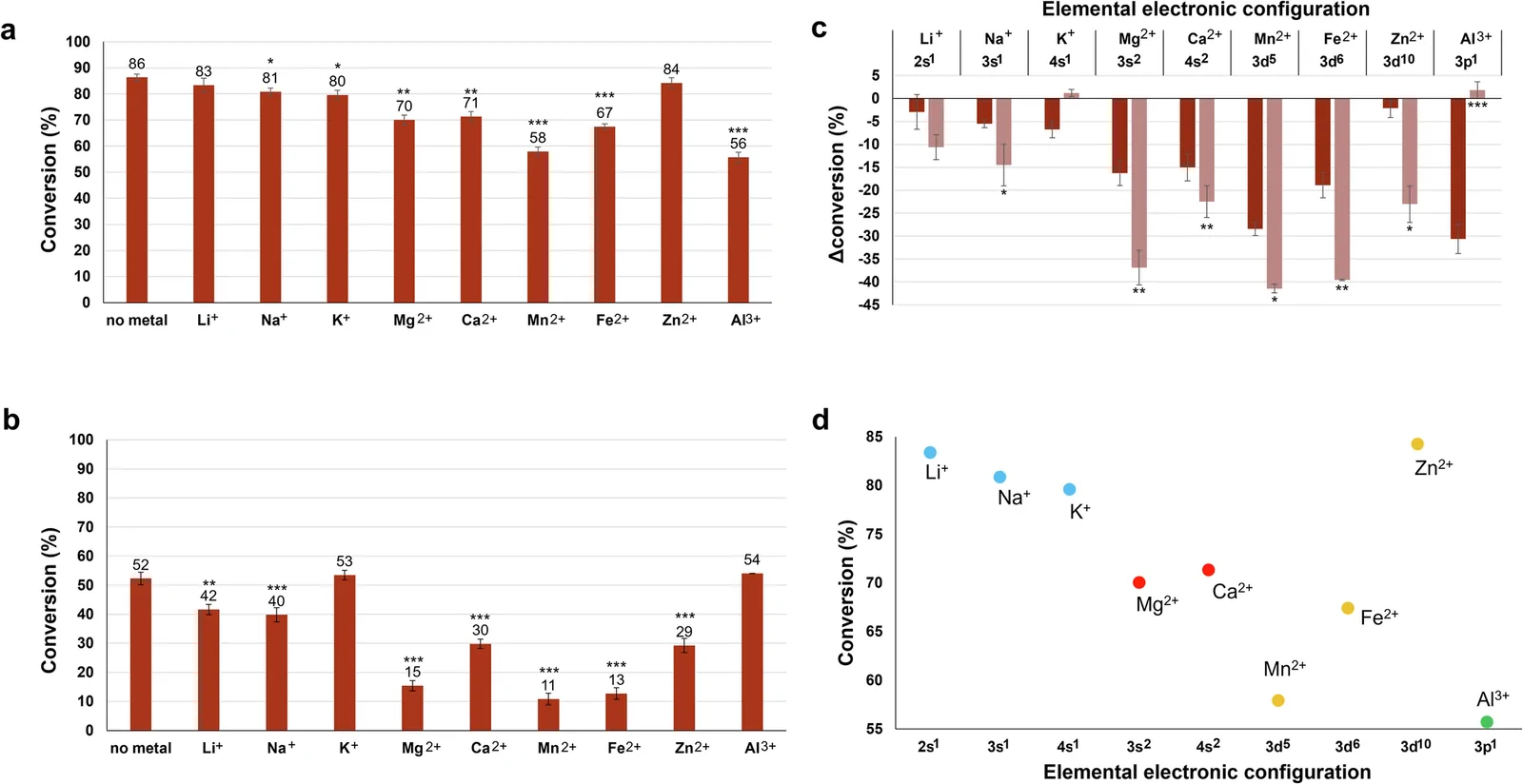
Quantification of conversion of _D_-Glucose into products based on ^1^H-NMR. Glucose conversion after 1 week of dry-down at 50 °C in the presence or absence of metal ions under acidified conditions (**a**) or upon addition of NaOH (**b**). Values are presented as the mean values of three independent replicate analyses with standard deviation. **c** Difference between the conversions of glucose in the presence of different metal ions under acidified conditions (red) and upon addition of NaOH (pink). Values are presented as the mean values of three independent replicate analyses with standard deviation. **d** A plot summarizing the conversion of _D_-Glucose under acidified conditions versus the elemental electronic configuration of the tested metals, highlighting trends across the tested metal set. *P*-values were calculated via a two-sided t-test (* indicates *p* < 0.05, **p < 0.01, ***p < 0.001, *n* = 3 independent experiments). The corresponding *p*-values are provided in Table S4.

In addition to affecting the anomeric ratio, pH will affect product formation and reaction rates: sugar oligomerization is acid-catalyzed, while isomerization rate increases at higher pH^17,42,95–97^. We observed that overall, under acidified conditions, the conversion of glucose was higher compared to the conversion observed upon addition of NaOH (Fig. 4a, b). Notably, Li et al. reported a much lower glucose conversion after 1 week of drying (11%)^42^, compared with our results (86%). This difference can be attributed to the higher amount of HCl (a 10-fold difference) used in this study, resulting in a much higher conversion due to acid-catalyzed acetal bond formation. Moreover, we found that, typically, metal ions in both conditions inhibited oligomerization, even though this effect was much more pronounced upon addition of NaOH (Fig. 4c). The only exceptions were Li^+^ and K^+^ ions, which did not inhibit glucose oligomerization in the presence of HCl and NaOH, respectively. For instance, while 86% of glucose was converted under acidified conditions, only 58% was converted in the presence of Mn^2+^. Similarly, while 52% of glucose was converted upon addition of NaOH, only 11% was converted in the presence of Mn^2+^ (41% reduction upon addition of NaOH compared to 28% reduction in the acidified environment).

In order to further understand the metal-specific effects on glucose oligomerization, we plotted the conversion of glucose under acidified conditions against the elemental electronic configuration of the various metals and observed clear trends (Fig. 4d). It is evident that alkaline metals did not dramatically inhibit the reaction. A non-significant trend in conversion indicates a possible positive correlation between atom size and the extent of conversion inhibition (Li < Na < K). On the other hand, alkaline earth metals (Ca and Mg) inhibited the conversion of glucose to a higher extent than alkaline metals. For transition metals, a positive correlation was observed between the number of electrons in the d-shell of the atom and the extent of glucose conversion (Zn > Fe > Mn), with only minimal inhibition for Zn (by ~2.5%). We noted that, also under the addition of NaOH, alkaline earth metals inhibited glucose oligomerization to a greater extent than alkaline metals. The conversion in the presence of Al^3+^ was similar under acidified conditions and upon addition of NaOH: 56% and 54%, respectively. As mentioned above, this can be explained by the similar pH under both conditions (pH ~4).

In order to further study the effect of metal ions on sugar oligomerization reactions, ^13^C-NMR and HSQC analyses were conducted (Supplementary Figs. S133–S140). To that end, we analyzed isotopically labeled ^13^C-glucose-6 that was dried down in the absence or presence of either Ca^2+^ or Zn^2+^ at both pH conditions. The results of ^13^C-NMR and HSQC supported our former ^1^H-NMR results: acidified samples contained more product peaks compared to samples to which NaOH was added^42^. Upon addition of NaOH, HSQC analysis revealed two peaks of anomeric C-H interaction in the absence of metals, while in the presence of both cations, only the β C-H interaction was detected. This could be potentially explained by selective interaction between the α-position of the monosaccharide and the cation, in which possibly the binding between the α-hydrogen and metal ion weakens the bond strength between the anomeric α-hydrogen and anomeric carbon. Indeed, upon the addition of NaOH, the conversion is lower compared to acidified samples. The results are supported by previous FTIR studies, indicating selective binding between the α-anomer of glucose and alkaline-earth cations^92,93^.

### Effect of various metal ions on the product distribution in glucose oligomerization reactions

The dry-down reaction of glucose under acidified conditions produced oligomers of various sizes, as indicated by ESI-MS measurements. We utilized targeted LC-MS to perform a qualitative analysis of glucose monomers as well as glucose oligomers (dimers up to nonamers) in the presence or absence of metal ions. This analysis is only qualitative due to the huge variety of products, their co-elution, presence of ions, methodological limitations, and lack of standards for the absolute majority of these species. To improve detection across products with a broad range of abundances, SIM-LC-MS analyses were therefore performed at two initial glucose concentrations (1 and 10 mM), as higher concentration facilitated detection of lower-abundance species, whereas lower concentration allowed clearer evaluation of more abundant species. Notably, the combinatorial variety of glucose oligomerization products is immense. Assuming the cyclic form only, the number of products would be 8^*n* − 1^ (where “*n*” is the number of glucose units). For instance, the number of different trimers assuming the cyclic structure would be 64 [two linkages, each of which can be either α or β (1 → 2, 1 → 3, 1 → 4, 1 → 6)]. Since the chromatographic separation was achieved on a C18-column, retention time was expected to increase with oligomer length. However, due to the presence of multiple products, oligomers of a certain chain length typically eluted as a cluster. This was observed predominantly for trimers, tetramers, pentamers, and hexamers that exhibited continuous elution (Supplementary Figs. S141–S164). Due to the choice of chromatographic separation, the longer oligomers eluted as a single peak or as a front-cluster (depending on the concentration) (Supplementary Figs. S141–S164).

**Fig. 5:**
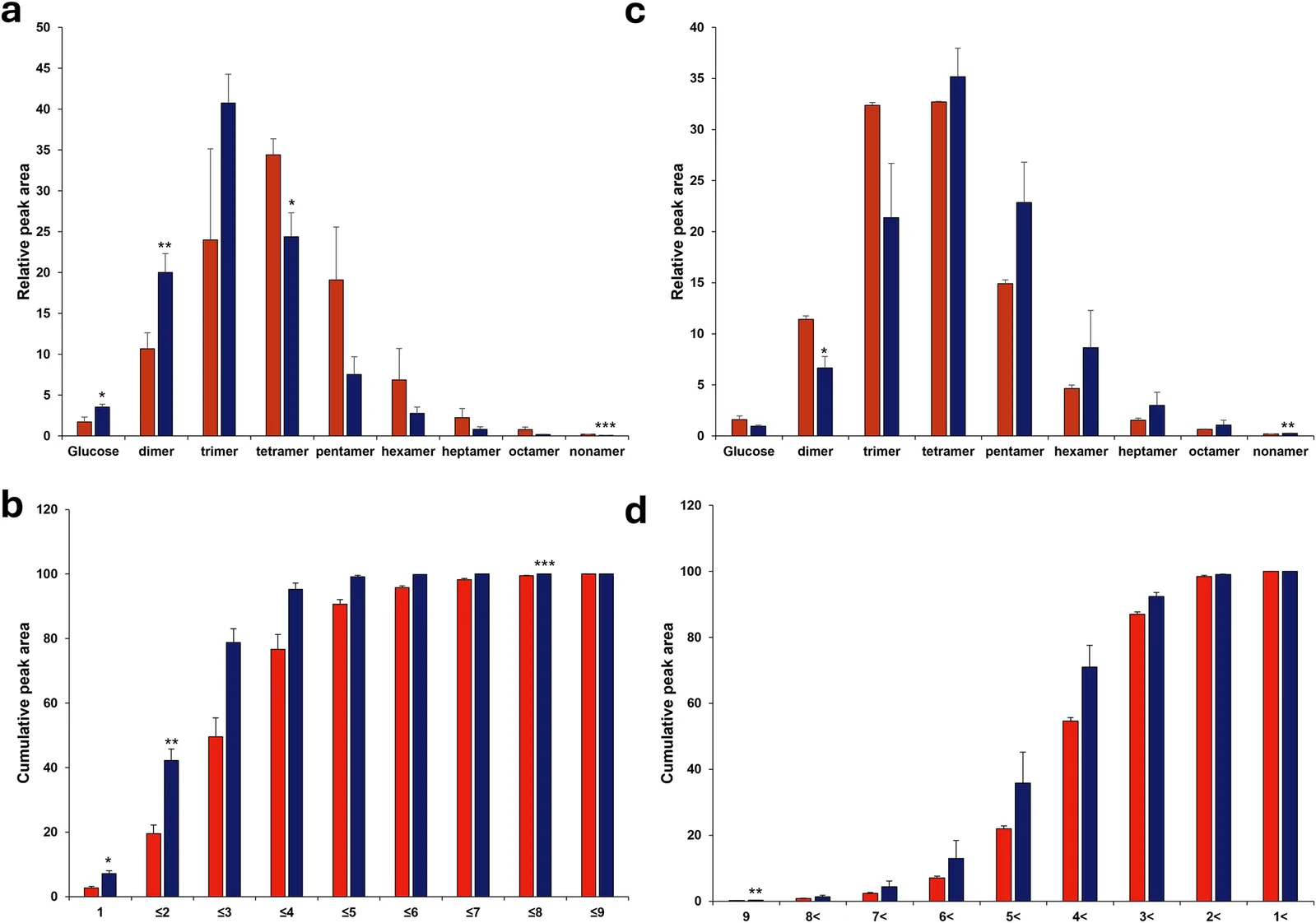
Evaluation of the degree of _D_-Glucose oligomerization based on SIM-LC-MS. Glucose product distribution and cumulative distribution after 1 week of dry-down at 50 °C in the presence (blue) or absence (red) of Al^3+^ (**a**, **b**) or Fe^2+^ (**c**, **d**) under acidified conditions. Values are presented as the mean values of three independent replicate analyses with standard deviation. Glucose concentration for the analysis was 10 mM. *P*-values were calculated via a two-sided t-test (* indicates *p* < 0.05, **p < 0.01, ***p < 0.001, *n* = 3 independent experiments). The corresponding *p*-values are provided in Table S5.

As indicated by the distribution profiles, for most samples, the most abundant oligomers in terms of peak area were trimers and tetramers (Fig. 5). When examining the peak areas of the longest oligomers (heptamers to nonamers), we did not observe significant differences between the samples, except for Ca^2+^ and Al^3+^, for which these oligomers were formed to a lesser extent compared with the control sample in the absence of metals. Al^3+^ produced the strongest shift in product distribution toward short oligomers (Fig. 5a). This trend is also evident from the cumulative distribution plot (Fig. 5b): in the presence of Al^3+^, ~80% of the total peak area corresponded to trimers or shorter oligomers, compared with ~50% in the glucose control, and ~95% corresponded to tetramers and shorter oligomers, whereas in most other samples the same cumulative proportion extended to hexamers, and to pentamers in the presence of Ca^2+^ (Supplementary Figs. S165–S176). For Fe^2+^, the product distribution suggested a modest shift toward longer oligomers (Fig. 5c), which is also reflected in the cumulative profile (Fig. 5d). In its presence, ~36% of the total peak area corresponded to pentamers or longer oligomers, compared with ~22% in the glucose control, consistent with the cumulative distribution profile (Fig. 5c, d, and Supplementary Figs. S175, 176). To assess whether glucose consumption could nevertheless be estimated by SIM-LC-MS, we performed a feasibility test using calibration curves of glucose standards analyzed in negative ionization mode (Supplementary Fig. S177). For the glucose control reaction, this analysis gave a glucose consumption value of 93 ± 1%, whereas ^1^H-NMR gave 86 ± 1%, consistent with underestimation by NMR due to partial overlap between the anomeric proton signals of glucose monomers and oligomers. While these results show that SIM-LC-MS can, in principle, be used to quantify glucose consumption in simple samples, extending this approach for the quantification of all reaction products is analytically challenging because of the large diversity of products, their co-elution, the presence of metal ions, and the lack of standards for most analytes.

### Possible explanations for the observed effect of metal ions on glucose oligomerization reactions

Taken together, while our NMR measurements clearly revealed an inhibitory effect in terms of overall glucose conversion, our qualitative LC-MS analysis indicated that there might be some differences in oligomeric distribution in the presence of specific metal ions. As the complexation of carbohydrates with metal ions has been previously reported^92,100–102^, one could expect such complexation to affect both reaction yield and oligomerization degree, as was also reported for other systems, such as depsipeptide oligomerization reactions^61^. Our LC-MS analysis suggested that for some metal ions (e.g., Ca^2+^, Fe^2+^, and Al^3+^), product formation was slightly altered compared to glucose control (in the absence of metal ions). Altogether, our results suggest that complexation of glucose with metal ions could occur and, as a consequence, can alter glucose oligomerization. Although such complexes are traditionally synthesized under non-aqueous or partially aqueous environments, their formation upon drying (i.e., by gradual evaporation of water), similarly to conditions used in this study, cannot be excluded. While in aqueous solutions, glucose-metal complexes are usually weak, in our experiments, evaporation of water results in a gradual increase in sugar and ion concentrations. Therefore, a distinct physical behavior of the system and potentially stronger interactions between glucose and metal ions may lead to their complexation.

**Fig. 6:**
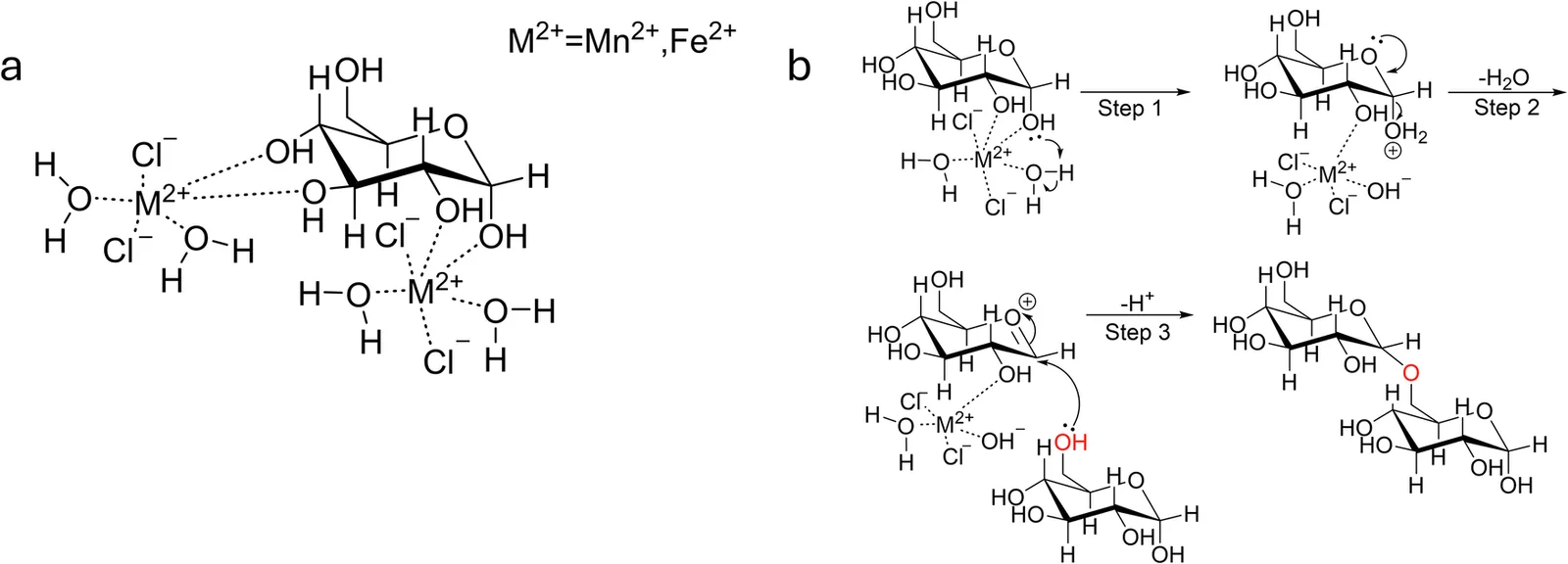
Glucose-metal ion interactions. **a** A possible complexation of glucose with transition metal ions. **b** Proposed mechanism of acetal bond formation in the presence of metal ions.

There are several possible explanations for the observed effect of metal ions on glucose oligomerization. We observed metal-specific effects, and it is likely that different metal-related effects are dominating for different metal ions (e.g., binding/coordination, Lewis acidity, pH, hardness, and electronic-structure effects) that could either inhibit or promote oligomerization. Our results do not support a strong correlation between any single parameter and the observed effects on oligomerization, and instead point to a more complex combined effect of multiple factors. Notably, the interaction of each specific metal ion with glucose monomer or oligomers could be different. As mentioned, it is known that cations can bind to glucose units^92^. It is possible that direct coordination between the anomeric oxygen and the metal cations led to inhibition of oligomerization due to hindrance of the anomeric group that participates in glycosidic bond formation, as protonation could be hindered. Indeed, transition metals, such as Mn^2+^ and Fe^2+^, can form stable complexes with glucose, in contrast to alkaline metals (Fig. 6a)^101,102^. Accordingly, various metal ions have been found to inhibit the formation of other biopolymers, such as depsipeptides^61^. Along these lines, thermodynamic calculations for glycine indicated that, in the presence of metal ions, the oligomer-monomer equilibria favor the monomeric form, especially in neutral or alkaline pH^103^. Although these systems are not directly analogous to sugar oligomerization, they support the broader idea that metal ions can alter condensation equilibria in ways that may disfavor oligomer formation.

In a previous work, Angyal suggested that carbohydrate-metal complex formation is favorable when three adjacent hydroxyls occupy *ax-eq-ax* positions in a chair conformation^104^. While this is not the case with glucose, its complexation with different metal ions has been reported. For instance, Pallagi et al. studied the complexation of glucose and Ca^2+^ in aqueous solution^105^. Despite the weak interaction in aqueous environment, they found that glucose and Ca^2+^ form a complex with a formation constant K of 1.5 and 1.7 L mol^−1^ for the α- and β-anomers, respectively. It was suggested that Ca^2+^ coordinates glucose at three sites: the ring oxygen, the anomeric oxygen, and the oxygen on C6. In another work, Tajmir-Riahi et al. reported that the complexation of Mg^2+^ and Ca^2+^ with α-glucose preferentially involves coordination at the C1 and C2 hydroxyl groups^92^. α-Glucopyranose exhibits *cis* configuration, which can support coordination^99,100^. Altogether, such complexation of Ca^2+^ and glucose, especially when mediated through the anomeric hydroxyl, may hinder access to the anomeric leaving group and inhibit the reaction.

The effects of the metal ions, observed mostly for divalent cations such as Mg^2+^ and Ca^2+^, can potentially be attributed to mechanistic aspects of glucose complexation (Fig. 6b). In the first step of the reaction, metal ion coordination to α-D-glucose at O–1 and O–2 can hinder protonation of the anomeric oxygen. In the second step of the reaction, it is expected that the cation binds only to O–2, while O–1 undergoes elimination. In the third step of the reaction, nucleophilic attack via a hydroxyl from another sugar that occurs on the oxocarbenium intermediate at the anomeric position leads to glycosidic bond formation. It is possible that the relatively large cation radius of Ca^2+^ might lead to steric hindrance during the various steps of the reaction. Coordination of the cation with various glucose oligomers could also inhibit the reaction. Understanding these metal-specific effects on glucose oligomerization will benefit from future studies that might unravel their complex nature and help better understand the mechanism by which each metal ion affects this oligomerization.

Notably, the most prominent effects were observed in the presence of Al^3+^, which is the only trivalent cation that was tested in our study. As mentioned, the pH of the glucose sample in the presence of Al^3+^ was higher than that of the other samples (~4 instead of ~2), and the oligomerization extent in its presence was lower. The higher pH is expected to decrease the extent of oligomerization, as acetal bond formation is acid-catalyzed. Among the studied group of cations, Al^3+^ is the hardest. Since the hydroxyl oxygens on the glucose molecule are also classified as hard donor sites, their interaction with Al^3+^ is expected to be particularly strong^106–109^. This stability is driven by the large energy gap between the HOMO of the glucose and the LUMO of the metal ion, which prevents significant electron sharing and instead facilitates a stable, primarily electrostatic interaction characteristic of hard-hard species according to the HSAB principle. This strong, localized binding can effectively hinder the reactive sites of the sugar.

Our analysis suggested that there might be a slight shift towards longer oligomers in the case of Fe^2+^. Fe^2+^ is known for its catalytic effect on various reactions, such as electron transfer in TMB or aminohydroxylation of olefins^60,110^. It is assumed that Fe^2+^ was highly abundant on the Hadean Earth prior to the Great Oxygen Event (~2.4 Ga ago) and likely played significant catalytic role in various reactions^60^. Nowadays, Fe^2+^ is mostly present in minerals, and the interaction between Fe^2+^ in Fe^2+^-enriched clinoptilolite mineral and glucose was studied^111^. It was observed that the opened form of glucose interacts with Fe^2+^ via the carbonyl oxygen.

In today’s biology, polymerization of simple saccharides into polysaccharides is performed by glycosyl transferases that catalyze glycosidic bond formation. The subgroup termed glycosyl transferase-A (GT-A) is metal-dependent, with the ionic divalent metal, mostly Mg^2+^ and Mn^2+^, stabilizing the sugar substrate in the active site of the enzyme^112,113^. Although the sugar substrates are commonly activated nucleotides, specifically phosphorylated sugars, the involvement of the metal ions in catalysis may imply a rudimentary connection to early enzyme evolution. Metal ions also affect the activity of hexokinases, which are enzymes that catalyze the phosphorylation of hexoses. In that case, the metal mediates sugar phosphorylation through complex formation with adenosine triphosphate. Interestingly, in a kinetic model of the enzymatic catalysis of hexokinases, it was found that replacement of Mg^2+^ by Ca^2+^ negatively affects the catalysis^114^. This was attributed to the effect of metal ion on polarization of bound water molecules, which directly facilitates the bond-breaking step.

While recent advancements in the field of glycobiology pave the way for a better understanding of their role in biology, the emergence of oligosaccharides under abiotic conditions is understudied, likely due to the complex nature of these reactions. Overall, our work sheds light on the rudimentary cooperation of sugars and metals under an abiotic setting. Our results suggest that the presence of some metal ions affects oligomerization reactions of glucose. We observed metal-specific effects, and it is likely that different metal-related effects are dominating for different metal ions. Our results imply that the effect of each metal ion varied depending on the tested metal. This strongly suggests that metal-glucose interactions are dictated by the chemical nature of the metal ion and the environmental conditions. While we used glucose as a model sugar for testing the effect of metal ions on abiotic sugar oligomerization reactions, it is reasonable to assume that metal ions will exhibit sugar- and metal-specific effects as well. Our systematic study underlines two crucial elements regarding the chemical evolution of sugars and metal ions: the complex nature of these reactions and their apparent specificity. Both are undisputedly relevant in today’s biology and perhaps provide the first step toward understanding of sugar-metal evolutionary interdependence.

## Methods

### Materials

All chemicals and reagents were of analytical grade, including α-D-glucose (Sigma-Aldrich, #158968), cellobiose (Sigma-Aldrich, #22150), isomaltose (Angene, #AG003R3Y), maltotriose (Sigma-Aldrich, #851493), isomaltotriose (Fischer Chemical, #J64186), lithium chloride (LiCl) (Sigma-Aldrich, #310468), sodium chloride (NaCl) (Sigma-Aldrich, #S9888), potassium chloride (KCl) (Sigma-Aldrich, #746436), magnesium chloride hexahydrate (MgCl_2_·6H_2_O) (Merck, #1.05833), calcium chloride dihydrate (CaCl_2_·2H_2_O) (Sigma-Aldrich, #C8106), zinc chloride (ZnCl_2_) (Sigma-Aldrich, #793523), manganese chloride(II) (MnCl_2_) (ACROS, #271410250), iron chloride (II) (FeCl_2_) (Sigma-Aldrich, #372870), aluminium chloride (AlCl_3_) (Rhenium, #217461000), D-Glucose-^13^C6 (Labsuite Shop, #AA008SJZ), cation ion-exchange resin (Sigma-Aldrich, #546976), 3-(trimethylsilyl)-1-propanesulfonic acid-d6 sodium salt (Sigma-Aldrich, #613150), D_2_O (ZEOtope, #ZEO-301496), Dimethyl sulfoxide-d6 (DMSO) (ZEOtope, #300372), sodium hydroxide (NaOH) (Sigma-Aldrich, #S8045), and hydrochloric acid (HCl) (Sigma-Aldrich, #320331). Acetonitrile was of LC-MS grade. Purified water (resistivity <18 MΩ cm) was obtained by the MilliQ purification system.

### Single-step dry-down reactions

Single-step dry-down reactions were performed to form oligosaccharides in the presence or absence of metal ions. To that end, aqueous solutions of glucose were dried in the presence or absence of metal ions at a 1:1 molar ratio (50 µmol each) in open microtubes (1.5 mL) at 50 °C for 1 week. Reactions were carried out under acidified (with the addition of 2.5 µmol HCl) or more basic (with the addition of 2.5 µmol NaOH) conditions. Prior to analysis, the single-step dry-down reactions were resuspended in water to give a 100 mM concentration, referring to the original monosaccharide and metal concentration prior to evaporation. The solutions were then vortexed and sonicated on ice before being diluted to the specified concentration for further analysis, as described below. All product sugar mixtures were fully soluble in water, except for the mixtures containing glucose and calcium in acidic conditions, for which only the supernatant was analyzed post-centrifugation. Reactions that involved Fe^2+^ were carried out in a Coy chamber under anaerobic conditions (95%/5% N_2_/H_2_).

### ATR-FTIR analysis

Attenuated total reflectance Fourier transform infrared (ATR-FTIR) analyses were done using Jasco ATR PRO 4X (Japan). For each sample, 45 μL overall (firstly, 25 μL of solution were taken, and after one hour, 20 μL of solution were taken again) were placed on Durapore® hydrophobic PVDF membranes with a pore size of 0.22 μm or on aluminum foil. The samples were dried in a hood overnight prior to analysis. The membranes were then placed on an ATR sample chamber for spectra collection. Spectral signals were averaged (60 scans per spectrum) and ranged from 400 to 4000 cm^−1^.

### Direct inject mass spectrometry

ESI-mass spectrometry data were collected using Agilent G6135 single quadrupole. For the analysis of dried samples via direct inject mass spectrometry, dried product solutions were diluted to 1 mM (referring to the original monosaccharide and metal concentration). As a control, fresh samples (prior to dry-down) were injected. The samples were injected into a mass spectrometer with the following parameters. Solvent: water with a flow rate of 5 mL/min. Injection volume: 10 μL. Capillary Voltage 4000 V. Scan range: 50–1500 m/z. MS data were processed using a suite of macros in Igor Pro8.0. For each type of species in positive mode, a set of *m/z* values was defined according to Table S2.

### Liquid chromatography-mass spectrometry

LC-MS analysis was performed using an Agilent 1260 quaternary pump and an autosampler with a DAD UV-Vis detector, coupled to an Agilent G6135 single quadrupole. Samples (either 1 mM or 10 mM, referring to the original monosaccharide and metal concentration) were injected onto an Agilent Poroshell 120 EC-C18 column. Column was held at 20 °C. Solvent A: water (0.1% formic acid); solvent B: ACN. Flow rate: 0.3 mL/min. Acquisition method: (A%) 100% (0–4 min), linear-gradient (A%) 78.6% to 77% (4–14 min), followed by wash linear-gradient (B%) 23% to 100% (14–21 min), (B%) 100% (21–28 min). Injection volume: 10 μL. Capillary voltage: 4000 V. Fragmentor voltage of either 70 V (for Scan mode) and 135 V (for SIM mode).

For SIM, mass values of each analyte (from glucose monomers up to glucose nonamers) were defined. For each analyte with exact mass *M*, three ionization species were considered: [M − H]^−^, [M + Cl]^−^ and [M + HCOO]^−^, as specified in Table S3. In total, 27 masses were monitored. Individual SIM chromatograms of each ionized species and a TIC-SIM chromatogram of all target masses were recorded. Analysis of oligomer formation was carried out by integrating the corresponding peaks in SIM chromatograms of each ionized species. For each species (a monomer or an oligomer), a total peak area was calculated, and distribution profiles of either absolute peak area or relative peak area were constructed.

Relative peak areas (RA_i_) were calculated as follows:

$${{RA}}_{i}=\frac{{A}_{i,{[M-H]}^{-}}+{A}_{i,{[M+{Cl}]}^{-}}+{A}_{i,{[M+{HCOO}]}^{-}}}{{\sum }_{i=1}^{n}{A}_{i,{[M-H]}^{-}}+{A}_{i,{[M+{Cl}]}^{-}}+{A}_{i,{[M+{HCOO}]}^{-}}}\times 100 \%$$

Where A_i[M − H]_^−^, A_i[M + Cl]_^−^ and A_i[M + HCOO]_^−^ are the peak areas of product *i* obtained for ionized species [M − H]^−^, [M + Cl]^−^ and [M + HCOO]^−^, respectively. *i* is an integer between 1 and 9 that indicates the specific product (monomer, dimer, trimer, etc).

Cumulative profiles were constructed as well, considering the total contribution of product *k* in a cumulative manner:

$${{CA}}_{k}={\sum}_{i=1}^{k}{{{\mathrm{RA}}}}_{i}$$

$$0 < \mathop{\sum}_{i=1}^{k}{{{\mathrm{RA}}}}_{{{{\rm{i}}}}}\le 100 \%$$

Where CA_k_ is the cumulative area of species *k*, which is the sum of RA_i_ from *i* = 1 until *i* = *k*. For example, CA_3_ is cumulative area of trimers, which is the sum of relative peak area obtained for monomers (*i* = 1), dimers (*i* = 2), and trimers (*i* = 3) species. Reverse cumulative profiles were also constructed considering the sum of RA_i_ from *i* = 9 to *i* = k in a reverse order. For example, CA_7_ represents the cumulative area of nonamers (*i* = 9), octamers (*i* = 8), and heptamers (*i* = 7).

To evaluate the feasibility of glucose quantification by SIM-LC-MS, glucose calibration standards were prepared at concentrations of 2.5–17.5 μM and analyzed by LC-MS in SIM mode under negative ionization conditions using fragmentor voltages of 70 V and 90 V. For glucose, the m/z values corresponding to the ionization species [M − H]⁻ and [M + HCOO]^−^ were monitored, and the summed peak areas of these two ions were used for quantification. Calibration curves were constructed by plotting the total peak area as a function of glucose concentration. Independently prepared quality-control samples at 2.5 and 10 μM were analyzed in the same manner to assess reproducibility. Dry-down glucose control samples were rehydrated to 100 mM glucose, with concentration referring to the initial glucose concentration prior to reaction, and were further diluted 500-fold or 1000-fold to fit within the calibration range.

### Statistical significance

Statistical significance was determined using Student’s *t* test, assuming a two-tailed and unequal variance.

### Nuclear magnetic resonance

150 µL of dried samples or 30 µL of fresh samples from a 0.1 M aqueous solution were lyophilized overnight. Lyophilized samples were dissolved in 588 µL of phosphate buffer with pH 2.66 in D_2_O and 12 µL of standard (3-(trimethylsilyl)-1-propanesulfonic acid-d6 sodium salt) with a proton concentration of 250 mM (27.8 mM of standard). For samples containing glucose and either Mn^2+^ or Fe^2+^, pre-treatment was carried out for direct removal of metal ions due to interference with the NMR signal due to their paramagnetic nature. Specifically, 60 µL of dried samples or 40 µL of fresh samples (0.1 M aqueous solutions) were incubated in water (3.96 mL for fresh samples or 5.94 mL for dried samples) and mixed with cation-exchange resin (approximately 0.8 g per sample) for 30 min. Following incubation, the sample supernatant (3 mL from fresh samples or 4.5 mL from dried samples) was collected and lyophilized overnight. Lyophilized samples were dissolved in 588 µL of DMSO-D_6_ and 12 µL of standard (3-(trimethylsilyl)-1-propanesulfonic acid-d6 sodium salt) with a proton concentration of 250 mM. Samples were analyzed using either a Bruker 500 MHz Neo spectrometer, a 400 MHz Neo spectrometer, or a 500 MHz Avance II spectrometer.

For quantitative ^1^H analysis of glucose, a 90-degree proton pulse sequence was used with a delay time of 26 s (~10T1) at 20 °C. Sixteen scans were taken per sample. The longest T1 detected was 2.7 s. For quantitative ^1^H analysis of samples containing glucose and either Mn^2+^ or Fe^2+^, a 90-degree proton pulse sequence was used with a delay time of 8 s (~10T1) at 25 °C. Sixteen scans were taken per sample.

Due to interference for some of the anomeric signals in drying reactions, to improve accuracy we integrated only half of each anomeric monomeric signal doublet in dried samples (and multiplied by two). We used the internal standard for calibration of integration and chemical shifts. Moreover, since at 25 °C the HOD peak strongly overlaps with the β-anomeric signal, we employed measurements at 20 °C for all samples, in which the HOD resonance shifts downfield, thus improving the accuracy of our quantification method. The full spectra and relevant integrals of all samples are shown in the Supplementary file “NMR spectra integrations.”

The conversion was calculated as follows:

$$\% \,{conversion}=\left(1-\frac{{I}_{{dd}}}{n* {I}_{f}}\right)* 100 \%$$

Where: $${I}_{f}=\frac{{I}_{\alpha }+{I}_{\beta }}{{I}_{{standard}}}$$; $${I}_{{dd}}=\frac{{I}_{\alpha }+{I}_{\beta }}{{I}_{{standard}}}$$

${I}_{f}$ - integrated sum of anomeric peaks for fresh samples

${I}_{{dd}}$ - integrated sum of anomeric monomeric peaks for dried-down samples

*n* – dilution factor between fresh and dried samples, where $n=\frac{{C}_{{dd}}}{{C}_{f}}$ (C-concentration; for samples in D_2_O *n* = 5; for samples in DMSO *n* = 1.5)

For experiments involving standards of glucose oligomers (cellobiose, isomaltose, maltotriose, and isomaltotriose), 0.1 M aqueous stock solutions were prepared. For analysis of standards alone, spectra were collected at a standard concentration of 5 mM in phosphate buffer with pH 2.66 in D_2_O. For spiking experiments, spectra were collected in mixtures containing 50 µL of dried samples (0.1 M aqueous glucose solution, referring to the initial monomer concentration prior to drying reaction) and oligomer standard (5 µL, final concentration 0.83 mM) in phosphate buffer with pH 2.66 in D_2_O.

For kinetic analysis, fresh (non-dried) samples of _D_-Glucose and CaCl_2_ at a ratio of 1:1 under acidified conditions were prepared in water, followed by lyophilization. The dried substance was dissolved in D_2_O buffer (pH = 2.66), and ^1^H-NMR spectra were immediately recorded for 250 min at 30-minute intervals.

For ^13^C and HSQC analysis, dried samples of isotopically labeled _D_-Glucose-^13^C6 were used. For the ^13^C analysis, 128 scans were acquired, while for HSQC, two scans were performed.

## Supplementary information

Transparent Peer Review File

Supplementary Information

Description of Additional Supplementary Files

Supplementary Data

## Data Availability

The data supporting the findings of this study are available within the article and its Supplementary Information. Source data are provided as a Source data file. Additional raw data, including NMR spectra and mass spectrometry data, are available from the corresponding author upon reasonable request.
