# Supplementary material for: Metal ions affect abiotic sugar oligomerization reactions
Source: Commun Chem. 2026 Jul 13;9:345. doi: 10.1038/s42004-026-02129-w (PMC13737047; doi:10.1038/s42004-026-02129-w)
Supplement: Supplementary file 1 [file 42004_2026_2129_MOESM1_ESM.pdf]

nature portfolio

Peer Review File

Metal Ions Affect Abiotic Sugar Oligomerization Reactions

Corresponding Author: Dr Moran Frenkel-Pinter

This file contains all reviewer reports in order by version, followed by all author rebuttals in order by version.

Attachments originally included by the reviewers as part of their assessment can be found at the end of this file.

Version 0:

Reviewer comments:

Reviewer #1

(Remarks to the Author)
The article by Akulenko et al aims at exploring how metal ions affect the oligomerization of glucose in a prebiotic context. Metal ions have been used to affect many other prebiotic reactions, but the oligomerization of sugars has been scarcely investigated in origin-of-life research (only reference 37). Sugar polymers are important in current biochemistry, and the hypothesis that understanding their interaction with metal ions would open new research avenues in the field is reasonable. Exploration of prebiotic roles of oligosaccharides would be interesting, for instance to address potential cooperation with peptides or nucleic acids towards relevant biological functions. The introduction sets this scenario quite appropriately, it is well-written and well-supported by references. However, from the experimental point of view and, mainly, the interpretation of results, the paper has various major problems that hinder its publication, at least in the current state. I list some of them below:

1) Page 4, first paragraph of results and discussion: How did the authors perform the sample drying under anaerobic conditions (for the case with Fe^{2+})? It is not clear from the text, and details of the atmosphere employed should be given: nitrogen, argon? Besides, why not using anaerobic conditions in all cases, since this would be a more prebiotic scenario?

2) Second section of results and discussion: For quantification of conversion, it is not clear at all which signals have been integrated, neither from Figure 3 nor from the rest of spectra in the SI. The authors mention that they are monitoring signals from the anomeric carbon, but in the blue spectra there are quite some new signals in the corresponding region. Are they integrating all? How are they sure all new signals correspond to oligomers and not to other degradation products? This part of the manuscript is slightly obscure. The relevant integrals should be shown in the spectra of all samples, together with a proper justification of why they correspond to oligomers rather than degradation compounds.

An additional problem is that the solvent signal appears in the same region, making impossible a precise quantification. This makes the results of Figure 4 quite uncertain.

Or is it that the conversion was just calculated with the remaining monomer concentration? In any case, it is not evident whether the majority of products that are being formed are oligomers or something else. Please take into account that bare metal ions tend to degrade biomolecules quite easily, when they are not protected in the coordination sphere of a coordination complex or a metalloenzyme.

3) Page 5, last paragraph: This paragraph is highly speculative. The interaction of each specific metal ion with the monomer could be different. I am not sure a general conclusion for all metals like this one can be really drawn. The electronic structure of the metal can also affect a lot (not only hindering acetal formation due to coordination to the anomeric O), and even Lewis acid-catalysis might be possible in some cases. The authors go too far here, specially taking into account the limitations of their quantification method.

4) Page 5, last paragraph: “The results of 13C-NMR and HSQC supported our former 1H-NMR results: acidified samples contained more oligomeric peaks compared to samples to which NaOH was added.” How do the authors know these are oligomer peaks? Have they compared with the spectra of some glucose oligomer standard?

5) LC-MS studies: I don´t really understand the data interpretation in this section? How is it that pentamers, hexamers,

heptamers, octamers and nonamers elute all at the same retention time in the chromatograms of Fig. S117-S125? If so, the chromatographic method (or the column) is not adequately chosen. Then trimers and tetramers give multiple peaks over a RT range of ca. 5 or 6 min. Why is it so?

Under these circumstances, the variations in peak areas between samples, which are not dramatic (as the authors claim at the end of the introduction) but quite minor (with the exception of Fe^{2+}), are not really significant. Indeed, with the current data I cannot be sure of which species they are really looking at through LC-MS and NMR. Further justification would be required.

Reviewer #2

(Remarks to the Author)
The paper by Akulenko et al. describes the effect of metal cations on the polymerization and isomerization of glucose. The authors use a plausible prebiotic setting, drying conditions, 50 ˚C, 1 week incubation, to trigger the oligomerization chemistry. The product mixtures are analyzed by a multitude of experimental techniques, including FTIR, NMR, ESI-MS, LC-MS for acidic as well as alkaline conditions and for a series of metal cations, including alkali, alkaline earth as well as first row transition metals. A comprehensive characterization of both the oligomerization products and cation-glucose binding is given, which is documented in a perfectly elaborated 127 pages long supplementary material.

I appreciated the effort of the authors to enter the so-far untouched area of prebiotic carbohydrate oligomerizations and especially that they directed their attention to the perhaps hardest problem in this topic, i.e. the effect of metal cations on this chemistry. Indeed, the study clearly demonstrates that metal cations may change the outcome of carbohydrate polymerization reactions dramatically, in addition, in a cation-specific manner. Considering the depth and highly professional realization of the work, I think this study will represent a seminal contribution to the prebiotic chemistry field and will form the foundation of future studies related to the origin of polysaccharides that is an important class of terrestrial biomolecules. For this reason, I strongly recommend publication of this work in Communications Chemistry even in its present form. Optionally, the authors might want to consider the following suggestions:

The authors give a very logical explanation for the inhibitory effect of Mn^{2+} and Fe^{2+} on page 5, last paragraph, attributing it to the binding of the anomeric OH to the metal cations. I think it would help the readers if the authors could illustrate this effect with a schematic drawing in the supplementary material.

Though the list of metal cations considered is very comprehensive from a prebiotic chemistry point of view, to my taste a very specific metal cation, Al^{3+}, is missing from the list. Al^{3+} is actually the most abundant metal cation in the Earth crust, and is a common component of silicate rocks. In addition, Al^{3+} is a group 3A element, the neighbor of boron, an element that is believed to play an important role in the stabilization of sugars in the prebiotic mix. It would be great if the authors could devote a couple of words to Al^{3+} in their discussion.

Reviewer #3

(Remarks to the Author)
In this manuscript, the authors present extensive work aimed at elucidating the potential role of several metal cations in the oligomerisation of sugars under plausible prebiotic conditions. Two series of experiments were conducted under acidic and basic pH values, respectively, and under drying conditions, using up to eight chloride salts containing metals considered abundant on the primitive Earth. All experiments were performed in triplicate, demonstrating good reproducibility. Glucose was selected as the model sugar, based on the only previous study that addressed a potential prebiotic synthesis of oligosaccharides. Several analytical techniques were employed for the characterisation of the final reaction mixtures, including ATR-FTIR, 1H and 13C NMR spectroscopy, ESI-MS, and LC-MS. A large amount of data was analysed to determine the influence of the initial pH and the presence of metals on both the conversion of glucose and its transformation into various oligosaccharides. Although this work may be relevant to the field of prebiotic chemistry, given the very limited number of studies concerning the oligomerisation of polysaccharides and their important role in contemporary biochemistry, several inconsistencies and details, must be more thoroughly addressed before the manuscript can be considered for publication in Communications Chemistry.

Main comments

1) The main focus of this paper is the conversion of glucose into its corresponding oligomers in the presence or absence of metal cations. It is somewhat surprising that, despite having access to a powerful analytical tool such as an LC–MS system coupled with a DAD UV–Vis detector, the authors did not take fuller advantage of this instrumentation. There are several commercially available maltodextrin and isomaltose oligosaccharide standards that could have been used to develop and validate an accurate analytical method for the proper quantification of both glucose consumption and oligosaccharide production. The following references provide useful examples that could have guided the authors in designing a suitable analytical method for quantifying oligomers derived from glucose: Carbohydrate Research 257 (1994) 249–268; Journal of Chromatography 207 (1981) 439–444; Chromatographia 50 (1999) 705–710; Z. Lebensm. Unters. Forsch. A 206 (1998) 175–178; and http://dx.doi.org/10.2139/ssrn.5413053. Since the stereochemistry of the oligomers is not a central aspect of this study, which focuses primarily on the production and chain length of the oligosaccharides, the use of external standards would have enabled the construction of calibration curves and an accurate quantification of the oligomers formed in the

presence of each metal cation. This methodology would allow a more meaningful comparison between experiments and support more robust overall conclusions.
2) The quantification of glucose consumption by 1H NMR is a rather complex methodology, which is described in detail only in the previous OLEB 2019. In that paper, the quantification of glucose conversion was clearly explained, and the NMR spectra were carefully interpreted, with comprehensive assignments of both hydrogen and carbon atoms based on one- and two-dimensional experiments. However, in the present manuscript, the NMR results are only superficially described. Rough assignments of 1H and 13C signals are provided, and the approach used for quantifying glucose is not clearly defined. Although numerous NMR spectra are presented, the information extracted from them is not clearly shown and the results are not fully conclusive. For clarity and conciseness, the large number of NMR spectra (Figures S50–S89) could be grouped into four summary figures corresponding to the four experimental series: (i) fresh samples – acidic conditions; (ii) fresh samples – basic conditions; (iii) Dry-down samples – acidic conditions; and (iv) Dry-down samples – basic conditions. Each figure could display nine representative spectra, following the layout used in the OLEB 2019 paper (for instance, Figures S31c, S32a, and S32c).
3) The ESI–MS data appear to have been treated somewhat carelessly, which may lead to inaccurate or misleading conclusions. Please unify the m/z scale (from 50 to 1500) across all mass spectra (Figures S19–S54) to allow consistent comparison. In addition, the mass peak labelling should be carefully revised and correctly positioned. For example, in Figure S25a, a peak around 600 amu is assigned to 7Glu + Ca, whereas this species should have a molecular mass of approximately 1193 amu. Similar inconsistencies are observed in Figure S27a. Although I have not examined every spectrum in detail, the authors are strongly encouraged to perform a thorough revision of all the mass spectra. Furthermore, it is unclear why nonamers are identified by LC–MS but not by ESI–MS. The authors should clarify this discrepancy and specify the limits of detection for both techniques.
4) I believe that the assignment of the oligomers by LC–MS was based on the SIM mass spectra; however, why do the species from the tetramer to the nonamer appear at the same retention time (Fig. 5a and Figs. S117–S125)? In line with the above comments, why were commercial standards not used for the calibration of the LC–MS analysis? On the other hand, in any case the conversion of glucose seems to be incomplete. The absence of a relative intensity scale in Figs. S118–S125 makes it difficult to interpret the data properly. What is the final concentration of glucose with respect to its oligomers? Moreover, using a logarithmic scale in Figs. 5b–5e would allow a clearer visualisation of the semiquantitative analysis of the different oligomers shown in the same graphs, especially if points rather than bars were used. In fact, these four figures could be conveniently summarised in a single one by employing a logarithmic scale.
5) The ATR–FTIR spectra are presented in a rather inconsistent manner throughout the manuscript. The quality of the recorded spectra should be improved, and the number of figures reduced, as suggested above for the 1H NMR data. After carefully examining all the FTIR spectra, I was unable to identify any discernible features around 1050 cm^{-1}. Likewise, in Figure 2a of the main text, the assignment of the FTIR bands is neither clearly explained nor properly marked. The bands are neither indicated on the spectra nor adequately discussed. The authors are therefore encouraged to enhance both the quality and the clarity of the FTIR data, ensuring that all relevant spectral bands are correctly identified and assigned. Alternatively, if the FTIR results do not provide significant or complementary information regarding the formation of oligomers —particularly when compared with the data obtained from NMR (if they would properly described) and ESI–MS—this section could reasonably be omitted from the manuscript.
6) What is the initial pH of each experiment? This is a very important point. The oligomerization of glucose is an acid-catalyzed process, as previously demonstrated and indicated in the present manuscript. A low pH favors the formation of oligomers. However, not all the salts used are neutral, and therefore the initial pH in each of the two experimental series (HCl and NaOH) may differ for the various salts. For a better interpretation of the results, Figure S55 and the associated discussion should consider the increase in pH rather than the absolute final pH value of each experiment. Thus, please indicate the initial and final pH values for each experiment, as well as the corresponding pH increase.

Minor comments

1) The manuscript could be rewritten to help the reader better appreciate the significance of the results. There is a large amount of data, but even for a specialised reader it is not easy to extract its full value. I believe that the Results and Discussion section should be reorganised. For example, the results obtained by each technique could be presented separately (technique by technique), followed by a comparison of the information provided by each of them, a discussion of the internal consistency of all the data, and finally a comparison with the only previous study on glucose polymerisation. Finally, a especific section could then address the mechanistic aspects of oligomerisation (coordination with metal cations, influence of pH, pH variations during the reactions and so on).
2) I have noticed some differences in the glucose conversion values obtained in the control experiments without salts, compared with those reported in the previous paper. Please place these differences in context within the discussion.
3) What is the reason for the change of solvent used to record the NMR spectra (DMSO for the $FeCl_2$ and $MgCl_2$ experiments)?
4) The introduction is well referenced; however, it should be reorganised. For example, on page 2: “While abiotic polymerisation of peptides and nucleic acid polymers has been studied extensively,21–36 abiotic oligosaccharide polymerisation was reported only once, in drying reactions of glucose.37”and on page 3: “In the context of prebiotic chemistry, metal-catalysed peptide and RNA bond formation has been demonstrated.21,51–53,56–65 […] While it is now known that oligosaccharides can form abiotically,37 it is unknown whether metal ions have any effect on the polymerisation of sugars.” These ideas should be unified for better coherence. Additionally, in the introduction, the following paragraph seems more appropriate for the Conclusions section: “We found that while metal ions typically inhibited the overall conversion of glucose, some promoted the formation of longer oligosaccharides. These include Fe^{2+}, K^{+}, Mg^{2+}, and Li^{+}. For example, while glucose conversion was ~67% in the presence of Fe^{2+}, compared to ~86% in its absence, it led to a significant increase in the abundance of octamers (by ~1.7-fold) and nonamers (by ~2.3-fold). The Ca^{2+} ion, on the other hand, inhibited the formation of longer oligomers.”

Version 1:

Reviewer comments:

Reviewer #1

(Remarks to the Author)
I can see that in this revised version the authors have made a significant effort to improve the analytical aspects of their work. Their responses to my comments are convincing, and the paper now shows much more consistent data analysis supporting their conclusions, and a better balanced interpretation of the results. Given that the relevance of the results is clear, I am happy to see that the work can be published in Commun. Chem.

Reviewer #2

(Remarks to the Author)
The authors carried out a very careful revision, in which they provide convincing new data for the effect of A3+ cations on the studied polymerization reaction. For this reason I strongly suggest publication of this fine work in Communications Chemistry in its present form.

Reviewer #3

(Remarks to the Author)
The authors have made a significant effort to improve the manuscript and have performed additional experiments and analyses. Despite these improvements, several points still require clarification, further detail, and refinement for the work to merit publication in Communications Chemistry. The volume of experimental work is huge, clearly representing many hours of effort, and deserves to be presented in a manuscript of commensurate quality.

Below, I provide my comments following the order of the manuscript:

Results and Discussion. Section 1
1) In the opening paragraph of Section 1, it would be helpful to clearly state that two experimental series were conducted: one under acidic conditions and one under basic conditions, along with the rationale for selecting these experimental setups. Additionally, in this revised version, the authors have carried out further experiments at an initial concentration of 10 mM to complement the 1 mM SIM-LC-MS analyses from the original submission. The motivation for this expanded experimental scope should also be briefly introduced in this first paragraph.
2) If feasible, and without requiring excessive additional work (given the already extensive number of figures in the Supporting Information), it would be beneficial to maintain a consistent color scheme across Figures S2–S20.
3) In Figure S61, the caption should specify how glucose conversion was calculated, in order to facilitate the reader's understanding of both the methodology and the results.
4) At the end of this section, the authors state: "We also detected sugar–metal ion adducts via MS in fresh, non-dried samples." Please provide specific examples of the adducts identified in this study and explain these results in greater detail. As currently written, the paragraph is rather opaque and difficult to follow. A clearer and more detailed discussion, including representative examples and an indication of whether adduct formation depends on the initial pH, is needed. While the ESI-MS results obtained in negative ionization mode are reasonably well explained, the discussion of the positive ionization mode is not. Please improve the clarity and depth of the discussion in this regard.

Results and Discussion. Section 2
5) At the end of the first paragraph, the authors state: "Notably, we acknowledge that integration of the anomeric protons is an underestimation of glucose conversion due to a partial overlap between the signals of the reducing end of glucose monomers and glucose oligomers." This underestimation is in fact demonstrated when glucose conversion is quantified by LC-MS. The limitations of NMR-based conversion estimates compared to LC-MS measurements are very well explained in the response-to-reviewers letter, as is the impracticality of using LC-MS to determine glucose conversion for all experiments. I strongly encourage the authors to include this information in the manuscript—briefly in the main text and in greater detail (as already done in the response letter) in the Supporting Information.
6) Figure 4 is not adequately discussed.
Building Figures 4a and 4b required at least 60 experiments and 60 1H NMR spectra, an effort that warrants a thorough analysis and the extraction of meaningful conclusions. In its current form, neither the text nor the figures achieve this. Please perform a statistical analysis to determine whether the differences in glucose conversion across experiments are statistically significant relative to the control. Furthermore, consider whether certain cations produce similar effects on conversion and whether they can be grouped based on statistically significant similarities.
Figure 4c is of limited value, as it only presents mean values. It is already apparent that basic conditions lead to lower glucose conversion. Instead, consider performing a statistical comparison between the acidic and basic series. A proper statistical treatment would significantly strengthen the robustness of the conclusions.
Figure 4d does not meaningfully contribute to the overall discussion unless it is explicitly connected to mechanistic implications regarding metal–sugar complex formation. In addition, the figure presents the electronic configurations of neutral elements rather than their ionic forms.
7) Why is the initial pH for the Al^{3+} experiments set to 4 after the addition of HCl or NaOH? This point is critical for explaining

the observed results regarding glucose conversion in the presence of $AlCl_3$ and likely in the generation of oligomers.

Results and Discussion. Section 3
8) I regret to say that I find the discussion of Figure 5 difficult to follow. In the second paragraph of this section, it appears that the authors are discussing cumulative peak areas rather than the figure itself. Please ensure that figures included in the main text are explicitly and clearly discussed in the main text. The Supporting Information already contains a large number of figures presenting cumulative area data. This paragraph should be carefully revised, as it is currently quite difficult to understand. While the dataset is extensive, it is not always clear which data and figures are being referred to. Increasing the number of figures in the main text would likely improve clarity. I encourage the authors to consider multi-panel figures that help the reader better visualize the results. The manuscript should not rely so heavily on general references to the Supporting Information while providing only a cursory discussion of the results.
9) In this section, the authors should also clearly explain why two sets of experiments were conducted at different concentrations (1 mM and 10 mM). What effect does this difference in concentration have on glucose oligomerization and in the extension of the oligomers? This point is not addressed in the main text, despite the substantial experimental effort and the additional Supporting Information figures it entails.

Results and Discussion. Section 4
10) The manuscript proposes reaction mechanisms and speculates on the possible formation of sugar–metal complexes; therefore, Figures S177 and S178 should be moved to the main text (e.g., as Figures 1c and 1d), clearly indicating their hypothetical nature. Please avoid further transferring key information to the Supporting Information.

In any case, I encourage the authors to further improve the writing of the main text and to increase the number of primary figures included in the manuscript. Additionally, the authors may wish to consider including their response-to-reviewers letters if the manuscript is ultimately published. These documents contain a wealth of experimental detail that helps ensure the traceability of the experiments and measurements performed. Moreover, the discussions of the limitations of the spectroscopic and chromatographic techniques, currently only addressed in the response letters, would be of considerable interest both to specialists and to non-specialists, particularly in the context of developing general analytical protocols.

Version 2:

Reviewer comments:

Reviewer #3

(Remarks to the Author)
The authors have made a substantial effort in the experimental work, data analysis, and the integration of these results into a thoroughly revised manuscript.

In light of these latest changes to the manuscript, I recommend its publication in Communications Chemistry.

Open Access This Peer Review File is licensed under a Creative Commons Attribution 4.0 International License, which permits use, sharing, adaptation, distribution and reproduction in any medium or format, as long as you give appropriate credit to the original author(s) and the source, provide a link to the Creative Commons license, and indicate if changes were made.
In cases where reviewers are anonymous, credit should be given to 'Anonymous Referee' and the source.
The images or other third party material in this Peer Review File are included in the article's Creative Commons license, unless indicated otherwise in a credit line to the material. If material is not included in the article's Creative Commons license and your intended use is not permitted by statutory regulation or exceeds the permitted use, you will need to obtain permission directly from the copyright holder.
To view a copy of this license, visit https://creativecommons.org/licenses/by/4.0/

Response to Reviewers

Reviewer #1 (Remarks to the Author):

Reviewer 1 comment 1. The article by Akulenko et al aims at exploring how metal ions affect the oligomerization of glucose in a prebiotic context. Metal ions have been used to affect many other prebiotic reactions, but the oligomerization of sugars has been scarcely investigated in origin-of-life research (only reference 37). Sugar polymers are important in current biochemistry, and the hypothesis that understanding their interaction with metal ions would open new research avenues in the field is reasonable. Exploration of prebiotic roles of oligosaccharides would be interesting, for instance to address potential cooperation with peptides or nucleic acids towards relevant biological functions. The introduction sets this scenario quite appropriately, it is well-written and well-supported by references. However, from the experimental point of view and, mainly, the interpretation of results, the paper has various major problems that hinder its publication, at least in the current state. I list some of them below:

Response to Reviewer 1 comment 1. We are happy that the reviewer recognizes the importance of the work and the justification as presented in the Introduction. We thank the reviewer for their comments and have addressed them as described below. We believe that these comments improved the clarity and the interpretation of our findings. Indeed, in the future, we plan to study the interactions between these sugar oligomers with other molecules (such as model peptides that we generate abiotically in our lab).

Reviewer 1 comment 2. 1) Page 4, first paragraph of results and discussion: How did the authors perform the sample drying under anaerobic conditions (for the case with Fe2+)? It is not clear from the text, and details of the atmosphere employed should be given: nitrogen, argon? Besides, why not using anaerobic conditions in all cases, since this would be a more prebiotic scenario?

Response to Reviewer 1 comment 2. Apologies, this was not clear. For anaerobic conditions (reactions involving Fe^{2+}), reactions were carried out in a Coy chamber (under 95%/5% N_2/H_2). We added the following to Materials and Methods:

> "Reactions that involved Fe^{2+} were carried out in a Coy chamber under anaerobic conditions (95%/5% N_2/H_2)".

Reactions that did not involve Fe^{2+} were carried out under an aerobic atmosphere only after we verified that glucose oligomerization reactions are similar under both aerobic and anaerobic conditions, as can be seen here (new Supplementary Fig. S1):

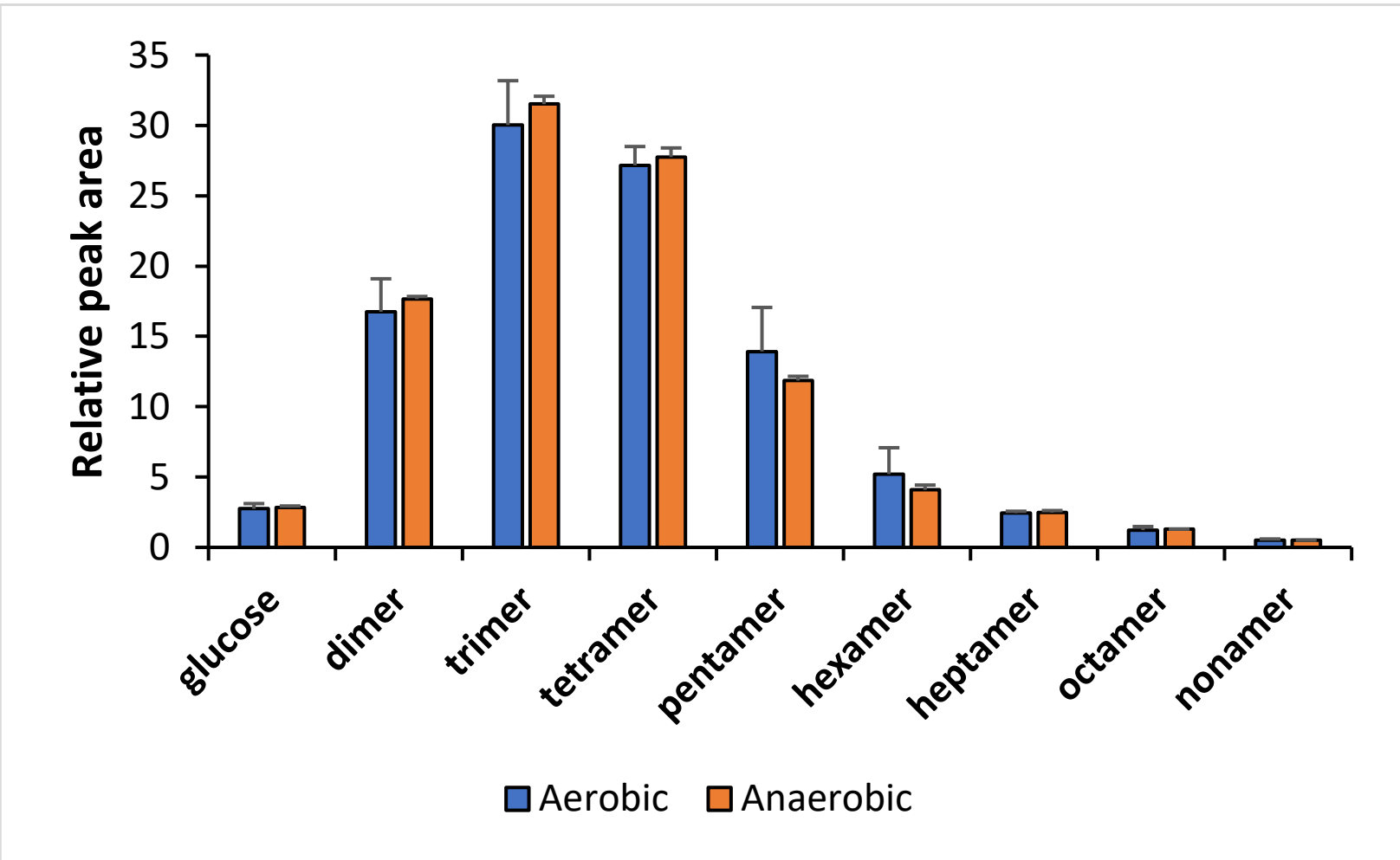

We do not expect any effect of the atmosphere on glucose oligomerization reactions for the other metal ions tested in our work (except for Fe^{2+}). To verify this further, we now carried out another reaction in which glucose was oligomerized in the presence of Zn^{2+} under anaerobic conditions, and indeed, no effect of the atmosphere was observed. Panel A shows a similar product distribution in the presence or absence of Zn^{2+} under aerobic conditions (original), and panel B shows a similar product distribution in the presence or absence of Zn^{2+} under anaerobic conditions (new):

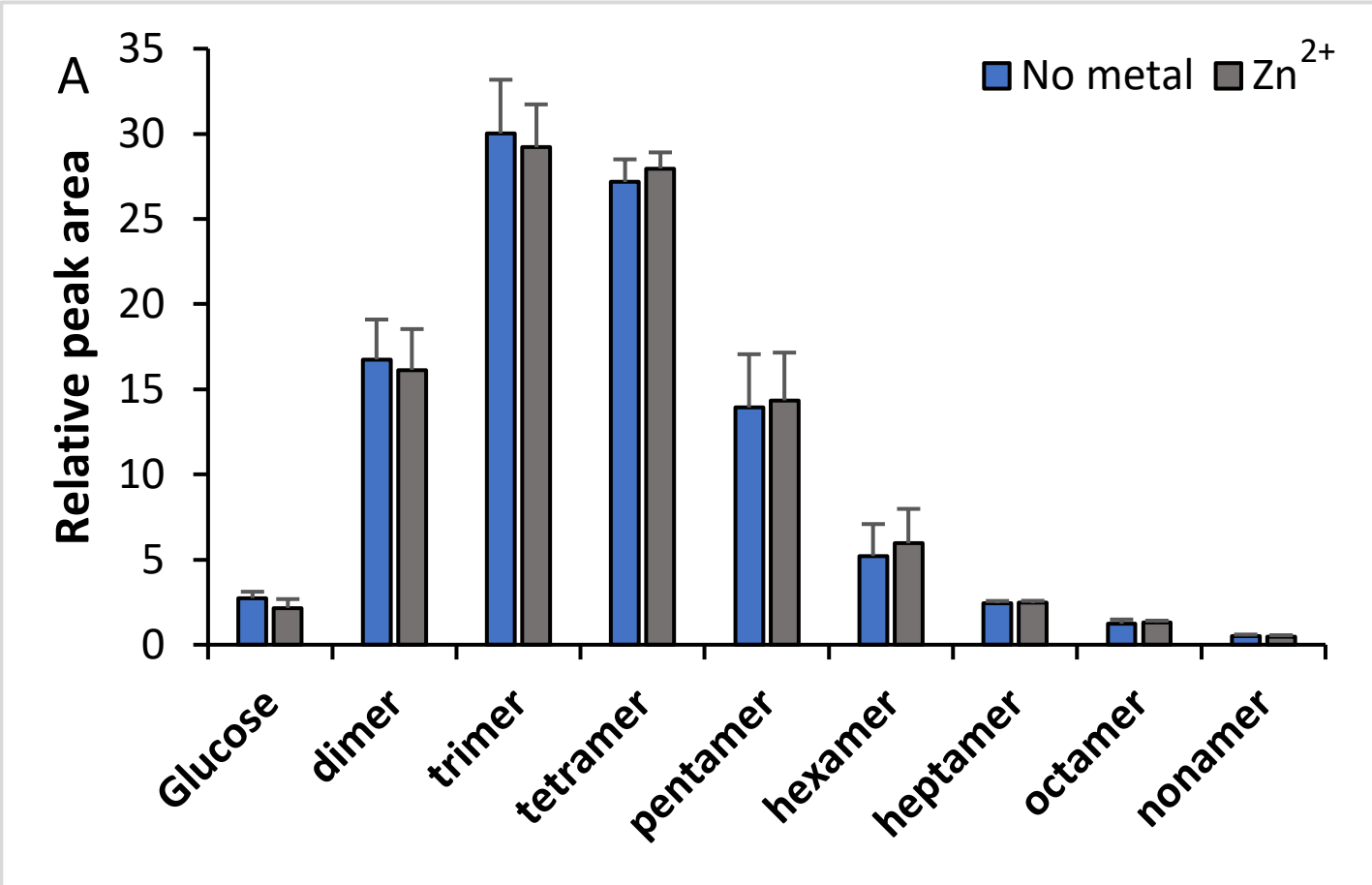

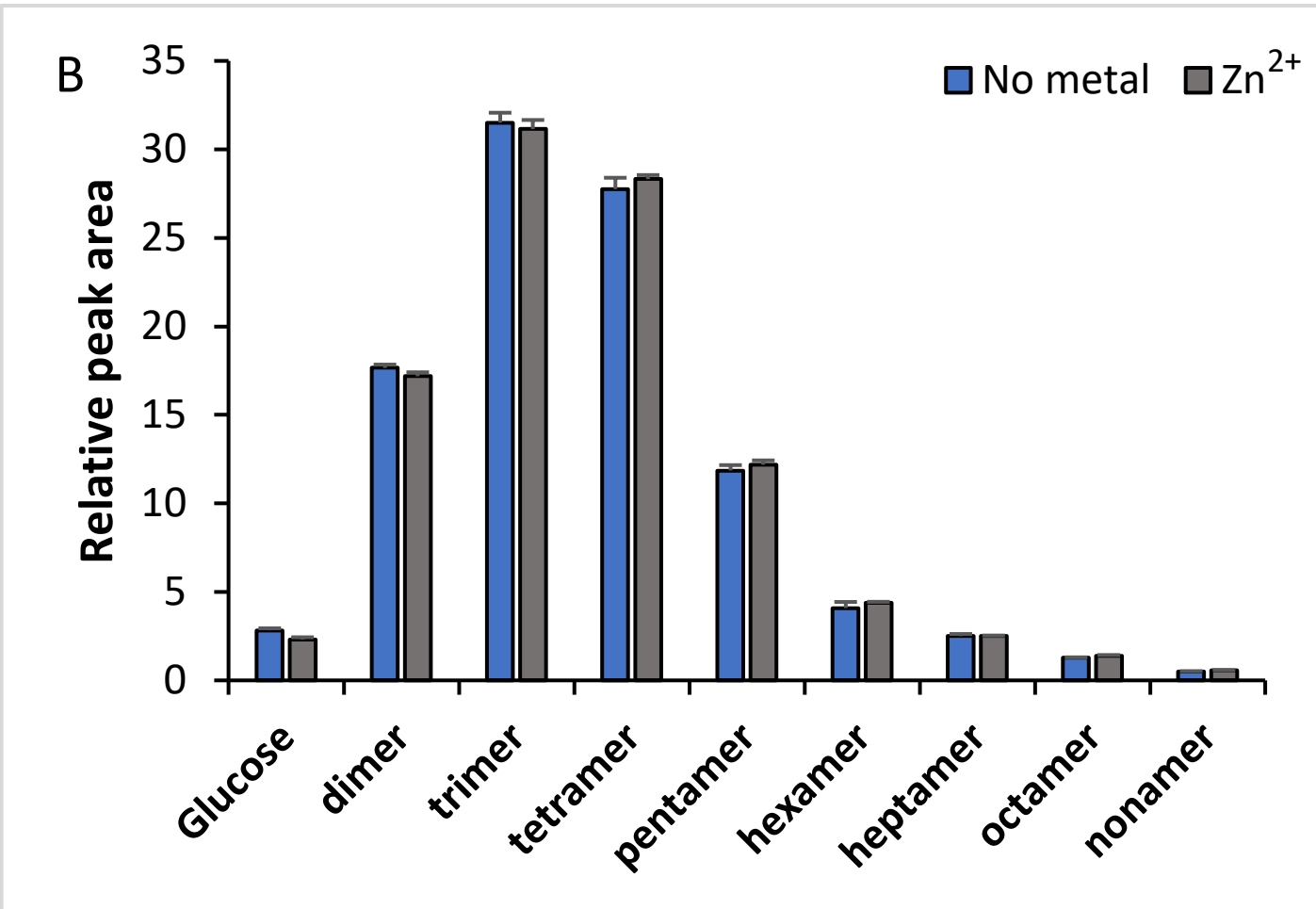

We now added this new experiment as Supplementary Fig. S1 and the associated text in Results and Discussion:

> "To verify that the atmosphere does not impact glucose oligomerization reactions for other metal ions, we performed a reaction in which glucose was oligomerized in the presence or absence of Zn^{2+} under anaerobic conditions, and indeed, no effect of the atmosphere was observed (Supplementary Fig. S1)."

To conclude, for systems that did not contain Fe^{2+}, we conducted the reactions under aerobic conditions, as these experiments are operationally simpler and our control experiments showed that the atmosphere does not measurably affect glucose oligomerization under these conditions.

Reviewer 1 comment 3. 2) Second section of results and discussion: For quantification of conversion, it is not clear at all which signals have been integrated, neither from Figure 3 nor from the rest of spectra in the SI. The authors mention that

they are monitoring signals from the anomeric carbon, but in the blue spectra there are quite some new signals in the corresponding region. Are they integrating all? How are they sure all new signals correspond to oligomers and not to other degradation products? This part of the manuscript is slightly obscure. The relevant integrals should be shown in the spectra of all samples, together with a proper justification of why they correspond to oligomers rather than degradation compounds.

Response to Reviewer 1 comment 3. This is a very good point and we have now clarified this. We integrated the anomeric proton signals using an internal standard, following Li *et al*. (originally ref 37). The two relevant peaks, at ~4.64 ppm and ~5.22 ppm, which correspond to the β- and α-anomeric hydrogens of glucose, respectively, were observed in D_2O. These peaks represent the reducing-end anomeric hydrogens, and changes in their integrals reflect glucose conversion during the reaction. We acknowledge that not all products correspond to oligomers, as elimination or isomerization side products may form (especially under basic conditions). Indeed, we observe some change of color upon dry-down of glucose samples. We modified the Results section as follows:

> "Notably, not all products correspond to oligomers, as elimination or isomerization side products may form (especially under basic conditions)."

> "Therefore, glucose oligomerization reactions result in a shift in the anomeric proton on the non-reducing end and allow us to estimate the extent of glucose conversion."

Importantly, we know that at least the majority of these new products under acidified conditions are oligomers, based on our MS analysis, published NMR references (e.g., Li *et al*, originally ref 37), as well as new NMR spectra of oligomer standards that we collected. Specifically, for this revision we ran ^{1}H-NMR of standards of various glucose dimers (cellobiose, isomaltose) and trimers (maltotriose, isomaltotriose). We also ran spiking experiments in which we added these standards to dried-down reactions of glucose in the absence and presence of Zn^{2+}. We clearly see some overlap between the new peaks that were observed in our experiments upon drying with these standards. Moreover, in some cases we observed a partial overlap between the oligomer standard with the reducing end of glucose monomer. This is an example of ^{1}H-NMR spectrum upon spiking of a dried glucose sample with cellobiose:

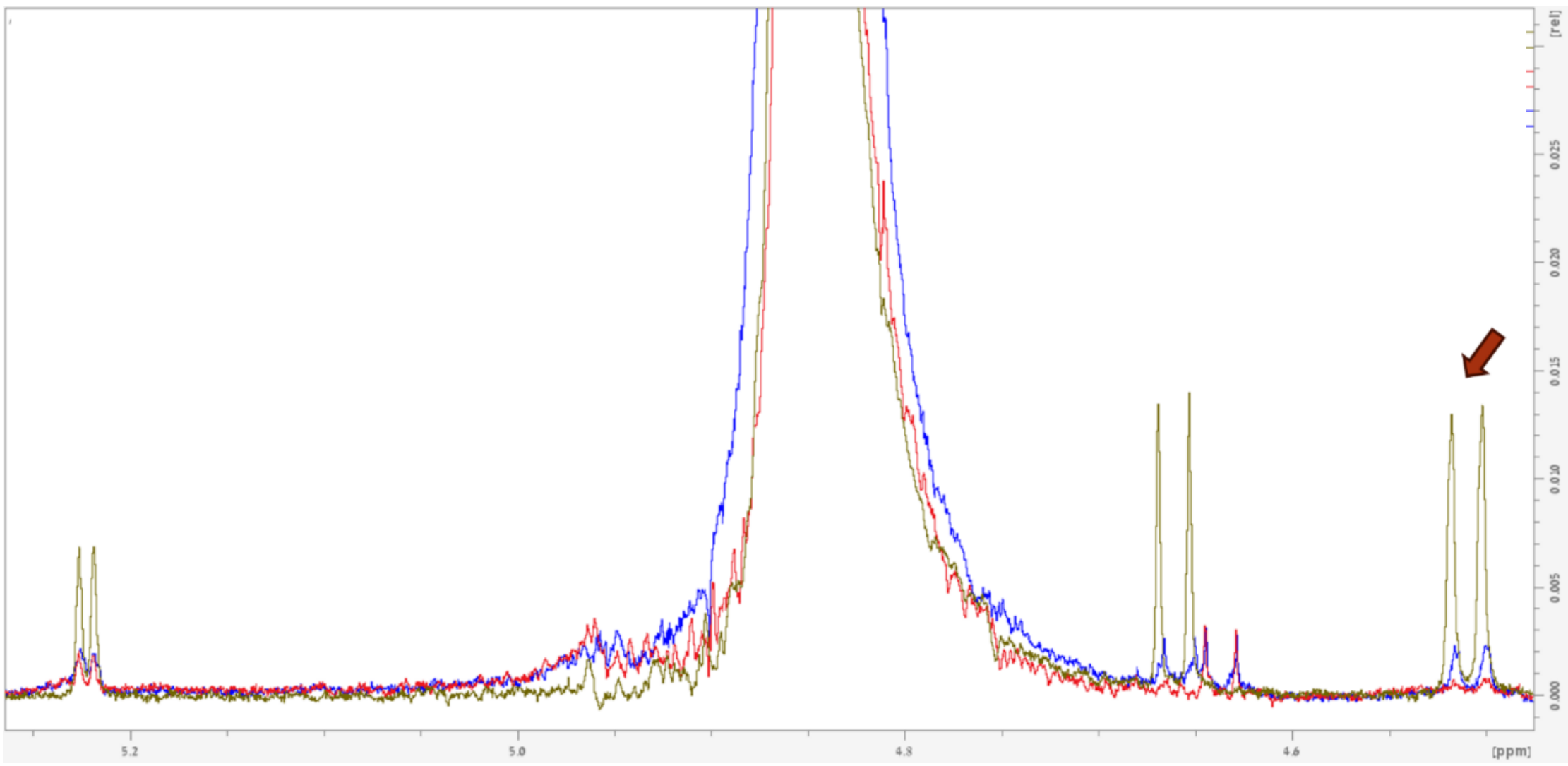

Overlay of ^{1}H-NMR spectra of a cellobiose standard (green), a dry-down reaction of glucose at 50 ˚C for seven days under acidified conditions (red), and the dry-down reaction of glucose that was spiked with

cellobiose (blue). Samples were dissolved in D_2O buffer (pH=2.66). The arrow indicates overlaps of the new peaks of the β-anomeric protons with the standard.

We added the following to Results and Discussion:

> “Spiking with standards of glucose dimers (cellobiose, isomaltose) and trimers (maltotriose, isomaltotriose) confirmed the presence of various glucose oligomers following drying reactions (Supplementary Figs. S124-S131).”
>
> “Notably, we acknowledge that integration of the anomeric protons is an underestimation of glucose conversion due to a partial overlap between the signals of the reducing end of glucose monomers and glucose oligomers.”

We added the NMR spectra of these standards and spectra overlay with reacted samples in the SI (new Supplementary Figs. S124-S131). We also added the following to Materials and Methods:

> “For experiments involving standards of glucose oligomers (cellobiose, isomaltose, maltotriose, and isomaltotriose), 0.1 M aqueous stock solutions were prepared. For analysis of standards alone, spectra were collected at a standard concentration of 5 mM in phosphate buffer with pH 2.66 in D_2O. For spiking experiments, spectra were collected in mixtures containing 50 μL of dried samples (0.1 M aqueous glucose solution, referring to the initial monomer concentration prior to drying reaction) and oligomer standard (5 μL, final concentration 0.83 mM) in phosphate buffer with pH 2.66 in D_2O.”

The purpose of the ^{1}H-NMR analysis was only to determine the amount of reacted glucose, not to perform a detailed product characterization. Due to interference for some of the anomeric signals in drying reactions, to improve accuracy we integrated only half of each anomeric monomeric signal doublet in dried samples (and multiplied by two). We used an internal standard (3-(Trimethylsilyl)-1-propanesulfonic acid-d6 sodium salt) for calibration of integration and chemical shifts. Moreover, since at 25 °C the HOD peak strongly overlaps with the β-anomeric signal, we employed measurements at 20 °C for all samples, in which the HOD resonance shifts downfield, thus improving the accuracy of our quantification method. We used the following equation for the calculation of conversion:

$$\%\,conversion = \left(1 - \frac{I_{dd}}{n * I_f}\right) * 100\%$$

Where: $I_f = \frac{I_\alpha + I_\beta}{I_{standard}}$; $I_{dd} = \frac{I_\alpha + I_\beta}{I_{standard}}$

I_f - integrated sum of anomeric peaks for fresh samples

I_{dd} - integrated sum of anomeric monomeric peaks for dried-down samples

n – dilution factor between fresh and dried samples, where $n = \frac{C_{dd}}{C_f}$ (C- concentration; for samples in D_2O n=5; for samples in DMSO n=1.5)

As an example, we show here a calculated conversion for a dry-down reaction of glucose in the absence of metal ions under acidified conditions. The top spectrum shows ^{1}H-NMR of the fresh samples, and the bottom shows the spectrum of the dried samples. Relevant integrations for the calculation of conversion are shown in red.

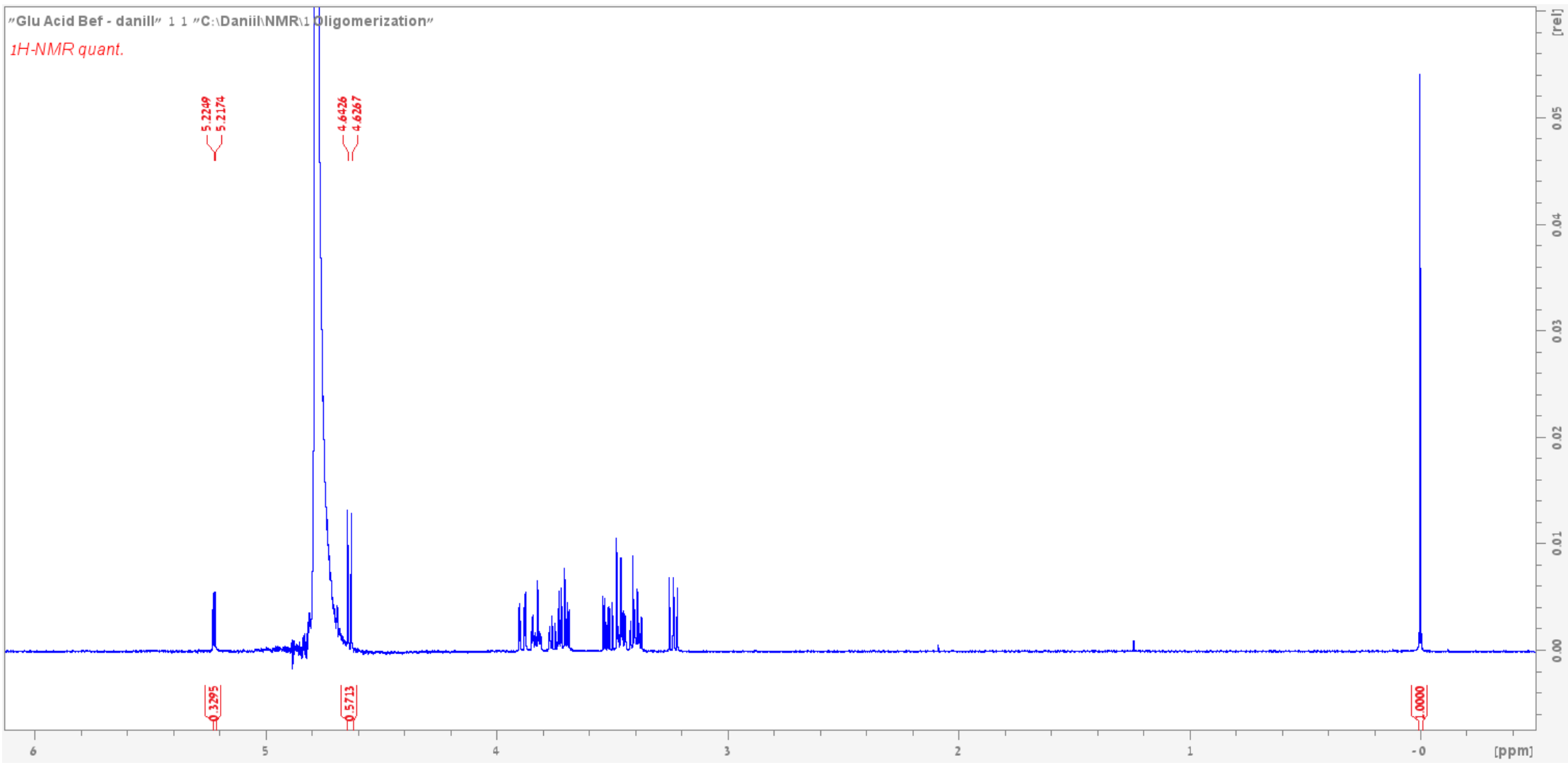

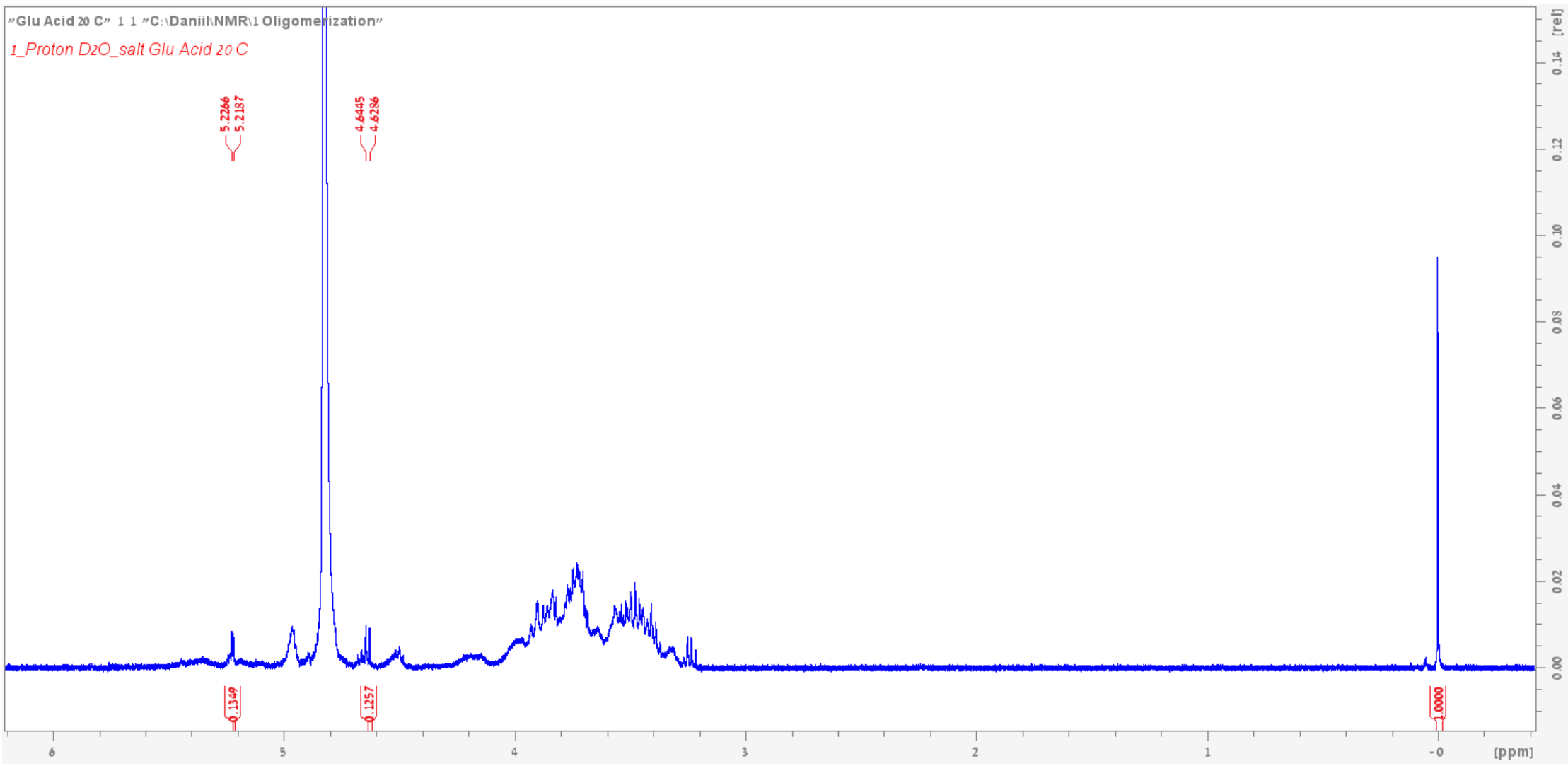

$$I_f = \frac{0.3295+0.5713}{1.0000} = 0.9008$$

$$I_{dd} = \frac{2*0.1349+2*0.1257}{1.0000} = 0.5212$$

$$\%\ conversion = \left(1 - \frac{0.5212}{5 * 0.9008}\right) * 100\% = 88.4\%$$

As requested, we added the full spectra and relevant integrals of all samples as a new Supplementary file (NMR spectra integrations).

We added this formula and the following explanation to Materials and Methods:

> "Due to interference for some of the anomeric signals in drying reactions, to improve accuracy we integrated only half of each anomeric monomeric signal doublet in dried samples (and multiplied by two). We used the internal standard for calibration of integration and chemical shifts. Moreover, since at 25 °C the HOD peak strongly overlaps with the β-anomeric signal, we employed measurements at 20 °C for all samples, in which the HOD resonance shifts downfield, thus improving the accuracy of our quantification method. The full spectra and relevant integrals of all samples are shown in the Supplementary file 'NMR spectra integrations'.
>
> The conversion was calculated as follows:

$$\% \, conversion = \left(1 - \frac{I_{dd}}{n * I_f}\right) * 100\%$$

Where: $I_f = \frac{I_\alpha + I_\beta}{I_{standard}}$; $I_{dd} = \frac{I_\alpha + I_\beta}{I_{standard}}$

I_f - integrated sum of anomeric peaks for fresh samples

I_{dd} - integrated sum of anomeric monomeric peaks for dried-down samples

n – dilution factor between fresh and dried samples, where $n = \frac{C_{dd}}{C_f}$ (C-concentration; for samples in D_2O n=5; for samples in DMSO n=1.5)"

Reviewer 1 comment 4. An additional problem is that the solvent signal appears in the same region, making impossible a precise quantification. This makes the results of Figure 4 quite uncertain.

Response to Reviewer 1 comment 4. Since at 25 °C the HOD peak strongly overlaps with the β-anomeric signal, we employed measurements at 20 °C for all samples, in which the HOD resonance shifts downfield, thus improving the accuracy of our quantification method. Moreover, due to interference for some of the anomeric signals in drying reactions, to improve accuracy we integrated only half of each anomeric monomeric signal doublet in dried samples (and multiplied by two). This is now clarified in Materials and Methods. Please also see the detailed response to Reviewer 1 Comment 3. Taken together, we believe these measures provide robust and reliable quantification for the NMR data.

Reviewer 1 comment 5. Or is it that the conversion was just calculated with the remaining monomer concentration? In any case, it is not evident whether the majority of products that are being formed are oligomers or something else. Please take into account that bare metal ions tend to degrade biomolecules quite easily, when they are not protected in the coordination sphere of a coordination complex or a metalloenzyme.

Response to Reviewer 1 comment 5. Exactly - the conversion was calculated with the remaining monomer concentration. We now understand that the methodology for calculating conversion was not adequately described, and we have now clarified this as outlined in response to Reviewer 1 Comment 3. As also mentioned in response to that comment, we know that at least the majority of these new products under acidified conditions are oligomers, based on our MS analysis, published NMR references (e.g., Li *et al*, originally ref 37), as well as new NMR spectra of oligomers that we collected. Also, as mentioned in response to Reviewer 1 Comment 3, we acknowledge that not all products correspond to oligomers, as elimination or isomerization side products may form (especially under basic conditions).

Regarding the comment about metal ion-induced degradation of sugars, while we cannot exclude this effect entirely, we do see very similar ^{1}H-NMR spectra of glucose in the presence or absence of metal ions. This observation implies similar product formation and minimal metal ion-induced degradation upon drying and heating, as indicated below for example in a dry-down reaction of glucose in the absence (blue) or presence (red) of LiCl under acidified conditions:

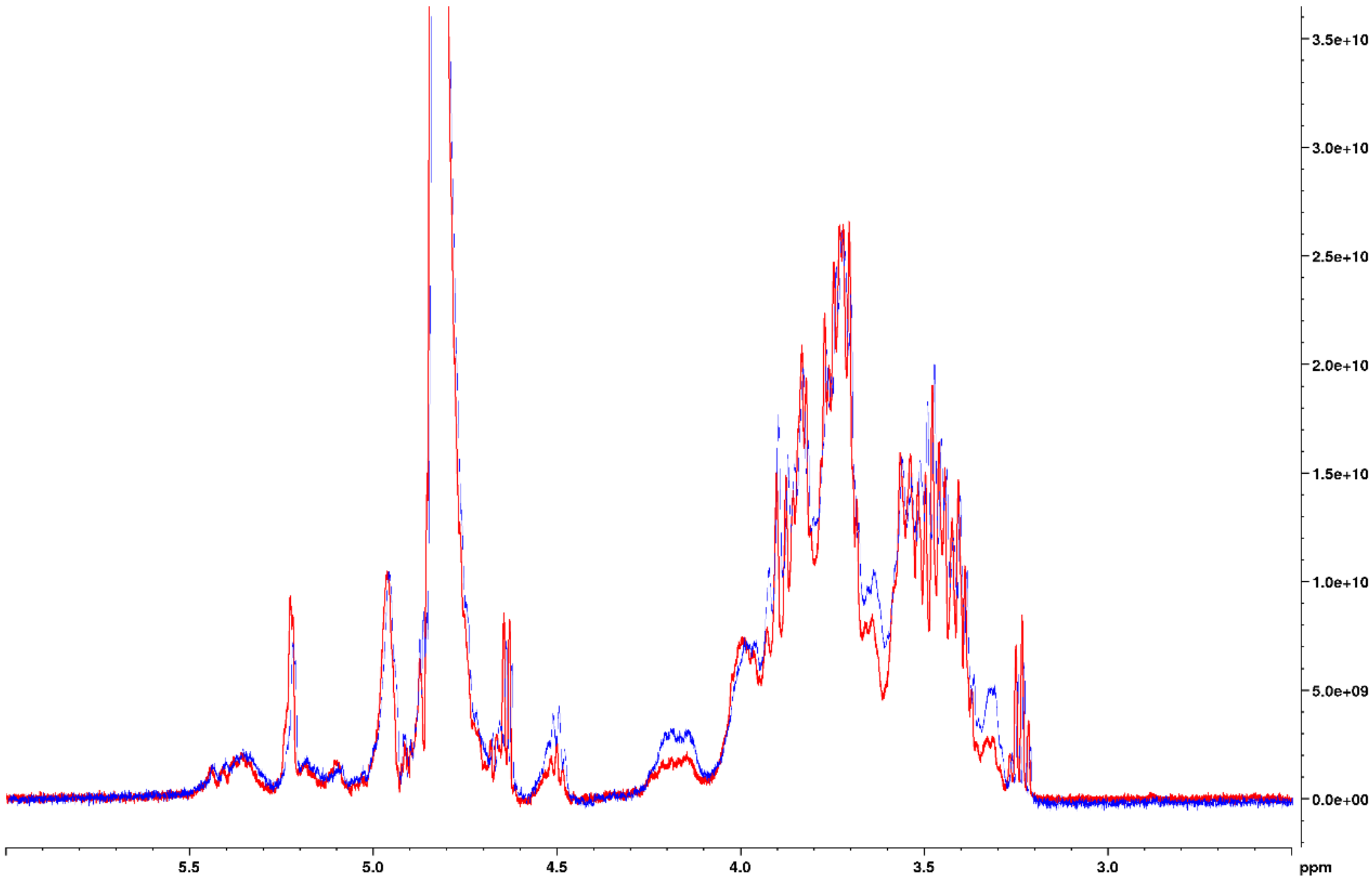

Reviewer 1 comment 6. 3) Page 5, last paragraph: This paragraph is highly speculative. The interaction of each specific metal ion with the monomer could be different. I am not sure a general conclusion for all metals like this one can be really drawn. The electronic structure of the metal can also affect a lot (not only hindering acetal formation due to coordination to the anomeric O), and even Lewis acid-catalysis might be possible in some cases. The authors go too far here, specially taking into account the limitations of their quantification method.

Response to Reviewer 1 comment 6. We agree with the reviewer that each metal ion can interact with the sugar in a different manner. To address this point, we revised the paragraph in the Results and Discussion to emphasize that the observed effects are metal-specific and that multiple contributing factors may be involved. We added the following text:

> "We observed metal-specific effects, and it is likely that different metal-related effects are dominating for different metal ions (e.g., binding/coordination, Lewis acidity, pH, hardness, and electronic-structure effects) that could either inhibit or promote oligomerization. Our results do not support a strong correlation between any single parameter and the observed effects on oligomerization, and instead point to a more complex combined effect of multiple factors. Notably, the interaction of each specific metal ion with glucose monomer or oligomers could be different."

> "Although these systems are not directly analogous to sugar oligomerization, they support the broader idea that metal ions can alter condensation equilibria in ways that may disfavor oligomer formation."

We also revised the discussion to reduce speculative language and to better separate observation from possible interpretation. In addition, we expanded and reorganized this part of the Results and Discussion under a new subtitle, 'Possible explanations for the observed effect of metal ions on glucose oligomerization reactions'.

We also note that, in response to the reviewer's concern regarding the limitations of the quantification method, we have now clarified the methodology in much greater detail and expanded the SIM-based analysis in the revised manuscript (see responses

to Reviewer 1 Comments 3, 4, 5, 8, and 9). These additions strengthen the basis for our claims. However, we have revised the discussion to ensure that the mechanistic interpretation remains cautious.

Reviewer 1 comment 7. 4) Page 5, last paragraph: "The results of 13C-NMR and HSQC supported our former 1H-NMR results: acidified samples contained more oligomeric peaks compared to samples to which NaOH was added." How do the authors know these are oligomer peaks? Have they compared with the spectra of some glucose oligomer standard?

Response to Reviewer 1 comment 7. The basis for this interpretation in the original manuscript relied on Li *et al.* (2019), where all chemical shifts are provided based on oligosaccharide standard analysis. Hence, we assumed that at least the majority of these new products under acidified conditions in the wide range between 4.5 ppm and 5.5 ppm correspond to the products of glycosidic bond formation, based on our MS analysis, published NMR references (e.g., Li *et al*, originally ref 37), as well as new NMR spectra of oligomer standards that we collected. Our interpretation was that if more peaks are observed in this region, a higher number of oligomers has likely been formed in the reactions. As mentioned in response to Reviewer 1 Comment 3, in this revision we ran ^{1}H-NMR of standards of various glucose dimers (cellobiose, isomaltose) and trimers (maltotriose, isomaltotriose) as well as spiking experiments in which we added these standards to dried-down reactions of glucose. We clearly see some overlap between the new peaks that were observed in our experiments upon drying with these standards. Please also see detailed response to Reviewer 1 Comment 3.

To be more precise, we have replaced the term "oligomeric" with "product" peaks. Now it reads as follows:

> "The results of ^{13}C-NMR and HSQC supported our former ^{1}H-NMR results: acidified samples contained more product peaks compared to samples to which NaOH was added."

Reviewer 1 comment 8. 5) LC-MS studies: I don´t really understand the data interpretation in this section? How is it that pentamers, hexamers, heptamers, octamers and nonamers elute all at the same retention time in the chromatograms of Fig. S117-S125? If so, the chromatographic method (or the column) is not adequately chosen. Then trimers and tetramers give multiple peaks over a RT range of ca. 5 or 6 min. Why is it so?

Response to Reviewer 1 comment 8. This is a good point. We agree that the relatively similar retention times observed for pentamers, hexamers, heptamers, octamers, and nonamers appear unusual at first glance. First of all, we understand that the previous stacked presentation of chromatograms was not visually clear, and now added the chromatograms for each oligomer separately for better clarity (Supplementary Figs. S141-164). Our SIM analysis generally showed that longer oligomers (heptamers up to nonamers) elute together after about 9 min, supposedly unlike shorter oligomers (trimers up to hexamers) that elute as a cluster. Our chromatography method (100% water from 0–4 min, followed by a slight gradient from 78.6% to 77% water from 4–14 min) was intentionally optimized to provide improved resolution for shorter oligomers, whereas the longer oligomers were less well resolved and eluted within a narrower retention-time window. However, this method was selected because it provides improved sensitivity of longer oligomers in SIM (single ion monitoring) mode. Because the higher oligomers were present at relatively low abundance, maximizing signal intensity was more important in this case than achieving

full chromatographic separation of each individual longer oligomer (which is practically impossible due to combinatorics, as discussed below). We therefore consider this chromatographic method to be the most suitable compromise for the analysis of this complex oligomer mixture.

Our goal was not to perform a comprehensive analysis of each oligomer peak, as this is practically impossible due to the huge combinatorial space, regardless of the chromatographic method. The multiple peaks observed for trimers and tetramers likely reflect the presence of multiple isomeric products that differ in linkage position and/or anomeric configuration, which can be partially resolved under these chromatographic conditions. Notably, the combinatorial variety of glucose oligomerization products is very large. Assuming the cyclic form solely, the number of products would be 8^{n-1} (where 'n' is the number of glucose units). For instance, the number of different trimers assuming the cyclic structure would be 64 [2 linkages of either α/β (1→2, 1→3, 1→4, 1→6)]. Since these are different products with different connectivity, their hydrophobicity is also expected to vary. Therefore, it is expected that the different oligomers of the same length would have different retention times depending on their exact chemical structure. Moreover, oligosaccharides with identical connectivity may have more than a single retention time due to α/β equilibrium at the reducing end (Dua, V. K., & Bush, C. A. (1983) *Analytical biochemistry*, *133*(1), 1-8). To verify how the retention time of different oligosaccharide products changes with respect to oligomer length and connectivity on our C18 column, we conducted LC-MS analysis of different glucose dimer (cellobiose, isomaltose) and trimer (maltotriose, isomaltotriose) standards and obtained the following chromatograms based on the targeted m/z values:

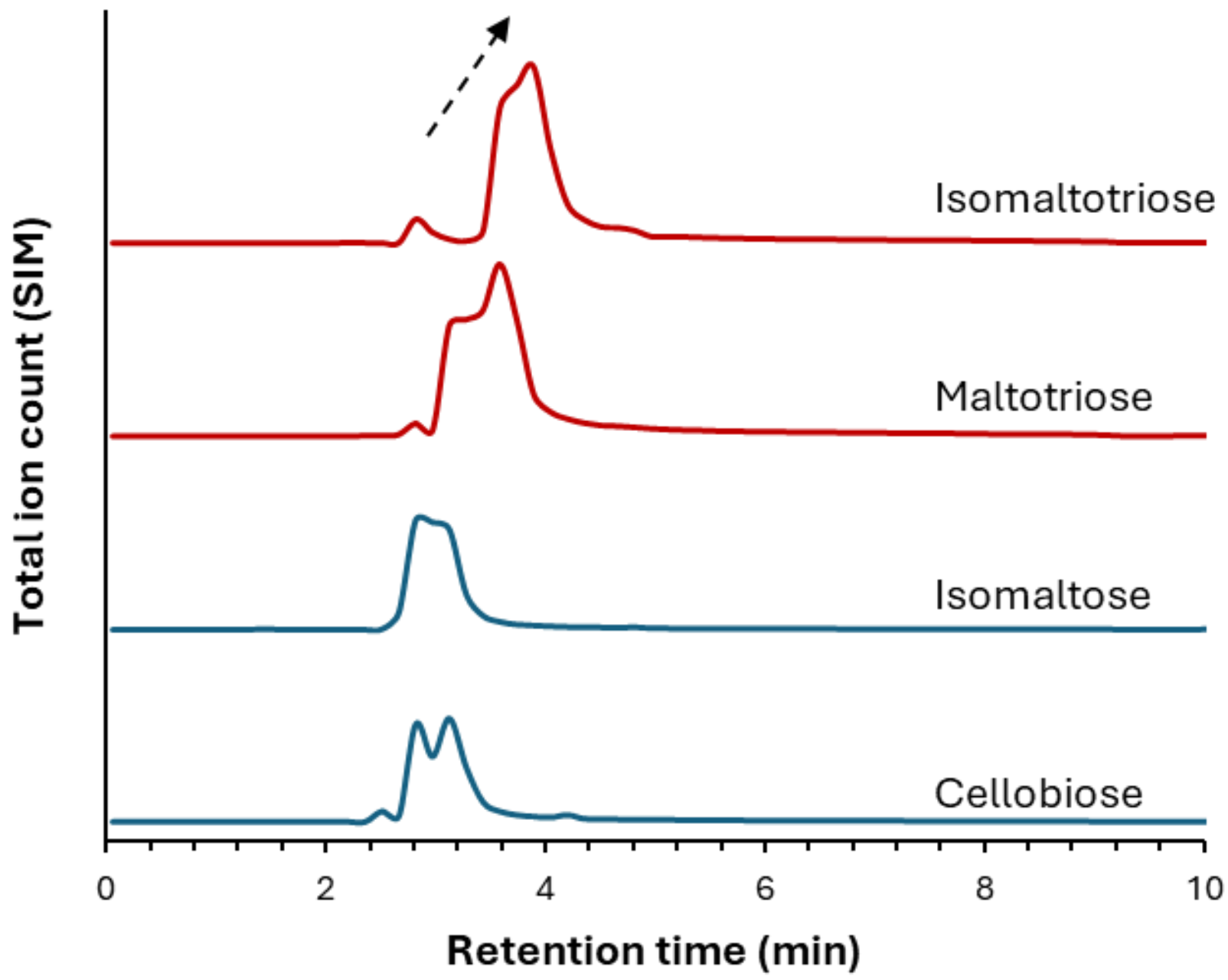

The two dimers, cellobiose (β(1→4) connectivity) and isomaltose (α(1→6) connectivity) are presented by the blue lines and the two trimers, maltotriose (α(1→4) connectivity) and isomaltotriose (α(1→6) connectivity), are presented by the red lines. While for the two tested glucose dimers, no separation (i.e., different retention times) was obtained despite the different connectivity, for glucose trimers we were able to detect a minor shift towards higher retention time in the case of isomaltotriose. For longer oligomers one may expect to have better separation between different isomers

of the same molecular weight. Moreover, in general, the longer the oligomer (assuming only linear combinations), the wider the expected retention time distributions. For oligosaccharide analytes, retention on a reverse-phase column depends primarily on the chain length and mostly on the orientation of the hydrogen atoms that form the hydrophobic region. For example, in both maltotriose and isomaltotriose, there are 8 hydrogen atoms and 3 methylene groups orienting towards the same direction (see figure below). However, in the case of maltotriose, the presence of free C6 hydroxyl lowers the hydrophobicity of the methylene regions. In isomaltotriose, on the other hand, the C6 hydroxyls form the glycosidic bond and exhibit greater hydrophobicity in that region, which indeed results in stronger interactions with the reverse stationary phase and, in accordance, slightly greater retention compared with maltotriose.

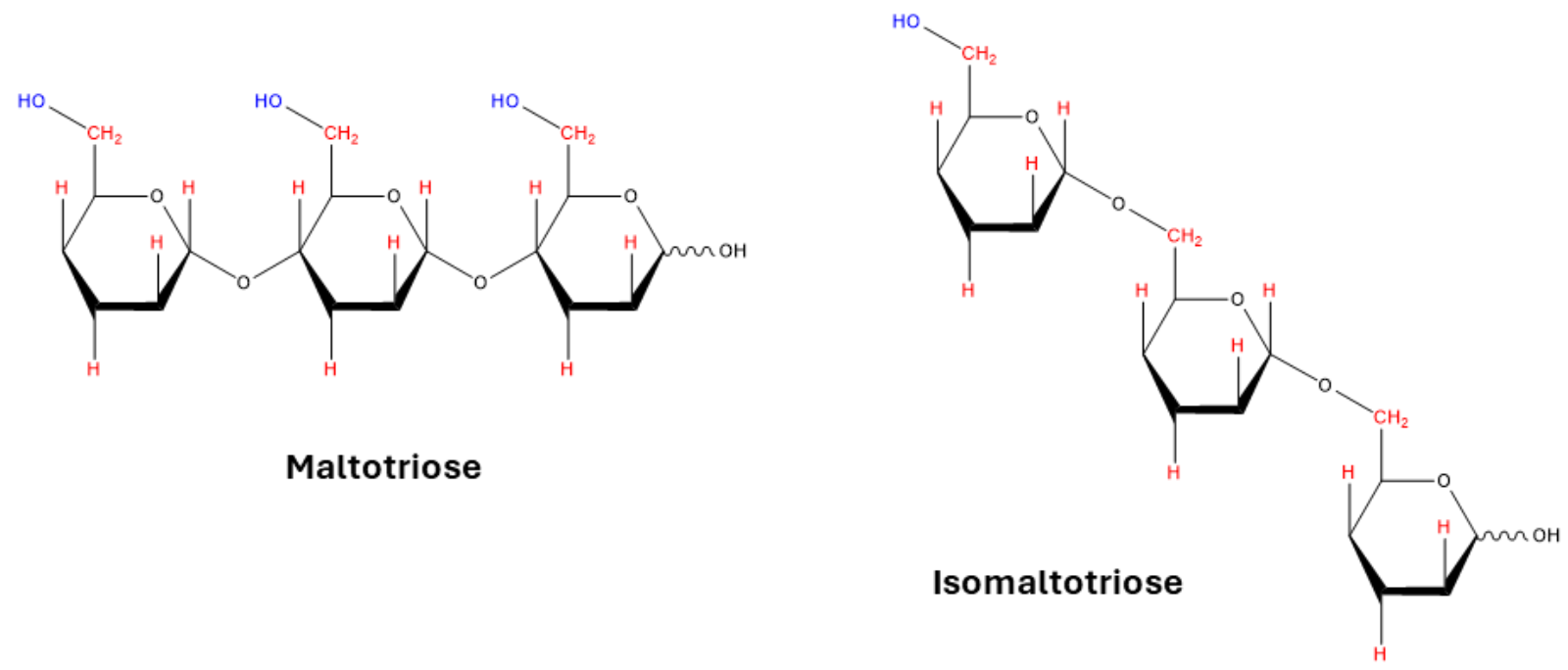

To further study oligosaccharide separation on our C18 column, we conducted LC-MS analysis of maltodextrin, which is a product of starch hydrolysis. Accordingly, maltodextrin is a mixture of glucose oligomers of various lengths, bound mostly through α(1→4) bonds but also through α(1→6) bonds. The presence of both α(1→4) and α(1→6) bonds results in “branching”, which also has an effect on the retention time. For the purpose of the analysis below, we constructed an individual chromatogram for every oligomer length in this mixture based on its corresponding m/z values:

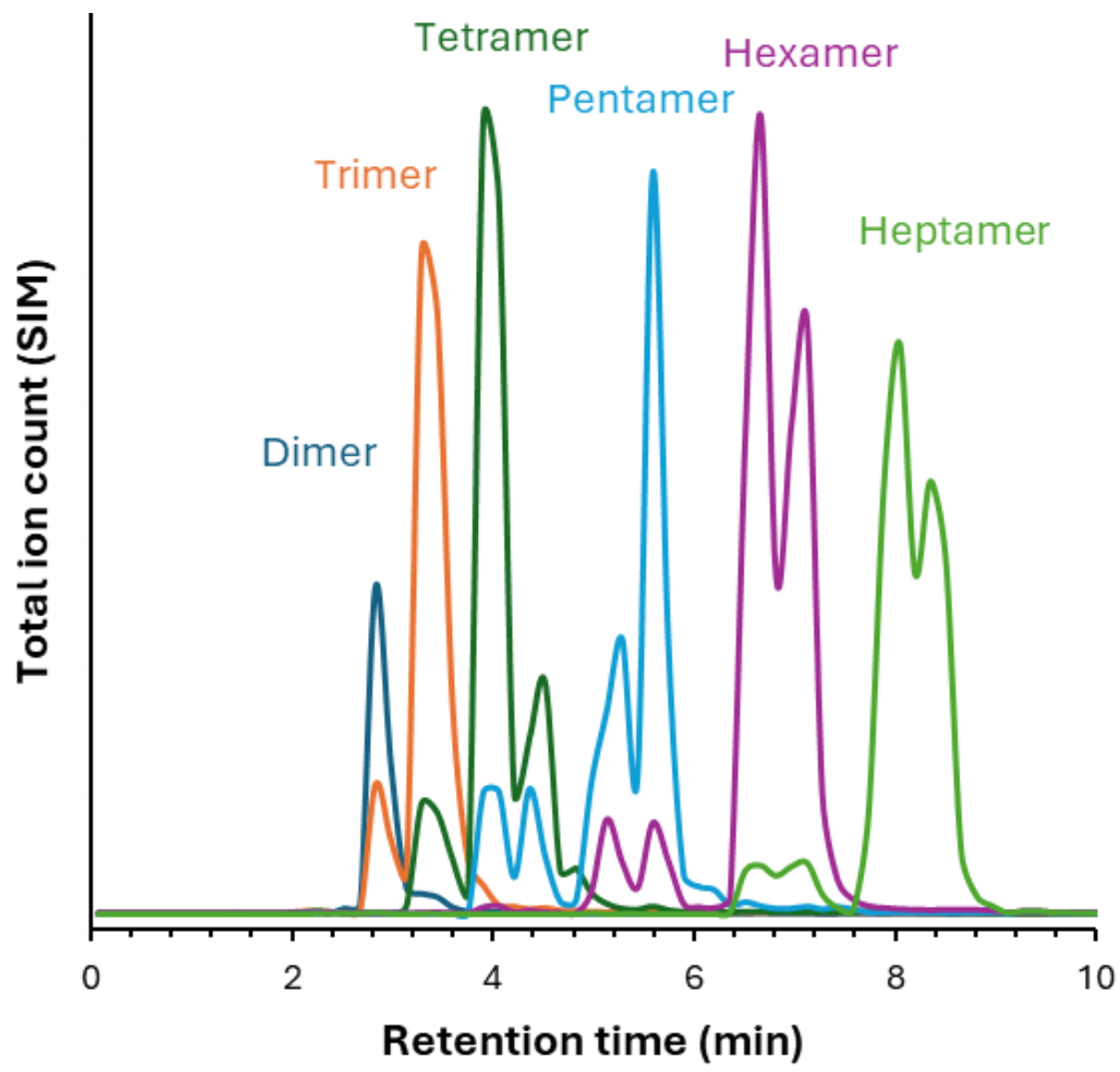

As indicated by the chromatograms above, for a given connectivity, there is a positive correlation between oligomer’s length and retention times (i.e., as the oligomer length increases - the retention time also increases). Moreover, one can see that for all oligomers except for dimers, for which no branching is possible, several peaks were observed. In addition, it is evident that oligomers of different lengths can elute together in our chromatographic separation.

In order to test the effect of the chromatographic method on the separation of the longest oligomers (hexamers up to nonamers), we injected the dry down products of glucose control (in the absence of metal ions) and analyzed it with isocratic methods at three different compositions of aqueous phase (0.1% formic acid): 80% (A), 98%(B) and 100% (C). In this analysis, we targeted the masses of hexamers to nonamers only. The obtained chromatograms are shown below (y axis is of the same scale for all chromatograms):

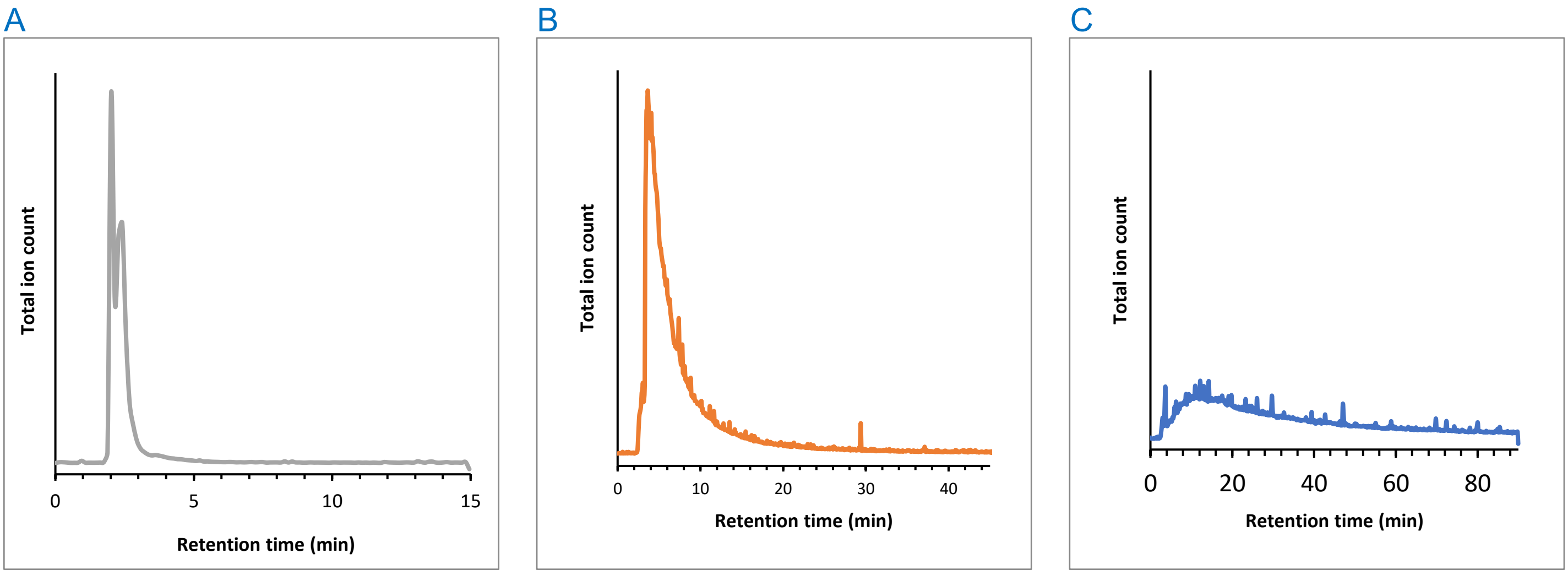

As indicated by the figures, when the content of the aqueous phase increased - the eluted peaks became broader. At 100% aqueous phase we were not able to reach a chromatographic recovery even after 90 mins, suggesting that analytes were still eluting out. Nonetheless, under these conditions we could not identify discrete peaks for all tested oligomers, and for octamers and nonamers, the bordering was at the expense of s/n. These results indicate that the elution of the longer oligomers can be prolonged, yet this is not sufficient in order to achieve better separation with the column in use (C18 column). To summarize, an isocratic 100% water method provides improved separation of some product peaks, but at the expense of sensitivity. While it is possible to use normal phase chromatography (i.e., polar stationary phase), change the chromatographic method, or perform sample pre-treatment, our goal was not to perform a comprehensive analysis of each oligomer peak, as this is practically impossible due to the huge combinatorial space, regardless of the chromatographic method.

To conclude this part, these results indicate that the chromatographic behavior observed does not reflect an artifact or misassignment, but rather the limited separation of higher oligomers under a method optimized for sensitive SIM detection, together with the observed presence of multiple isomeric species among the shorter oligomers. A comprehensive analysis of each oligomer peak is beyond the scope of this work (and practically beyond the capability of any chromatographic method). We have now clarified this in the revised manuscript as follows:

> "Since the chromatographic separation was achieved on a C18-column, retention time was expected to increase with oligomer length. However, due to the presence of multiple products, oligomers of a certain chain length typically eluted as a cluster. This was observed predominantly for trimers, tetramers, pentamers, and hexamers that exhibited continuous elution (Supplementary Figs. S141-S164). Due to the choice of chromatographic separation, the longer

oligomers eluted as a single peak or as a front-cluster (depending on the concentration) (Supplementary Figs. S141-S164)."

Moreover, for this revision, we repeated the SIM analysis for all samples at two analyte concentrations (10 mM, as in the original submission, and 1 mM) and expanded the analysis to include all observed adduct species rather than only the major adducts. This more comprehensive treatment led us to refine the interpretation of these results in several cases. We modified the paragraph describing SIM results as follows:

> "Effect of various metal ions on the product distribution in glucose oligomerization reactions. The dry-down reaction of glucose under acidified conditions produced oligomers of various sizes, as indicated by ESI-MS measurements. We utilized targeted LC-MS to perform a qualitative analysis of glucose monomers as well as glucose oligomers (dimers up to nonamers) in the presence or absence of metal ions. This analysis is only qualitative due to the huge variety of products, their co-elution, presence of ions, methodological limitations, and lack of standards for the absolute majority of these species. Notably, the combinatorial variety of glucose oligomerization products is immense. Assuming the cyclic form only, the number of products would be 8^{n-1} (where 'n' is the number of glucose units). For instance, the number of different trimers assuming the cyclic structure would be 64 [two linkages, each of which can be either α or β (1→2, 1→3, 1→4, 1→6)]."

We also modified Figure 5 as follows:

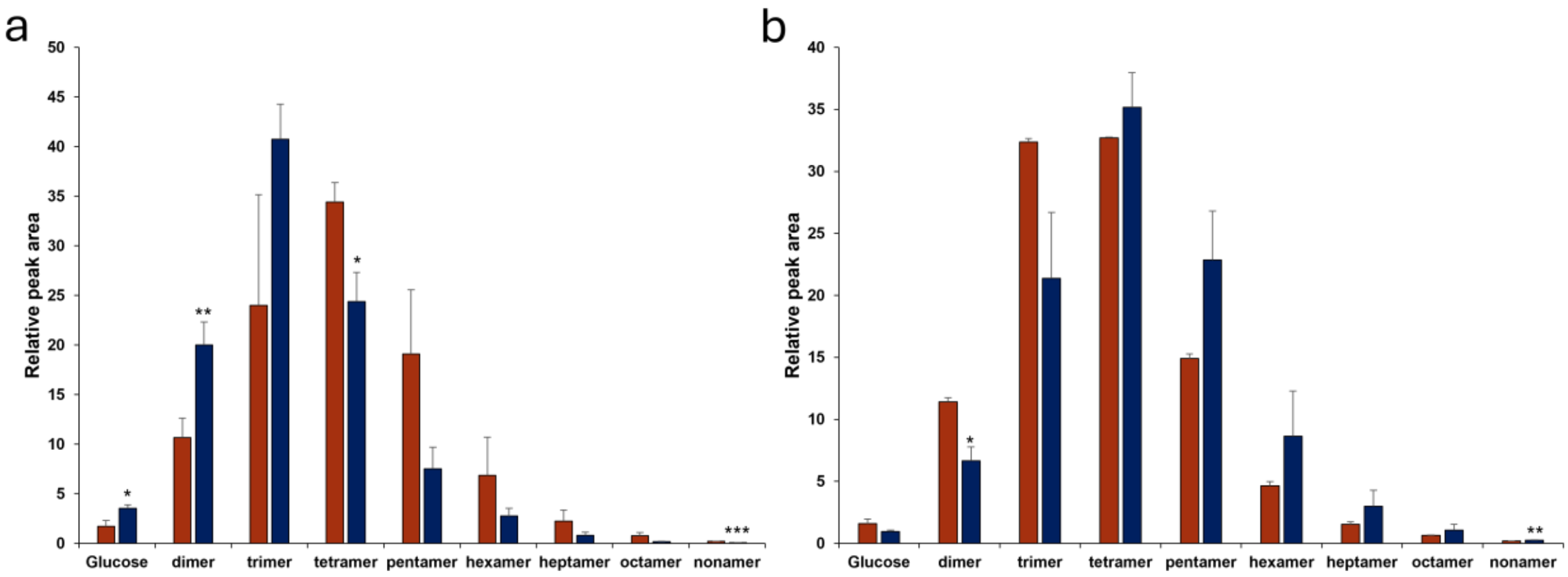

Fig. 5: Evaluation of the degree of D-Glucose oligomerization based on SIM-LC-MS. Glucose product distribution after one week of dry-down at 50 °C in the presence (blue) or absence (red) of Al^{3+} (**a**) or Fe^{2+} (**b**) under acidified conditions. Values are presented as the mean values of three independent replicate analyses with standard deviation. P-values were calculated via a t-test (* indicates $p < 0.05$, ** $p < 0.01$, *** $p < 0.001$).

We added these results as Supplementary figures in the SI (Supplementary Figs. S165-S176). We also added the following to Materials and Methods:

> "**Liquid Chromatography-Mass Spectrometry.** LC-MS analysis was performed using an Agilent 1260 quaternary pump and an autosampler with a DAD UV-Vis detector, coupled to an Agilent G6135 single quadrupole. Samples (either 1 mM or 10 mM) were injected onto an Agilent Poroshell 120 EC-C18 column. Column was held at 20 °C. Solvent A: Water (0.1 % formic acid); solvent B: ACN. Flow rate: 0.3 mL/min. Acquisition method: (A%) 100% (0-4

min), linear-gradient (A%) 78.6% to 77% (4-14 min), followed by wash linear-gradient (B%) 23% to 100% (14-21 min), (B%) 100% (21-28 min). Injection volume: 10 µL. Capillary voltage: 4000 V. Fragmentor voltage of either 70 V (for Scan mode) and 135 V (for SIM mode).

For SIM, mass values of each analyte (from glucose monomers up to glucose nonamers) were defined. For each analyte with exact mass *M*, three ionization species were considered: $[M\text{-}H]^-$, $[M+Cl]^-$ and $[M+HCOO]^-$, as specified in Table S3. In total, 27 masses were monitored. Individual SIM chromatograms of each ionized species and a TIC-SIM chromatogram of all target masses were recorded. Analysis of oligomer formation was carried out by integrating the corresponding peaks in SIM chromatograms of each ionized species. For each species (a monomer or an oligomer), a total peak area was calculated and distribution profiles of either absolute peak area or relative peak area were constructed.

Relative peak areas (RA_i) were calculated as follows:

$$RA_i = \frac{A_{i,[M-H]^-} + A_{i,[M+Cl]^-} + A_{i,[M+HCOO]^-}}{\sum_{i=1}^{n} A_{i,[M-H]^-} + A_{i,[M+Cl]^-} + A_{i,[M+HCOO]^-}} \times 100\%$$

Where $A_{i[M\text{-}H]^-}$, $A_{i[M+Cl]^-}$ and $A_{i[M+HCOO]^-}$ are the peak areas of product *i* obtained for ionized species $[M\text{-}H]^-$, $[M+Cl]^-$ and $[M+HCOO]^-$, respectively. *i* is an integer between 1 and 9 that indicates the specific product (monomer, dimer, trimer etc.).

Cumulative profiles were constructed as well, considering the total contribution of product *k* in a cumulative manner:

$$CA_k = \sum_{i=1}^{k} RA_i$$

$$0 < \sum_{i=1}^{k} RA_i \leq 100\%$$

Where CA_k is the cumulative area of species *k,* which is the sum of RA_i from *i*=1 until *i*=*k*. For example, CA_3 is cumulative area of trimers, which is the sum of relative peak area obtained for monomers (*i*=1), dimers (*i*=2), and trimers (*i*=3) species. "

Reviewer 1 comment 9. Under these circumstances, the variations in peak areas between samples, which are not dramatic (as the authors claim at the end of the introduction) but quite minor (with the exception of Fe2+), are not really significant. Indeed, with the current data I cannot be sure of which species they are really looking at through LC-MS and NMR. Further justification would be required.

Response to Reviewer 1 comment 9. We appreciate the reviewer's concern and have substantially revised and clarified our analyses, as discussed above. For example, as mentioned in response to Reviewer 1 Comment 3, NMR was used only to quantify glucose conversion and was not used for the assignment of individual oligomeric products. Product distributions were evaluated qualitatively by LC-MS SIM, which allowed us to monitor ions corresponding to oligomers of different chain length (e.g., dimers, trimers, tetramers, etc.). This approach does not distinguish between

isomeric products of identical chain length that differ in connectivity, but it does enable comparison of oligomer-length distributions across samples.

Moreover, as noted in response to Reviewer 1 Comment 8, for this revision, we repeated the SIM analysis for all samples at two analyte concentrations (10 mM, as in the original submission, and 1 mM) and expanded the analysis to include all observed adduct species rather than only the major adducts. This more comprehensive reanalysis led us to refine the interpretation in several cases and to revise some of our conclusions accordingly. Regarding the SIM analysis, we now state as follows:

> "When examining the peak areas of the longest oligomers (heptamers to nonamers), we did not observe significant differences between the samples, except for Ca^{2+} and Al^{3+}, for which these oligomers were formed to a lesser extent compared with the control sample in the absence of metals. Al^{3+} produced the strongest shift in product distribution toward short oligomers (Fig. 5a). In its presence, ~80% of the total peak area corresponded to trimers or shorter oligomers, compared with ~50% in the glucose control and other metal-containing samples. Likewise, ~95% of total peak area in the Al^{3+} sample was assigned to tetramers and shorter oligomers, whereas in most other samples the same cumulative proportion extended to hexamers, and to pentamers in the presence of Ca^{2+} (Supplementary Figs. S165-S176). Our analysis suggested that there might be a slight shift towards longer oligomers in the case of Fe^{2+}. In its presence, ~36% of the total peak area corresponded to pentamers or longer oligomers, compared with ~22% in the glucose control (Fig. 5b and Supplementary Figs. S175-176)."

Taken together, we hope that the added controls, standards, repeated SIM analyses, expanded adduct analysis, and statistical treatment now clarify both what species are being monitored and which differences between conditions are supported by the data. Please also see our responses to Reviewer 1 Comments 3 and 8.

Reviewer #2 (Remarks to the Author):

Reviewer 2 comment 1. The paper by Akulenko et al. describes the effect of metal cations on the polymerization and isomerization of glucose. The authors use a plausible prebiotic setting, drying conditions, 50 ˚C, 1 week incubation, to trigger the oligomerization chemistry. The product mixtures are analyzed by a multitude of experimental techniques, including FTIR, NMR, ESI-MS, LC-MS for acidic as well as alkaline conditions and for a series of metal cations, including alkali, alkaline earth as well as first row transition metals. A comprehensive characterization of both the oligomerization products and cation-glucose binding is given, which is documented in a perfectly elaborated 127 pages long supplementary material.

Response to Reviewer 2 comment 1. We thank the reviewer for this positive assessment and for recognizing the scope of the experimental design and the analytical characterization across multiple cations and conditions. We thank the reviewer for their comments and believe that these comments have strengthened our manuscript.

Reviewer 2 comment 2. I appreciated the effort of the authors to enter the so-far untouched area of prebiotic carbohydrate oligomerizations and especially that they directed their attention to the perhaps hardest problem in this topic, i.e. the effect of metal cations on this chemistry. Indeed, the study clearly demonstrates that metal cations may change the outcome of carbohydrate polymerization reactions

dramatically, in addition, in a cation-specific manner. Considering the depth and highly professional realization of the work, I think this study will represent a seminal contribution to the prebiotic chemistry field and will form the foundation of future studies related to the origin of polysaccharides that is an important class of terrestrial biomolecules. For this reason, I strongly recommend publication of this work in Communications Chemistry even in its present form. Optionally, the authors might want to consider the following suggestions:

Response to Reviewer 2 comment 2. We thank the reviewer for this supportive assessment and for highlighting the significance of our study. We appreciate the recognition of the novelty of investigating prebiotic sugar oligomerization and, in particular, the challenging and underexplored role of metal cations in shaping these reactions. We have carefully considered the reviewer's suggestions and addressed them point-by-point below.

Reviewer 2 comment 3. The authors give a very logical explanation for the inhibitory effect of Mn2+ and Fe2+ on page 5, last paragraph, attributing it to the binding of the anomeric OH to the metal cations. I think it would help the readers if the authors could illustrate this effect with a schematic drawing in the supplementary material.

Response to Reviewer 2 comment 3. Great idea. We added a schematic drawing as suggested (new Supplementary Fig. S177):

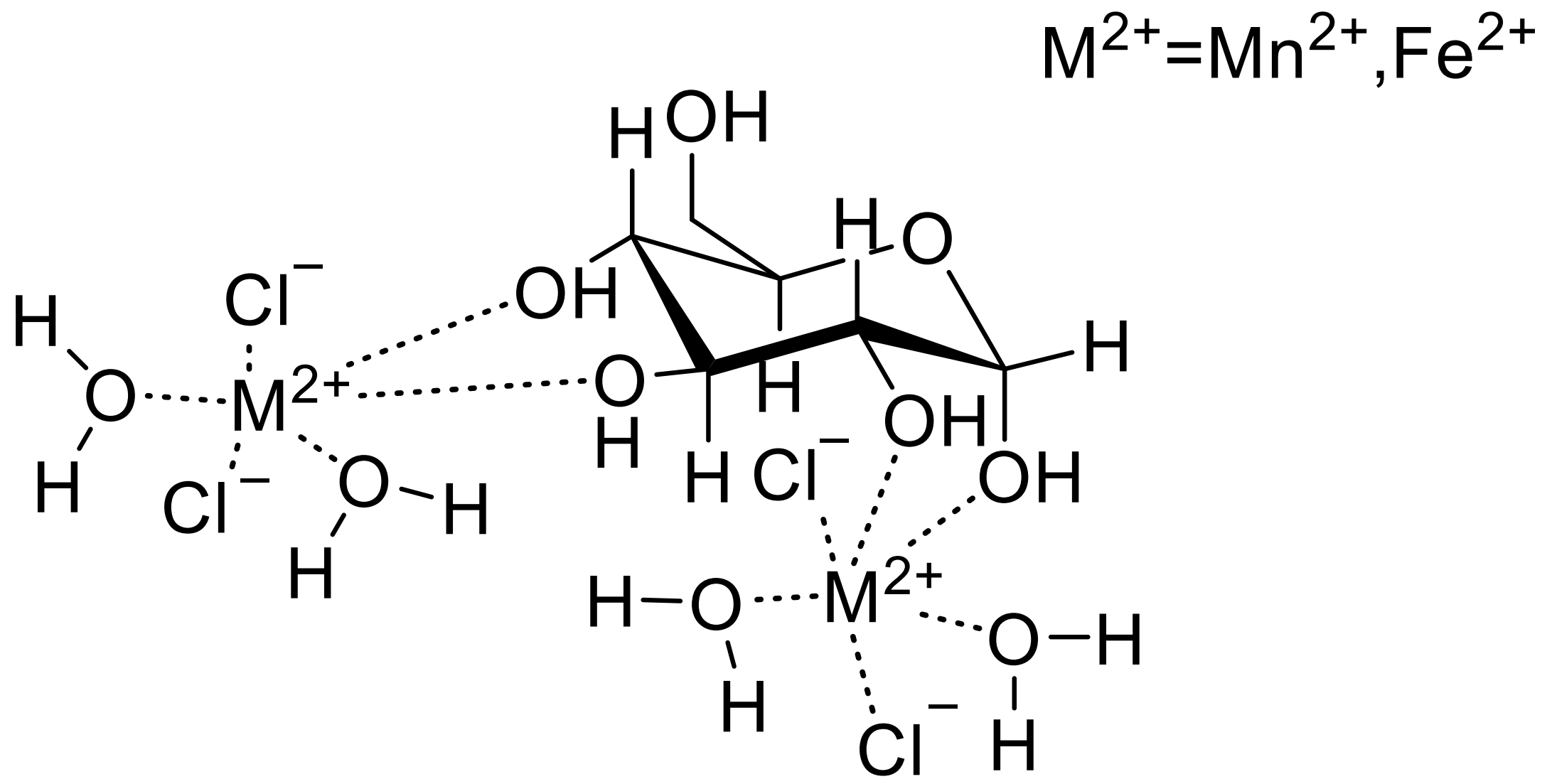

Possible complexation of glucose with transition metal ions

Reviewer 2 comment 4. Though the list of metal cations considered is very comprehensive from a prebiotic chemistry point of view, to my taste a very specific metal cation, Al3+, is missing from the list. Al3+ is actually the most abundant metal cation in the Earth crust, and is a common component of silicate rocks. In addition, Al3+ is a group 3A element, the neighbor of boron, an element that is believed to play an important role in the stabilization of sugars in the prebiotic mix. It would be great if the authors could devote a couple of words to Al3+ in their discussion.

Response to Reviewer 2 comment 4. We appreciate the comment. While today Al^{3+} is mostly bound in minerals such as silicates, oxides, and hydroxides, it could have possibly played a key role due to its interesting properties and known catalytic role in various reactions (such as the Friedel–Crafts). This comment prompted us to include

this metal ion as well in our study for this revision. Indeed, Al^{3+} had a strong effect. We found that overall, Al^{3+} inhibited glucose oligomerization under both tested conditions (Figure 4 and Supplementary Figs. S149, S161). Notably, the conversion according to NMR quantification analysis was similar in both tested conditions (56% for acidified conditions and 54% upon addition of NaOH, respectively), probably due to the nature of Al^{3+} as a strong Lewis acid (the pH in the two environments was ~4). MS analysis indicated that the largest oligomer observed was the pentamer. SIM analysis indicated a lower degree of oligomerization compared to control samples (Supplementary Figs. SX-SY). We added the following to Results and Discussion:

> " Indeed, the pH of the glucose sample in the presence of Al3+ was higher than that of the other samples (~4 instead of ~2), and the oligomerization extent in its presence was lower."

> "The conversion in the presence of Al^{3+} was similar under acidified conditions and upon addition of NaOH: 56% and 54%, respectively. As mentioned above, this can be explained by the similar pH under both conditions (pH ~ 4)."

> "As indicated by the distribution profiles, for most samples, the most abundant oligomers in terms of peak area were trimers (~30% of total peak area) and tetramers (~34% of total peak area). The only exception was Al^{3+}, for which the most abundant oligomers were dimers (~35% of total peak area) and trimers (~40% of total peak area). When examining the peak areas of the longest oligomers (heptamers to nonamers), we did not observe significant differences between the samples, except for Ca^{2+} and Al^{3+}, for which these oligomers were formed to a lesser extent compared with the control sample in the absence of metals. Al^{3+} produced the strongest shift in product distribution toward short oligomers (Fig. 5a). In its presence, ~80% of the total peak area corresponded to trimers or shorter oligomers, compared with ~50% in the glucose control and other metal-containing samples. Likewise, ~95% of total peak area in the Al^{3+} sample was assigned to tetramers and shorter oligomers, whereas in most other samples the same cumulative proportion extended to hexamers, and to pentamers in the presence of Ca^{2+} (Supplementary Figs. S165-S176)."

> "Notably, the most prominent effects were observed in the presence of Al^{3+}, which is the only trivalent cation that was tested in our study. As mentioned, the pH of the glucose sample in the presence of Al^{3+} was higher than that of the other samples (~4 instead of ~2), and the oligomerization extent in its presence was lower. The higher pH is expected to decrease the extent of oligomerization, as acetal bond formation is acid-catalyzed. Among the studied group of cations, Al^{3+} is the hardest. Since the hydroxyl oxygens on the glucose molecule are also classified as hard donor sites, their interaction with Al^{3+} is expected to be particularly strong. [106-109] This stability is driven by the large energy gap between the HOMO of the glucose and the LUMO of the metal ion, which prevents significant electron sharing and instead facilitates a stable, primarily electrostatic interaction characteristic of hard-hard species according to the HSAB principle. This strong, localized binding can effectively hinder the reactive sites of the sugar."

We added Supplementary figures S10, S19, S30, S40, S50, S60, S70, S79, S89, S99, S109, S119, S149, S161 and incorporated the results for Al^{3+} into Figures 4 and 5. For example, we modified Figure 5 as follows:

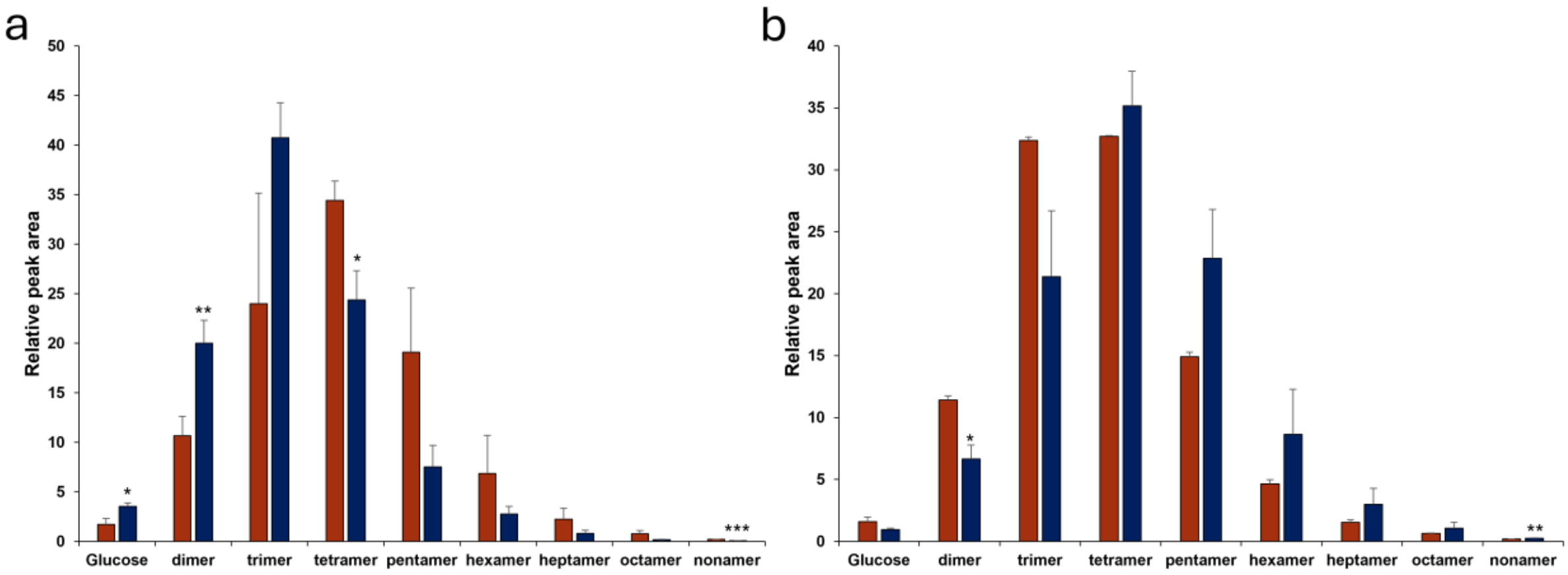

Fig. 5: Evaluation of the degree of D-Glucose oligomerization based on SIM-LC-MS. Glucose product distribution after one week of dry-down at 50 °C in the presence (blue) or absence (red) of Al^{3+} (**a**) or Fe^{2+} (**b**) under acidified conditions. Values are presented as the mean values of three independent replicate analyses with standard deviation. P-values were calculated via a t-test (* indicates $p < 0.05$, ** $p < 0.01$, *** $p < 0.001$).

Reviewer #3 (Remarks to the Author):

Reviewer 3 comment 1. In this manuscript, the authors present extensive work aimed at elucidating the potential role of several metal cations in the oligomerisation of sugars under plausible prebiotic conditions. Two series of experiments were conducted under acidic and basic pH values, respectively, and under drying conditions, using up to eight chloride salts containing metals considered abundant on the primitive Earth. All experiments were performed in triplicate, demonstrating good reproducibility. Glucose was selected as the model sugar, based on the only previous study that addressed a potential prebiotic synthesis of oligosaccharides. Several analytical techniques were employed for the characterisation of the final reaction mixtures, including ATR-FTIR, 1H and 13C NMR spectroscopy, ESI-MS, and LC-MS. A large amount of data was analysed to determine the influence of the initial pH and the presence of metals on both the conversion of glucose and its transformation into various oligosaccharides. Although this work may be relevant to the field of prebiotic chemistry, given the very limited number of studies concerning the oligomerisation of polysaccharides and their important role in contemporary biochemistry, several inconsistencies and details, must be more thoroughly addressed before the manuscript can be considered for publication in Communications Chemistry.

Response to Reviewer 3 comment 1. We thank the reviewer for recognizing the novelty of this underexplored subfield of prebiotic sugar chemistry. This paper represents our first step in this direction. We thank the reviewer for their comments and have addressed them as described herein. We believe that these comments improved the manuscript and the clarity of our findings.

Reviewer 3 comment 2. Main comments

1) The main focus of this paper is the conversion of glucose into its corresponding oligomers in the presence or absence of metal cations. It is somewhat surprising that, despite having access to a powerful analytical tool such as an LC–MS system coupled with a DAD UV–Vis detector, the authors did not take fuller advantage of this

instrumentation. There are several commercially available maltodextrin and isomaltose oligosaccharide standards that could have been used to develop and validate an accurate analytical method for the proper quantification of both glucose consumption and oligosaccharide production. The following references provide useful examples that could have guided the authors in designing a suitable analytical method for quantifying oligomers derived from glucose: Carbohydrate Research 257 (1994) 249–268; Journal of Chromatography 207 (1981) 439–444; Chromatographia 50 (1999) 705–710; Z. Lebensm. Unters. Forsch. A 206 (1998) 175–178; and http://dx.doi.org/10.2139/ssrn.5413053. Since the stereochemistry of the oligomers is not a central aspect of this study, which focuses primarily on the production and chain length of the oligosaccharides, the use of external standards would have enabled the construction of calibration curves and an accurate quantification of the oligomers formed in the presence of each metal cation. This methodology would allow a more meaningful comparison between experiments and support more robust overall conclusions.

Response to Reviewer 3 comment 2. We appreciate the reviewer's suggestions and included new analyses in this revision. However, it is important to stress that quantifying each individual oligomer peak is extremely analytically challenging and beyond the scope of this work, due to reasons discussed below.

We would like to discuss the analytical challenges one faces when analyzing complex mixtures such as the ones we obtained in model prebiotic sugar reactions. The combinatorial variety of glucose oligomerization products is immense and therefore cannot be simply analyzed by standard LC. Assuming the cyclic form solely, the number of products would be 8^{n-1} (where 'n' is the number of glucose units). For instance, the number of different trimers assuming the cyclic structure would be 64 [2 linkages of either α/β (1→2, 1→3, 1→4, 1→6)]. Please also see response to Reviewer 1 Comment 8.

Regarding the point raised about LC-MS, we agree with the reviewer that LCMS is a powerful analytical tool. Notably, DAD UV–Vis detector is not helpful as sugars and acetal bonds are not good chromophores. We thank the reviewer for bringing to our attention the analytical work done for characterizing and quantifying different oligosaccharides. We read all references carefully. Notably, in four out of five studies, the tested oligomers were maltodextrins or other starch derivatives, which are mixtures of mostly linear glucose oligomers linked through α(1→4) bonds. It is possible that minor fractions also contained α(1→6) bonds. That is, the variety of different bond types is limited, and separation is mostly based on oligomerization degree. As we also showed (see response to reviewer 1 comment 8), the separation between glucose oligomers of up to heptamers is feasible even on a traditional reverse phase column (C18). The chromatographic challenge is rather separating between oligomers of similar chain length but different connectivity.

Following the reviewers' comments, in this revision we added analysis of glucose dimer and trimer standards as well as maltodextrin samples. Moreover, we performed a more comprehensive SIM analysis (see below). We believe that these additions have strengthened our manuscript.

As for glucose standards, we ran ^{1}H-NMR of standards of various glucose dimers (cellobiose, isomaltose) and trimers (maltotriose, isomaltotriose). We also ran spiking experiments in which we added these standards to dried-down reactions of glucose in the absence and presence of Zn^{2+}. We clearly see some overlap between the new

peaks that were observed in our experiments upon drying with these standards. We added the following to Results and Discussion:

> "Spiking with standards of glucose dimers (cellobiose, isomaltose) and trimers (maltotriose, isomaltotriose) confirmed the presence of various glucose oligomers following drying reactions (Supplementary Figs. S124-S131)."

We also added the NMR spectra of these standards and spectra overlay with reacted samples in the SI (Supplementary Figs. S124-S131). Please also see response to Reviewer 1 Comment 3.

Regarding the analysis of maltodextrin samples, we conducted LC-MS analysis of maltodextrin. Maltodextrin is a mixture of glucose oligomers of various lengths, bound mostly through α(1→4) bonds but also through α(1→6) bonds. The presence of both α(1→4) and α(1→6) bonds results in branching, which also has an effect on the retention time on the C18 column. For the purpose of the analysis below, we constructed an individual chromatogram for every oligomer length in this mixture based on its corresponding m/z values:

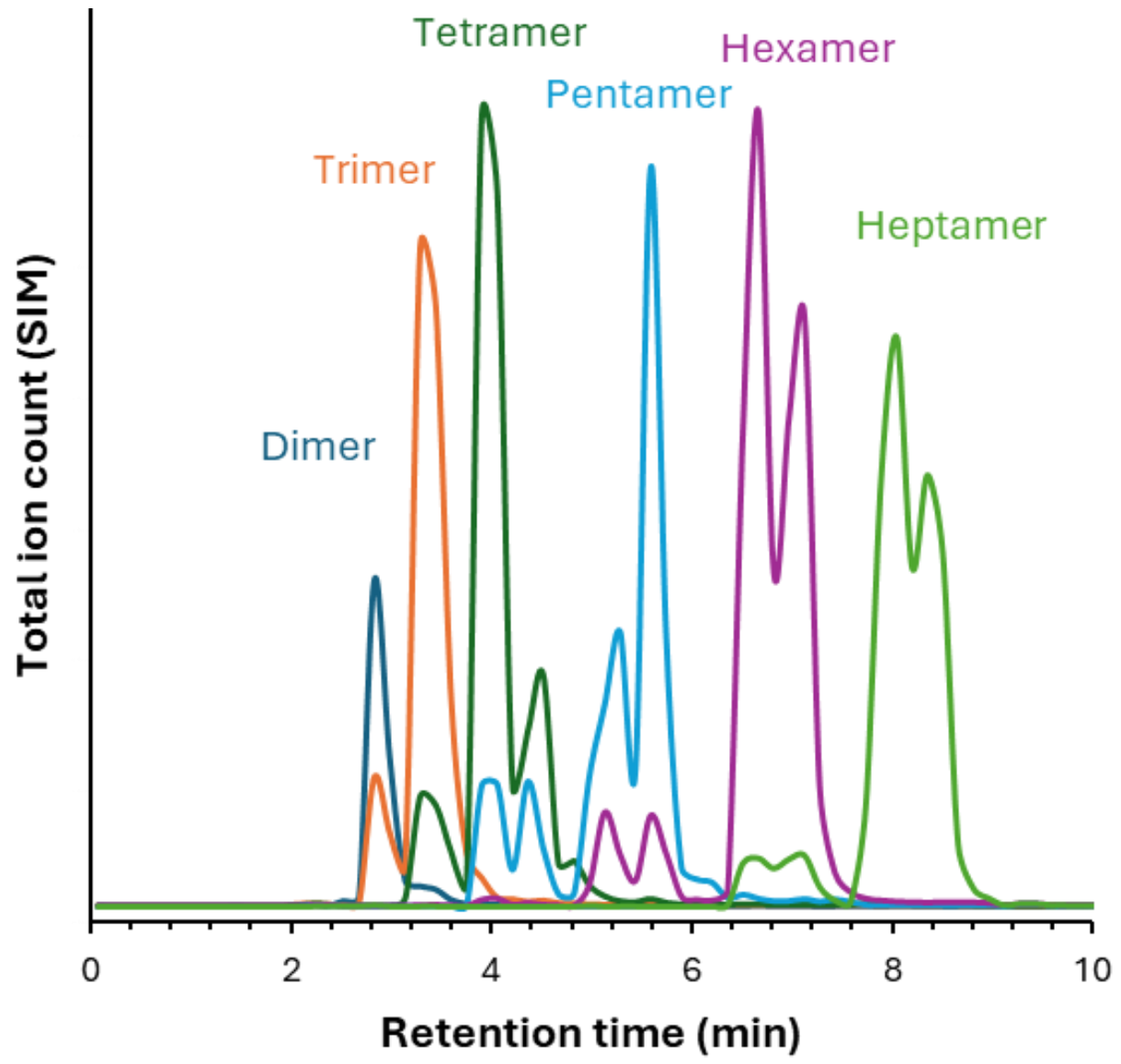

As indicated by the chromatograms above, for a given connectivity, there is a positive correlation between oligomer's length and retention times (i.e., as the oligomer length increases - the retention time also increases). Moreover, one can see that for all oligomers except for dimers, for which no branching is possible, several peaks were observed. Taken together, this control experiment indicates that on our C18 column, oligosaccharide retention is driven primarily by chain length, while many linkage/anomeric isomers are only partially resolved. A comprehensive analysis of each oligomer peak is beyond the scope of this work. Please also see response to Reviewer 1 Comment 8.

Regarding the reviewer's point about accurate quantification of the oligomers formed in the presence of each metal cation, the reviewer is correct that SIM-LC-MS can be used to quantify the concentration of various analytes. However, as explained above, our post reaction mixtures contain an immense amount of products, which only enables a qualitative estimation and not quantitative analysis via SIM-LC-MS. Moreover, our mixtures contain metal cations, which can affect ionization efficiency and ionization patterns, which make accurate quantification extremely analytically challenging. As a feasibility test for determination of glucose consumption using SIM-LC-MS, following the reviewer's comment, we prepared calibration standards of glucose monomers of

2.5-17.5 μM and analyzed them with LC-MS using SIM mode (operated at either 70V or 90V). We defined glucose m/z species corresponding to ionization $[M-H]^-$ and $[M+HCOO]^-$ and integrated the peak area of both species. A calibration curve (peak area as a function of glucose concentration) was constructed. The dry-down products of glucose in the absence of metal ions were rehydrated to give 100 mM glucose referring to the initial concentration prior to reaction. The rehydrated samples were further diluted either 500 or 1000 times to fit into the calibration curve.

The obtained calibration curves are shown in the figure below:

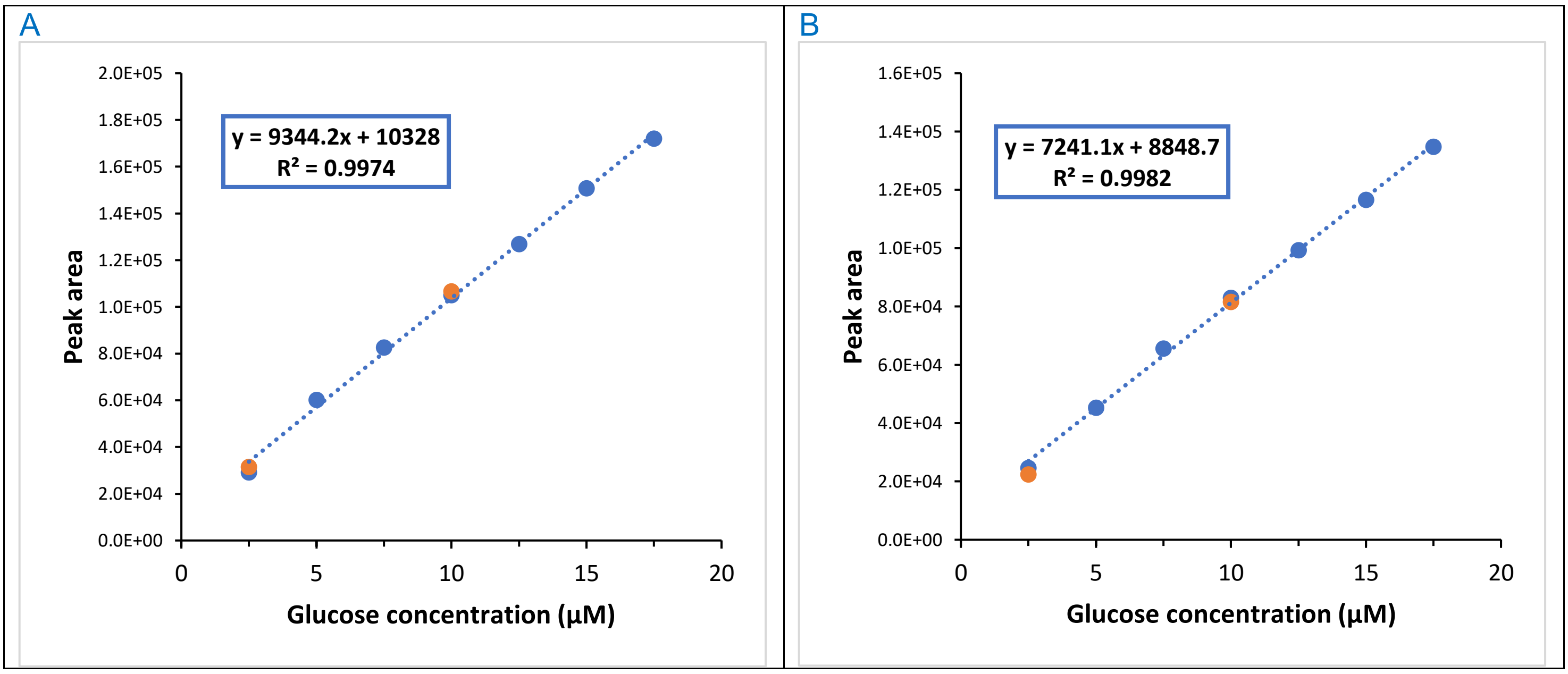

The calibration curve obtained at fragmentor voltage of 70V (panel A) and 90V (panel B) showed high linearity. Orange points are quality control samples (independent preparation of glucose standards at a concentration of 2.5 μM and 10 μM). The quantitative analysis of glucose control samples resulted in 93 ± 1% consumption of glucose for both tested voltages. In NMR analysis the consumption of glucose was 86 ± 1%. The difference between the two methods (7%) might be attributed to the fact that quantitative NMR analysis underestimates the consumption of glucose since in some cases there is an overlap between the signals of the reducing end of glucose monomers and glucose oligomers, resulting in an underestimation of glucose consumption by ^{1}H-NMR. As mentioned, while it is possible in principle to do such quantitative analysis using SIM-LC-MS, due to the huge variety of products, their co-elution, presence of cations, and lack of standards for the absolute majority of these species, such quantitative analysis is beyond the scope of this paper.

Moreover, for this revision, we repeated the SIM analysis for all samples at two analyte concentrations (10 mM, as in the original submission, and 1 mM) and expanded the analysis to include all observed adduct species rather than only the major adducts. This more comprehensive treatment led us to refine the interpretation of these results in several cases. We modified the paragraph describing SIM results as follows:

> "Effect of various metal ions on the product distribution in glucose oligomerization reactions. The dry-down reaction of glucose under acidified conditions produced oligomers of various sizes, as indicated by ESI-MS measurements. We utilized targeted LC-MS to perform a qualitative analysis of glucose monomers as well as glucose oligomers (dimers up to nonamers) in

the presence or absence of metal ions. This analysis is only qualitative due to the huge variety of products, their co-elution, presence of ions, methodological limitations, and lack of standards for the absolute majority of these species. Notably, the combinatorial variety of glucose oligomerization products is immense. Assuming the cyclic form only, the number of products would be 8n-1 (where 'n' is the number of glucose units). For instance, the number of different trimers assuming the cyclic structure would be 64 [two linkages, each of which can be either α or β (1→2, 1→3, 1→4, 1→6)]."

We also modified Figure 5 as follows:

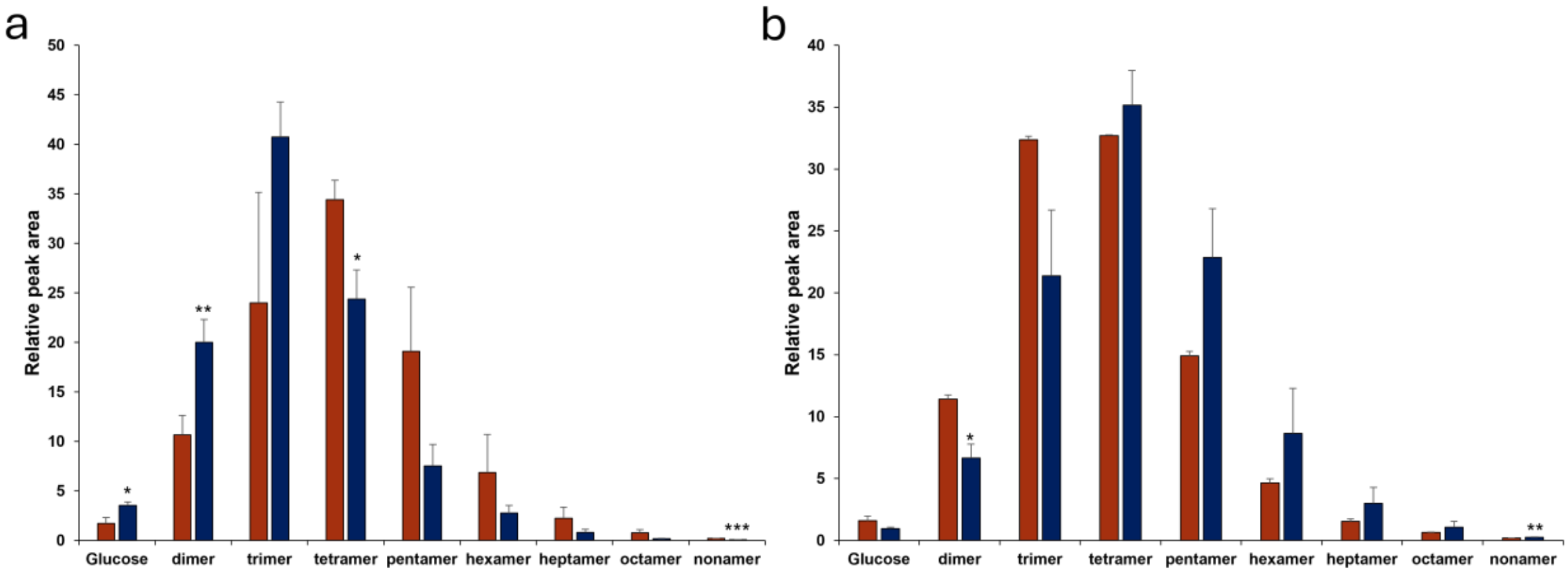

Fig. 5: Evaluation of the degree of D-Glucose oligomerization based on SIM-LC-MS. Glucose product distribution after one week of dry-down at 50 °C in the presence (blue) or absence (red) of Al^{3+} (**a**) or Fe^{2+} (**b**) under acidified conditions. Values are presented as the mean values of three independent replicate analyses with standard deviation. P-values were calculated via a t-test (* indicates $p < 0.05$, ** $p < 0.01$, *** $p < 0.001$).

We added these results as Supplementary figures in the SI (Supplementary Figs. S165-S176). We also added the following to Materials and Methods:

"**Liquid Chromatography-Mass Spectrometry.** LC-MS analysis was performed using an Agilent 1260 quaternary pump and an autosampler with a DAD UV-Vis detector, coupled to an Agilent G6135 single quadrupole. Samples (either 1 mM or 10 mM) were injected onto an Agilent Poroshell 120 EC-C18 column. Column was held at 20 °C. Solvent A: Water (0.1 % formic acid); solvent B: ACN. Flow rate: 0.3 mL/min. Acquisition method: (A%) 100% (0-4 min), linear-gradient (A%) 78.6% to 77% (4-14 min), followed by wash linear-gradient (B%) 23% to 100% (14-21 min), (B%) 100% (21-28 min). Injection volume: 10 µL. Capillary voltage: 4000 V. Fragmentor voltage of either 70 V (for Scan mode) and 135 V (for SIM mode).

For SIM, mass values of each analyte (from glucose monomers up to glucose nonamers) were defined. For each analyte with exact mass *M*, three ionization species were considered: $[M-H]^-$, $[M+Cl]^-$ and $[M+HCOO]^-$, as specified in Table S3. In total, 27 masses were monitored. Individual SIM chromatograms of each ionized species and a TIC-SIM chromatogram of all target masses were recorded. Analysis of oligomer formation was carried out by integrating the corresponding peaks in SIM chromatograms of each ionized species. For each species (a monomer or an oligomer), a total peak area was calculated and

distribution profiles of either absolute peak area or relative peak area were constructed.

Relative peak areas (RA_i) were calculated as follows:

$$RA_i = \frac{A_{i,[M-H]^-} + A_{i,[M+Cl]^-} + A_{i,[M+HCOO]^-}}{\sum_{i=1}^{n} A_{i,[M-H]^-} + A_{i,[M+Cl]^-} + A_{i,[M+HCOO]^-}} \times 100\%$$

Where $A_{i[M-H]^-}$, $A_{i[M+Cl]^-}$ and $A_{i[M+HCOO]^-}$ are the peak areas of product *i* obtained for ionized species $[M-H]^-$, $[M+Cl]^-$ and $[M+HCOO]^-$, respectively. *i* is an integer between 1 and 9 that indicates the specific product (monomer, dimer, trimer etc.).

Cumulative profiles were constructed as well, considering the total contribution of product *k* in a cumulative manner:

$$CA_k = \sum_{i=1}^{k} RA_i$$

$$0 < \sum_{i=1}^{k} RA_i \leq 100\%$$

Where CA_k is the cumulative area of species *k,* which is the sum of RA_i from *i*=1 until *i*=*k*. For example, CA_3 is cumulative area of trimers, which is the sum of relative peak area obtained for monomers (*i*=1), dimers (*i*=2), and trimers (*i*=3) species. "

To summarize, our main purpose in this work was to study general trends of glucose oligomerization in the presence of different metal ions. We agree that it will be good in the future to dive deeper into the identification and detailed characterization of all reaction products, but this is beyond the scope of the current work.

Reviewer 3 comment 3. 2) The quantification of glucose consumption by 1H NMR is a rather complex methodology, which is described in detail only in the previous OLEB 2019. In that paper, the quantification of glucose conversion was clearly explained, and the NMR spectra were carefully interpreted, with comprehensive assignments of both hydrogen and carbon atoms based on one- and two-dimensional experiments. However, in the present manuscript, the NMR results are only superficially described. Rough assignments of 1H and 13C signals are provided, and the approach used for quantifying glucose is not clearly defined. Although numerous NMR spectra are presented, the information extracted from them is not clearly shown and the results are not fully conclusive. For clarity and conciseness, the large number of NMR spectra (Figures S50–S89) could be grouped into four summary figures corresponding to the four experimental series: (i) fresh samples – acidic conditions; (ii) fresh samples – basic conditions; (iii) Dry-down samples – acidic conditions; and (iv) Dry-down samples – basic conditions. Each figure could display nine representative spectra, following the layout used in the OLEB 2019 paper (for instance, Figures S31c, S32a, and S32c).

Response to Reviewer 3 comment 3. The reviewer is correct that a detailed interpretation of the glucose reaction NMR spectra and the workflow for quantifying glucose conversion (that we also used in this study) was established and validated in Li *et al*. (originally ref 37). This is exactly why, in the present manuscript, we did not repeat the full assignment because the spectral features used for quantification are the same and have already been rigorously assigned in that prior work.

We have now better clarified our methodology for determining the conversion of glucose into products. We integrated the anomeric proton signals using an internal standard, following Li *et al.* (originally ref 37). The two relevant peaks, at ~4.64 ppm and ~5.22 ppm, correspond to the β- and α-anomeric hydrogens of glucose, respectively, observed in D_2O at room temperature. These peaks represent the reducing-end anomeric hydrogens, and changes in their integrals reflect glucose conversion during the reaction.

The purpose of the ¹H-NMR analysis was only to determine the amount of reacted glucose, not to perform a detailed product characterization. Due to interference for some of the anomeric signals in drying reactions, to improve accuracy we integrated only half of each anomeric monomeric signal doublet in dried samples (and multiplied by two). We used an internal standard (3-(Trimethylsilyl)-1-propanesulfonic acid-d6 sodium salt) for calibration of integration and chemical shifts. Moreover, since at 25 °C the HOD peak strongly overlaps with the β-anomeric signal, we employed measurements at 20 °C for all samples, in which the HOD resonance shifts downfield, thus improving the accuracy of our quantification method. We used the following equation for the calculation of conversion:

$$\%\ conversion = \left(1 - \frac{I_{dd}}{n * I_f}\right) * 100\%$$

Where: $I_f = \frac{I_\alpha + I_\beta}{I_{standard}}$; $I_{dd} = \frac{I_\alpha + I_\beta}{I_{standard}}$

I_f - integrated sum of anomeric peaks for fresh samples

I_{dd} - integrated sum of anomeric monomeric peaks for dried-down samples

n – dilution factor between fresh and dried samples, where $n = \frac{C_{dd}}{C_f}$ (C- concentration; for samples in D_2O n=5; for samples in DMSO n=1.5)

We added this detailed explanation in Materials and Methods. We also added the full spectra and relevant integrals of all samples as a new Supplementary file (NMR spectra integrations).

Please also see response to Reviewer 1 Comment 3 for a detailed explanation about the revised modifications.

Regarding the comment about clarity and conciseness, we thank the reviewer for the suggestion to add stacked spectra to the SI. We concluded that while stacked representations improve visualization, they can obscure some of the underlying information. Hence, we decided to both keep the original overlaid spectra and also add new stacked spectra with arrows and boxes that clearly indicate the anomeric hydrogens used for quantification (new Supplementary Figs. S120-S123). Here is an example of a stacked graph of a dry-down reaction of glucose in the absence or presence of various metal ions under acidified conditions:

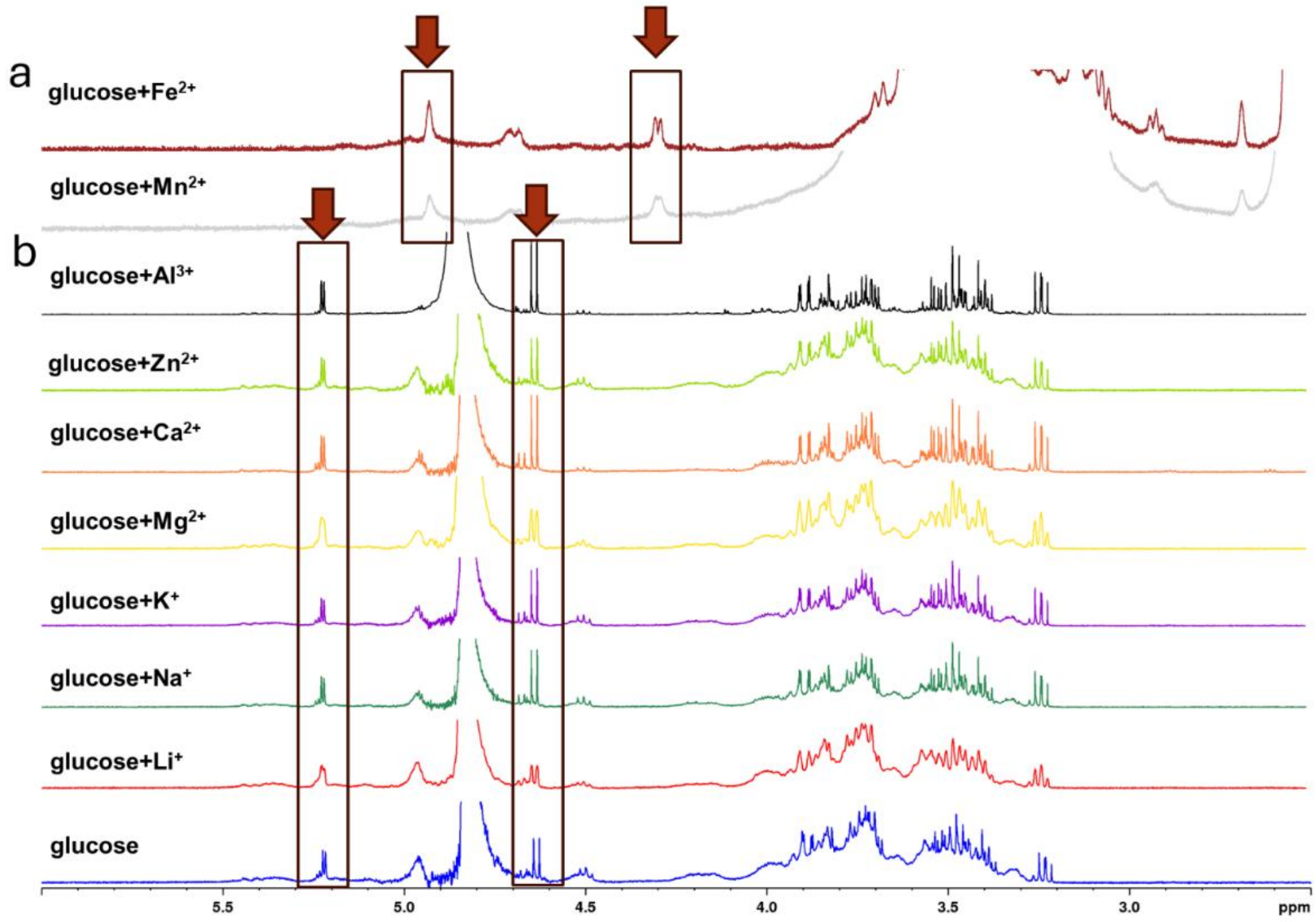

^{1}H-NMR spectra of dry-down reaction of glucose in the absence or presence of various metal ions under acidified conditions. Solutions were prepared following drying at 50 ˚ C for seven days. The products were dissolved in DMSO-d_6 (a) or D_2O buffer (pH=2.66) (b).

Reviewer 3 comment 4. 3) The ESI–MS data appear to have been treated somewhat carelessly, which may lead to inaccurate or misleading conclusions. Please unify the m/z scale (from 50 to 1500) across all mass spectra (Figures S19–S54) to allow consistent comparison. In addition, the mass peak labelling should be carefully revised and correctly positioned. For example, in Figure S25a, a peak around 600 amu is assigned to 7Glu + Ca, whereas this species should have a molecular mass of approximately 1193 amu. Similar inconsistencies are observed in Figure S27a. Although I have not examined every spectrum in detail, the authors are strongly encouraged to perform a thorough revision of all the mass spectra. Furthermore, it is unclear why nonamers are identified by LC–MS but not by ESI–MS. The authors should clarify this discrepancy and specify the limits of detection for both techniques.

Response to Reviewer 3 comment 4. We now unified m/z scale (from 50 to 1500) across all mass spectra for the consistent comparison, as suggested. To improve clarity, we have added Table S2, which presents all relevant m/z values for positive-mode ESI-MS. Regarding the 600 amu peak in Figure S25a (now Supplementary Figure S27a), we apologize for the oversight. This is indeed the correct assignment (7Glu + Ca) but we didn't label the peak correctly in the figure legend, as it is $[M+Ca]^{2+}$, hence z=2 in m/z value (and not [M+Ca]+ as originally written). We verified once again all of the assignments in the MS data and corrected the charge assignments in the figure legends where necessary so that the species now correspond to their actual charges (for example, $[M+Ca]^{2+}$).

We added to the materials and methods:

> "For each type of species in positive mode, a set of *m/z* values was defined according to Table S2."

Regarding why nonamers are observed by LC–MS but not by direct-infusion ESI–MS, the key issue is ion identification in an unseparated, complex mixture. In direct infusion, co-ionizing components (e.g., monomers, oligomers, salts) compete for charge and reduce analyte response, lowering effective sensitivity. Chromatographic separation mitigates these effects to some extent by temporally isolating species and reducing co-elution/competition in the ESI source. Accordingly, we used SIM LC–MS for qualitative analysis because targeted monitoring of selected m/z values improves signal-to-noise and lowers detection limits compared with full-scan acquisition.

Reviewer 3 comment 5. 4) I believe that the assignment of the oligomers by LC–MS was based on the SIM mass spectra; however, why do the species from the tetramer to the nonamer appear at the same retention time (Fig. 5a and Figs. S117–S125)? In line with the above comments, why were commercial standards not used for the calibration of the LC–MS analysis? On the other hand, in any case the conversion of glucose seems to be incomplete. The absence of a relative intensity scale in Figs. S118–S125 makes it difficult to interpret the data properly. What is the final concentration of glucose with respect to its oligomers? Moreover, using a logarithmic scale in Figs. 5b–5e would allow a clearer visualisation of the semiquantitative analysis of the different oligomers shown in the same graphs, especially if points rather than bars were used. In fact, these four figures could be conveniently summarised in a single one by employing a logarithmic scale.

Response to Reviewer 3 comment 5. Yes, the assignment of the oligomers by LC–MS was based on the SIM mass spectra. As mentioned above in response to Reviewer 3 Comment 2 and response to Reviewer 1 Comment 8, our main purpose in this work was to study general trends of glucose oligomerization in the presence of different metal ions. In this revision we included standards, but due to the immense chemical space of product oligomers, we cannot quantify the abundance of each individual oligomer due to the limitations of the analytical techniques used. We agree that it will be good in the future to dive deeper into the identification and detailed characterization of all reaction products, but this is beyond the scope of the current work.

Regarding the question about similarities in retention times of oligomers, we understand that the previous stacked presentation of chromatograms was not visually clear, and now added the chromatograms for each oligomer separately for better clarity (Supplementary Figs. S141-S164). Our SIM analysis generally showed that longer oligomers (heptamers up to nonamers) elute together after about 9 min, supposedly unlike shorter oligomers (trimers up to hexamers) that elute as a cluster. Our chromatography method was intentionally optimized to provide improved resolution for shorter oligomers, whereas the longer oligomers were less well resolved and eluted within a narrower retention-time window. However, this method was selected because it provides improved sensitivity of longer oligomers in SIM (single ion monitoring) mode. Because the higher oligomers were present at relatively low abundance, maximizing signal intensity was more important in this case than achieving full chromatographic separation of each individual longer oligomer (which is practically impossible due to combinatorics, as discussed in detail in response to Reviewer 3 Comment 2). Please also see detailed response to Reviewer 1 Comment 8.

We have now clarified this in the revised manuscript as follows:

> "Since the chromatographic separation was achieved on a C18-column, retention time was expected to increase with oligomer length. However, due to the presence of multiple products, oligomers of a certain chain length typically eluted as a cluster. This was observed predominantly for trimers, tetramers,

pentamers, and hexamers that exhibited continuous elution (Supplementary Figs. S141-S164). Due to the choice of chromatographic separation, the longer oligomers eluted as a single peak or as a front-cluster (depending on the concentration) (Supplementary Figs. S141-S164)."

Regarding the comment about the final concentration of glucose with respect to its oligomers, we can only estimate the concentration of glucose monomer based on quantitative ^{1}H-NMR and qualitative SIM-LC-MS, but we cannot estimate the exact concentration of its oligomers. Please refer to response to Reviewer 3 Comment 2.

Regarding the comment about the relative intensity scale, for better visualization, we have now added all individual SIM chromatograms for each species (i.e., monomer and oligomers, new Supplementary Figs. S141-S164). Regarding the comment about a logarithmic scale, it is no longer relevant as we replaced Fig. 5 to show the product distribution of glucose species (see response to Reviewer 3 Comment 2).

Reviewer 3 comment 6. 5) The ATR–FTIR spectra are presented in a rather inconsistent manner throughout the manuscript. The quality of the recorded spectra should be improved, and the number of figures reduced, as suggested above for the 1H NMR data. After carefully examining all the FTIR spectra, I was unable to identify any discernible features around 1050 cm^{-1}. Likewise, in Figure 2a of the main text, the assignment of the FTIR bands is neither clearly explained nor properly marked. The bands are neither indicated on the spectra nor adequately discussed. The authors are therefore encouraged to enhance both the quality and the clarity of the FTIR data, ensuring that all relevant spectral bands are correctly identified and assigned. Alternatively, if the FTIR results do not provide significant or complementary information regarding the formation of oligomers—particularly when compared with the data obtained from NMR (if they would properly described) and ESI–MS—this section could reasonably be omitted from the manuscript.

Response to Reviewer 3 comment 6. We agree with the reviewer that the FTIR data were not sufficiently elaborated and spectral differences were not visually clear in the original version of the manuscript. We do consider these data informative, as they provide complementary evidence regarding both glycosidic bond formation and metal-ion interactions, but this was not explained clearly enough. We have therefore revised this section to improve both the quality of presentation and the discussion of the relevant spectral features. We now discuss the utilization of FTIR as an indication for both the formation of new glycosidic bonds at ~1050 cm^{-1} [88] as well as complexation with metal ions. We added the following text to Results and Discussion:

> "FTIR can be indicative of the formation of new glycosidic bonds at ~1050 cm^{-1} as well as complexation with metal ions. [88,92-94] An indication for the formation of new glycosidic bonds was observed via broadening of the C-O-H peak (~1050-1000 cm^{-1}) upon drying reactions (Fig. 2a and Supplementary Figs. S2-S20).[93] An indication for interactions between glucose saccharides and metal ions, which might be indicative of complexation, was observed in frequencies in the range of ~1300-800 cm^{-1}. For example, such differences were noted in the presence of Ca^{2+} under acidified conditions. The C-O-H peak positions for fresh and dried samples in the presence of Ca^{2+} were 1024 cm^{-1} and 1017 cm^{-1}, respectively. In contrast, the C-O-H peak positions for the fresh and dried samples in the absence of Ca^{2+} were 1010 cm^{-1} and 1009 cm^{-1}, respectively."

Moreover, for improved clarity we modified Figure 2a to include peak annotations while zooming in into the relevant region in the spectra. We included stacked spectra of

glucose (fresh or dry) in the presence or absence of Ca^{2+} in order to highlight and discuss some specific observed differences:

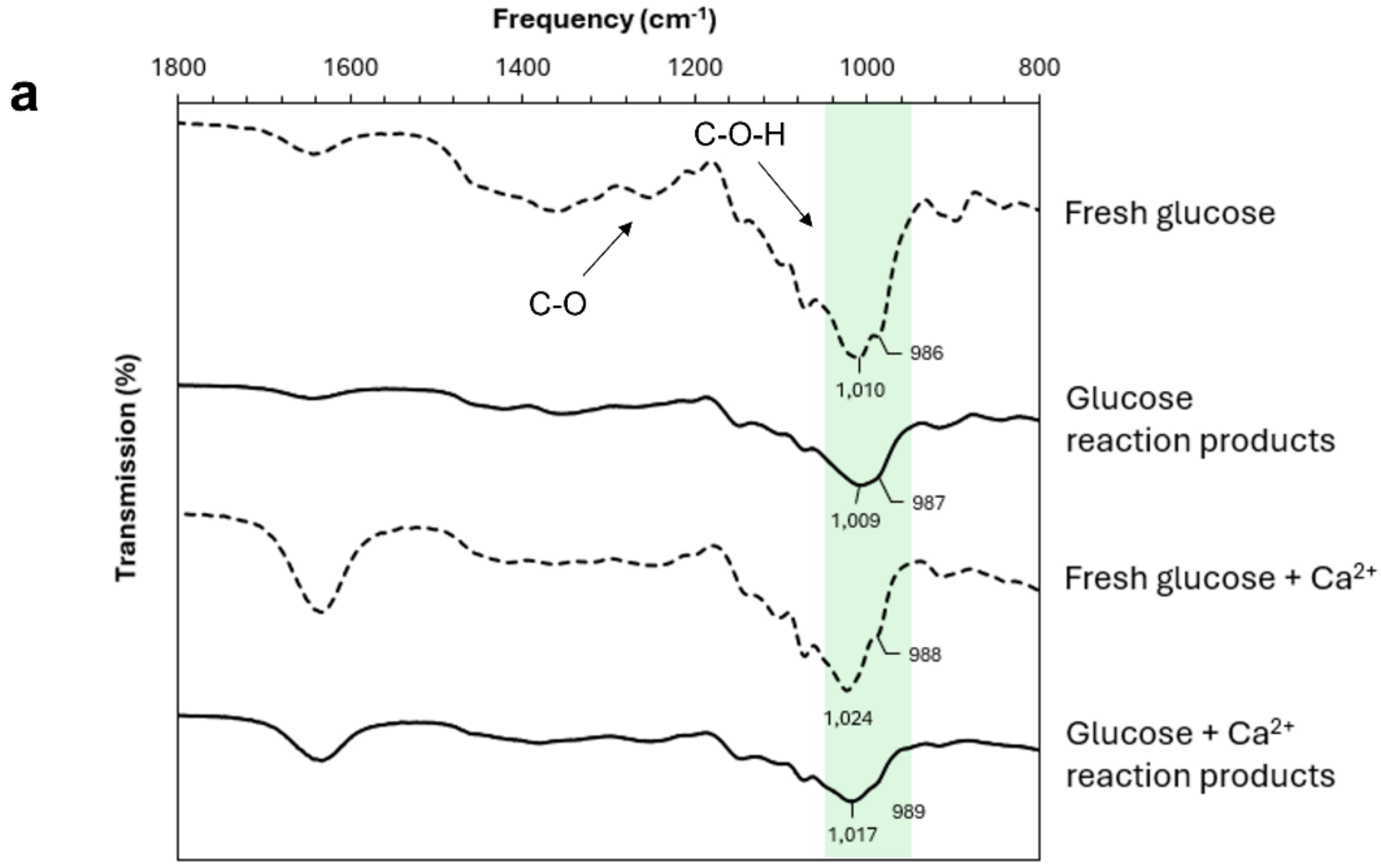

Fig. 2: Characterization of dry-down reactions of D-Glucose under acidified conditions via FTIR and MS analysis in the absence of metal ions. a An FTIR spectra stack of D-glucose in the absence or presence of Ca^{2+} before (dashed line) and after drying for one week at 50 °C (solid line).

Following the reviewer's suggestion, for better clarity we now present the data as an overlay of dried and fresh samples for each metal ion (Supplementary Figures S2-S19). We also added FTIR analysis of standards (Supplementary Figure S20).

Reviewer 3 comment 7. 6) What is the initial pH of each experiment? This is a very important point. The oligomerization of glucose is an acid-catalyzed process, as previously demonstrated and indicated in the present manuscript. A low pH favors the formation of oligomers. However, not all the salts used are neutral, and therefore the initial pH in each of the two experimental series (HCl and NaOH) may differ for the various salts. For a better interpretation of the results, Figure S55 and the associated discussion should consider the increase in pH rather than the absolute final pH value of each experiment. Thus, please indicate the initial and final pH values for each experiment, as well as the corresponding pH increase.

Response to Reviewer 3 comment 7. The reviewer is correct that pH is a crucial parameter for these reactions, and the pH values for these experiments were only briefly mentioned in the original submission. We have now included the full information about the initial and final pH values for the different reactions in the SI. Table S1 lists both the initial and final pH values of all samples. We discuss this as follows:

> "In the samples that contained glucose and either Mn^{2+}, Zn^{2+}, or Fe^{2+}, trimers and tetramers were also observed, which might be explained by the lower pH (~5-6) of these product mixtures following one week of dry-down (Supplementary Fig. S61 and Table S1). The decrease in pH levels can be attributed to the formation of insoluble hydroxides in the mixture. This observation is explained mechanistically by the fact that acidic pH drives protonation of the anomeric oxygen and hence increases the rate of the reaction (Fig. 1b). Indeed, the pH of the glucose sample in the presence of Al^{3+}

was higher than that of the other samples (~4 instead of ~2), and the oligomerization extent in its presence was lower.“

Reviewer 3 comment 8. Minor comments

1) The manuscript could be rewritten to help the reader better appreciate the significance of the results. There is a large amount of data, but even for a specialised reader it is not easy to extract its full value. I believe that the Results and Discussion section should be reorganised. For example, the results obtained by each technique could be presented separately (technique by technique), followed by a comparison of the information provided by each of them, a discussion of the internal consistency of all the data, and finally a comparison with the only previous study on glucose polymerisation. Finally, a especific section could then address the mechanistic aspects of oligomerisation (coordination with metal cations, influence of pH, pH variations during the reactions and so on).

Response to Reviewer 3 comment 8. We agree that the manuscript contains a large amount of data and that the significance of the results should be made easier to follow for the reader. In response, we have done a major reorganization of the manuscript to improve its clarity and flow. We now first focus on the different characterization methods (MS, FTIR, NMR, and SIM) and then discuss the possible mechanistic explanations while integrating the various characterization methods. We expanded and reorganized this latter part of the Results and Discussion under a new subtitle, ‘Possible explanations for the observed effect of metal ions on glucose oligomerization reactions’. We also added clarifications that we believe improved the clarity of our findings. For example, we state the following in Results and Discussion:

> “**Possible explanations for the observed effect of metal ions on glucose oligomerization reactions.** Taken together, while our NMR measurements clearly revealed an inhibitory effect in terms of overall glucose conversion, our qualitative LC-MS analysis indicated that there might be some differences in oligomeric distribution in the presence of specific metal ions. As the complexation of carbohydrates with metal ions has been previously reported,[92,100-102] one could expect such complexation to affect both reaction yield and oligomerization degree, as was also reported for oligomers such as depsipeptides.[61] Our LC-MS analysis suggested that for some metal ions (e.g., Ca^{2+}, Fe^{2+}, and Al^{3+}), product formation was slightly altered compared to glucose control (in the absence of metal ions).”

Overall, we believe that these revisions make the manuscript easier to navigate.

Reviewer 3 comment 9. 2) I have noticed some differences in the glucose conversion values obtained in the control experiments without salts, compared with those reported in the previous paper. Please place these differences in context within the discussion.

Response to Reviewer 3 comment 9. This is a good point. Indeed, Li *et al.* (2019) reported a much lower conversion (~11%). We believe that the reason for this difference lies in differences in reaction conditions: in our study, we added 2.5 µmol of HCl, whereas Li *et al*. used only 0.25 µmol. This substantial difference readily explains the higher conversion of glucose observed in our results, as acetal bond formation is acid-catalyzed. To address this difference, we have added the following clarification in the Results section:

> “Notably, Li *et al*. reported a much lower glucose conversion after one week of drying (11%),[42] compared with our results (86%). This difference

> can be attributed to the higher amount of HCl (a 10-fold difference) used in this study, resulting in a much higher conversion due to acid-catalyzed acetal bond formation."

Reviewer 3 comment 10. 3) What is the reason for the change of solvent used to record the NMR spectra (DMSO for the $FeCl_2$ and $MgCl_2$ experiments)?

Response to Reviewer 3 comment 10. Both Mn^{2+} and Fe^{2+} are paramagnetic ions, which directly influence the NMR analysis. The resulting peaks are very broad, making quantification not reliable. To minimize this effect, we used a cation-exchange resin to remove these cations (as specified in M&M). Although the resulting spectrum showed improvement, there was still some interference from the HOD peak. The samples, however, are readily soluble in DMSO, and in this solvent, the HOD peak does not appear in the anomeric region. Hence, in the case of Mn^{2+} and Fe^{2+} we decided to work with DMSO as a solvent for more accurate proton quantification.

We added the following clarification in the Materials and Methods section:

> "For samples containing glucose and either Mn^{2+} or Fe^{2+}, pre-treatment was carried out for direct removal of metal ions due to interference with the NMR signal due to their paramagnetic nature."

Reviewer 3 comment 11. 4) The introduction is well referenced; however, it should be reorganised. For example, on page 2: "While abiotic polymerisation of peptides and nucleic acid polymers has been studied extensively,21–36 abiotic oligosaccharide polymerisation was reported only once, in drying reactions of glucose.37"and on page 3: "In the context of prebiotic chemistry, metal-catalysed peptide and RNA bond formation has been demonstrated.21,51–53,56–65 […] While it is now known that oligosaccharides can form abiotically,37 it is unknown whether metal ions have any effect on the polymerisation of sugars." These ideas should be unified for better coherence. Additionally, in the introduction, the following paragraph seems more appropriate for the Conclusions section: "We found that while metal ions typically inhibited the overall conversion of glucose, some promoted the formation of longer oligosaccharides. These include Fe^{2+}, K^{+}, Mg^{2+}, and Li^{+}. For example, while glucose conversion was ~67% in the presence of Fe^{2+}, compared to ~86% in its absence, it led to a significant increase in the abundance of octamers (by ~1.7-fold) and nonamers (by ~2.3-fold). The Ca^{2+} ion, on the other hand, inhibited the formation of longer oligomers."

Response to Reviewer 3 comment 11. We have reorganized parts of the introduction for clarity and shortened the last paragraph (which summarizes the results).

Response to Reviewers

Reviewer #1 (Remarks to the Author):

Reviewer 1 Comment 1. I can see that in this revised version the authors have made a significant effort to improve the analytical aspects of their work. Their responses to my comments are convincing, and the paper now shows much more consistent data analysis supporting their conclusions, and a better balanced interpretation of the results. Given that the relevance of the results is clear, I am happy to see that the work can be published in Commun. Chem.

Response to Reviewer 1 Comment 1. We thank the reviewer for their positive feedback and for their constructive comments in the previous round. We believe that addressing these comments has significantly strengthened our manuscript.

Reviewer #2 (Remarks to the Author):

Reviewer 2 Comment 1. The authors carried out a very careful revision, in which they provide convincing new data for the effect of A3+ cations on the studied polymerization reaction. For this reason I strongly suggest publication of this fine work in Communications Chemistry in its present form.

Response to Reviewer 2 Comment 1. We thank the reviewer for their positive feedback and constructive suggestions, which have helped improve the quality of our manuscript.

Reviewer #3 (Remarks to the Author):

Reviewer 3 Comment 1. The authors have made a significant effort to improve the manuscript and have performed additional experiments and analyses. Despite these improvements, several points still require clarification, further detail, and refinement for the work to merit publication in Communications Chemistry. The volume of experimental work is huge, clearly representing many hours of effort, and deserves to be presented in a manuscript of commensurate quality.

Response to Reviewer 3 Comment 1. We thank the reviewer for recognizing the substantial effort invested in this study and for the constructive evaluation. In response to the reviewer's comments, we have carefully revised the manuscript to improve its clarity, rigor, and presentation. In particular, in this revision we clarified the rationale for the two concentrations used in the SIM-LC-MS analysis, expanded the discussion of the positive-mode MS data with representative examples of sugar–metal ion adducts, added statistical analysis and significance annotation to the glucose-conversion data in Fig. 4, clarified the interpretation and limitations of the SIM-LC-MS-based analyses, including addition of a new SIM-LC-MS calibration figure and corresponding methodological details, substantially revised the discussion of oligomer distributions in Fig. 5, and expanded the main-text figures by moving key mechanistic schemes from the Supporting Information into the manuscript (new Fig. 6). We believe that these changes have strengthened the manuscript and improved the traceability of the data and conclusions throughout.

Reviewer 3 Comment 2. Below, I provide my comments following the order of the manuscript:

Results and Discussion. Section 1

1) In the opening paragraph of Section 1, it would be helpful to clearly state that two experimental series were conducted: one under acidic conditions and one under basic conditions, along with the rationale for selecting these experimental setups. Additionally, in this revised version, the authors have carried out further experiments at an initial concentration of 10 mM to complement the 1 mM SIM-LC-MS analyses from the original submission. The motivation for this expanded experimental scope should also be briefly introduced in this first paragraph.

Response to Reviewer 3 Comment 2. We added the information about the conditions in the main text as follows:

> "D-Glucose (Glu) was dried at 50 °C for one week in the presence or absence of 1 eq. of metal ions under unbuffered conditions adjusted by the addition of HCl or NaOH (Fig. 1). HCl was added to promote oligomerization, as acetal bond formation is acid-catalyzed. NaOH was added to examine the effects of metal ions under alternative conditions, even though this environment is not expected to favor sugar oligomerization."

We agree that the rationale for using two concentrations in the SIM-LC-MS analysis should be clarified. We considered that this clarification is better placed in the Results and Discussion section describing the SIM-LC-MS analysis, rather than in the introductory paragraph. We therefore added the following sentence:

> "To improve detection across products with a broad range of abundances, SIM-LC-MS analyses were therefore performed at two initial glucose concentrations (1 mM and 10 mM), as higher concentration facilitated detection of lower-abundance species, whereas lower concentration allowed clearer evaluation of more abundant species."

Reviewer 3 Comment 3. 2) If feasible, and without requiring excessive additional work (given the already extensive number of figures in the Supporting Information), it would be beneficial to maintain a consistent color scheme across Figures S2–S20.

Response to Reviewer 3 Comment 3. Fixed for consistency.

Reviewer 3 Comment 4. 3) In Figure S61, the caption should specify how glucose conversion was calculated, in order to facilitate the reader's understanding of both the methodology and the results.

Response to Reviewer 3 Comment 4. We added the following clarification in the figure legend:

> "Integrations were based on ^{1}H-NMR resonances of the anomeric protons before and after the dry-down reactions (see Materials and Methods)."

Reviewer 3 Comment 5. 4) At the end of this section, the authors state: "We also detected sugar–metal ion adducts via MS in fresh, non-dried samples." Please provide specific examples of the adducts identified in this study and explain these results in greater detail. As currently written, the paragraph is rather opaque and difficult to follow. A clearer and more detailed discussion, including representative examples and an indication of whether adduct formation depends on the initial pH, is needed. While the ESI-MS results obtained in negative ionization mode are reasonably well explained,

the discussion of the positive ionization mode is not. Please improve the clarity and depth of the discussion in this regard.

Response to Reviewer 3 Comment 5. We thank the reviewer for this comment. To address this, we revised the paragraph to include specific representative examples of the detected sugar–metal ion adducts and added a reference to Table S2, which lists the species that were detected via positive-mode. We also now clarify that these signals are consistent with sugar–metal ion adduct formation, but that such adducts detected by ESI-MS do not necessarily represent pre-existing solution-state or reaction-state complexes, as some may form during the ionization process itself. Regarding the effect of initial pH, we did not observe major qualitative differences in adduct formation between fresh acidic and basic conditions, and therefore we do not interpret pH as a dominant factor in this part of the analysis. We modified the relevant paragraph as follows:

> "In the MS-positive mode, we observed adducts involving sugars (monomers and oligomers) and cations at a 1:1 molar ratio (e.g., $[Glu+Li]^{+}$, $[Glu+Mg]^{2+}$, Table S2), in accordance with previous studies that investigated sugar-metal ion interactions.[90,91,93] For instance, Mg^{2+} forms a stable complex with one glucose molecule and four water molecules.[92] On the other hand, it is known that at a 1:1 molar ratio of Ca^{2+} and glucose, a 7-coordinated complex is observed, whereas for a 1:2 molar ratio (Ca^{2+}: glucose), the coordination number is eight, in which each metal ion is bound to two units of monosaccharide.[93] We also detected sugar-metal ion adducts via MS in fresh, non-dried samples. These observations are consistent with sugar–metal ion association, although we note that adducts detected by ESI-MS do not necessarily correspond to pre-existing complexes in solution or in the dry-down reaction mixture, since some adduct formation may also occur during ionization in the instrument. We further note that no major qualitative differences in positive-mode adduct formation were observed between fresh samples adjusted with HCl or NaOH (e.g., Figs. S43 and S53)."

Reviewer 3 Comment 6. Results and Discussion. Section 2

5) At the end of the first paragraph, the authors state: "Notably, we acknowledge that integration of the anomeric protons is an underestimation of glucose conversion due to a partial overlap between the signals of the reducing end of glucose monomers and glucose oligomers." This underestimation is in fact demonstrated when glucose conversion is quantified by LC-MS. The limitations of NMR-based conversion estimates compared to LC-MS measurements are very well explained in the response-to-reviewers letter, as is the impracticality of using LC-MS to determine glucose conversion for all experiments. I strongly encourage the authors to include this information in the manuscript—briefly in the main text and in greater detail (as already done in the response letter) in the Supporting Information.

Response to Reviewer 3 Comment 6. We have now added a brief clarification in the main manuscript noting that ^{1}H-NMR likely underestimates glucose conversion relative to SIM-LC-MS. We also added a new supplementary figure (S177) showing the glucose calibration curves used for the SIM-LC-MS feasibility test, and included the corresponding experimental details in the Methods section. Together, these additions clarify both the basis for the comparison between the two methods and the practical limitations that prevented us from extending SIM-LC-MS quantification to all reaction products. Specifically, we added the following text in the Results section:

"To assess whether glucose consumption could nevertheless be estimated by SIM-LC-MS, we performed a feasibility test using calibration curves of glucose standards analyzed in negative ionization mode (Supplementary Fig. S177). For the glucose control reaction, this analysis gave a glucose consumption value of 93 ± 1%, whereas ^{1}H-NMR gave 86 ± 1%, consistent with underestimation by NMR due to partial overlap between the anomeric proton signals of glucose monomers and oligomers. While these results show that SIM-LC-MS can in principle be used to quantify glucose consumption in simple samples, extending this approach for the quantification of all reaction products is analytically challenging because of the large diversity of products, their co-elution, the presence of metal ions, and the lack of standards for most analytes."

We added the following text in the Methods section:

"To evaluate the feasibility of glucose quantification by SIM-LC-MS, glucose calibration standards were prepared at concentrations of 2.5–17.5 μM and analyzed by LC-MS in SIM mode under negative ionization conditions using fragmentor voltages of 70 V and 90 V. For glucose, the m/z values corresponding to the ionization species $[M-H]^-$ and $[M+HCOO]^-$ were monitored, and the summed peak areas of these two ions were used for quantification. Calibration curves were constructed by plotting the total peak area as a function of glucose concentration. Independently prepared quality-control samples at 2.5 and 10 μM were analyzed in the same manner to assess reproducibility. Dry-down glucose control samples were rehydrated to 100 mM glucose, with concentration referring to the initial glucose concentration prior to reaction, and were further diluted 500-fold or 1000-fold to verify that the quantitation is consistent"

Last, we added the following Supplementary Figure:

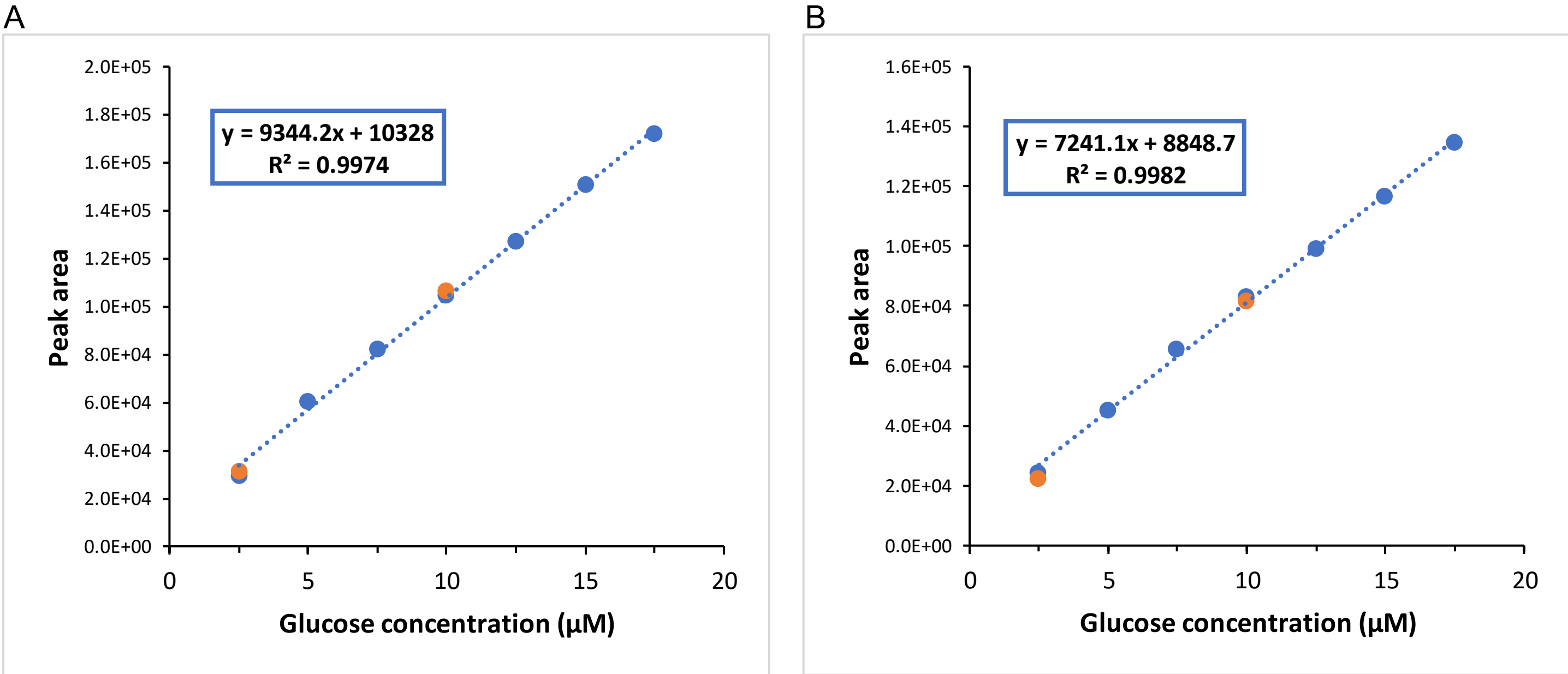

Figure S177. Calibration curves for glucose quantification by SIM-LC-MS. Glucose standards (2.5–17.5 μM) were analyzed by SIM-LC-MS in negative ionization mode using fragmentor voltages of 70 V (A) and 90 V (B). Quantification was based on the summed peak areas of

the glucose ions corresponding to $[M-H]^-$ and $[M+HCOO]^-$. Blue points represent calibration standards used to generate the linear fit, and orange points represent independently prepared quality-control samples at 2.5 and 10 μM. The corresponding linear regression equations and R^2 values are shown in each panel.

Reviewer 3 Comment 7. 6) Figure 4 is not adequately discussed.

Building Figures 4a and 4b required at least 60 experiments and 60 1H NMR spectra, an effort that warrants a thorough analysis and the extraction of meaningful conclusions. In its current form, neither the text nor the figures achieve this. Please perform a statistical analysis to determine whether the differences in glucose conversion across experiments are statistically significant relative to the control. Furthermore, consider whether certain cations produce similar effects on conversion and whether they can be grouped based on statistically significant similarities.

Figure 4c is of limited value, as it only presents mean values. It is already apparent that basic conditions lead to lower glucose conversion. Instead, consider performing a statistical comparison between the acidic and basic series. A proper statistical treatment would significantly strengthen the robustness of the conclusions.

Figure 4d does not meaningfully contribute to the overall discussion unless it is explicitly connected to mechanistic implications regarding metal–sugar complex formation. In addition, the figure presents the electronic configurations of neutral elements rather than their ionic forms.

Response to Reviewer 3 Comment 7. Regarding the comment about Figures 4a and 4b, we have now performed a statistical analysis of the glucose conversion data presented in these figures. Relative to the control, most differences were found to be statistically significant, with the exception of Li^+ and Zn^{2+} under acidified conditions, and K^+ and Al^{3+} under NaOH-adjusted conditions. The question of whether certain cations produce similar effects on glucose conversion is already addressed in the main text. For example, we discuss that under acidified conditions, most metal ions decreased glucose conversion relative to the control, whereas the extent of this effect differed between cations, with some showing only minor effects. We therefore did not add a separate grouping analysis. We now indicate statistical significance in the figures using asterisks and added the corresponding explanation to the figure legend:

> "P-values were calculated via a t-test (* indicates $p < 0.05$, ** $p < 0.01$, *** $p < 0.001$)"

Regarding the comment about Figure 4c, we added error bars to this figure and performed a statistical comparison between the acidic and basic series. We agree that including the variability and the corresponding statistical analysis strengthens the robustness of this comparison.

Regarding the comment about Figure 4d, respectfully, we prefer to keep this panel because we view it as a descriptive comparative framework that helps visualize the metal-specific trends observed in glucose conversion, rather than as a direct mechanistic proof. In the manuscript, Figure 4d is used to show that metals from different groups display distinct patterns of inhibition, and this observation is discussed together with the broader conclusion that multiple factors likely contribute to the

outcome, including binding/coordination, pH, hardness, Lewis acidity, and electronic-structure effects.

We agree that the chemically relevant species in the reactions are the metal ions, not the neutral atoms. However, the purpose of the figure is not to depict the exact electronic structure of the solution-phase complexes, but rather to provide a simple way to organize the tested metals according to their position in the periodic table and valence-shell character, which may relate to broader differences in coordination behaviour. We therefore use the elemental electronic configuration only as a comparative visualization of trends across the tested metal set, while the mechanistic discussion in the text focuses on the ionic forms and their interactions with glucose. This is consistent with our discussion of sugar–metal association, metal-specific effects, and possible coordination of cations to glucose hydroxyl groups and the anomeric position. For example, we say:

> “In order to further understand the metal-specific effects on glucose oligomerization, we plotted the conversion of glucose under acidified conditions against the elemental electronic configuration of the various metals and observed clear trends (Fig. 4d). It is evident that alkaline metals did not dramatically inhibit the reaction. A non-significant trend in conversion indicates a possible correlation between atom size and the extent of conversion inhibition (Li < Na < K). On the other hand, alkaline earth metals inhibited the conversion of glucose to a higher extent than alkaline metals (Ca and Mg). For transition metals, a positive correlation was observed between the number of electrons in the d-shell of the atom and the extent of glucose conversion (Zn > Fe > Mn), with only minimal inhibition for Zn (by ~2.5%). We noted that, also under the addition of NaOH, alkaline earth metals inhibited glucose oligomerization to a greater extent than alkaline metals. The conversion in the presence of Al^{3+} was similar under acidified conditions and upon addition of NaOH: 56% and 54%, respectively. As mentioned above, this can be explained by the similar pH under both conditions (pH ~ 4).”

Following these suggestions, we have revised Figure 4 as follows:

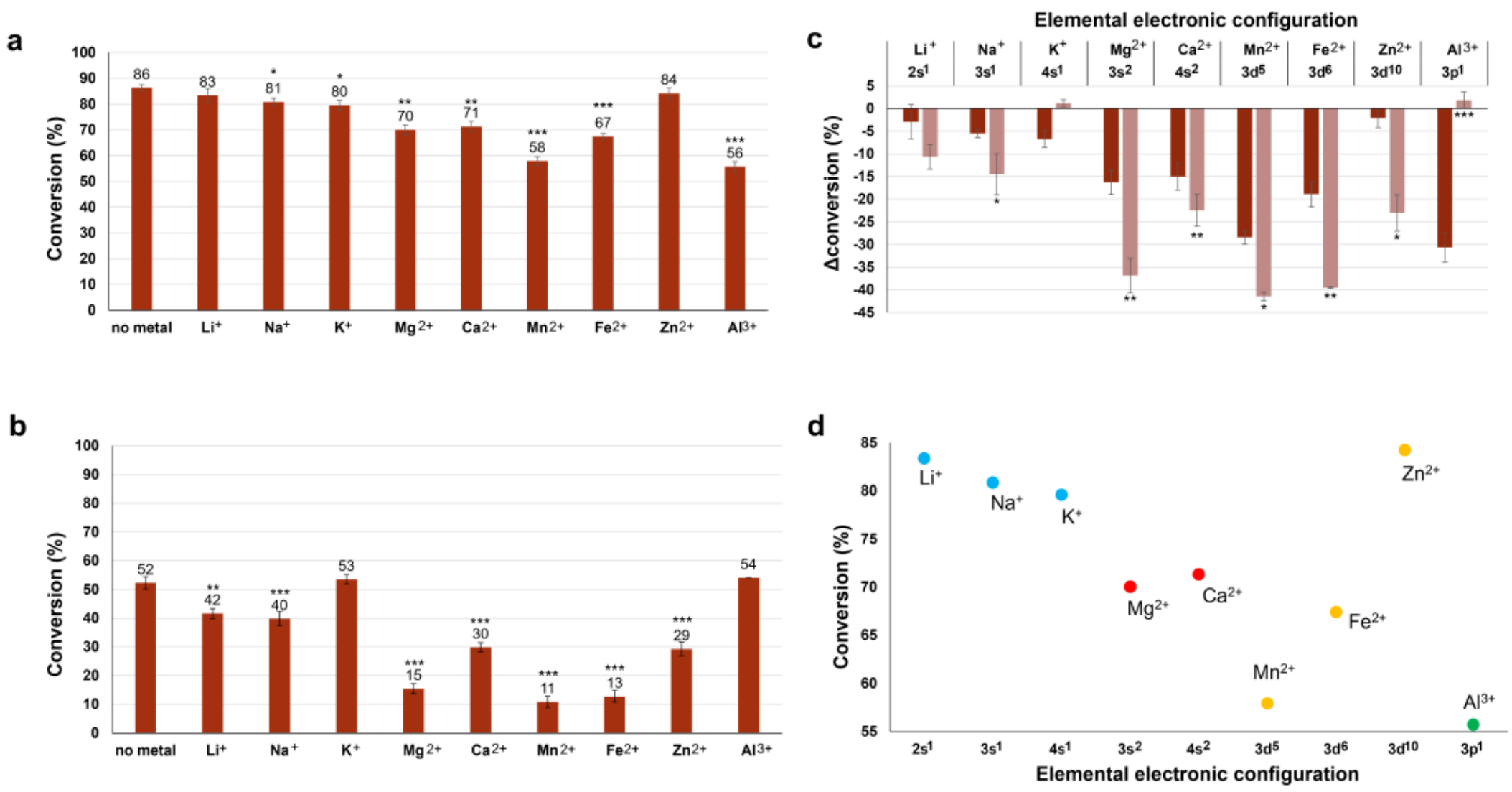

Fig. 4: Quantification of conversion of D-Glucose into products based on ^{1}H-NMR. Glucose conversion after one week of dry-down at 50 °C in the presence or absence of metal ions under acidified conditions (**a**) or upon addition of NaOH (**b**). **c** Difference between the conversions of glucose in the presence of different metal ions under acidified conditions (red) and upon addition of NaOH (pink). **d** A plot summarizing the conversion of D-Glucose under acidified conditions versus the elemental electronic configuration of the tested metals, highlighting trends across the tested metal set. P-values were calculated via a t-test (* indicates $p < 0.05$, ** $p < 0.01$, *** $p < 0.001$).

Reviewer 3 Comment 8. 7) Why is the initial pH for the Al^{3+} experiments set to 4 after the addition of HCl or NaOH? This point is critical for explaining the observed results regarding glucose conversion in the presence of $AlCl_3$ and likely in the generation of oligomers.

Response to Reviewer 3 Comment 8. We agree with the reviewer that pH is a key parameter in these reactions and is likely an important factor underlying the behavior observed in the presence of Al^{3+}. As clarified in the main text, the glucose sample containing Al^{3+} exhibited a pH of ~4 under both acidified conditions and upon addition of NaOH, in contrast to the other samples. This likely contributes to the similar glucose conversion values observed for Al^{3+} in the two experimental series as well as to the lower extent of oligomerization observed in the presence of Al^{3+}. We further note that the persistence of pH ~4 in the presence of $AlCl_3$ under both starting conditions likely reflects Al^{3+}-dependent solution equilibria. Dissolved Al^{3+} is strongly hydrated, and its coordinated water ligands undergo stepwise hydrolysis to form coordinated hydroxide species and release of protons, which makes the solution acidic. Under these conditions, added NaOH can be consumed by shifts in Al^{3+} hydrolysis/speciation equilibria, while added HCl can protonate hydroxide ligands and shift the equilibria toward water-rich complexes, limiting the net change in bulk pH. A detailed investigation of these equilibria is beyond the scope of the present study. We added a short explanation in the manuscript as follows:

> “Indeed, the pH of the glucose sample in the presence of Al^{3+} was higher than that of the other samples (~4 instead of ~2), and the oligomerization extent in its presence was lower, likely due to the distinct Al^{3+}-dependent hydrolysis/speciation environment.“

> “The conversion in the presence of Al^{3+} was similar under acidified conditions and upon addition of NaOH: 56% and 54%, respectively. As mentioned above, this can be explained by the similar pH under both conditions (pH ~ 4)”

Reviewer 3 Comment 9. Results and Discussion. Section 3

8) I regret to say that I find the discussion of Figure 5 difficult to follow. In the second paragraph of this section, it appears that the authors are discussing cumulative peak areas rather than the figure itself. Please ensure that figures included in the main text are explicitly and clearly discussed in the main text. The Supporting Information already contains a large number of figures presenting cumulative area data. This paragraph should be carefully revised, as it is currently quite difficult to understand. While the dataset is extensive, it is not always clear which data and figures are being referred to. Increasing the number of figures in the main text would likely improve clarity. I encourage the authors to consider multi-panel figures that help the reader

better visualize the results. The manuscript should not rely so heavily on general references to the Supporting Information while providing only a cursory discussion of the results.

Response to Reviewer 3 Comment 9. We thank the reviewer for this helpful comment. To improve clarity, we have substantially revised the discussion of Fig. 5 to refer explicitly to the relevant panels and to distinguish more clearly between the relative distribution data and the cumulative distribution data. In addition, we expanded Fig. 5 into a multi-panel figure that now includes both the relative product distributions and the cumulative distributions for the Al^{3+} and Fe^{2+} conditions, so that the main text discussion is less reliant on the Supporting Information. We modified the paragraph as follows:

> "As indicated by the distribution profiles, for most samples, the most abundant oligomers in terms of peak area were trimers and tetramers (Fig. 5). When examining the peak areas of the longest oligomers (heptamers to nonamers), we did not observe significant differences between the samples, except for Ca^{2+} and Al^{3+}, for which these oligomers were formed to a lesser extent compared with the control sample in the absence of metals. Al^{3+} produced the strongest shift in product distribution toward short oligomers (Fig. 5a). This trend is also evident from the cumulative distribution plot (Fig. 5b): in the presence of Al^{3+}, ~80% of the total peak area corresponded to trimers or shorter oligomers, compared with ~50% in the glucose control, and ~95% corresponded to tetramers and shorter oligomers, whereas in most other samples the same cumulative proportion extended to hexamers, and to pentamers in the presence of Ca^{2+} (Supplementary Figs. S165-S176). For Fe^{2+}, the product distribution suggested a modest shift toward longer oligomers (Fig. 5c), which is also reflected in the cumulative profile (Fig. 5d). In its presence, ~36% of the total peak area corresponded to pentamers or longer oligomers, compared with ~22% in the glucose control, consistent with the cumulative distribution profile (Fig. 5c-d and Supplementary Figs. S175-176)."

And we modified the figure as follows:

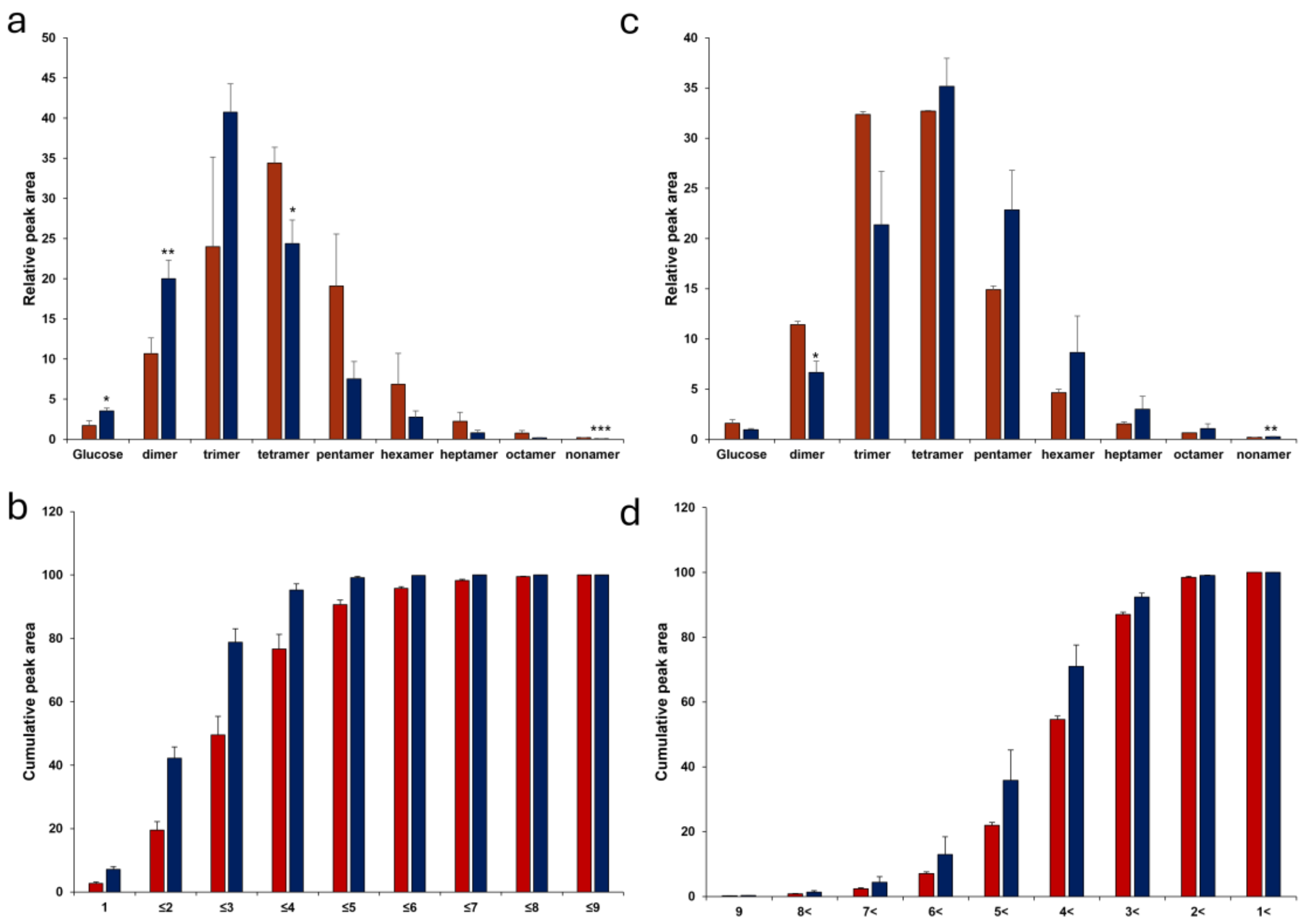

Fig. 5: Evaluation of the degree of D-Glucose oligomerization based on SIM-LC-MS. Glucose product distribution and cumulative distribution after one week of dry-down at 50 °C in the presence (blue) or absence (red) of Al^{3+} (**a-b**) or Fe^{2+} (**c-d**) under acidified conditions. Values are presented as the mean values of three independent replicate analyses with standard deviation. Glucose concentration for the analysis was 10 mM. P-values were calculated via a t-test (* indicates $p < 0.05$, ** $p < 0.01$, *** $p < 0.001$).

Reviewer 3 Comment 10. 9) In this section, the authors should also clearly explain why two sets of experiments were conducted at different concentrations (1 mM and 10 mM). What effect does this difference in concentration have on glucose oligomerization and in the extension of the oligomers? This point is not addressed in the main text, despite the substantial experimental effort and the additional Supporting Information figures it entails.

Response to Reviewer 3 Comment 10. As mentioned in response to Reviewer 3 Comment 2, we agree that the rationale for using two concentrations in the SIM-LC-MS analysis should be clarified. We considered that this clarification is better placed in the Results and Discussion section describing the SIM-LC-MS analysis, rather than in the introductory paragraph. We therefore added the following sentence:

> "To improve detection across products with a broad range of abundances, SIM-LC-MS analyses were therefore performed at two initial glucose concentrations (1 mM and 10 mM), as higher concentration facilitated detection of lower-abundance species, whereas lower concentration allowed clearer evaluation of more abundant species."

Reviewer 3 Comment 11. Results and Discussion. Section 4

10) The manuscript proposes reaction mechanisms and speculates on the possible formation of sugar–metal complexes; therefore, Figures S177 and S178 should be moved to the main text (e.g., as Figures 1c and 1d), clearly indicating their hypothetical nature. Please avoid further transferring key information to the Supporting Information.

Response to Reviewer 3 Comment 11. As suggested, we moved these Figures as new main text Figure 6a-b:

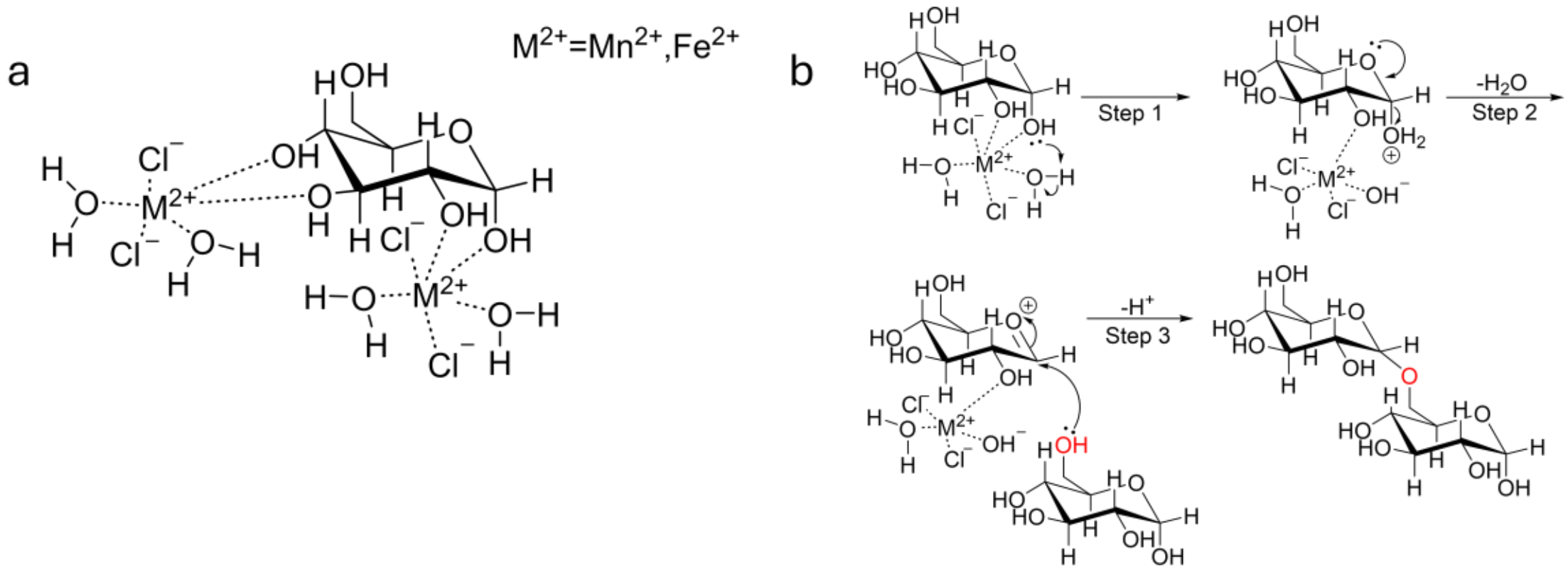

Fig. 6: a A possible complexation of glucose with transition metal ions. **b** Proposed mechanism of acetal bond formation in the presence of metal ions.

Reviewer 3 Comment 12. In any case, I encourage the authors to further improve the writing of the main text and to increase the number of primary figures included in the manuscript. Additionally, the authors may wish to consider including their response-to-reviewers letters if the manuscript is ultimately published. These documents contain a wealth of experimental detail that helps ensure the traceability of the experiments and measurements performed. Moreover, the discussions of the limitations of the spectroscopic and chromatographic techniques, currently only addressed in the response letters, would be of considerable interest both to specialists and to non-specialists, particularly in the context of developing general analytical protocols.

Response to Reviewer 3 Comment 12. As noted, we have further revised the main text and increased the amount of primary figure content included in the manuscript. In particular, we revised some of the discussion throughout to guide the reader more clearly through the datasets, expanded Fig. 5 into a multi-panel main-text figure that explicitly presents both relative and cumulative distribution data, and moved the proposed mechanistic schemes from the Supporting Information into the main text as Fig. 6. We agree that these points are important for traceability and interpretation. We also appreciate the reviewer's suggestion regarding the response-to-reviewers letters and would be happy to have them included alongside the published manuscript.

In this manuscript, the authors present extensive work aimed at elucidating the potential role of several metal cations in the oligomerisation of sugars under plausible prebiotic conditions. Two series of experiments were conducted under acidic and basic pH values, respectively, and under drying conditions, using up to eight chloride salts containing metals considered abundant on the primitive Earth. All experiments were performed in triplicate, demonstrating good reproducibility. Glucose was selected as the model sugar, based on the only previous study that addressed a potential prebiotic synthesis of oligosaccharides. Several analytical techniques were employed for the characterisation of the final reaction mixtures, including ATR-FTIR, ^{1}H and ^{13}C NMR spectroscopy, ESI-MS, and LC-MS. A large amount of data was analysed to determine the influence of the initial pH and the presence of metals on both the conversion of glucose and its transformation into various oligosaccharides. Although this work may be relevant to the field of prebiotic chemistry, given the very limited number of studies concerning the oligomerisation of polysaccharides and their important role in contemporary biochemistry, several inconsistencies and details, must be more thoroughly addressed before the manuscript can be considered for publication in *Communications Chemistry*.

Main comments

1) The main focus of this paper is the conversion of glucose into its corresponding oligomers in the presence or absence of metal cations. It is somewhat surprising that, despite having access to a powerful analytical tool such as an LC–MS system coupled with a DAD UV–Vis detector, the authors did not take fuller advantage of this instrumentation. There are several commercially available maltodextrin and isomaltose oligosaccharide standards that could have been used to develop and validate an accurate analytical method for the proper quantification of both glucose consumption and oligosaccharide production. The following references provide useful examples that could have guided the authors in designing a suitable analytical method for quantifying oligomers derived from glucose: Carbohydrate Research 257 (1994) 249–268; Journal of Chromatography 207 (1981) 439–444; Chromatographia 50 (1999) 705–710; Z. Lebensm. Unters. Forsch. A 206 (1998) 175–178; and http://dx.doi.org/10.2139/ssrn.5413053. Since the stereochemistry of the oligomers is not a central aspect of this study, which focuses primarily on the production and chain length of the oligosaccharides, the use of external standards would have enabled the construction of calibration curves and an accurate quantification of the oligomers formed in the presence of each metal cation. This methodology would allow a more meaningful comparison between experiments and support more robust overall conclusions.
2) The quantification of glucose consumption by ^{1}H NMR is a rather complex methodology, which is described in detail only in the previous OLEB 2019. In that paper, the quantification of glucose conversion was clearly explained, and the NMR spectra were carefully interpreted, with comprehensive assignments of both hydrogen and carbon atoms based on one- and two-dimensional experiments. However, in the present manuscript, the NMR results are only superficially described. Rough assignments of ^{1}H and ^{13}C signals are provided, and the approach used for quantifying glucose is not clearly defined. Although numerous NMR spectra are presented, the information extracted from them is not clearly shown and the results are not fully conclusive. For clarity and conciseness, the large number of NMR spectra (Figures S50–S89) could be grouped into four summary figures corresponding to the four experimental series: (i) fresh samples

– acidic conditions; (ii) fresh samples – basic conditions; (iii) Dry-down samples – acidic conditions; and (iv) Dry-down samples – basic conditions. Each figure could display nine representative spectra, following the layout used in the OLEB 2019 paper (for instance, Figures S31c, S32a, and S32c).

3) The ESI–MS data appear to have been treated somewhat carelessly, which may lead to inaccurate or misleading conclusions. Please unify the m/z scale (from 50 to 1500) across all mass spectra (Figures S19–S54) to allow consistent comparison. In addition, the mass peak labelling should be carefully revised and correctly positioned. For example, in Figure S25a, a peak around 600 amu is assigned to 7Glu + Ca, whereas this species should have a molecular mass of approximately 1193 amu. Similar inconsistencies are observed in Figure S27a. Although I have not examined every spectrum in detail, the authors are strongly encouraged to perform a thorough revision of all the mass spectra. Furthermore, it is unclear why nonamers are identified by LC–MS but not by ESI–MS. The authors should clarify this discrepancy and specify the limits of detection for both techniques.
4) I believe that the assignment of the oligomers by LC–MS was based on the SIM mass spectra; however, why do the species from the tetramer to the nonamer appear at the same retention time (Fig. 5a and Figs. S117–S125)? In line with the above comments, why were commercial standards not used for the calibration of the LC–MS analysis? On the other hand, in any case the conversion of glucose seems to be incomplete. The absence of a relative intensity scale in Figs. S118–S125 makes it difficult to interpret the data properly. What is the final concentration of glucose with respect to its oligomers? Moreover, using a logarithmic scale in Figs. 5b–5e would allow a clearer visualisation of the semiquantitative analysis of the different oligomers shown in the same graphs, especially if points rather than bars were used. In fact, these four figures could be conveniently summarised in a single one by employing a logarithmic scale.
5) The ATR–FTIR spectra are presented in a rather inconsistent manner throughout the manuscript. The quality of the recorded spectra should be improved, and the number of figures reduced, as suggested above for the 1H NMR data. After carefully examining all the FTIR spectra, I was unable to identify any discernible features around 1050 cm^{-1}. Likewise, in Figure 2a of the main text, the assignment of the FTIR bands is neither clearly explained nor properly marked. The bands are neither indicated on the spectra nor adequately discussed. The authors are therefore encouraged to enhance both the quality and the clarity of the FTIR data, ensuring that all relevant spectral bands are correctly identified and assigned. Alternatively, if the FTIR results do not provide significant or complementary information regarding the formation of oligomers—particularly when compared with the data obtained from NMR (if they would properly described) and ESI–MS—this section could reasonably be omitted from the manuscript.
6) What is the initial pH of each experiment? This is a very important point. The oligomerization of glucose is an acid-catalyzed process, as previously demonstrated and indicated in the present manuscript. A low pH favors the formation of oligomers. However, not all the salts used are neutral, and therefore the initial pH in each of the two experimental series (HCl and NaOH) may differ for the various salts. For a better interpretation of the results, Figure S55 and the associated discussion should consider the increase in pH rather than the absolute final pH value of each experiment. Thus, please indicate the initial and final pH values for each experiment, as well as the corresponding pH increase.

Minor comments

1) The manuscript could be rewritten to help the reader better appreciate the significance of the results. There is a large amount of data, but even for a specialised reader it is not easy to extract its full value. I believe that the Results and Discussion section should be reorganised. For example, the results obtained by each technique could be presented separately (technique by technique), followed by a comparison of the information provided by each of them, a discussion of the internal consistency of all the data, and finally a comparison with the only previous study on glucose polymerisation. Finally, a especific section could then address the mechanistic aspects of oligomerisation (coordination with metal cations, influence of pH, pH variations during the reactions and so on).
2) I have noticed some differences in the glucose conversion values obtained in the control experiments without salts, compared with those reported in the previous paper. Please place these differences in context within the discussion.
3) What is the reason for the change of solvent used to record the NMR spectra (DMSO for the $FeCl_2$ and $MgCl_2$ experiments)?
4) The introduction is well referenced; however, it should be reorganised. For example, on page 2: "While abiotic polymerisation of peptides and nucleic acid polymers has been studied extensively,21–36 abiotic oligosaccharide polymerisation was reported only once, in drying reactions of glucose.37"and on page 3: "In the context of prebiotic chemistry, metal-catalysed peptide and RNA bond formation has been demonstrated.21,51–53,56–65 […] While it is now known that oligosaccharides can form abiotically,37 it is unknown whether metal ions have any effect on the polymerisation of sugars." These ideas should be unified for better coherence. Additionally, in the introduction, the following paragraph seems more appropriate for the Conclusions section: "We found that while metal ions typically inhibited the overall conversion of glucose, some promoted the formation of longer oligosaccharides. These include Fe^{2+}, K^{+}, Mg^{2+}, and Li^{+}. For example, while glucose conversion was ~67% in the presence of Fe^{2+}, compared to ~86% in its absence, it led to a significant increase in the abundance of octamers (by ~1.7-fold) and nonamers (by ~2.3-fold). The Ca^{2+} ion, on the other hand, inhibited the formation of longer oligomers."
