# Supplementary material for: Metal ions affect abiotic sugar oligomerization reactions
Source: Commun Chem. 2026 Jul 13;9:345. doi: 10.1038/s42004-026-02129-w (PMC13737047; doi:10.1038/s42004-026-02129-w)
Supplement: Supplementary file 2 [file 42004_2026_2129_MOESM2_ESM.pdf]

Supplementary Information

Metal Ions Affect Prebiotic Sugar Oligomerization Reactions

Daniil Akulenko[a], Rotem Edri[a], and Moran Frenkel-Pinter[a,b,c*]

[a] Institute of Chemistry, The Hebrew University of Jerusalem, Jerusalem, Israel 9190401
[b] The Center for Nanoscience and Nanotechnology, The Hebrew University of Jerusalem, Jerusalem, Israel 9190401
[c] Casali Center of Applied Chemistry, The Hebrew University of Jerusalem, Jerusalem 9190401, Israel

*Corresponding author:

Dr. Moran Frenkel-Pinter
The Center for Nanoscience and Nanotechnology
Institute of Chemistry
The Hebrew University of Jerusalem
Edmond J. Safra Campus
Jerusalem 9190401, Israel
Ph: (+972)-2-6584171
moran.fp@mail.huji.ac.il

Table of Contents

Figure S1. Comparison of relative distributions of D-Glucose oligomerization products based on SIM-LC-MS. 11

Figure S2. FTIR spectra upon single-step dry-down reactions of glucose (glu) and LiCl. 12

Figure S3. FTIR spectra upon single-step dry-down reactions of glucose (glu) and $ZnCl_2$. 12

Figure S4. FTIR spectra upon single-step dry-down reactions of glucose (glu) and NaCl. 13

Figure S5. FTIR spectra upon single-step dry-down reactions of glucose (glu) and KCl. 13

Figure S6. FTIR spectra upon single-step dry-down reactions of glucose (glu) and $MgCl_2$. 14

Figure S7. FTIR spectra upon single-step dry-down reactions of glucose (glu) and $CaCl_2$. 14

Figure S8. FTIR spectra upon single-step dry-down reactions of glucose (glu) and $MnCl_2$. 15

Figure S9. FTIR spectra upon single-step dry-down reactions of glucose (glu) and $FeCl_2$. 15

Figure S10. FTIR spectra upon single-step dry-down reactions of glucose (glu) and $AlCl_3$. 16

Figure S11. FTIR spectra upon single-step dry-down reactions of glucose (glu) and LiCl. 16

Figure S12. FTIR spectra upon single-step dry-down reactions of glucose (glu) and $ZnCl_2$. 17

Figure S13. FTIR spectra upon single-step dry-down reactions of glucose (glu) and NaCl. 17

Figure S14. FTIR spectra upon single-step dry-down reactions of glucose (glu) and KCl. 18

Figure S15. FTIR spectra upon single-step dry-down reactions of glucose (glu) and $MgCl_2$. 18

Figure S16. FTIR spectra upon single-step dry-down reactions of glucose (glu) and $CaCl_2$. 19

Figure S17. FTIR spectra upon single-step dry-down reactions of glucose (glu) and $MnCl_2$. 19

Figure S18. FTIR spectra upon single-step dry-down reactions of glucose (glu) and $FeCl_2$. 20

Figure S19. FTIR spectra upon single-step dry-down reactions of glucose (glu) and $AlCl_3$. 20

Figure S20. FTIR signals of glucose oligomer standards. 21

Figure S21. ESI-MS of a dry-down reaction of glucose (glu) in an acidified environment 22

Figure S22. ESI-MS of a dry-down reaction of glucose (glu) and LiCl in an acidified environment 23

Figure S23. ESI-MS of a dry-down reaction of glucose (glu) and $ZnCl_2$ in an acidified environment 24

Figure S24. ESI-MS of a dry-down reaction of glucose (glu) and NaCl in an acidified environment 25

Figure S25. ESI-MS of a dry-down reaction of glucose (glu) and KCl in an acidified environment 26

Figure S26. ESI-MS of a dry-down reaction of glucose (glu) and $MgCl_2$ in an acidified environment 27

Figure S27. ESI-MS of a dry-down reaction of glucose (glu) and $CaCl_2$ in an acidified environment 28

Figure S28. ESI-MS of a dry-down reaction of glucose (glu) and $MnCl_2$ in an acidified environment 29

Figure S29. ESI-MS of a dry-down reaction of glucose (glu) and $FeCl_2$ in an acidified environment 30
Figure S30. ESI-MS of a dry-down reaction of glucose (glu) and $AlCl_3$ in an acidified environment 31
Figure S31. ESI-MS of a dry-down reaction of glucose (glu) upon addition of NaOH 32
Figure S32. ESI-MS of a dry-down reaction of glucose (glu) and LiCl upon addition of NaOH 33
Figure S33. ESI-MS of a dry-down reaction of glucose (glu) and $ZnCl_2$ upon addition of NaOH 34
Figure S34. ESI-MS of a dry-down reaction of glucose (glu) and NaCl upon addition of NaOH 35
Figure S35. ESI-MS of a dry-down reaction of glucose (glu) and KCl upon addition of NaOH . 36
Figure S36. ESI-MS of a dry-down reaction of glucose (glu) and $MgCl_2$ upon addition of NaOH 37
Figure S37. ESI-MS of a dry-down reaction of glucose (glu) and $CaCl_2$ upon addition of NaOH 38
Figure S38. ESI-MS of a dry-down reaction of glucose (glu) and $MnCl_2$ upon addition of NaOH 39
Figure S39. ESI-MS of a dry-down reaction of glucose (glu) and $FeCl_2$ upon addition of NaOH 40
Figure S40. ESI-MS of a dry-down reaction of glucose (glu) and $AlCl_3$ upon addition of NaOH 41
Figure S41. ESI-MS of a fresh solution of glucose (glu) in an acidified environment 42
Figure S42. ESI-MS of a fresh solution of glucose (glu) and LiCl in an acidified environment . 43
Figure S43. ESI-MS of a fresh solution of glucose (glu) and $ZnCl_2$ in an acidified environment 44
Figure S44. ESI-MS of a fresh solution of glucose (glu) and NaCl in an aacidified environment 45
Figure S45. ESI-MS of a fresh solution of glucose (glu) and KCl in an acidified environment.. 46
Figure S46. ESI-MS of a fresh solution of glucose (glu) and $MgCl_2$ in an acidified environment 47
Figure S47. ESI-MS of a fresh solution of glucose (glu) and $CaCl_2$ in an acidified environment 48
Figure S48. ESI-MS of a fresh solution of glucose (glu) and $MnCl_2$ in an acidified environment 49
Figure S49. ESI-MS of a fresh solution of glucose (glu) and $FeCl_2$ in an acidified environment 50
Figure S50. ESI-MS of a fresh solution of glucose (glu) and $AlCl_3$ in an acidified environment51
Figure S51. ESI-MS of a fresh solution of glucose (glu) upon addition of NaOH 52
Figure S52. ESI-MS of a fresh solution of glucose (glu) and LiCl upon addition of NaOH 53

Figure S53. ESI-MS of a fresh solution of glucose (glu) and $ZnCl_2$ upon addition of NaOH......54
Figure S54. ESI-MS of a fresh solution of glucose (glu) and NaCl upon addition of NaOH 55
Figure S55. ESI-MS of a fresh solution of glucose (glu) and KCl upon addition of NaOH.........56
Figure S56. ESI-MS of a fresh solution of glucose (glu) and $MgCl_2$ upon addition of NaOH 57
Figure S57. ESI-MS of a fresh solution of glucose (glu) and $CaCl_2$ upon addition of NaOH 58
Figure S58. ESI-MS of a fresh solution of glucose (glu) and $MnCl_2$ upon addition of NaOH 59
Figure S59. ESI-MS of a fresh solution of glucose (glu) and $FeCl_2$ upon addition of NaOH......60
Figure S60. ESI-MS of a fresh solution of glucose (glu) and $AlCl_3$ upon addition of NaOH 61
Figure S61. A plot summarizing the conversion of D-Glucose under acidified conditions (red) and upon addition of NaOH (blue) versus final pH in the samples. Integrations were based on ^{1}H-NMR resonances of the anomeric protons before and after the dry-down reactions (see Materials and Methods). 62
Figure S62. ^{1}H-NMR spectra overlay of dry-down reaction of glucose in the absence (blue) or presence (red) of LiCl under acidified environment......63
Figure S63. ^{1}H-NMR spectra overlay of dry-down reaction of glucose in the absence (blue) or presence (red) of $ZnCl_2$ under acidified environment......64
Figure S64. ^{1}H-NMR spectra overlay of dry-down reaction of glucose in the absence (blue) or presence (red) of NaCl under acidified environment......65
Figure S65. ^{1}H-NMR spectra overlay of dry-down reaction of glucose in the absence (blue) or presence (red) of KCl under acidified environment 66
Figure S66. ^{1}H-NMR spectra overlay of dry-down reaction of glucose in the absence (blue) or presence (red) of $MgCl_2$ under acidified environment......67
Figure S67. ^{1}H-NMR spectra overlay of dry-down reaction of glucose in the absence (blue) or presence (red) of $CaCl_2$ under acidified environment......68
Figure S68. ^{1}H-NMR spectra stack of dry-down reaction of glucose in the absence (blue) or presence (red) of $MnCl_2$ under acidified environment. 69
Figure S69. ^{1}H-NMR spectra stack of dry-down reaction of glucose in the absence (blue) or presence (red) of $FeCl_2$ under acidified environment 70
Figure S70. ^{1}H-NMR spectra overlay of dry-down reaction of glucose in the absence (blue) or presence (red) of $AlCl_3$ under acidified environment......71
Figure S71. ^{1}H-NMR spectra overlay of dry-down reaction of glucose in the absence (blue) or presence (red) of LiCl upon addition of NaOH 72
Figure S72. ^{1}H-NMR spectra overlay of dry-down reaction of glucose in the absence (blue) or presence (red) of $ZnCl_2$ upon addition of NaOH......73
Figure S73. ^{1}H-NMR spectra overlay of dry-down reaction of glucose in the absence (blue) or presence (red) of NaCl upon addition of NaOH 74
Figure S74. ^{1}H-NMR spectra overlay of dry-down reaction of glucose in the absence (blue) or presence (red) of KCl upon addition of NaOH......75
Figure S75. ^{1}H-NMR spectra overlay of dry-down reaction of glucose in the absence (blue) or presence (red) of $MgCl_2$ upon addition of NaOH 76

Figure S76. ^{1}H-NMR spectra overlay of dry-down reaction of glucose in the absence (blue) or presence (red) of $CaCl_2$ upon addition of NaOH..77
Figure S77. ^{1}H-NMR spectra stack of dry-down reaction of glucose in the absence (blue) or presence (red) of $MnCl_2$ upon addition of NaOH 78
Figure S78. ^{1}H-NMR spectra stack of dry-down reaction of glucose in the absence (blue) or presence (red) of $FeCl_2$ upon addition of NaOH...79
Figure S79. ^{1}H-NMR spectra overlay of dry-down reaction of glucose in the absence (blue) or presence (red) of $AlCl_3$ upon addition of NaOH 80
Figure S80. Overlay of ^{1}H-NMR spectra of a fresh (blue) or a dry-down reaction at 50˚C for seven days under acidified conditions (red) of glucose in the absence of metals...................81
Figure S81.Overlay of ^{1}H-NMR spectra of a fresh (blue) or a dry-down reaction at 50˚C for seven days under acidified conditions (red) of glucose in the presence of LiCl 82
Figure S82. Overlay of ^{1}H-NMR spectra of a fresh (blue) or a dry-down reaction at 50˚C for seven days under acidified conditions (red) of glucose in the presence of $ZnCl_2$....................83
Figure S83. Overlay of ^{1}H-NMR spectra of a fresh (blue) or a dry-down reaction at 50˚C for seven days under acidified conditions (red) of glucose in the presence of NaCl.....................84
Figure S84. Overlay of ^{1}H-NMR spectra of a fresh (blue) or a dry-down reaction at 50˚C for seven days under acidified conditions (red) of glucose in the presence of KCl 85
Figure S85. Overlay of ^{1}H-NMR spectra of a fresh (blue) or a dry-down reaction at 50˚C for seven days under acidified conditions (red) of glucose in the presence of $MgCl_2$ 86
Figure S86. Overlay of ^{1}H-NMR spectra of a fresh (blue) or a dry-down reaction at 50˚C for seven days under acidified conditions (red) of glucose in the presence of $CaCl_2$....................87
Figure S87. Overlay of ^{1}H-NMR spectra of a fresh (blue) or a dry-down reaction at 50˚C for seven days under acidified conditions (red) of glucose in the presence of $MnCl_2$ 88
Figure S88. Overlay of ^{1}H-NMR spectra of a fresh (blue) or a dry-down reaction at 50˚C for seven days under acidified conditions (red) of glucose in the presence of $FeCl_2$....................89
Figure S89. Overlay of ^{1}H-NMR spectra of a fresh (blue) or a dry-down reaction at 50˚C for seven days under acidified conditions (red) of glucose in the presence of $AlCl_3$.....................90
Figure S90. Overlay of ^{1}H-NMR spectra of a fresh (blue) or a dry-down reaction at 50˚C for seven days upon addition of NaOH (red) of glucose in the absence of metals 91
Figure S91. Overlay of ^{1}H-NMR spectra of a fresh (blue) or a dry-down reaction at 50˚C for seven days upon addition of NaOH (red) of glucose in the presence of LiCl 92
Figure S92. Overlay of ^{1}H-NMR spectra of a fresh (blue) or a dry-down reaction at 50˚C for seven days upon addition of NaOH (red) of glucose in the presence of $ZnCl_2$ 93
Figure S93. Overlay of ^{1}H-NMR spectra of a fresh (blue) or a dry-down reaction at 50˚C for seven days upon addition of NaOH (red) of glucose in the presence of NaCl 94
Figure S94. Overlay of ^{1}H-NMR spectra of a fresh (blue) or a dry-down reaction at 50˚C for seven days upon addition of NaOH (red) of glucose in the presence of KCl............................95
Figure S95. Overlay of ^{1}H-NMR spectra of a fresh (blue) or a dry-down reaction at 50˚C for seven days upon addition of NaOH (red) of glucose in the presence of $MgCl_2$.......................96

Figure S96. Overlay of ^{1}H-NMR spectra of a fresh (blue) or a dry-down reaction at 50˚C for seven days upon addition of NaOH (red) of glucose in the presence of $CaCl_2$ 97

Figure S97. Overlay of ^{1}H-NMR spectra of a fresh (blue) or a dry-down reaction at 50˚C for seven days upon addition of NaOH (red) of glucose in the presence of $MnCl_2$.......................98

Figure S98. Overlay of ^{1}H-NMR spectra of a fresh (blue) or a dry-down reaction at 50˚C for seven days upon addition of NaOH (red) of glucose in the presence of $FeCl_2$.........................99

Figure S99. Overlay of ^{1}H-NMR spectra of a fresh (blue) or a dry-down reaction at 50˚C for seven days upon addition of NaOH (red) of glucose in the presence of $AlCl_3$ 100

Figure S100. ^{1}H-NMR spectra of three independent dry-down reactions of glucose in the absence of metal ions under acidified conditions...101

Figure S101. ^{1}H-NMR spectra of three independent dry-down reactions of glucose and LiCl under acidified conditions..102

Figure S102. ^{1}H-NMR spectra of three independent dry-down reactions of glucose and $ZnCl_2$ under acidified conditions..103

Figure S103. ^{1}H-NMR spectra of three independent dry-down reactions of glucose and NaCl under acidified conditions..104

Figure S104. ^{1}H-NMR spectra of three independent dry-down reactions of glucose and KCl under acidified conditions..105

Figure S105. ^{1}H-NMR spectra of three independent dry-down reactions of glucose and $MgCl_2$ under acidified conditions..106

Figure S106. ^{1}H-NMR spectra of three independent dry-down reactions of glucose and $CaCl_2$ under acidified conditions..107

Figure S107. ^{1}H-NMR spectra of three independent dry-down reactions of glucose and $MnCl_2$ under acidified conditions..108

Figure S108. ^{1}H-NMR spectra of three independent dry-down reactions of glucose and $FeCl_2$ under acidified conditions..109

Figure S109. ^{1}H-NMR spectra of three independent dry-down reactions of glucose and $AlCl_3$ under acidified conditions..110

Figure S110. ^{1}H-NMR spectra of three independent dry-down reactions of glucose in the absence of metal ions upon addition of NaOH 111

Figure S111. ^{1}H-NMR spectra of three independent dry-down reactions of glucose and LiCl upon addition of NaOH 112

Figure S112. ^{1}H-NMR spectra of three independent dry-down reactions of glucose and $ZnCl_2$ upon addition of NaOH 113

Figure S113. ^{1}H-NMR spectra of three independent dry-down reactions of glucose and NaCl upon addition of NaOH 114

Figure S114. ^{1}H-NMR spectra of three independent dry-down reactions of glucose and KCl upon addition of NaOH 115

Figure S115. ^{1}H-NMR spectra of three independent dry-down reactions of glucose and $MgCl_2$ upon addition of NaOH 116

Figure S116. ^{1}H-NMR spectra of three independent dry-down reactions of glucose and $CaCl_2$ upon addition of NaOH 117

Figure S117. ^{1}H-NMR spectra of three independent dry-down reactions of glucose and $MnCl_2$ upon addition of NaOH 118

Figure S118. ^{1}H-NMR spectra of three independent dry-down reactions of glucose and $FeCl_2$ upon addition of NaOH 119

Figure S119. ^{1}H-NMR spectra of three independent dry-down reactions of glucose and $AlCl_3$ upon addition of NaOH 120

Figure S120. ^{1}H-NMR spectra of fresh glucose in the absence or presence of various metal ions under acidified conditions 121

Figure S121. ^{1}H-NMR spectra of fresh glucose in the absence or presence of various metal ions upon addition of NaOH.. 122

Figure S122. ^{1}H-NMR spectra of dry-down reaction of glucose in the absence or presence of various metal ions under acidified conditions 123

Figure S123. ^{1}H-NMR spectra of dry-down reaction of glucose in the absence or presence of various metal ions upon addition of NaOH. 124

Figure S124. Overlay of ^{1}H-NMR spectra of a cellobiose standard (green), a dry-down reaction of glucose at 50˚C for seven days under acidified conditions (red), and the dry-down reaction of glucose that was spiked with cellobiose (blue) 125

Figure S125. Overlay of ^{1}H-NMR spectra of an isomaltose standard (red), a dry-down reaction of glucose at 50˚C for seven days under acidified conditions (green), and the dry-down reaction of glucose that was spiked with isomaltose (blue)...................................... 126

Figure S126. Overlay of ^{1}H-NMR spectra of a maltotriose standard (red), a dry-down reaction of glucose at 50˚C for seven days under acidified conditions (green), and the dry-down reaction of glucose that was spiked with maltotriose (blue)..................................... 127

Figure S127. Overlay of ^{1}H-NMR spectra of an isomaltotriose standard (red), a dry-down reaction of glucose at 50˚C for seven days under acidified conditions (green), and the dry-down reaction of glucose that was spiked with isomaltotriose (blue) 128

Figure S128. Overlay of ^{1}H-NMR spectra of a cellobiose standard (red), a dry-down reaction of glucose at 50˚C for seven days under acidified conditions in the presence of $ZnCl_2$ (green), and the dry-down reaction of glucose that was spiked with cellobiose (blue)........ 129

Figure S129. Overlay of ^{1}H-NMR spectra of an isomaltose standard (red), a dry-down reaction of glucose at 50˚C for seven days under acidified conditions in the presence of $ZnCl_2$ (green), and the dry-down reaction of glucose that was spiked with isomaltose (blue)....... 130

Figure S130. Overlay of ^{1}H-NMR spectra of a maltotriose standard (red), a dry-down reaction of glucose at 50˚C for seven days under acidified conditions in the presence of $ZnCl_2$ (green), and the dry-down reaction of glucose that was spiked with maltotriose (blue) 131

Figure S131. Overlay of ^{1}H-NMR spectra of an isomaltotriose standard (red), a dry-down reaction of glucose at 50˚C for seven days under acidified conditions in the presence of $ZnCl_2$ (green), and the dry-down reaction of glucose that was spiked with isomaltotriose (blue). 132

Figure S132. Kinetics of fresh D-Glucose mutarotation in the presence of Ca^{2+} under acidified conditions 133

Figure S133. ^{13}C-NMR spectra of dry-down reactions of D-Glucose-^{13}C6 under acidified conditions. 134

Figure S134. ^{13}C-NMR spectra of dry-down reactions of D-Glucose-^{13}C6 upon addition of NaOH. 135

Figure S135. HSQC spectra of dry-down reaction of D-Glucose-^{13}C6 in the absence of metal ions under acidified conditions 136

Figure S136. HSQC spectra of dry-down reaction of D-Glucose-^{13}C6 in the presence of $ZnCl_2$ under acidified conditions 137

Figure S137. HSQC spectra of dry-down reaction of D-Glucose-^{13}C6 in the presence of $CaCl_2$ under acidified conditions 138

Figure S138. HSQC spectra of dry-down reaction of D-Glucose-^{13}C6 in the absence of metal ions upon addition of NaOH 139

Figure S139. HSQC spectra of dry-down reaction of D-Glucose-^{13}C6 in the presence of $ZnCl_2$ upon addition of NaOH 140

Figure S140. HSQC spectra of dry-down reaction of D-Glucose-^{13}C6 in the presence of $CaCl_2$ upon addition of NaOH 141

Figure S141. SIM-LC-MS of a dry down reaction of glucose (glu) under acidified conditions in the absence of metal ions at a 1mM final concentration 142

Figure S142. SIM-LC-MS of a dry down reaction of glucose (glu) under acidified conditions in the presence of LiCl at a 1mM final concentration 143

Figure S143. SIM-LC-MS of a dry down reaction of glucose (glu) under acidified conditions in the presence of $ZnCl_2$ at a 1mM final concentration 144

Figure S144. SIM-LC-MS of a dry down reaction of glucose (glu) under acidified conditions in the presence of NaCl at a 1mM final concentration 145

Figure S145. SIM-LC-MS of a dry down reaction of glucose (glu) under acidified conditions in the presence of KCl at a 1mM final concentration....... 146

Figure S146. SIM-LC-MS of a dry down reaction of glucose (glu) under acidified conditions in the presence of $MgCl_2$ at a 1mM final concentration 147

Figure S147. SIM-LC-MS of a dry down reaction of glucose (glu) under acidified conditions in the presence of $CaCl_2$ at a 1mM final concentration 148

Figure S148. SIM-LC-MS of a dry down reaction of glucose (glu) under acidified conditions in the presence of $MnCl_2$ at a 1mM final concentration 149

Figure S149. SIM-LC-MS of a dry down reaction of glucose (glu) under acidified conditions in the presence of $AlCl_3$ under anaerobic conditions at a 1mM final concentration 150

Figure S150. SIM-LC-MS of a dry down reaction of glucose (glu) under acidified conditions in the absence of metals under anaerobic conditions at a 1mM final concentration 151

Figure S151. SIM-LC-MS of a dry down reaction of glucose (glu) under acidified conditions in the presence of $FeCl_2$ at a 1mM final concentration....... 152

Figure S152. SIM-LC-MS of a dry down reaction of glucose (glu) under acidified conditions in the presence of $ZnCl_2$ under anaerobic conditions at a 1mM final concentration 153

Figure S153. SIM-LC-MS of a dry down reaction of glucose (glu) under acidified conditions in the absence of metals at a 10mM final concentration 154

Figure S154. SIM-LC-MS of a dry down reaction of glucose (glu) under acidified conditions in the presence of LiCl at a 10mM final concentration 155

Figure S155. SIM-LC-MS of a dry down reaction of glucose (glu) under acidified conditions in the presence of $ZnCl_2$ at a 10mM final concentration 156

Figure S156. SIM-LC-MS of a dry down reaction of glucose (glu) under acidified conditions in the presence of NaCl at a 10mM final concentration 157

Figure S157. SIM-LC-MS of a dry down reaction of glucose (glu) under acidified conditions in the presence of KCl at a 10mM final concentration...158

Figure S158. SIM-LC-MS of a dry down reaction of glucose (glu) under acidified conditions in the presence of $MgCl_2$ at a 10mM final concentration 159

Figure S159. SIM-LC-MS of a dry down reaction of glucose (glu) under acidified conditions in the presence of $CaCl_2$ at a 10mM final concentration 160

Figure S160. SIM-LC-MS of a dry down reaction of glucose (glu) under acidified conditions in the presence of $MnCl_2$ at a 10mM final concentration..161

Figure S161. SIM-LC-MS of a dry down reaction of glucose (glu) under acidified conditions in the presence of $AlCl_3$ at a 10mM final concentration 162

Figure S162. SIM-LC-MS of a dry down reaction of glucose (glu) under acidified conditions in the absence of metals under anaerobic conditions at a 10mM final concentration............163

Figure S163. SIM-LC-MS of a dry down reaction of glucose (glu) under acidified conditions in the presence of $FeCl_2$ under anaerobic conditions at a 10mM final concentration 164

Figure S164. SIM-LC-MS of a dry down reaction of glucose (glu) under acidified conditions in the presence of $ZnCl_2$ under anaerobic conditions at a 10mM final concentration 165

Figure S165. Relative distribution of D-Glucose oligomerization products based on SIM-LC-MS at 1 mM concentration (referring to glucose concentration prior to the drying reaction). 166

Figure S166. Absolute distribution of D-Glucose oligomerization products based on SIM-LC-MS at 1 mM concentration (referring to glucose concentration prior to the drying reaction). 167

Figure S167. Relative distribution of D-Glucose oligomerization products based on SIM-LC-MS under anaerobic conditions SIM at 1 mM concentration (referring to glucose concentration prior to the drying reaction). 168

Figure S168. Absolute distribution of D-Glucose oligomerization products based on SIM-LC-MS under anaerobic conditions SIM at 1 mM concentration (referring to glucose concentration prior to the drying reaction). 169

Figure S169. Relative distribution of D-Glucose oligomerization products based on SIM-LC-MS under aerobic conditions at 10 mM concentration (referring to glucose concentration prior to the drying reaction)...170

Figure S170. Absolute distribution of D-Glucose oligomerization products based on SIM-LC-MS under aerobic conditions at 10 mM concentration (referring to glucose concentration prior to the drying reaction)...171

Figure S171. Relative distribution of D-Glucose oligomerization products based on SIM-LC-MS under anaerobic conditions at 10 mM concentration (referring to glucose concentration prior to the drying reaction). 172

Figure S172. Absolute distribution of D-Glucose oligomerization products based on SIM-LC-MS under anaerobic conditions at 10 mM concentration (referring to glucose concentration prior to the drying reaction). 173

Figure S173. Cumulative distribution of D-Glucose oligomerization products based on SIM-SIM-LC-MS at 1 mM concentration (referring to glucose concentration prior to the drying reaction). 174

Figure S174. Cumulative distribution of D-Glucose oligomerization products based on SIM-LC-MS at 10 mM concentration (referring to glucose concentration prior to the drying reaction). 175

Figure S175. Cumulative distribution of D-Glucose oligomerization products based on SIM-LC-MS under anaerobic conditions at 1 mM concentration (referring to glucose concentration prior to the drying reaction). 176

Figure S176. Cumulative distribution of D-Glucose oligomerization products based on SIM-LC-MS under anaerobic conditions at 10 mM concentration (referring to glucose concentration prior to the drying reaction). 177

Figure S177. Calibration curves for glucose quantification by SIM-LC-MS. 178

Table S1. pH values for fresh and post-reaction mixtures. 179

Table S2. m/z values obtained by positive mode ESI-MS. Only detected species are shown. 179

Table S3. m/z values of detected glucose oligomer. Species correspond to either $[M-H]^-$, $[M-Cl]^-$ or $[M+HCOO]^-$. 180

Table S4. Statistical p-values for Figure 4 a-c based on three independent experiments. 180

Table S5. Statistical p-values for Figure 5 a-d based on three independent experiments. 181

Supplementary Figures

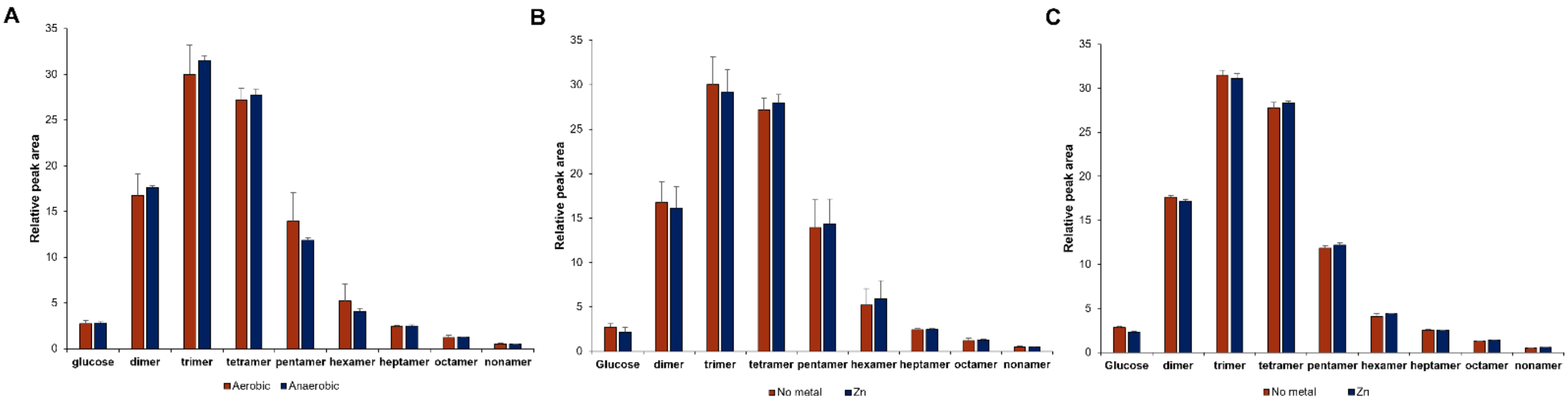

Figure S1. Comparison of relative distributions of D-Glucose oligomerization products based on SIM-LC-MS. Glucose product distribution after one week of dry down at 50 °C under acidified conditions. A comparison is shown for product distribution in the absence of metal ions under aerobic and anaerobic conditions (A), in the absence or presence of Zn^{2+} under aerobic conditions (B), and in the absence or presence of Zn^{2+} under anaerobic conditions (C).

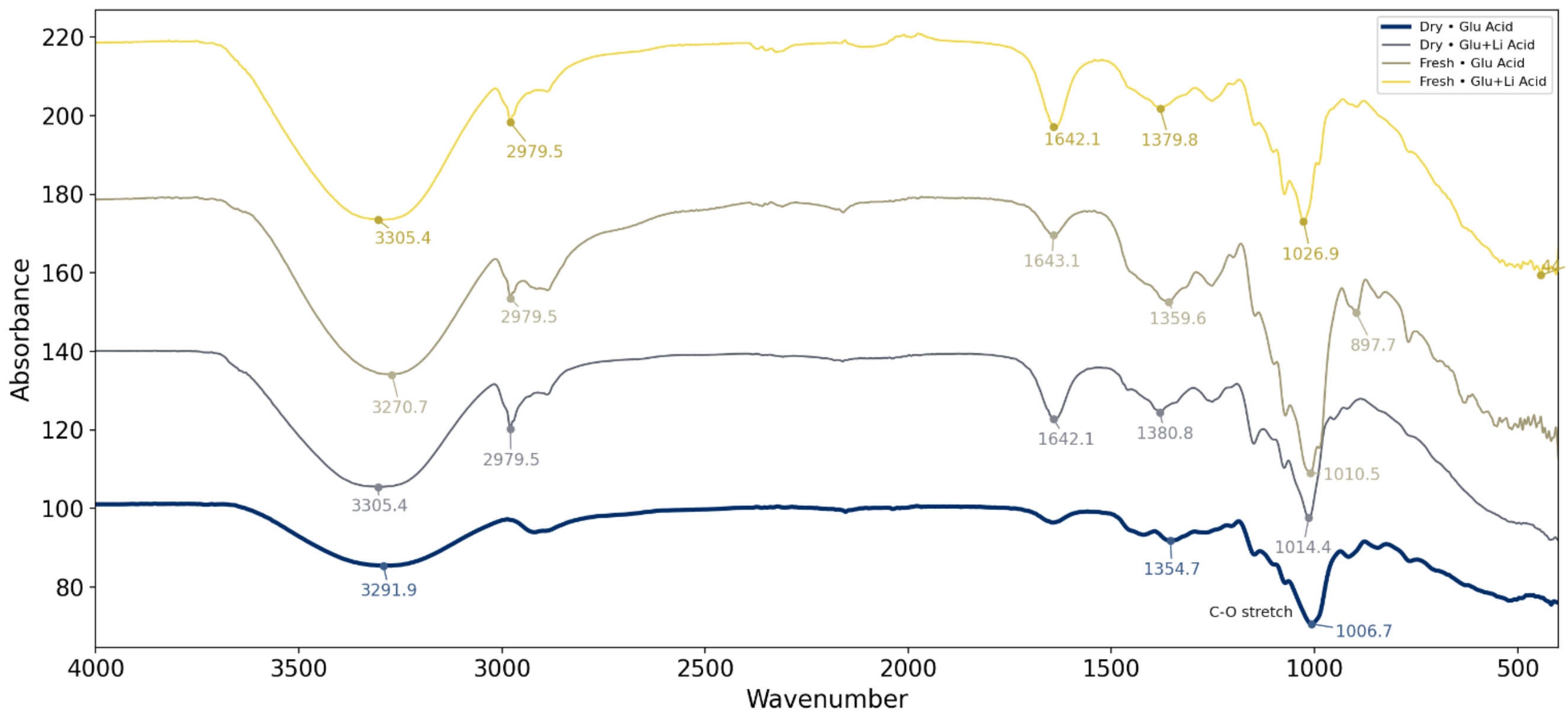

Figure S2. FTIR spectra upon single-step dry-down reactions of glucose (glu) and LiCl. Glu was dried in the absence or presence of LiCl at 50 ˚C for seven days under acidified conditions. Products were analyzed by FTIR and compared to unreacted stock solutions in the absence or presence of LiCl.

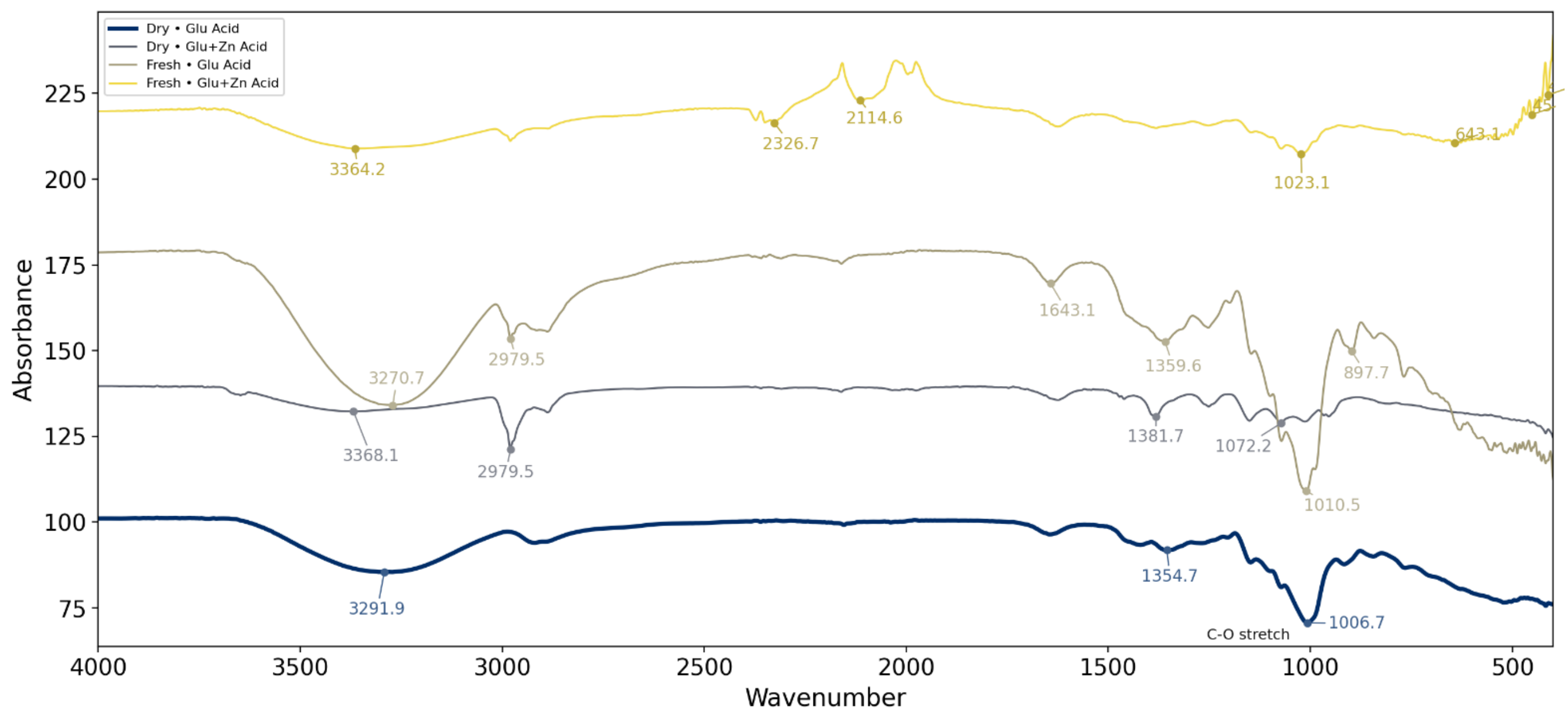

Figure S3. FTIR spectra upon single-step dry-down reactions of glucose (glu) and $ZnCl_2$. Glu was dried in the absence or presence of $ZnCl_2$ at 50 ˚C for seven days under acidified conditions. Products were analyzed by FTIR and compared to unreacted stock solutions in the absence or presence of $ZnCl_2$.

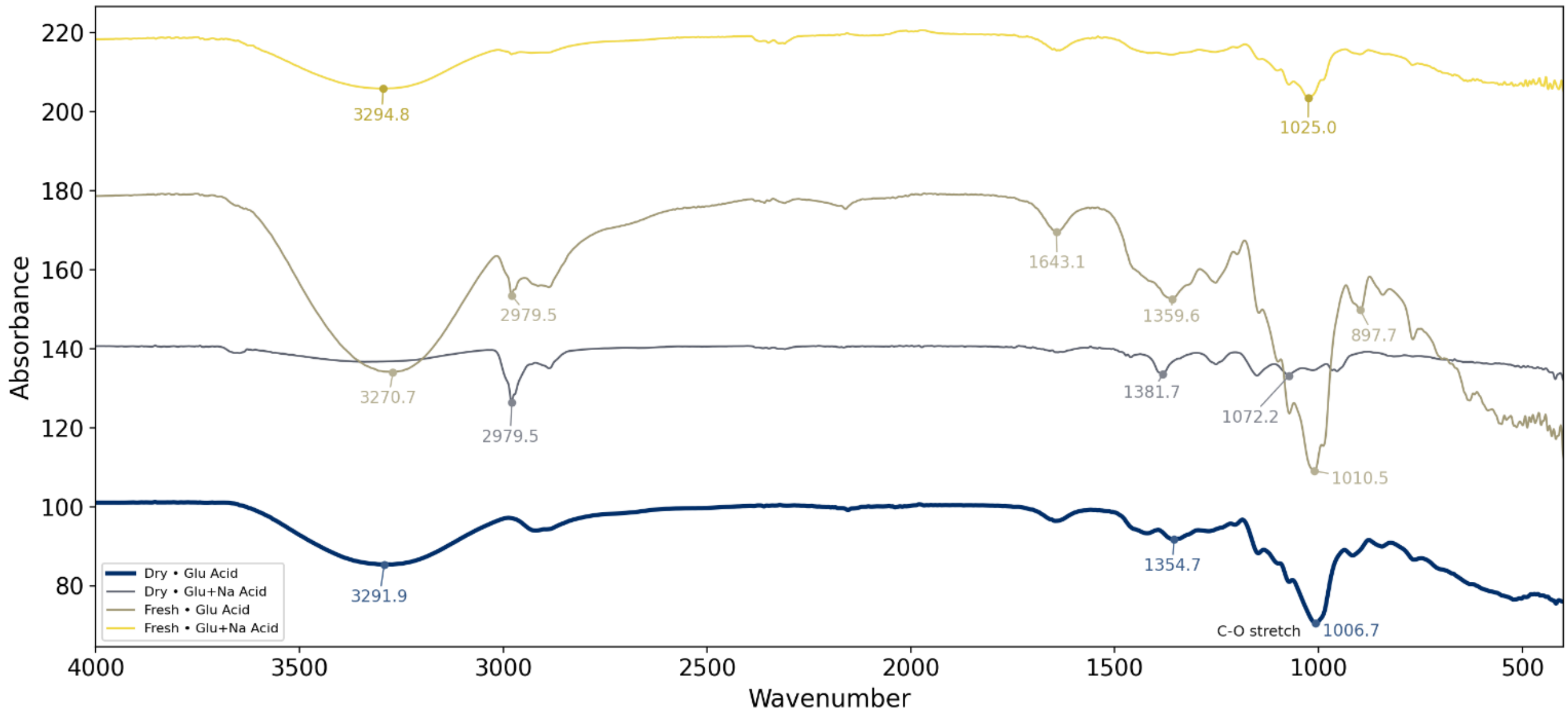

Figure S4. FTIR spectra upon single-step dry-down reactions of glucose (glu) and NaCl. Glu was dried in the absence or presence of NaCl at 50 ˚C for seven days under acidified conditions. Products were analyzed by FTIR and compared to unreacted stock solutions in the absence or presence of NaCl.

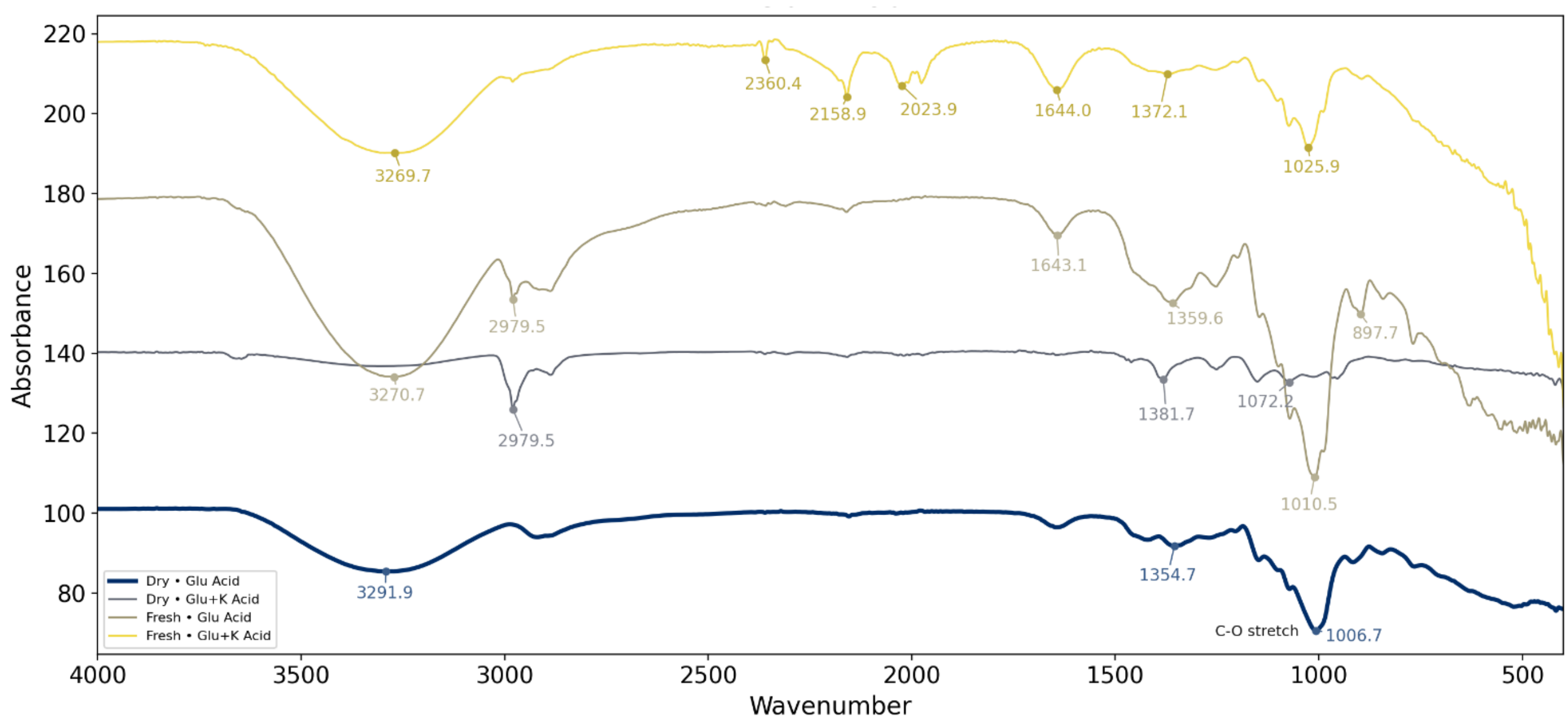

Figure S5. FTIR spectra upon single-step dry-down reactions of glucose (glu) and KCl. Glu was dried in the absence or presence of KCl at 50 ˚C for seven days under acidified conditions. Products were analyzed by FTIR and compared to unreacted stock solutions in the absence or presence of KCl.

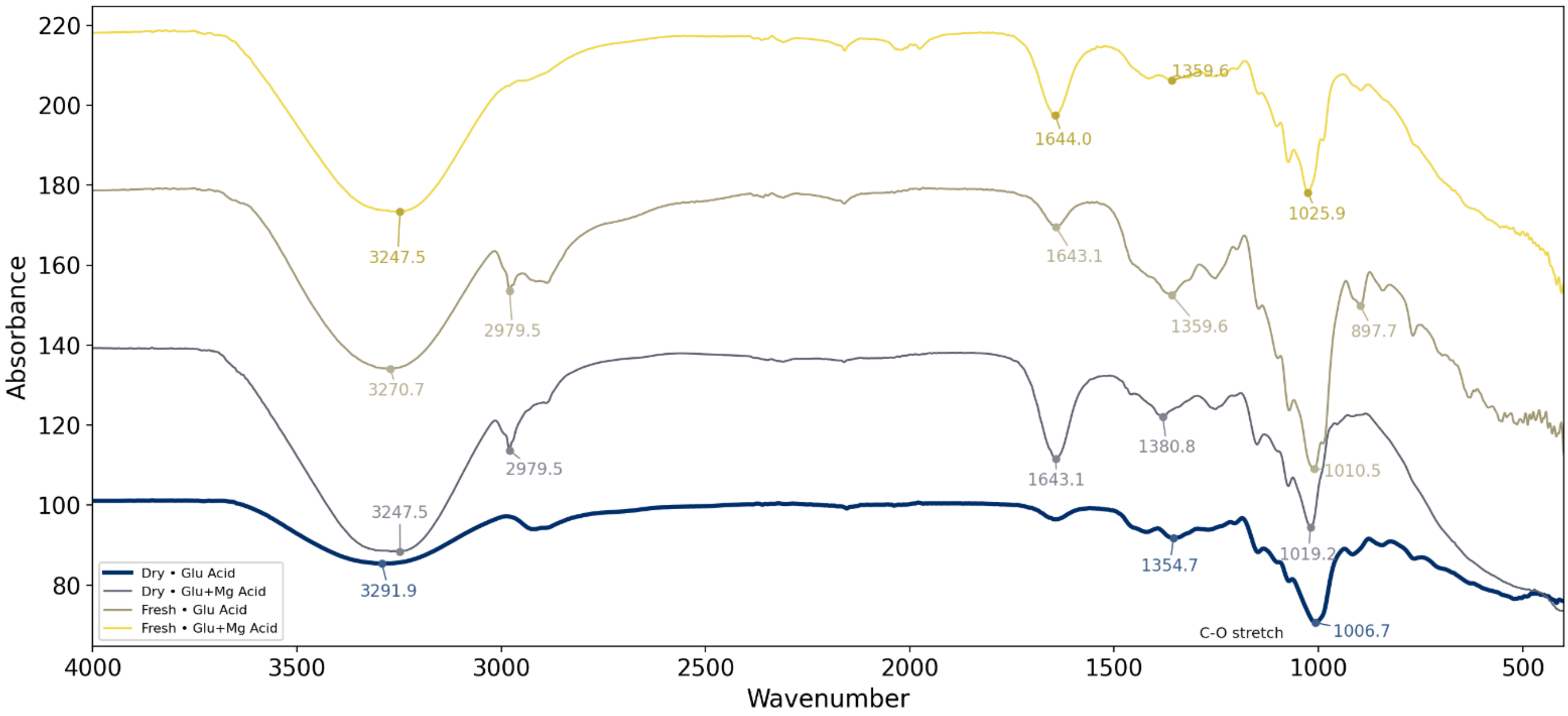

Figure S6. FTIR spectra upon single-step dry-down reactions of glucose (glu) and $MgCl_2$. Glu was dried in the absence or presence of $MgCl_2$ at 50 ˚C for seven days under acidified conditions. Products were analyzed by FTIR and compared to unreacted stock solutions in the absence or presence of $MgCl_2$.

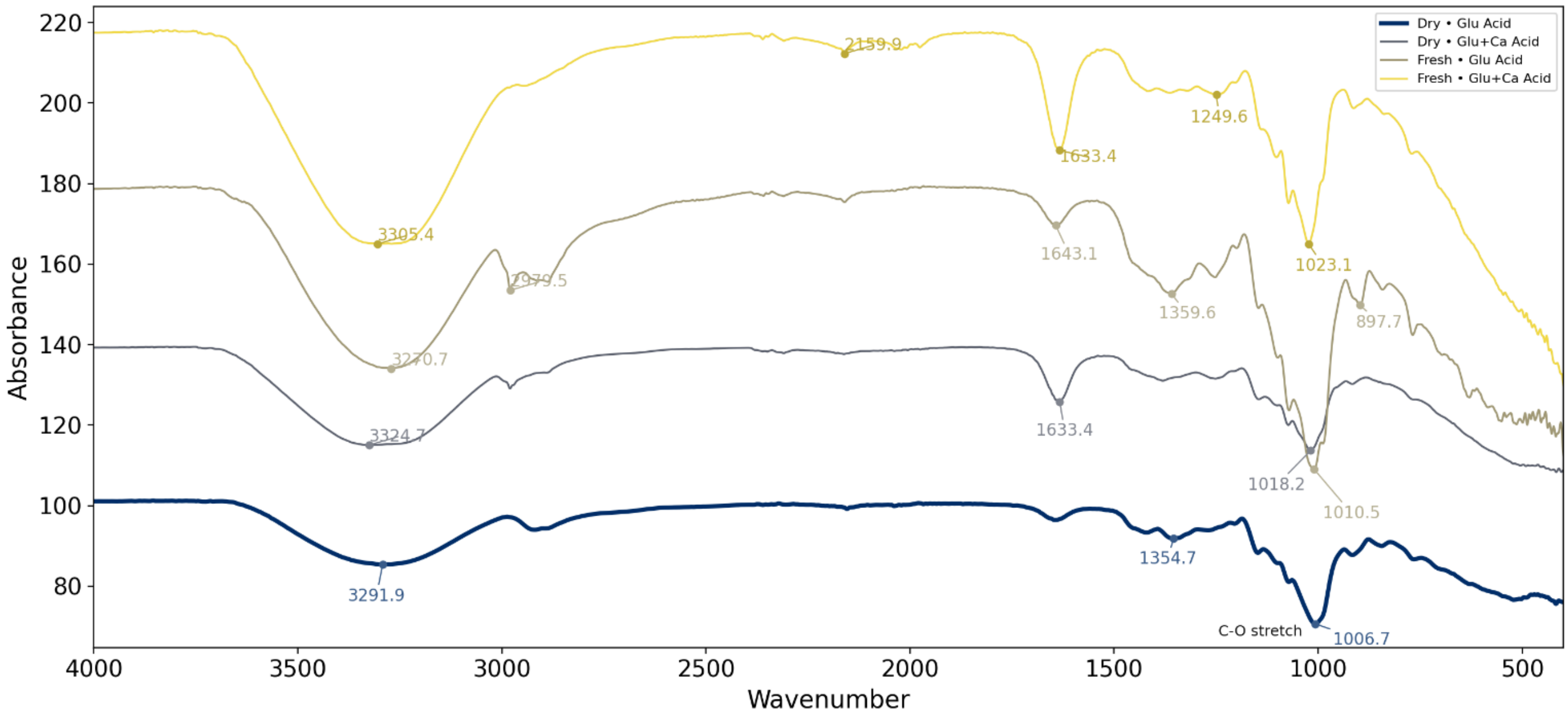

Figure S7. FTIR spectra upon single-step dry-down reactions of glucose (glu) and $CaCl_2$. Glu was dried in the absence or presence of $CaCl_2$ at 50 ˚C for seven days under acidified conditions. Products were analyzed by FTIR and compared to unreacted stock solutions in the absence or presence of $CaCl_2$.

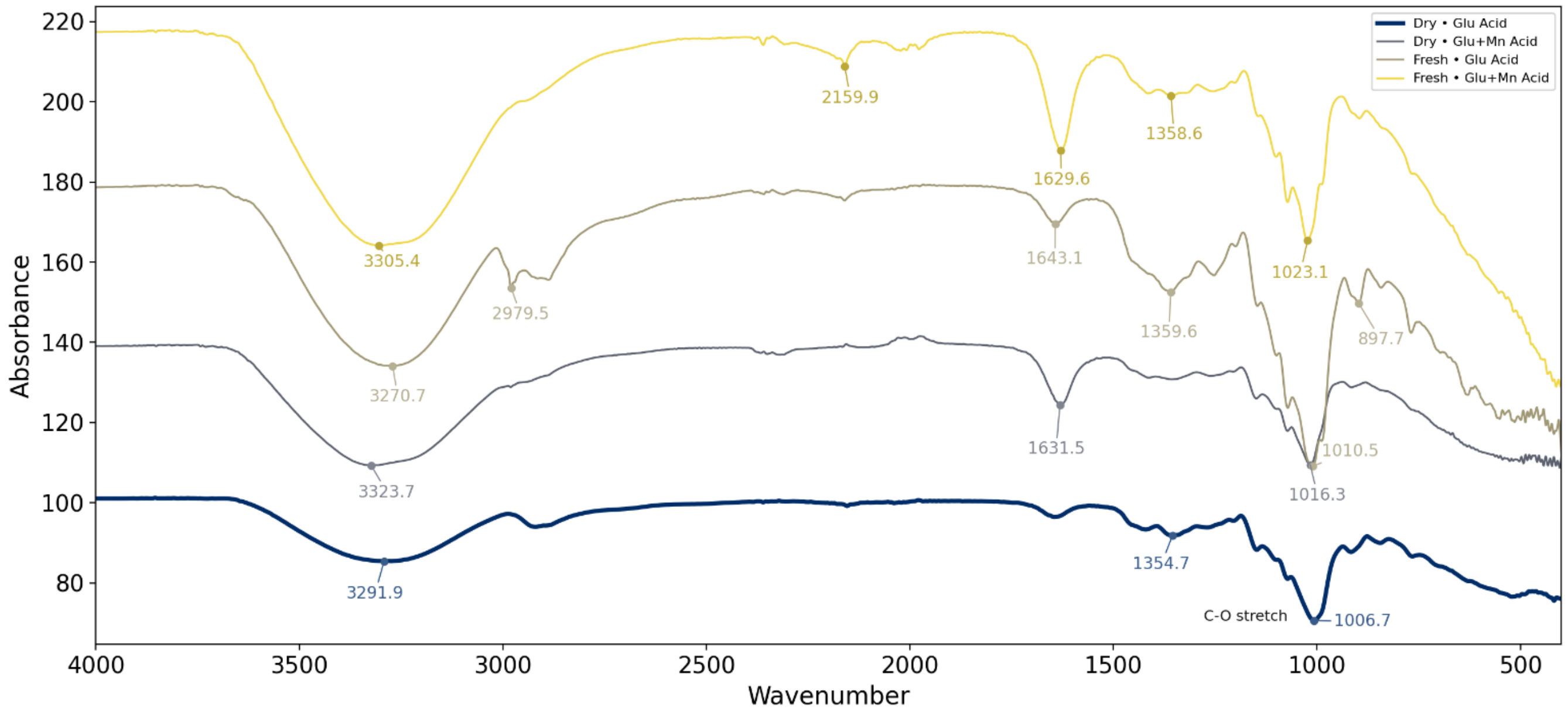

Figure S8. FTIR spectra upon single-step dry-down reactions of glucose (glu) and $MnCl_2$. Glu was dried in the absence or presence of $MnCl_2$ at 50 ˚C for seven days under acidified conditions. Products were analyzed by FTIR and compared to unreacted stock solutions in the absence or presence of $MnCl_2$.

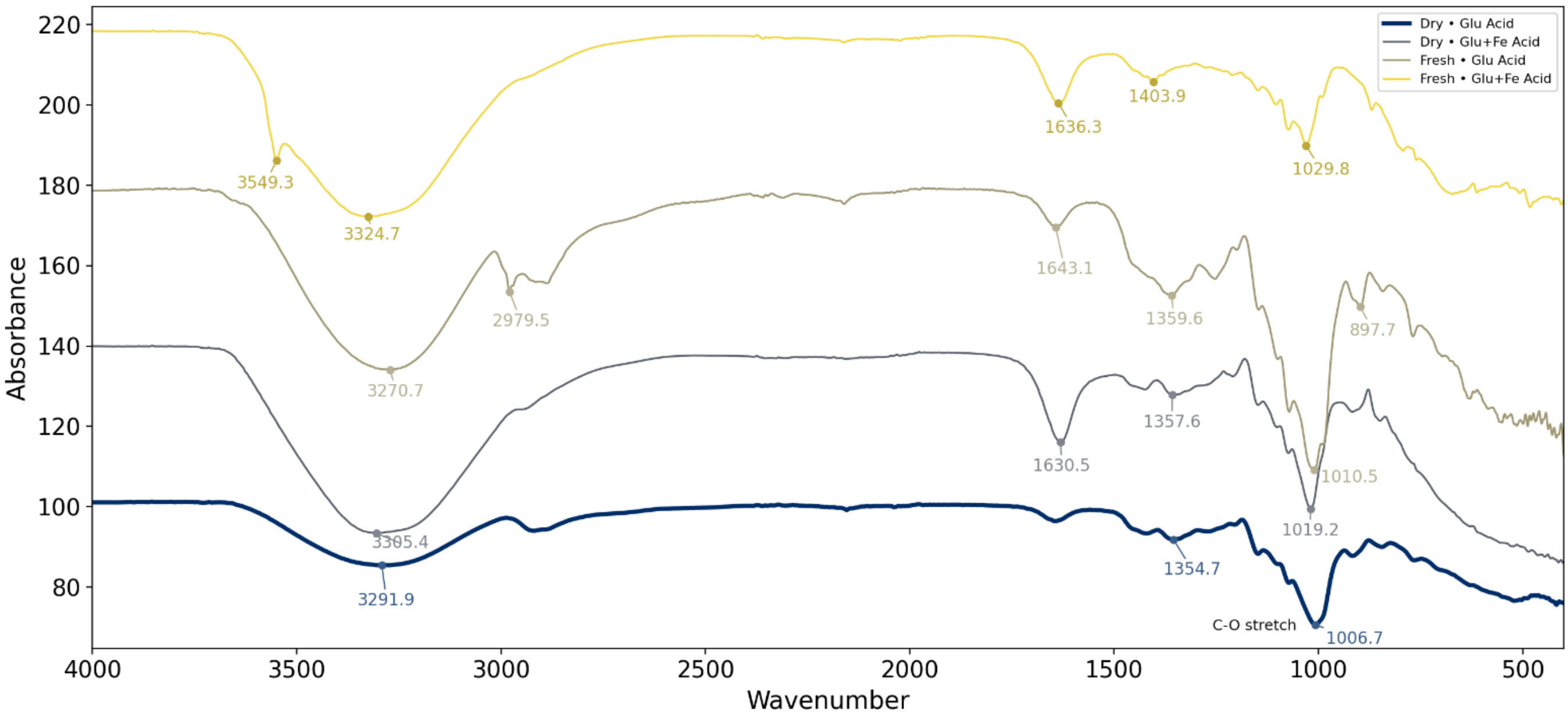

Figure S9. FTIR spectra upon single-step dry-down reactions of glucose (glu) and $FeCl_2$. Glu was dried in the absence or presence of $FeCl_2$ at 50 ˚C for seven days under acidified conditions. Products were analyzed by FTIR and compared to unreacted stock solutions in the absence or presence of $FeCl_2$.

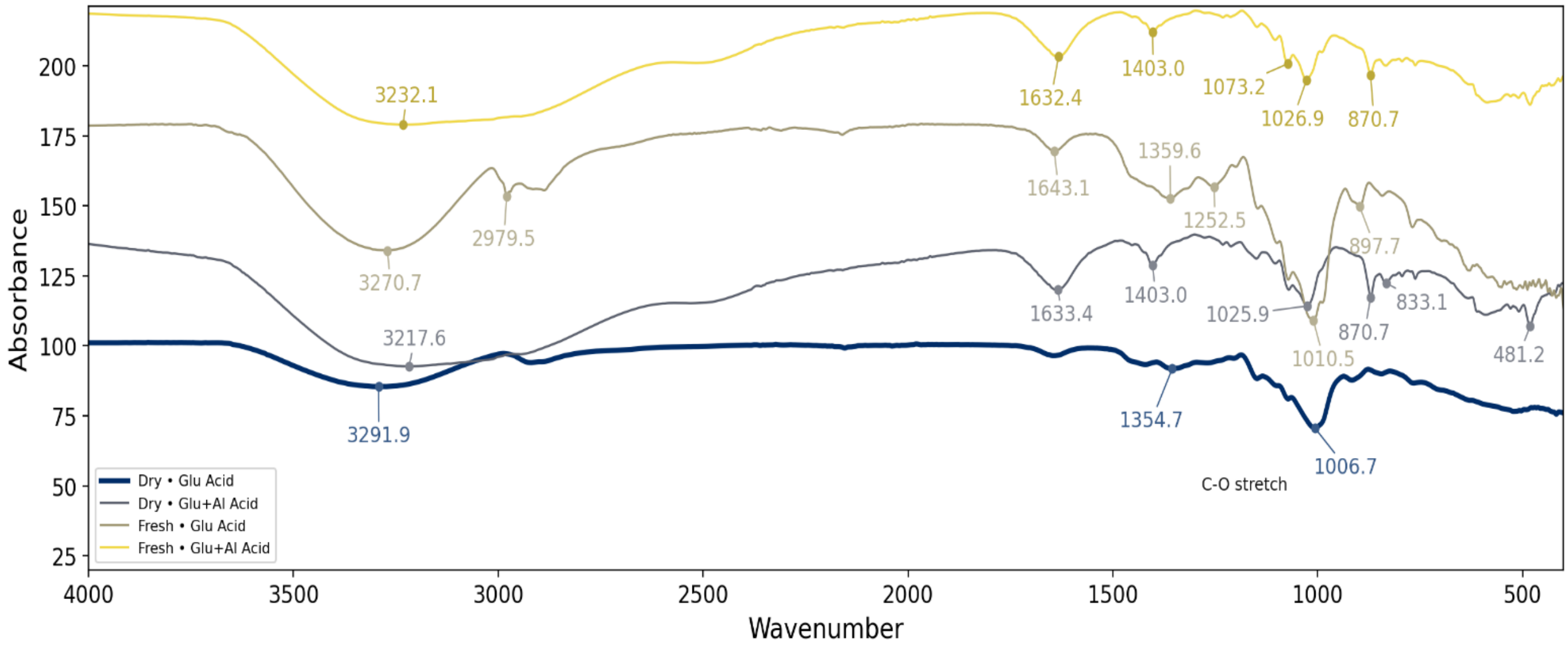

Figure S10. FTIR spectra upon single-step dry-down reactions of glucose (glu) and $AlCl_3$. Glu was dried in the absence or presence of $AlCl_3$ at 50 ˚C for seven days under acidified conditions. Products were analyzed by FTIR and compared to unreacted stock solutions in the absence or presence of $AlCl_3$.

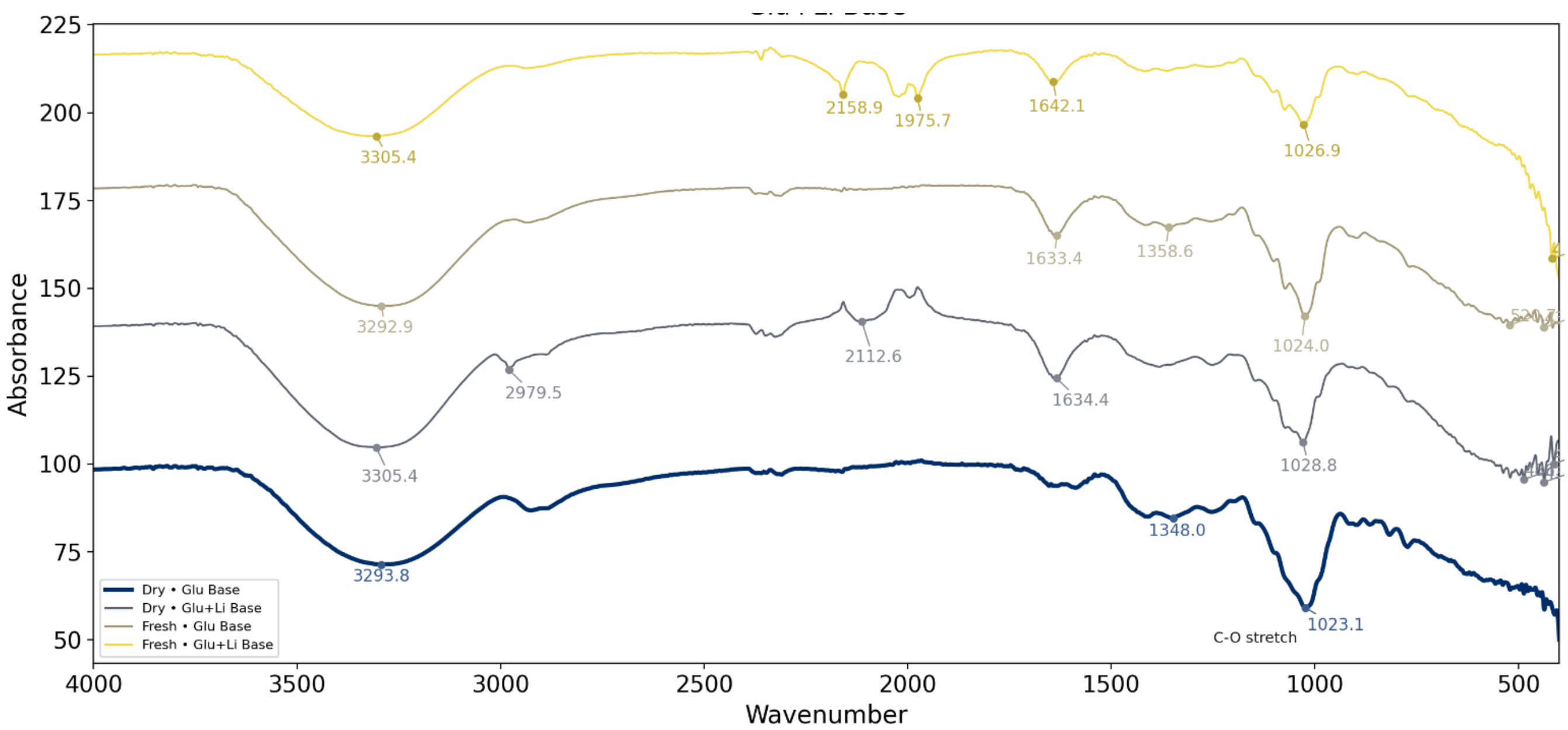

Figure S11. FTIR spectra upon single-step dry-down reactions of glucose (glu) and LiCl. Glu was dried in the absence or presence of LiCl at 50 ˚C for seven days upon addition of NaOH. Products were analyzed by FTIR and compared to unreacted stock solutions in the absence or presence of LiCl.

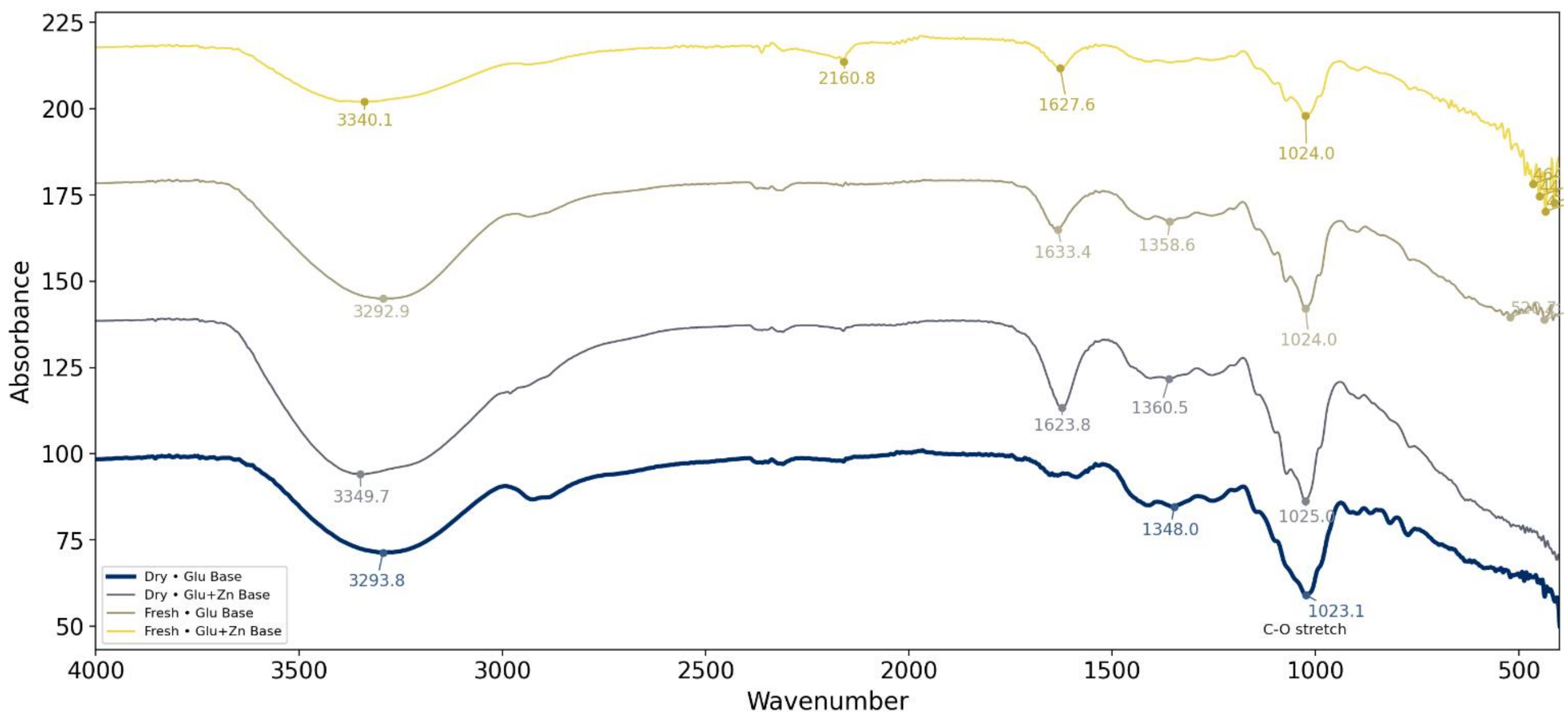

Figure S12. FTIR spectra upon single-step dry-down reactions of glucose (glu) and $ZnCl_2$. Glu was dried in the absence or presence of $ZnCl_2$ at 50 ˚C for seven days upon addition of NaOH. Products were analyzed by FTIR and compared to unreacted stock solutions in the absence or presence of $ZnCl_2$.

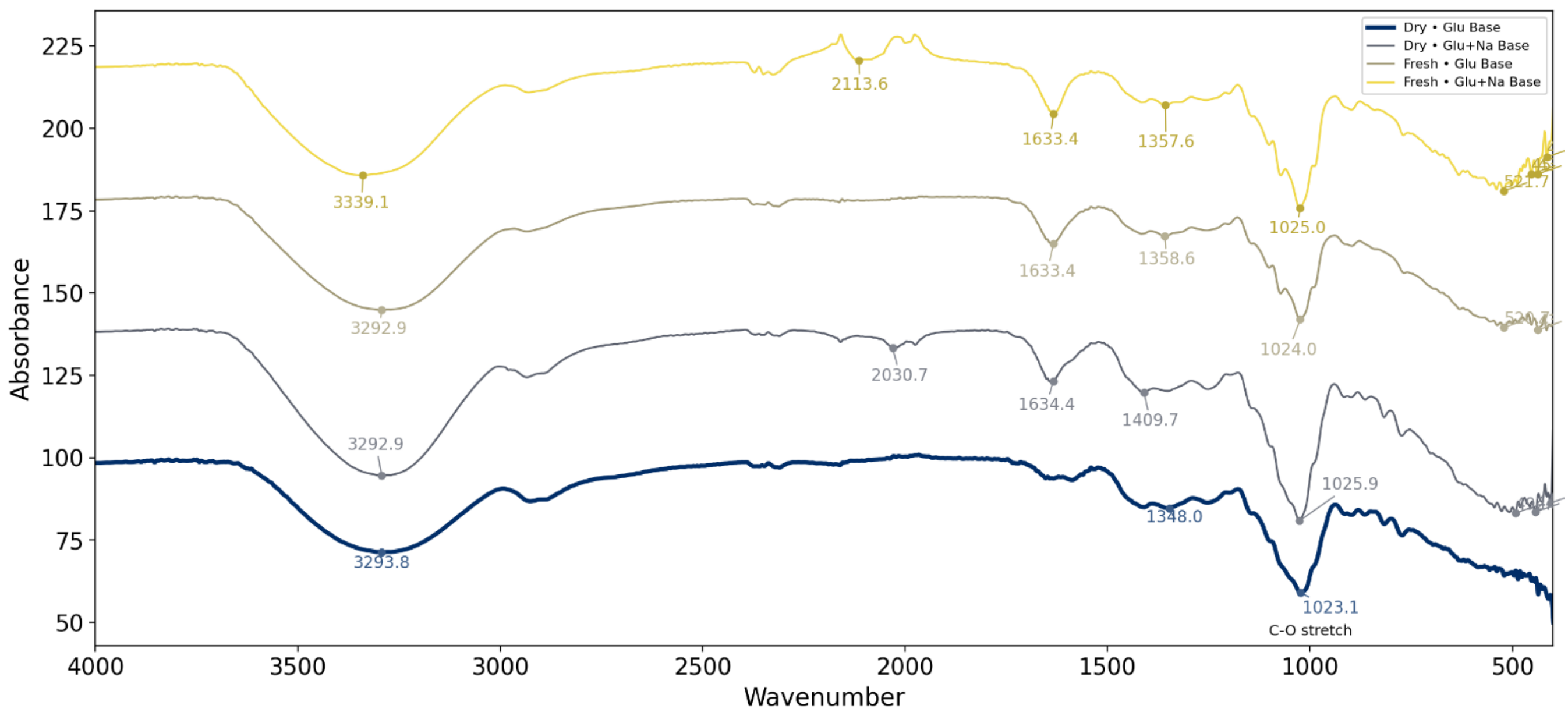

Figure S13. FTIR spectra upon single-step dry-down reactions of glucose (glu) and NaCl. Glu was dried in the absence or presence of NaCl at 50 ˚C for seven days upon addition of NaOH. Products were analyzed by FTIR and compared to unreacted stock solutions in the absence or presence of NaCl.

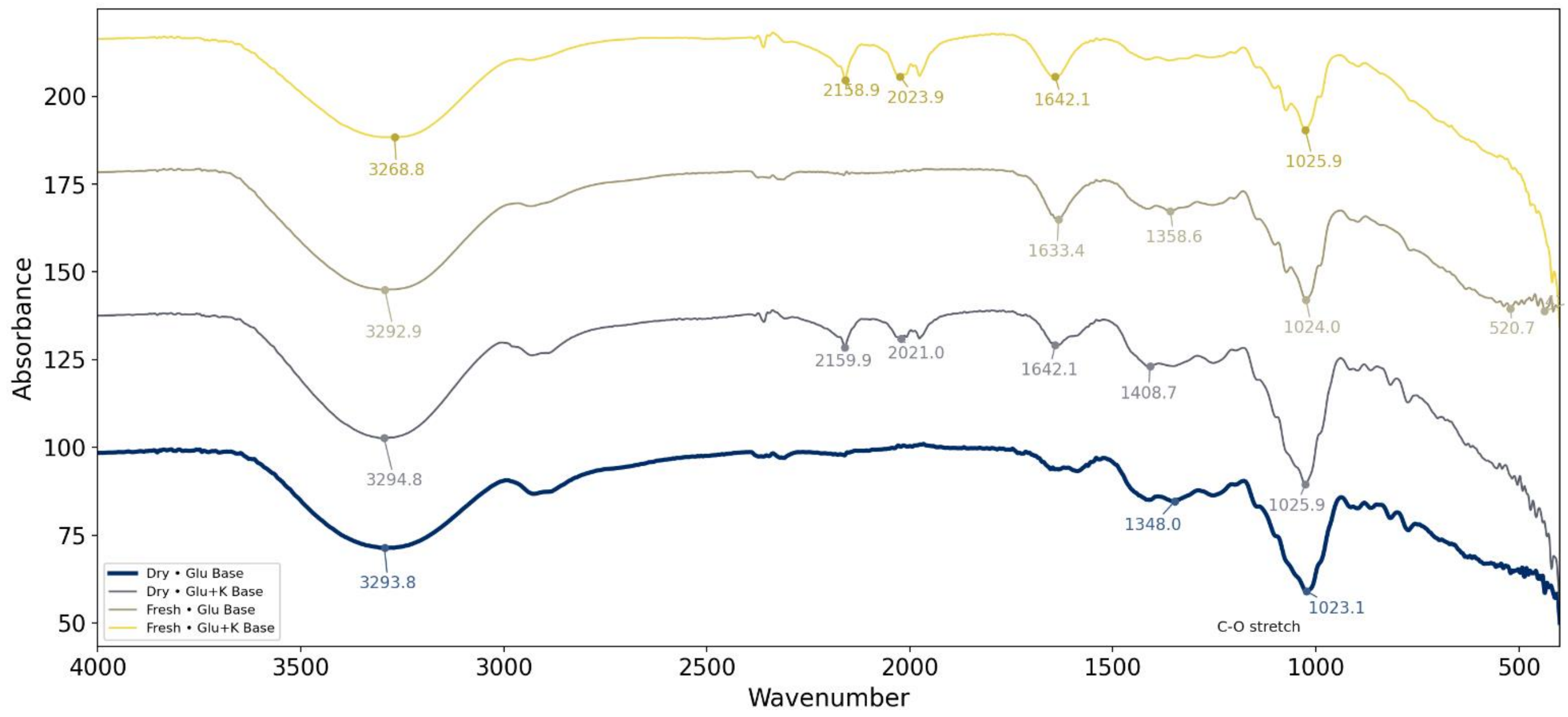

Figure S14. FTIR spectra upon single-step dry-down reactions of glucose (glu) and KCl. Glu was dried in the absence or presence of KCl at 50 ˚C for seven days upon addition of NaOH. Products were analyzed by FTIR and compared to unreacted stock solutions in the absence or presence of KCl**.**

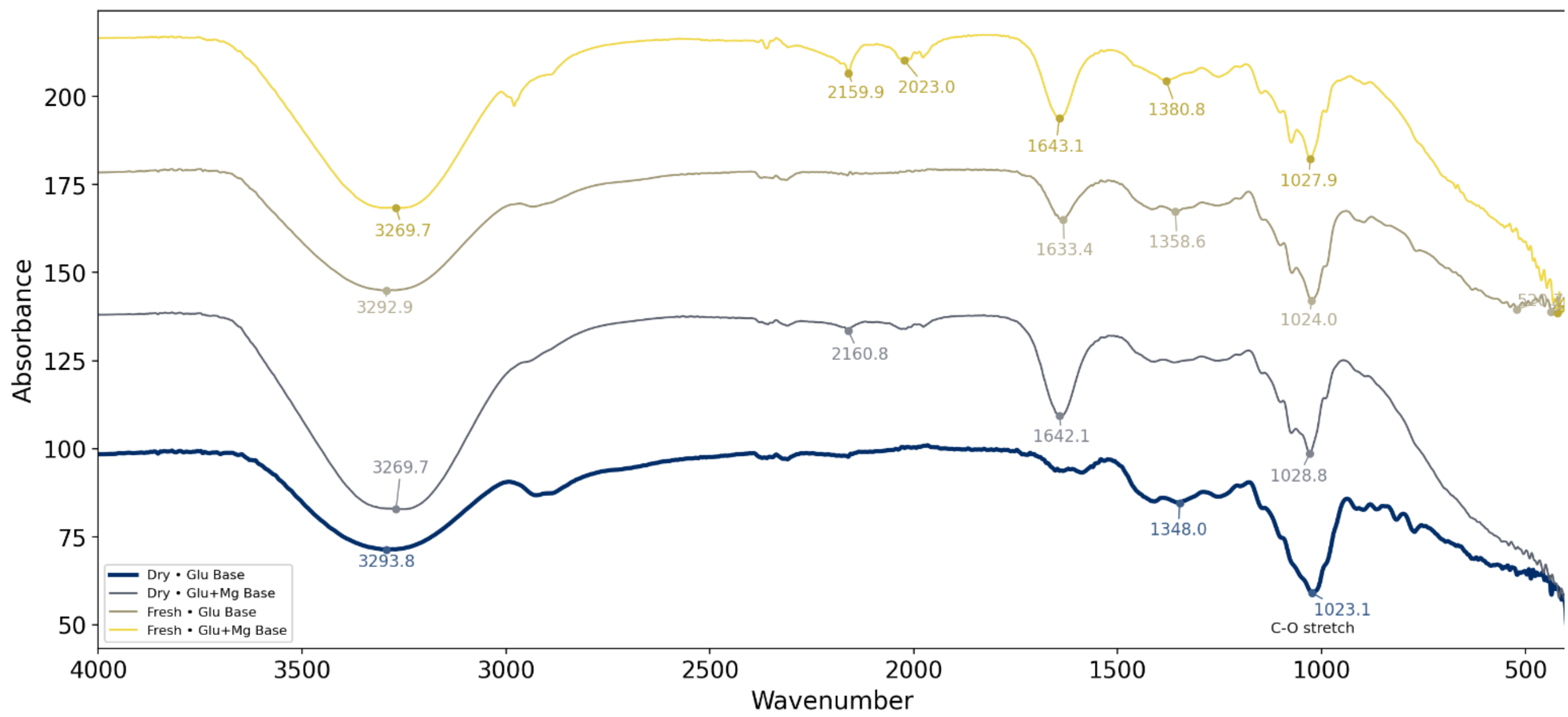

Figure S15. FTIR spectra upon single-step dry-down reactions of glucose (glu) and $MgCl_2$. Glu was dried in the absence or presence of $MgCl_2$ at 50 ˚C for seven days upon addition of NaOH. Products were analyzed by FTIR and compared to unreacted stock solutions in the absence or presence of $MgCl_2$.

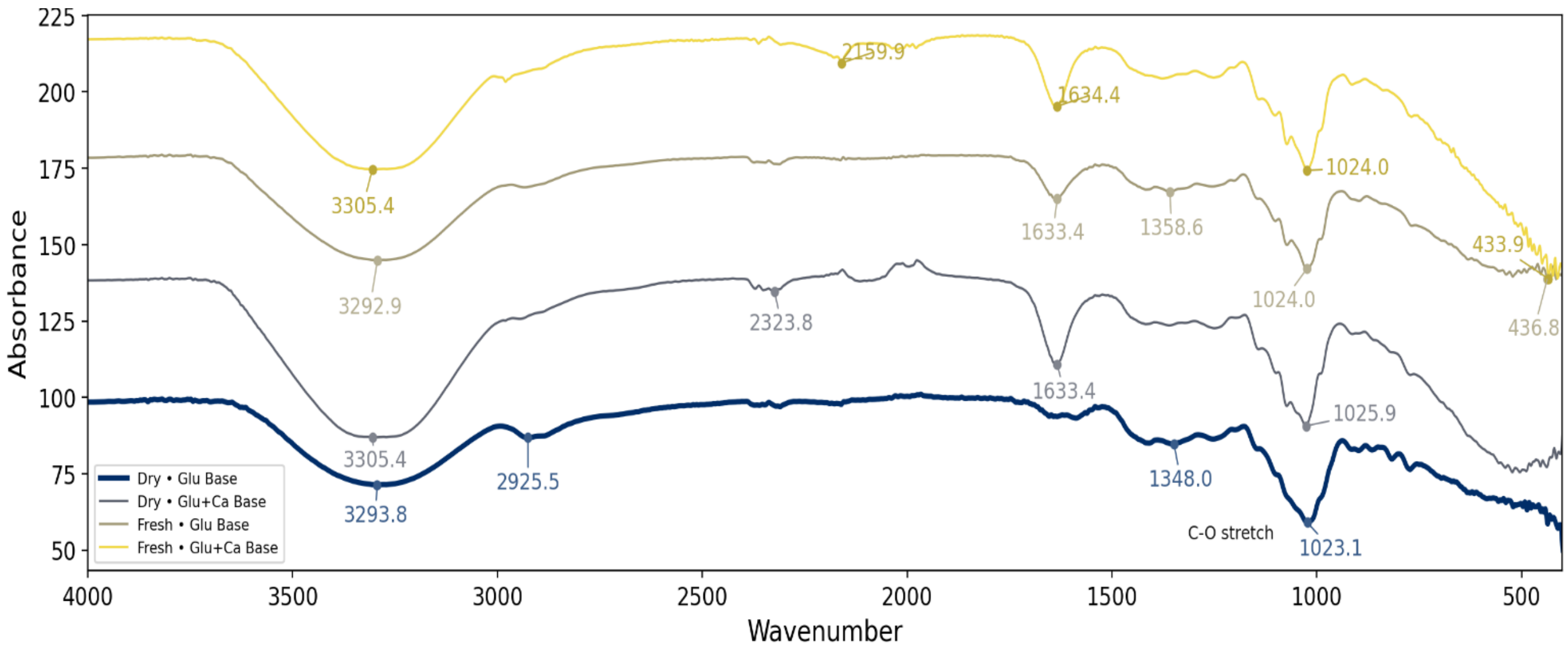

Figure S16. FTIR spectra upon single-step dry-down reactions of glucose (glu) and $CaCl_2$. Glu was dried in the absence or presence of $CaCl_2$ at 50 ˚C for seven days upon addition of NaOH. Products were analyzed by FTIR and compared to unreacted stock solutions in the absence or presence of $CaCl_2$.

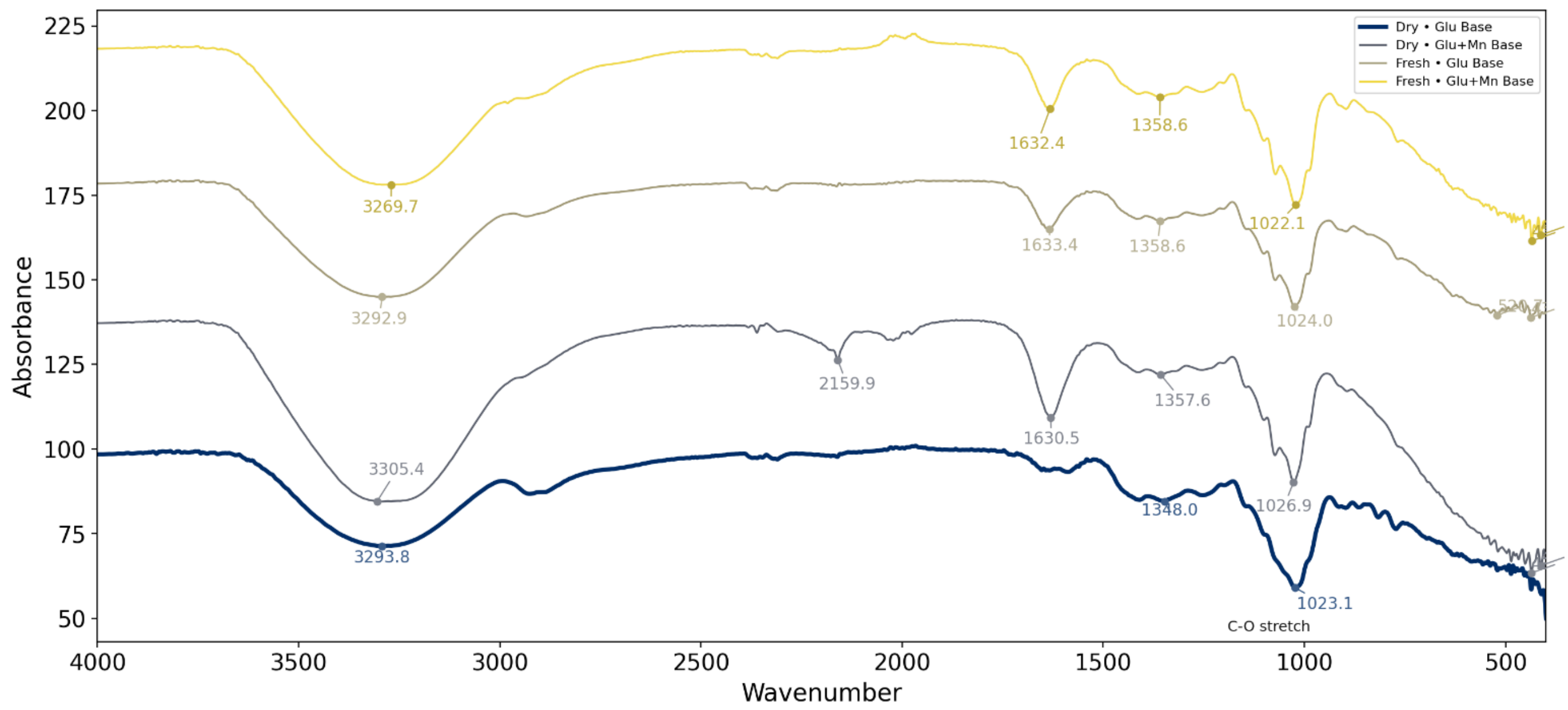

Figure S17. FTIR spectra upon single-step dry-down reactions of glucose (glu) and $MnCl_2$. Glu was dried in the absence or presence of $MnCl_2$ at 50 ˚C for seven days upon addition of NaOH. Products were analyzed by FTIR and compared to unreacted stock solutions in the absence or presence of $MnCl_2$.

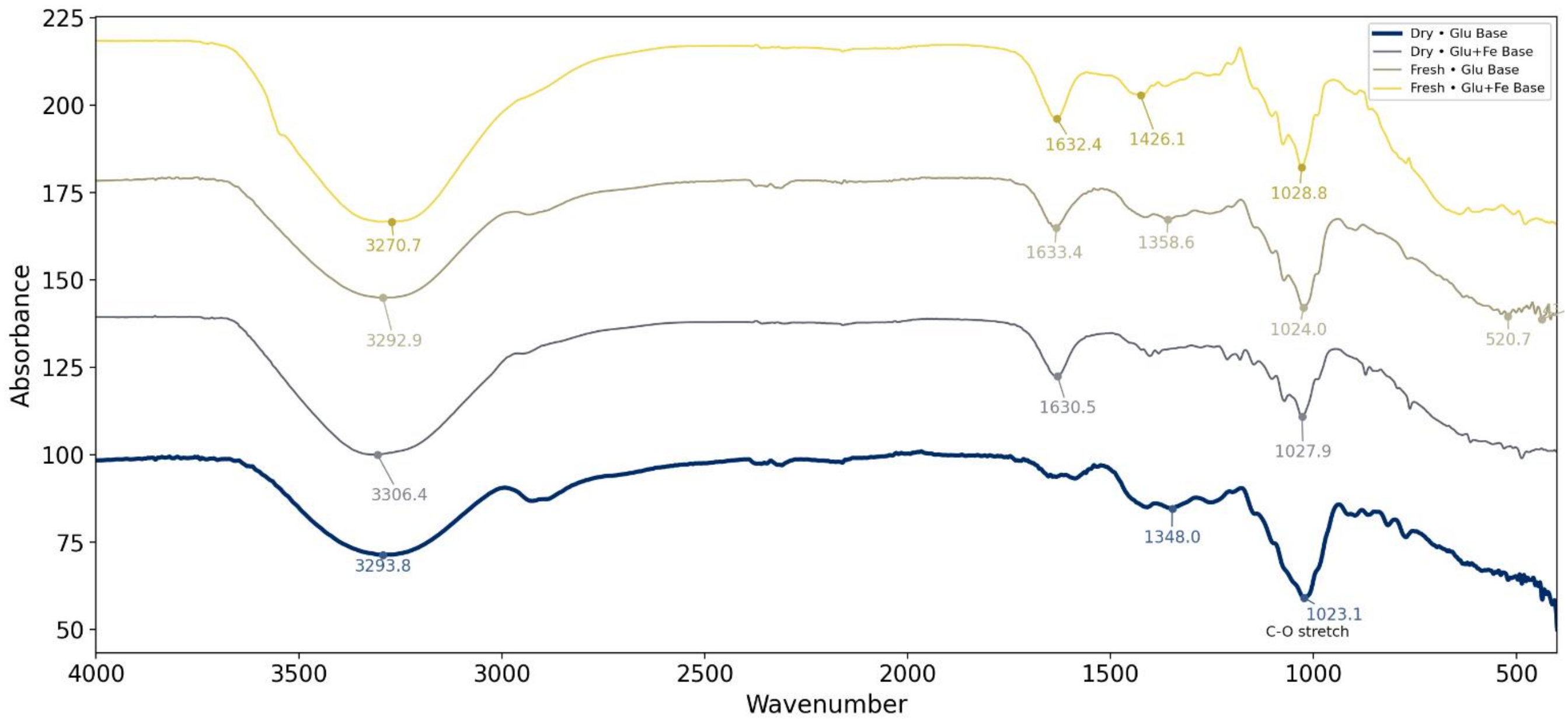

Figure S18. FTIR spectra upon single-step dry-down reactions of glucose (glu) and $FeCl_2$. Glu was dried in the absence or presence of $FeCl_2$ at 50 ˚C for seven days upon addition of NaOH. Products were analyzed by FTIR and compared to unreacted stock solutions in the absence or presence of $FeCl_2$.

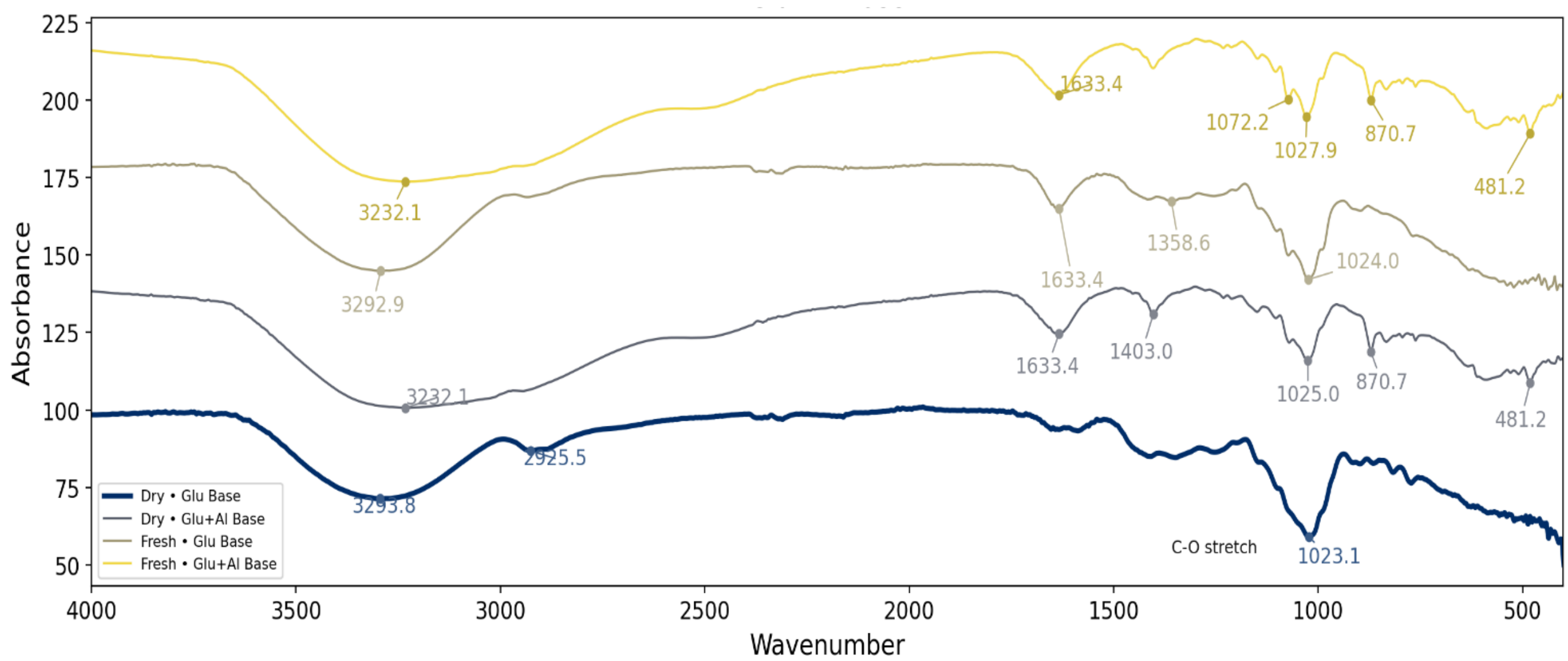

Figure S19. FTIR spectra upon single-step dry-down reactions of glucose (glu) and $AlCl_3$. Glu was dried in the absence or presence of $AlCl_3$ at 50 ˚C for seven days upon addition of NaOH. Products were analyzed by FTIR and compared to unreacted stock solutions in the absence or presence of $AlCl_3$.

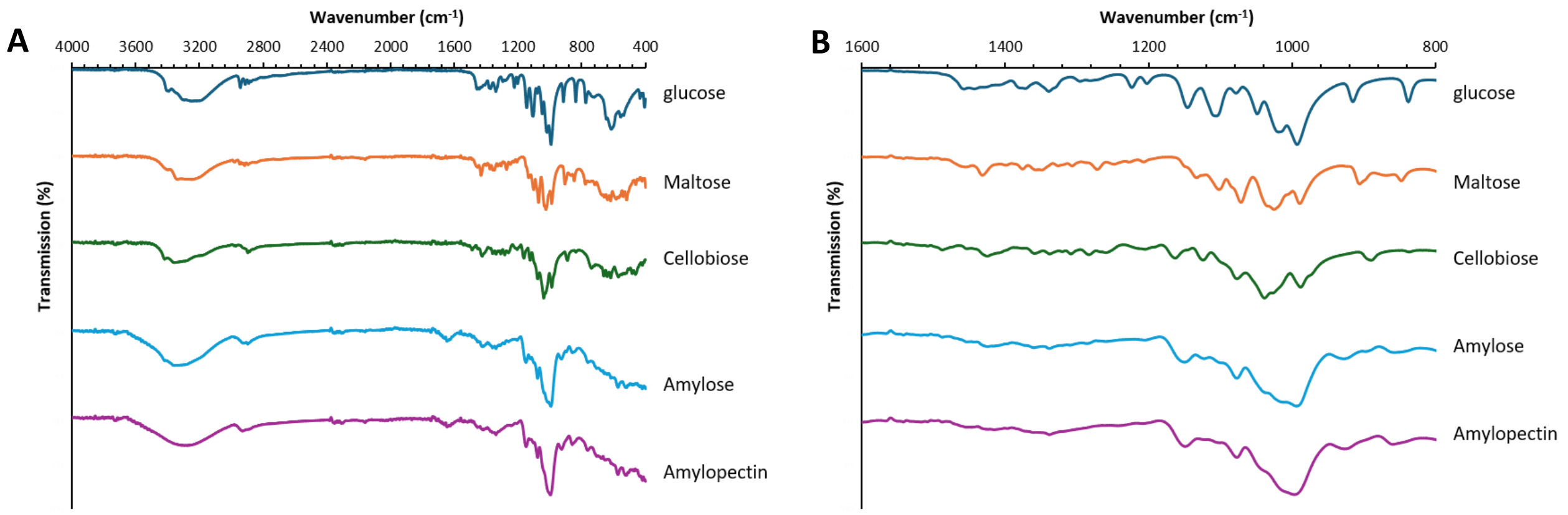

Figure S20. FTIR signals of glucose oligomer standards. The FTIR spectra were obtained for fresh glucose, maltose (α1→4 linkage), cellobiose (β1→4 linkage), amylose (α1→4 linkage), and amylopectin (α1→4 and α1→46 linkages). The full spectrum is shown in A, and a zoomed-in view is shown in B. As indicated by the spectra, the signals in the region assigned to the C-O-H bending are significantly shifted and broadened upon glucose oligomerization. While mostly signal shifts are observed when comparing the dimers to glucose, at higher degrees of oligomerization (as in the case of amylose and amylopectin), the signals become broader and less discrete compared to glucose.

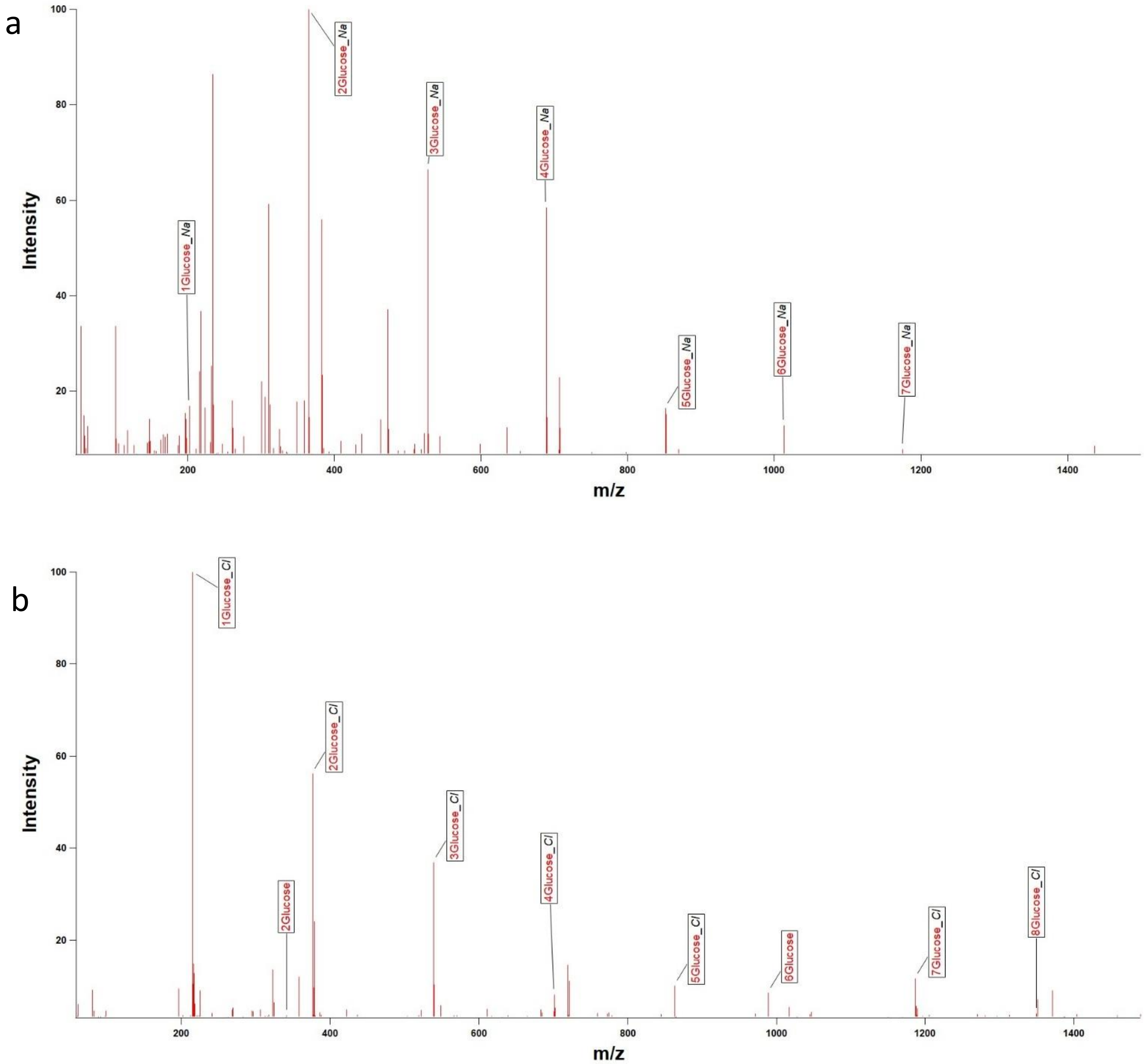

Figure S21. ESI-MS of a dry-down reaction of glucose (glu) in an acidified environment supports the formation of oligosaccharides. Glu was dried at 50˚C for seven days and the resulting oligosaccharides were analyzed by positive mode ESI-MS (a) or negative-mode ESI-MS (b), indicating a variety of oligosaccharides. Labeled species correspond to $[M + Na]^{+}$ ions in the positive mode (a), or to $[M - H]^{-}$ or $[M + Cl]^{-}$ ions in the negative mode (b).

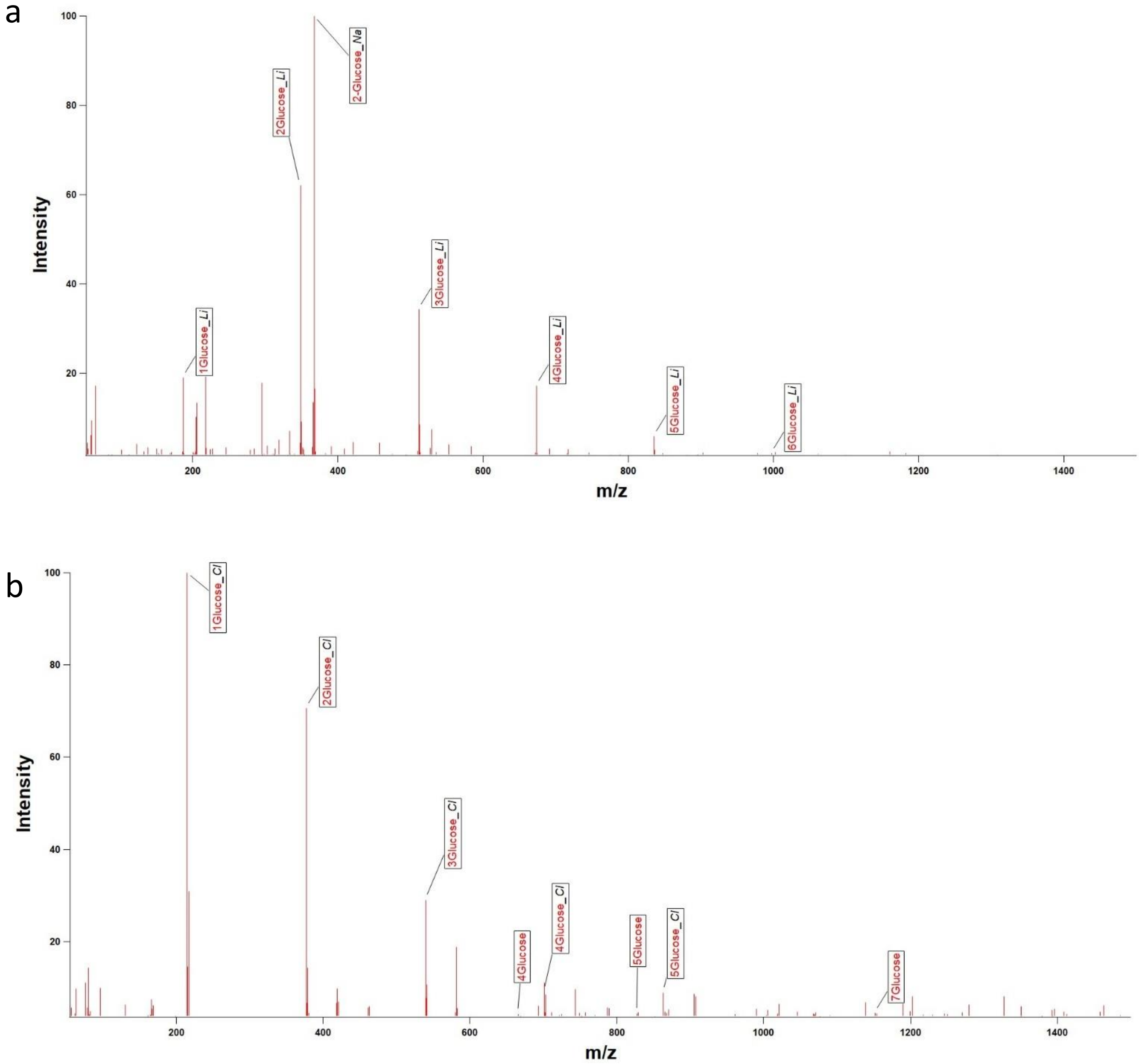

Figure S22. ESI-MS of a dry-down reaction of glucose (glu) and LiCl in an acidified environment supports the formation of oligosaccharides. Glu and LiCl were dried at 50˚C for seven days and the resulting oligosaccharides were analyzed by positive mode ESI-MS (a) or negative-mode ESI-MS (b), indicating a variety of oligosaccharides. Labeled species correspond to $[M + Li]^+$ ions (a) or $[M - H]^-$ or $[M + Cl]^-$ ions (b).

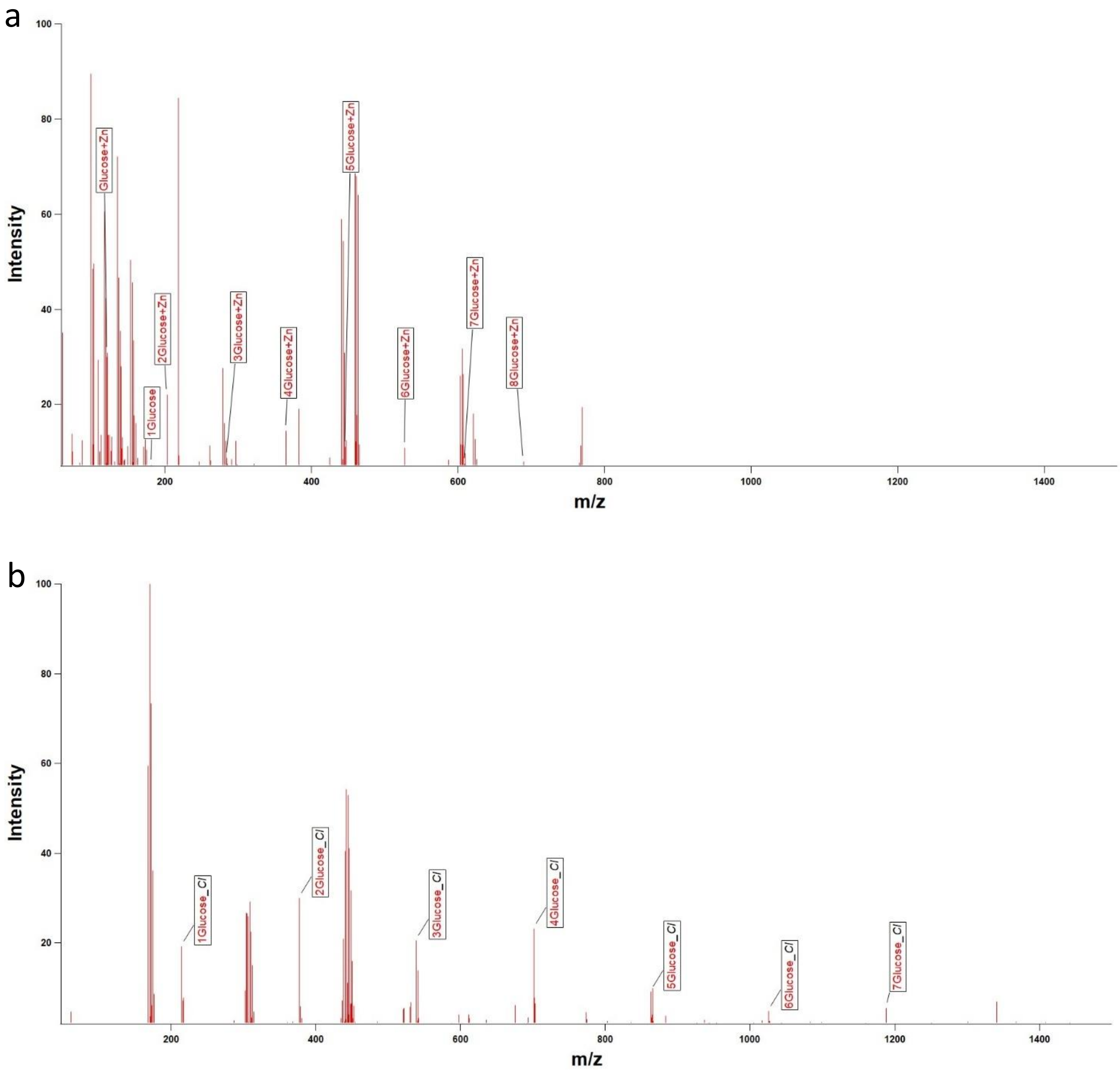

Figure S23. ESI-MS of a dry-down reaction of glucose (glu) and $ZnCl_2$ in an acidified environment supports the formation of oligosaccharides. Glu and $ZnCl_2$ were dried at 50˚C for seven days and the resulting oligosaccharides were analyzed by positive mode ESI-MS (a) or negative-mode ESI-MS (b), indicating a variety of oligosaccharides. Labeled species correspond to $[M + H]^{+}$or $[M + Zn]^{2+}$ ions (a) or $[M + Cl]^{-}$ ions (b).

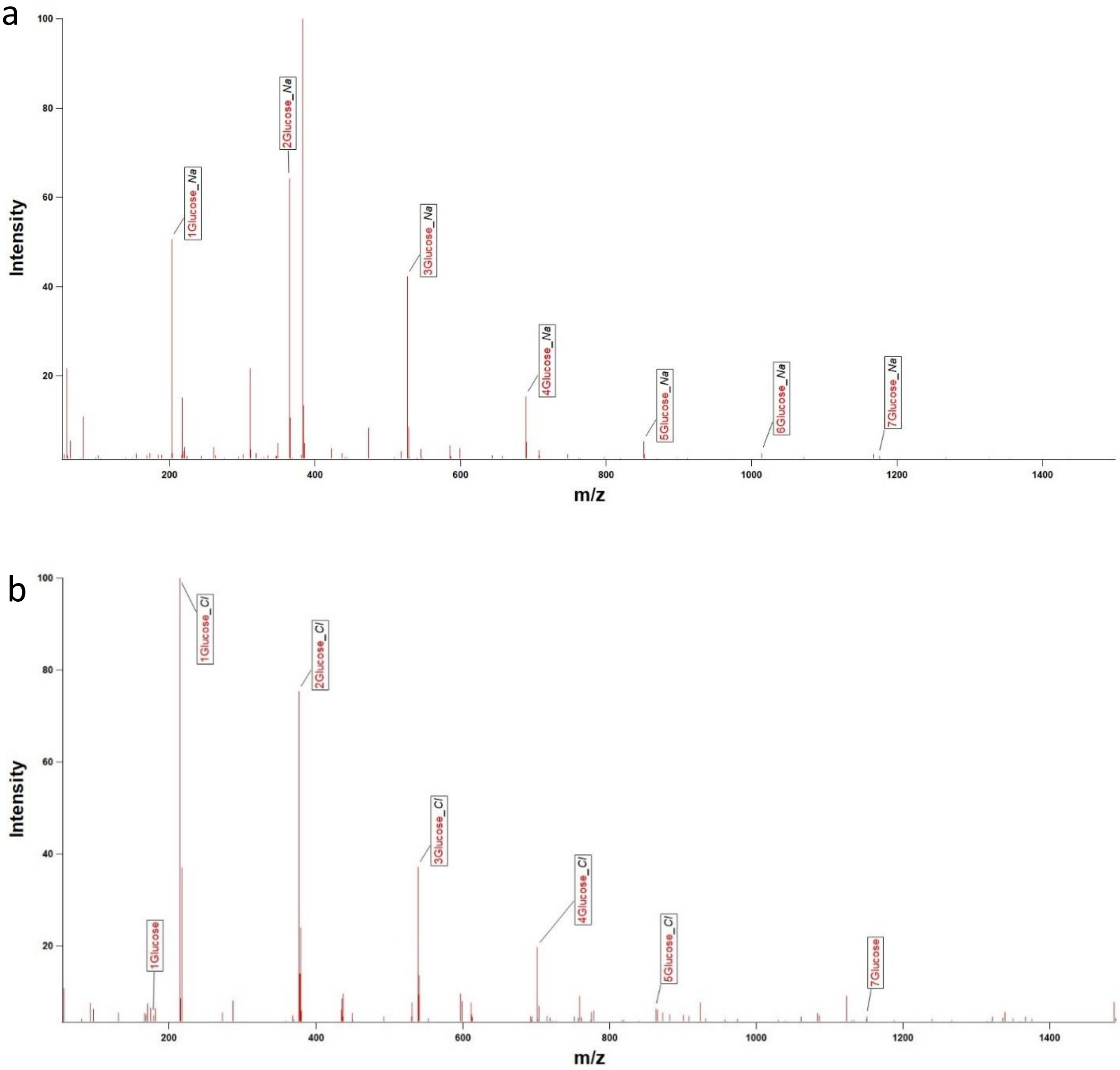

Figure S24. ESI-MS of a dry-down reaction of glucose (glu) and NaCl in an acidified environment supports the formation of oligosaccharides. Glu and NaCl were dried at 50˚C for seven days and the resulting oligosaccharides were analyzed by positive mode ESI-MS (a) or negative-mode ESI-MS (b), indicating a variety of oligosaccharides. Labeled species correspond to $[M+Na]^{+}$ ions (a) or $[M-H]^{-}$ or $[M+Cl]^{-}$ ions (b).

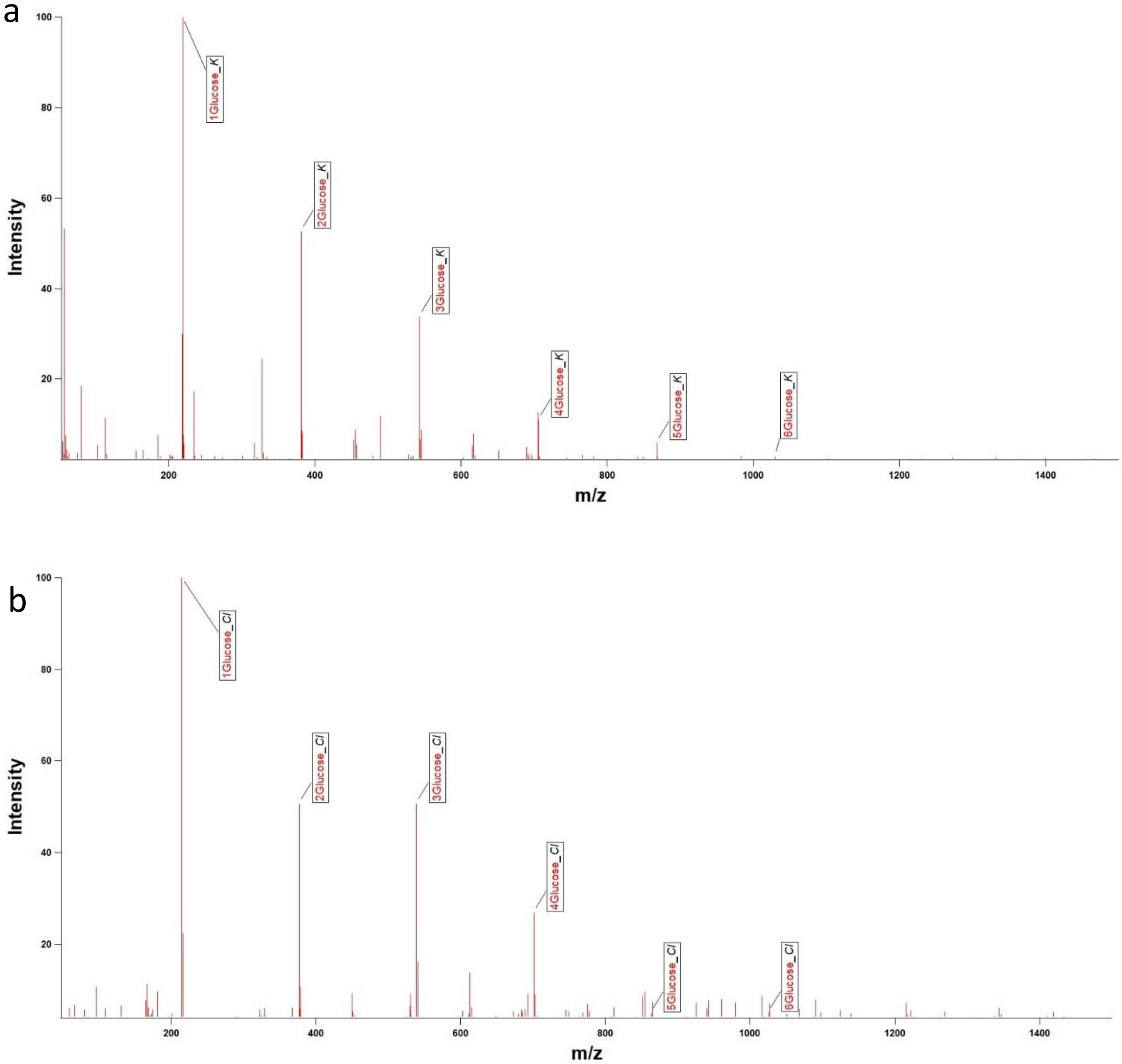

Figure S25. ESI-MS of a dry-down reaction of glucose (glu) and KCl in an acidified environment supports the formation of oligosaccharides. Glu and KCl were dried at 50˚C for seven days and the resulting oligosaccharides were analyzed by positive mode ESI-MS (a) or negative-mode ESI-MS (b), indicating a variety of oligosaccharides. Labeled species correspond to $[M + K]^{+}$ions (a) or$[M + Cl]^{-}$ ions (b).

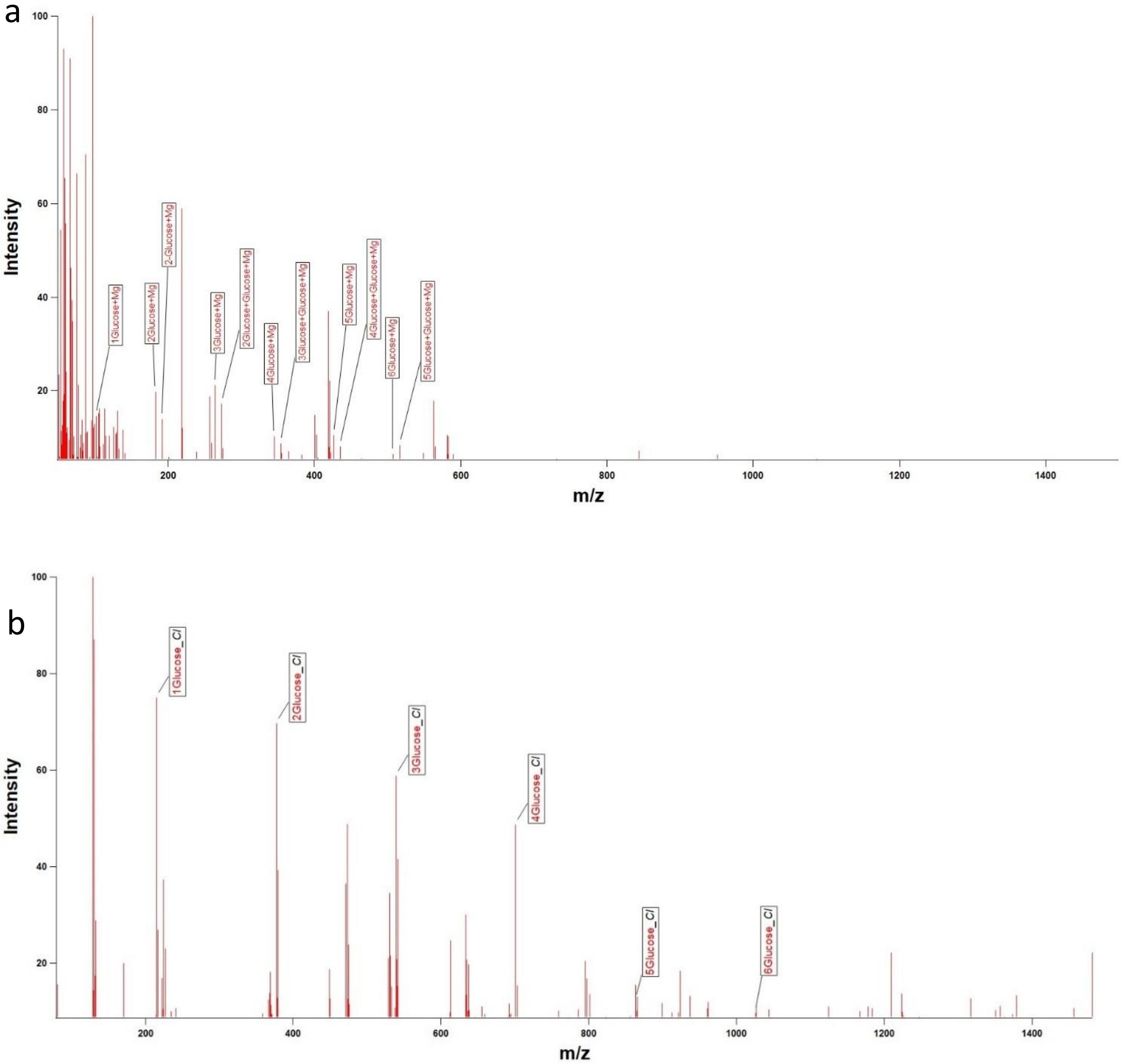

Figure S26. ESI-MS of a dry-down reaction of glucose (glu) and $MgCl_2$ in an acidified environment supports the formation of oligosaccharides. Glu and $MgCl_2$ were dried at 50˚C for seven days and the resulting oligosaccharides were analyzed by positive mode ESI-MS (a) or negative-mode ESI-MS (b), indicating a variety of oligosaccharides. Labeled species correspond to $[M + Mg]^{2+}$ ions (a) or $[M + Cl]^{-}$ ions (b).

a

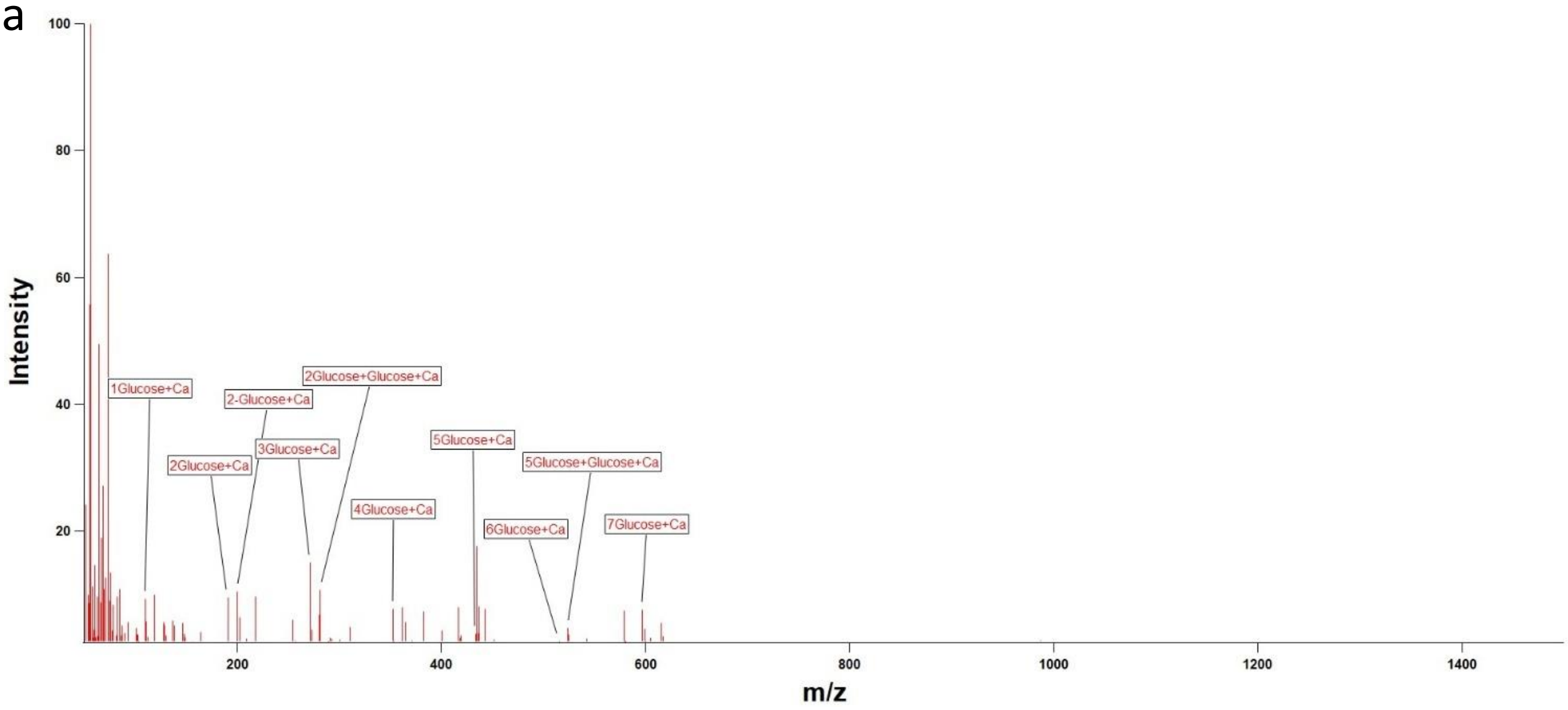

b

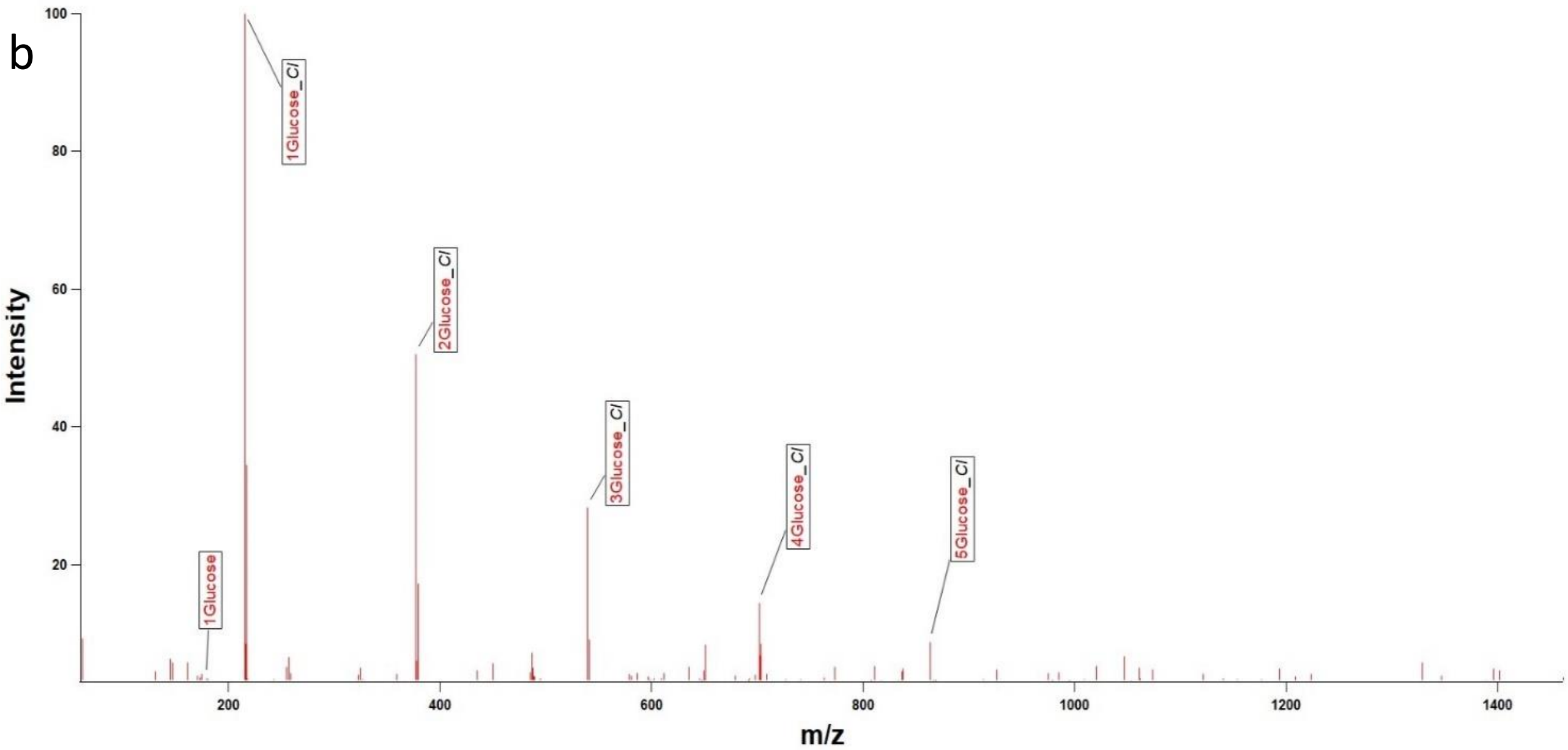

Figure S27. ESI-MS of a dry-down reaction of glucose (glu) and $CaCl_2$ in an acidified environment supports the formation of oligosaccharides. Glu and $CaCl_2$ were dried at 50˚C for seven days and the resulting oligosaccharides were analyzed by positive mode ESI-MS (a) or negative-mode ESI-MS (b), indicating a variety of oligosaccharides. Labeled species correspond to $[M+Ca]^{2+}$ ions (a) or $[M-H]^-$ or $[M+Cl]^-$ ions (b).

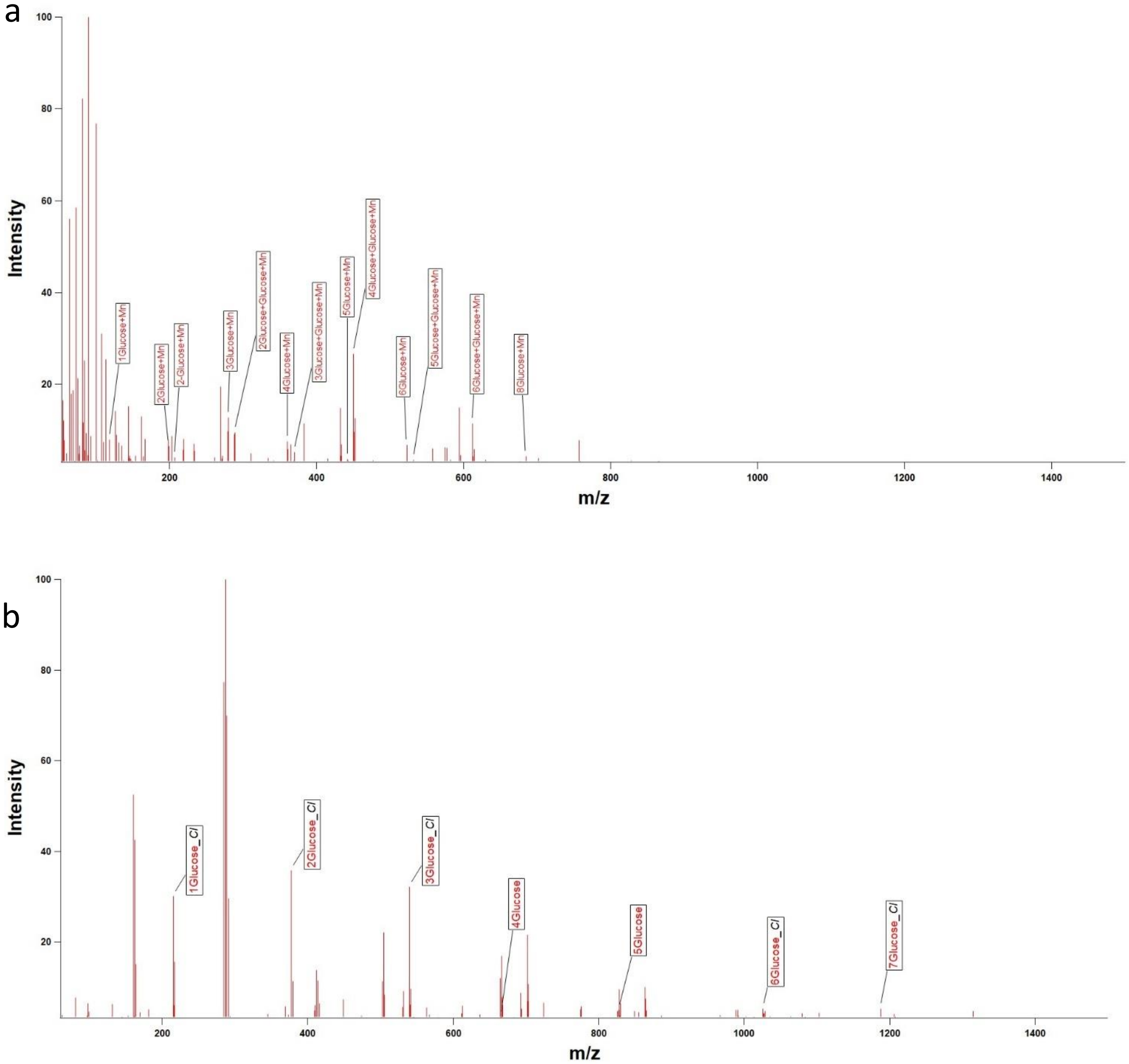

Figure S28. ESI-MS of a dry-down reaction of glucose (glu) and $MnCl_2$ in an acidified environment supports the formation of oligosaccharides. Glu and $MnCl_2$ were dried at 50˚C for seven days and the resulting oligosaccharides were analyzed by positive mode ESI-MS (a) or negative-mode ESI-MS (b), indicating a variety of oligosaccharides. Labeled species correspond to $[M + Mn]^{2+}$ ions (a) or $[M - H]^-$ or $[M + Cl]^-$ ions (b).

a

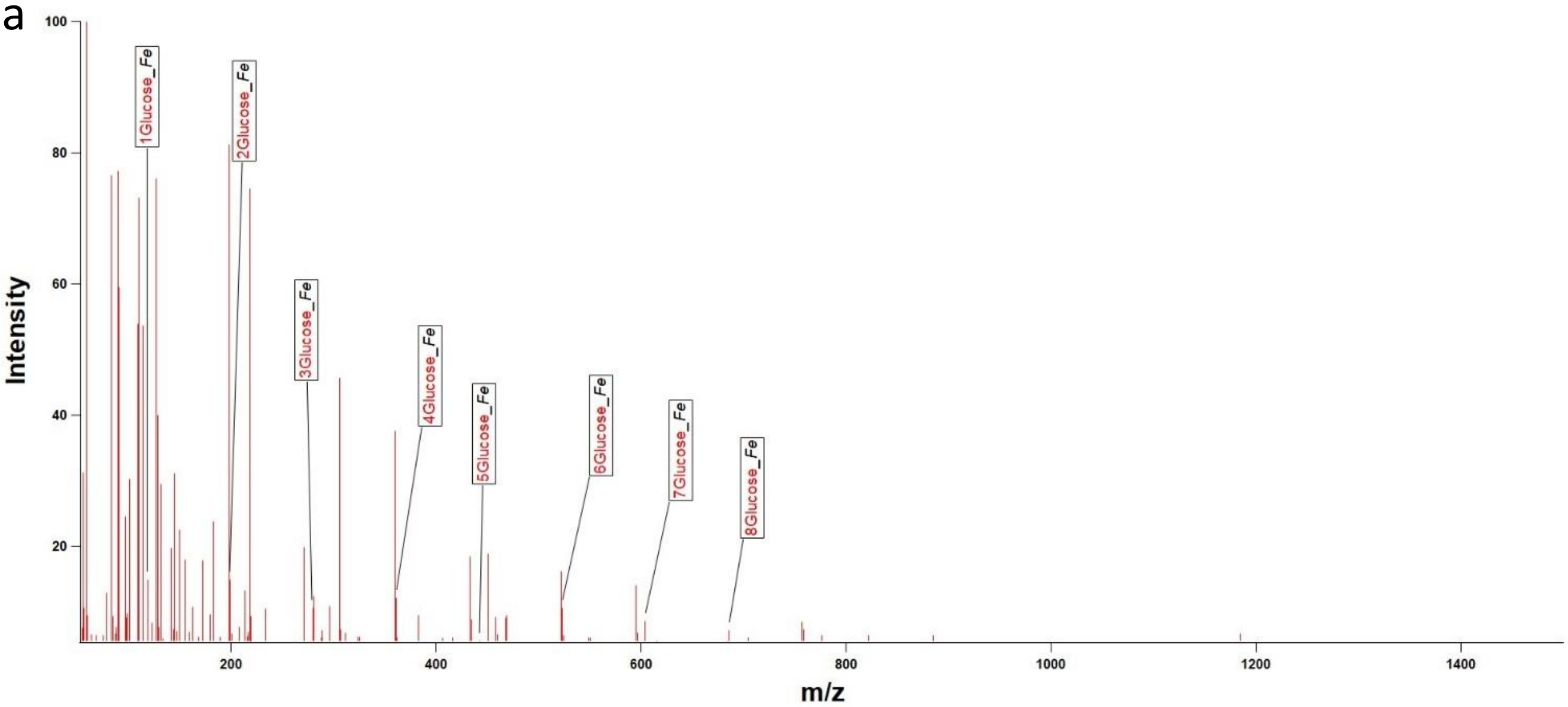

b

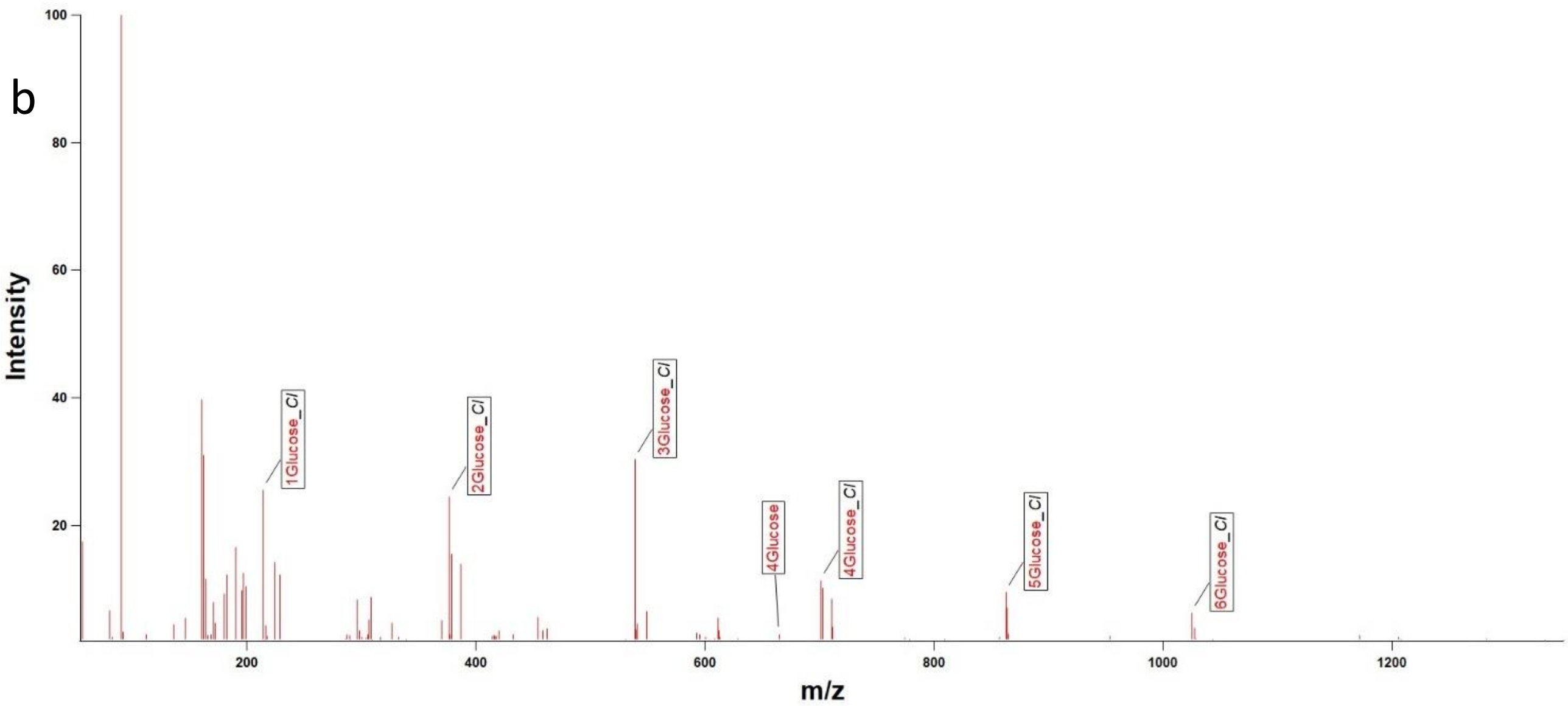

Figure S29. ESI-MS of a dry-down reaction of glucose (glu) and $FeCl_2$ in an acidified environment supports the formation of oligosaccharides. Glu and $FeCl_2$ were dried at 50˚C for seven days and the resulting oligosaccharides were analyzed by positive mode ESI-MS (a) or negative-mode ESI-MS (b), indicating a variety of oligosaccharides. Labeled species correspond to $[M + Fe]^{2+}$ ions (a) or $[M - H]^-$ or $[M + Cl]^-$ ions (b).

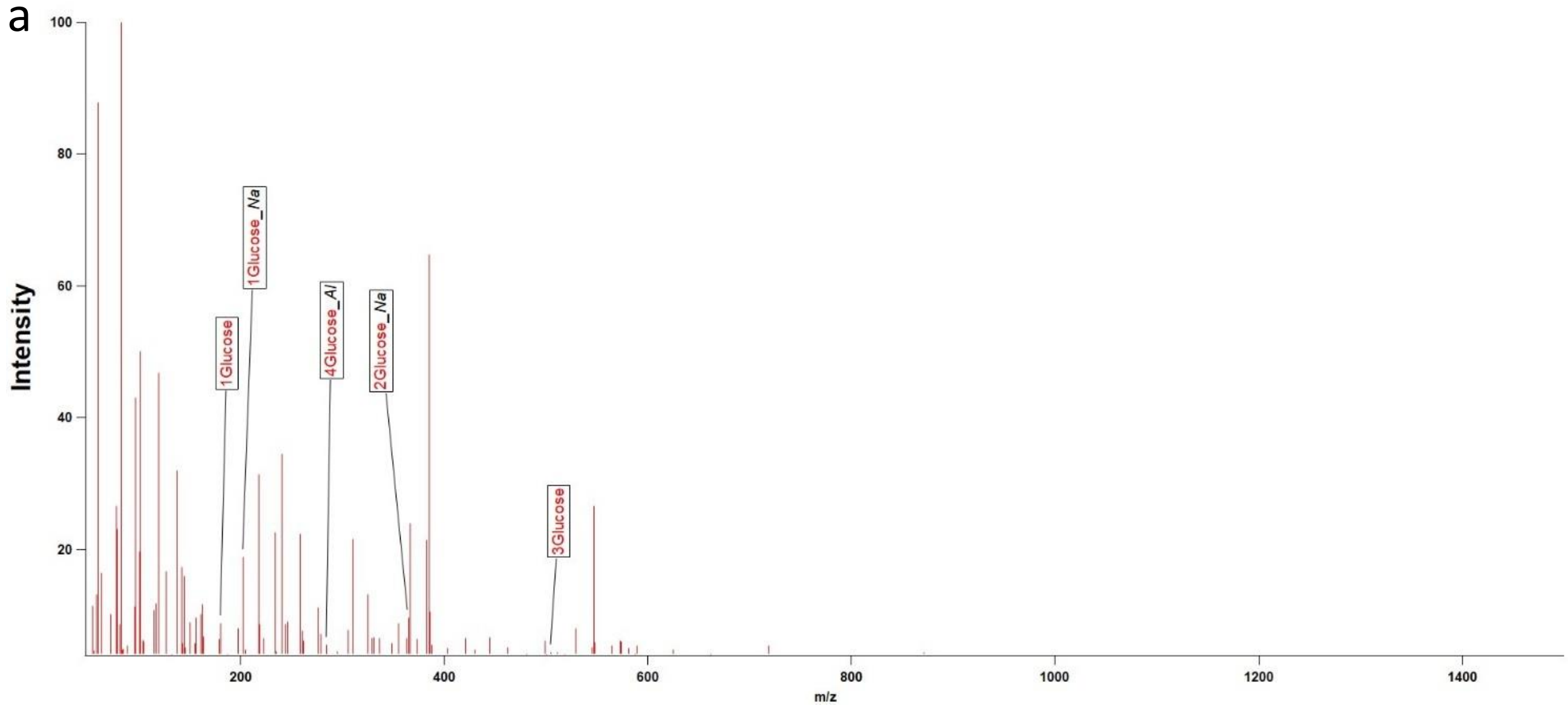

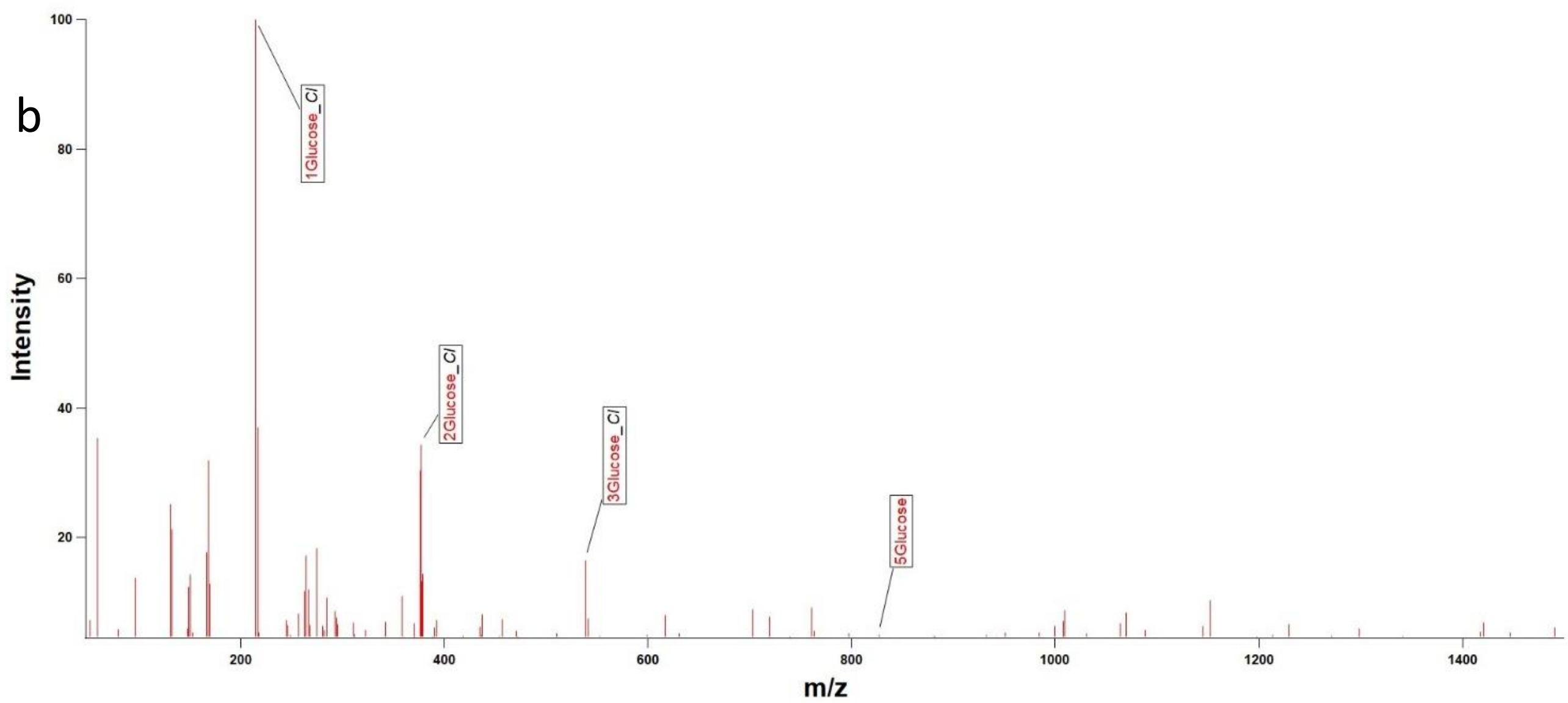

Figure S30. ESI-MS of a dry-down reaction of glucose (glu) and $AlCl_3$ in an acidified environment supports the formation of oligosaccharides. Glu and $AlCl_3$ were dried at 50˚C for seven days and the resulting oligosaccharides were analyzed by positive mode ESI-MS (a) or negative-mode ESI-MS (b), indicating a variety of oligosaccharides. Labeled species correspond to $[M+H]^+$ or $[M+Al]^{3+}$ or $[M+Na]^+$ ions (a) or $[M-H]^-$ or $[M+Cl]^-$ ions (b).

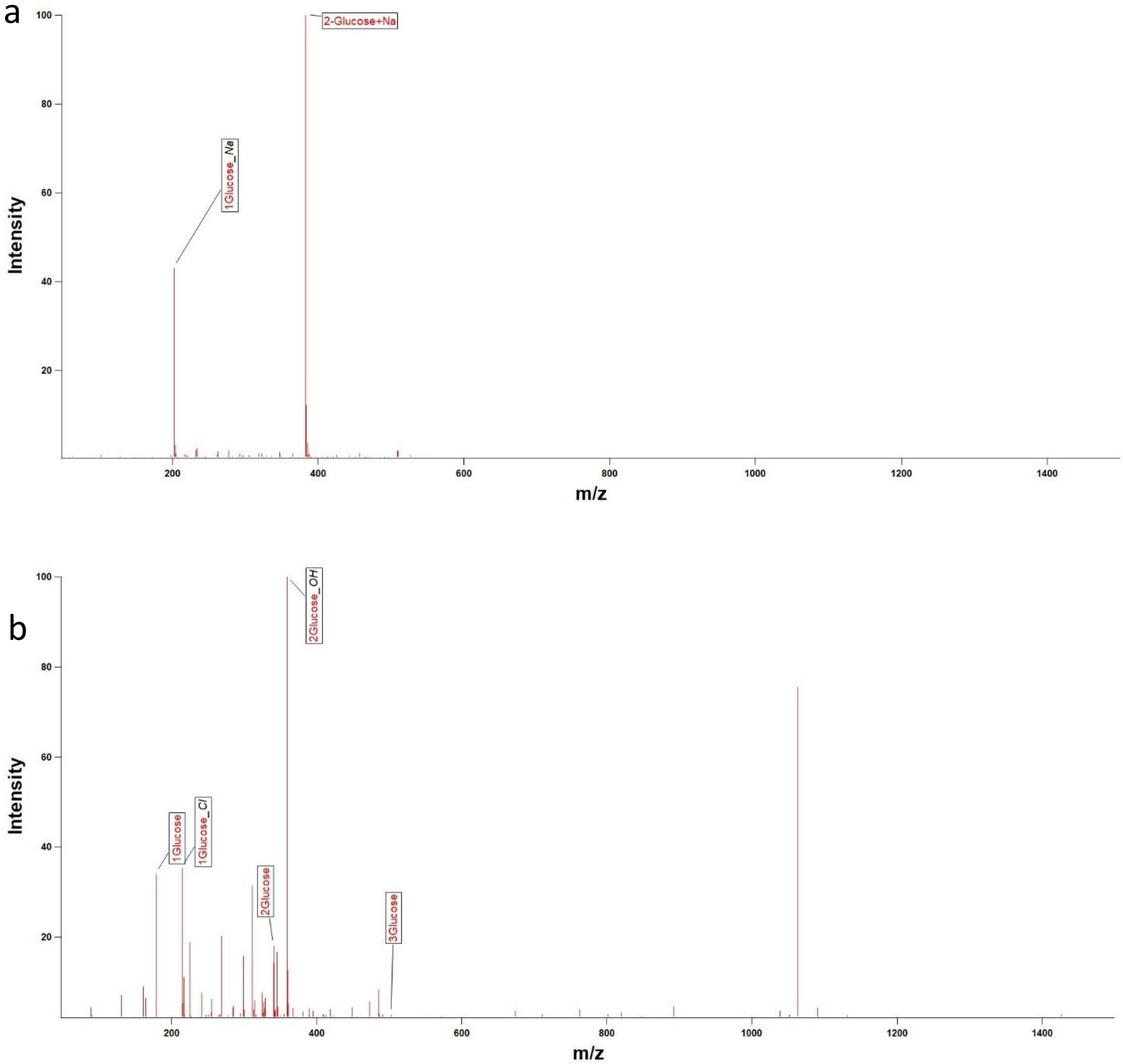

Figure S31. ESI-MS of a dry-down reaction of glucose (glu) upon addition of NaOH supports the formation of short oligosaccharides. Glu was dried at 50˚C for seven days and the resulting oligosaccharides were analyzed by positive mode ESI-MS (a) or negative-mode ESI-MS (b), indicating a small variety of oligosaccharides. Labeled species correspond to $[M + Na]^+$ ions (annotations with a hyphen indicate non-covalent complex) (a) or $[M - H]^-$ or $[M + Cl]^-$ or $[M + OH]^-$ ions (b).

a

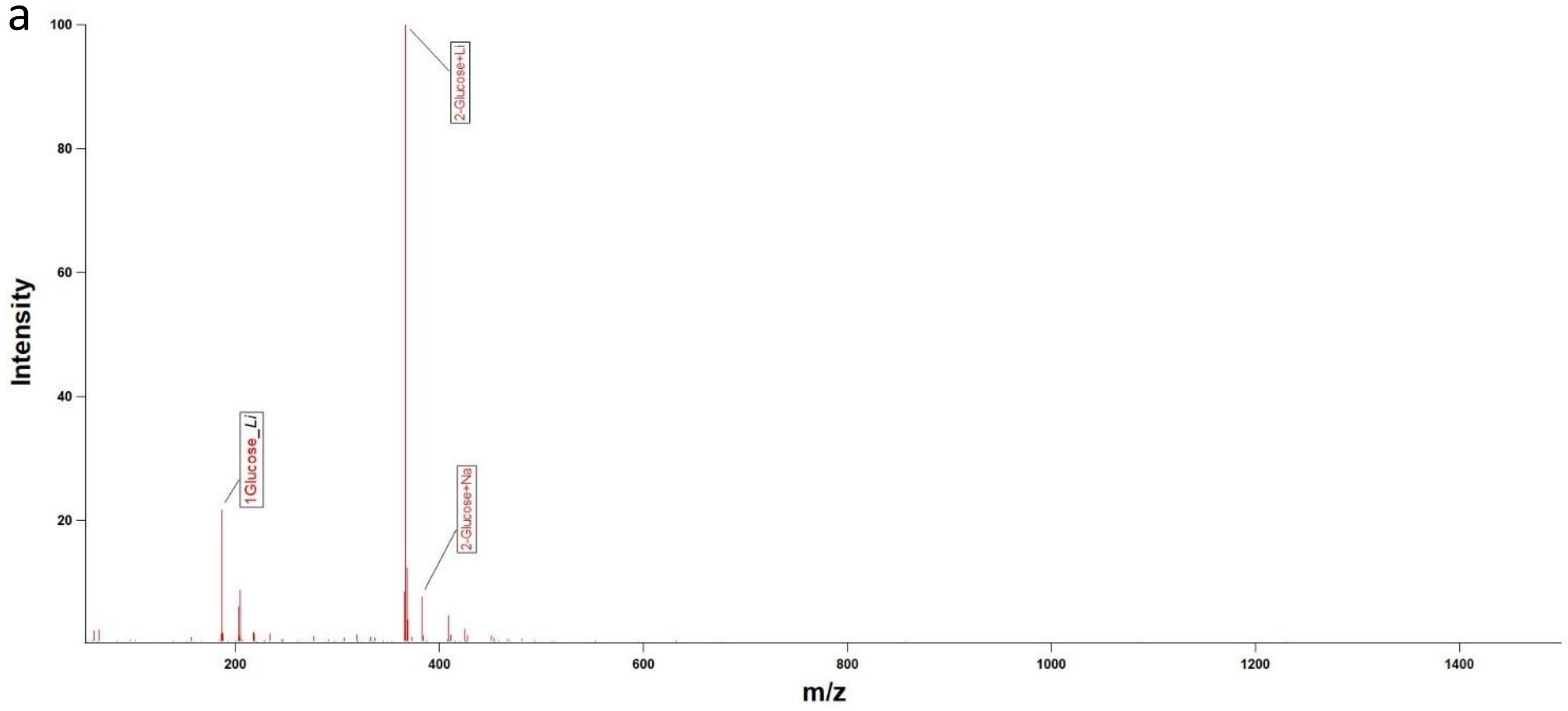

b

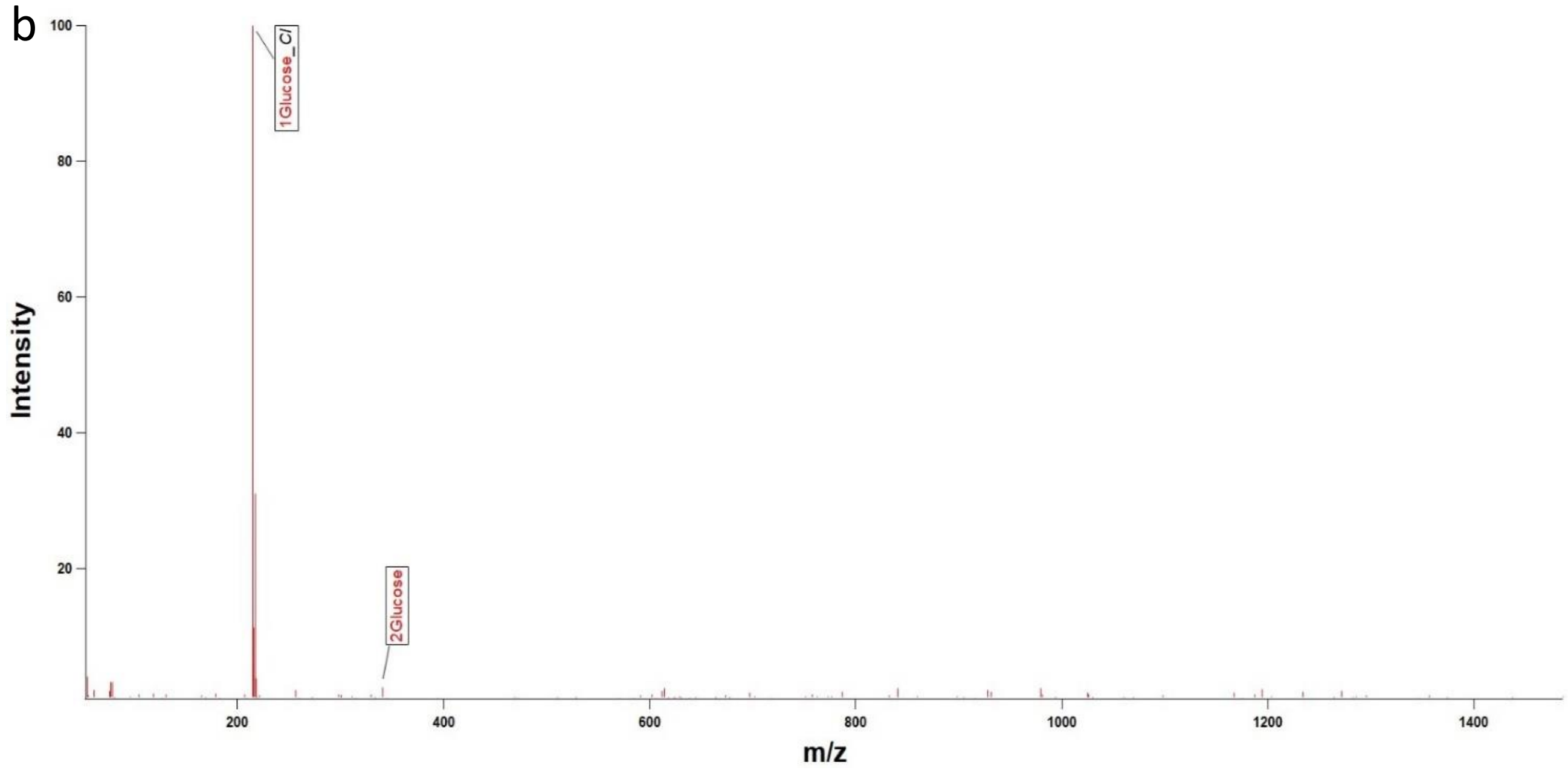

Figure S32. ESI-MS of a dry-down reaction of glucose (glu) and LiCl upon addition of NaOH. Glu and LiCl were dried at 50˚C for seven days and the resulting oligosaccharides were analyzed by positive mode ESI-MS (a) or negative-mode ESI-MS (b). Labeled species correspond to $[M + Li]^+$ or $[M + Na]^+$ ions (annotations with a hyphen indicate non-covalent complex) (a) or $[M - H]^-$ or $[M + Cl]^-$ ions (b).

a

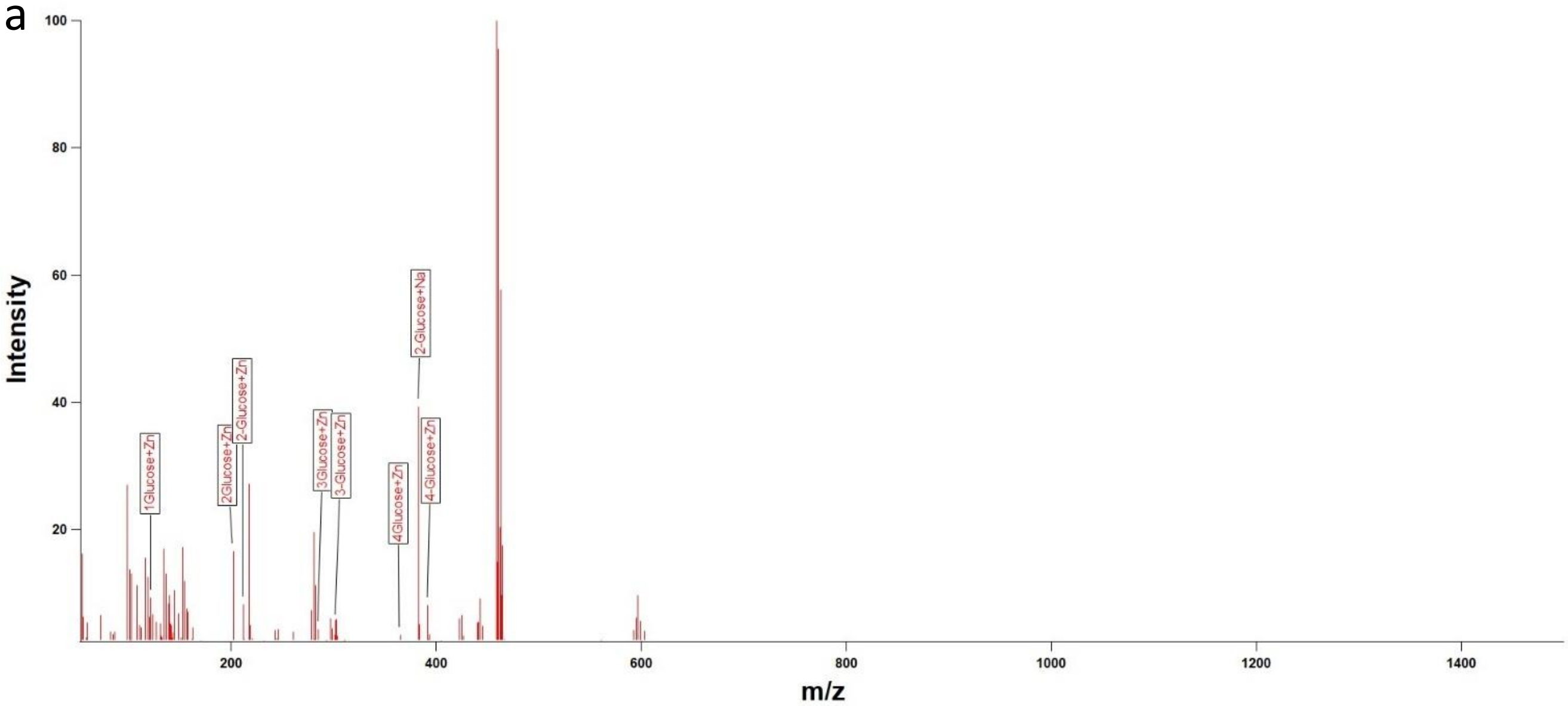

b

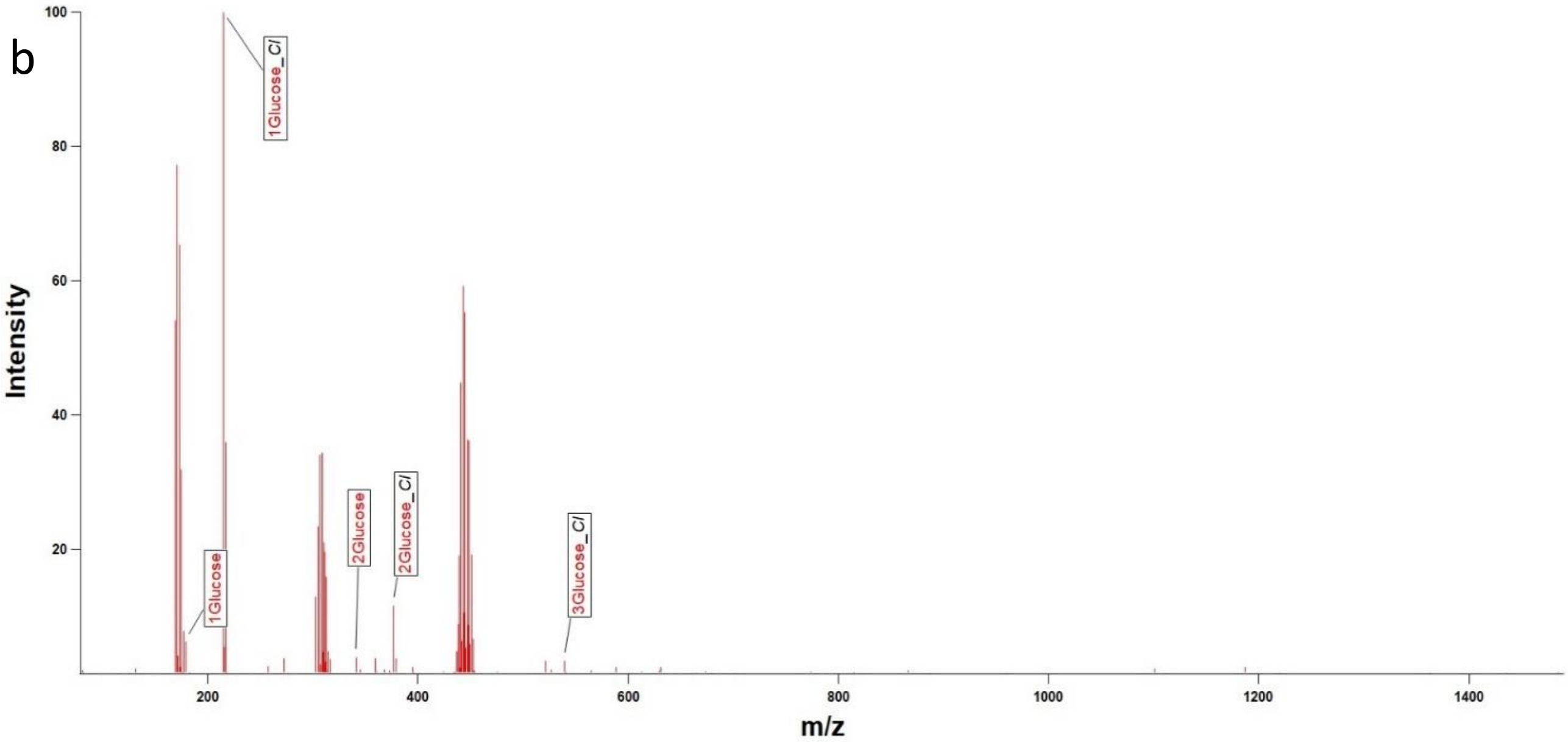

Figure S33. ESI-MS of a dry-down reaction of glucose (glu) and $ZnCl_2$ upon addition of NaOH supports the formation of short oligosaccharides. Glu and $ZnCl_2$ were dried at 50˚C for seven days and the resulting oligosaccharides were analyzed by positive mode ESI-MS (a) or negative-mode ESI-MS (b), indicating a small variety of oligosaccharides. Labeled species correspond to $[M + Zn]^{2+}$ or $[M + Na]^{+}$ ions (annotations with a hyphen indicate non-covalent complex) (a) or $[M - H]^{-}$ or $[M + Cl]^{-}$ ions (b).

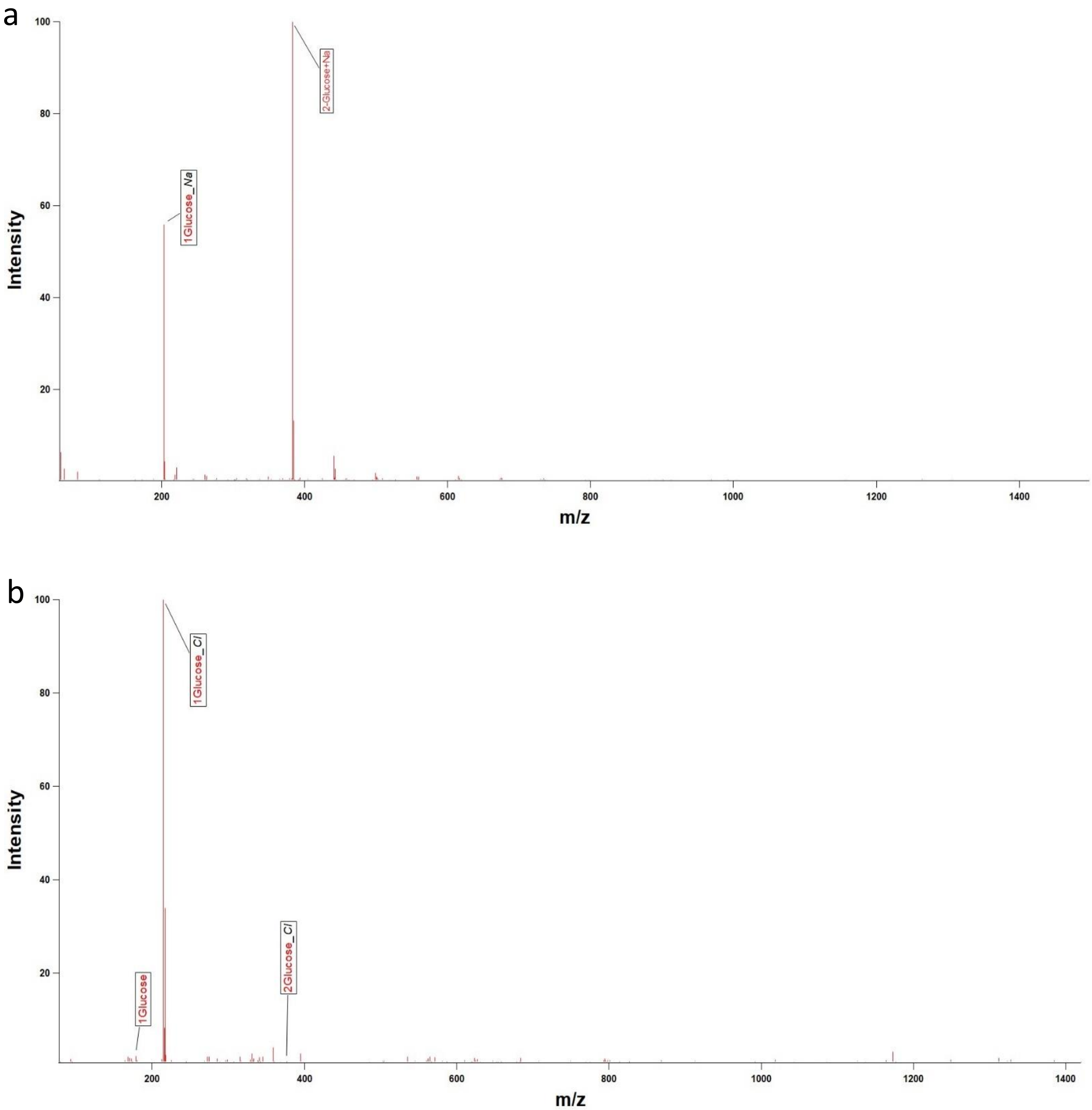

Figure S34. ESI-MS of a dry-down reaction of glucose (glu) and NaCl upon addition of NaOH**.** Glu and NaCl were dried at 50˚C for seven days and the resulting oligosaccharides were analyzed by positive mode ESI-MS (a) or negative-mode ESI-MS (b). Labeled species correspond to $[M + Na]^{+}$ ions (annotations with a hyphen indicate non-covalent complex) (a) or $[M - H]^{-}$ or $[M + Cl]^{-}$ ions (b).

a

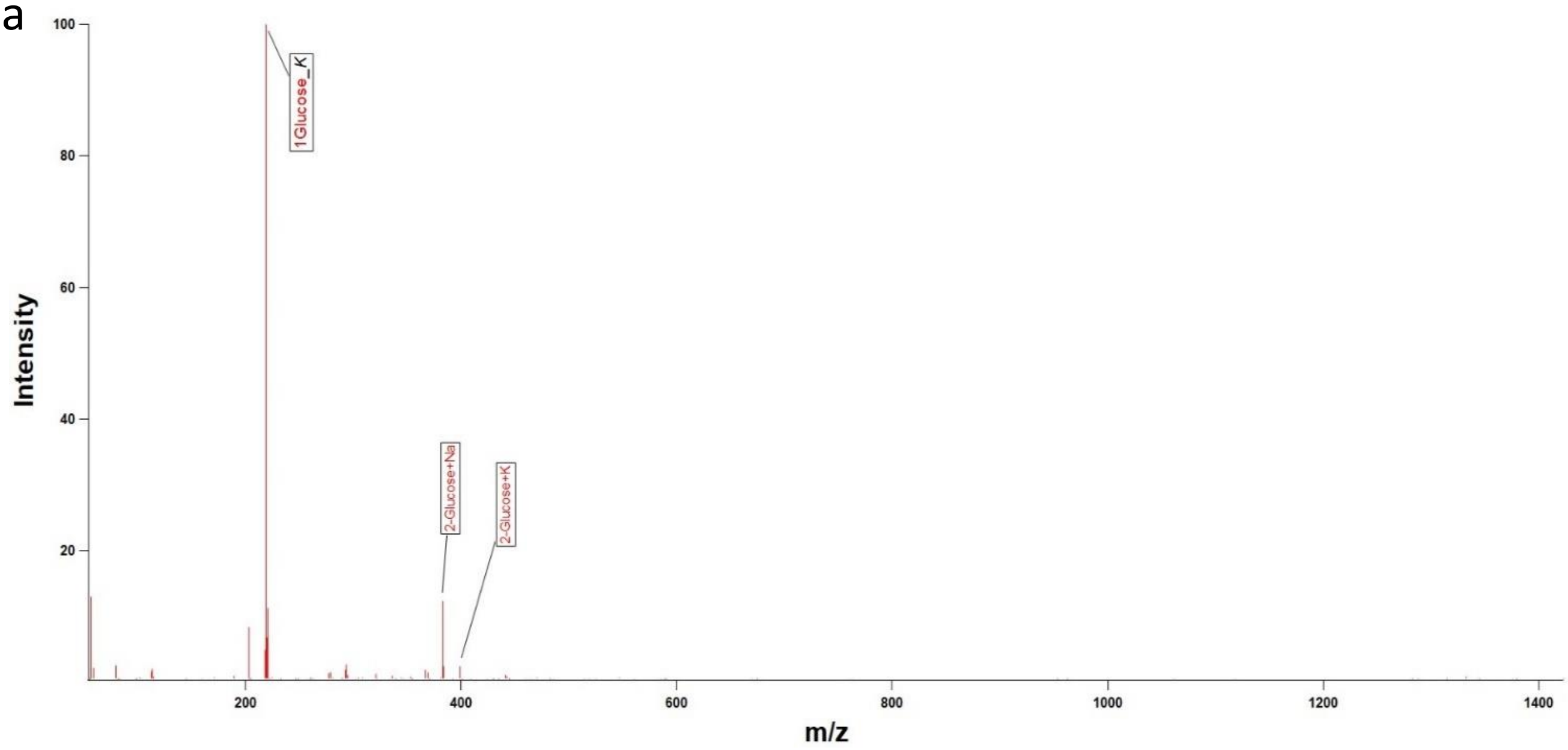

b

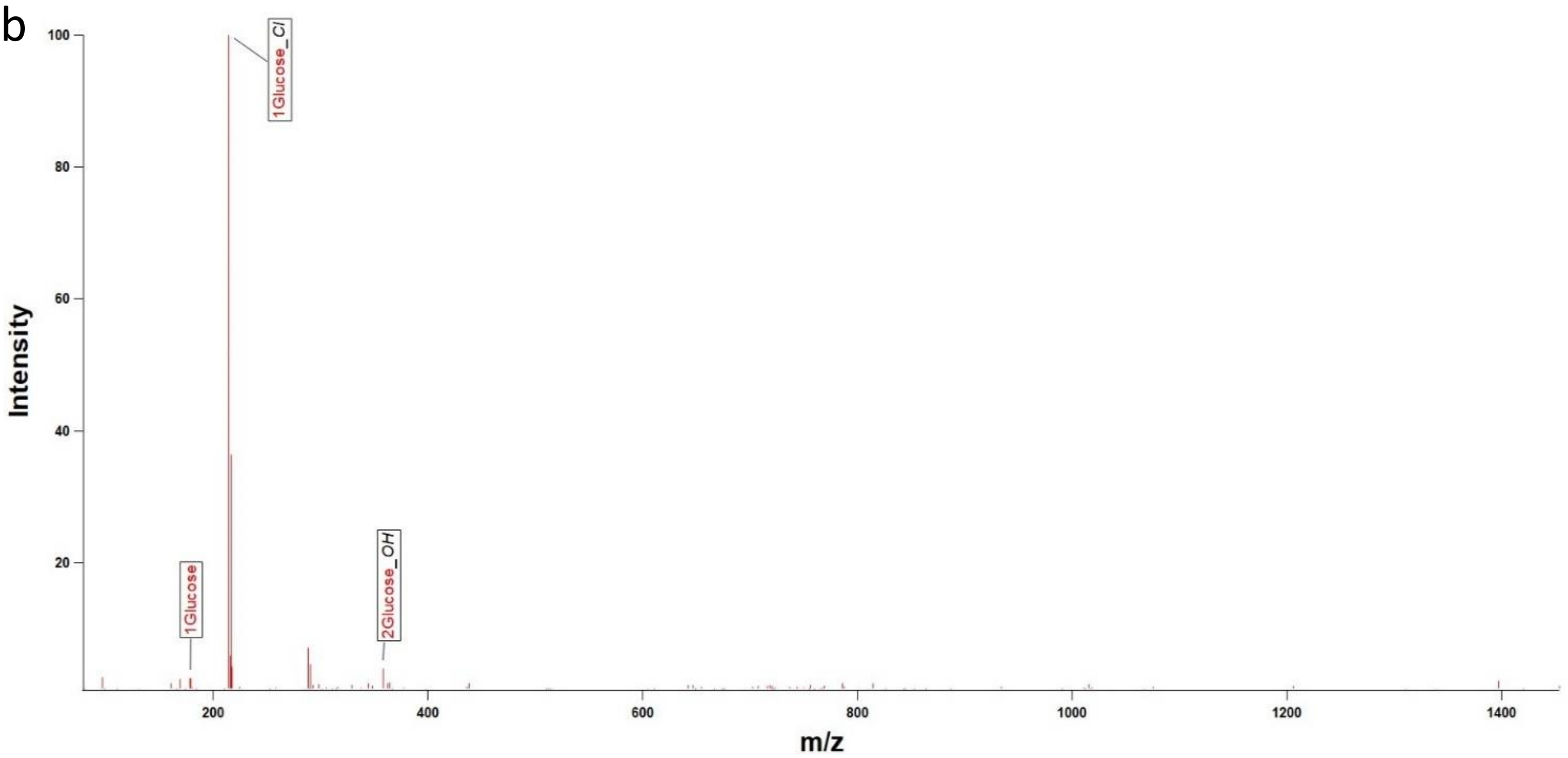

Figure S35. ESI-MS of a dry-down reaction of glucose (glu) and KCl upon addition of NaOH**.** Glu and KCl were dried at 50˚C for seven days and the resulting oligosaccharides were analyzed by positive mode ESI-MS (a) or negative-mode ESI-MS (b). Labeled species correspond to $[M+K]^+$ or $[M+Na]^+$ ions(annotations with a hyphen indicate non-covalent complex) (a) or $[M-H]^-$ or $[M+Cl]^-$ or $[M+OH]^-$ ions (b).

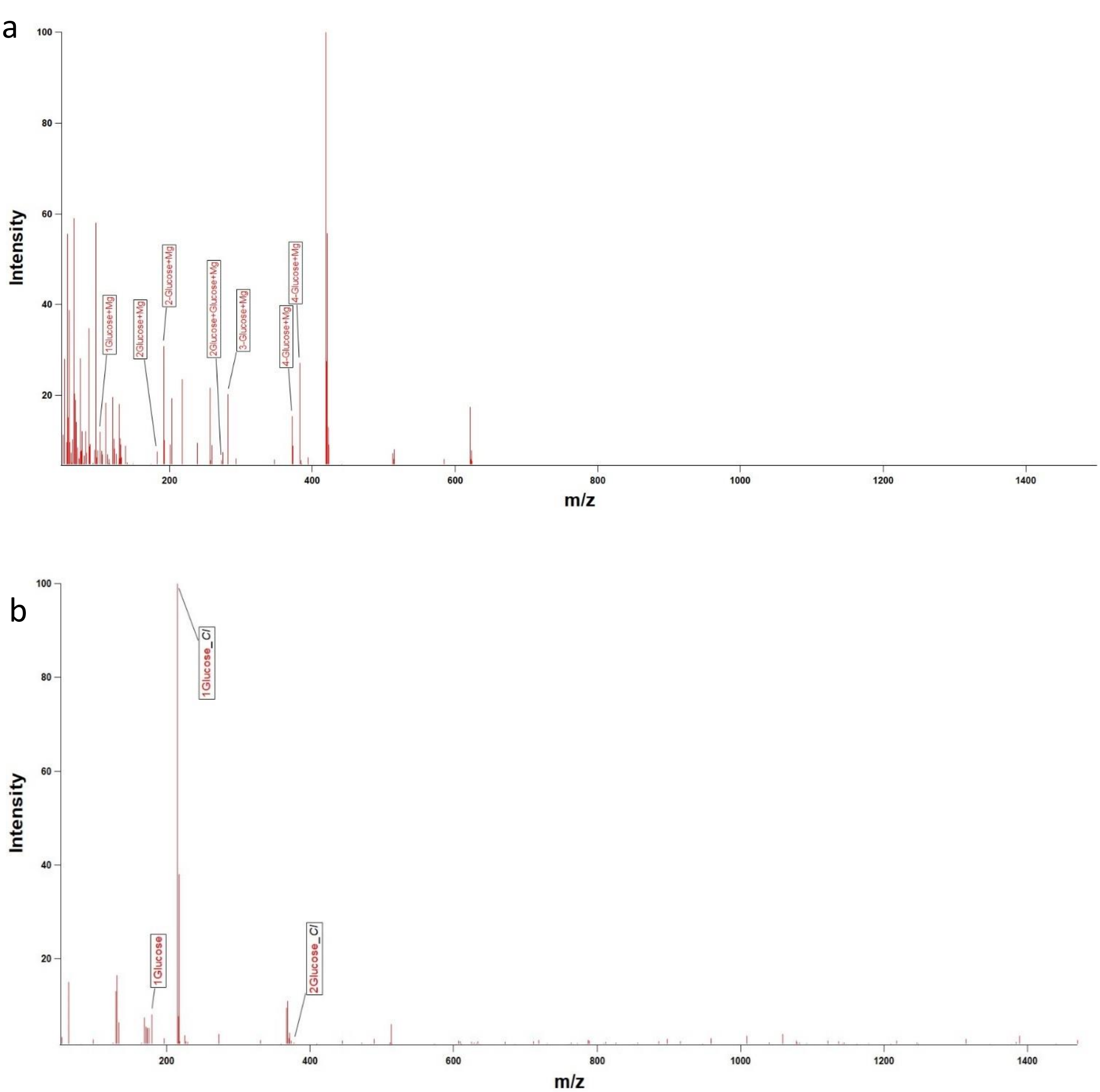

Figure S36. ESI-MS of a dry-down reaction of glucose (glu) and $MgCl_2$ upon addition of NaOH**.** Glu and $MgCl_2$ were dried at 50˚C for seven days and the resulting oligosaccharides were analyzed by positive mode ESI-MS (a) or negative-mode ESI-MS (b). Labeled species correspond to $[M + Mg]^{2+}$ ions (annotations with a hyphen indicate non-covalent complex) (a) or $[M - H]^-$ or $[M + Cl]^-$ ions (b).

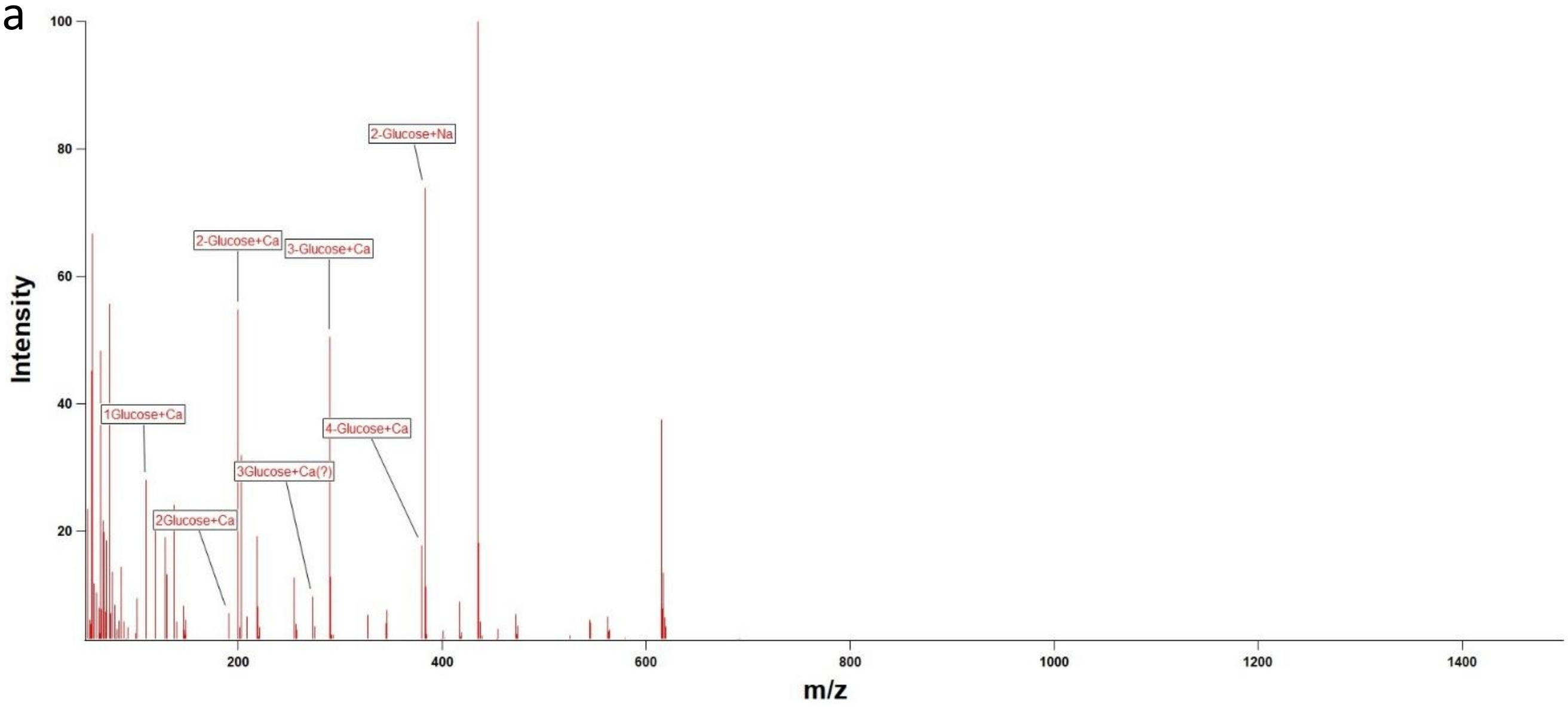

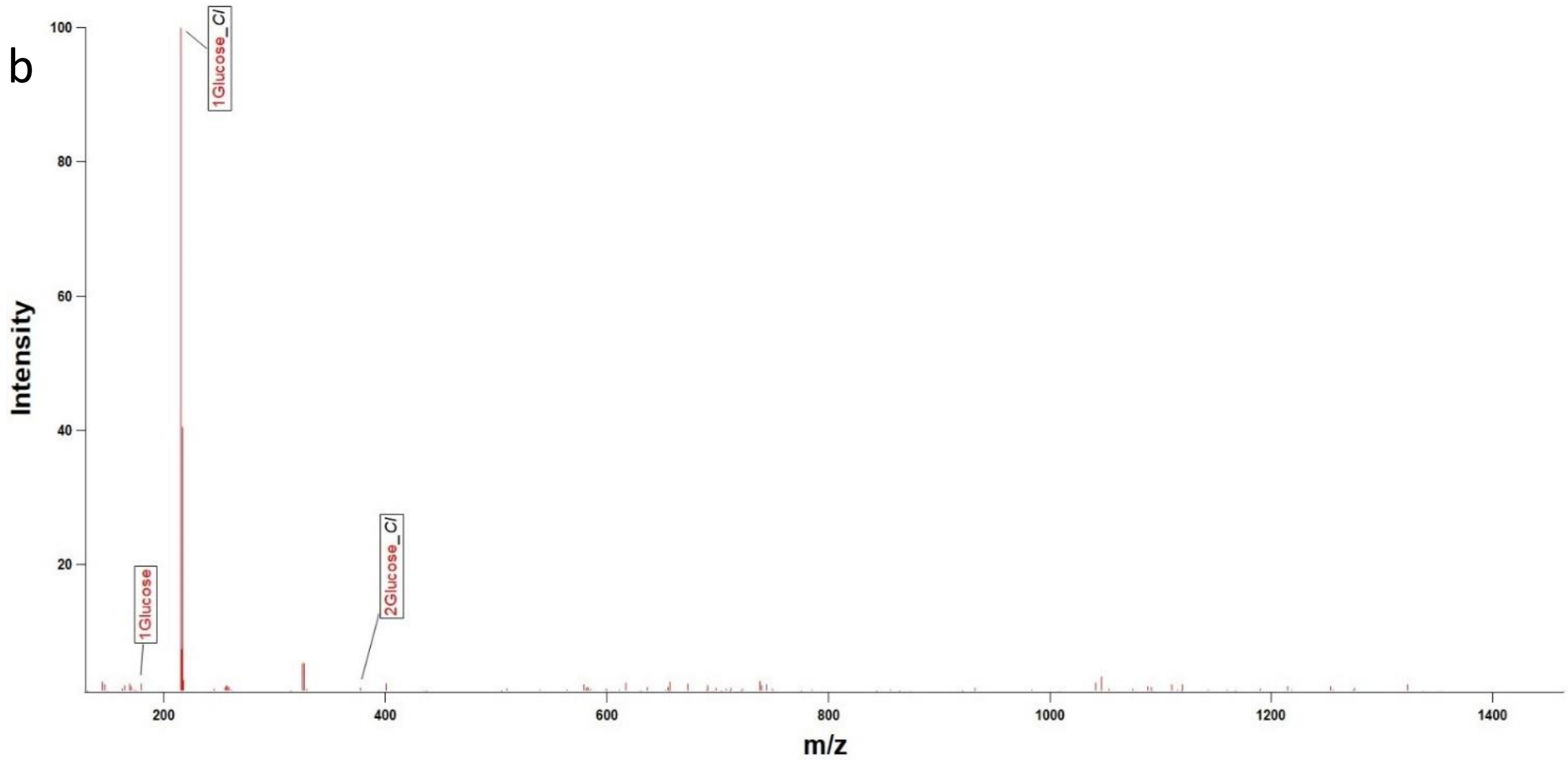

Figure S37. ESI-MS of a dry-down reaction of glucose (glu) and $CaCl_2$ upon addition of NaOH**.** Glu and $CaCl_2$ were dried at 50˚C for seven days and the resulting oligosaccharides were analyzed by positive mode ESI-MS (a) or negative-mode ESI-MS (b), indicating a variety of oligosaccharides. Labeled species correspond to $[M + Ca]^{2+}$ or $[M + Na]^{+}$ ions (annotations with a hyphen indicate non-covalent complex) (a) or $[M - H]^{-}$ or $[M + Cl]^{-}$ ions (b).

a

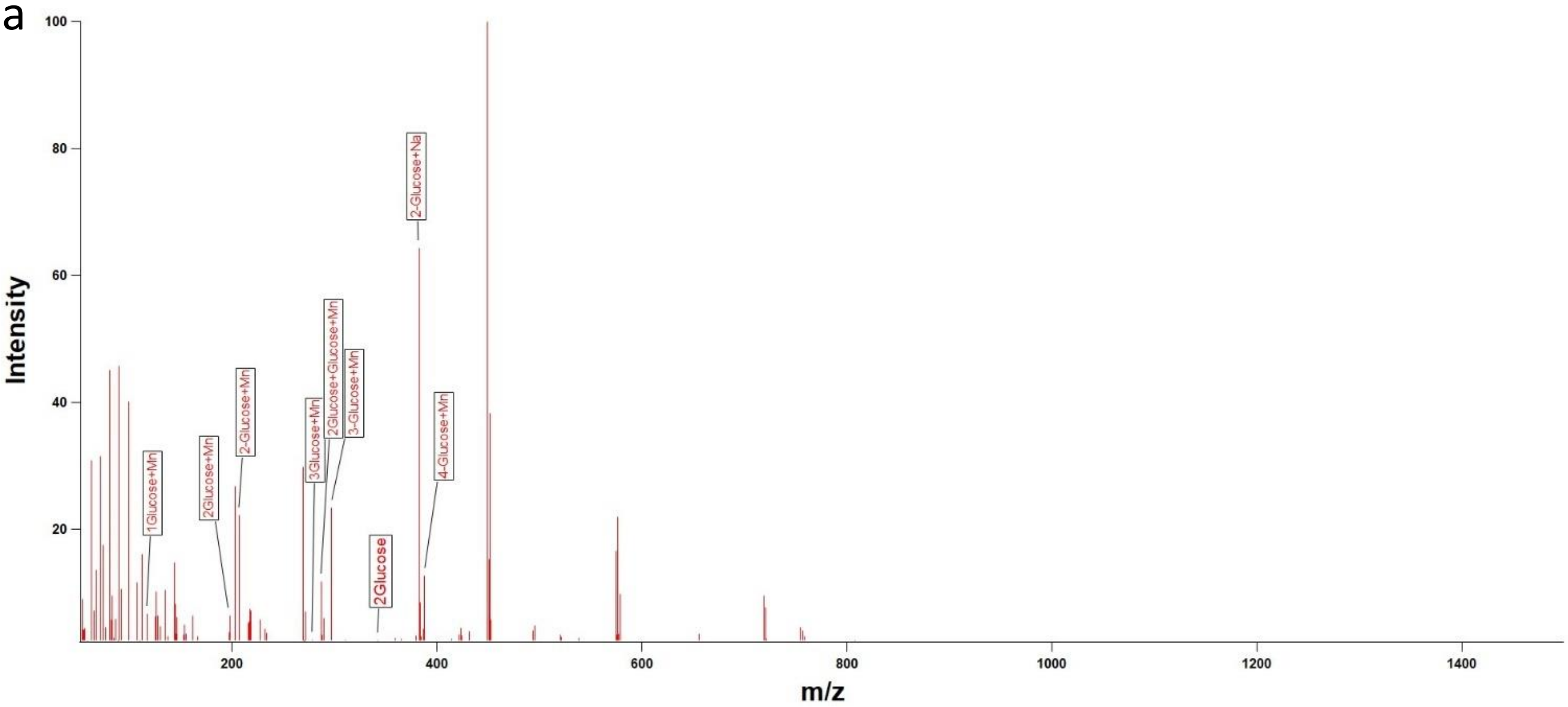

b

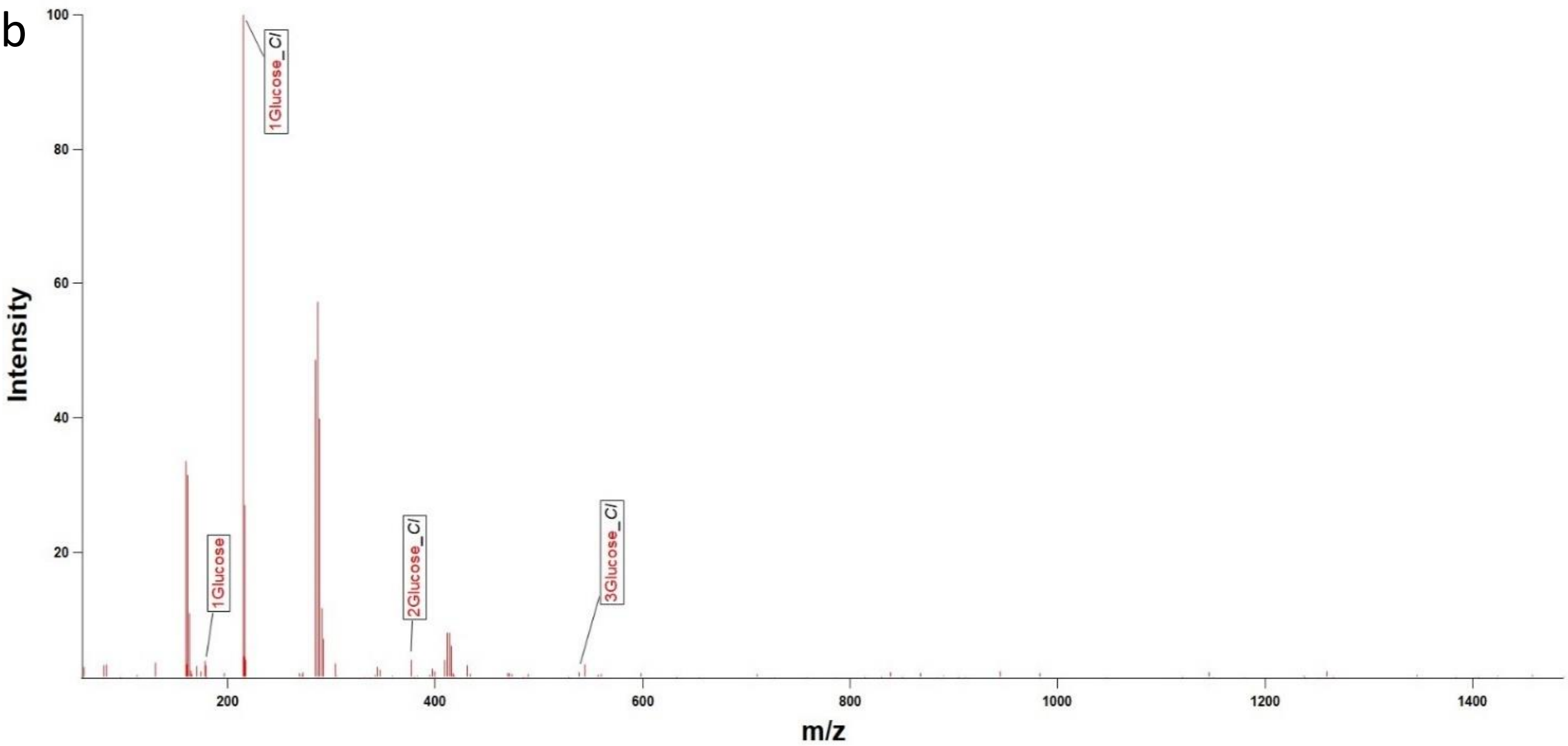

Figure S38. ESI-MS of a dry-down reaction of glucose (glu) and $MnCl_2$ upon addition of NaOH supports the formation of short oligosaccharides. Glu and $MnCl_2$ were dried at 50˚C for seven days and the resulting oligosaccharides were analyzed by positive mode ESI-MS (a) or negative-mode ESI-MS (b), indicating a small variety of oligosaccharides. Labeled species correspond to $[M+H]^{+}$ or $[M+Mn]^{2+}$ or $[M+Na]^{+}$ ions (annotations with a hyphen indicate non-covalent complex) (a) or $[M-H]^{-}$ or $[M+Cl]^{-}$ ions (b).

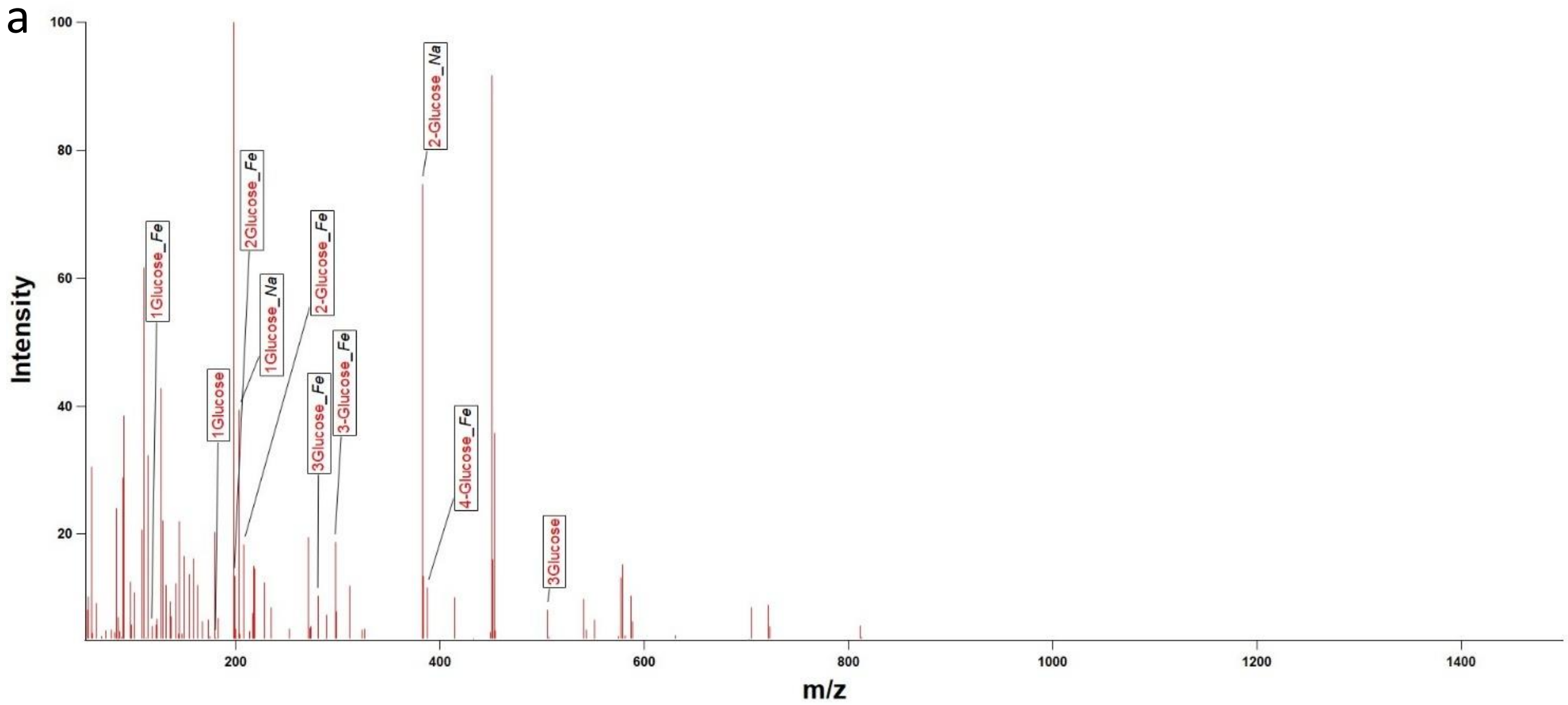

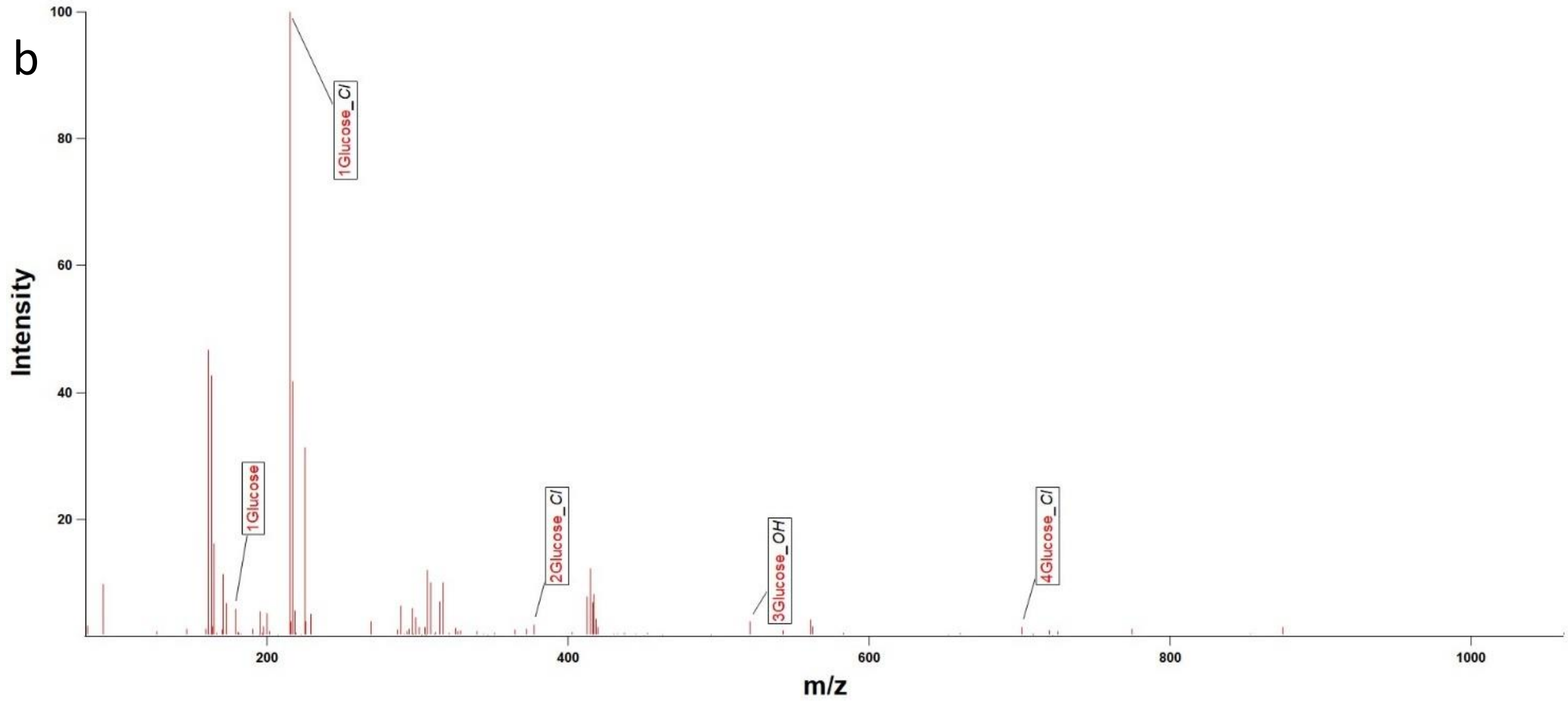

Figure S39. ESI-MS of a dry-down reaction of glucose (glu) and $FeCl_2$ upon addition of NaOH supports the formation of short oligosaccharides. Glu and $FeCl_2$ were dried at 50˚C for seven days and the resulting oligosaccharides were analyzed by positive mode ESI-MS (a) or negative-mode ESI-MS (b), indicating a variety of oligosaccharides. Labeled species correspond to $[M+H]^+$ or $[M+Fe]^{2+}$ or $[M+Na]^+$ ions (annotations with a hyphen indicate non-covalent complex) (a) or $[M-H]^-$ or $[M+Cl]^-$ ions (b).

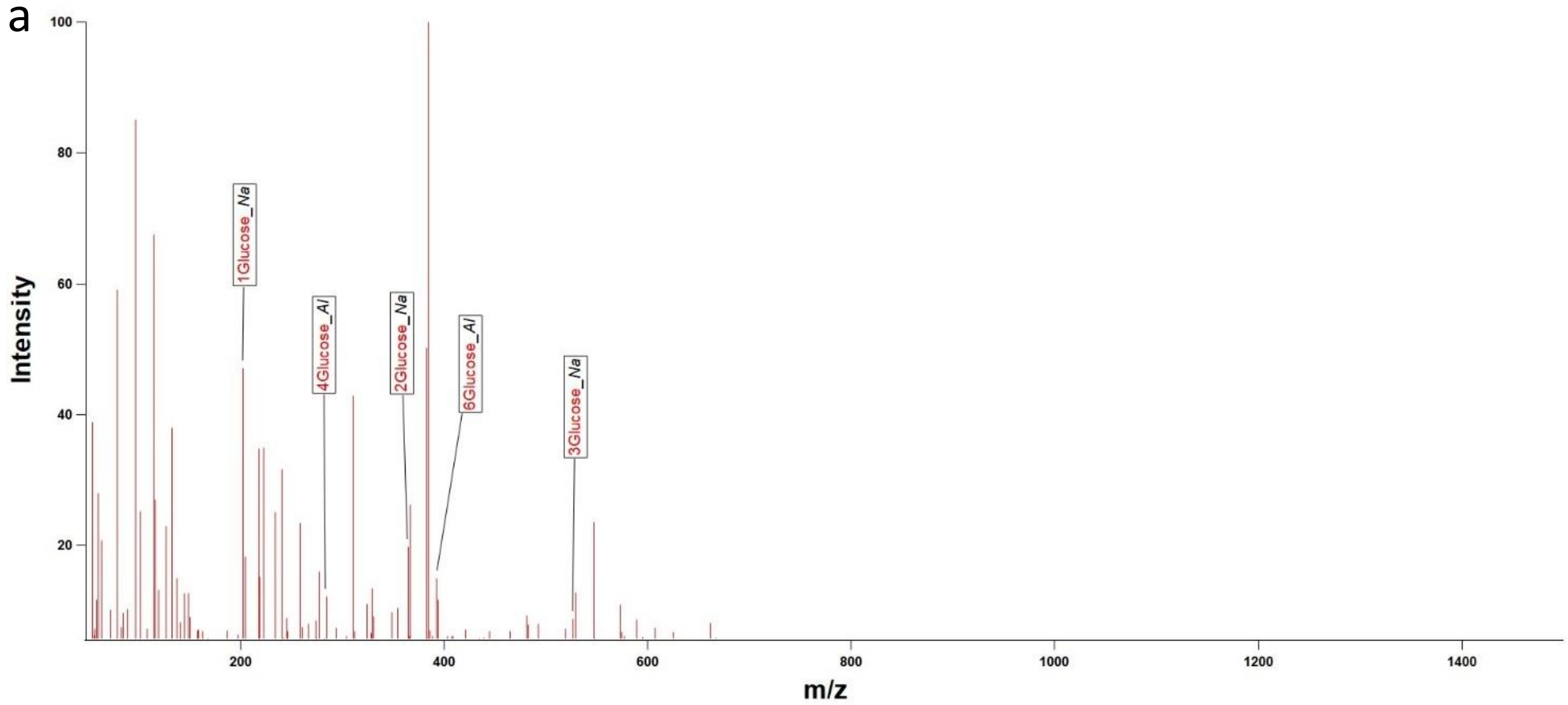

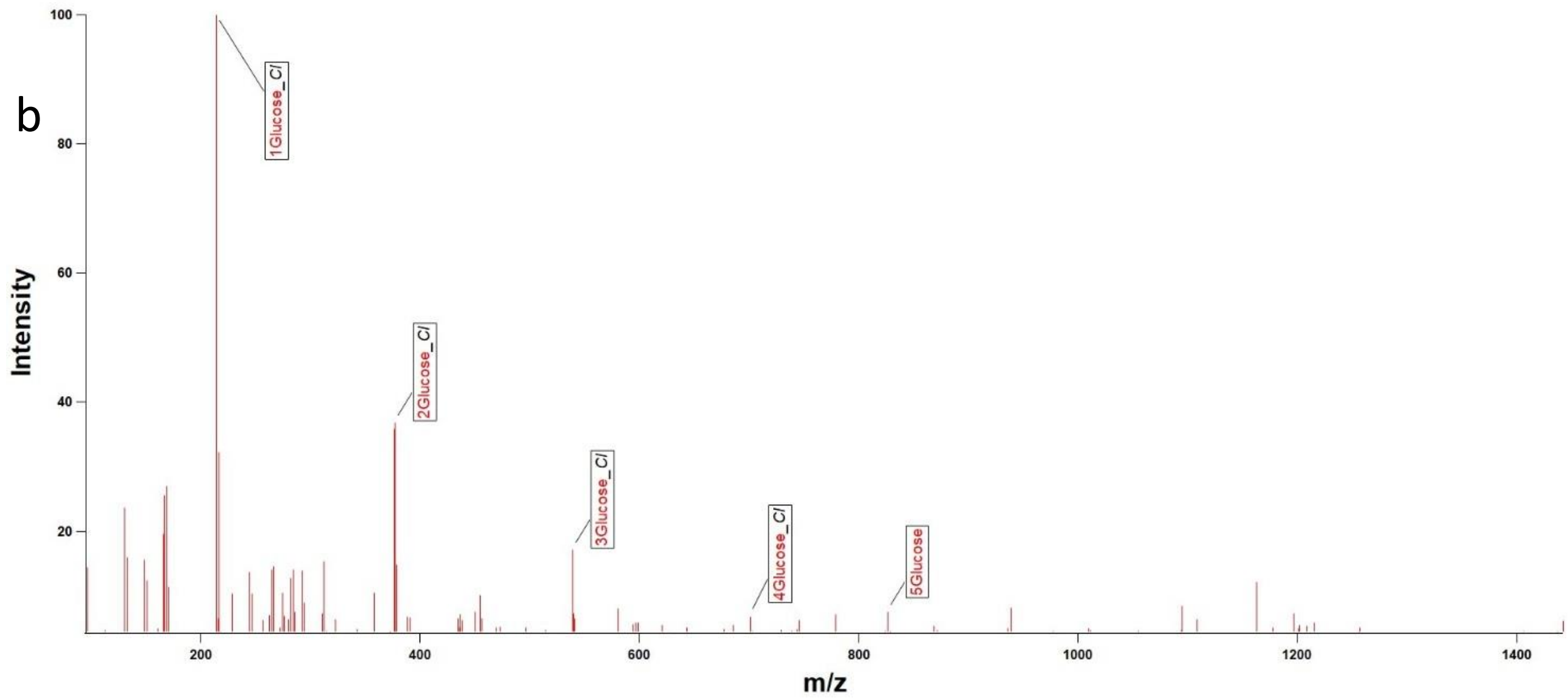

Figure S40. ESI-MS of a dry-down reaction of glucose (glu) and $AlCl_3$ upon addition of NaOH supports the formation of short oligosaccharides. Glu and $AlCl_3$ were dried at 50˚C for seven days and the resulting oligosaccharides were analyzed by positive mode ESI-MS (a) or negative-mode ESI-MS (b), indicating a variety of oligosaccharides. Labeled species correspond to $[M + Al]^{3+}$ or $[M + Na]^{+}$ ions (a) or $[M - H]^{-}$ or $[M + Cl]^{-}$ ions (b).

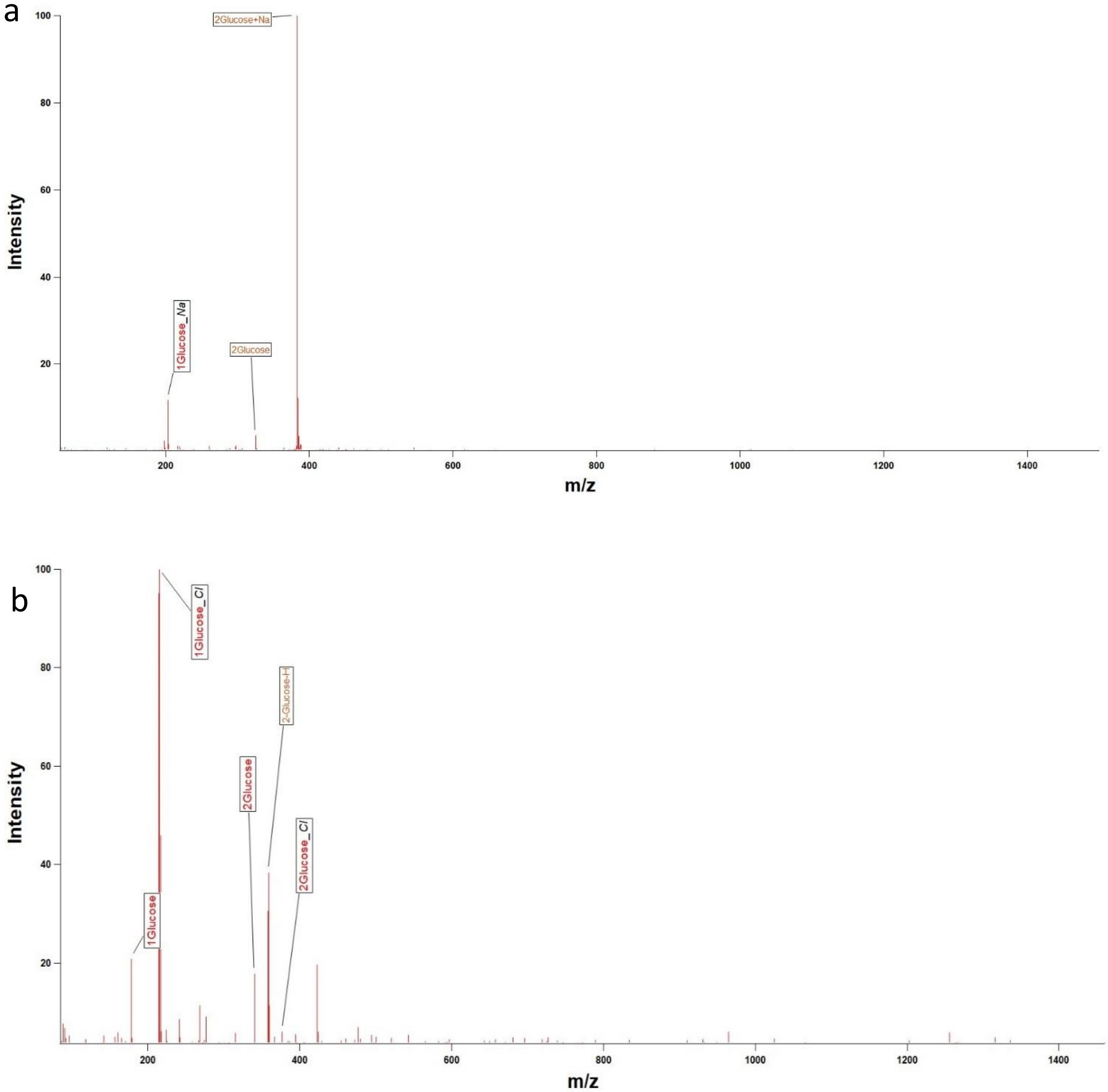

Figure S41. ESI-MS of a fresh solution of glucose (glu) in an acidified environment**.** Glu was analyzed by positive mode ESI-MS (a) or negative-mode ESI-MS (b). Labeled species correspond to $[M+H]^{+}$ or $[M+Na]^{+}$ions (a) or $[M-H]^{-}$ or $[M+Cl]^{-}$ ions (b).

a

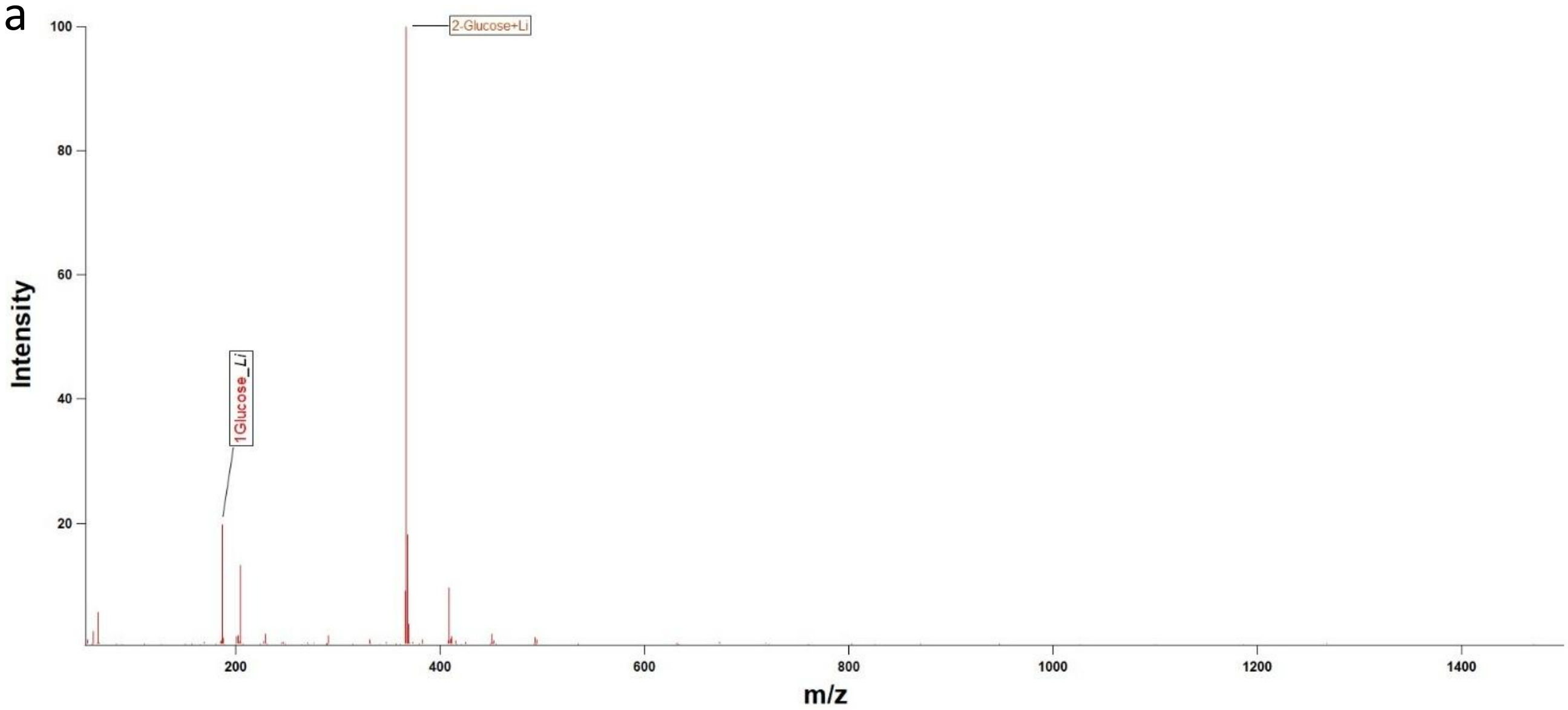

b

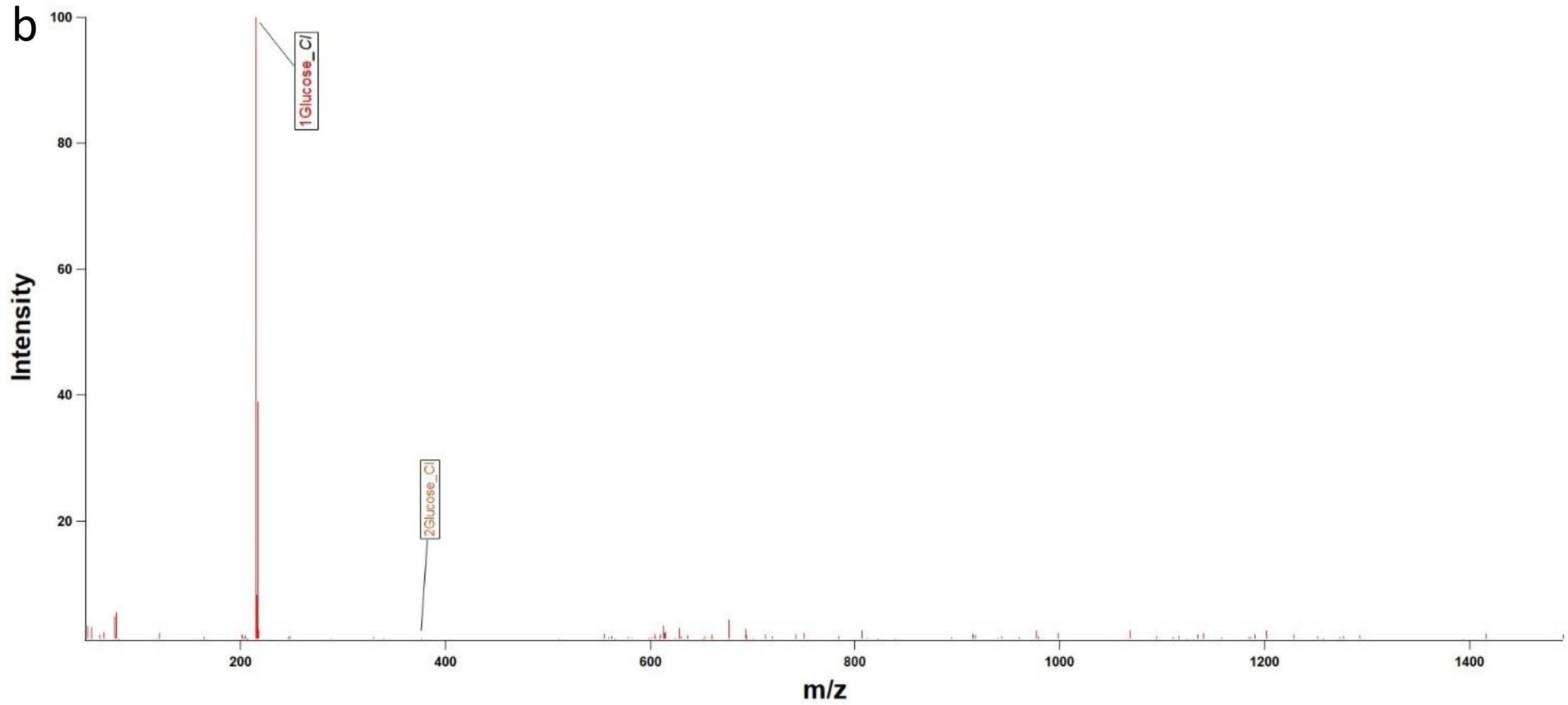

Figure S42. ESI-MS of a fresh solution of glucose (glu) and LiCl in an acidified environment**.** Glu and LiCl were analyzed by positive mode ESI-MS (a) or negative-mode ESI-MS (b). Labeled species correspond to $[M + Li]^+$ ions (annotations with a hyphen indicate non-covalent complex) (a) or $[M + Cl]^-$ ions (b).

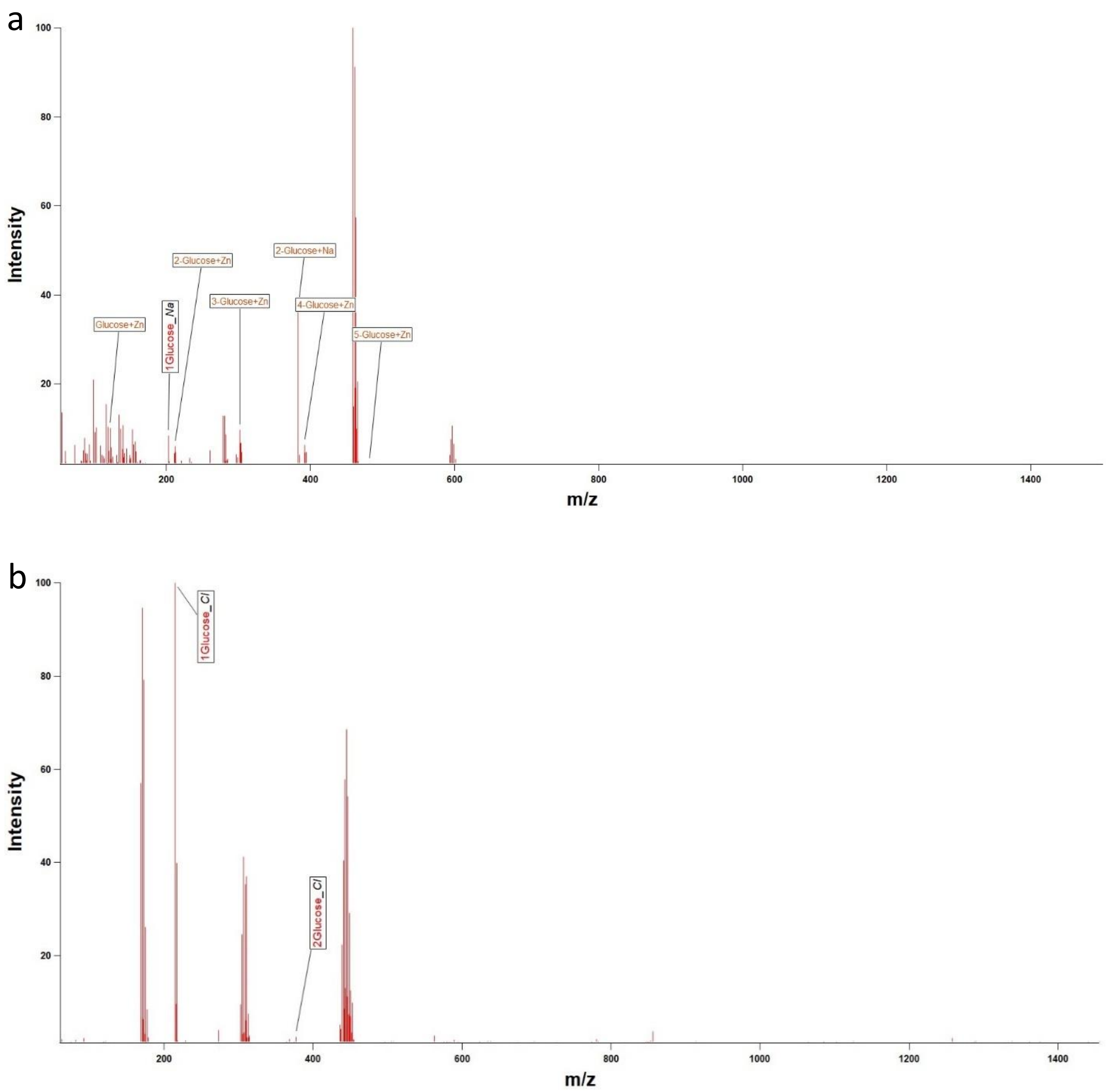

Figure S43. ESI-MS of a fresh solution of glucose (glu) and $ZnCl_2$ in an acidified environment**.** Glu and $ZnCl_2$ were analyzed by positive mode ESI-MS (a) or negative-mode ESI-MS (b). Labeled species correspond to $[M + Na]^{+}$ or $[M + Zn]^{2+}$ ions (annotations with a hyphen indicate non-covalent complex) (a) or $[M + Cl]^{-}$ ions (b).

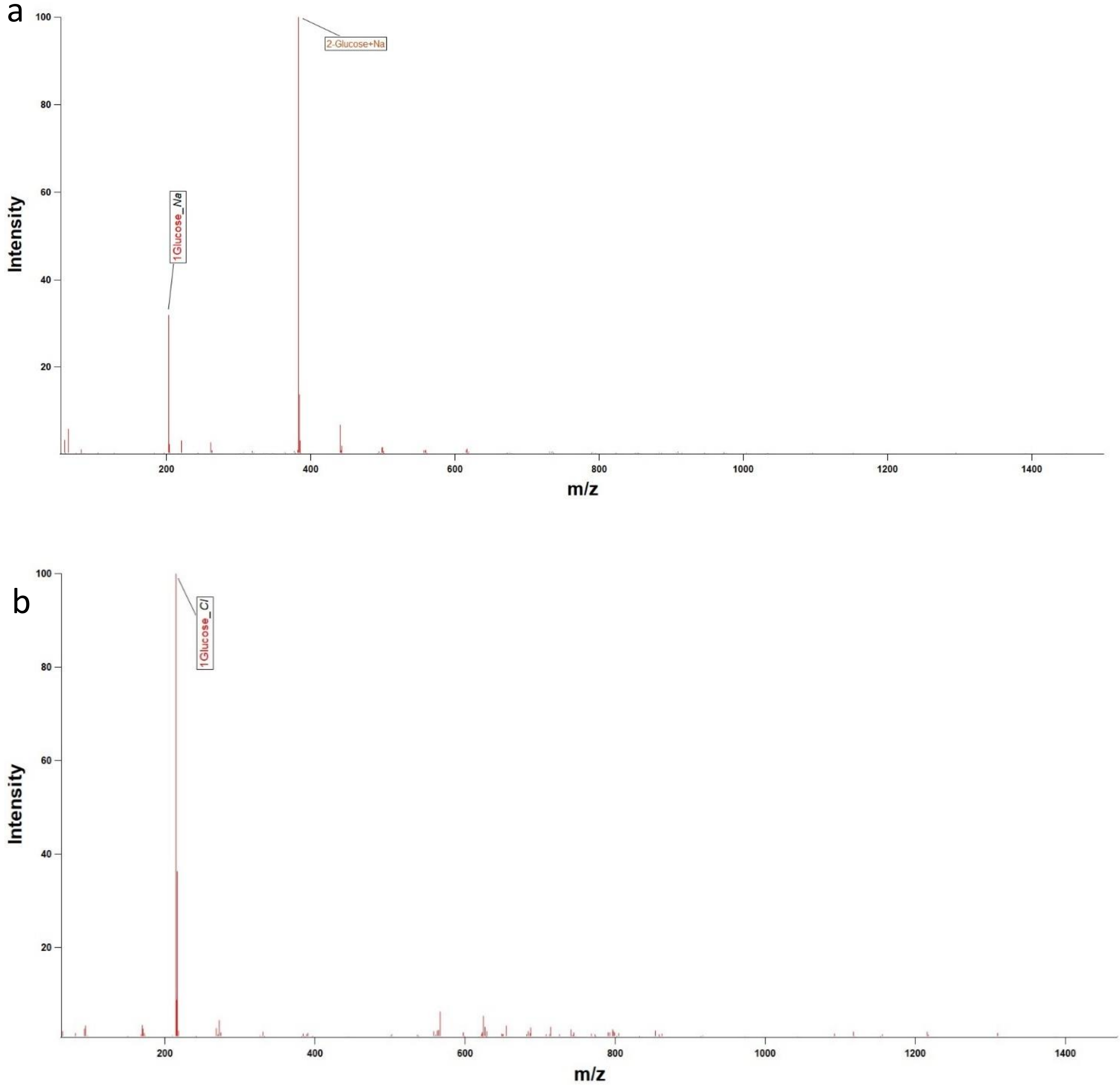

Figure S44. ESI-MS of a fresh solution of glucose (glu) and NaCl in an aacidified environment**.** Glu and NaCl were analyzed by positive mode ESI-MS (a) or negative-mode ESI-MS (b). Labeled species correspond to $[M+Na]^{+}$ ions (annotations with a hyphen indicate non-covalent complex) (a) or $[M+Cl]^{-}$ ions (b).

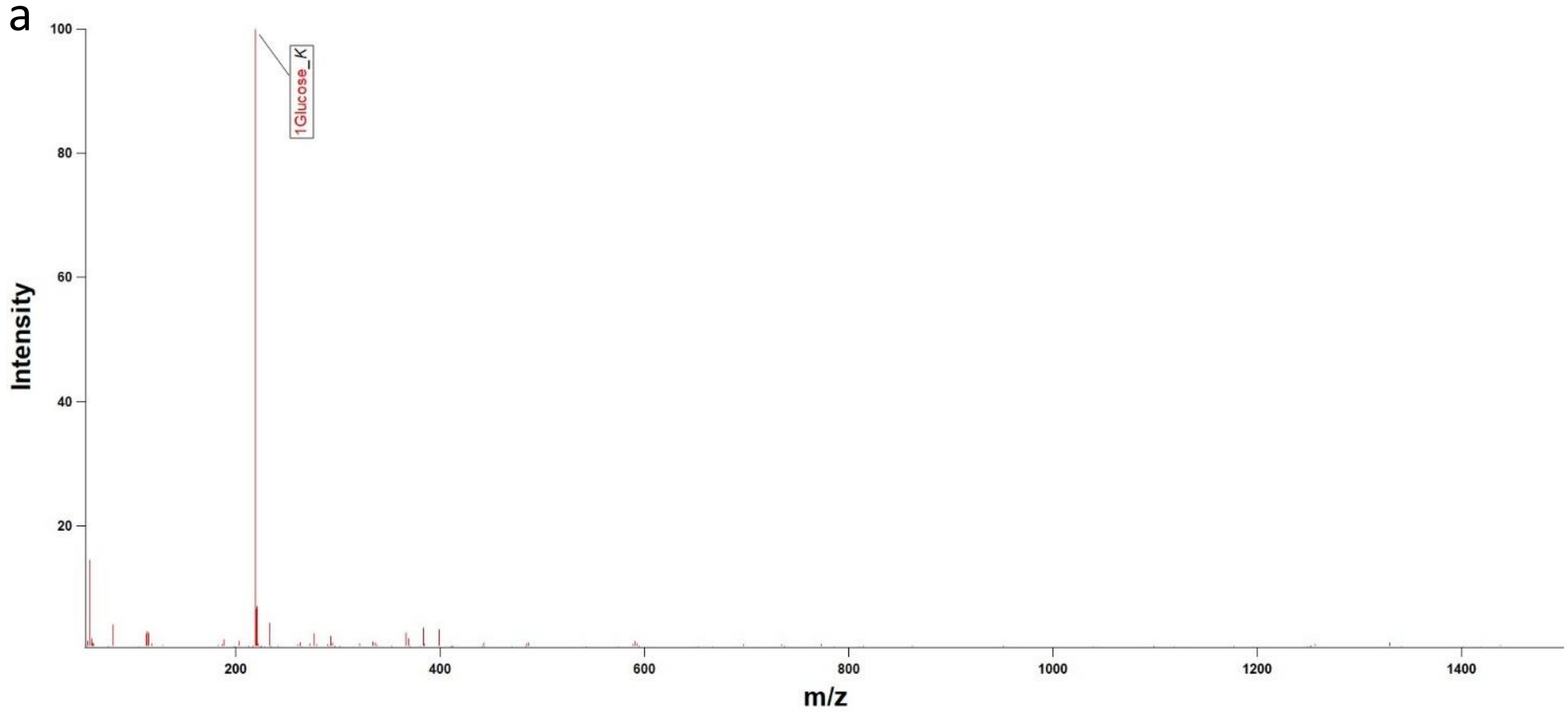

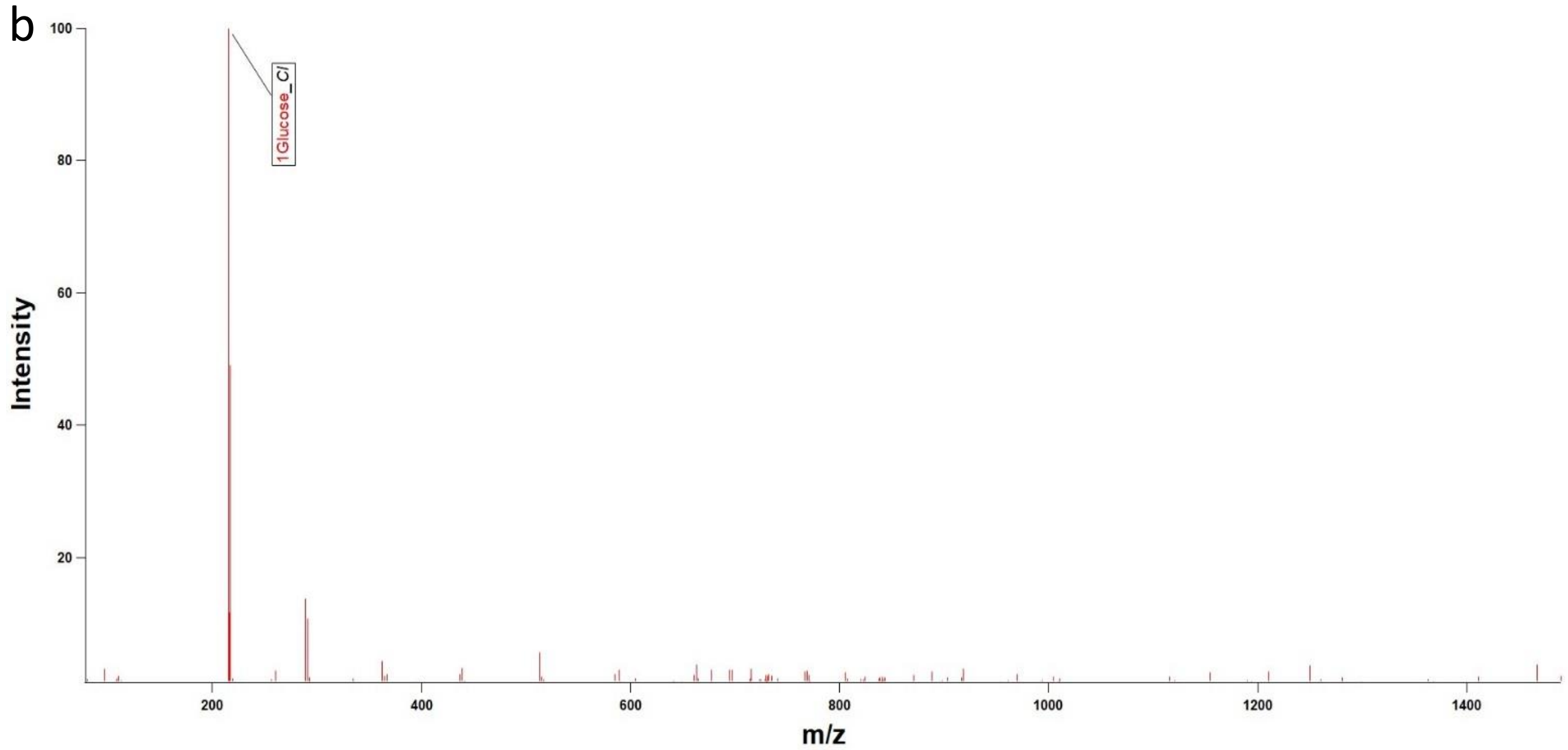

Figure S45. ESI-MS of a fresh solution of glucose (glu) and KCl in an acidified environment**.** Glu and KCl were analyzed by positive mode ESI-MS (a) or negative-mode ESI-MS (b). Labeled species correspond to $[M + K]^{+}$ ions (annotations with a hyphen indicate non-covalent complex) (a) or $[M + Cl]^{-}$ ions (b).

a

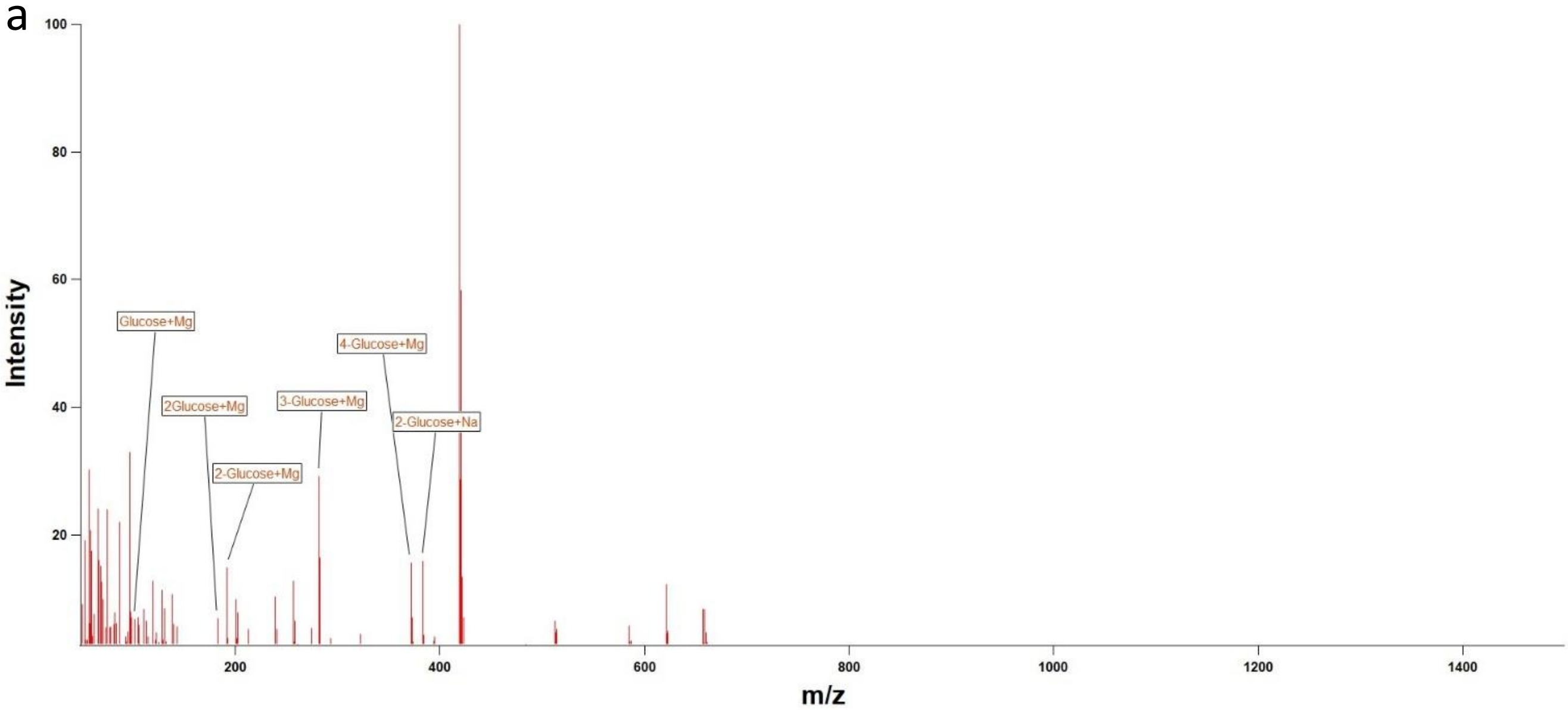

b

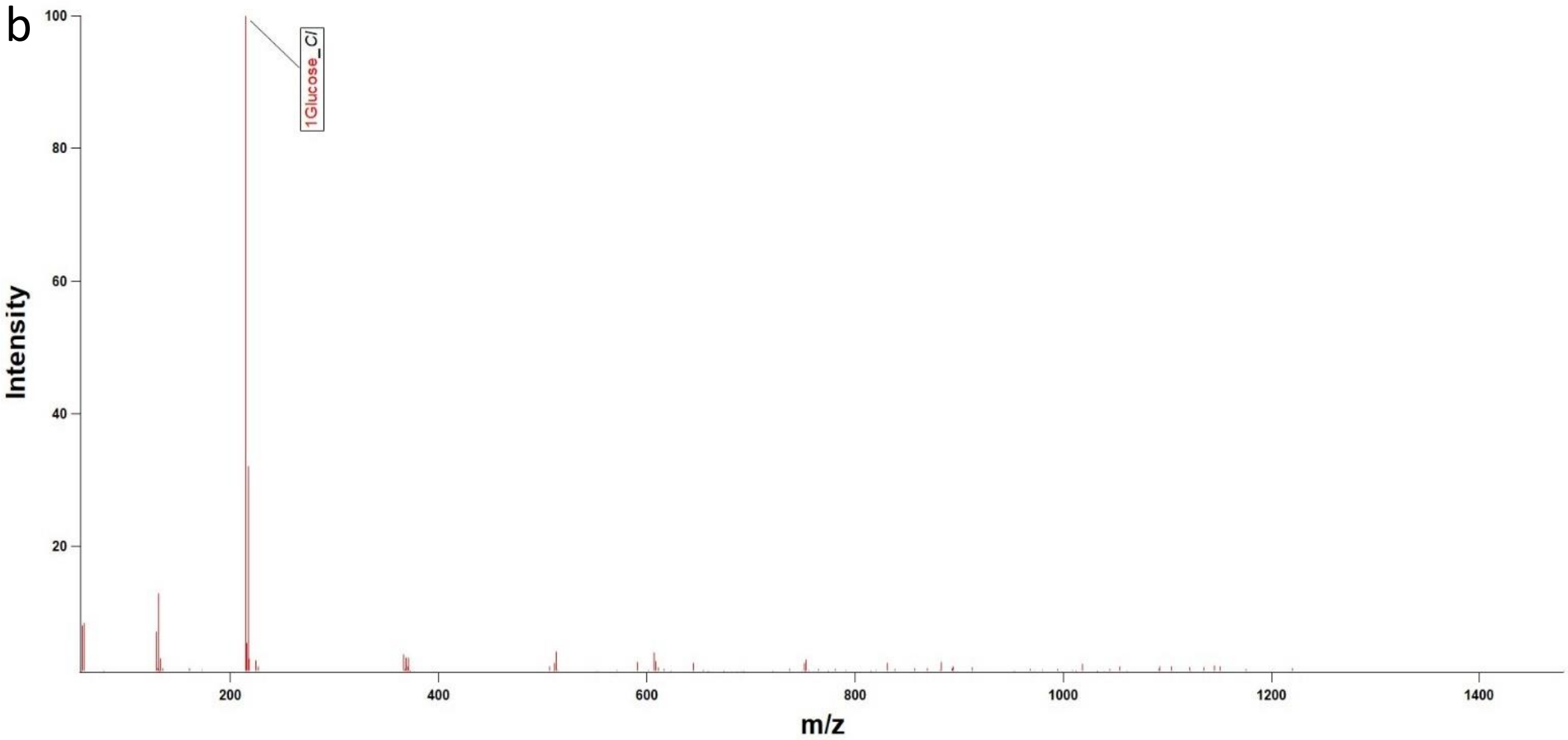

Figure S46. ESI-MS of a fresh solution of glucose (glu) and $MgCl_2$ in an acidified environment. Glu and $MgCl_2$ were analyzed by positive mode ESI-MS (a) or negative-mode ESI-MS (b). Labeled species correspond to $[M + Na]^{+}$ or $[M + Mg]^{2+}$ ions (annotations with a hyphen indicate non-covalent complex) (a) or $[M + Cl]^{-}$ ions (b).

a

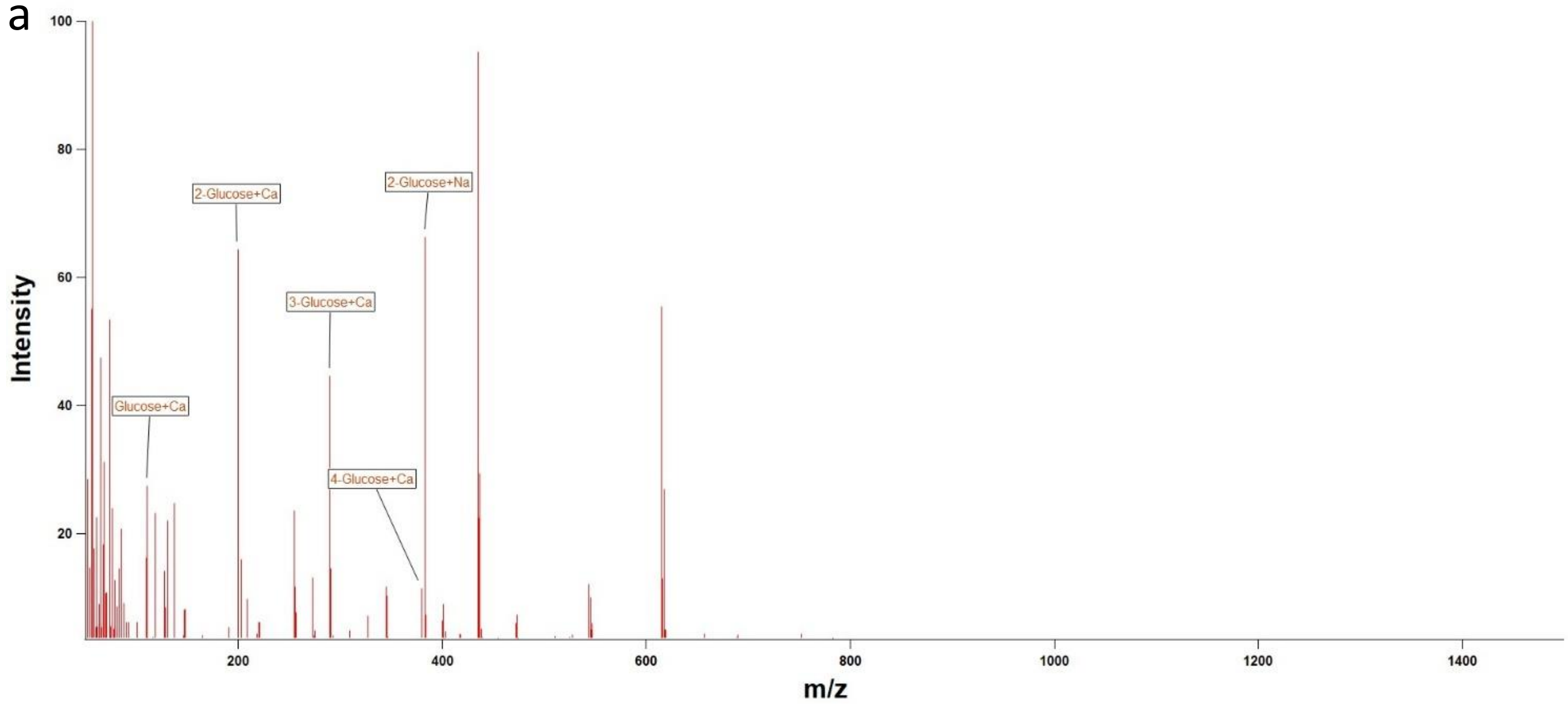

b

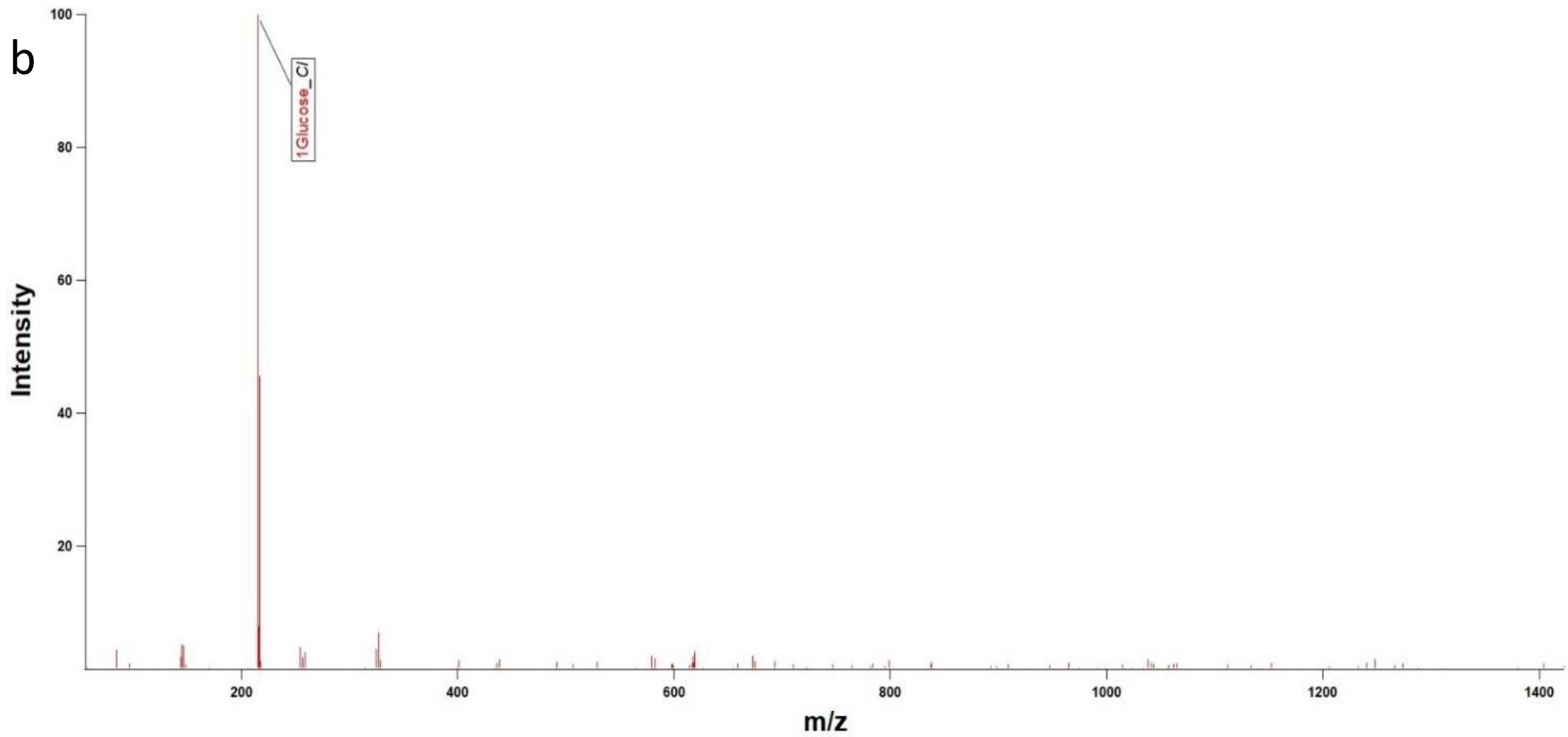

Figure S47. ESI-MS of a fresh solution of glucose (glu) and $CaCl_2$ in an acidified environment**.** Glu and $CaCl_2$ were analyzed by positive mode ESI-MS (a) or negative-mode ESI-MS (b). Labeled species correspond to $[M + Na]^{+}$ or $[M + Ca]^{2+}$ ions (annotations with a hyphen indicate non-covalent complex) (a) or $[M + Cl]^{-}$ ions (b).

a

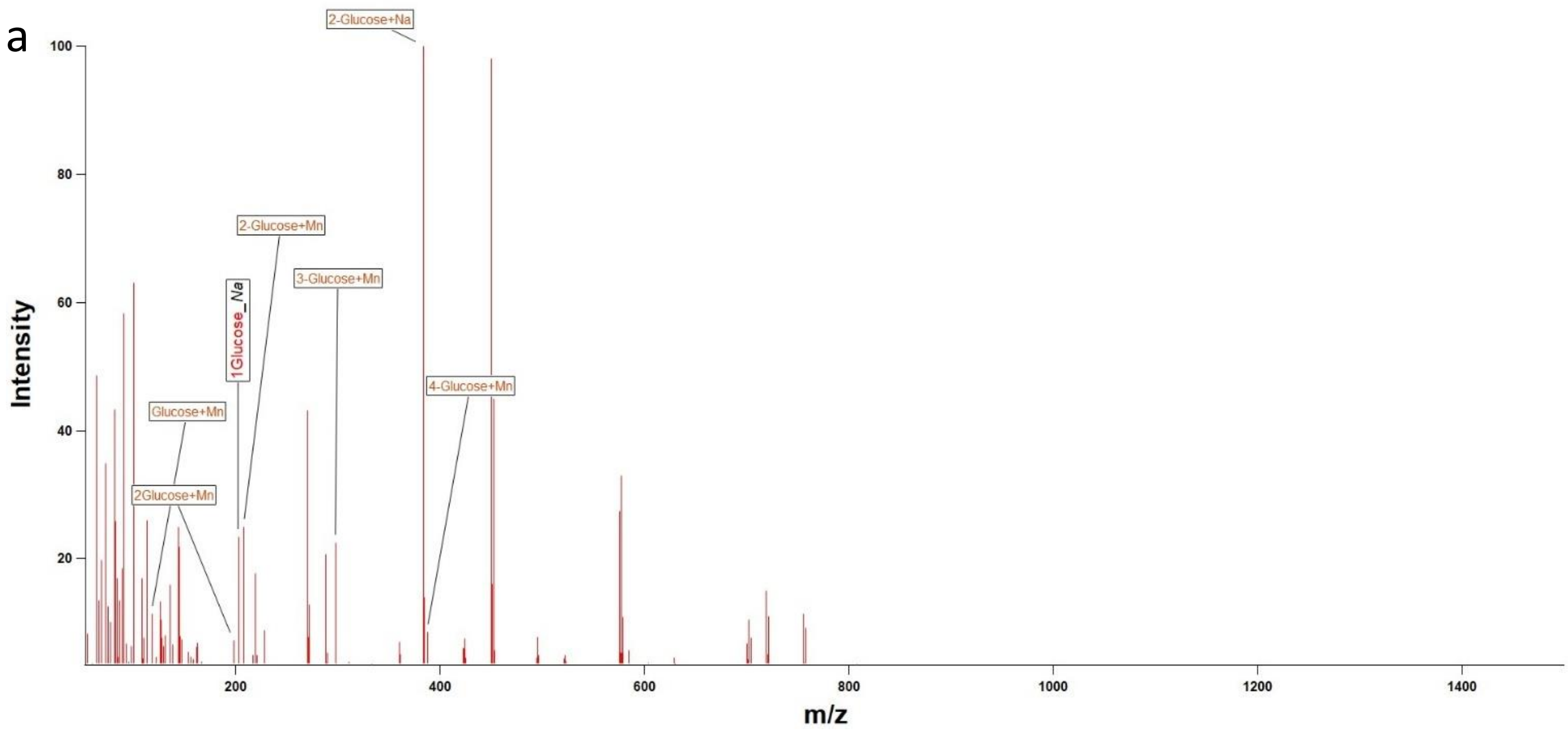

b

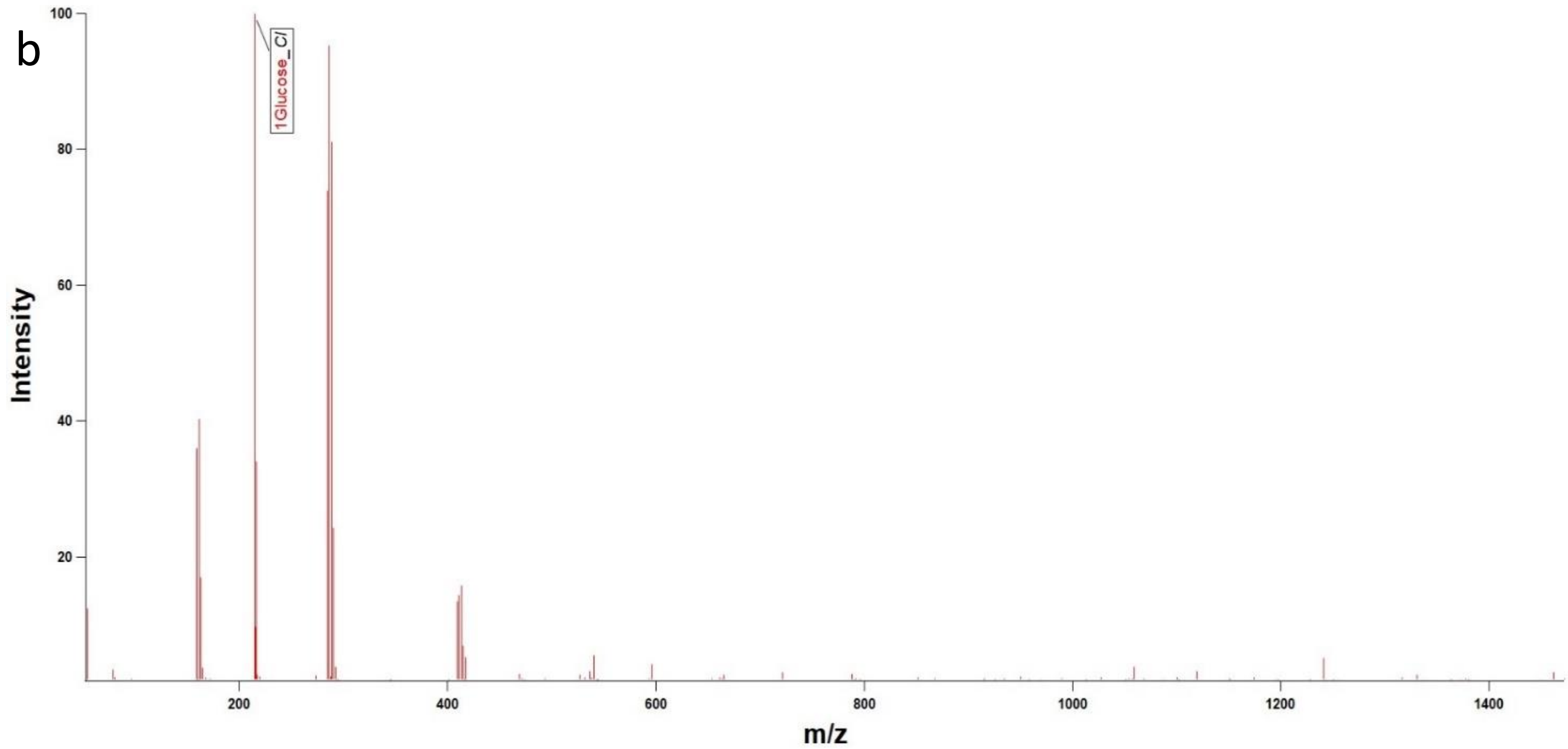

Figure S48. ESI-MS of a fresh solution of glucose (glu) and $MnCl_2$ in an acidified environment**.** Glu and $MnCl_2$ were analyzed by positive mode ESI-MS (a) or negative-mode ESI-MS (b). Labeled species correspond to $[M + Na]^{+}$ or $[M + Mn]^{2+}$ ions (annotations with a hyphen indicate non-covalent complex) (a) or $[M + Cl]^{-}$ ions (b).

a

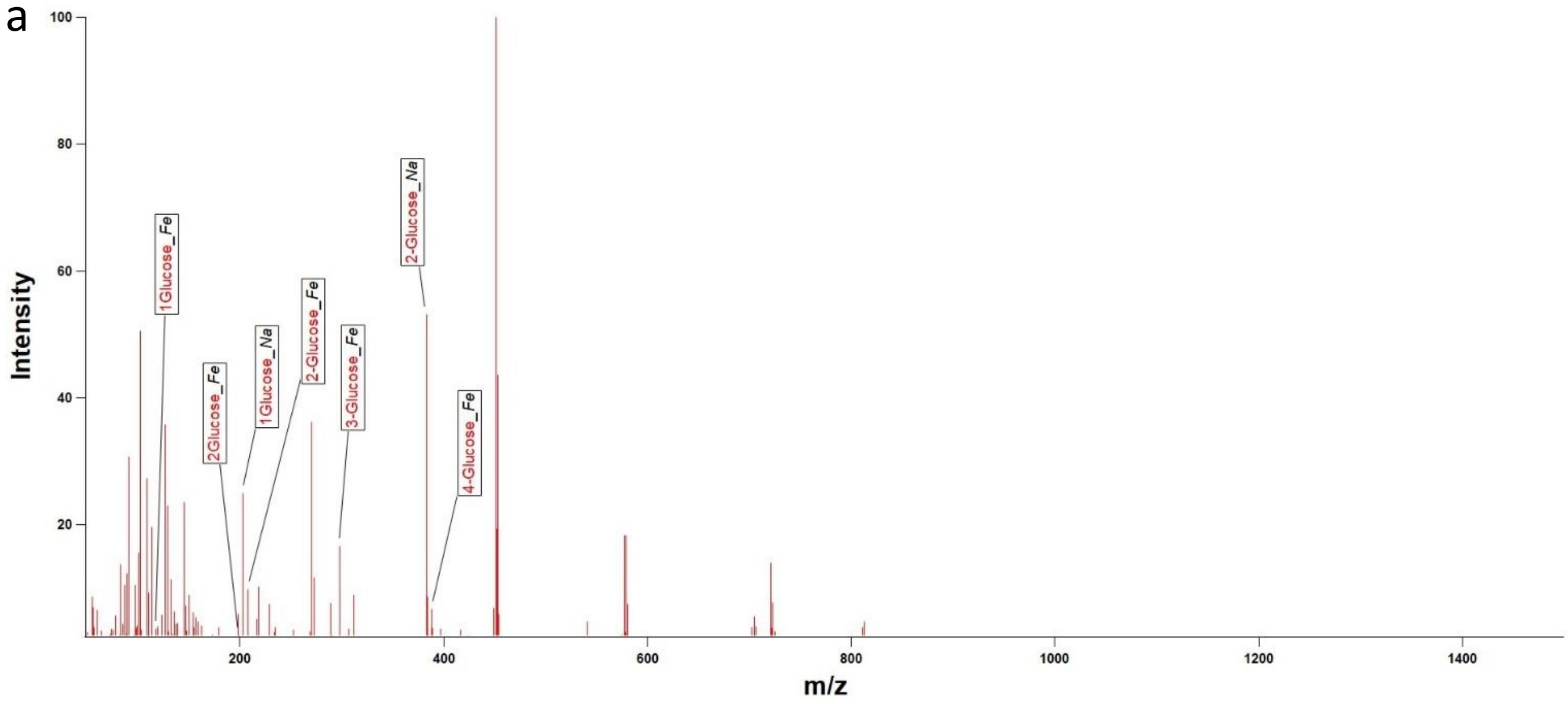

b

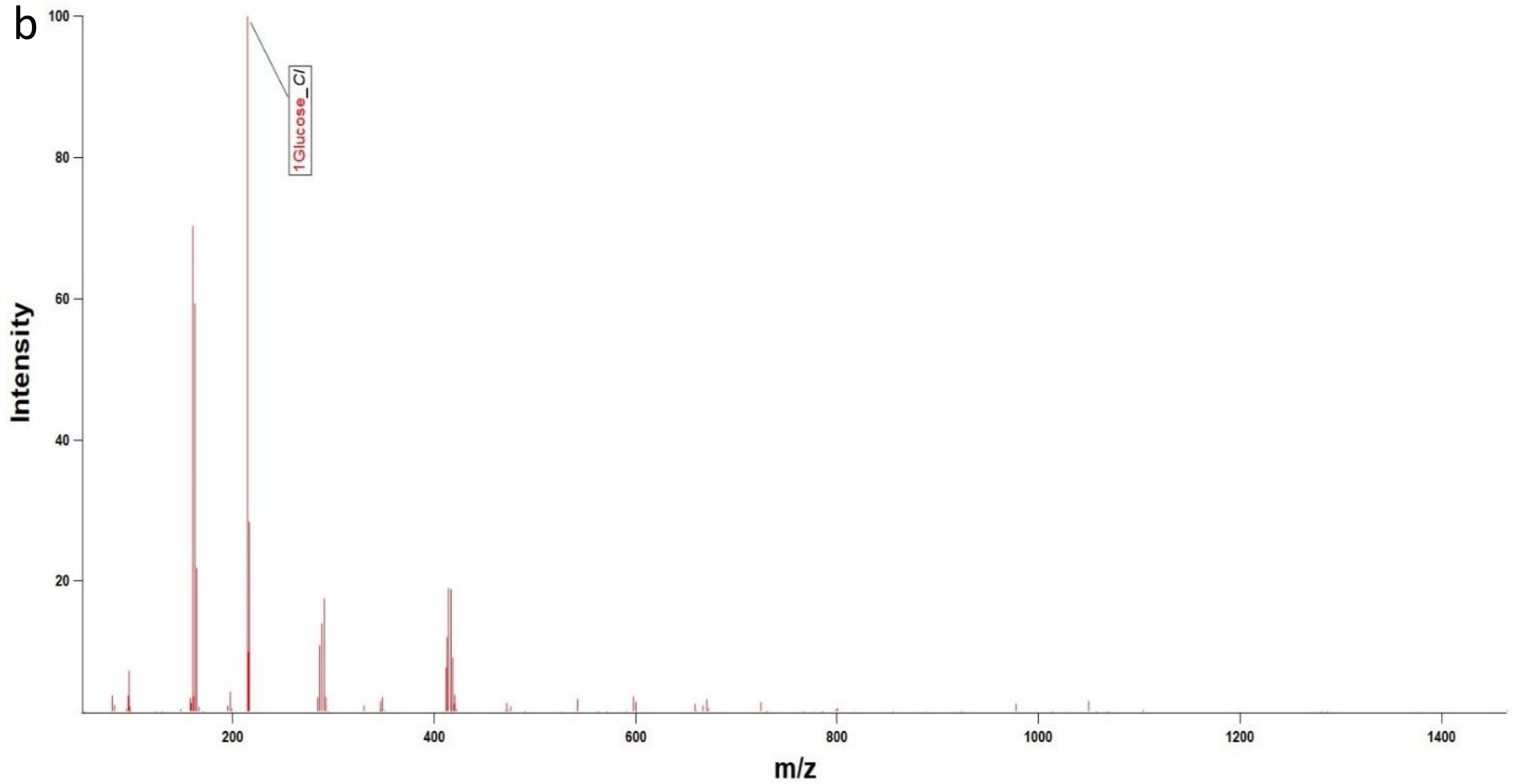

Figure S49. ESI-MS of a fresh solution of glucose (glu) and $FeCl_2$ in an acidified environment**.** Glu and $FeCl_2$ were analyzed by positive mode ESI-MS (a) or negative-mode ESI-MS (b). Labeled species correspond to $[M + Na]^{+}$ or $[M + Fe]^{2+}$ ions (annotations with a hyphen indicate non-covalent complex) (a) or $[M + Cl]^{-}$ ions (b).

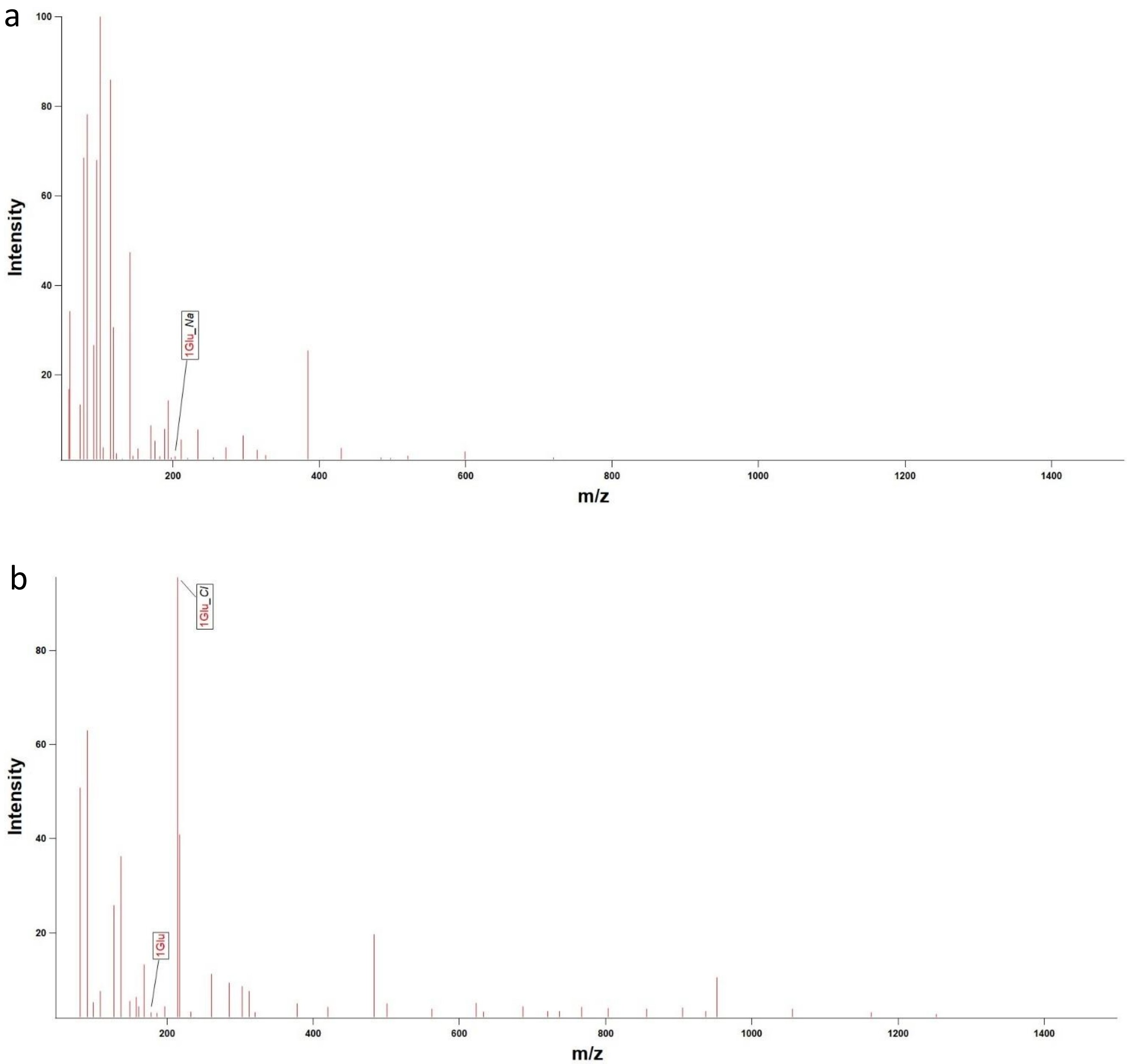

Figure S50. ESI-MS of a fresh solution of glucose (glu) and $AlCl_3$ in an acidified environment**.** Glu and $AlCl_3$ were analyzed by positive mode ESI-MS (a) or negative-mode ESI-MS (b). Labeled species correspond to $[M + Na]^+$ ions (a) or $[M - H]^-$ or $[M + Cl]^-$ ions (b).

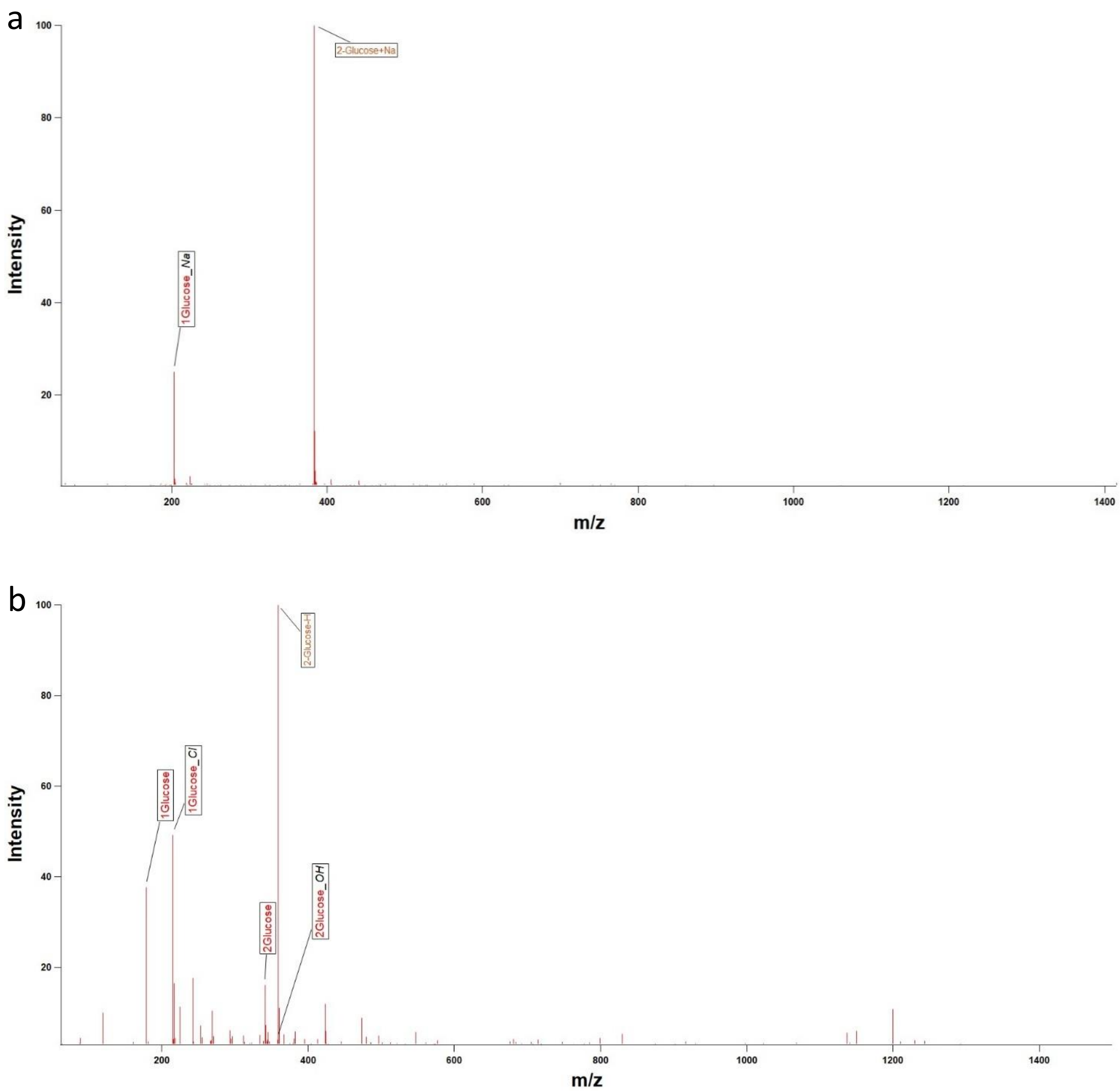

Figure S51. ESI-MS of a fresh solution of glucose (glu) upon addition of NaOH**.** Glu was analyzed by positive mode ESI-MS (a) or negative-mode ESI-MS (b). Labeled species correspond to $[M + Na]^{+}$ions (annotations with a hyphen indicate non-covalent complex) (a) or $[M - H]^{-}$ or $[M + Cl]^{-}$ or $[M + OH]^{-}$ ions (b).

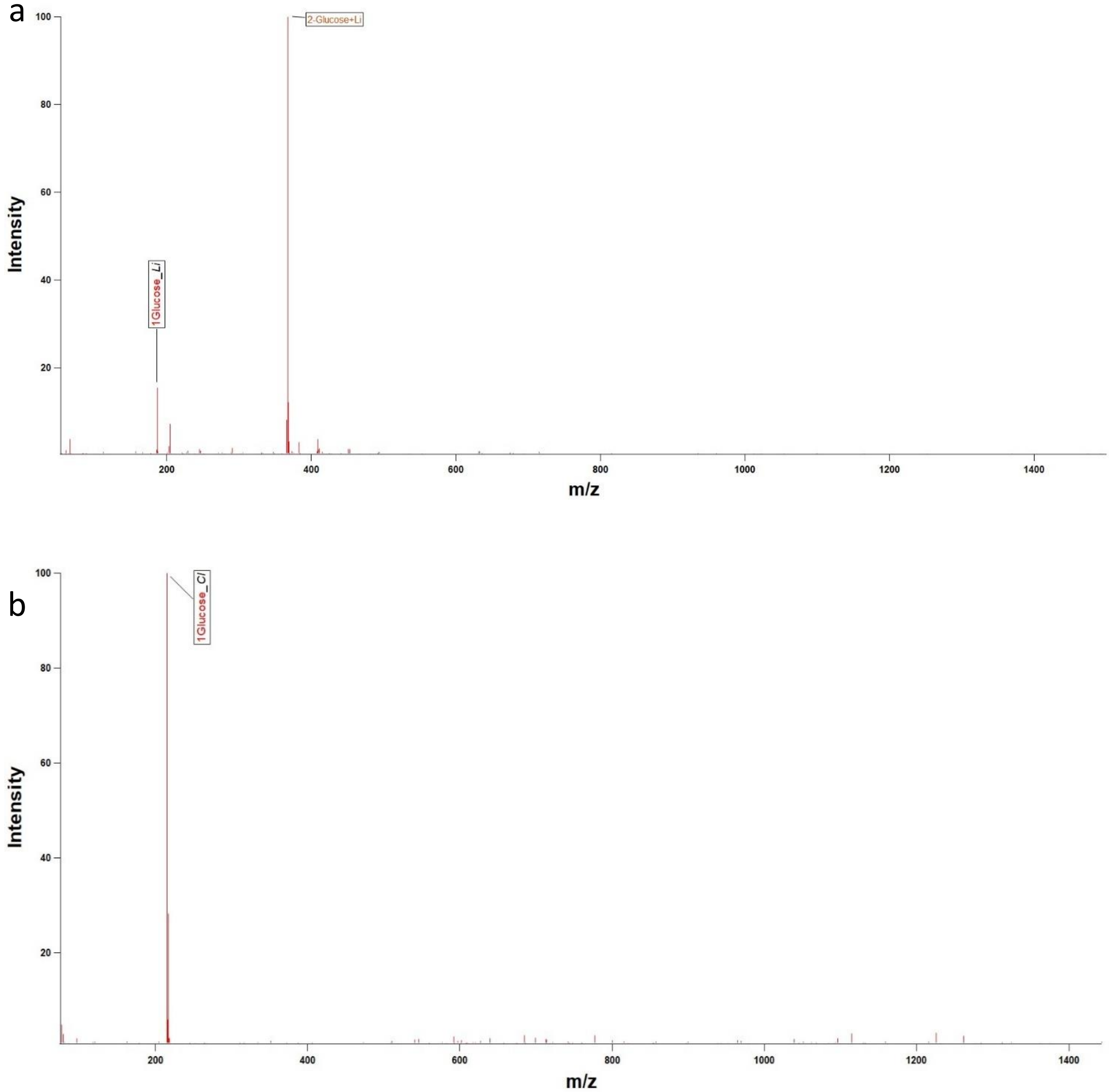

Figure S52. ESI-MS of a fresh solution of glucose (glu) and LiCl upon addition of NaOH**.** Glu and LiCl were analyzed by positive mode ESI-MS (a) or negative-mode ESI-MS (b). Labeled species correspond to $[M + Li]^{+}$ ions (annotations with a hyphen indicate non-covalent complex) (a) or $[M + Cl]^{-}$ ions (b).

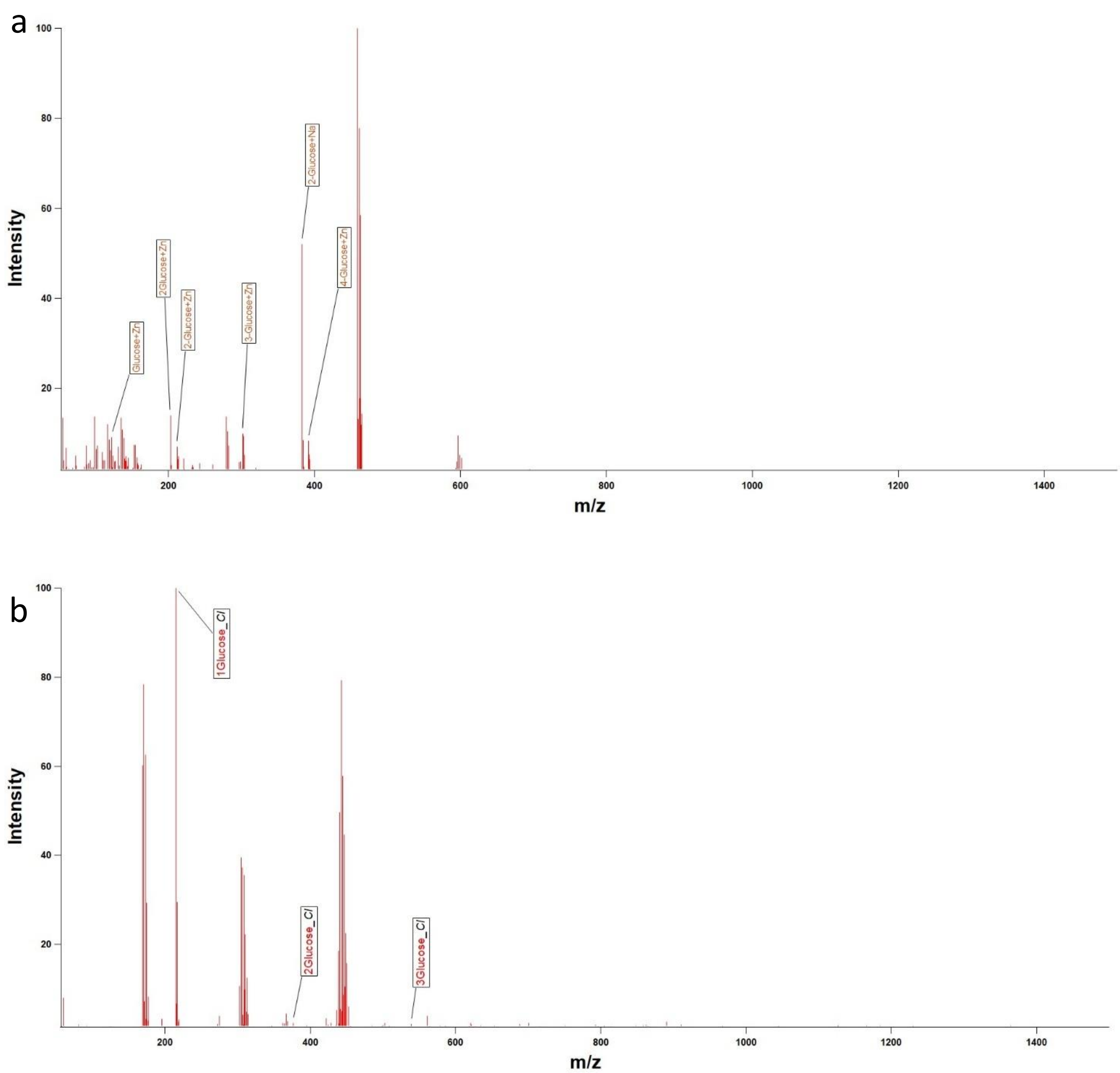

Figure S53. ESI-MS of a fresh solution of glucose (glu) and $ZnCl_2$ upon addition of NaOH**.** Glu and $ZnCl_2$ were analyzed by positive mode ESI-MS (a) or negative-mode ESI-MS (b). Labeled species correspond to $[M + Na]^+$ or $[M + Zn]^{2+}$ ions (annotations with a hyphen indicate non-covalent complex) (a) or $[M + Cl]^-$ ions (b).

a
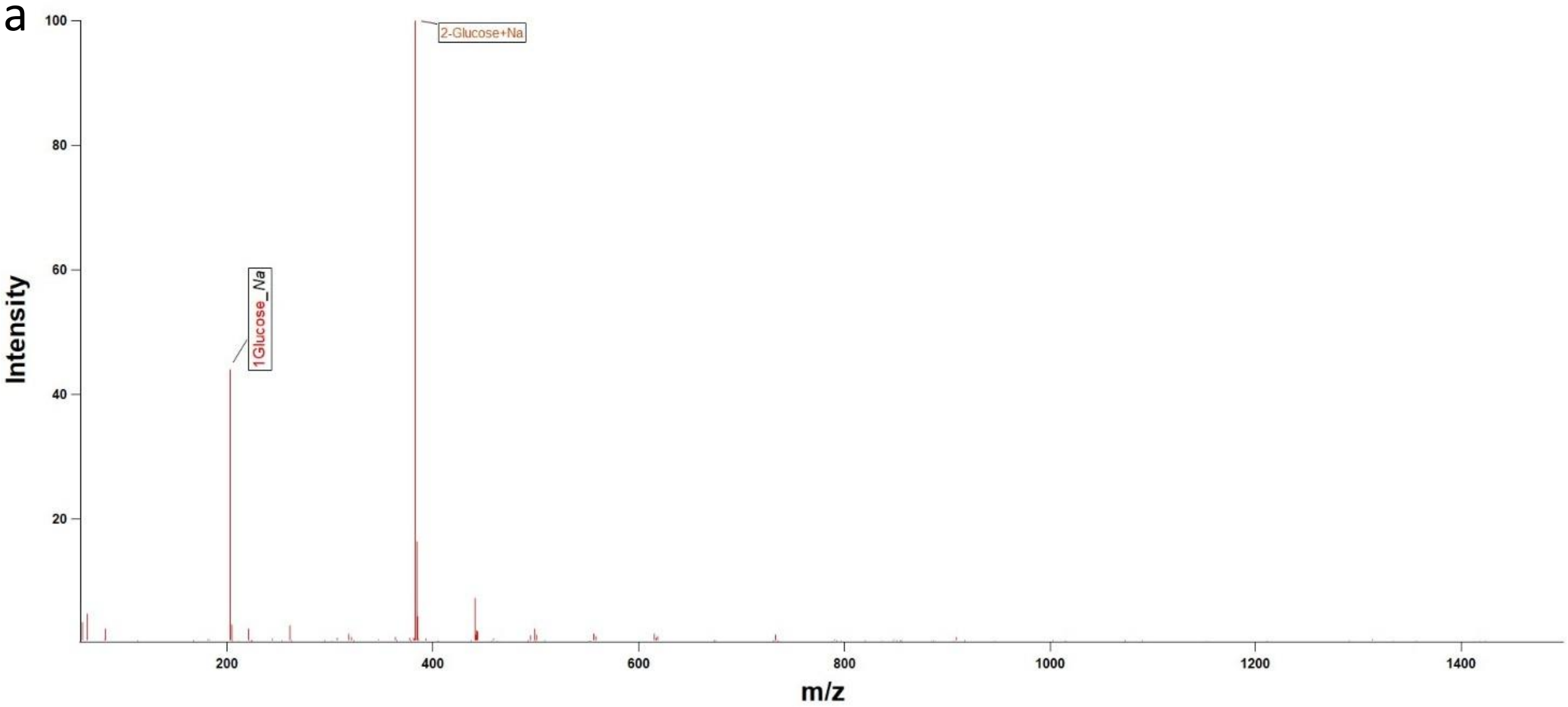

b
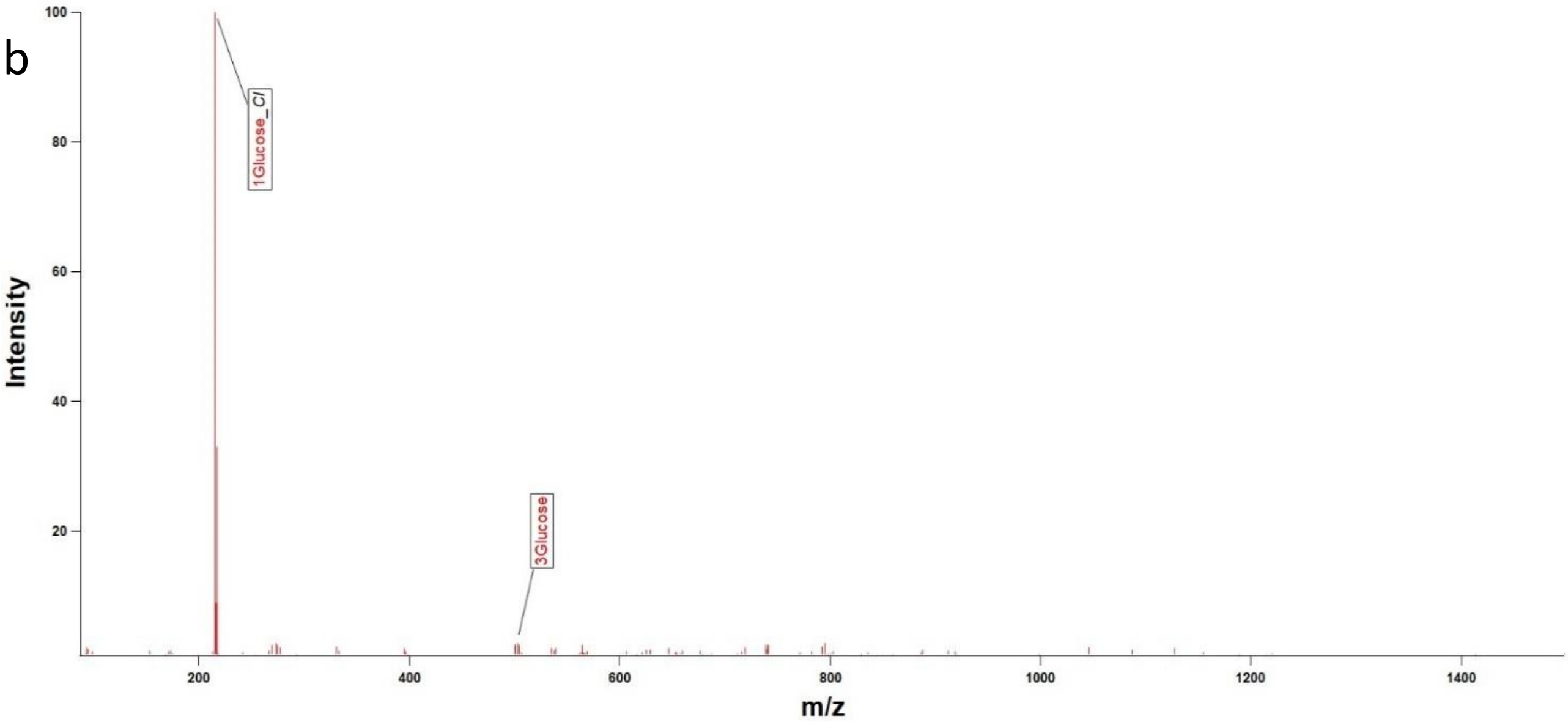

Figure S54. ESI-MS of a fresh solution of glucose (glu) and NaCl upon addition of NaOH. Glu and NaCl were analyzed by positive mode ESI-MS (a) or negative-mode ESI-MS (b). Labeled species correspond to $[M+H]^+$ or $[M+Na]^+$ ions (annotations with a hyphen indicate non-covalent complex) (a) or $[M-H]^-$ or $[M+Cl]^-$ ions (b).

a

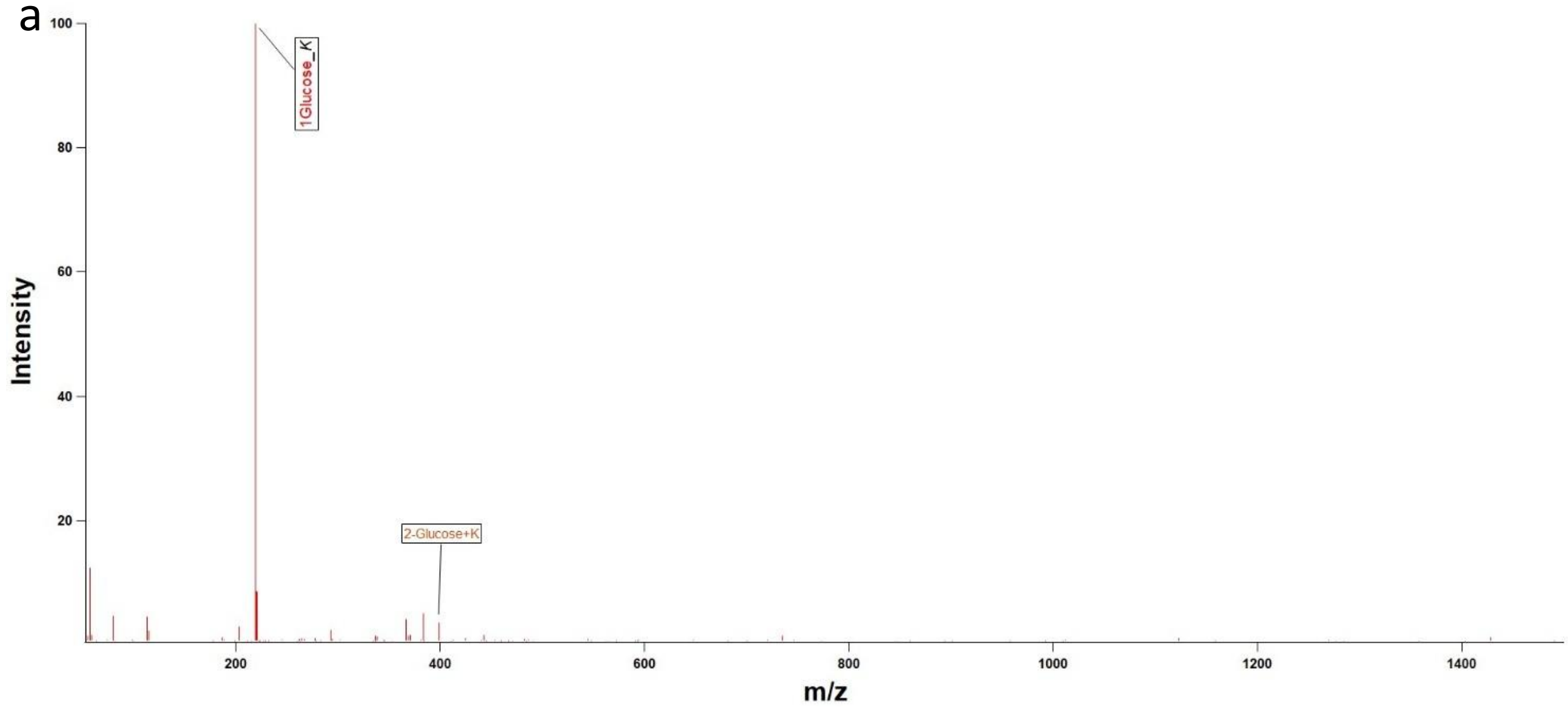

b

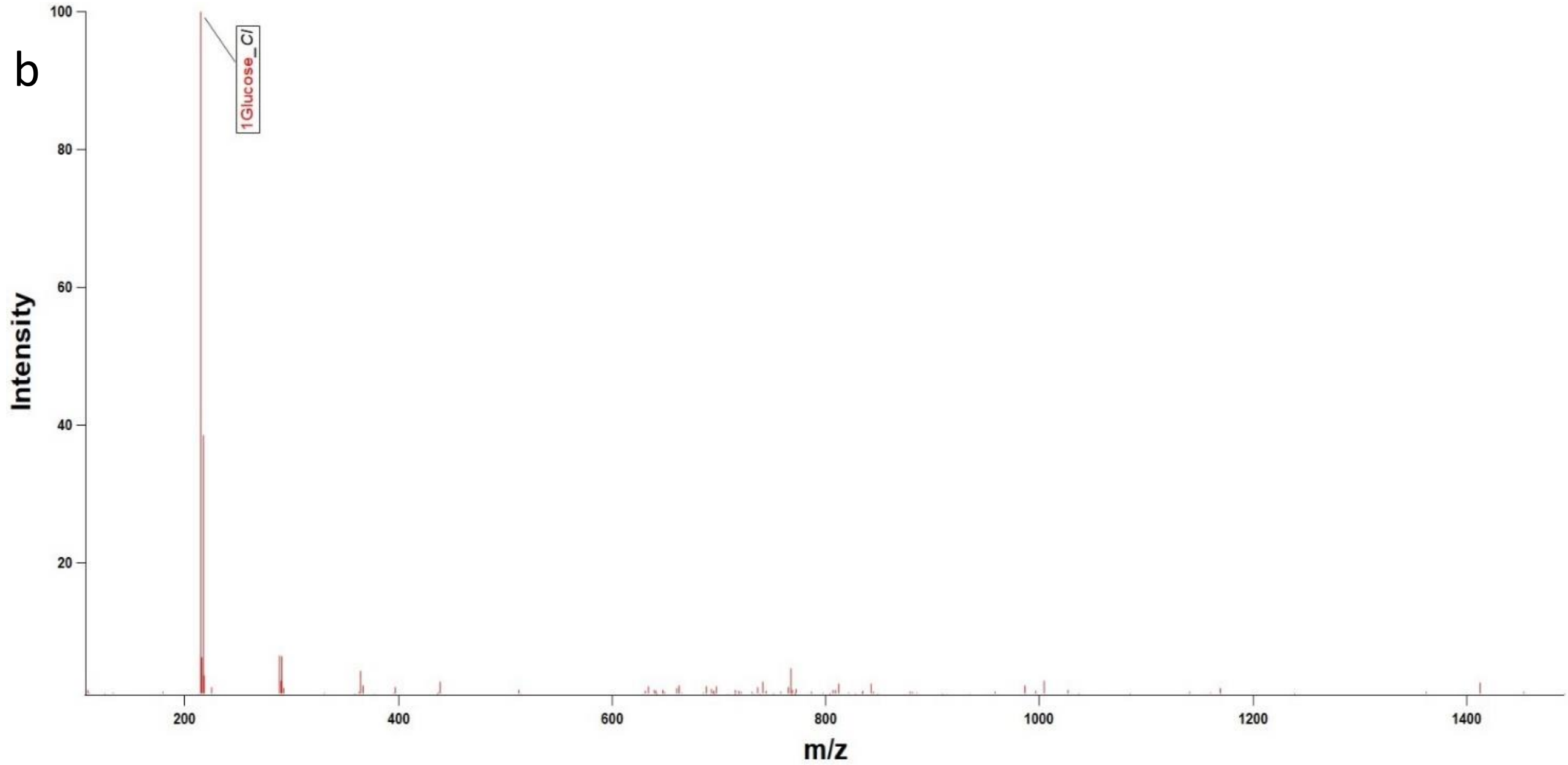

Figure S55. ESI-MS of a fresh solution of glucose (glu) and KCl upon addition of NaOH**.** Glu and KCl were analyzed by positive mode ESI-MS (a) or negative-mode ESI-MS (b). Labeled species correspond to $[M+K]^+$ ions (annotations with a hyphen indicate non-covalent complex) (a) or $[M+Cl]^-$ ions (b).

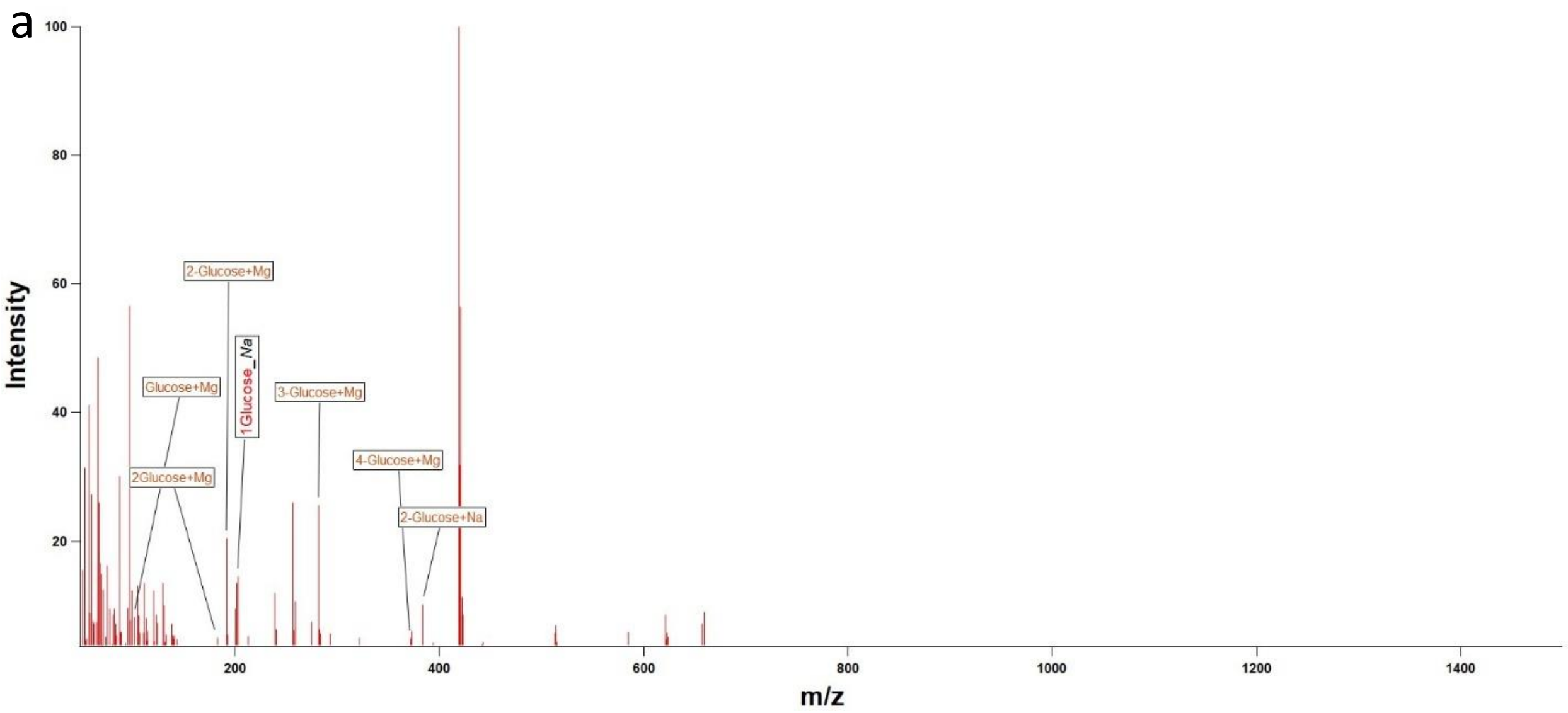

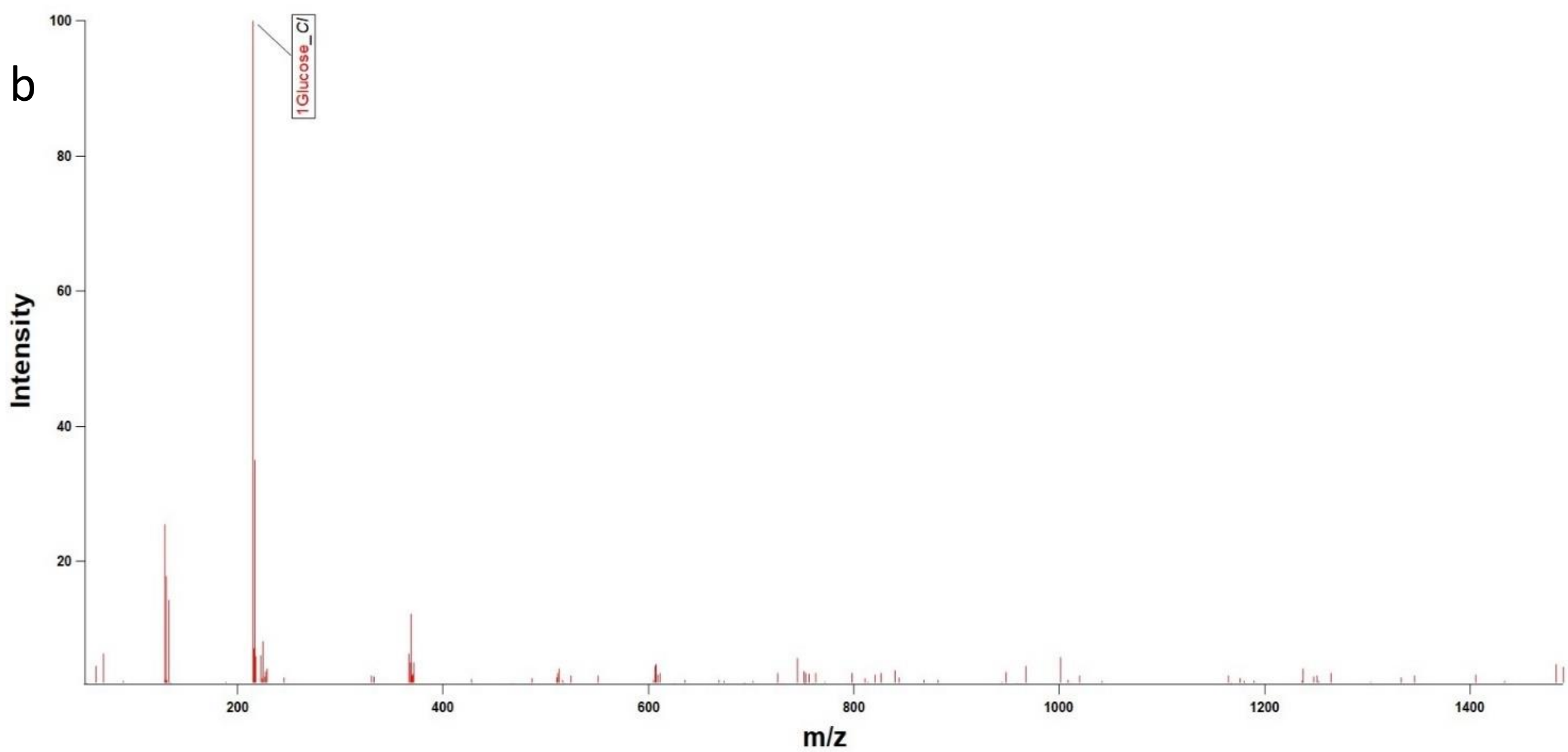

Figure S56. ESI-MS of a fresh solution of glucose (glu) and $MgCl_2$ upon addition of NaOH. Glu and $MgCl_2$ were analyzed by positive mode ESI-MS (a) or negative-mode ESI-MS (b). Labeled species correspond to $[M + Na]^{+}$ or $[M + Mg]^{2+}$ ions (annotations with a hyphen indicate non-covalent complex) (a) or $[M + Cl]^{-}$ ions (b).

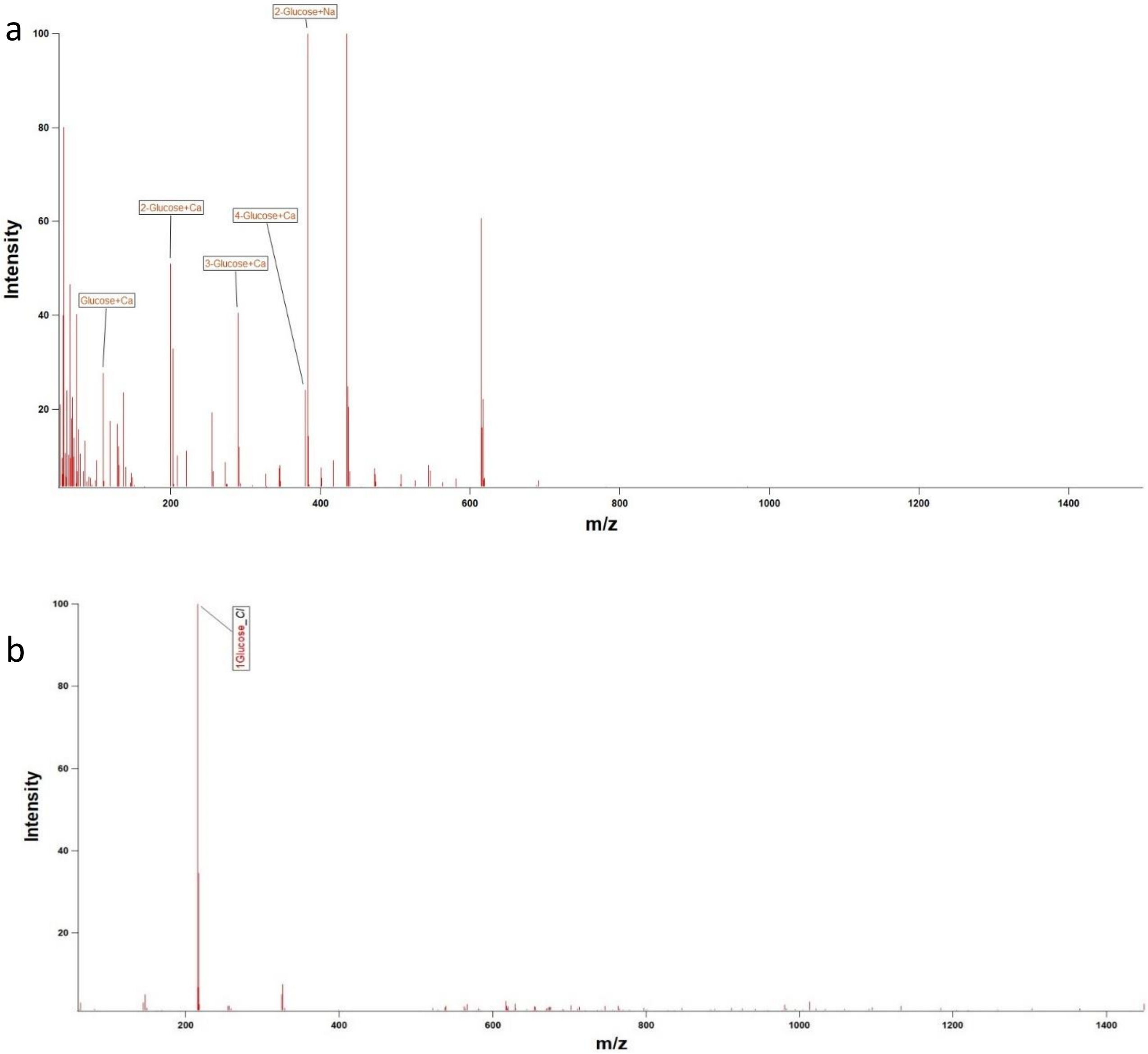

Figure S57. ESI-MS of a fresh solution of glucose (glu) and $CaCl_2$ upon addition of NaOH**.** Glu and $CaCl_2$ were analyzed by positive mode ESI-MS (a) or negative-mode ESI-MS (b). Labeled species correspond to $[M + Na]^{+}$ or $[M + Ca]^{2+}$ ions (annotations with a hyphen indicate non-covalent complex) (a) or $[M + Cl]^{-}$ ions (b).

a

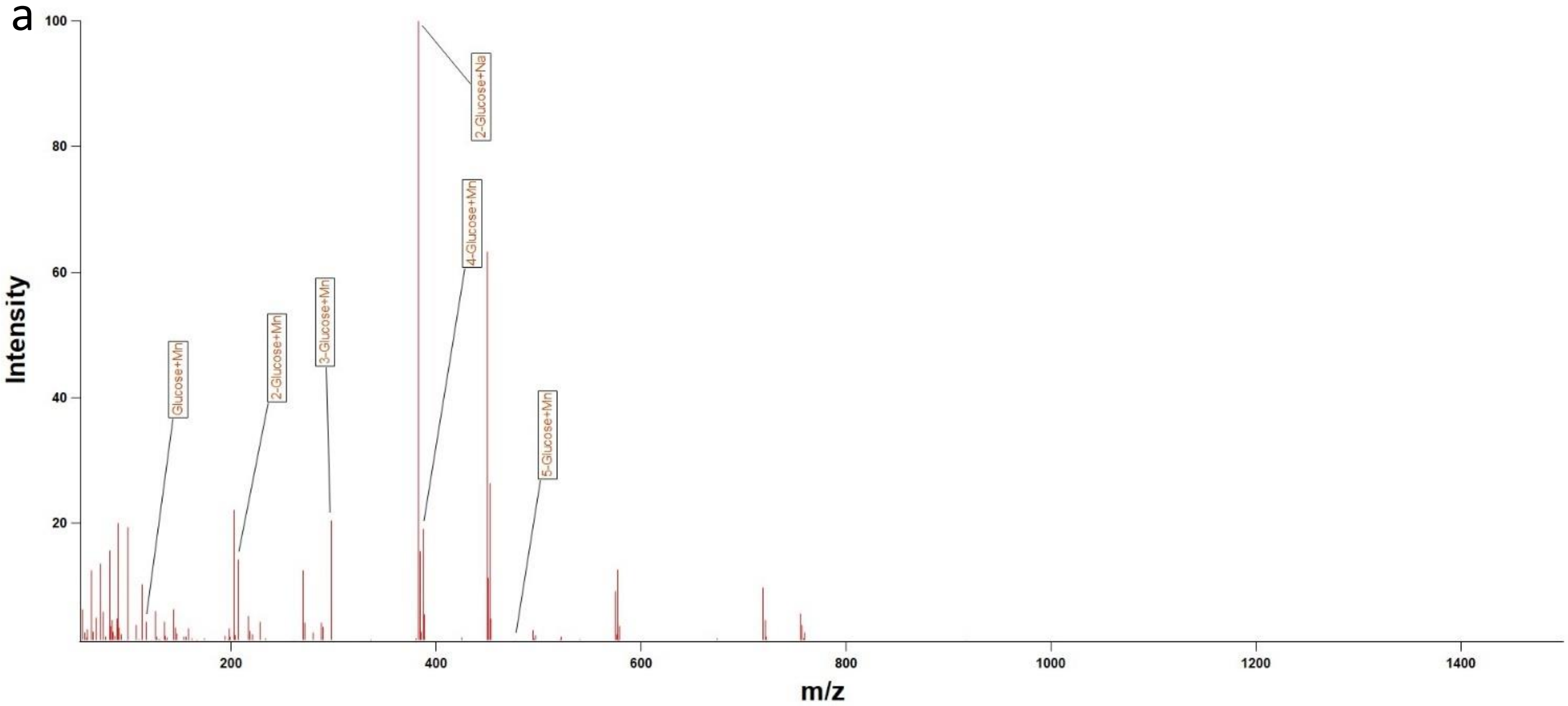

b

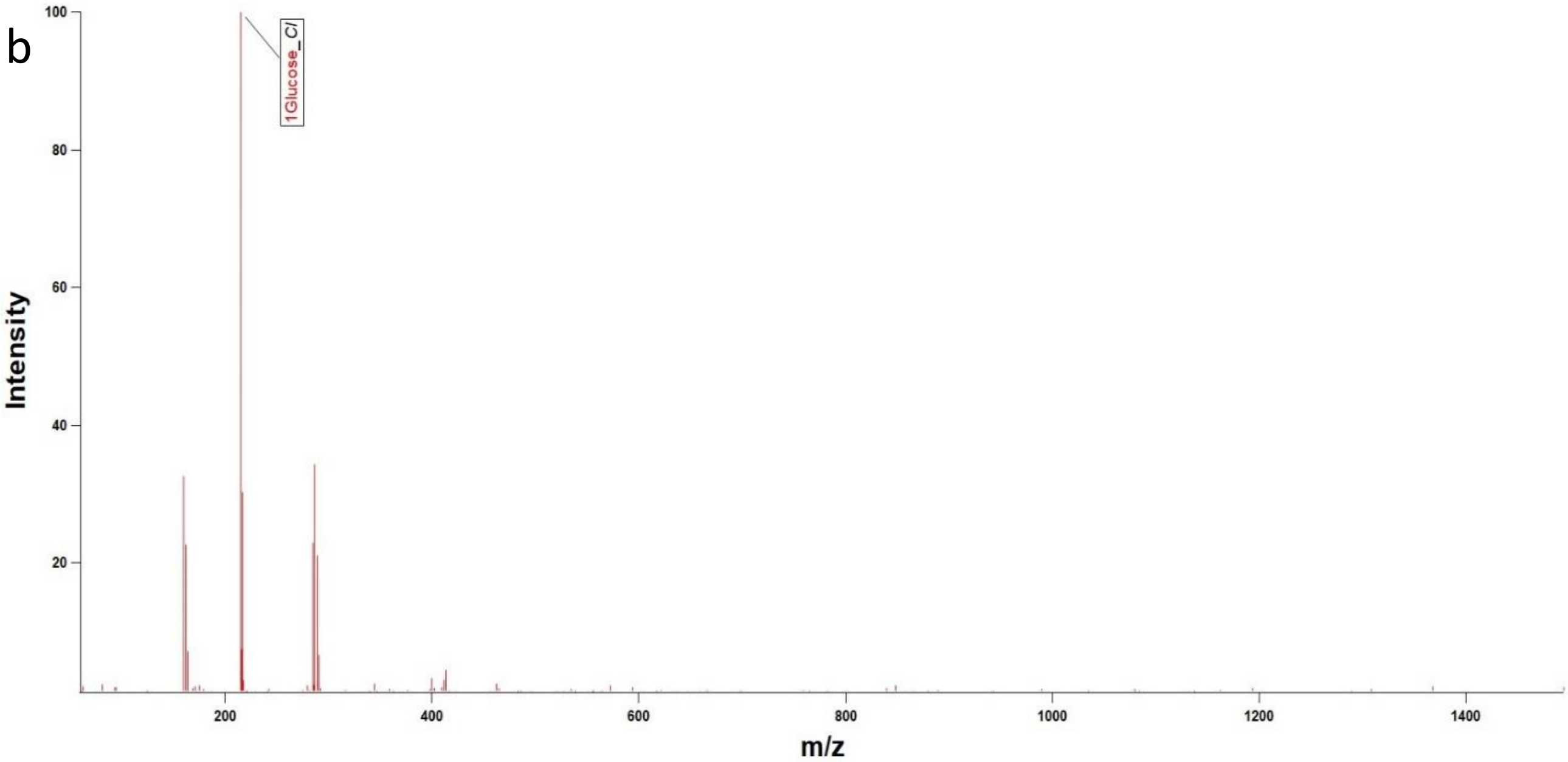

Figure S58. ESI-MS of a fresh solution of glucose (glu) and $MnCl_2$ upon addition of NaOH**.** Glu and $MnCl_2$ were analyzed by positive mode ESI-MS (a) or negative-mode ESI-MS (b). Labeled species correspond to $[M + Na]^{+}$ or $[M + Mn]^{2+}$ ions (annotations with a hyphen indicate non-covalent complex) (a) or $[M + Cl]^{-}$ ions (b).

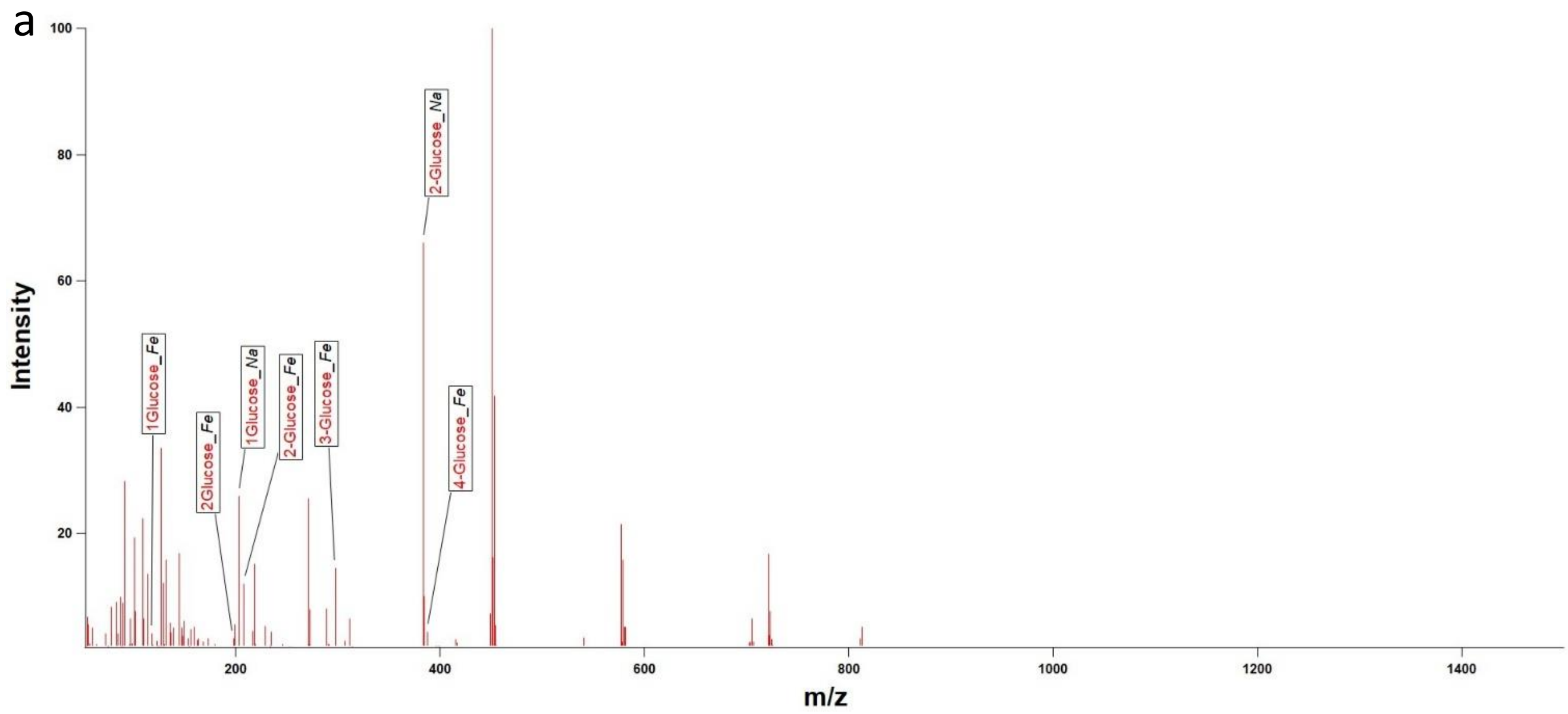

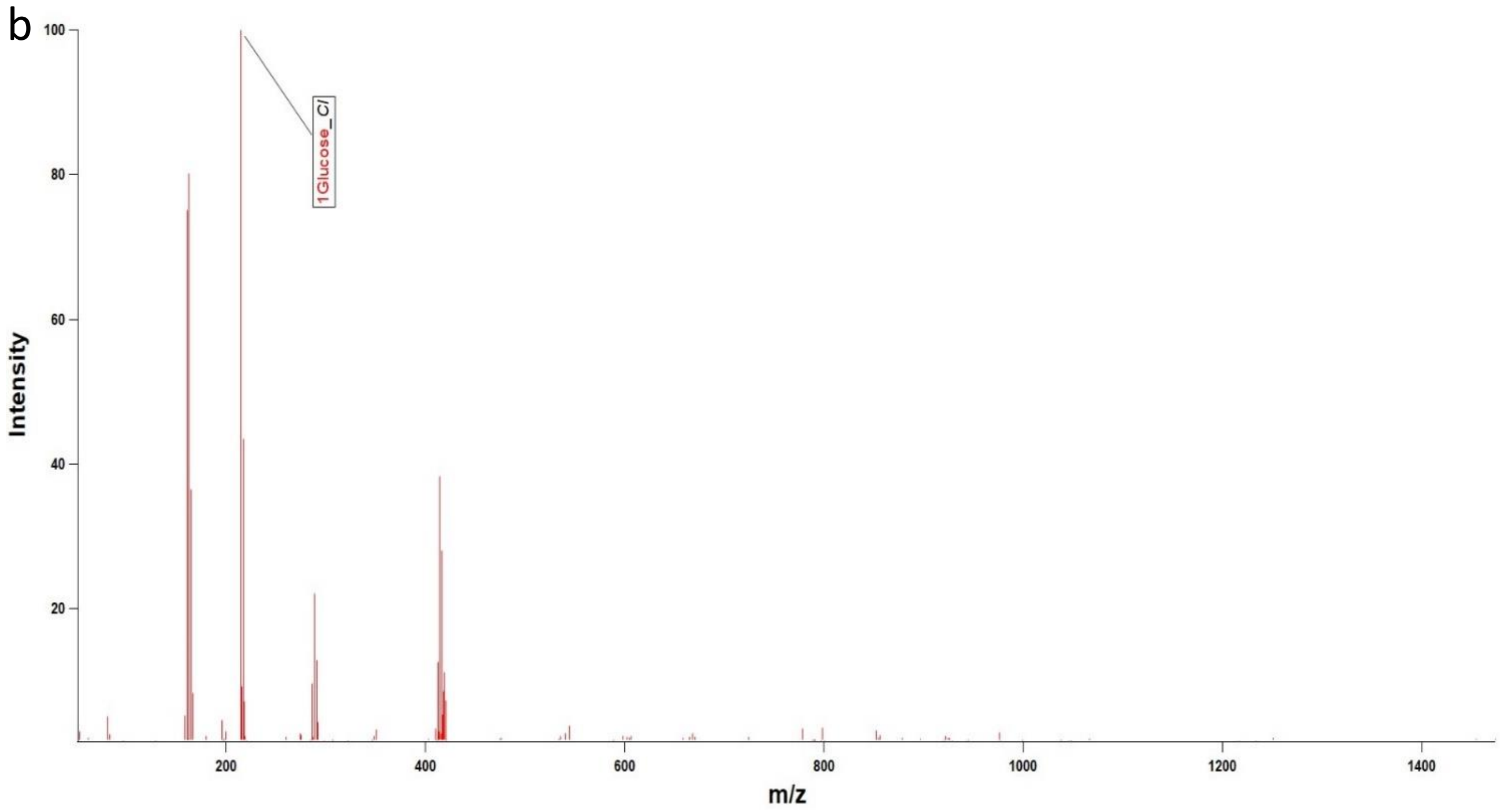

Figure S59. ESI-MS of a fresh solution of glucose (glu) and $FeCl_2$ upon addition of NaOH**.** Glu and $FeCl_2$ were analyzed by positive mode ESI-MS (a) or negative-mode ESI-MS (b). Labeled species correspond to $[M + Na]^{+}$ or $[M + Fe]^{2+}$ ions (annotations with a hyphen indicate non-covalent complex) (a) or $[M + Cl]^{-}$ ions (b).

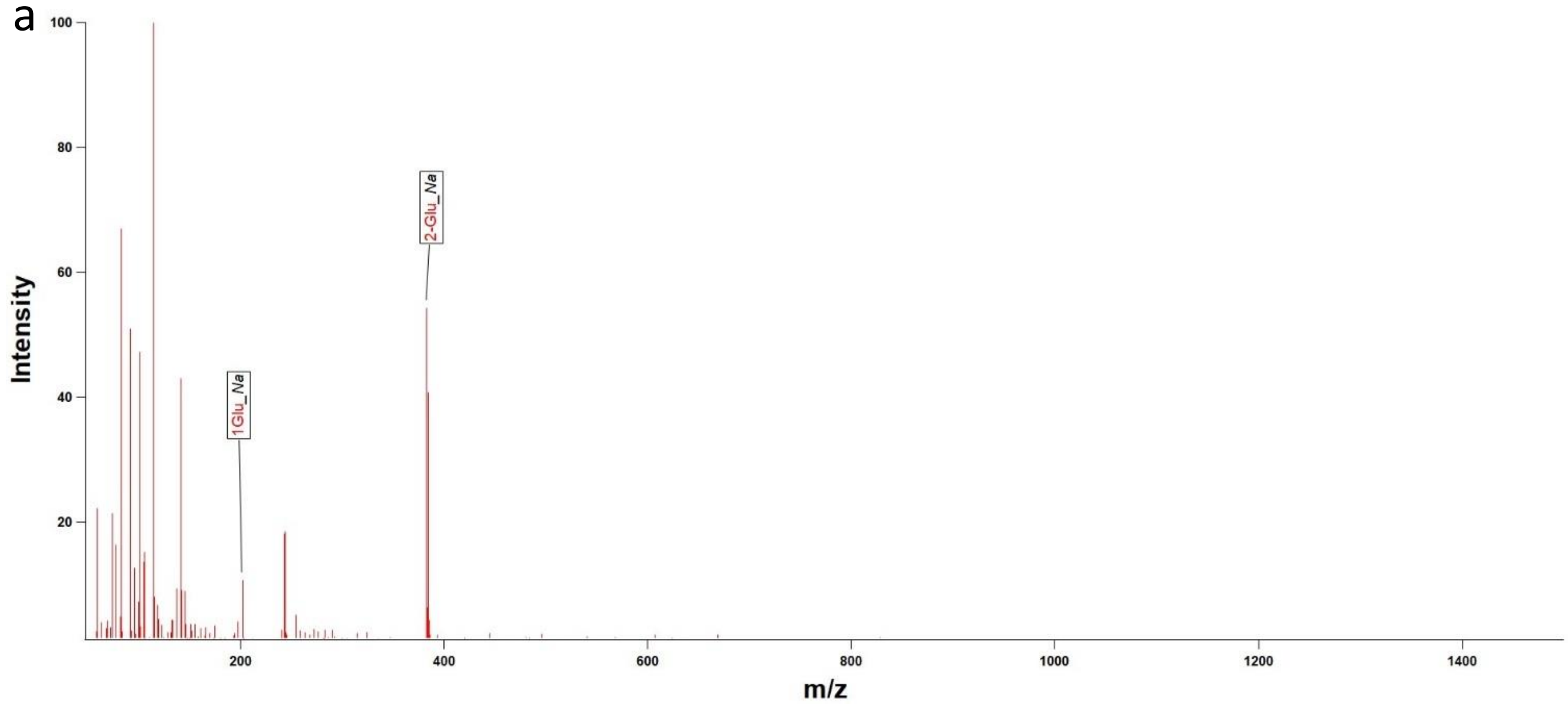

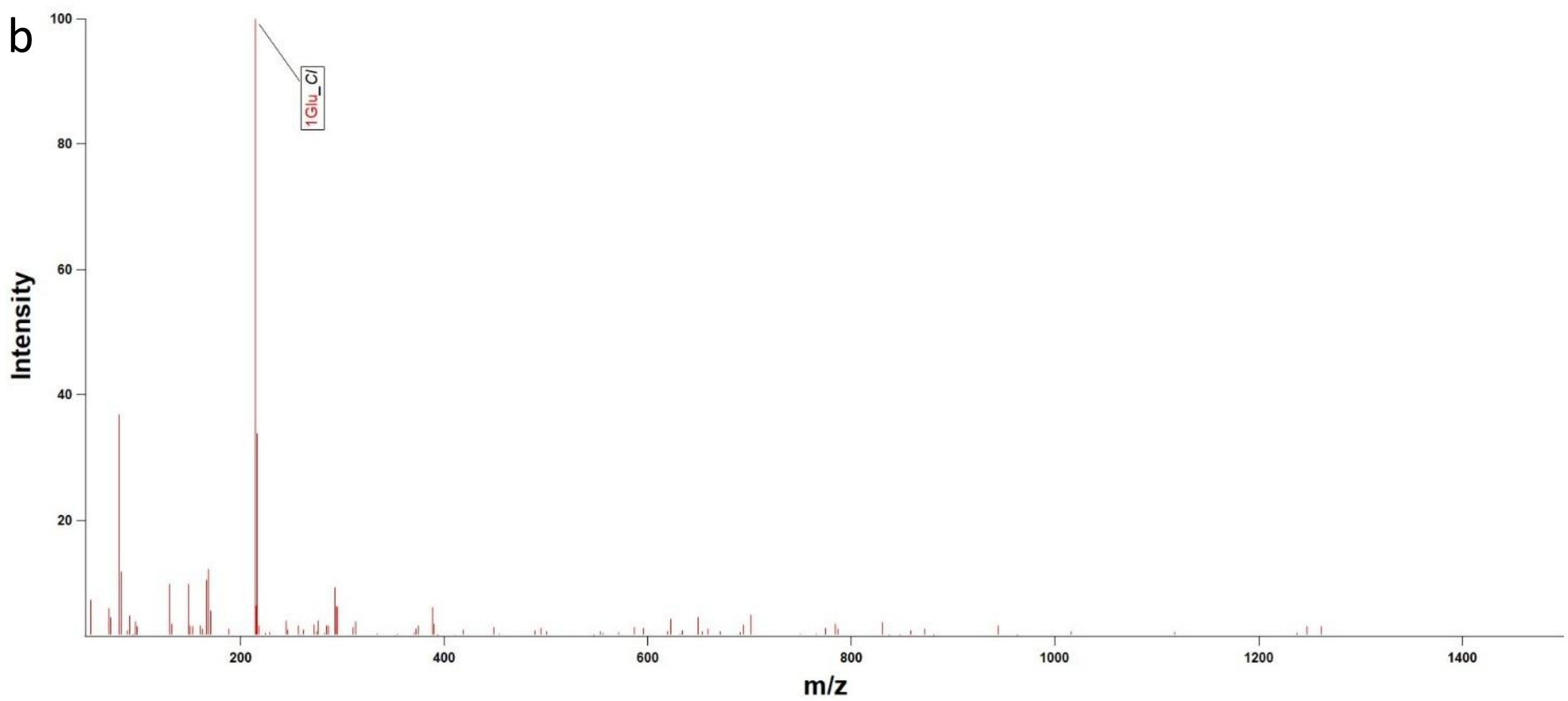

Figure S60. ESI-MS of a fresh solution of glucose (glu) and $AlCl_3$ upon addition of NaOH**.** Glu and $AlCl_3$ were analyzed by positive mode ESI-MS (a) or negative-mode ESI-MS (b). Labeled species correspond to $[M+Na]^+$ ions (annotations with a hyphen indicate non-covalent complex) (a) or $[M+Cl]^-$ ions (b).

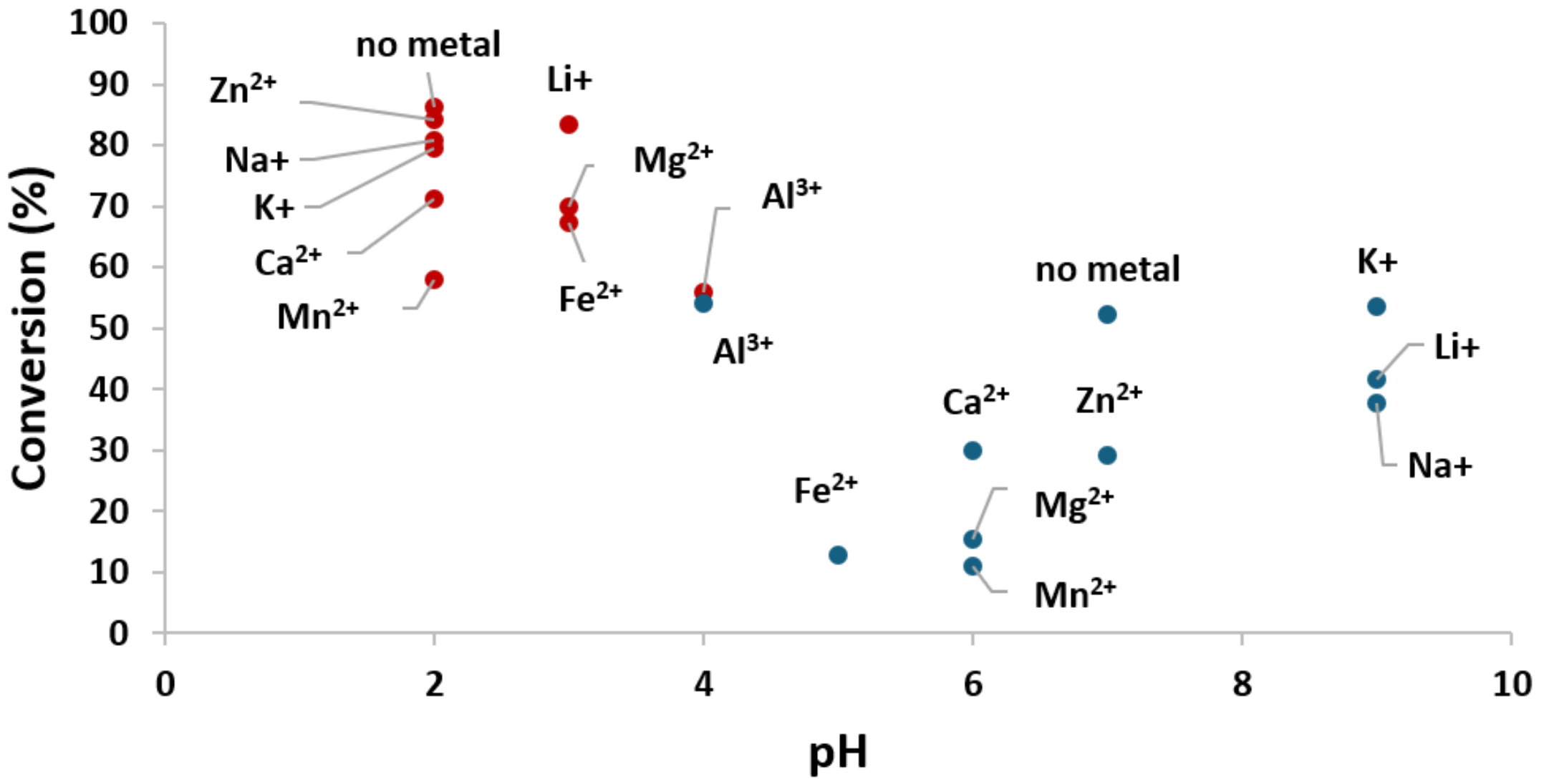

Figure S61. A plot summarizing the conversion of D-Glucose under acidified conditions (red) and upon addition of NaOH (blue) versus final pH in the samples. Integrations were based on ^{1}H-NMR resonances of the anomeric protons before and after the dry-down reactions (see Materials and Methods).

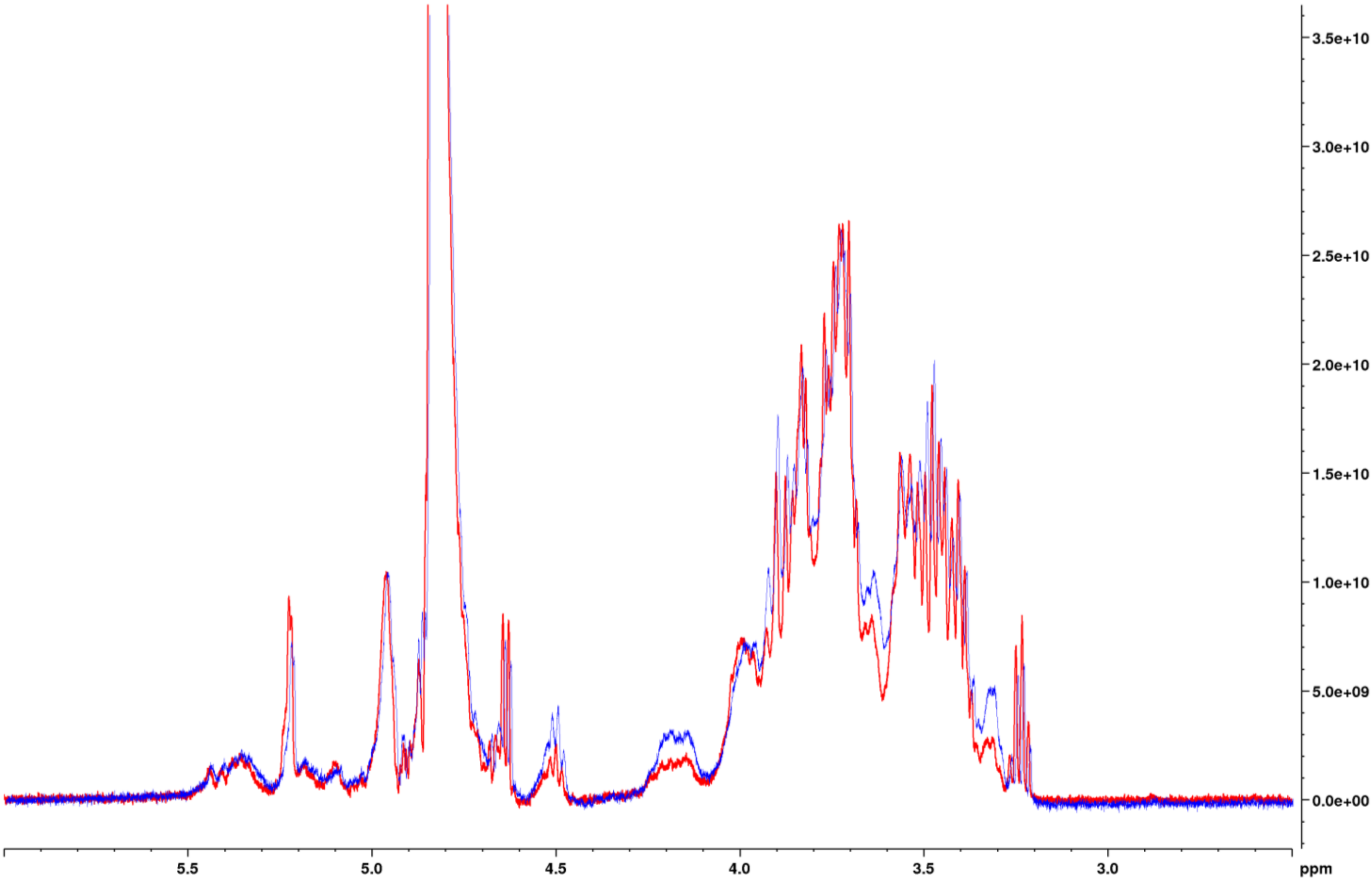

Figure S62. ^{1}H-NMR spectra overlay of dry-down reaction of glucose in the absence (blue) or presence (red) of LiCl under acidified environment. Solutions were prepared following drying at 50˚C for seven days. The products were dissolved in D_2O buffer (pH=2.66).

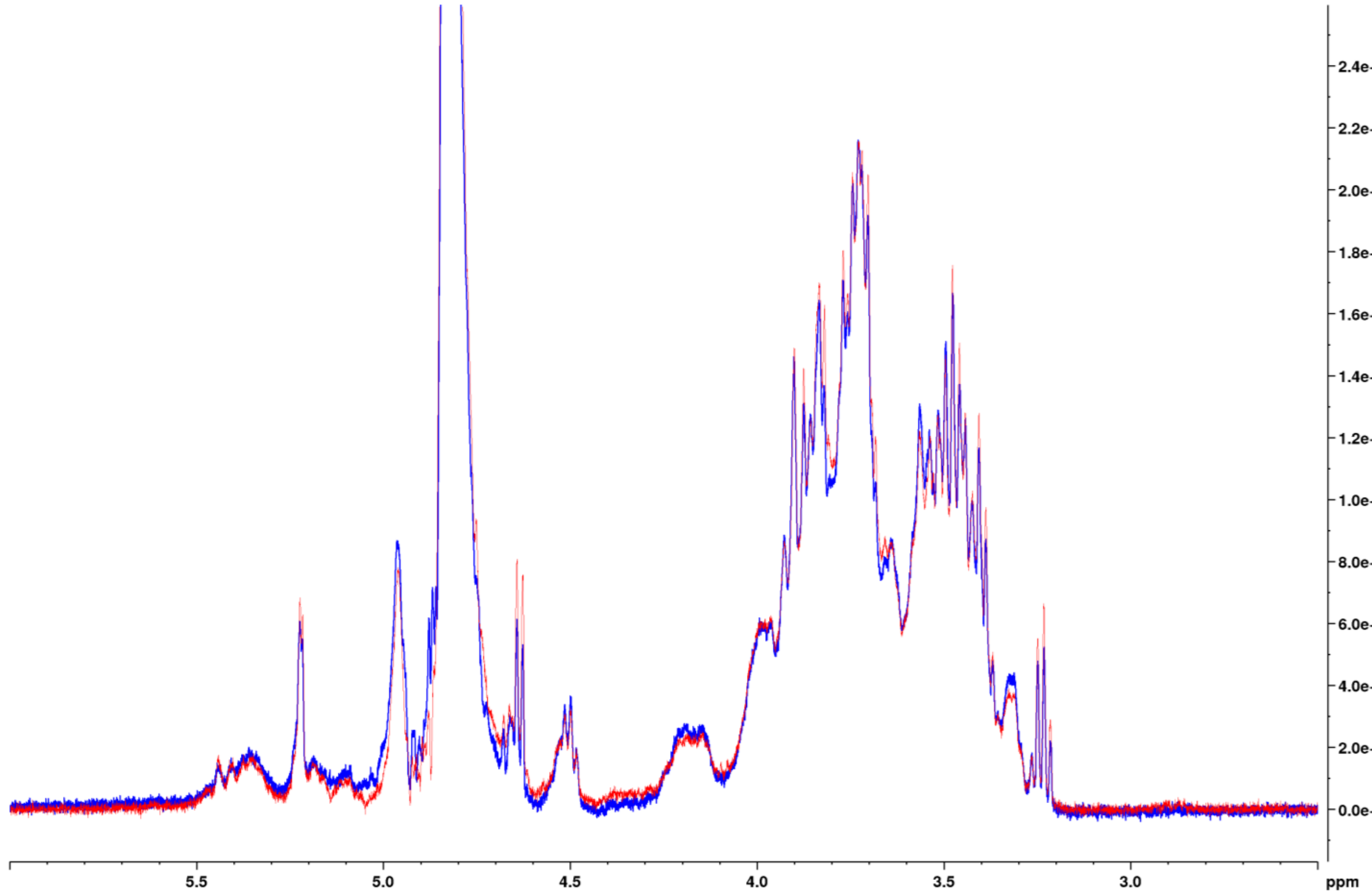

Figure S63. ^{1}H-NMR spectra overlay of dry-down reaction of glucose in the absence (blue) or presence (red) of $ZnCl_2$ under acidified environment. Solutions were prepared following drying at 50˚C for seven days. The products were dissolved in D_2O buffer (pH=2.66).

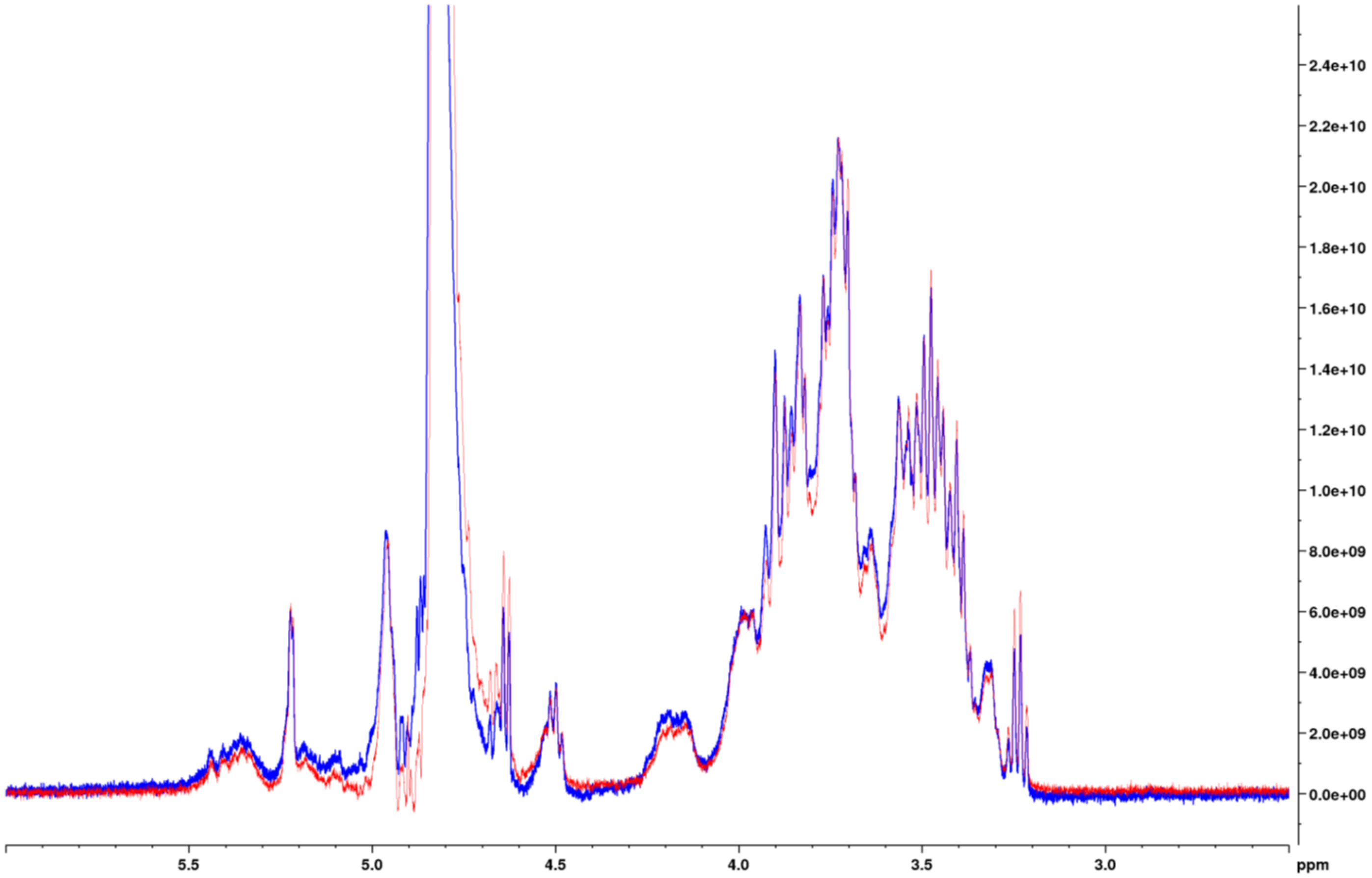

Figure S64. ^{1}H-NMR spectra overlay of dry-down reaction of glucose in the absence (blue) or presence (red) of NaCl under acidified environment. Solutions were prepared following drying at 50˚C for seven days. The products were dissolved in D_2O buffer (pH=2.66).

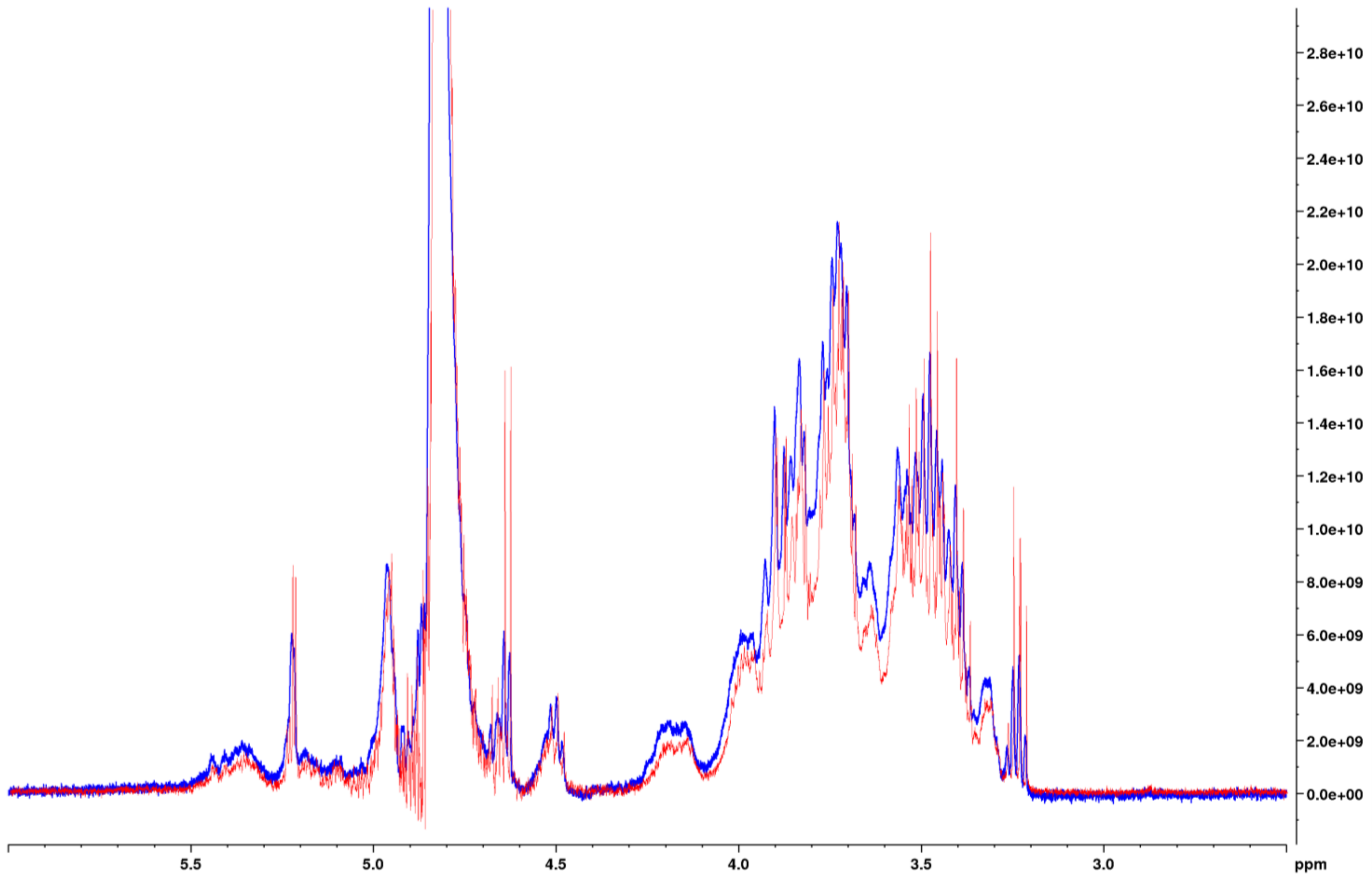

Figure S65. ^{1}H-NMR spectra overlay of dry-down reaction of glucose in the absence (blue) or presence (red) of KCl under acidified environment. Solutions were prepared following drying at 50˚C for seven days. The products were dissolved in D_2O buffer (pH=2.66).

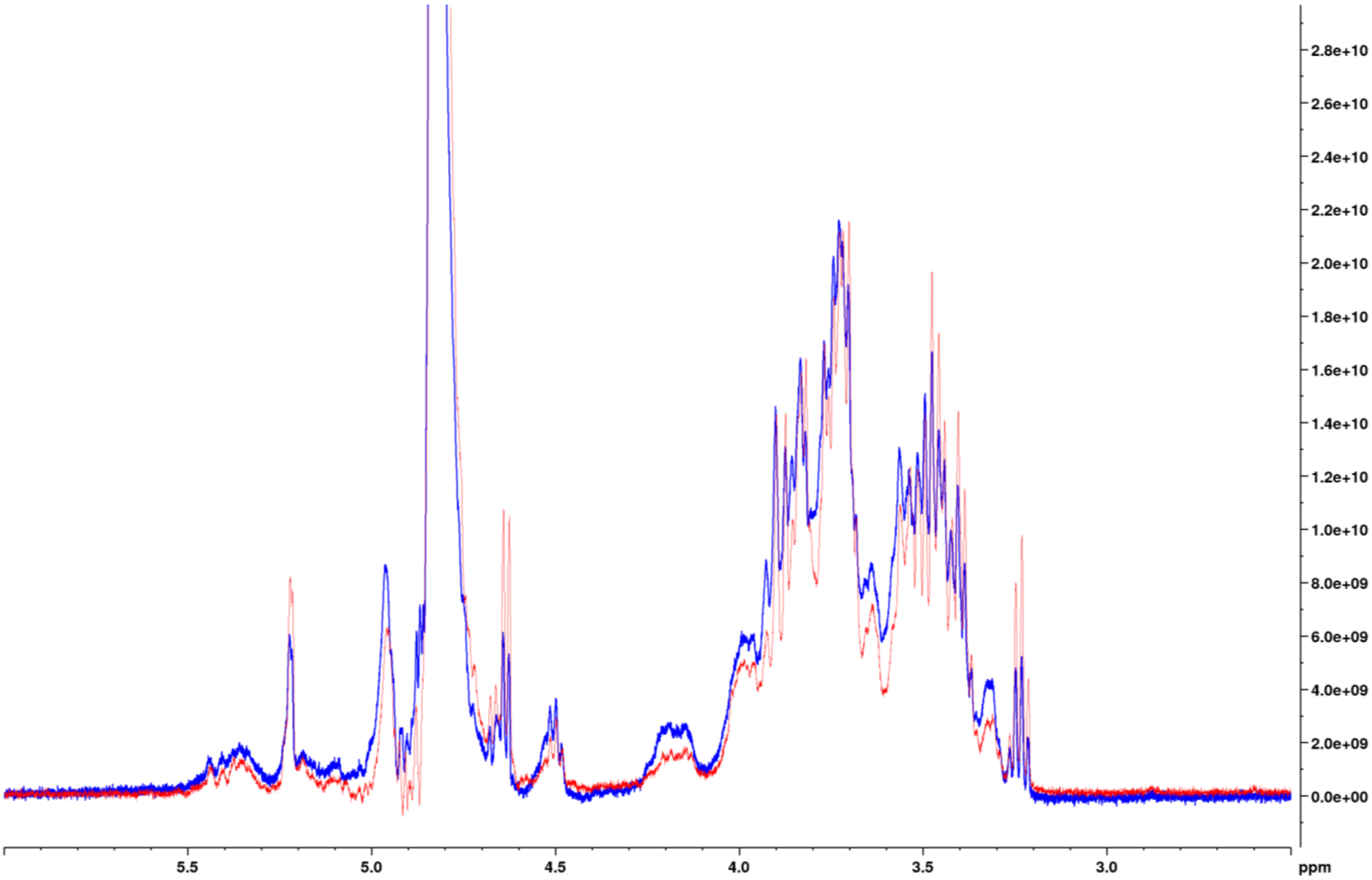

Figure S66. ^{1}H-NMR spectra overlay of dry-down reaction of glucose in the absence (blue) or presence (red) of $MgCl_2$ under acidified environment. Solutions were prepared following drying at 50˚C for seven days. The products were dissolved in D_2O buffer (pH=2.66).

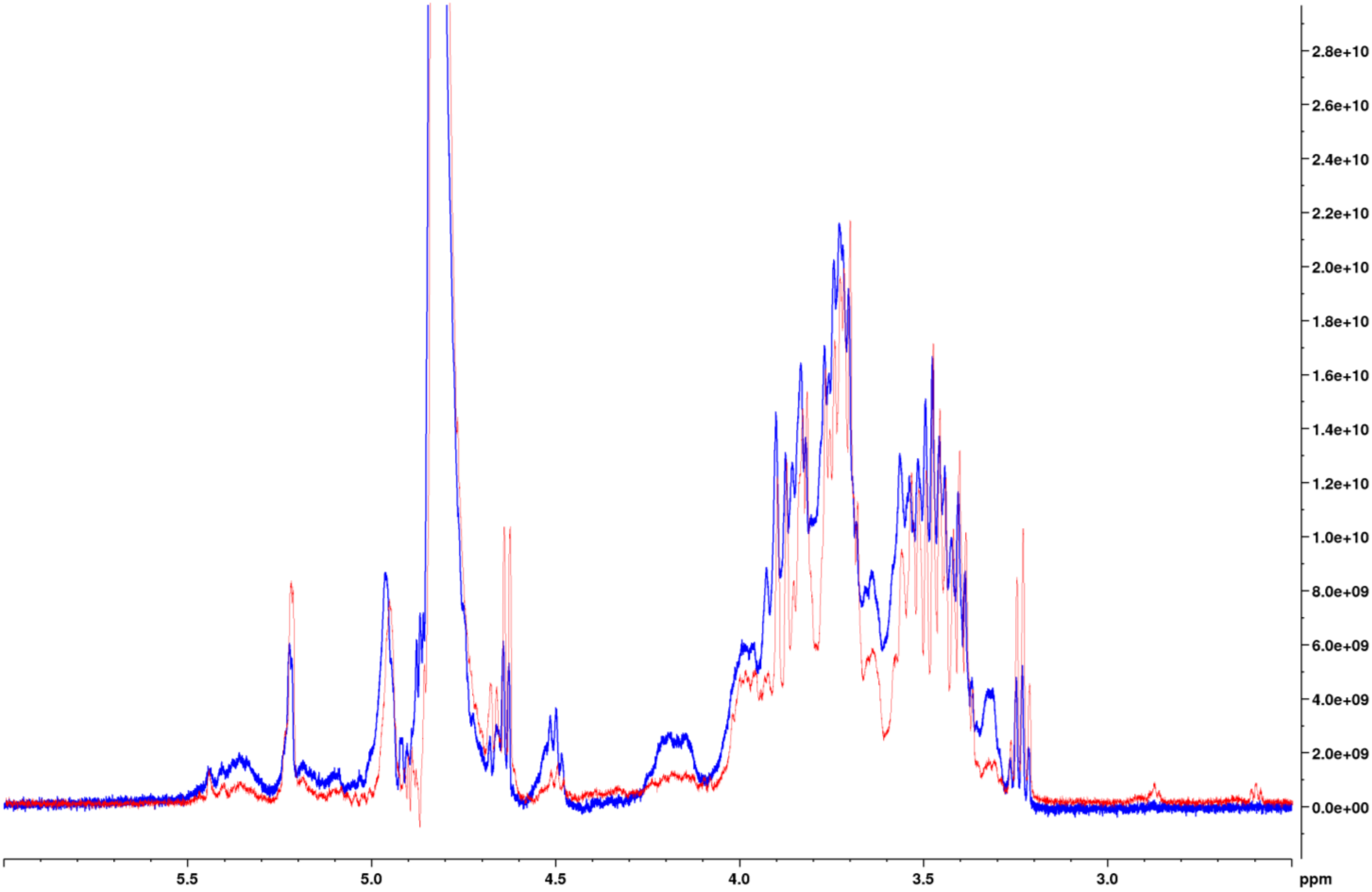

Figure S67. ^1H-NMR spectra overlay of dry-down reaction of glucose in the absence (blue) or presence (red) of $CaCl_2$ under acidified environment. Solutions were prepared following drying at 50˚C for seven days. The products were dissolved in D_2O buffer (pH=2.66).

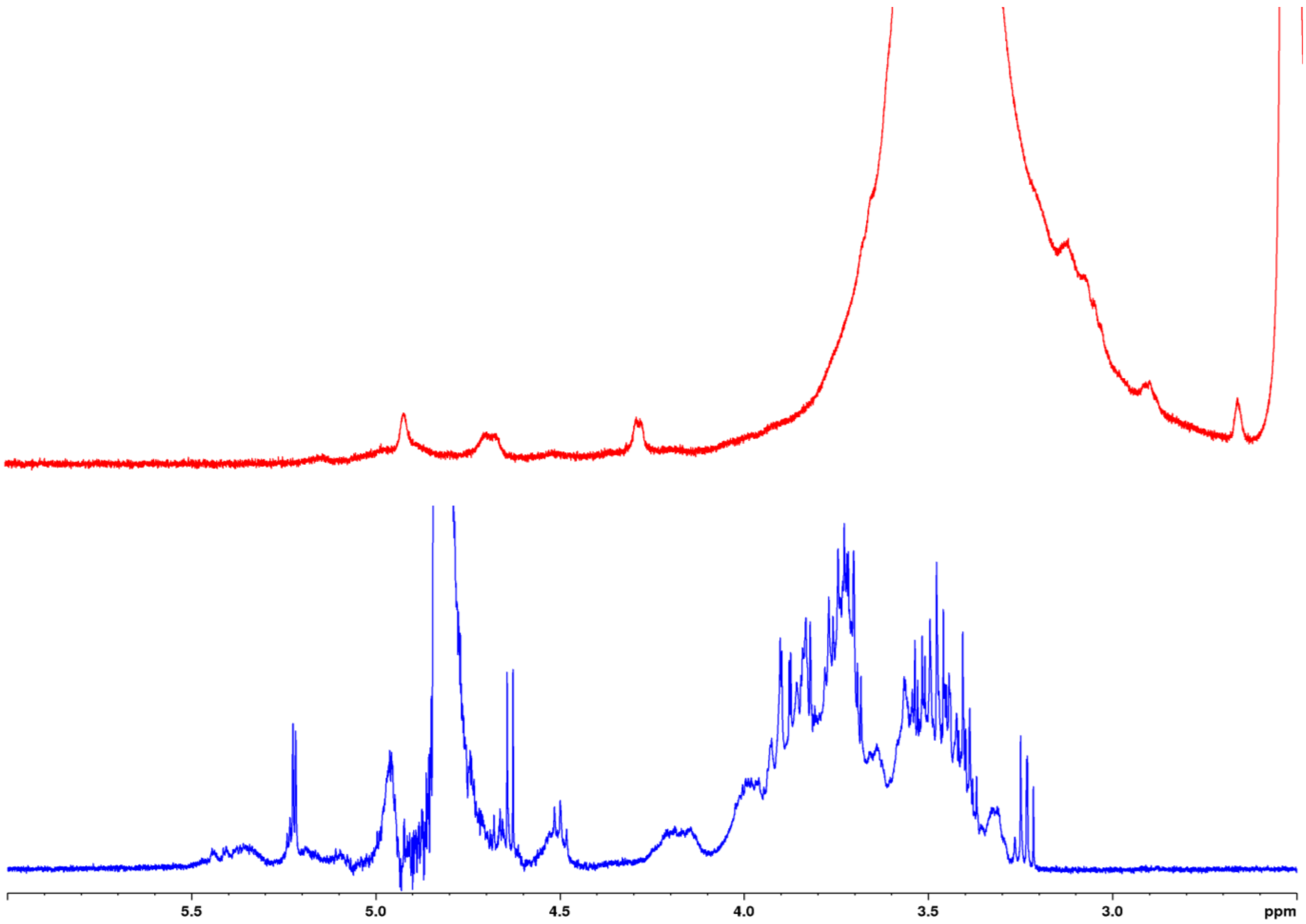

Figure S68. ^{1}H-NMR spectra stack of dry-down reaction of glucose in the absence (blue) or presence (red) of $MnCl_2$ under acidified environment. Solutions were prepared following drying at 50˚C for seven days. The products were dissolved in DMSO-d6.

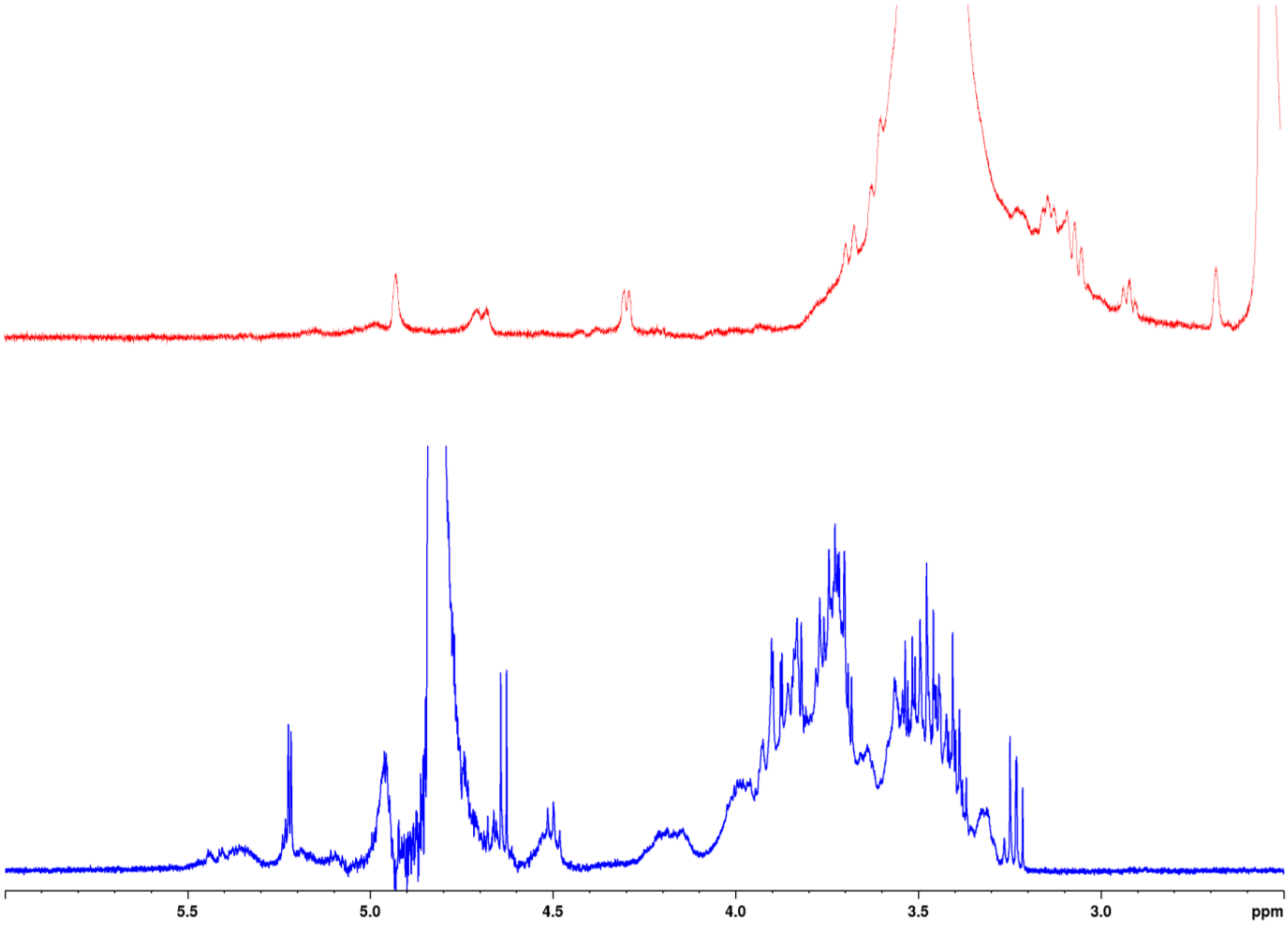

Figure S69. ^{1}H-NMR spectra stack of dry-down reaction of glucose in the absence (blue) or presence (red) of $FeCl_2$ under acidified environment. Solutions were prepared following drying at 50˚C for seven days. The products were dissolved in DMSO-d_6.

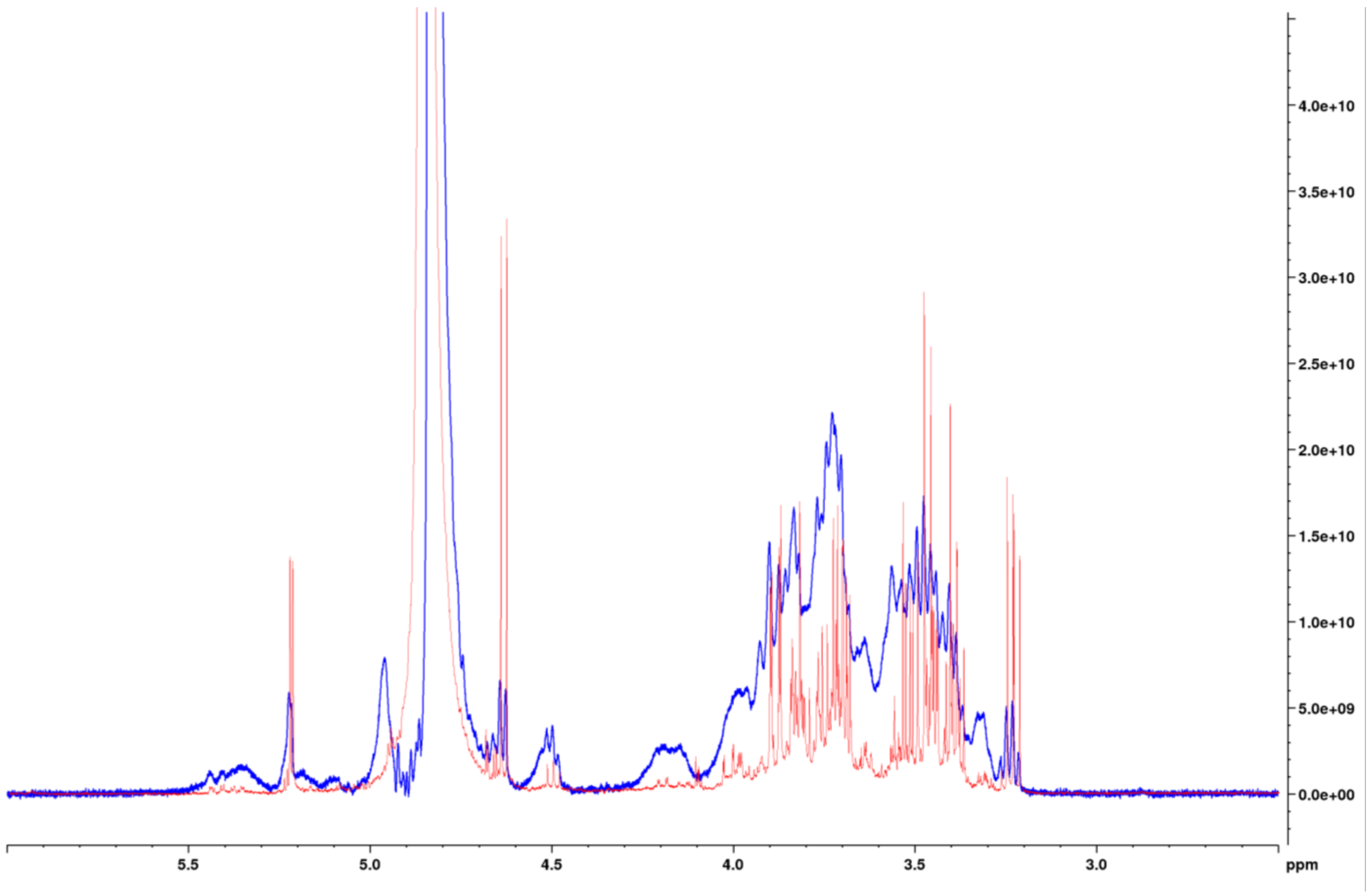

Figure S70. ^{1}H-NMR spectra overlay of dry-down reaction of glucose in the absence (blue) or presence (red) of $AlCl_3$ under acidified environment. Solutions were prepared following drying at 50˚C for seven days. The products were dissolved in D_2O buffer (pH=2.66).

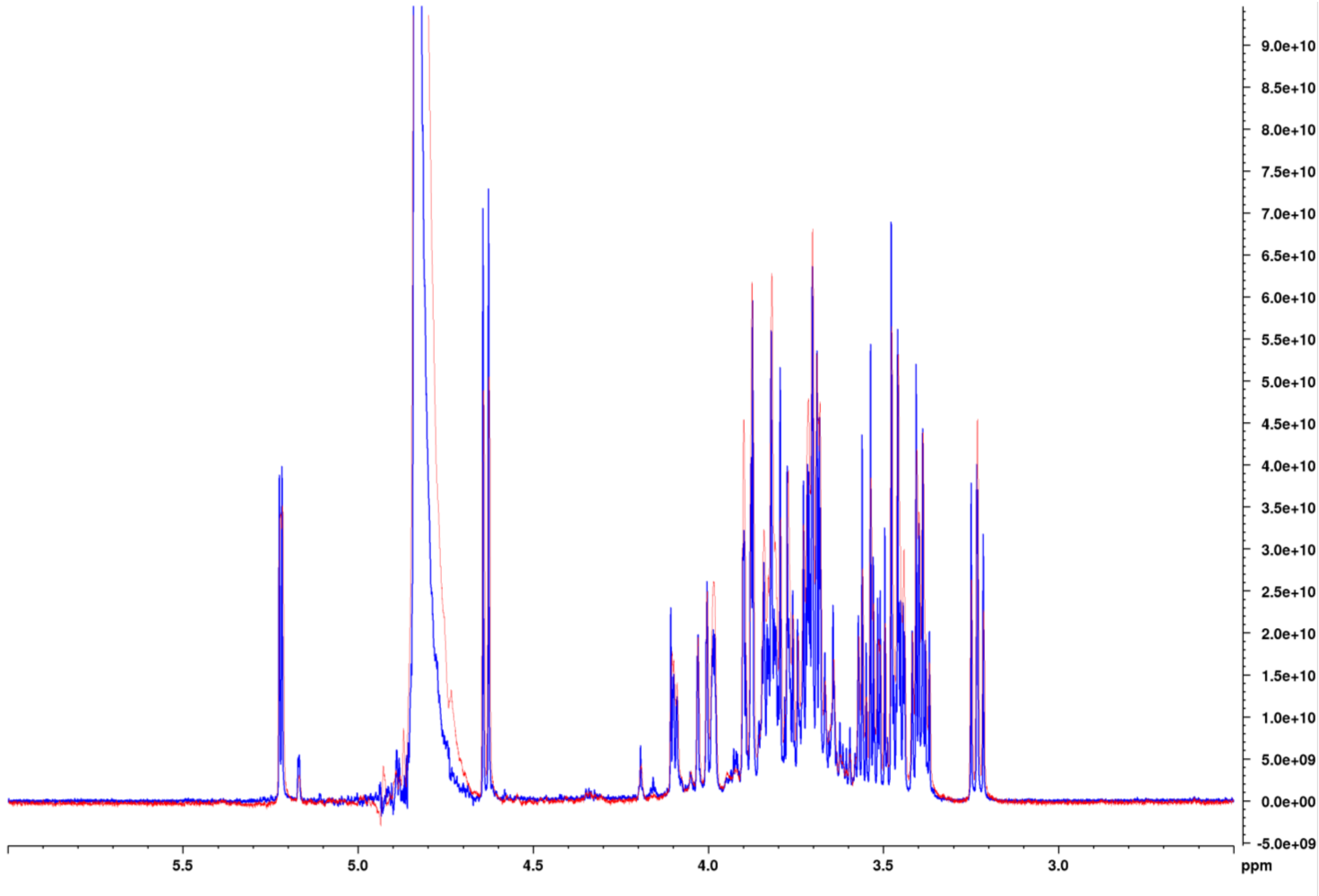

Figure S71. ^{1}H-NMR spectra overlay of dry-down reaction of glucose in the absence (blue) or presence (red) of LiCl upon addition of NaOH. Solutions were prepared following drying at 50˚C for seven days. The products were dissolved in D_2O buffer (pH=2.66).

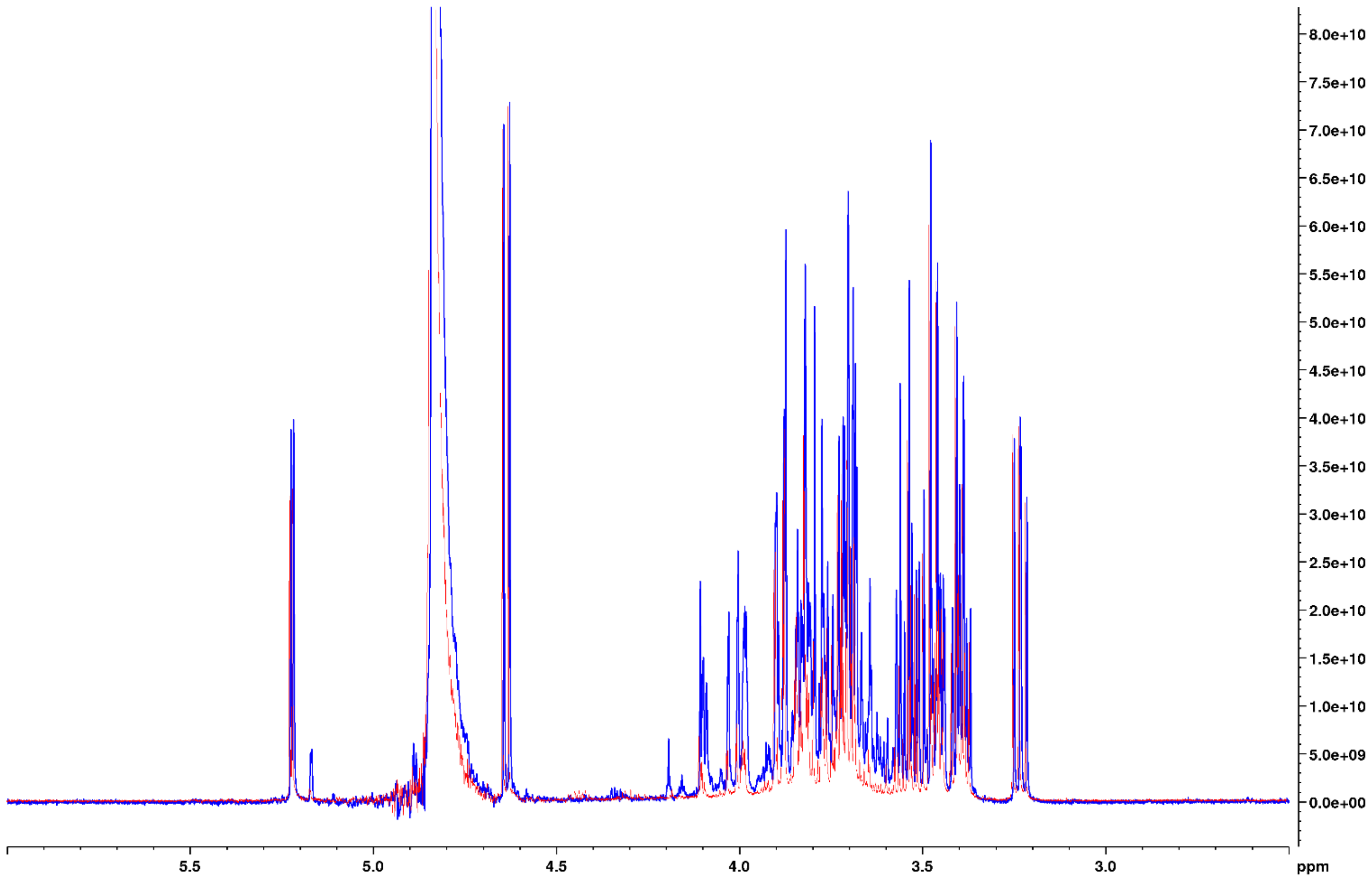

Figure S72. ^{1}H-NMR spectra overlay of dry-down reaction of glucose in the absence (blue) or presence (red) of $ZnCl_2$ upon addition of NaOH. Solutions were prepared following drying at 50˚C for seven days. The products were dissolved in D_2O buffer (pH=2.66).

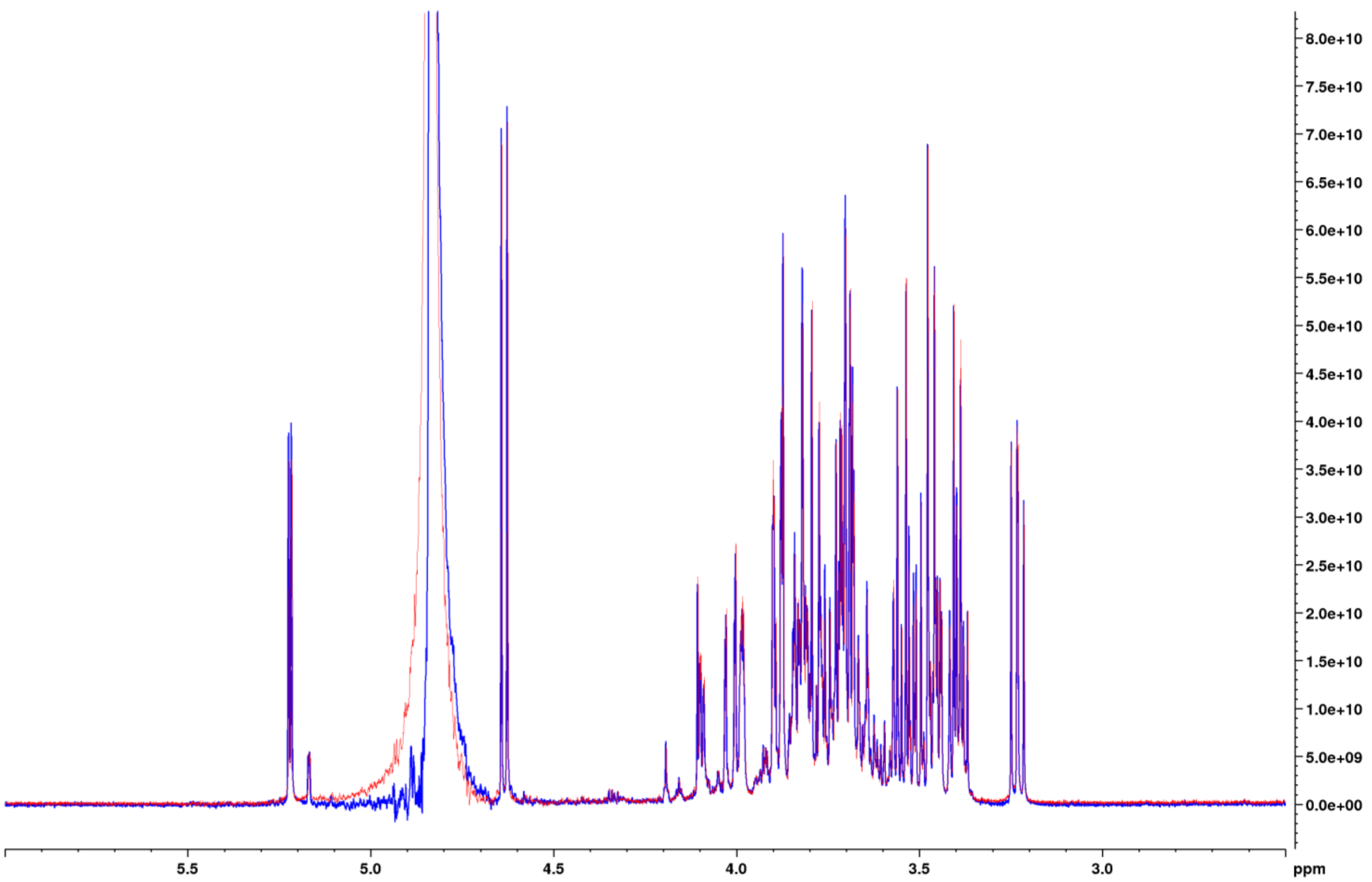

Figure S73. ^{1}H-NMR spectra overlay of dry-down reaction of glucose in the absence (blue) or presence (red) of NaCl upon addition of NaOH. Solutions were prepared following drying at 50˚C for seven days. The products were dissolved in D_2O buffer (pH=2.66).

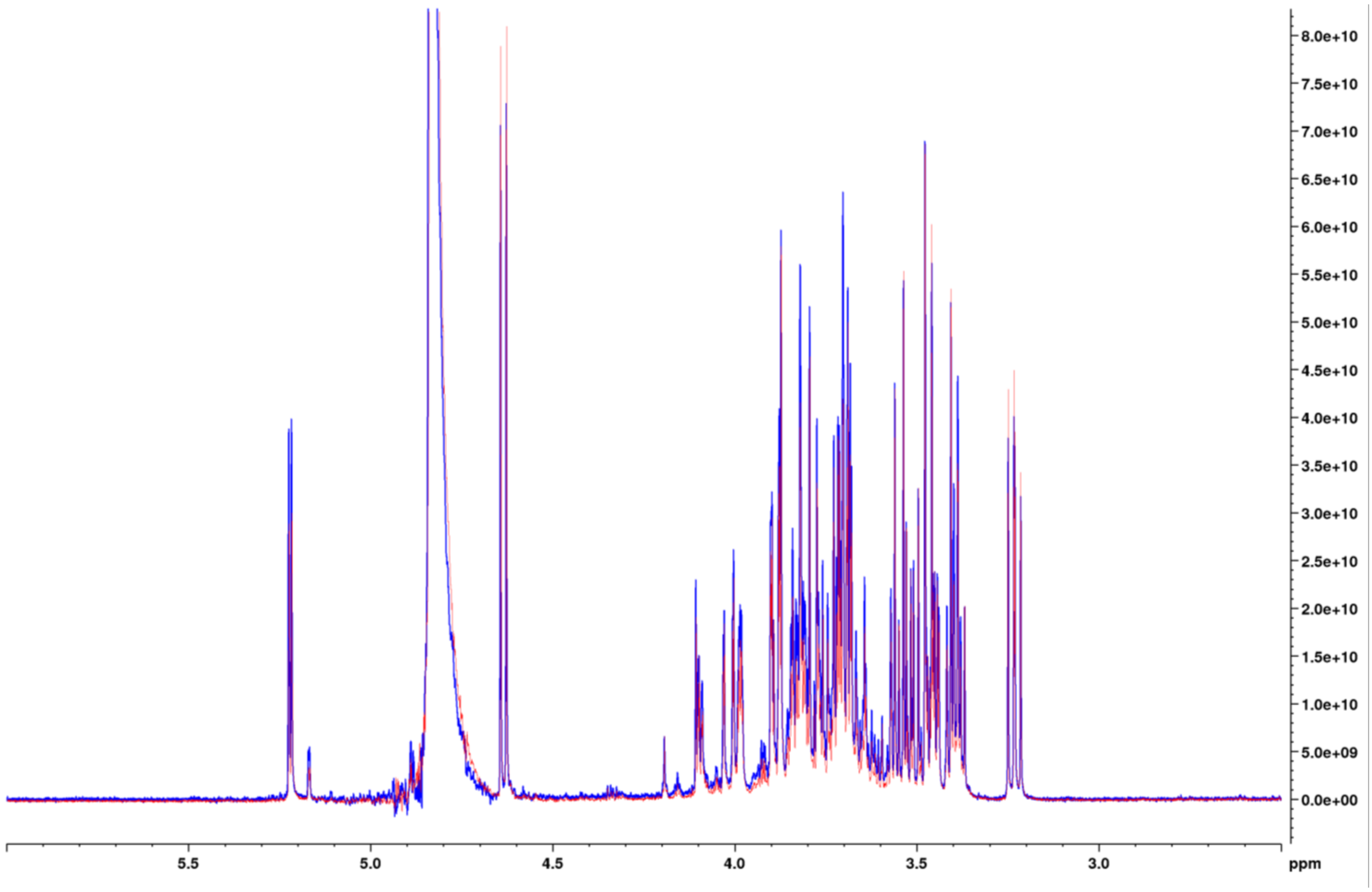

Figure S74. ^{1}H-NMR spectra overlay of dry-down reaction of glucose in the absence (blue) or presence (red) of KCl upon addition of NaOH. Solutions were prepared following drying at 50˚C for seven days. The products were dissolved in D_2O buffer (pH=2.66).

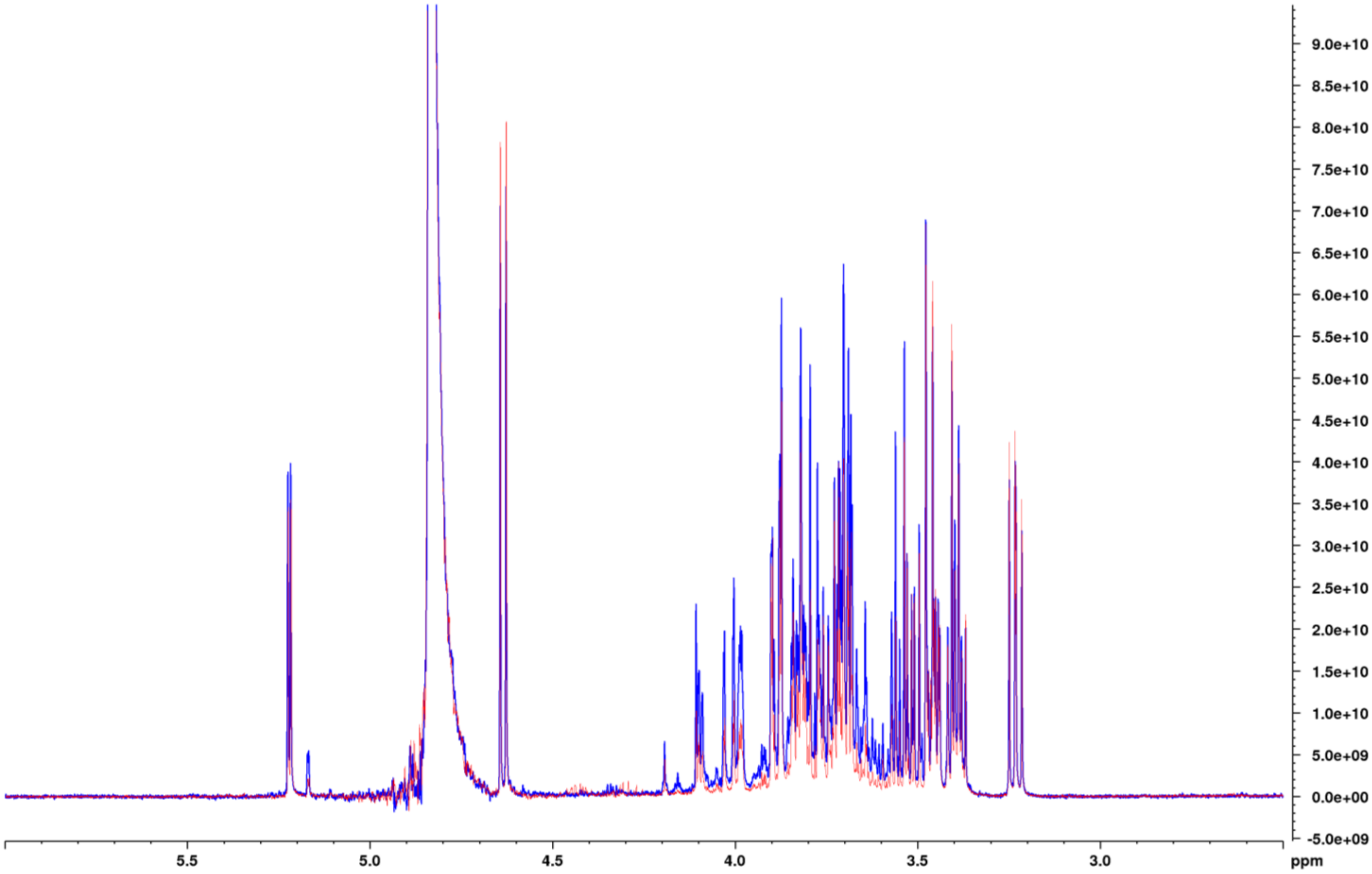

Figure S75. ^{1}H-NMR spectra overlay of dry-down reaction of glucose in the absence (blue) or presence (red) of $MgCl_2$ upon addition of NaOH. Solutions were prepared following drying at 50˚C for seven days. The products were dissolved in D_2O buffer (pH=2.66).

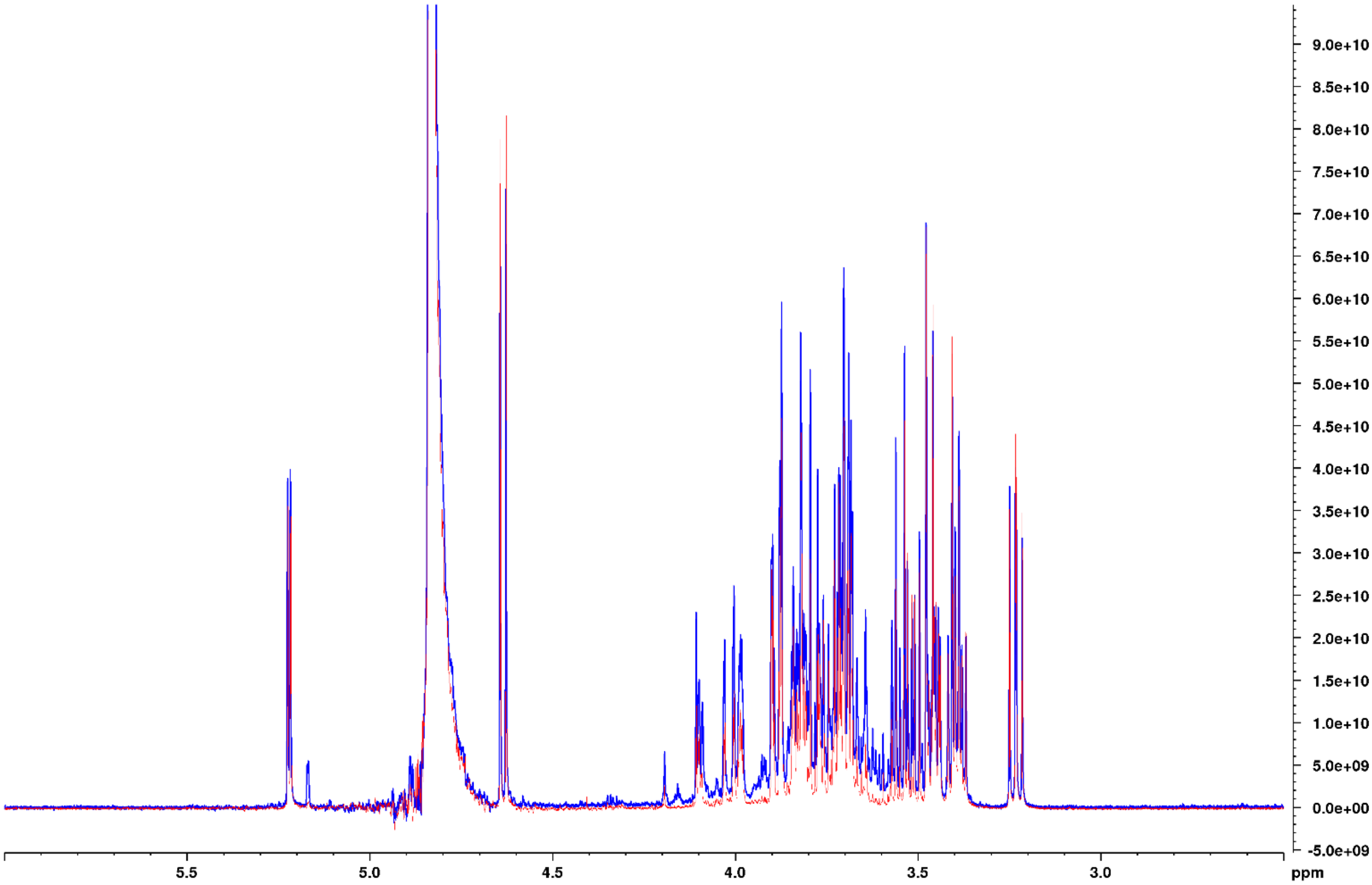

Figure S76. ^{1}H-NMR spectra overlay of dry-down reaction of glucose in the absence (blue) or presence (red) of $CaCl_2$ upon addition of NaOH. Solutions were prepared following drying at 50˚C for seven days. The products were dissolved in D_2O buffer (pH=2.66).

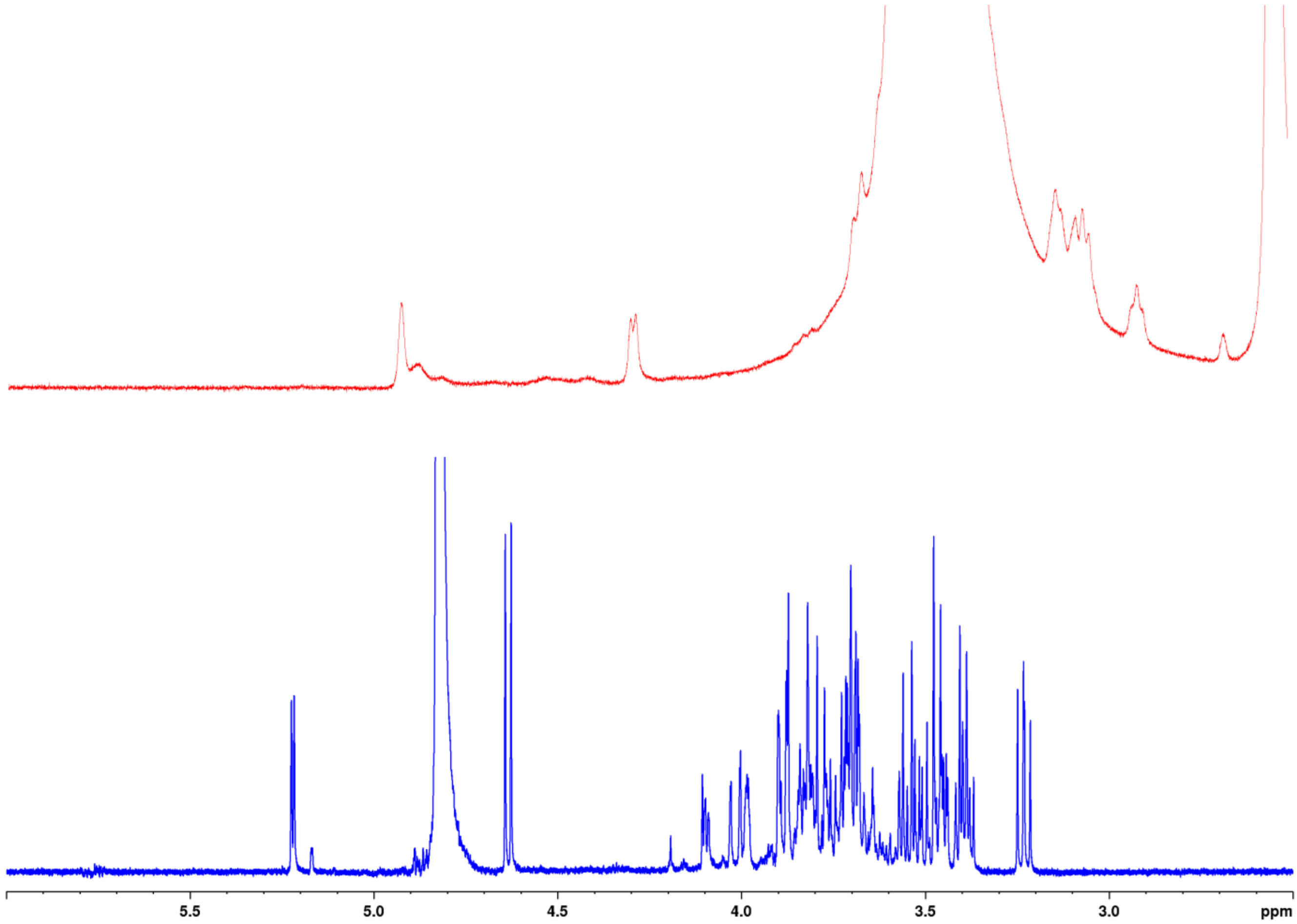

Figure S77. ^{1}H-NMR spectra stack of dry-down reaction of glucose in the absence (blue) or presence (red) of $MnCl_2$ upon addition of NaOH. Solutions were prepared following drying at 50˚C for seven days. The products were dissolved in DMSO-d_6.

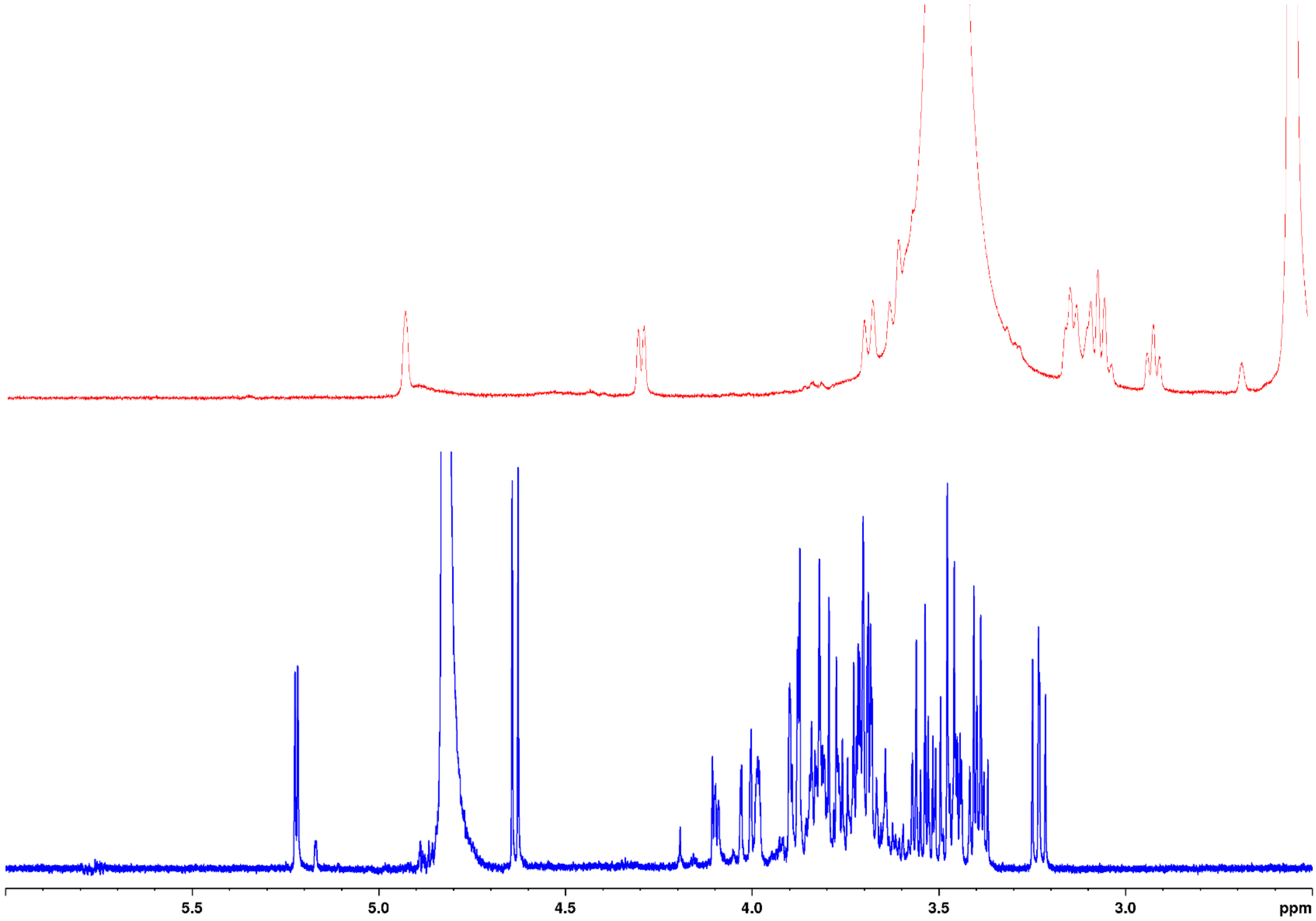

Figure S78. ^{1}H-NMR spectra stack of dry-down reaction of glucose in the absence (blue) or presence (red) of $FeCl_2$ upon addition of NaOH. Solutions were prepared following drying at 50˚C for seven days. The products were dissolved in DMSO-d_6.

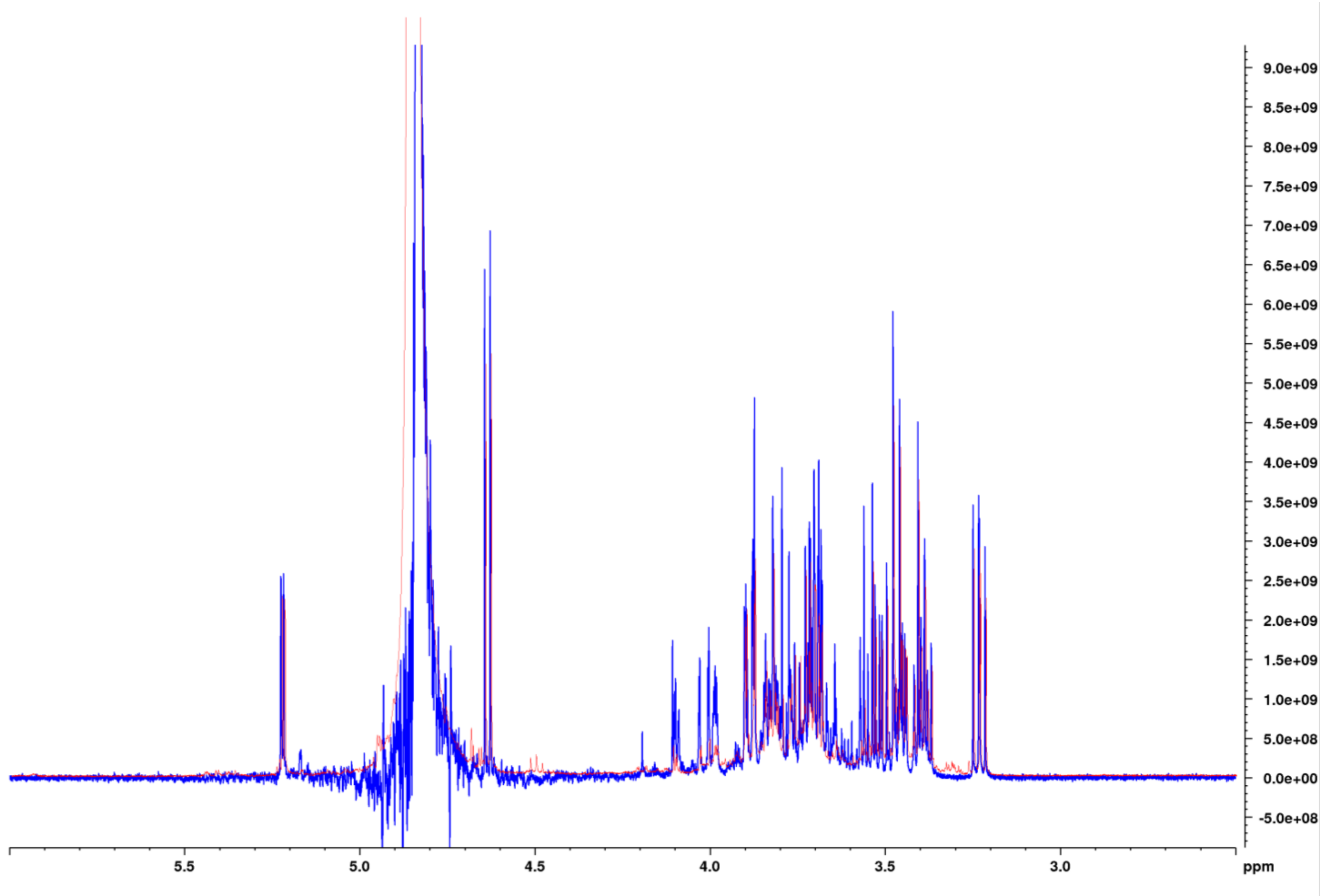

Figure S79. ^1H-NMR spectra overlay of dry-down reaction of glucose in the absence (blue) or presence (red) of $AlCl_3$ upon addition of NaOH. Solutions were prepared following drying at 50˚C for seven days. The products were dissolved in D_2O buffer (pH=2.66).

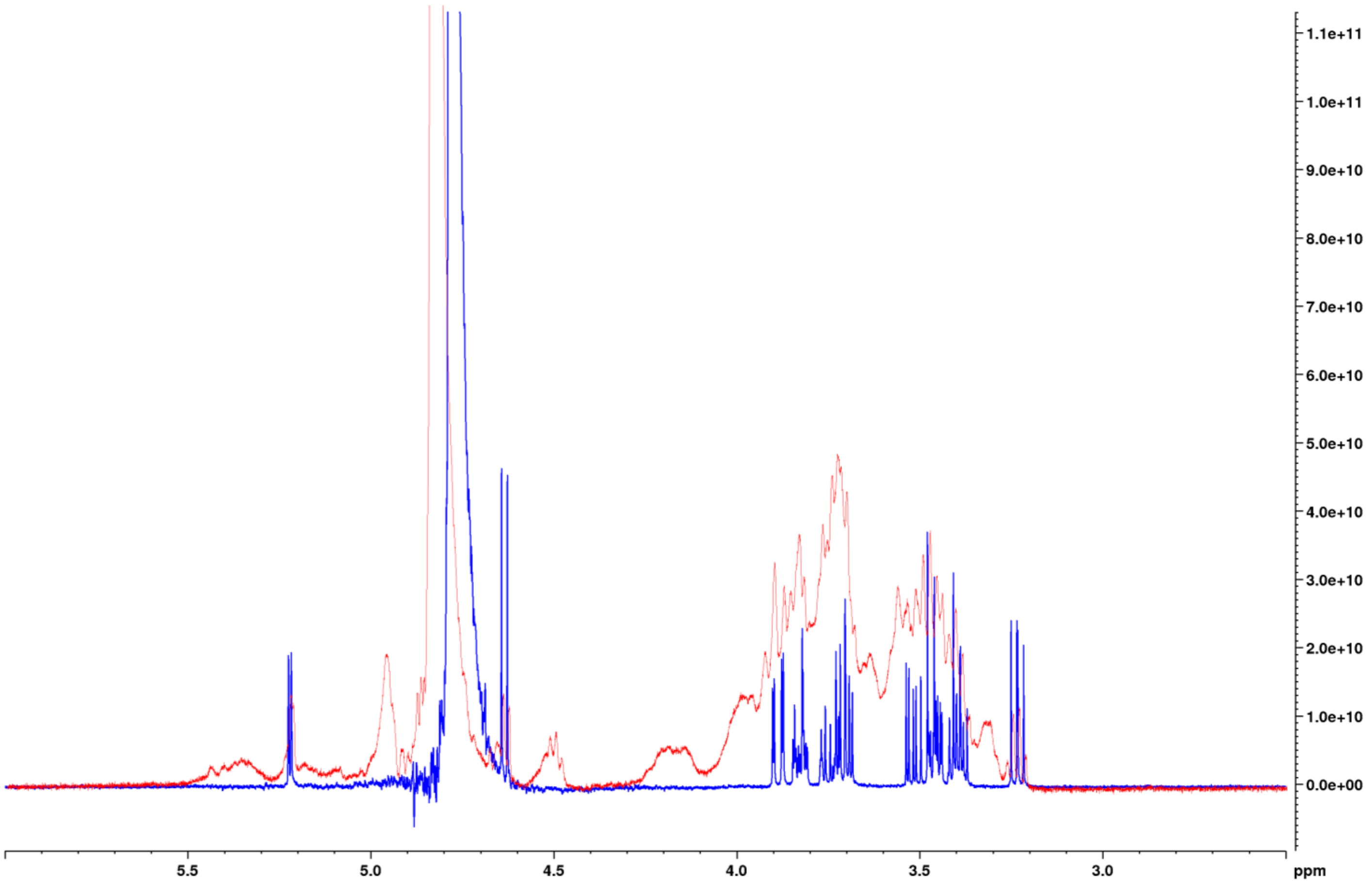

Figure S80. Overlay of ^{1}H-NMR spectra of a fresh (blue) or a dry-down reaction at 50˚C for seven days under acidified conditions (red) of glucose in the absence of metals. Samples were dissolved in D_2O buffer (pH=2.66).

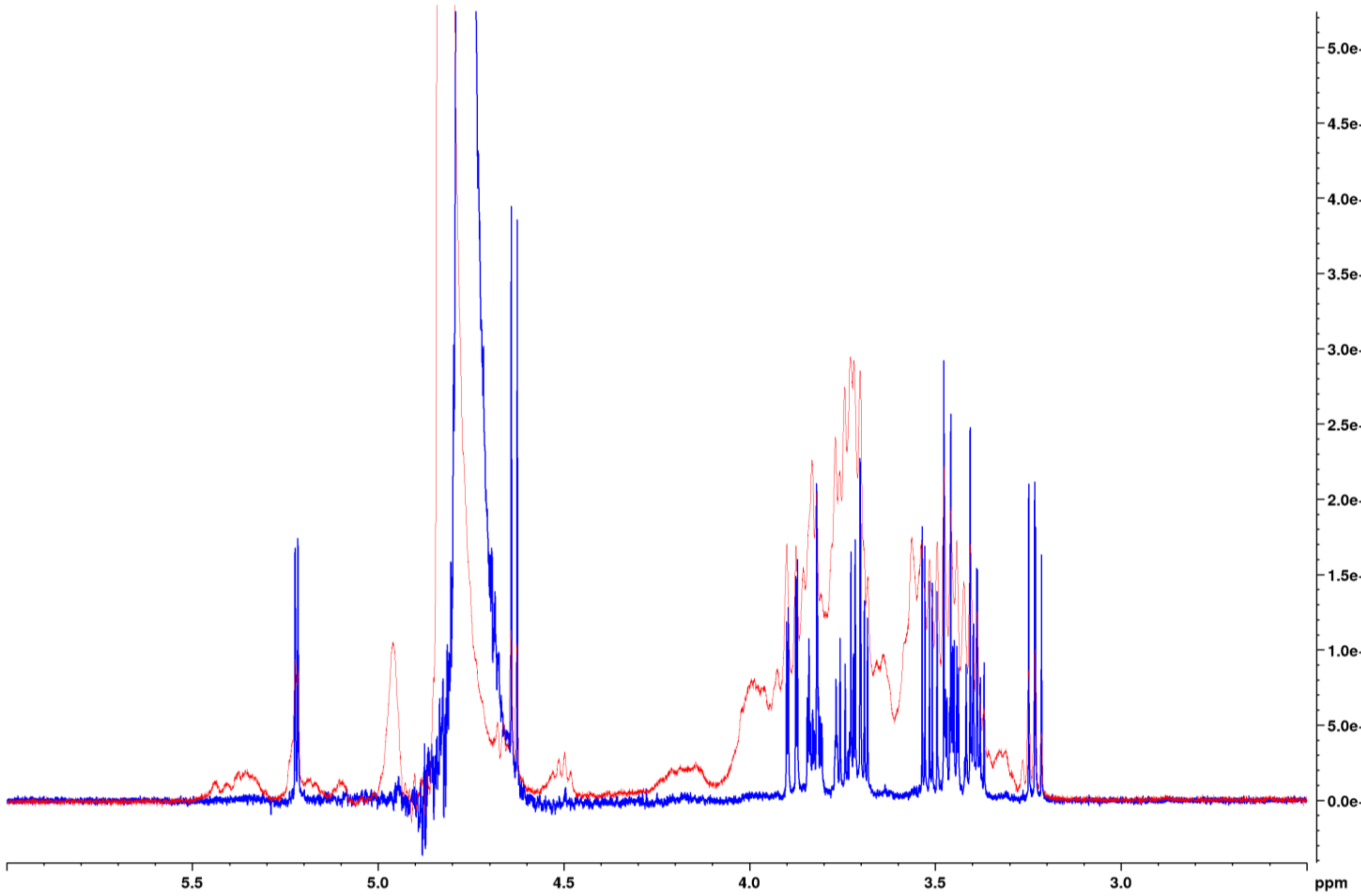

Figure S81.Overlay of ^{1}H-NMR spectra of a fresh (blue) or a dry-down reaction at 50˚C for seven days under acidified conditions (red) of glucose in the presence of LiCl. Samples were dissolved in D_2O buffer (pH=2.66).

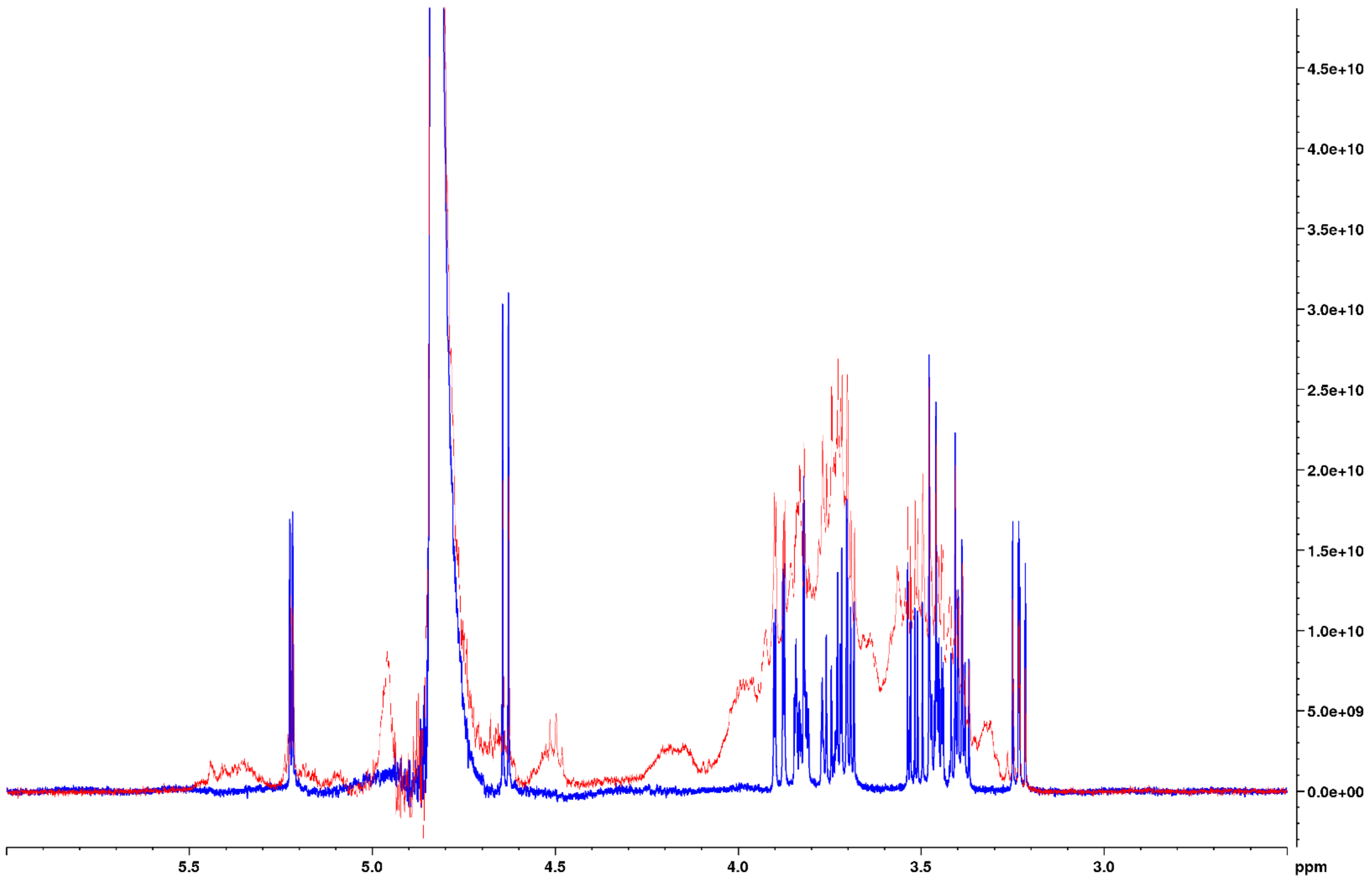

Figure S82. Overlay of ^{1}H-NMR spectra of a fresh (blue) or a dry-down reaction at 50˚C for seven days under acidified conditions (red) of glucose in the presence of $ZnCl_2$. Samples were dissolved in D_2O buffer (pH=2.66).

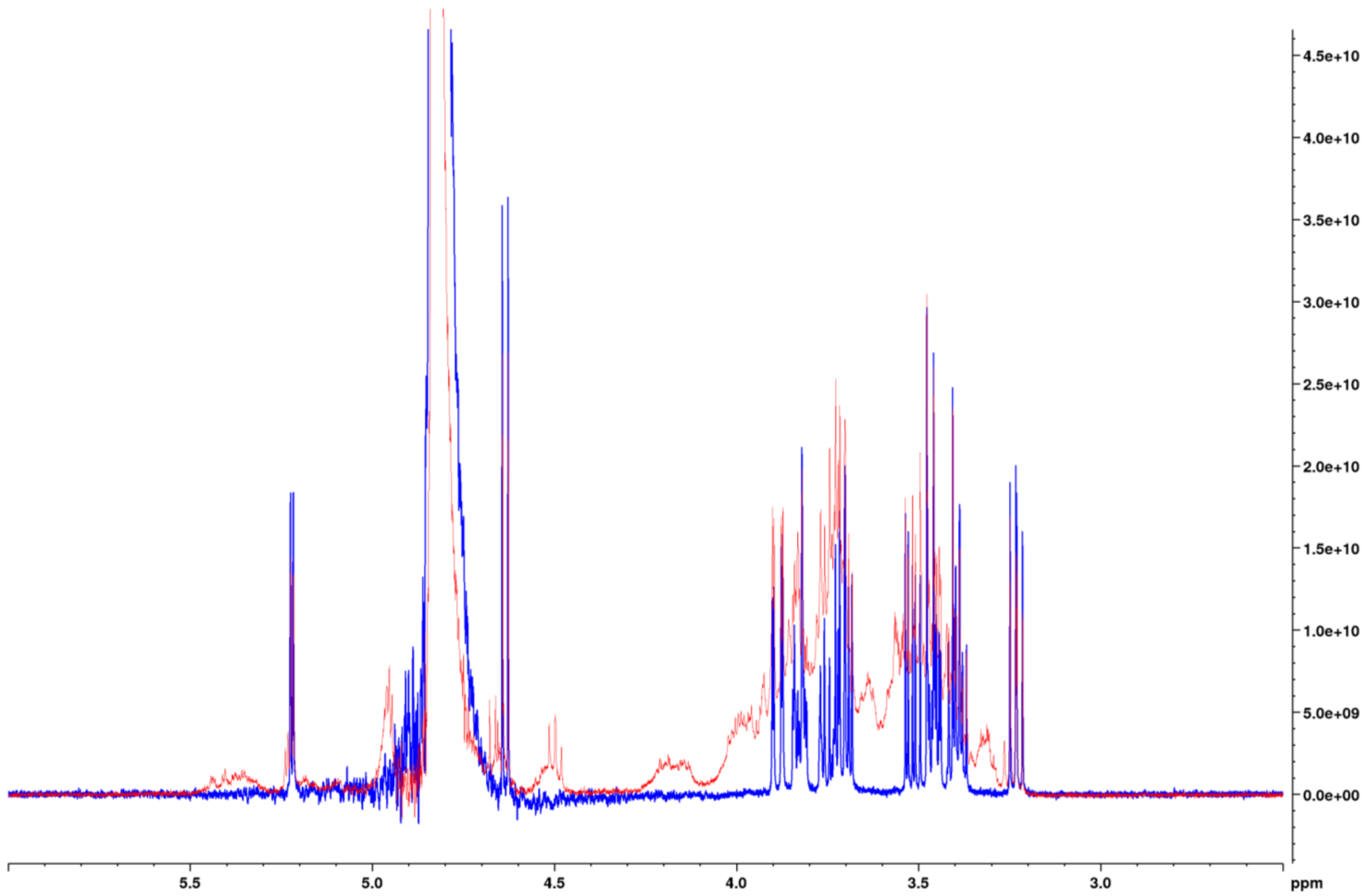

Figure S83. Overlay of ^{1}H-NMR spectra of a fresh (blue) or a dry-down reaction at 50˚C for seven days under acidified conditions (red) of glucose in the presence of NaCl. Samples were dissolved in D_2O buffer (pH=2.66).

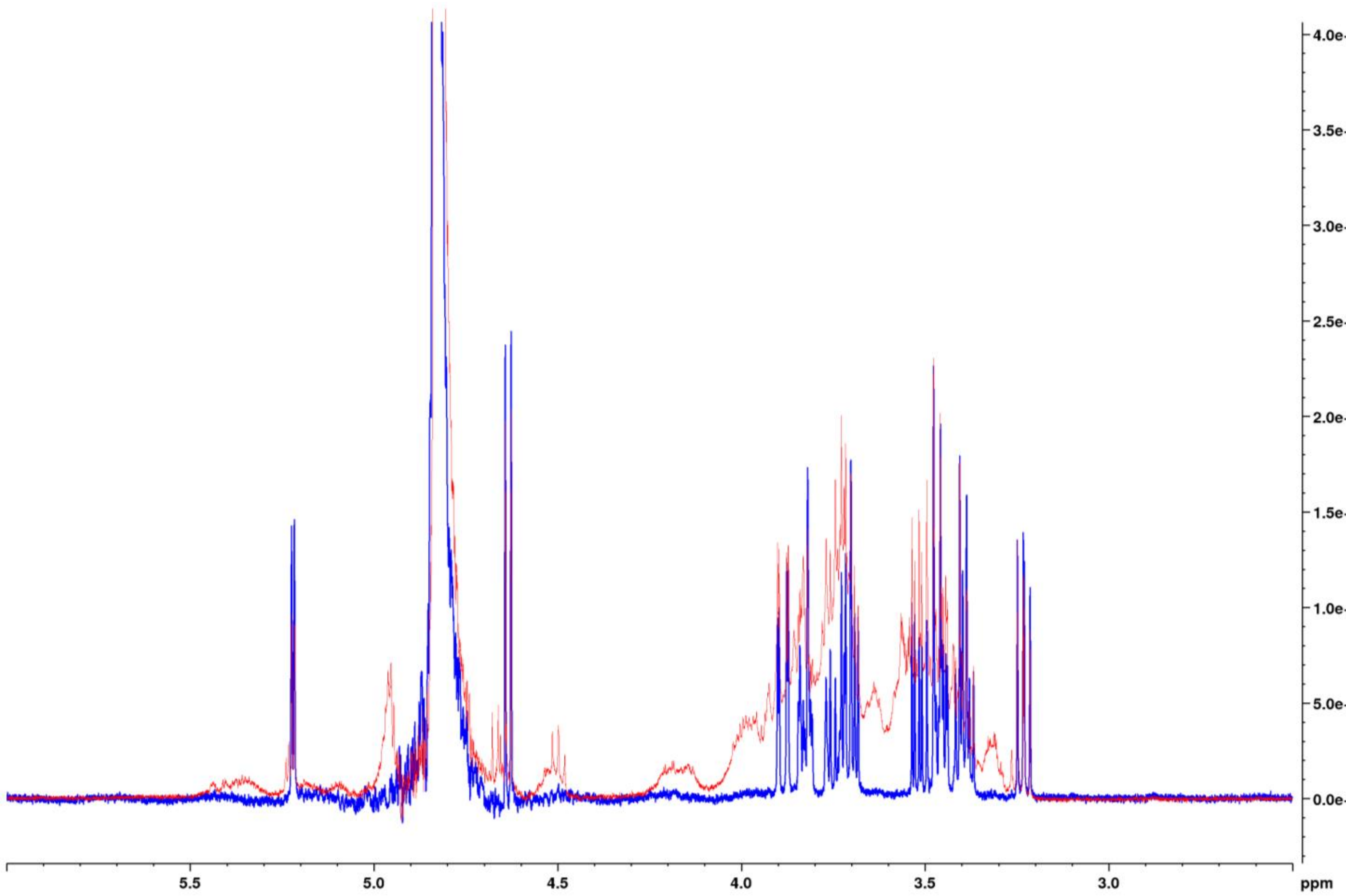

Figure S84. Overlay of ^1H-NMR spectra of a fresh (blue) or a dry-down reaction at 50˚C for seven days under acidified conditions (red) of glucose in the presence of KCl. Samples were dissolved in D_2O buffer (pH=2.66).

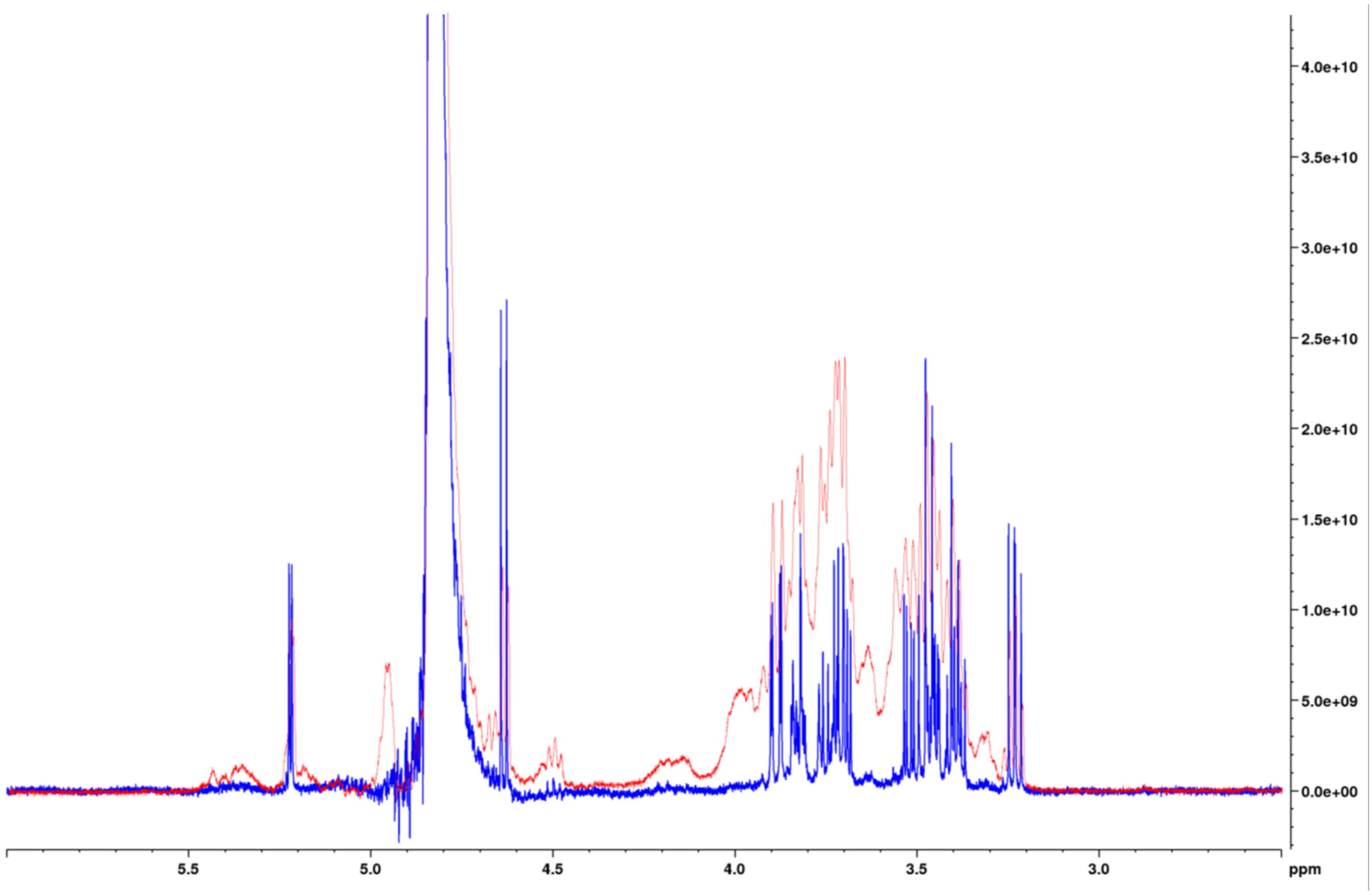

Figure S85. Overlay of ^{1}H-NMR spectra of a fresh (blue) or a dry-down reaction at 50˚C for seven days under acidified conditions (red) of glucose in the presence of $MgCl_2$. Samples were dissolved in D_2O buffer (pH=2.66).

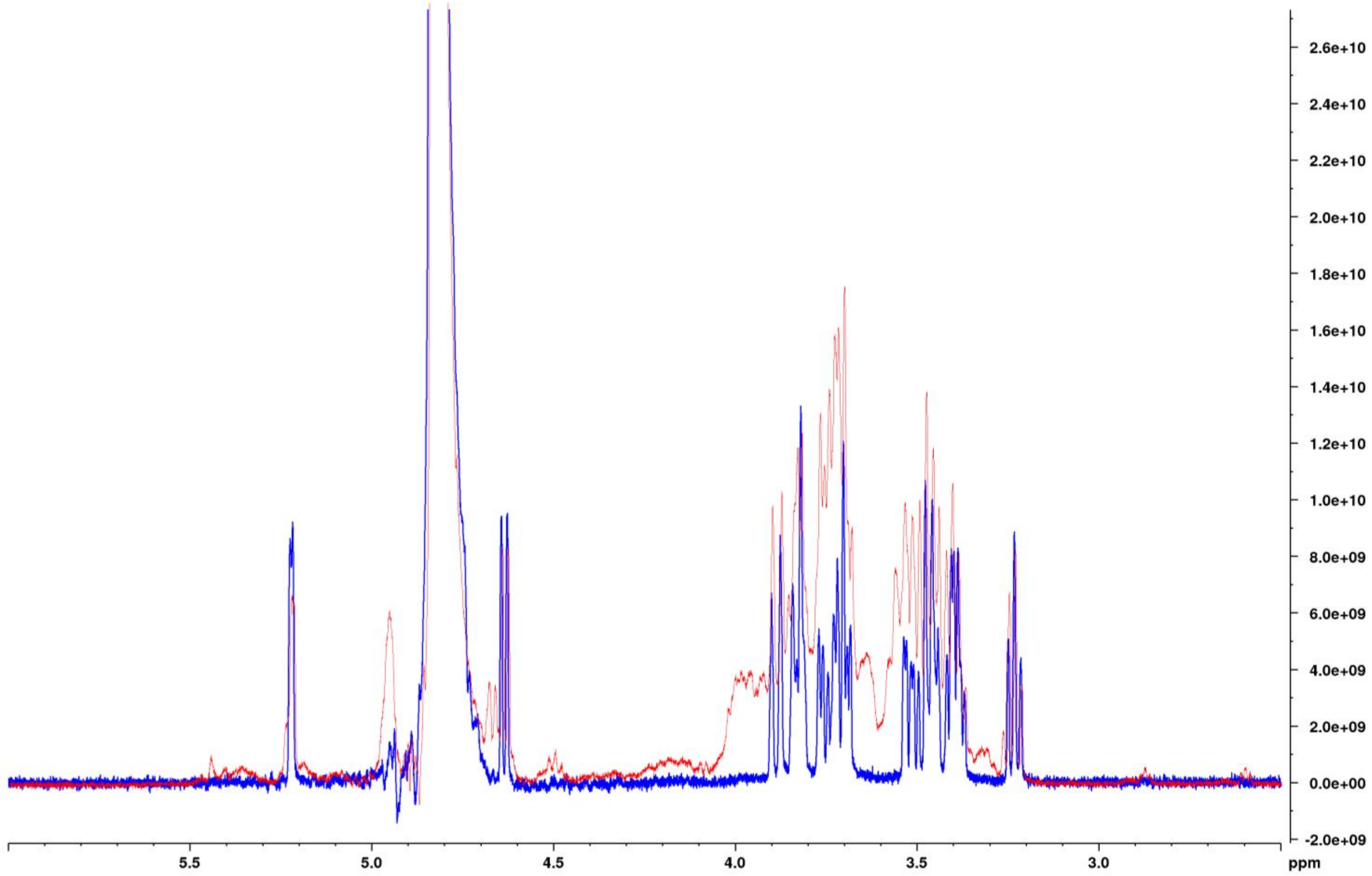

Figure S86. Overlay of ¹H-NMR spectra of a fresh (blue) or a dry-down reaction at 50˚C for seven days under acidified conditions (red) of glucose in the presence of $CaCl_2$. Samples were dissolved in D_2O buffer (pH=2.66).

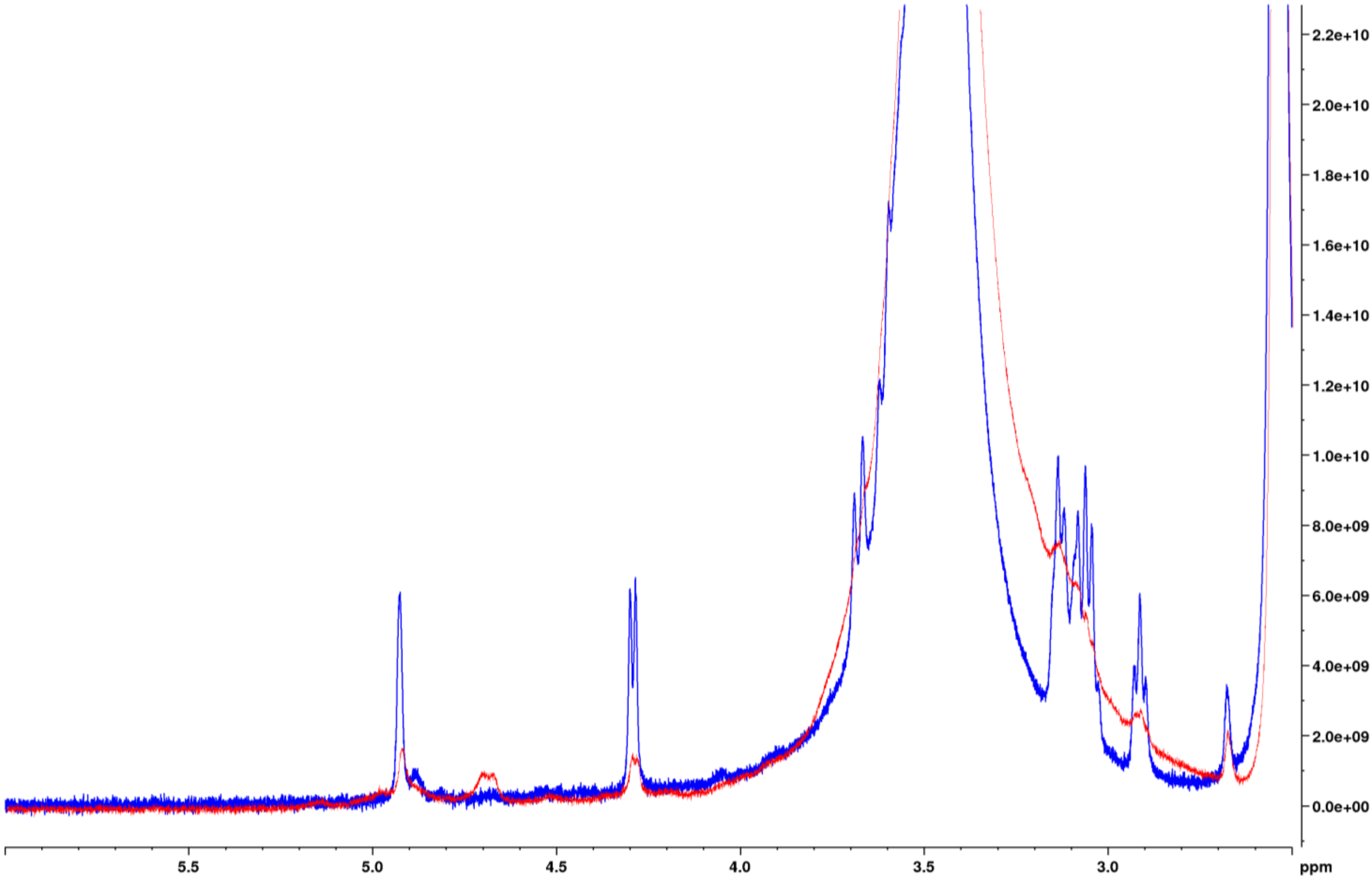

Figure S87. Overlay of ^{1}H-NMR spectra of a fresh (blue) or a dry-down reaction at 50˚C for seven days under acidified conditions (red) of glucose in the presence of $MnCl_2$. Samples were dissolved in DMSO-d_6.

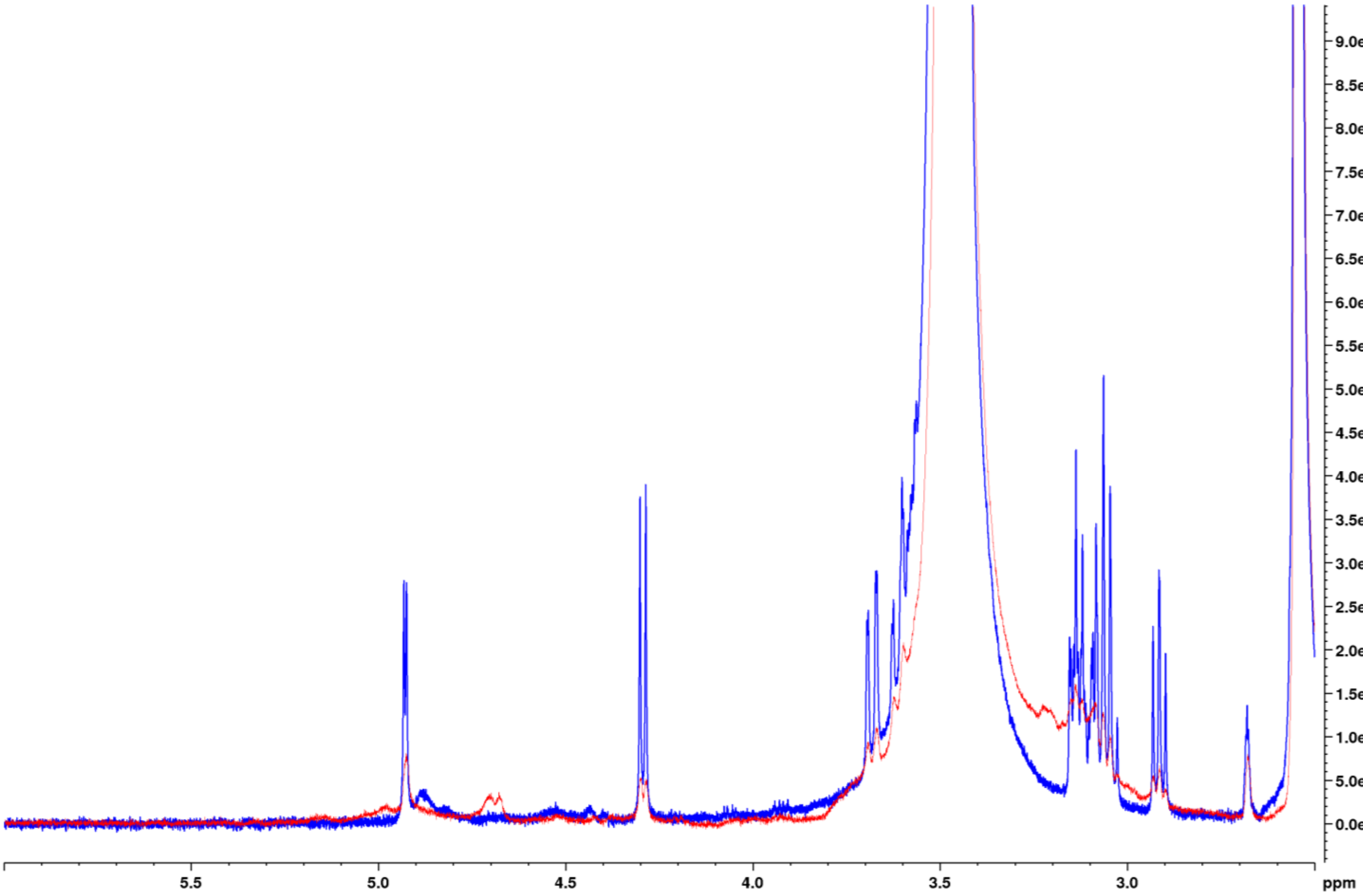

Figure S88. Overlay of ^{1}H-NMR spectra of a fresh (blue) or a dry-down reaction at 50˚C for seven days under acidified conditions (red) of glucose in the presence of $FeCl_2$. Samples were dissolved in DMSO-d_6.

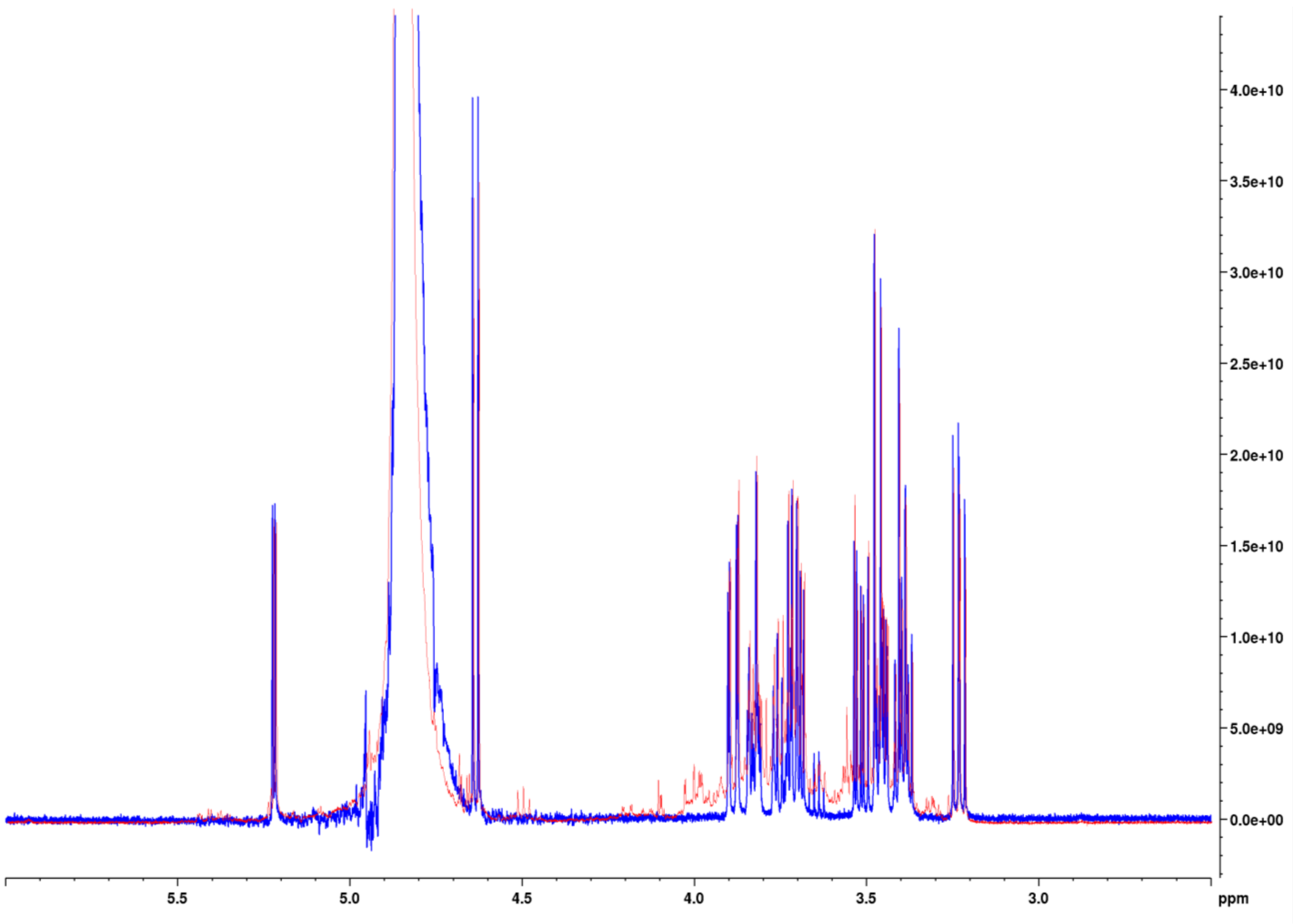

Figure S89. Overlay of ^{1}H-NMR spectra of a fresh (blue) or a dry-down reaction at 50˚C for seven days under acidified conditions (red) of glucose in the presence of $AlCl_3$. Samples were dissolved in DMSO-d_6.

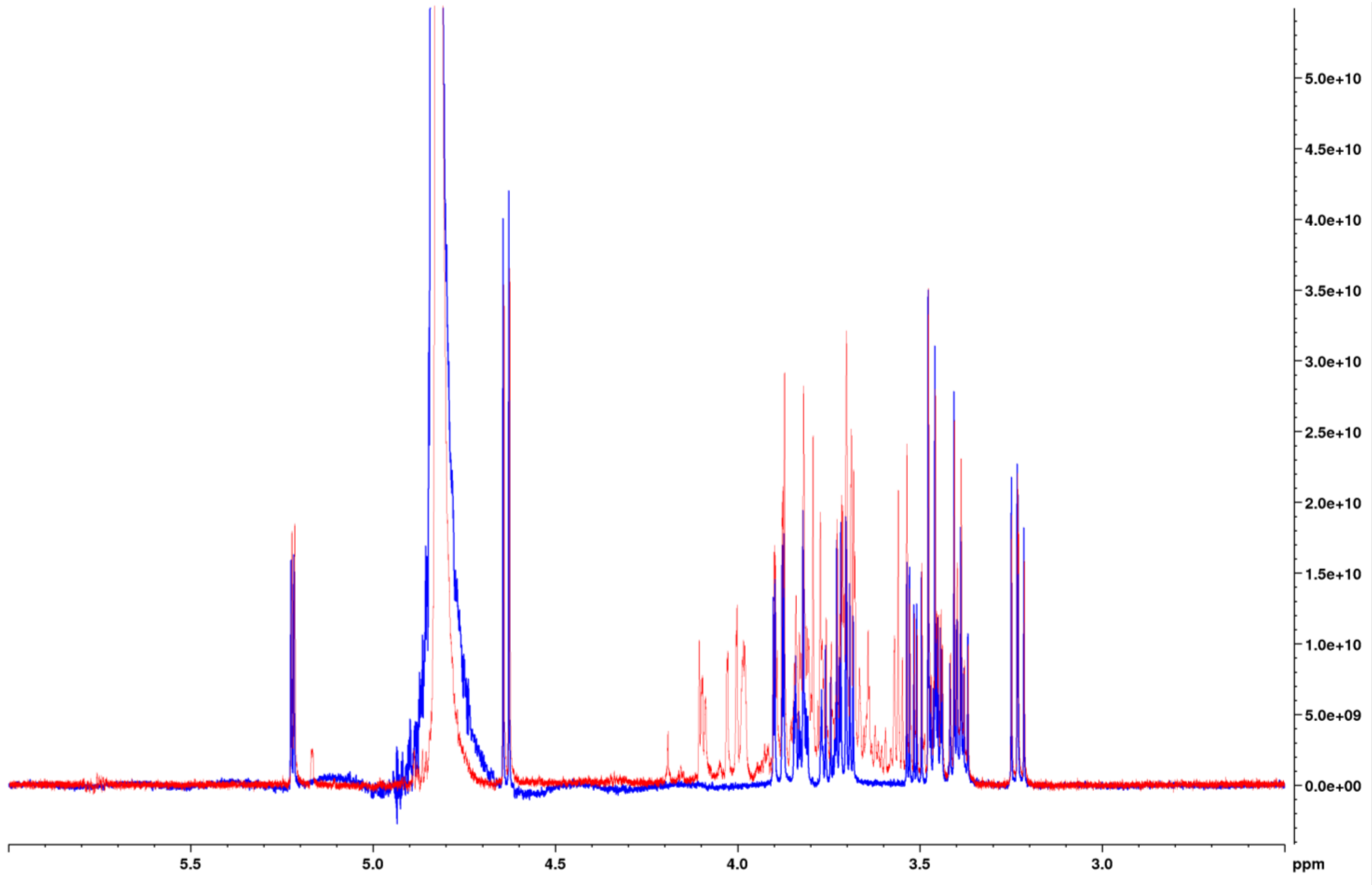

Figure S90. Overlay of ^{1}H-NMR spectra of a fresh (blue) or a dry-down reaction at 50˚C for seven days upon addition of NaOH (red) of glucose in the absence of metals. Samples were dissolved in D_2O buffer (pH=2.66).

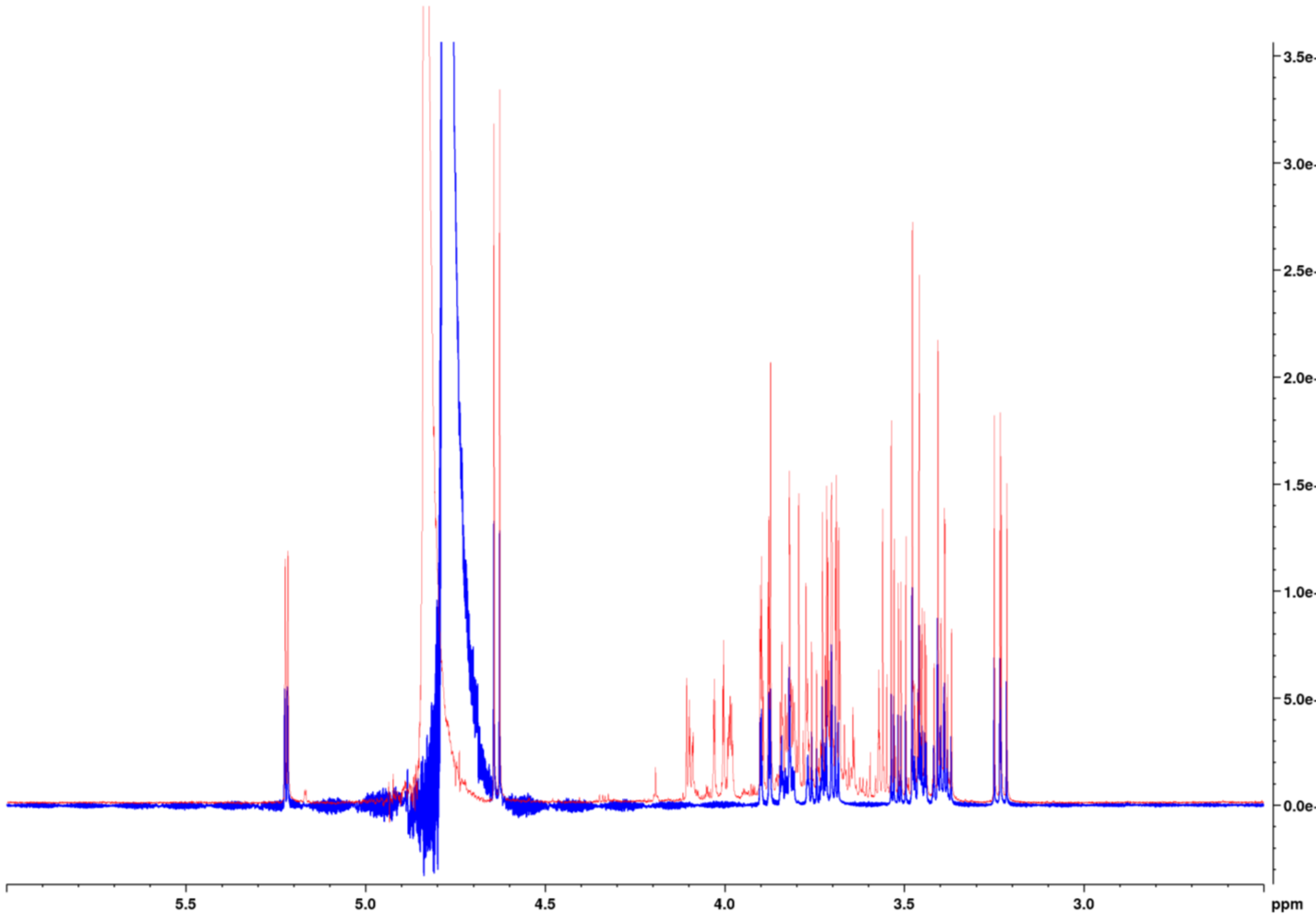

Figure S91. Overlay of ^{1}H-NMR spectra of a fresh (blue) or a dry-down reaction at 50˚C for seven days upon addition of NaOH (red) of glucose in the presence of LiCl. Samples were dissolved in D_2O buffer (pH=2.66).

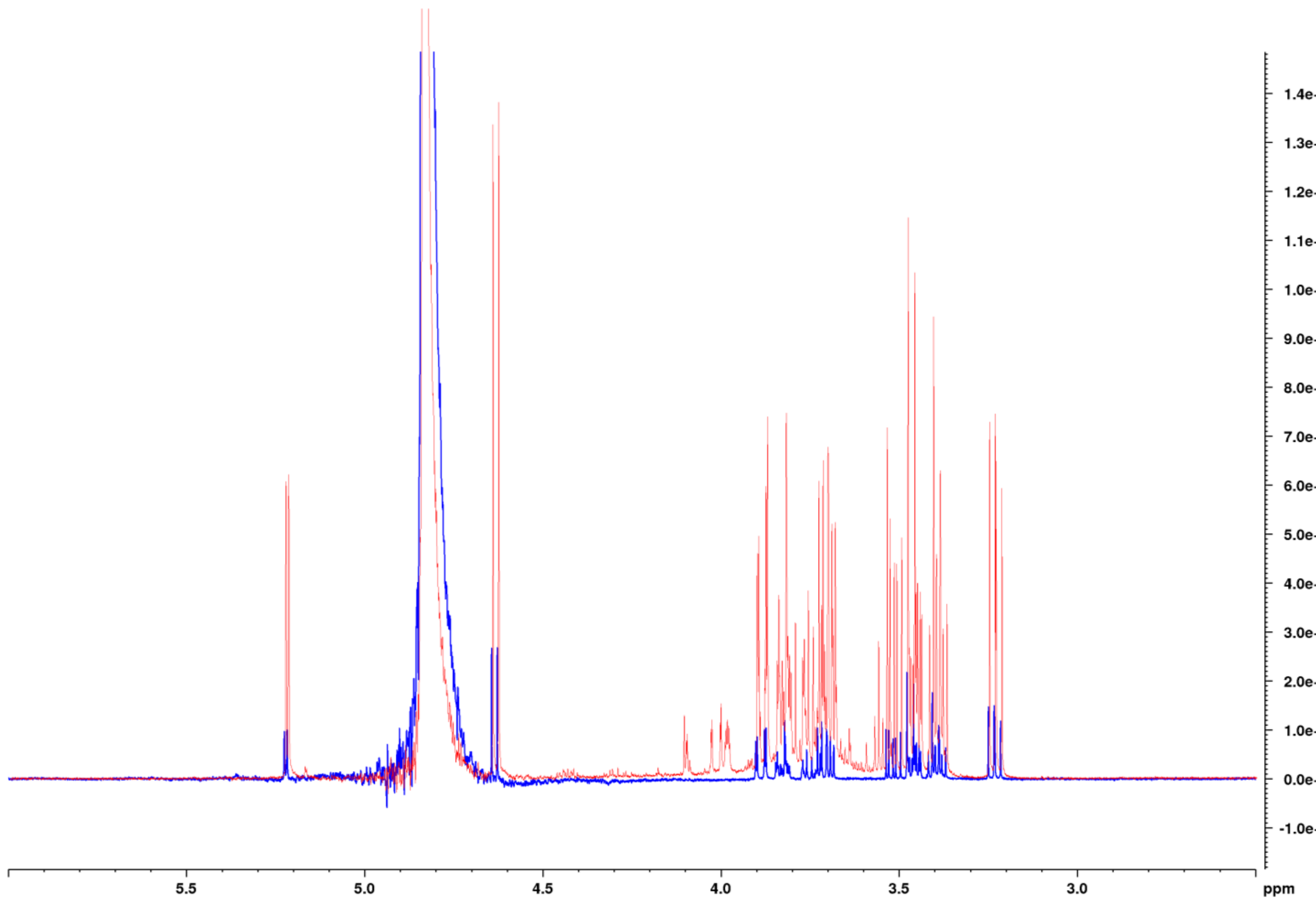

Figure S92. Overlay of ^{1}H-NMR spectra of a fresh (blue) or a dry-down reaction at 50˚C for seven days upon addition of NaOH (red) of glucose in the presence of $ZnCl_2$. Samples were dissolved in D_2O buffer (pH=2.66).

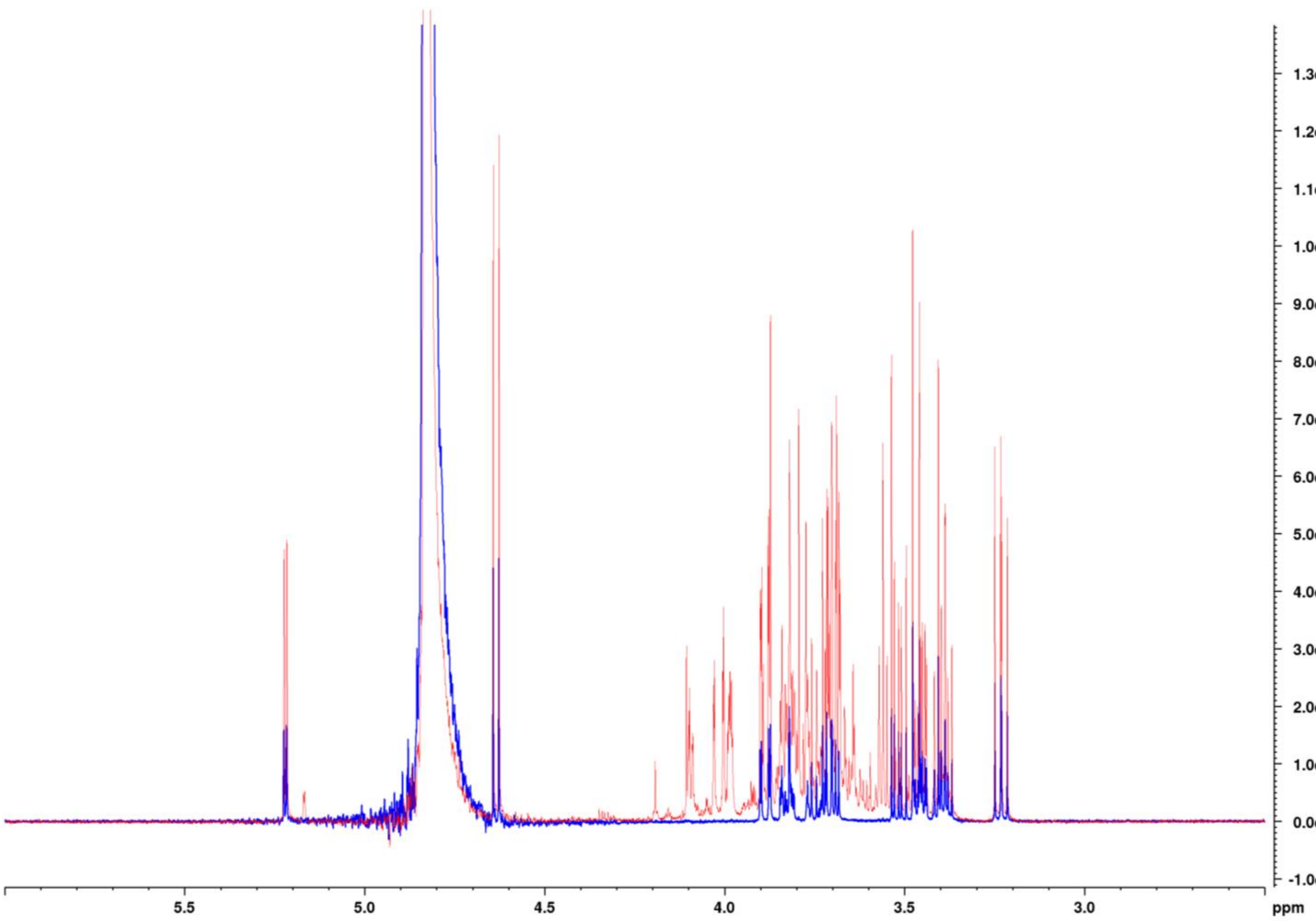

Figure S93. Overlay of ^{1}H-NMR spectra of a fresh (blue) or a dry-down reaction at 50˚C for seven days upon addition of NaOH (red) of glucose in the presence of NaCl. Samples were dissolved in D_2O buffer (pH=2.66).

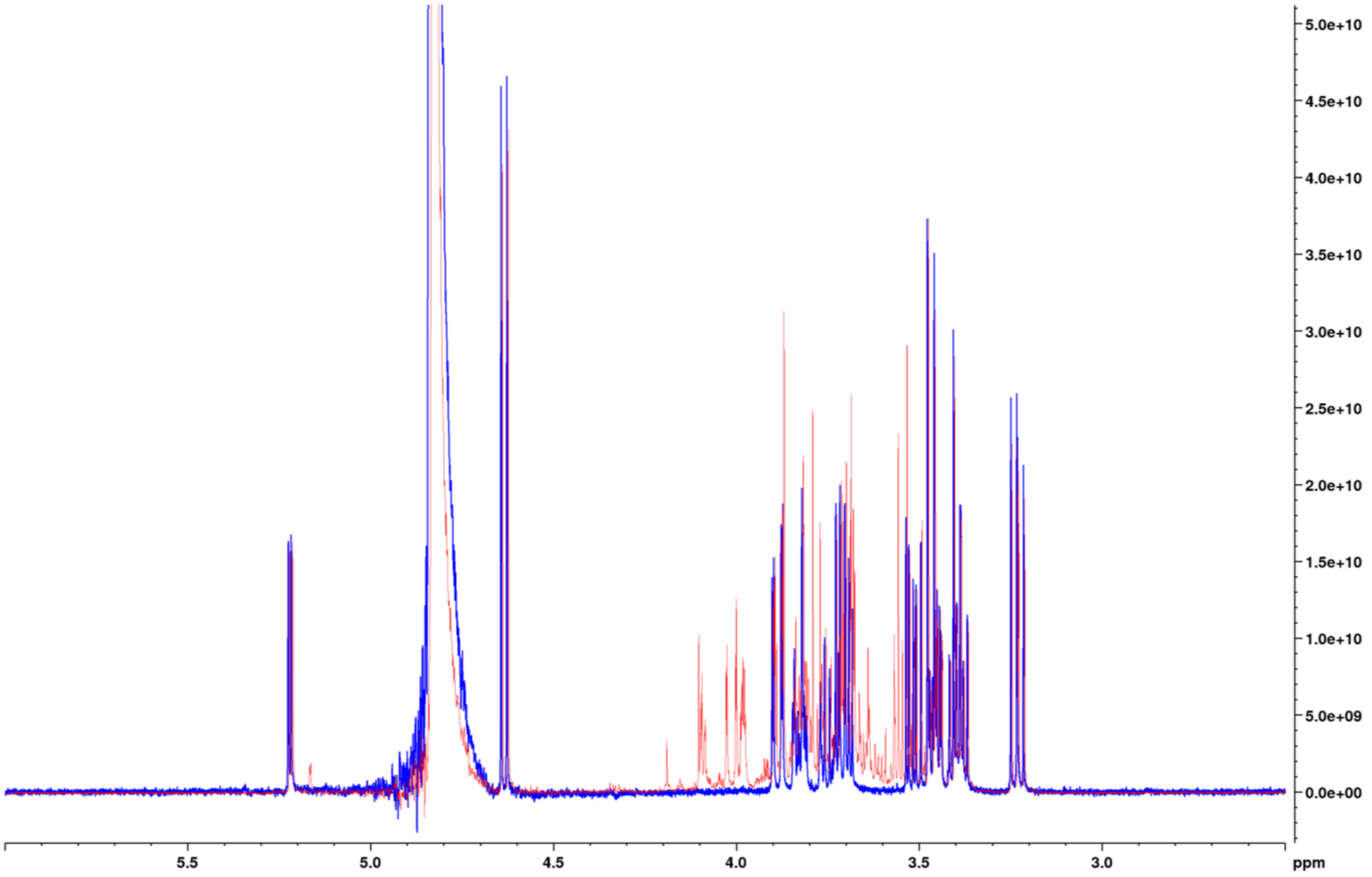

Figure S94. Overlay of ^{1}H-NMR spectra of a fresh (blue) or a dry-down reaction at 50˚C for seven days upon addition of NaOH (red) of glucose in the presence of KCl. Samples were dissolved in D_2O buffer (pH=2.66).

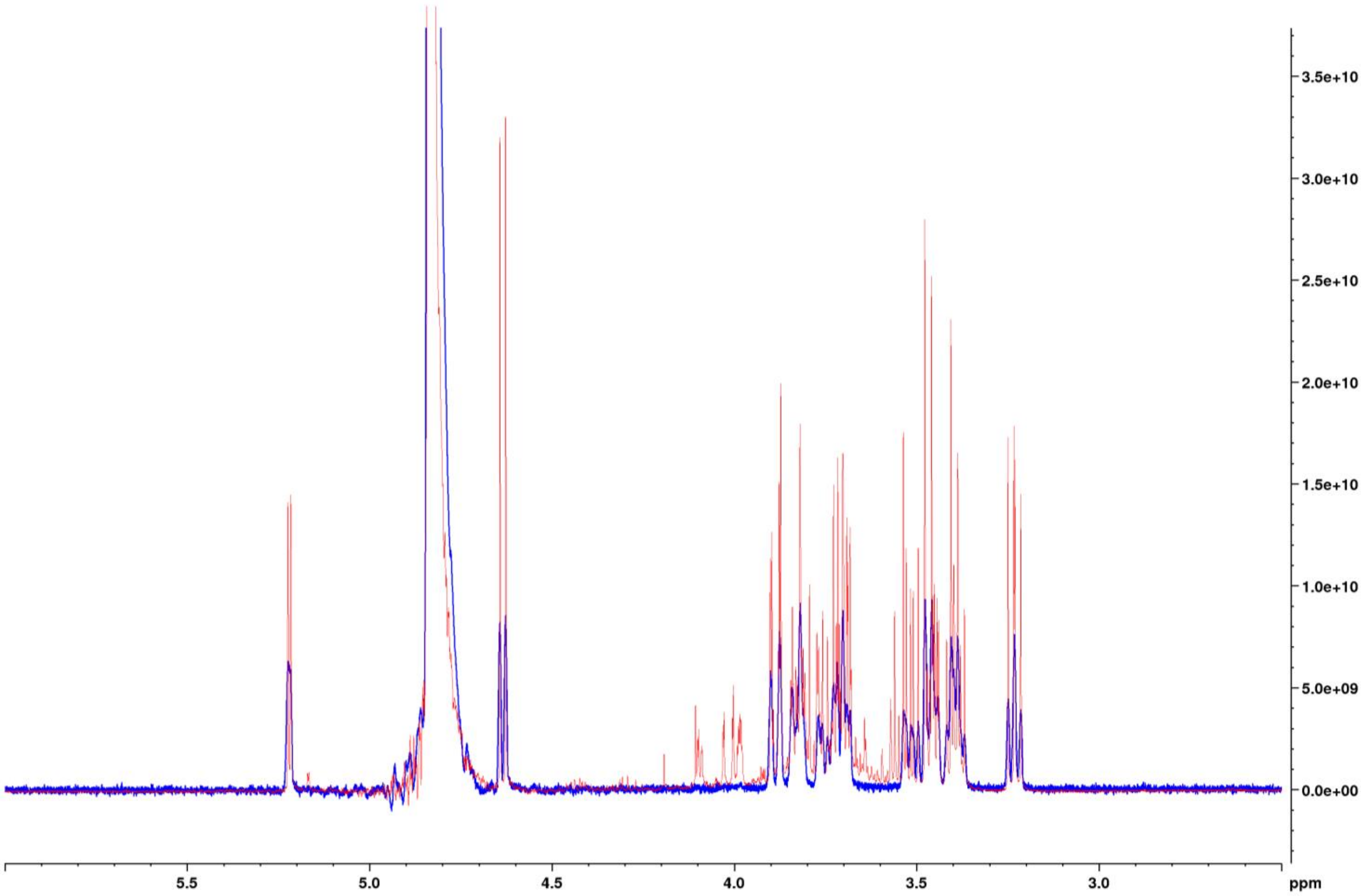

Figure S95. Overlay of ^{1}H-NMR spectra of a fresh (blue) or a dry-down reaction at 50˚C for seven days upon addition of NaOH (red) of glucose in the presence of $MgCl_2$. Samples were dissolved in D_2O buffer (pH=2.66).

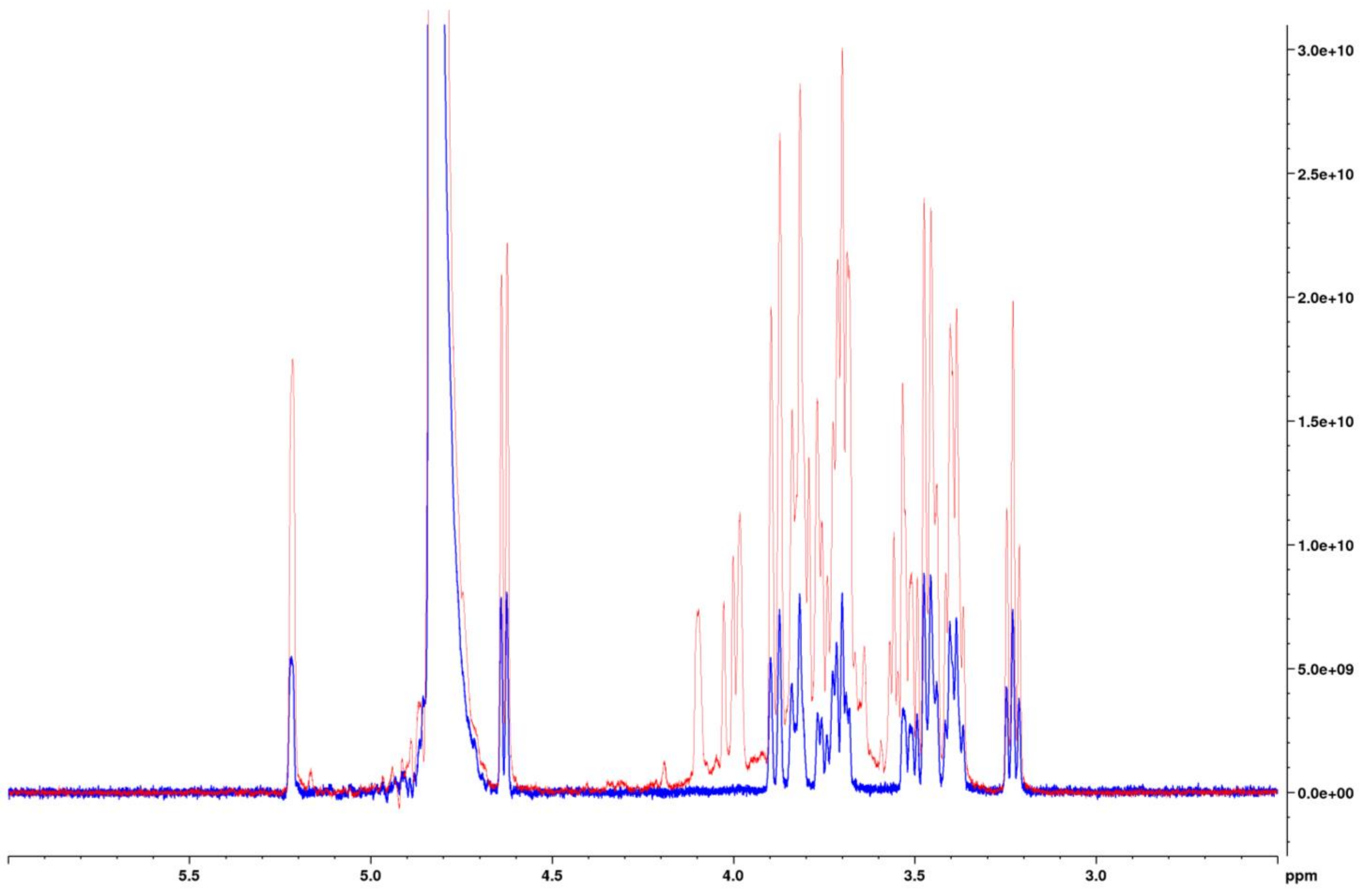

Figure S96. Overlay of ^{1}H-NMR spectra of a fresh (blue) or a dry-down reaction at 50˚C for seven days upon addition of NaOH (red) of glucose in the presence of $CaCl_2$. Samples were dissolved in D_2O buffer (pH=2.66).

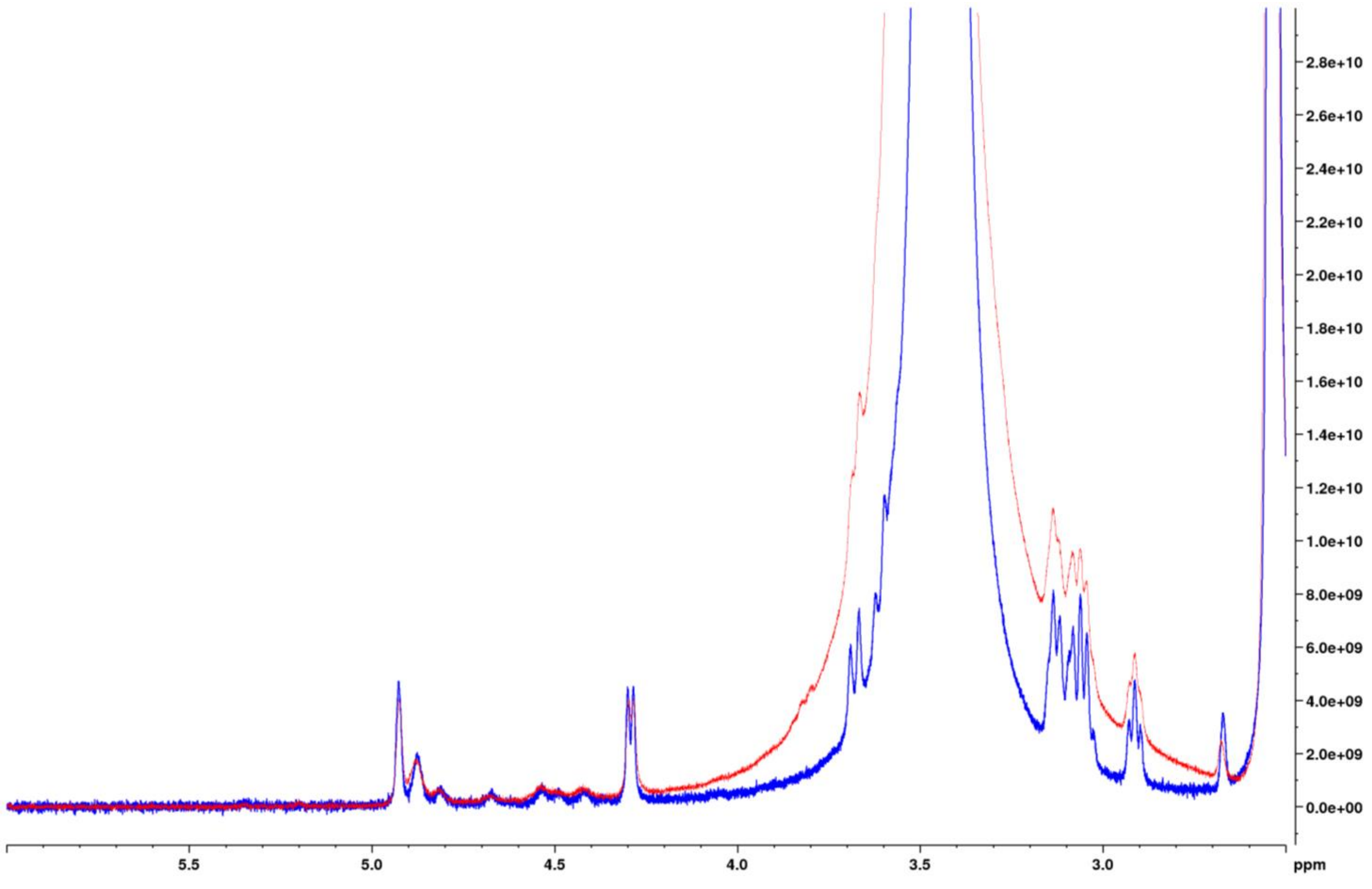

Figure S97. Overlay of ^{1}H-NMR spectra of a fresh (blue) or a dry-down reaction at 50˚C for seven days upon addition of NaOH (red) of glucose in the presence of $MnCl_2$. Samples were dissolved in DMSO-d_6.

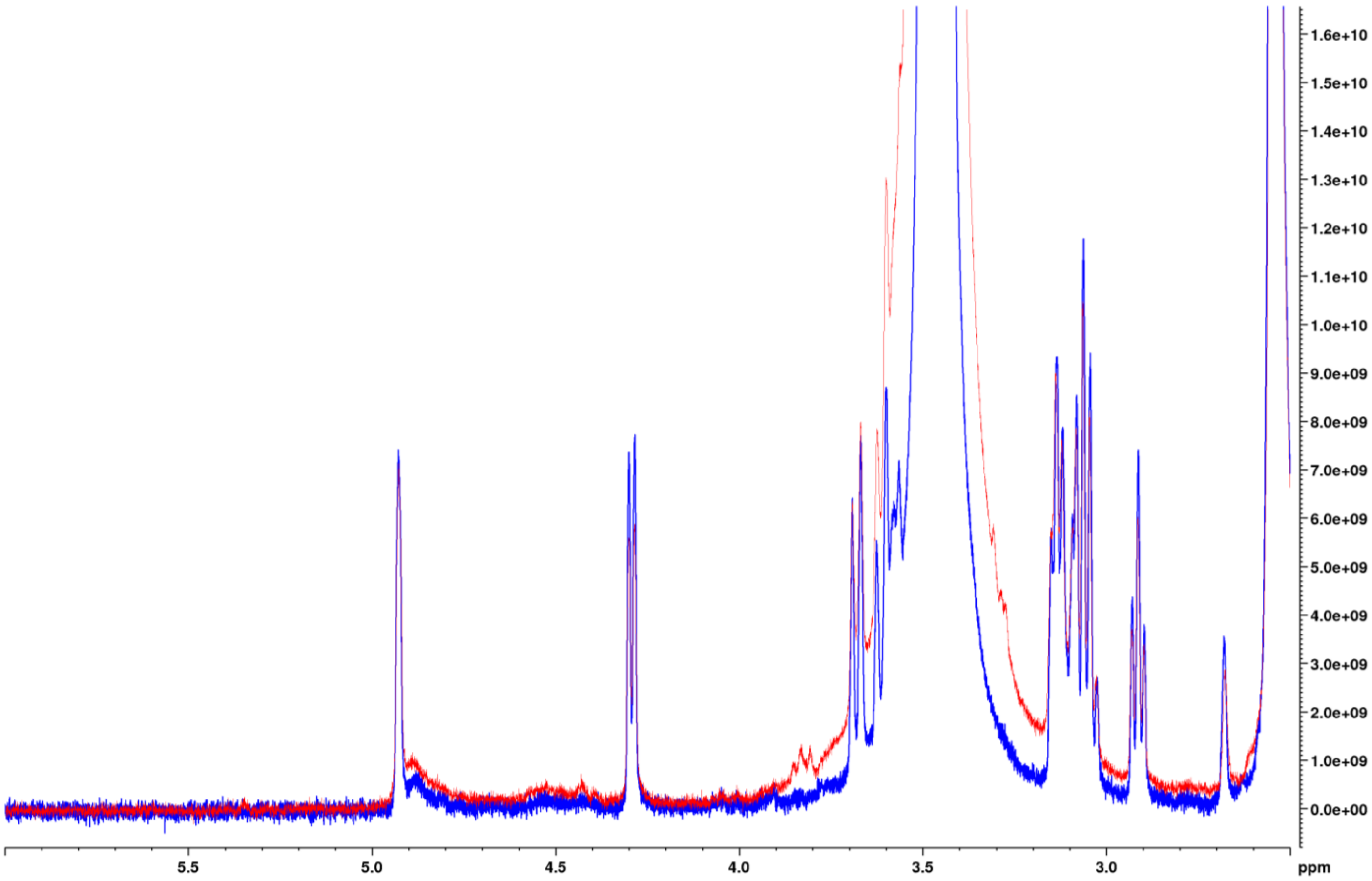

Figure S98. Overlay of ^{1}H-NMR spectra of a fresh (blue) or a dry-down reaction at 50˚C for seven days upon addition of NaOH (red) of glucose in the presence of $FeCl_2$. Samples were dissolved in DMSO-d_6.

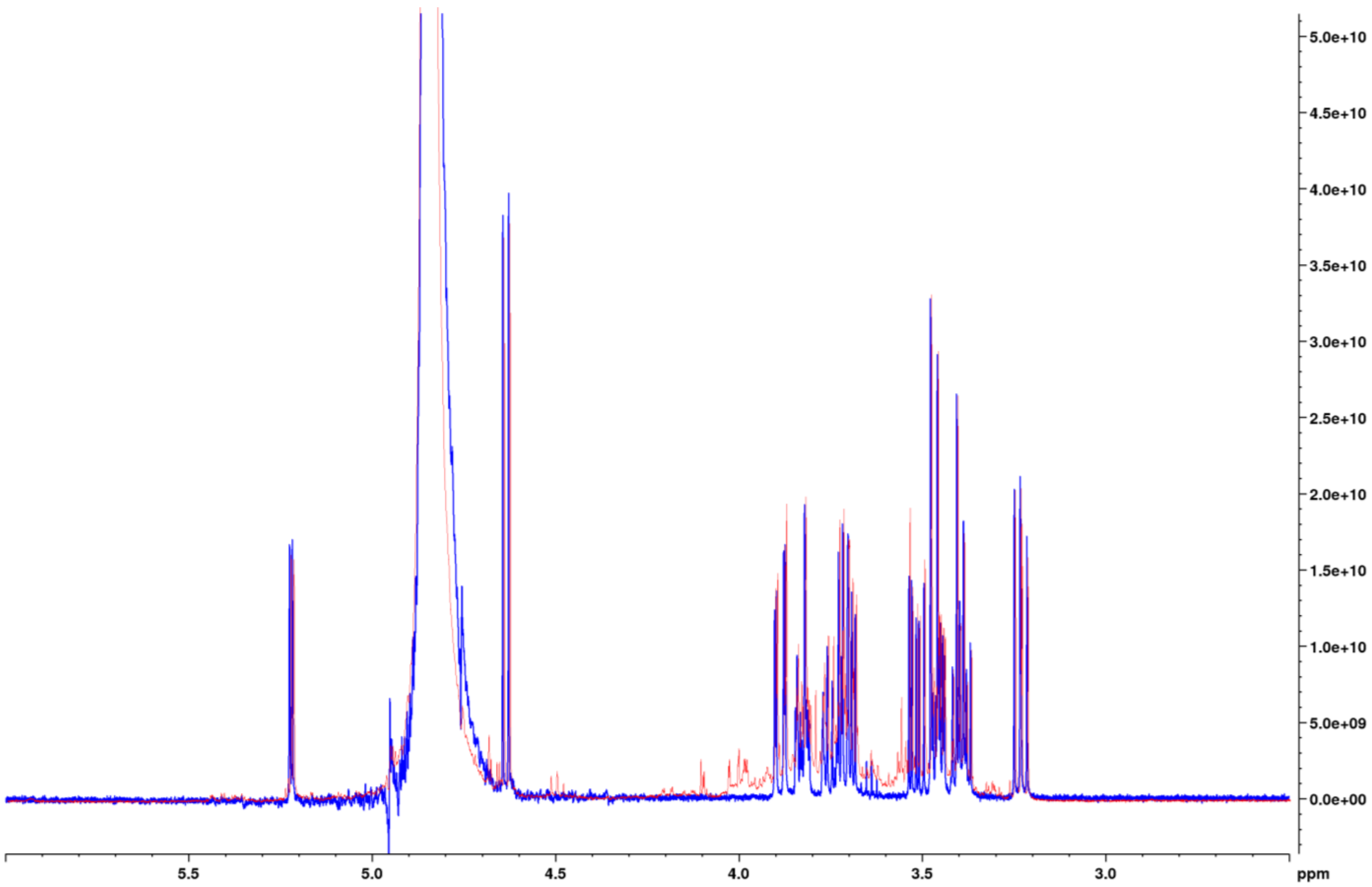

Figure S99. Overlay of ^{1}H-NMR spectra of a fresh (blue) or a dry-down reaction at 50˚C for seven days upon addition of NaOH (red) of glucose in the presence of $AlCl_3$. Samples were dissolved in DMSO-d_6.

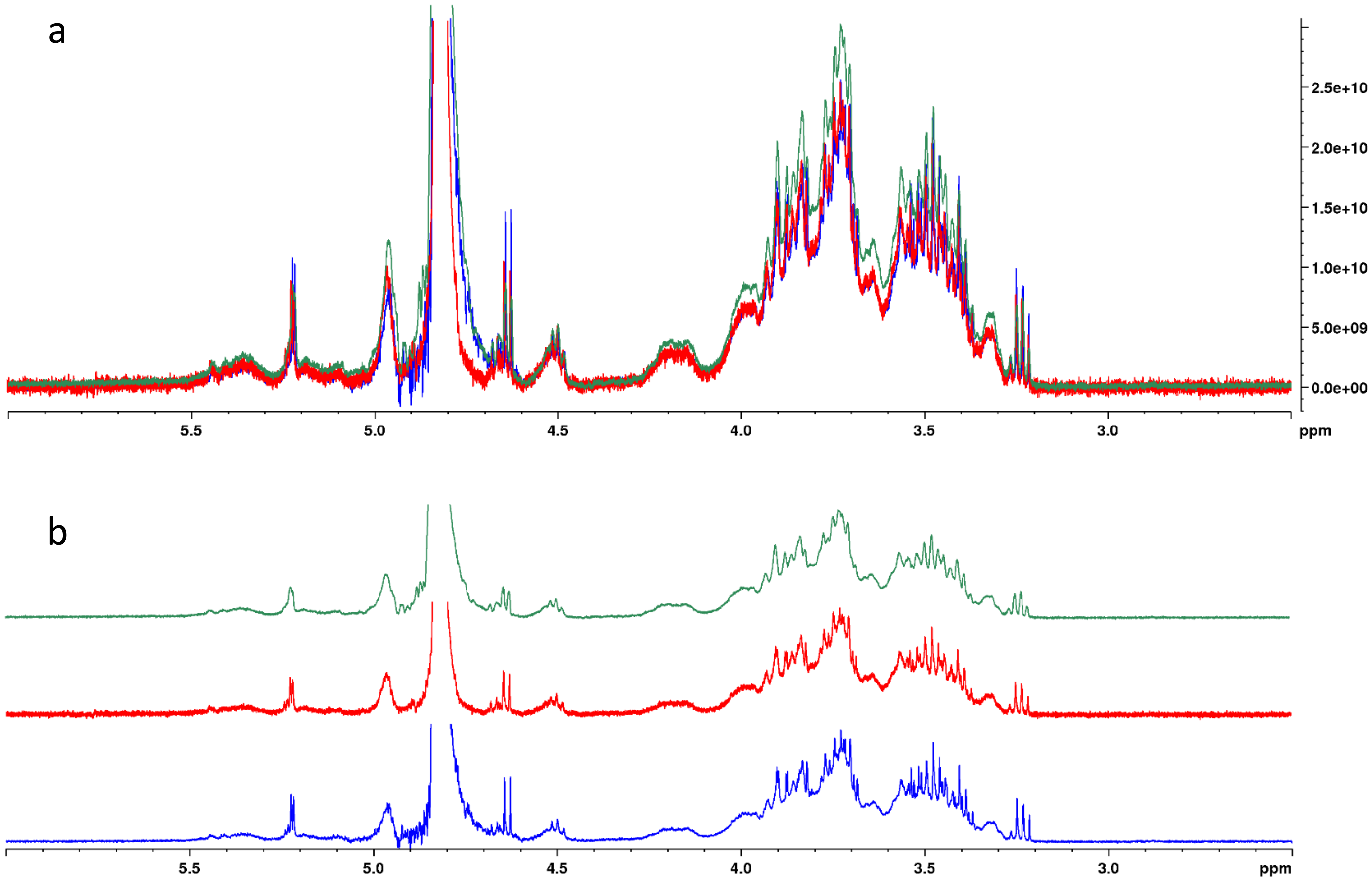

Figure S100. ^{1}H-NMR spectra of three independent dry-down reactions of glucose in the absence of metal ions under acidified conditions. Glucose was dried at 50 ˚C for seven days in three replicates. Overlay (a) and stack spectra (b) of the three replicates are shown.

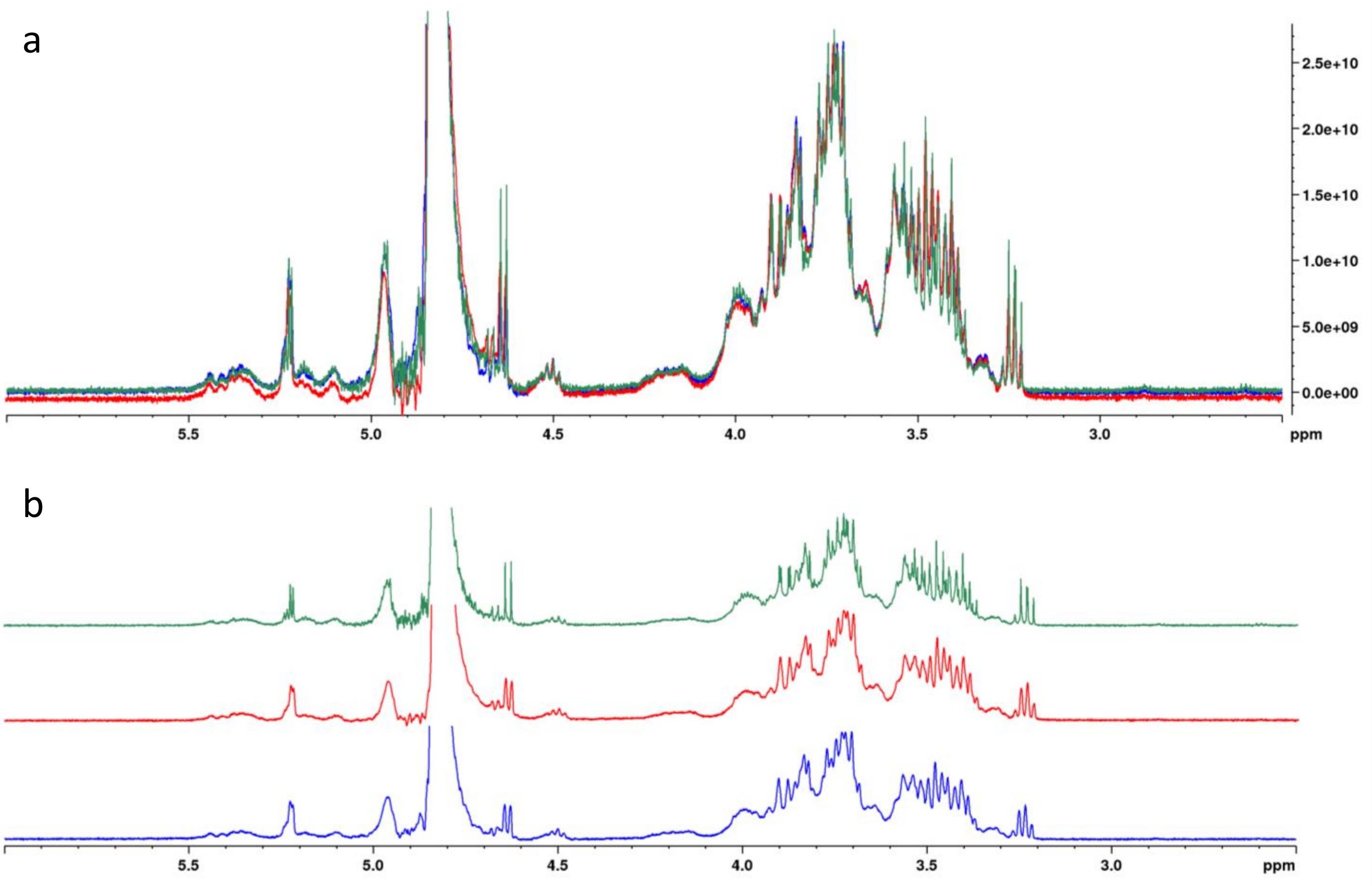

Figure S101. ^{1}H-NMR spectra of three independent dry-down reactions of glucose and LiCl under acidified conditions. Glucose was dried in the presence of LiCl at 50 ˚C for seven days in three replicates. Overlay (a) and stack spectra (b) of the three replicates are shown.

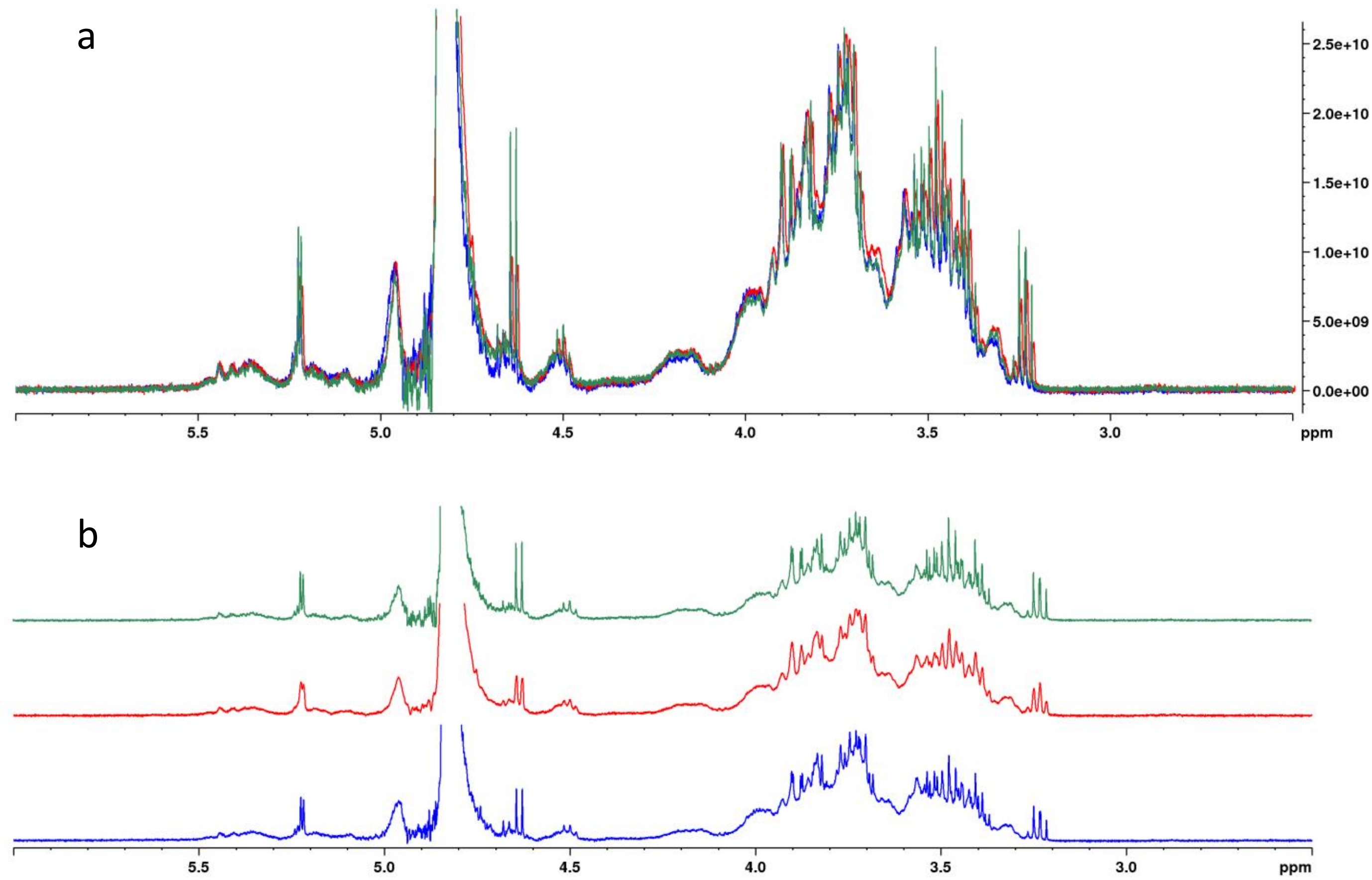

Figure S102. ^{1}H-NMR spectra of three independent dry-down reactions of glucose and $ZnCl_2$ under acidified conditions. Glucose was dried in the presence of $ZnCl_2$ at 50 ˚C for seven days in three replicates. Overlay (a) and stack spectra (b) of the three replicates are shown.

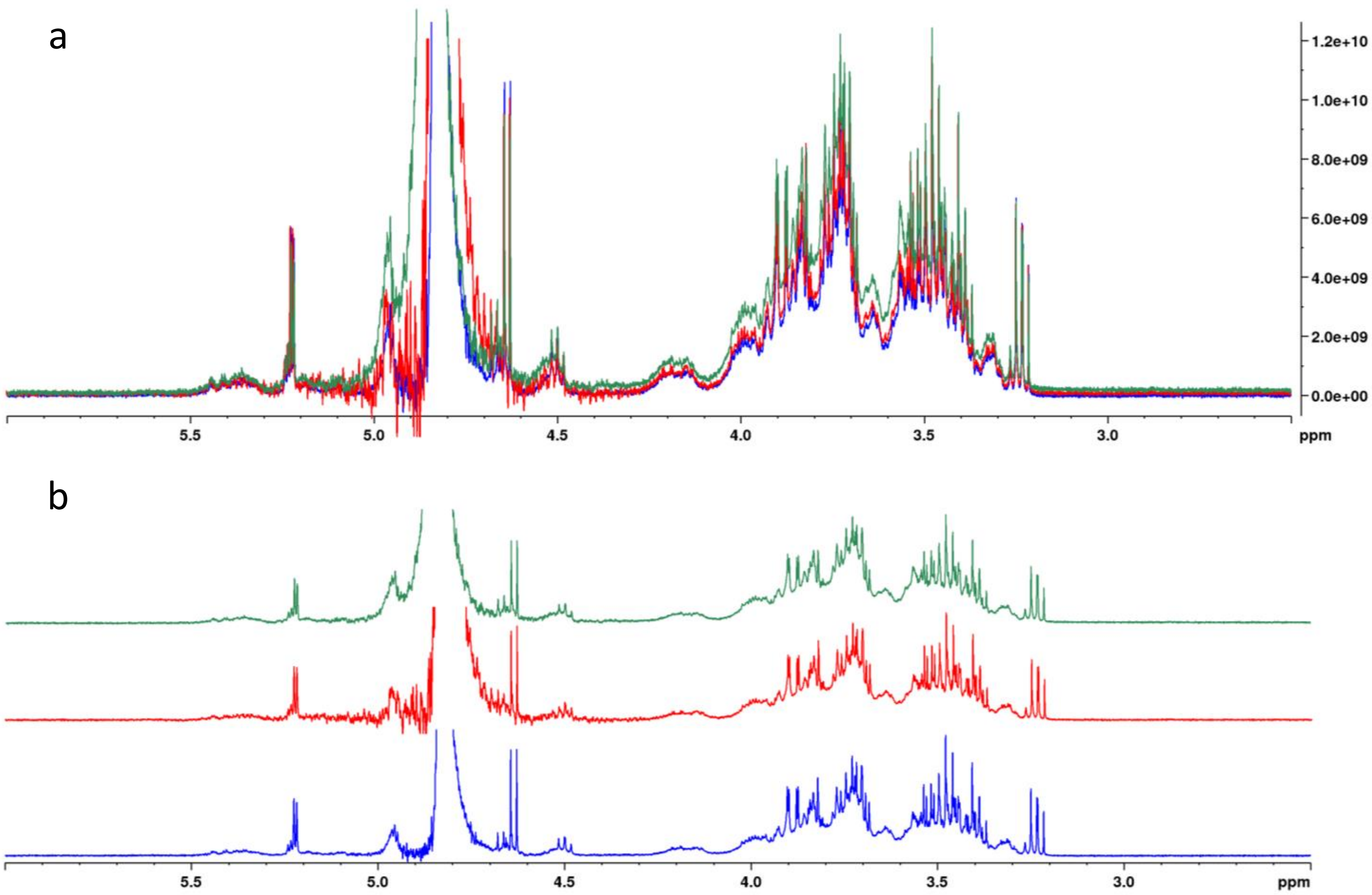

Figure S103. ^{1}H-NMR spectra of three independent dry-down reactions of glucose and NaCl under acidified conditions. Glucose was dried in the presence of NaCl at 50 ˚C for seven days in three replicates. Overlay (a) and stack spectra (b) of the three replicates are shown.

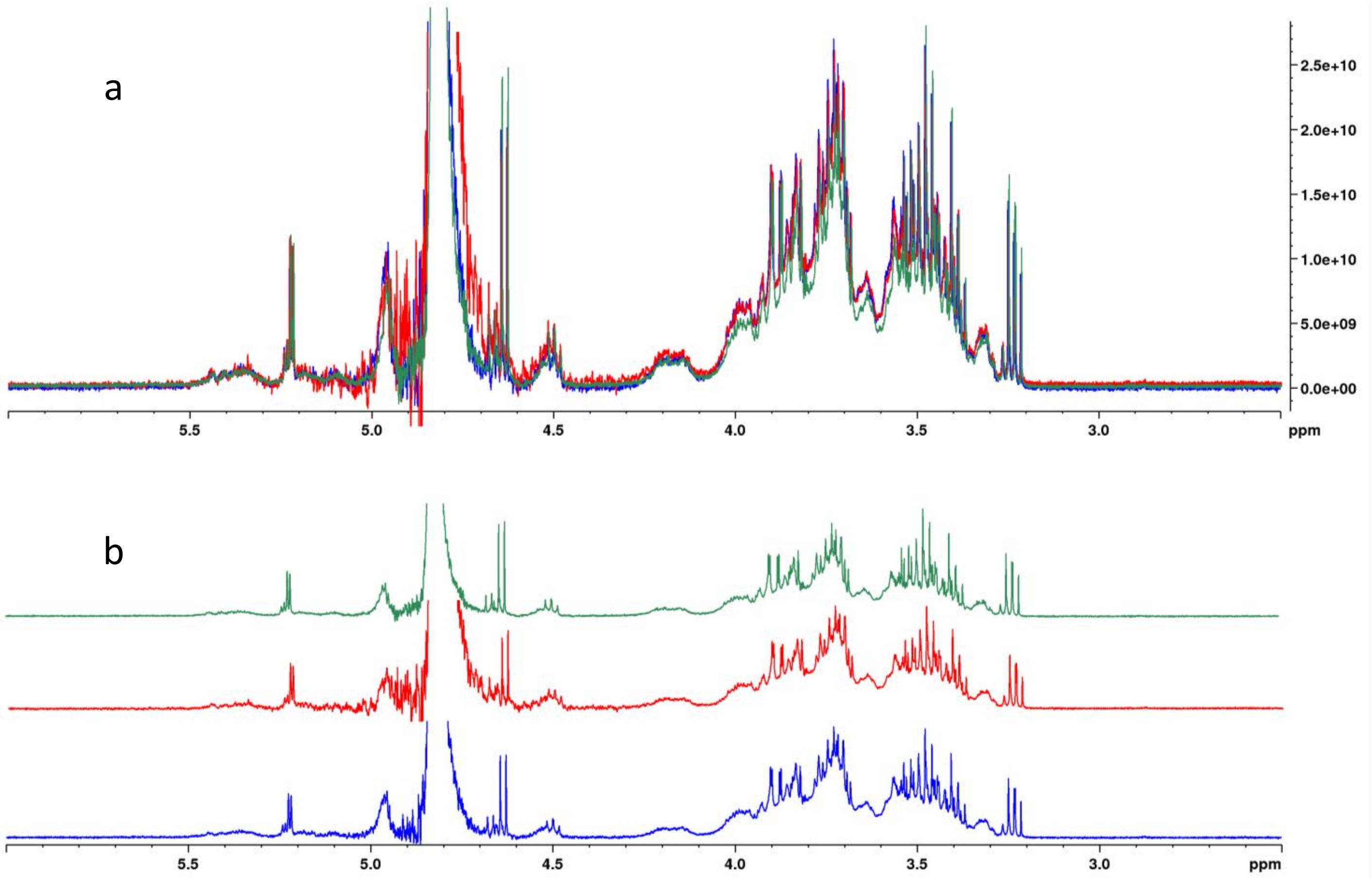

Figure S104. ^{1}H-NMR spectra of three independent dry-down reactions of glucose and KCl under acidified conditions. Glucose was dried in the presence of KCl at 50 ˚C for seven days in three replicates. Overlay (a) and stack spectra (b) of the three replicates are shown.

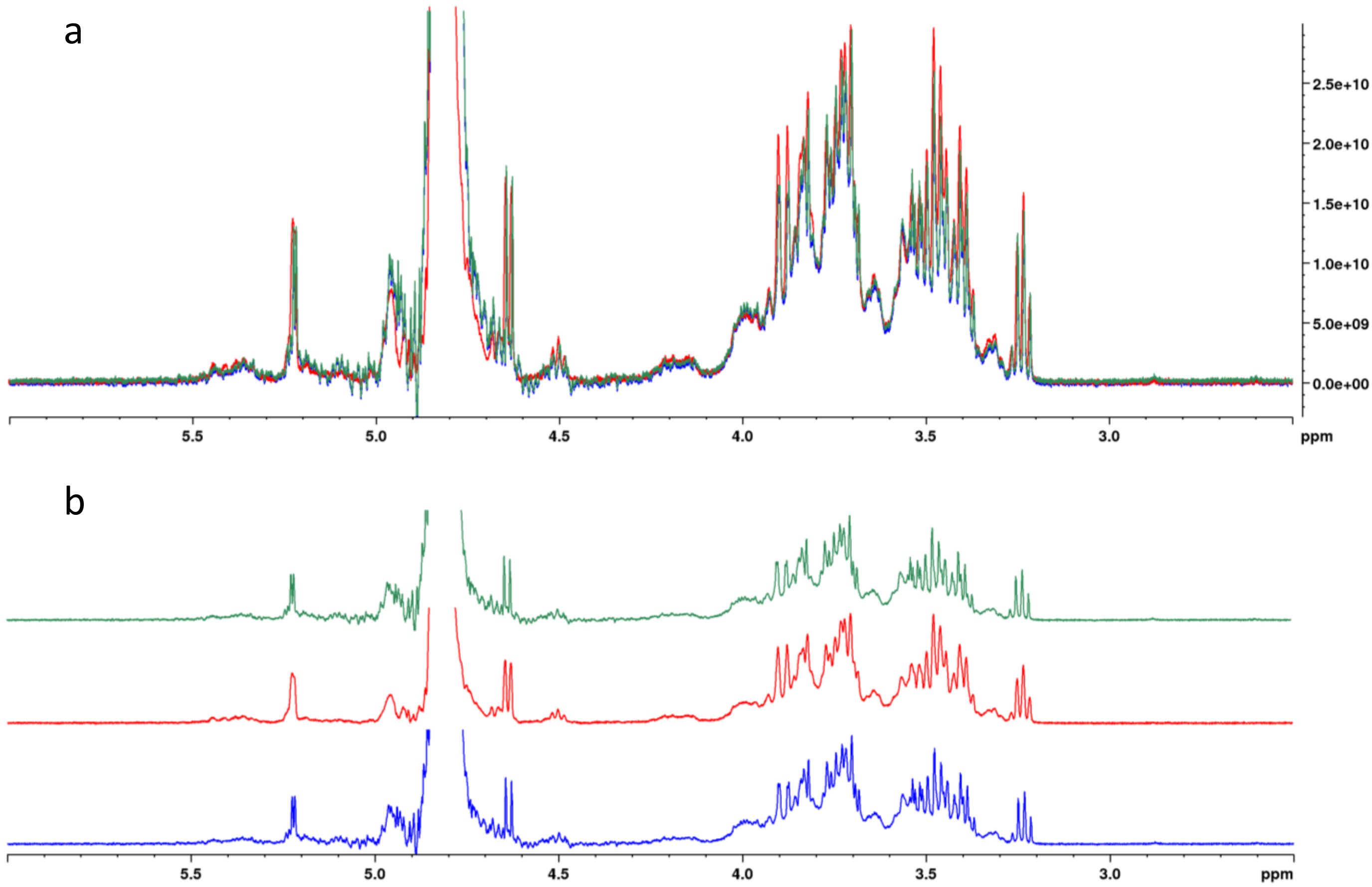

Figure S105. ^{1}H-NMR spectra of three independent dry-down reactions of glucose and $MgCl_2$ under acidified conditions. Glucose was dried in the presence of $MgCl_2$ at 50 ˚C for seven days in three replicates. Overlay (a) and stack spectra (b) of the three replicates are shown.

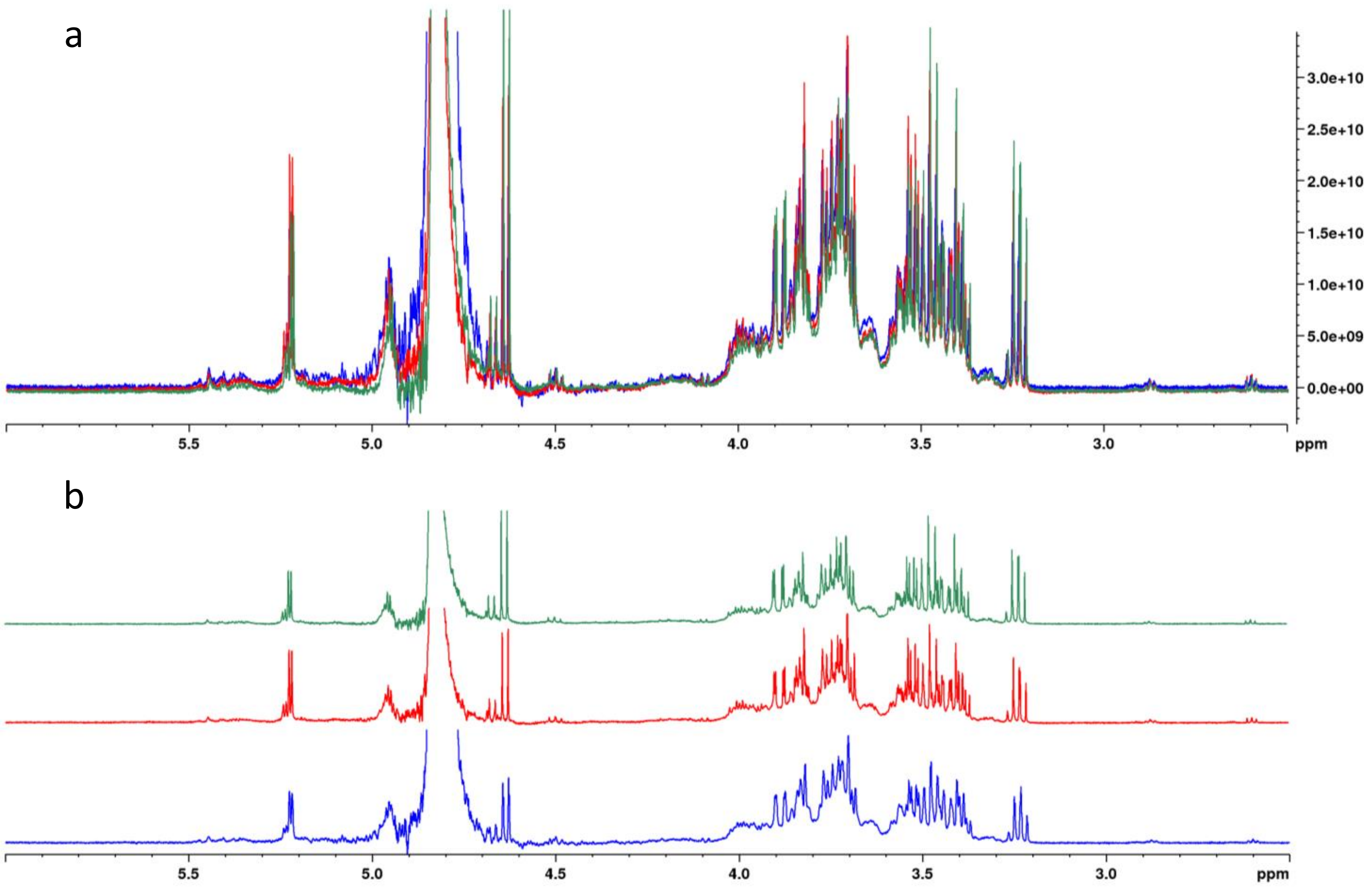

Figure S106. ^{1}H-NMR spectra of three independent dry-down reactions of glucose and $CaCl_2$ under acidified conditions. Glucose was dried in the presence of $CaCl_2$ at 50 ˚C for seven days in three replicates. Overlay (a) and stack spectra (b) of the three replicates are shown.

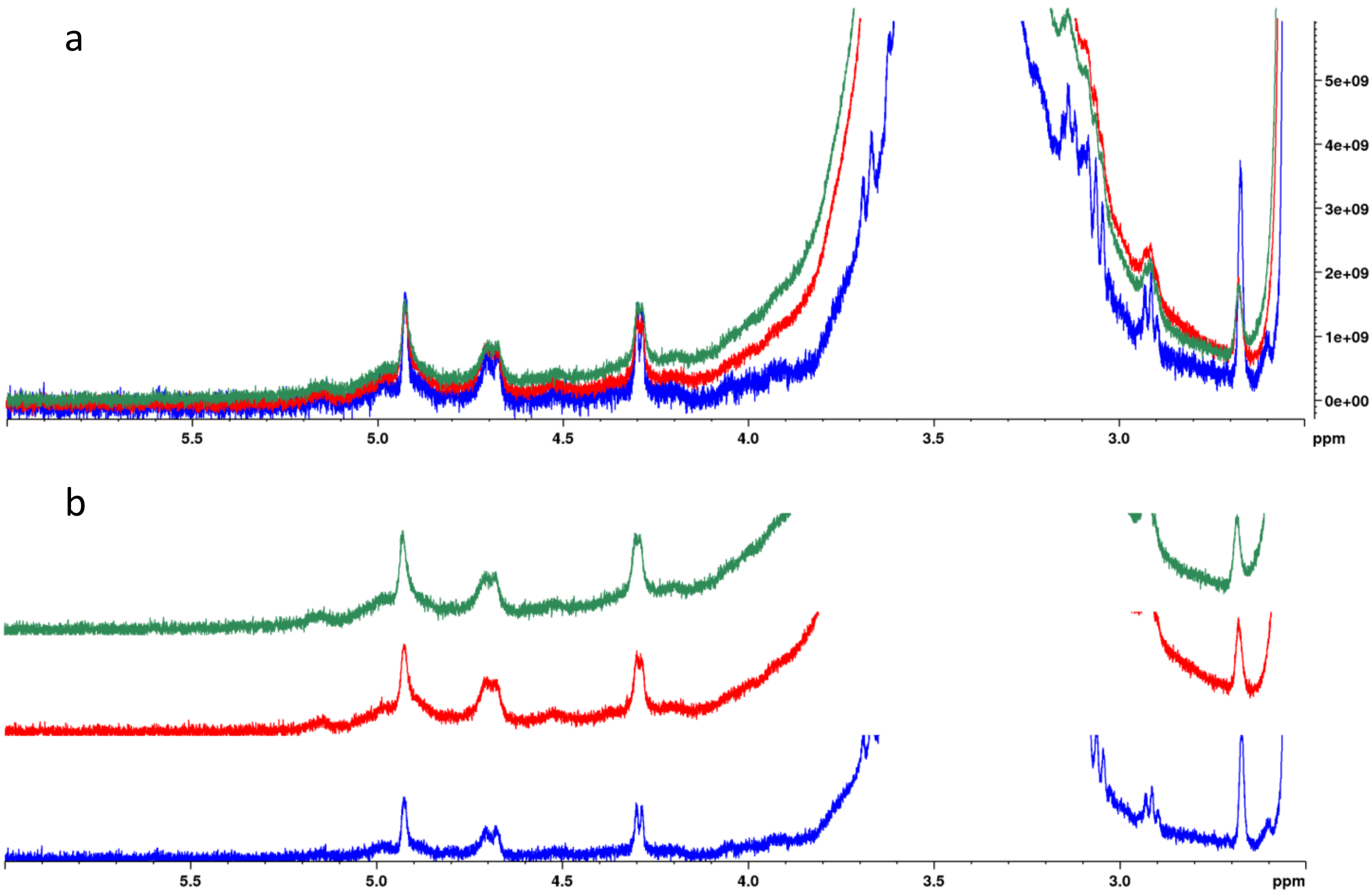

Figure S107. ^{1}H-NMR spectra of three independent dry-down reactions of glucose and $MnCl_2$ under acidified conditions. Glucose was dried in the presence of $MnCl_2$ at 50 ˚C for seven days in three replicates. Overlay (a) and stack spectra (b) of the three replicates are shown.

a

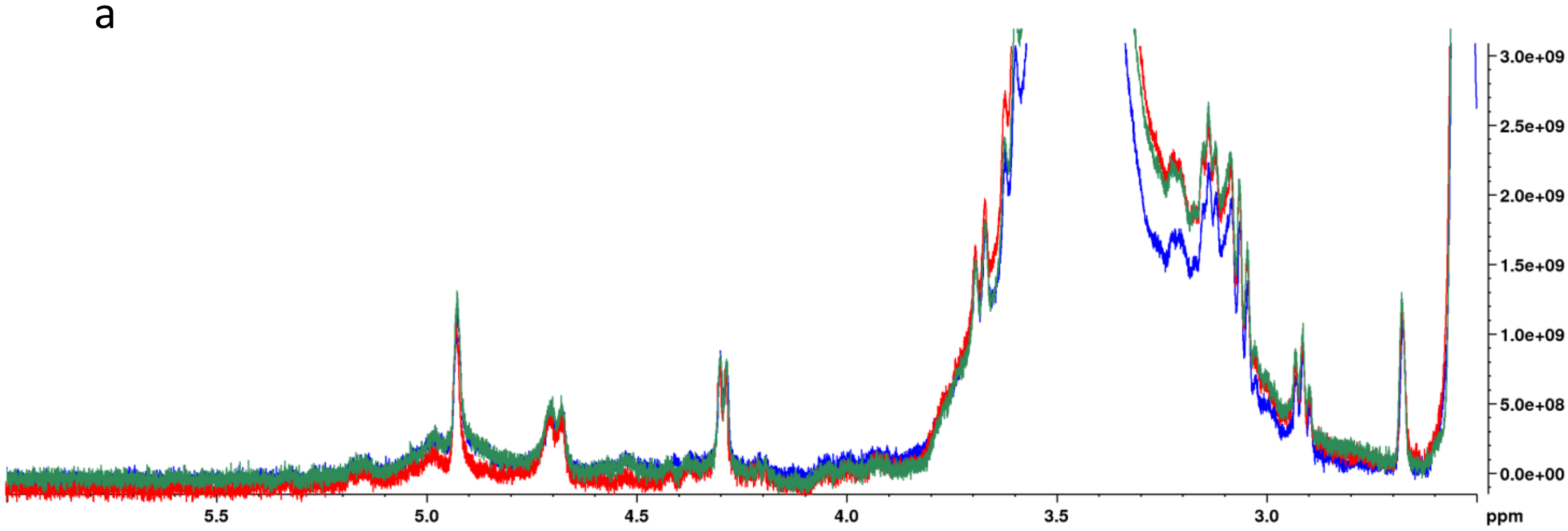

b

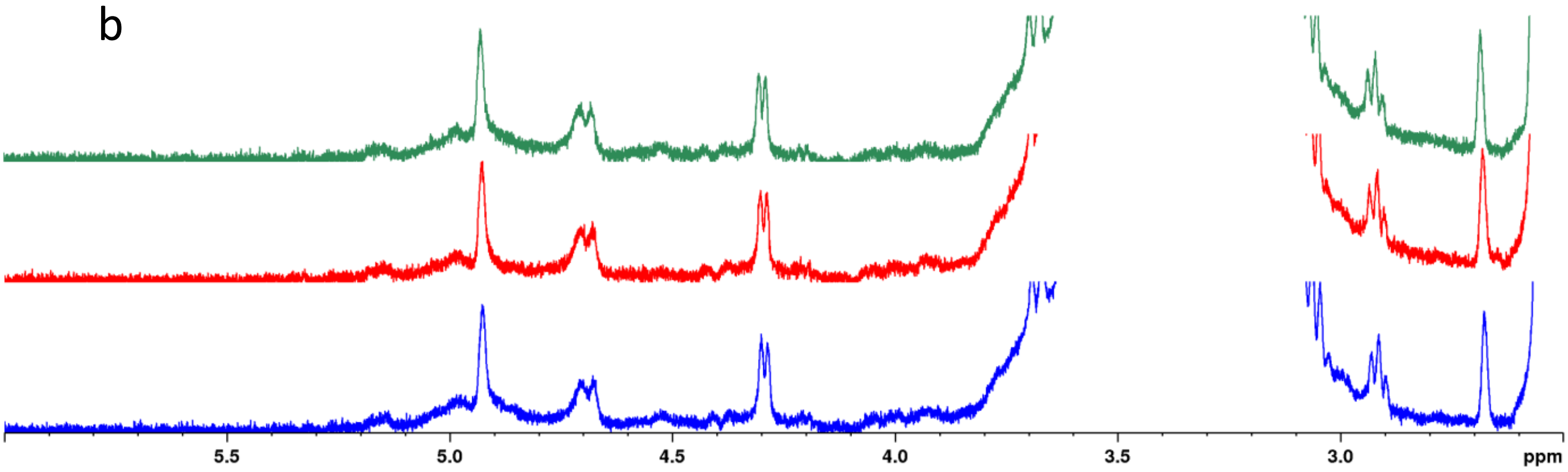

Figure S108. ^{1}H-NMR spectra of three independent dry-down reactions of glucose and $FeCl_2$ under acidified conditions. Glucose was dried in the presence of $FeCl_2$ at 50 ˚C for seven days in three replicates. Overlay (a) and stack spectra (b) of the three replicates are shown.

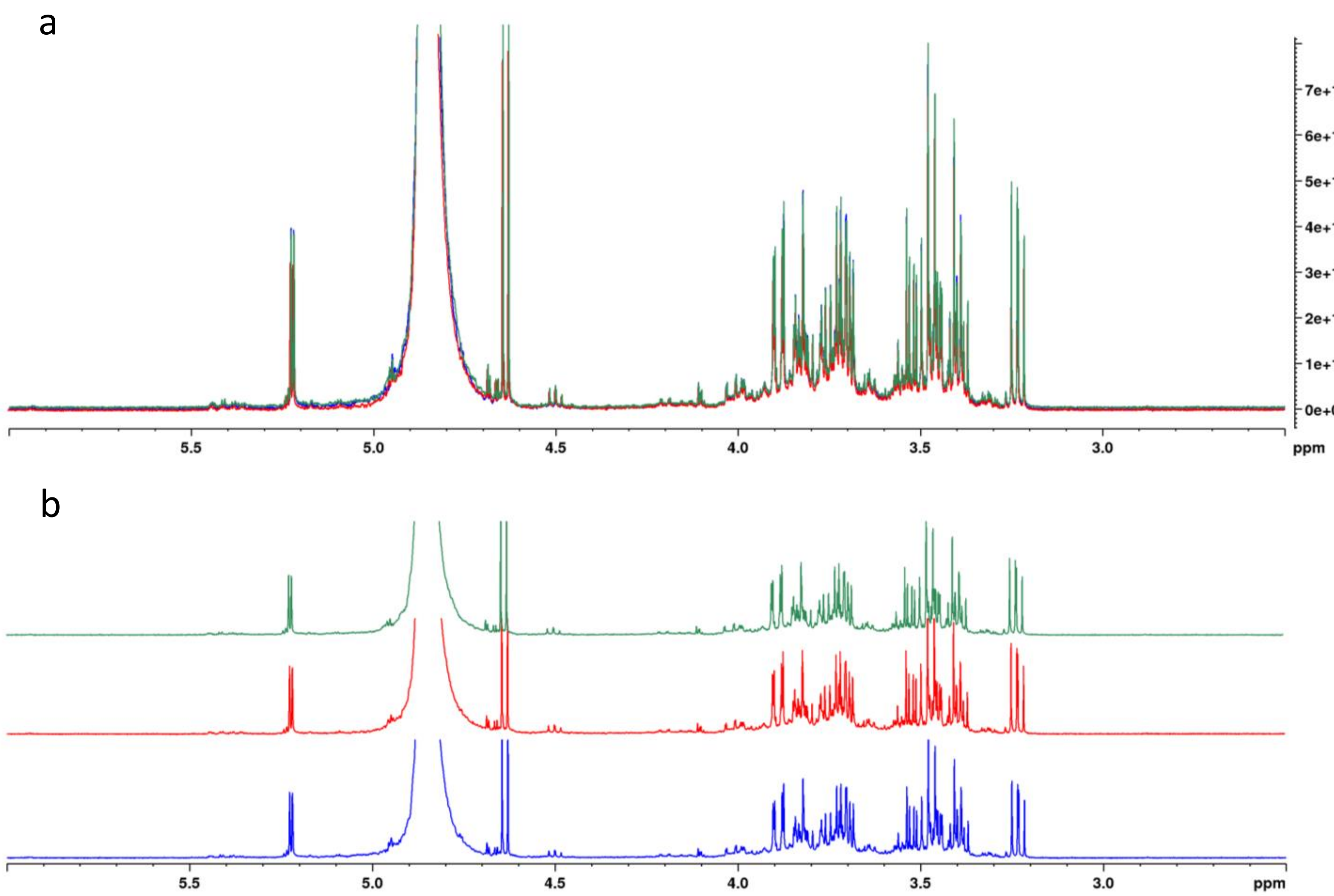

Figure S109. ^{1}H-NMR spectra of three independent dry-down reactions of glucose and $AlCl_3$ under acidified conditions. Glucose was dried in the presence of $AlCl_3$ at 50 ˚C for seven days in three replicates. Overlay (a) and stack spectra (b) of the three replicates are shown.

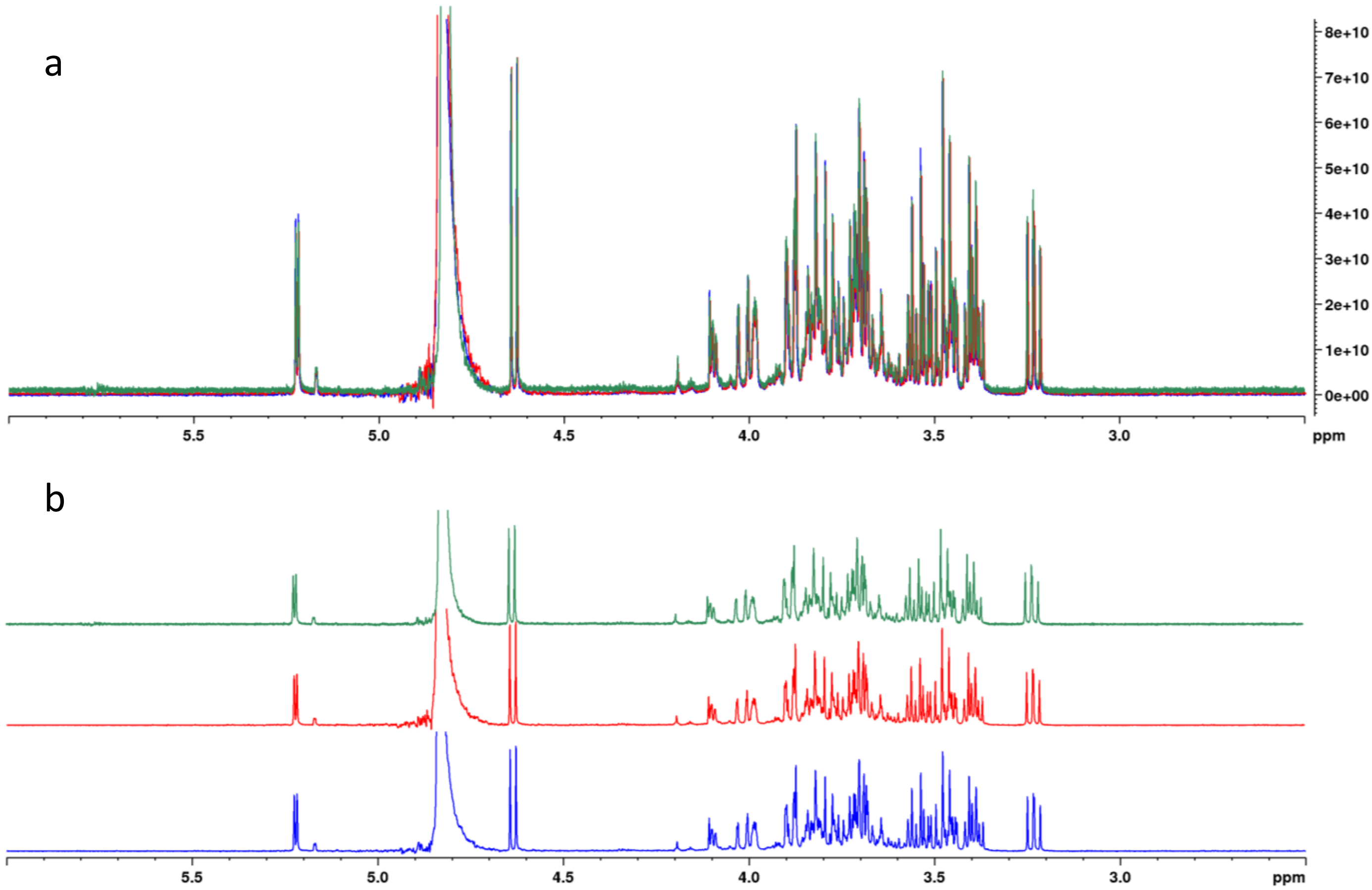

Figure S110. ^{1}H-NMR spectra of three independent dry-down reactions of glucose in the absence of metal ions upon addition of NaOH. Glucose was dried at 50 ˚C for seven days in three replicates. Overlay (a) and stack spectra (b) of the three replicates are shown.

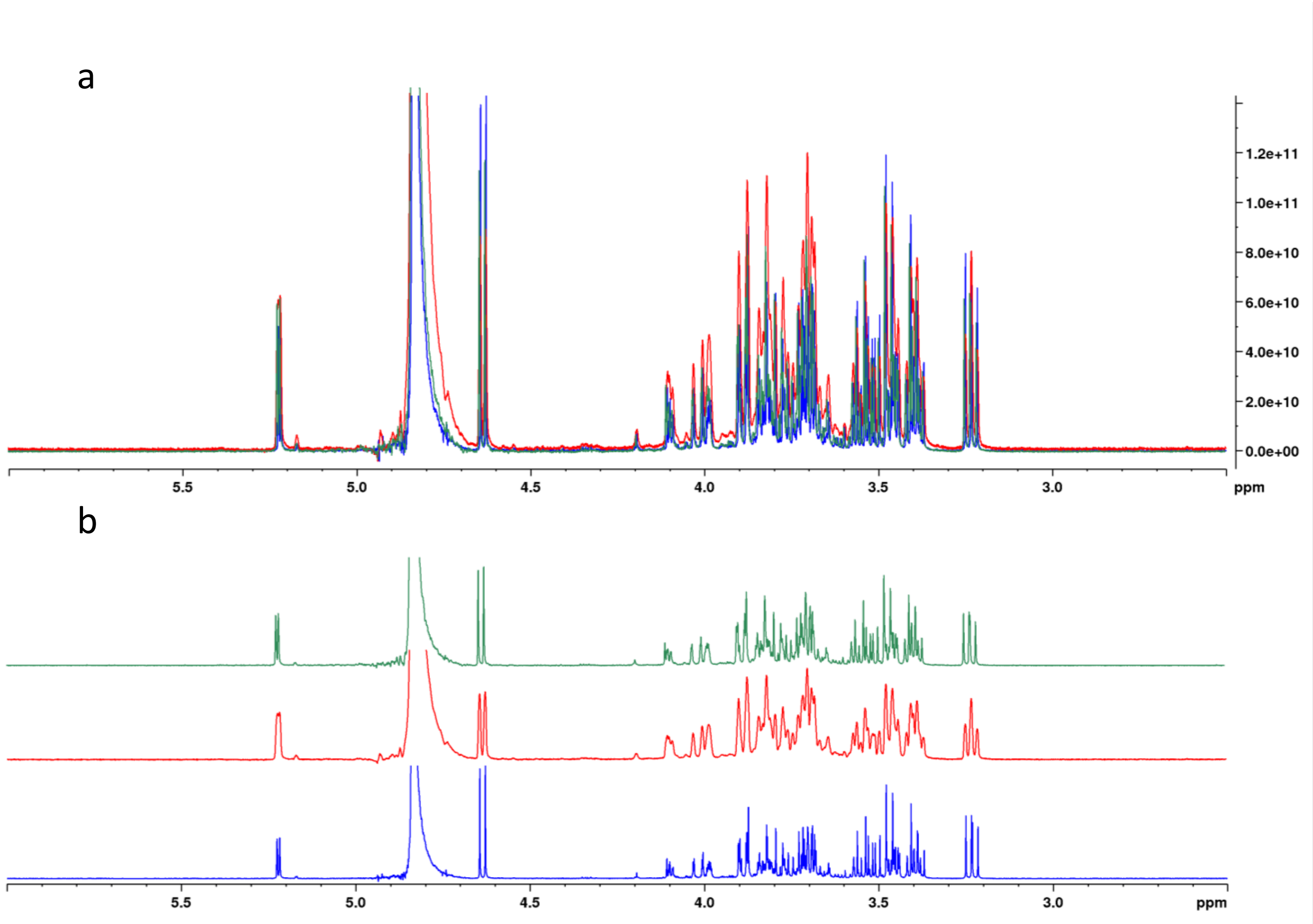

Figure S111. ^{1}H-NMR spectra of three independent dry-down reactions of glucose and LiCl upon addition of NaOH. Glucose was dried in the presence of LiCl at 50 ˚C for seven days in three replicates. Overlay (a) and stack spectra (b) of the three replicates are shown.

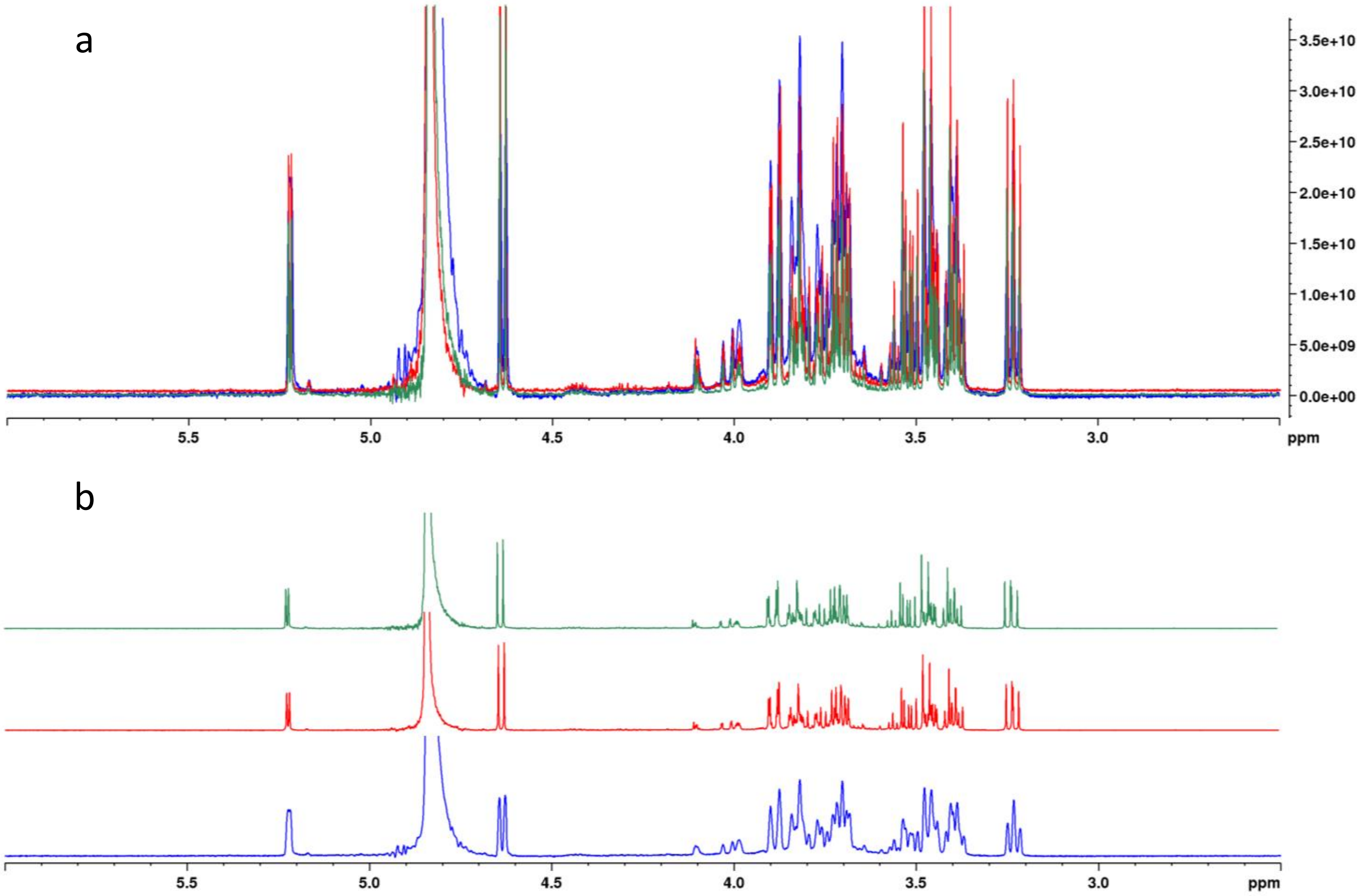

Figure S112. ^{1}H-NMR spectra of three independent dry-down reactions of glucose and $ZnCl_2$ upon addition of NaOH. Glucose was dried in the presence of $ZnCl_2$ at 50 ˚C for seven days in three replicates. Overlay (a) and stack spectra (b) of the three replicates are shown.

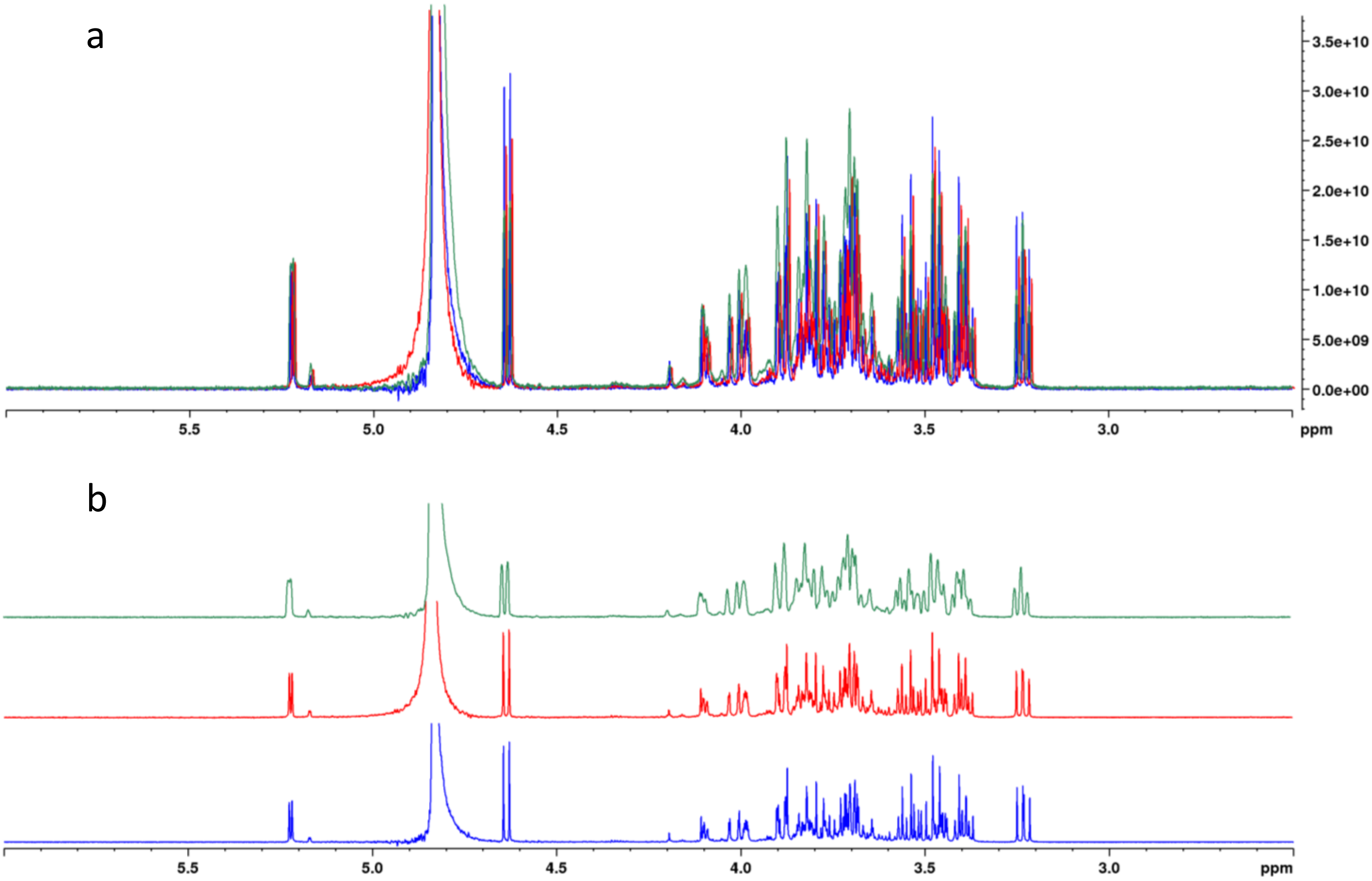

Figure S113. ^{1}H-NMR spectra of three independent dry-down reactions of glucose and NaCl upon addition of NaOH. Glucose was dried in the presence of NaCl at 50 ˚C for seven days in three replicates. Overlay (a) and stack spectra (b) of the three replicates are shown.

a

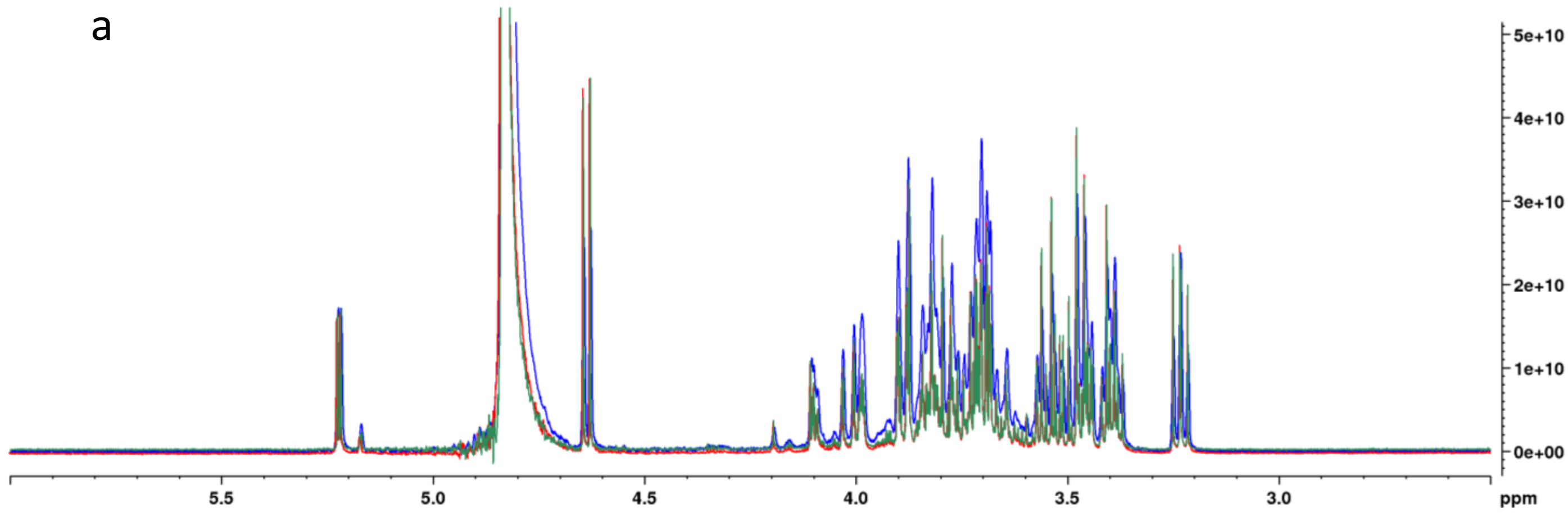

b

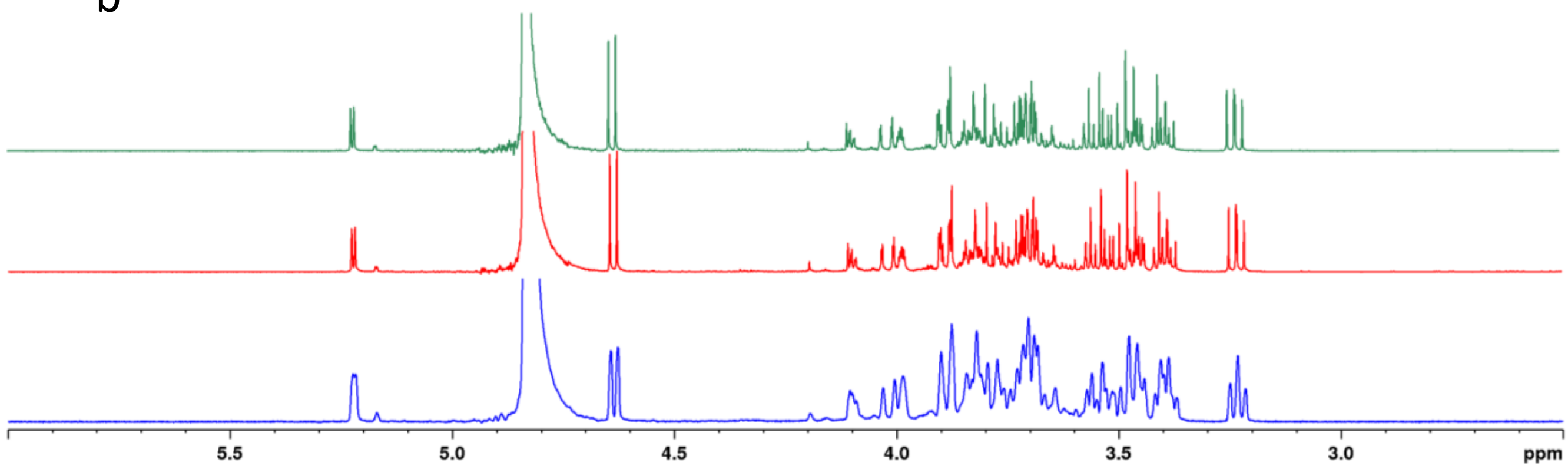

Figure S114. ^{1}H-NMR spectra of three independent dry-down reactions of glucose and KCl upon addition of NaOH. Glucose was dried in the presence of KCl at 50 ˚C for seven days in three replicates. Overlay (a) and stack spectra (b) of the three replicates are shown.

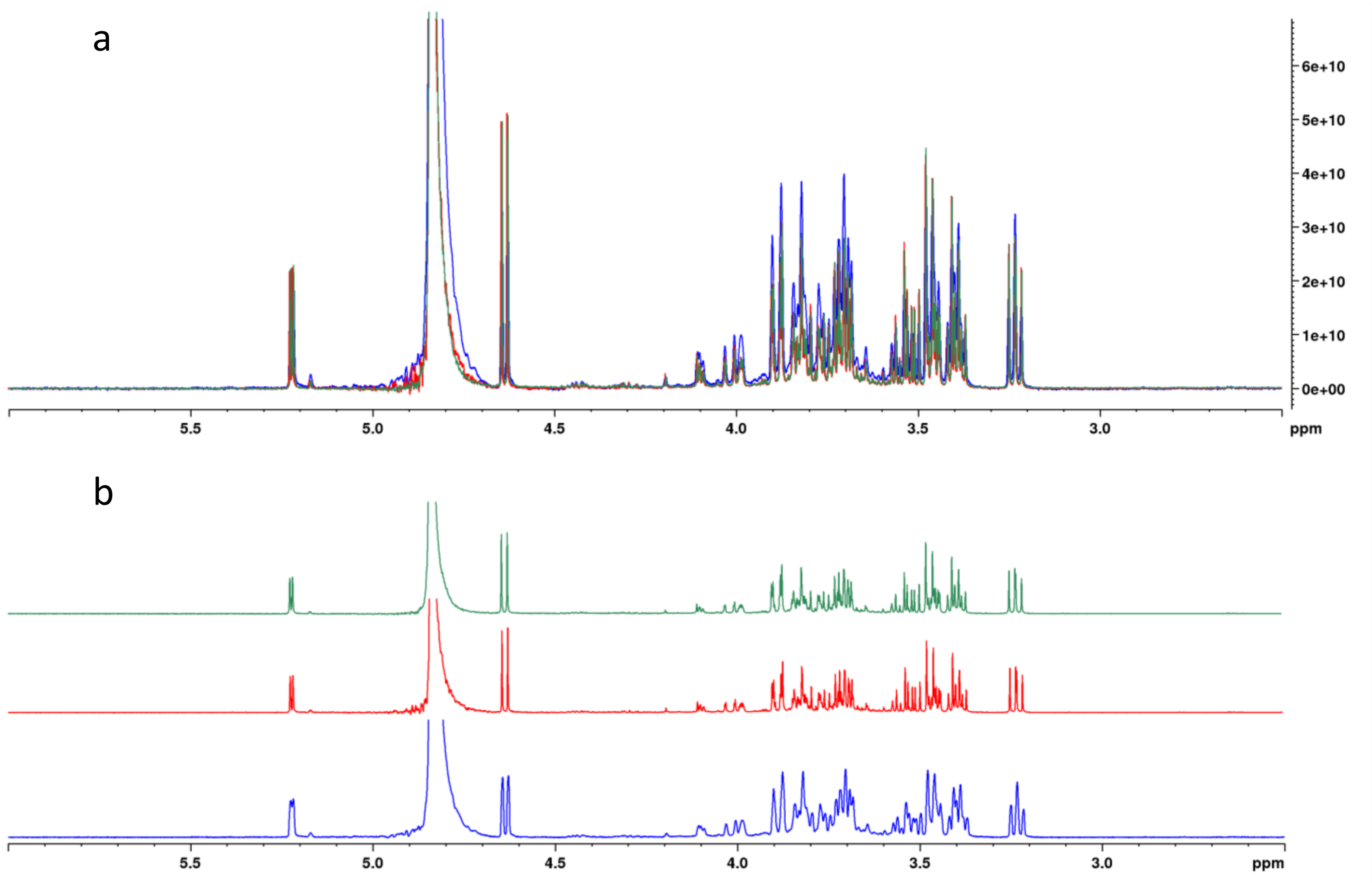

Figure S115. ^{1}H-NMR spectra of three independent dry-down reactions of glucose and $MgCl_2$ upon addition of NaOH. Glucose was dried in the presence of $MgCl_2$ at 50 ˚C for seven days in three replicates. Overlay (a) and stack spectra (b) of the three replicates are shown.

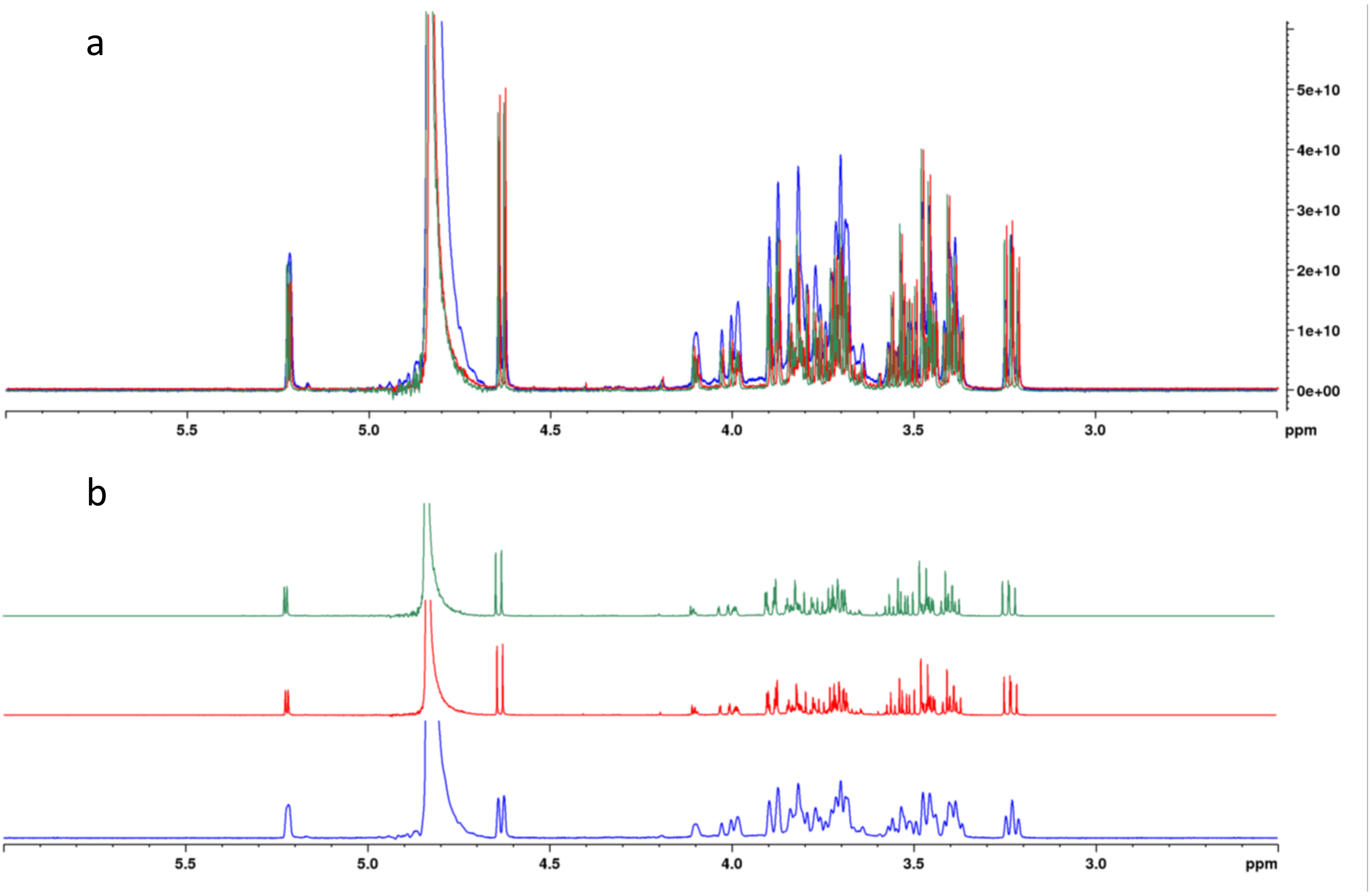

Figure S116. ^{1}H-NMR spectra of three independent dry-down reactions of glucose and $CaCl_2$ upon addition of NaOH. Glucose was dried in the presence of $CaCl_2$ at 50 ˚C for seven days in three replicates. Overlay (a) and stack spectra (b) of the three replicates are shown.

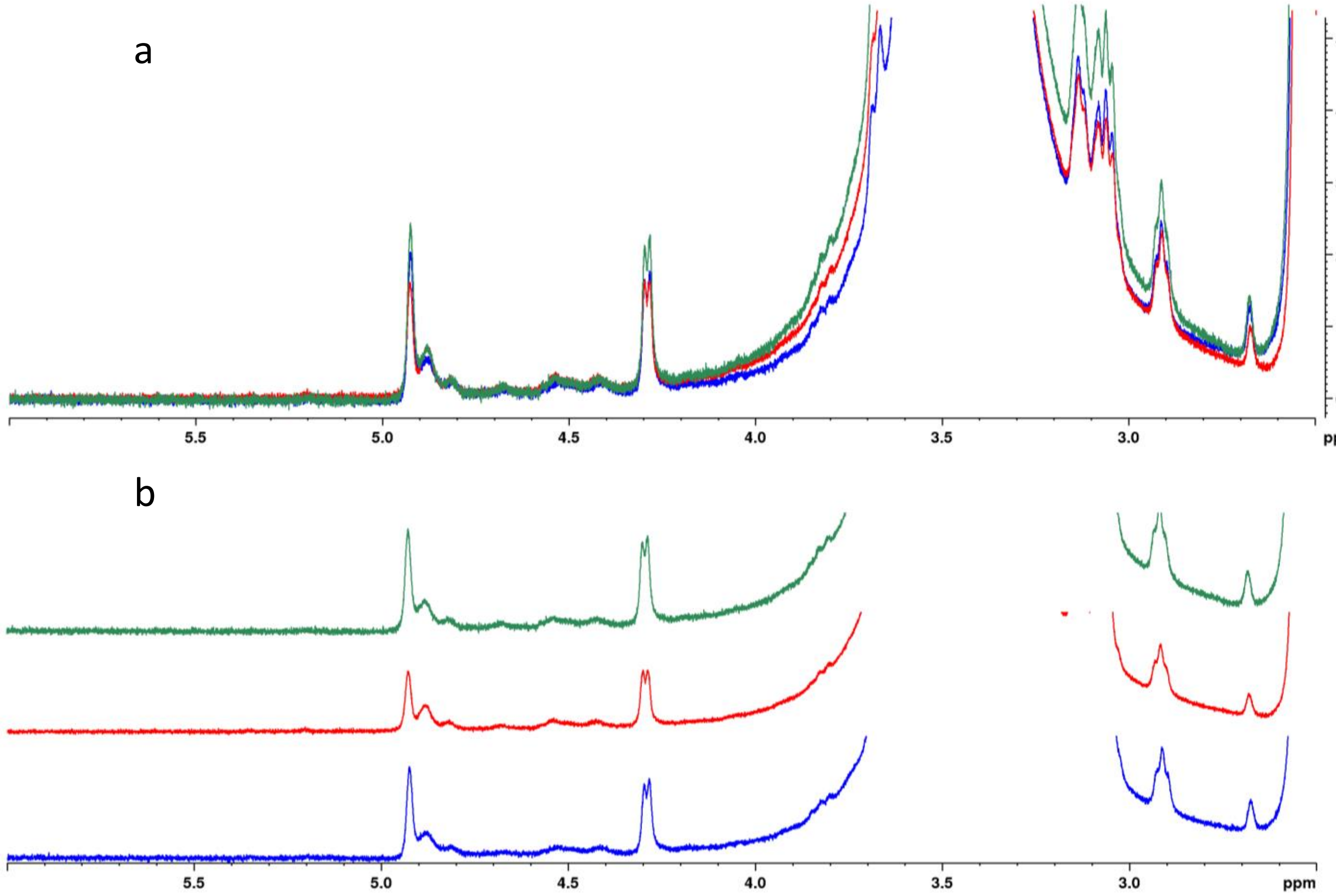

Figure S117. ^{1}H-NMR spectra of three independent dry-down reactions of glucose and $MnCl_2$ upon addition of NaOH. Glucose was dried in the presence of $MnCl_2$ at 50 ˚C for seven days in three replicates. Overlay (a) and stack spectra (b) of the three replicates are shown.

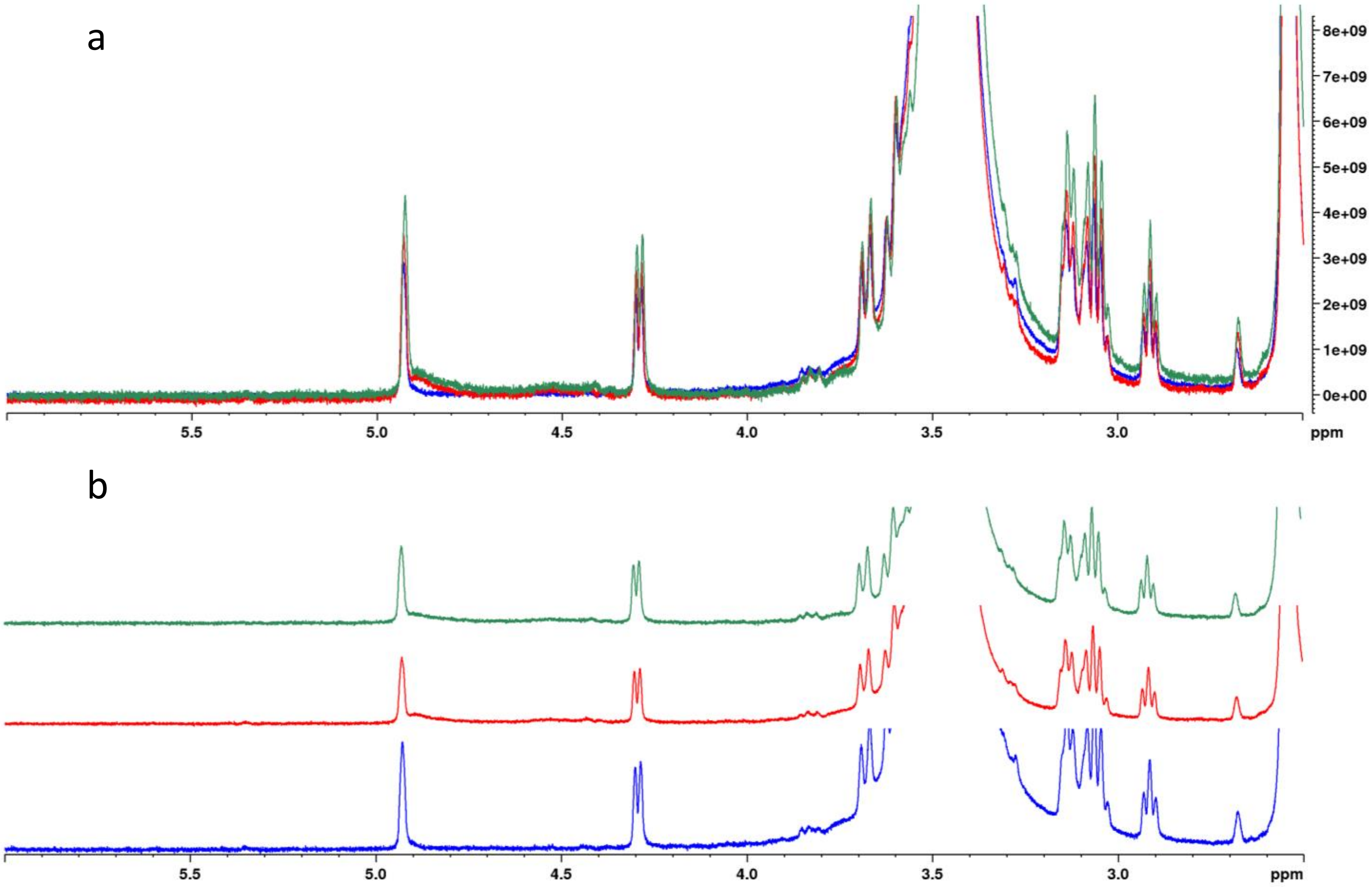

Figure S118. ^{1}H-NMR spectra of three independent dry-down reactions of glucose and $FeCl_2$ upon addition of NaOH. Glucose was dried in the presence of $FeCl_2$ at 50 ˚C for seven days in three replicates. Overlay (a) and stack spectra (b) of the three replicates are shown.

a

b

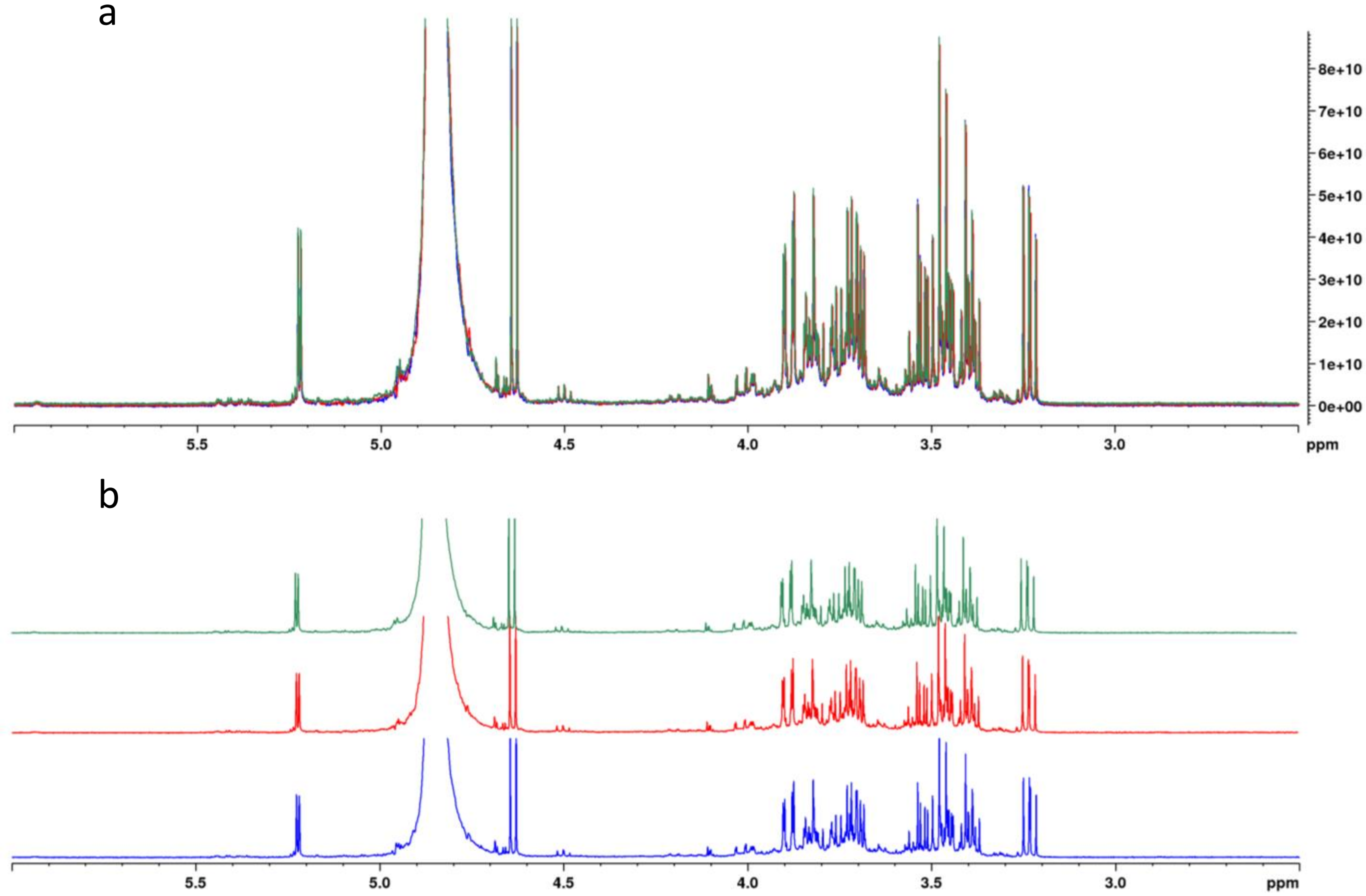

Figure S119. ^{1}H-NMR spectra of three independent dry-down reactions of glucose and $AlCl_3$ upon addition of NaOH. Glucose was dried in the presence of $AlCl_3$ at 50 ˚C for seven days in three replicates. Overlay (a) and stack spectra (b) of the three replicates are shown.

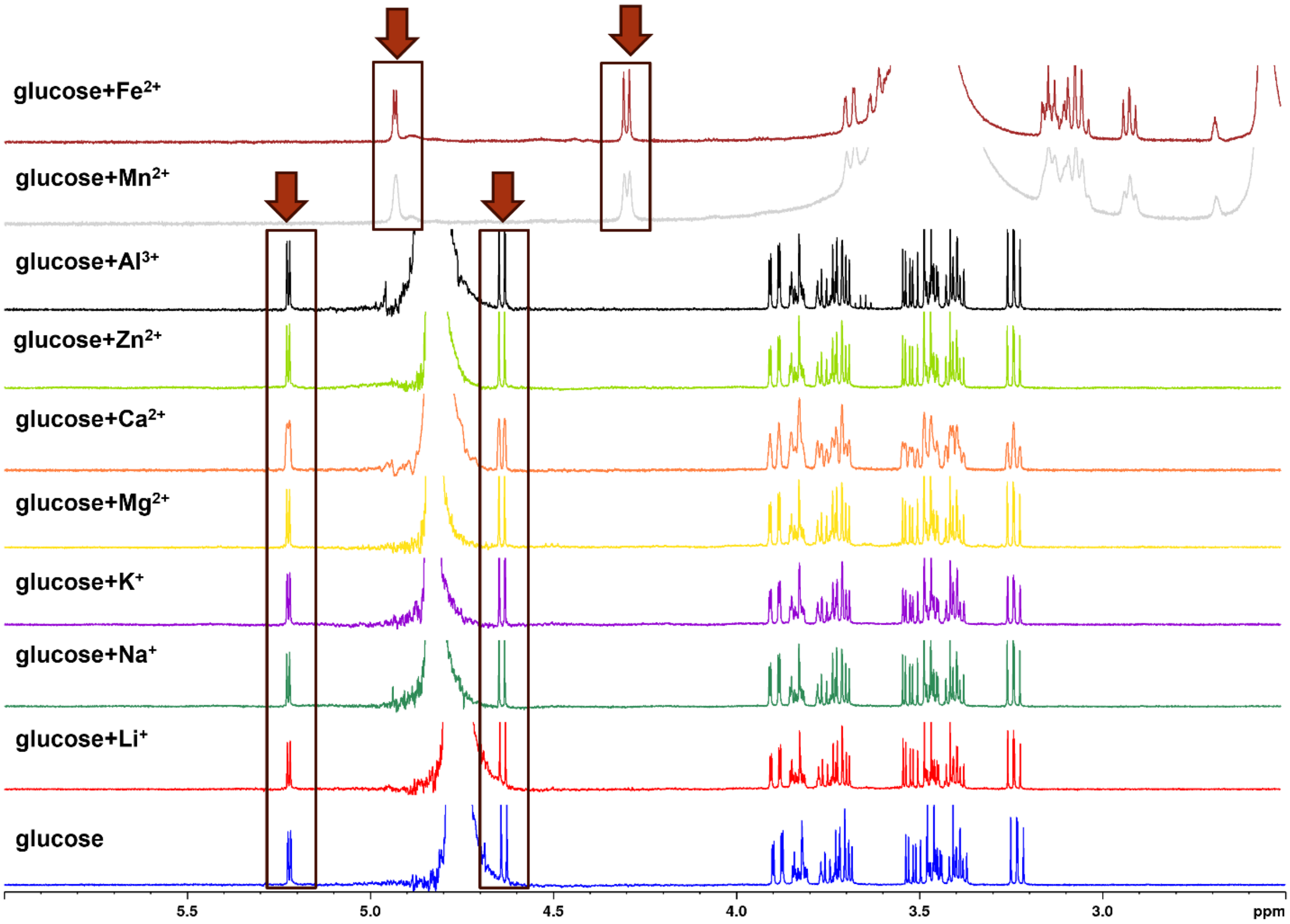

Figure S120. ^{1}H-NMR spectra of fresh glucose in the absence or presence of various metal ions under acidified conditions. The samples were dissolved in DMSO-d_6 (a) or D_2O buffer (pH=2.66) (b).

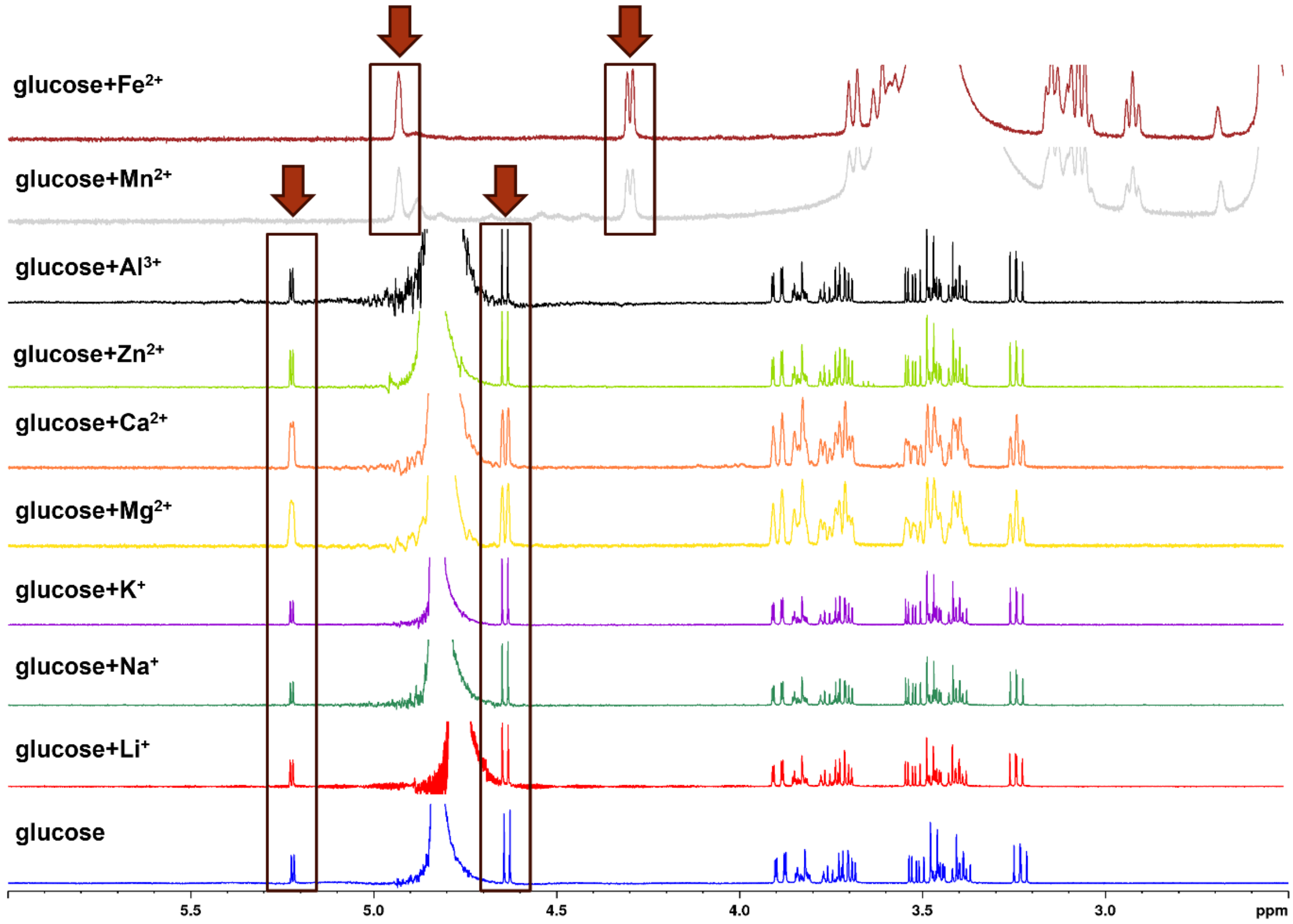

Figure S121. ^{1}H-NMR spectra of fresh glucose in the absence or presence of various metal ions upon addition of NaOH. The samples were dissolved in DMSO-d_6 (a) or D_2O buffer (pH=2.66) (b).

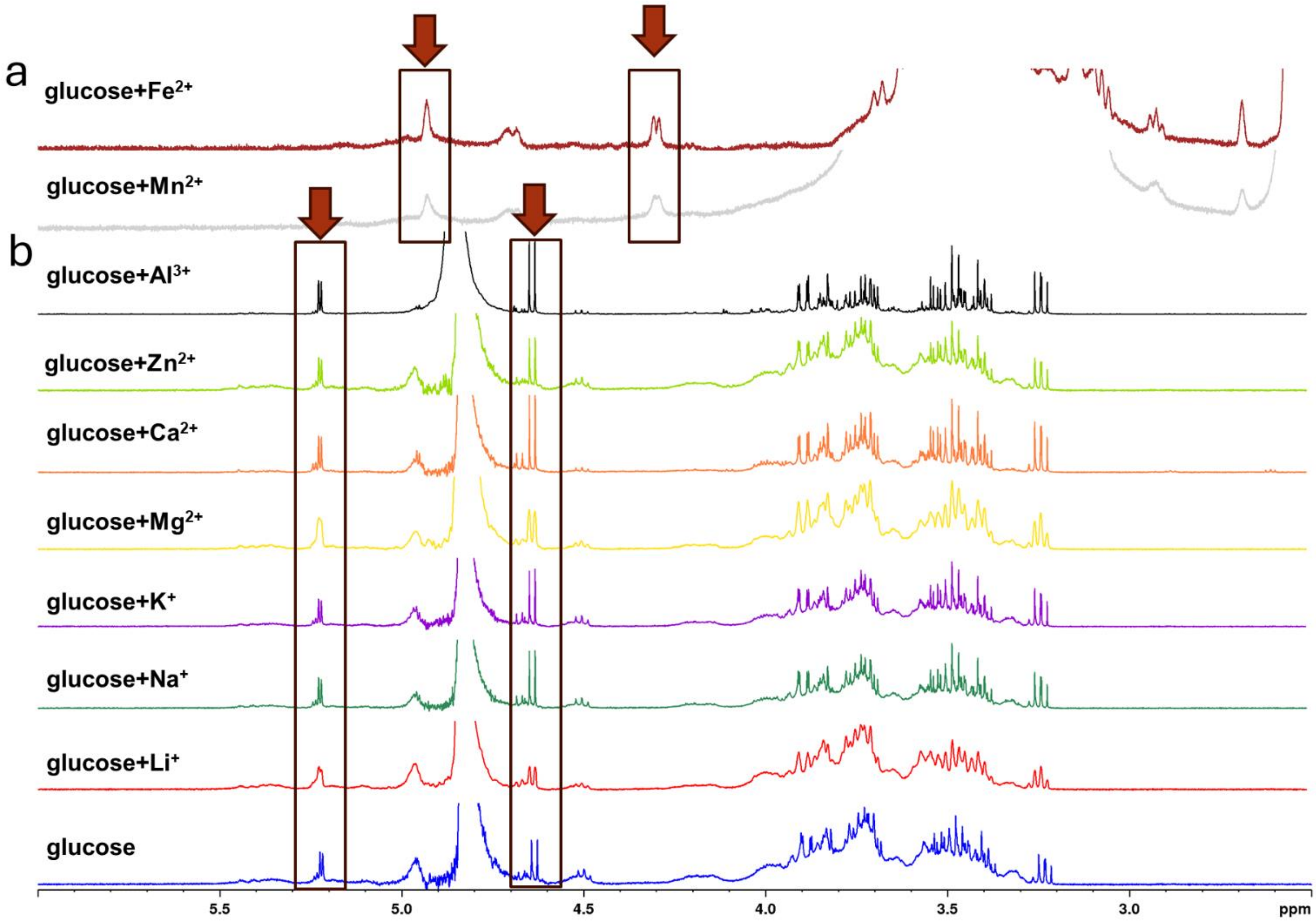

Figure S122. ^{1}H-NMR spectra of dry-down reaction of glucose in the absence or presence of various metal ions under acidified conditions. Solutions were prepared following drying at 50˚C for seven days. The products were dissolved in DMSO-d_6 (a) or D_2O buffer (pH=2.66) (b).

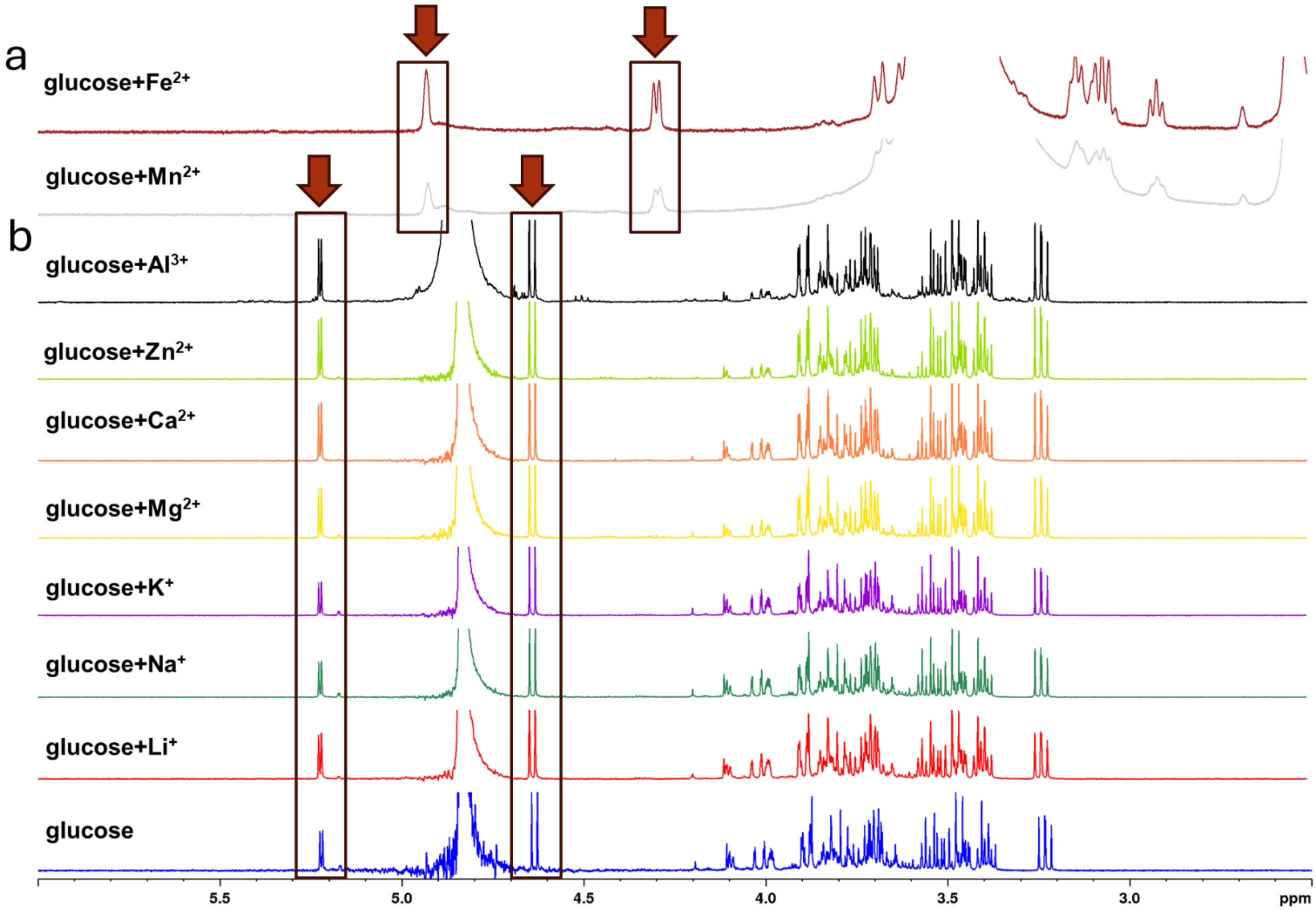

Figure S123. ^{1}H-NMR spectra of dry-down reaction of glucose in the absence or presence of various metal ions upon addition of NaOH. Solutions were prepared following drying at 50˚C for seven days. The products were dissolved in DMSO-d_6 (a) or D_2O buffer (pH=2.66) (b).

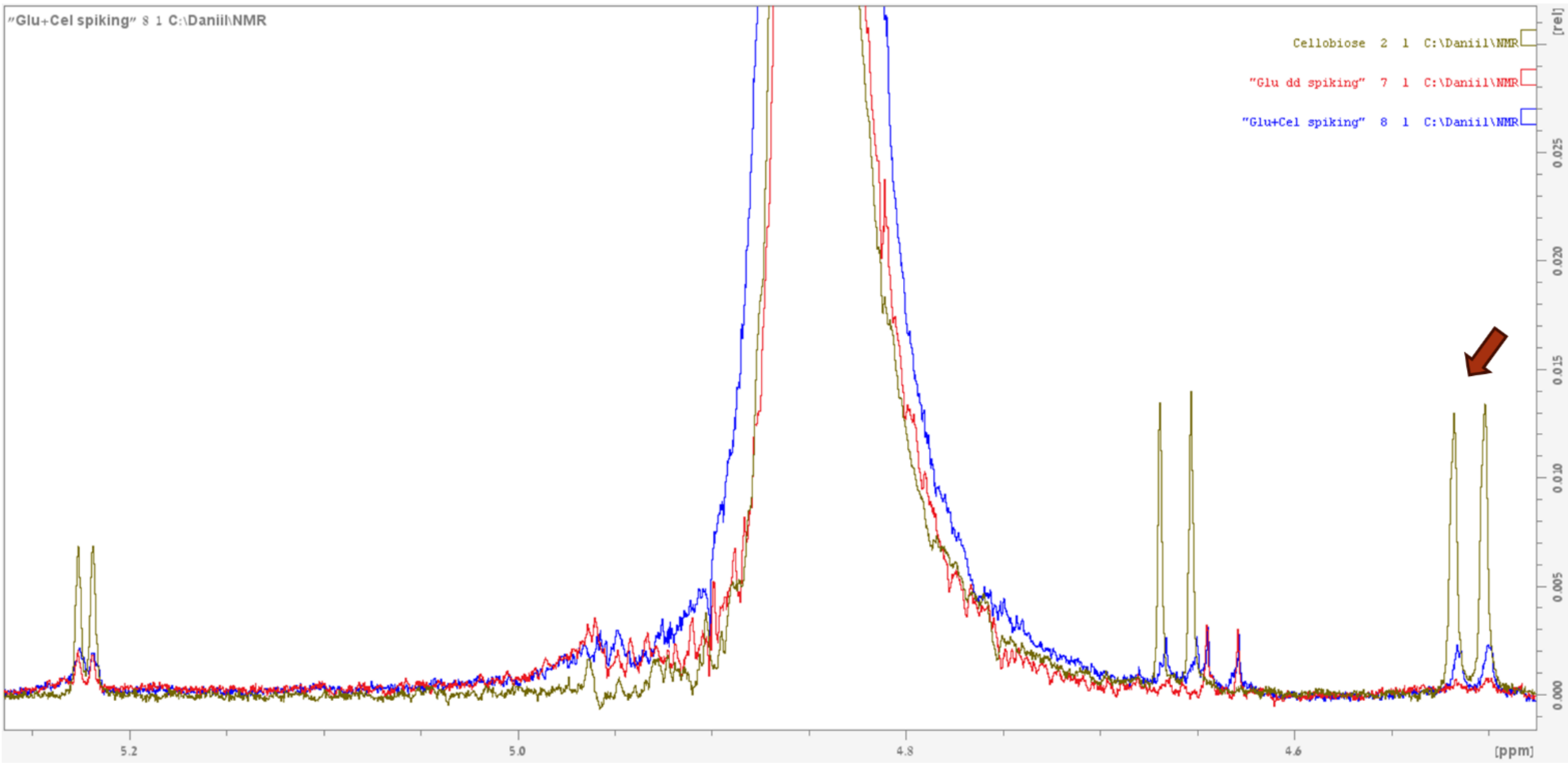

Figure S124. Overlay of ^{1}H-NMR spectra of a cellobiose standard (green), a dry-down reaction of glucose at 50˚C for seven days under acidified conditions (red), and the dry-down reaction of glucose that was spiked with cellobiose (blue). Samples were dissolved in D_2O buffer (pH=2.66). Arrows indicate overlaps of the new product peaks of the anomeric protons with the standard.

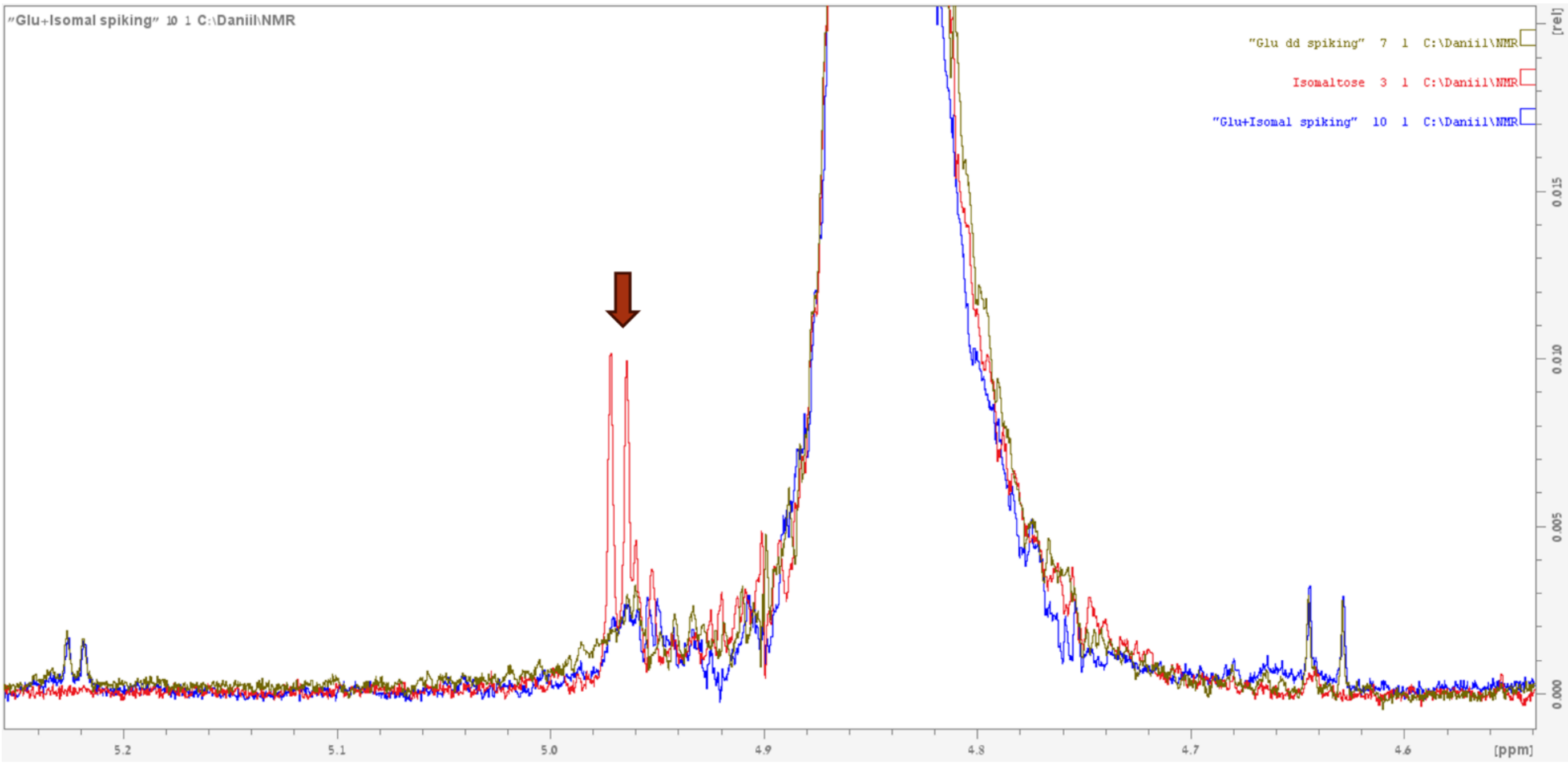

Figure S125. Overlay of ^{1}H-NMR spectra of an isomaltose standard (red), a dry-down reaction of glucose at 50˚C for seven days under acidified conditions (green), and the dry-down reaction of glucose that was spiked with isomaltose (blue). Samples were dissolved in D_2O buffer (pH=2.66). Arrows indicate overlaps of the new product peaks of the anomeric protons with the standard.

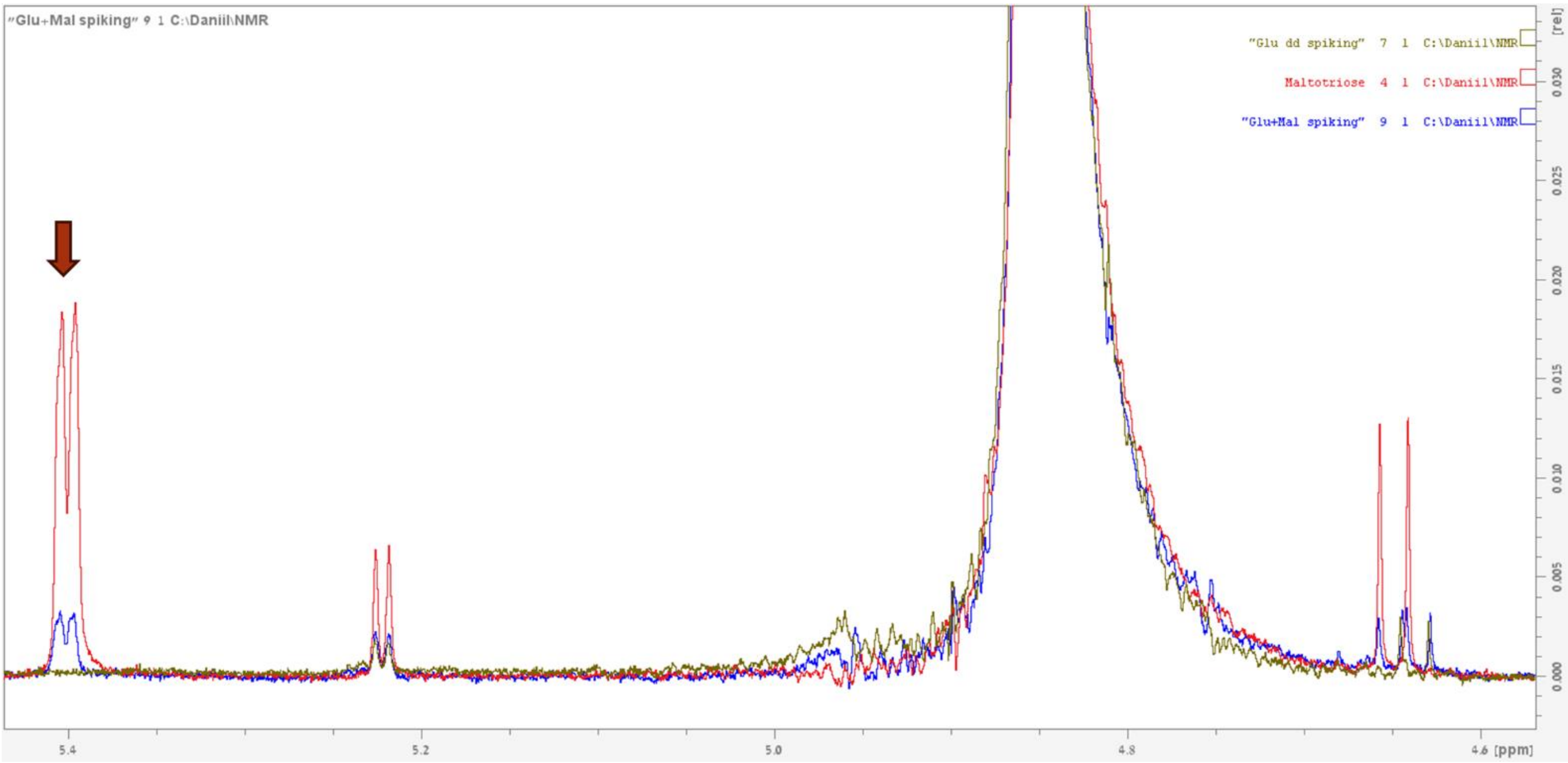

Figure S126. Overlay of ^{1}H-NMR spectra of a maltotriose standard (red), a dry-down reaction of glucose at 50˚C for seven days under acidified conditions (green), and the dry-down reaction of glucose that was spiked with maltotriose (blue). Samples were dissolved in D_2O buffer (pH=2.66). Arrows indicate overlaps of the new product peaks of the anomeric protons with the standard.

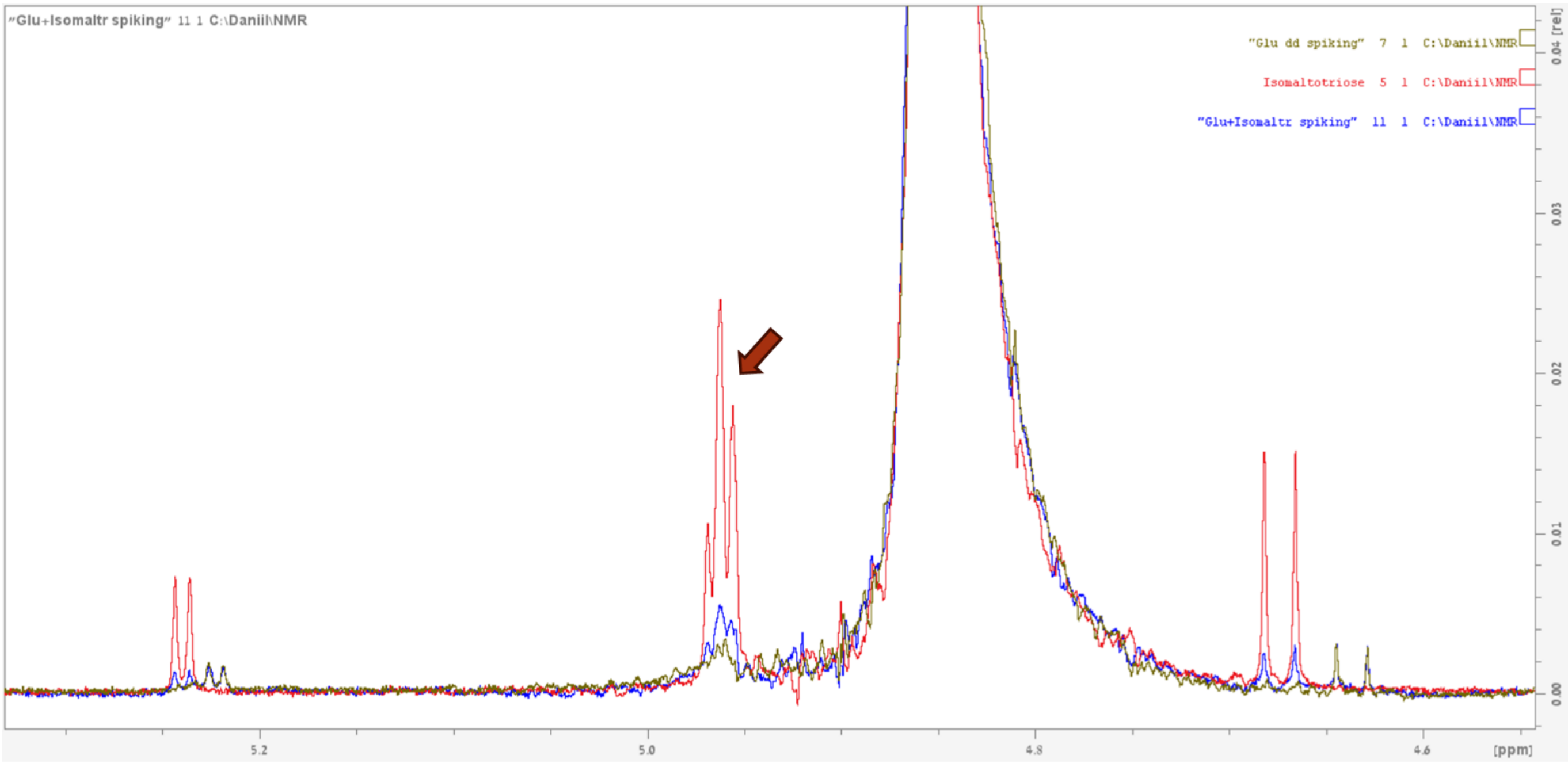

Figure S127. Overlay of ^{1}H-NMR spectra of an isomaltotriose standard (red), a dry-down reaction of glucose at 50˚C for seven days under acidified conditions (green), and the dry-down reaction of glucose that was spiked with isomaltotriose (blue). Samples were dissolved in D_2O buffer (pH=2.66). Arrows indicate overlaps of the new product peaks of the anomeric protons with the standard.

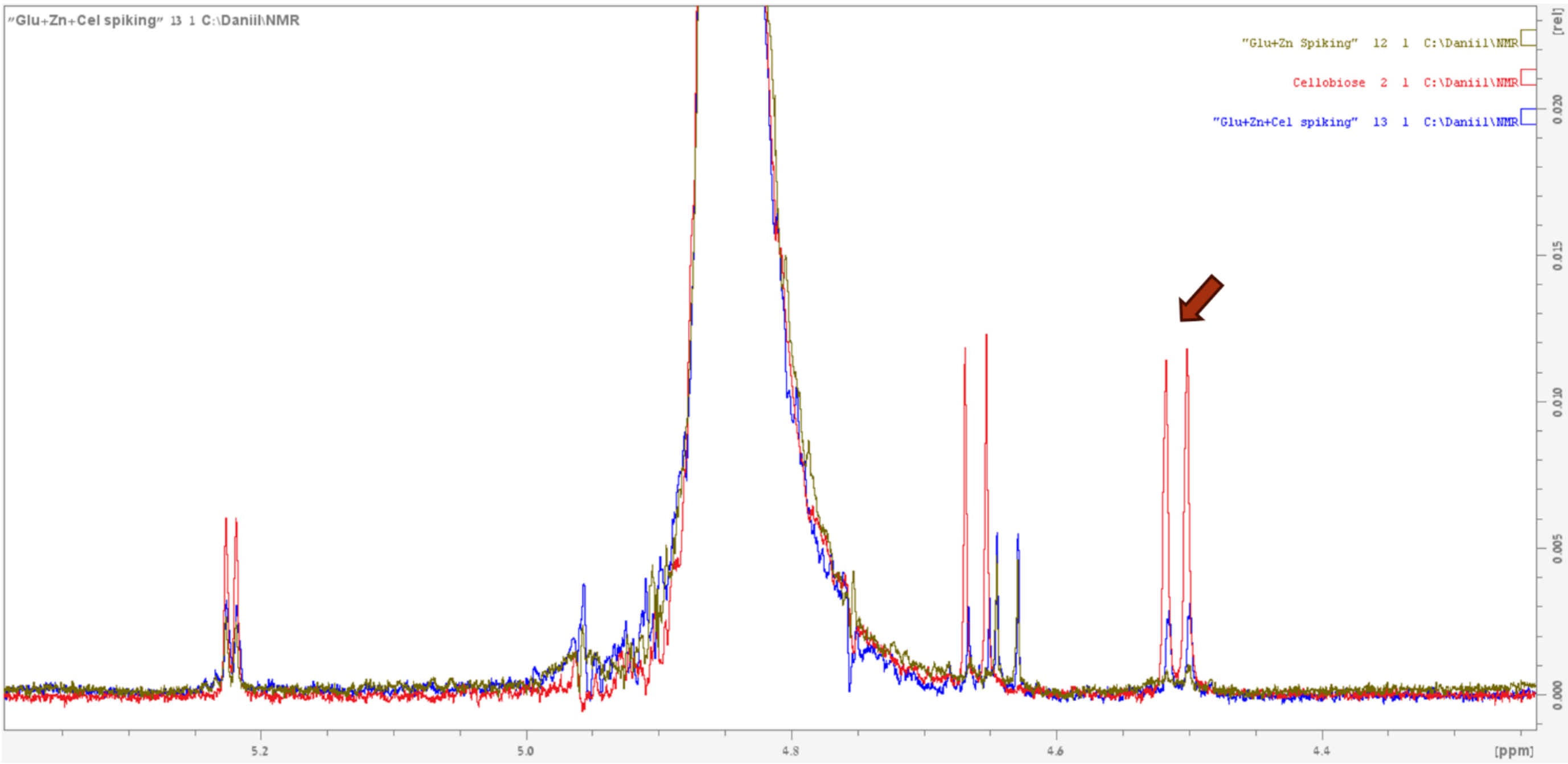

Figure S128. Overlay of ^{1}H-NMR spectra of a cellobiose standard (red), a dry-down reaction of glucose at 50˚C for seven days under acidified conditions in the presence of $ZnCl_2$ (green), and the dry-down reaction of glucose that was spiked with cellobiose (blue). Samples were dissolved in D_2O buffer (pH=2.66). Arrows indicate overlaps of the new product peaks of the anomeric protons with the standard.

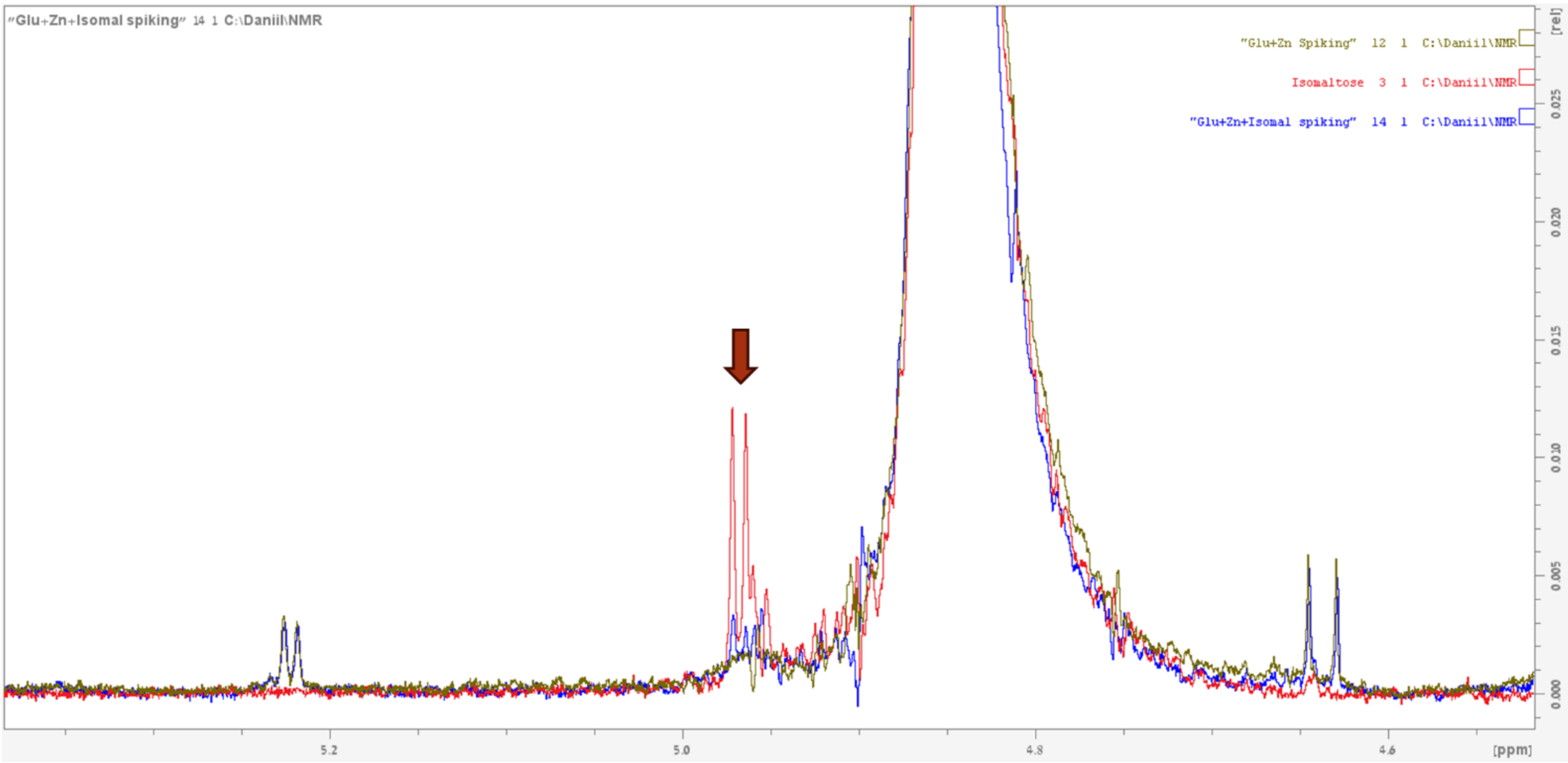

Figure S129. Overlay of ^{1}H-NMR spectra of an isomaltose standard (red), a dry-down reaction of glucose at 50˚C for seven days under acidified conditions in the presence of $ZnCl_2$ (green), and the dry-down reaction of glucose that was spiked with isomaltose (blue). Samples were dissolved in D_2O buffer (pH=2.66). Arrows indicate overlaps of the new product peaks of the anomeric protons with the standard.

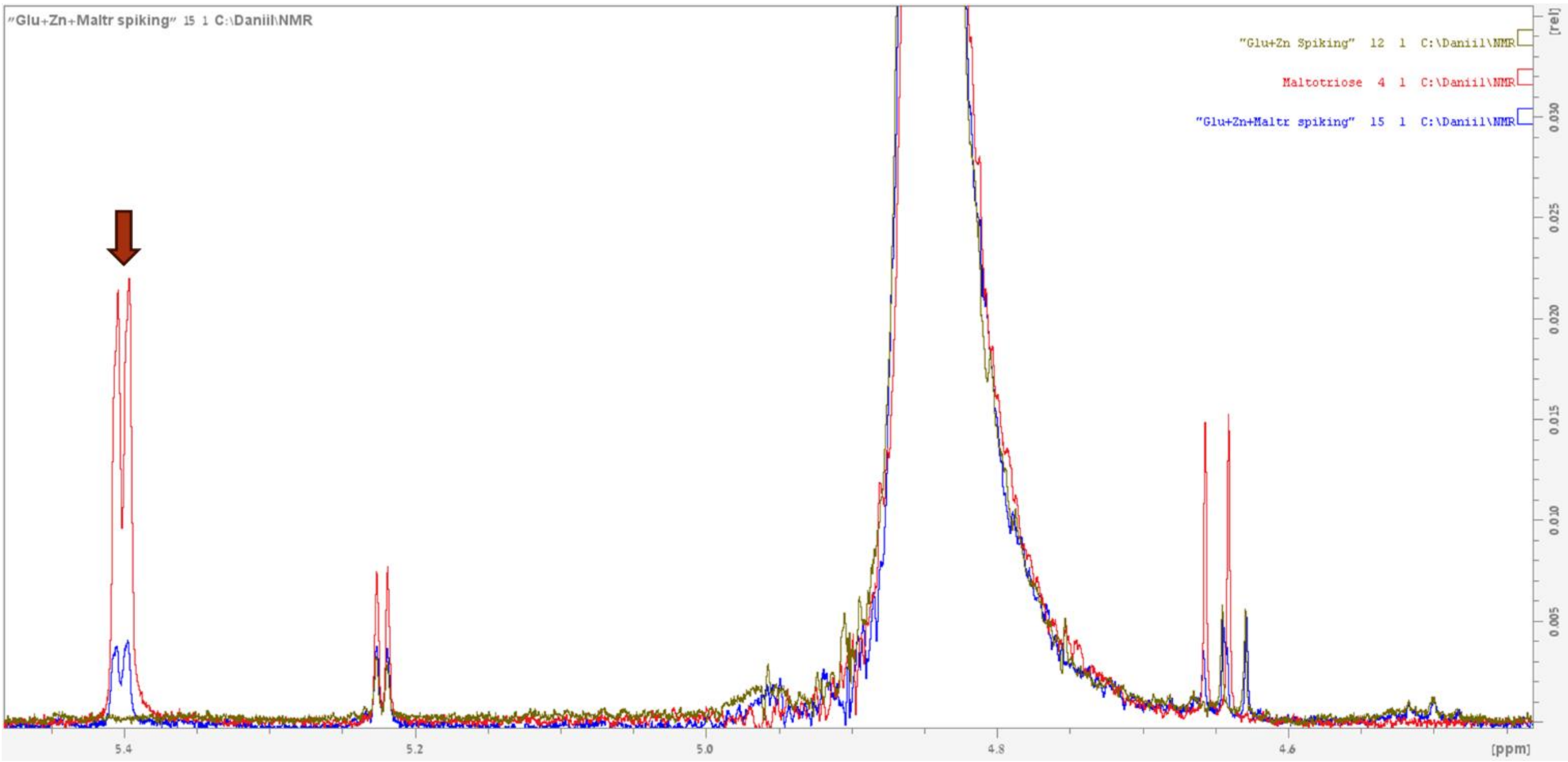

Figure S130. Overlay of ^{1}H-NMR spectra of a maltotriose standard (red), a dry-down reaction of glucose at 50˚C for seven days under acidified conditions in the presence of $ZnCl_2$ (green), and the dry-down reaction of glucose that was spiked with maltotriose (blue). Samples were dissolved in D_2O buffer (pH=2.66). Arrows indicate overlaps of the new product peaks of the anomeric protons with the standard.

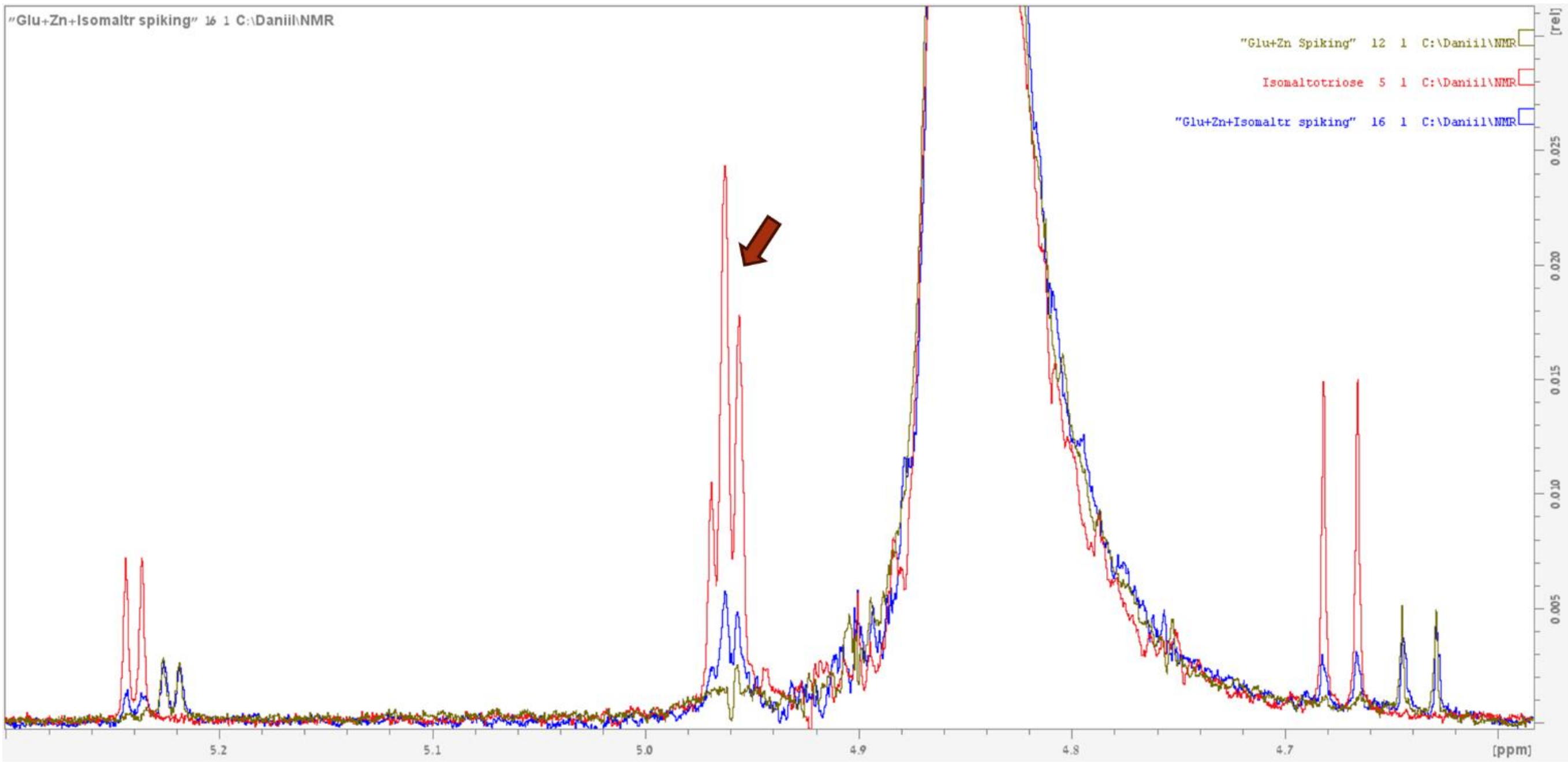

Figure S131. Overlay of ^{1}H-NMR spectra of an isomaltotriose standard (red), a dry-down reaction of glucose at 50˚C for seven days under acidified conditions in the presence of $ZnCl_2$ (green), and the dry-down reaction of glucose that was spiked with isomaltotriose (blue). Samples were dissolved in D_2O buffer (pH=2.66). Arrows indicate overlaps of the new prodcut peaks of the anomeric protons with the standard.

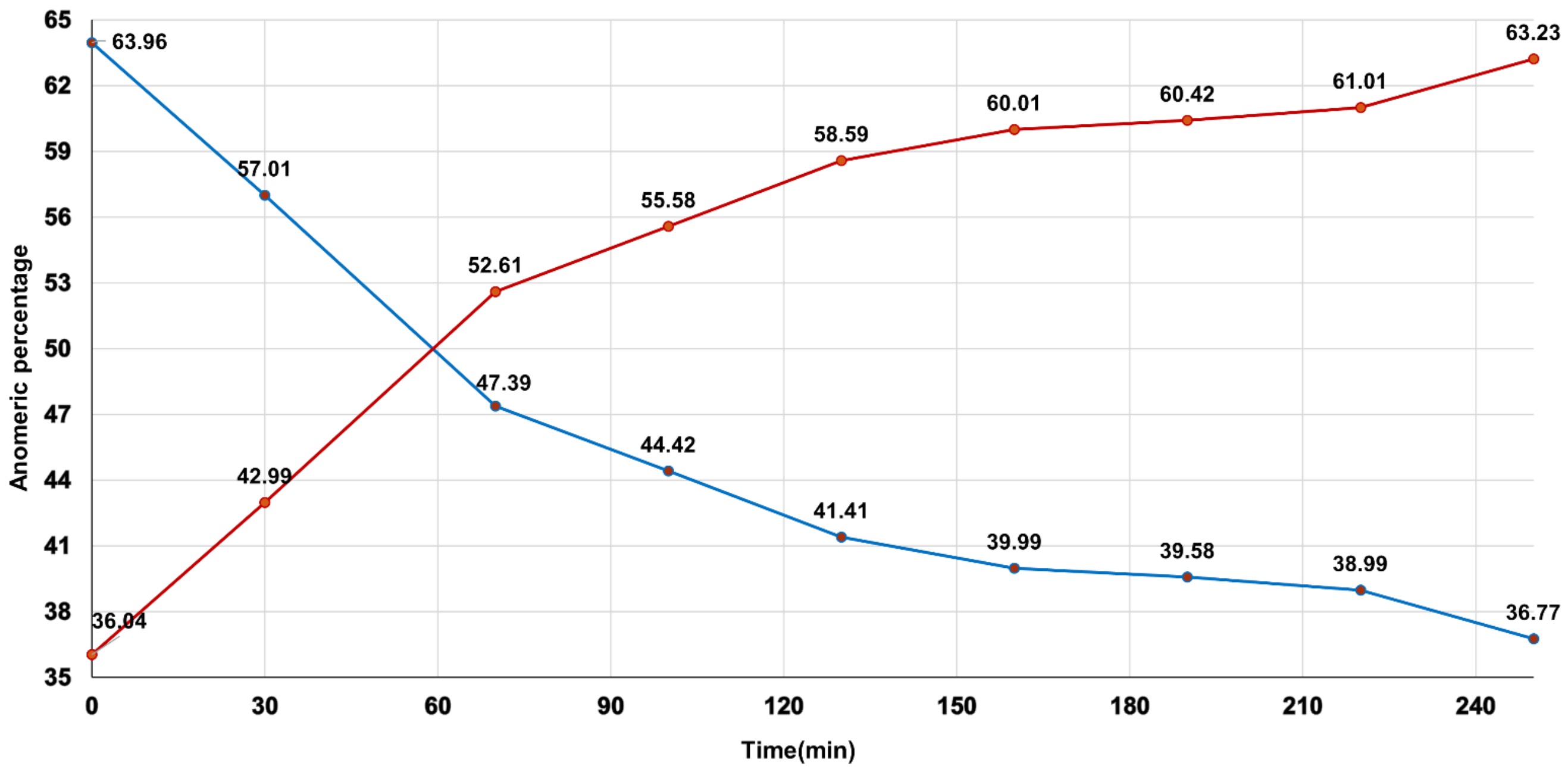

Figure S132. Kinetics of fresh D-Glucose mutarotation in the presence of Ca^{2+} under acidified conditions. The percentage distribution of the α-anomer (blue) and β-anomer (red) is shown over time upon mixing of D-Glucose with $CaCl_2$. $CaCl_2$ was added to glucose in a ratio of 1:1 under acidified conditions. Samples were dissolved in D_2O buffer (pH=2.66).

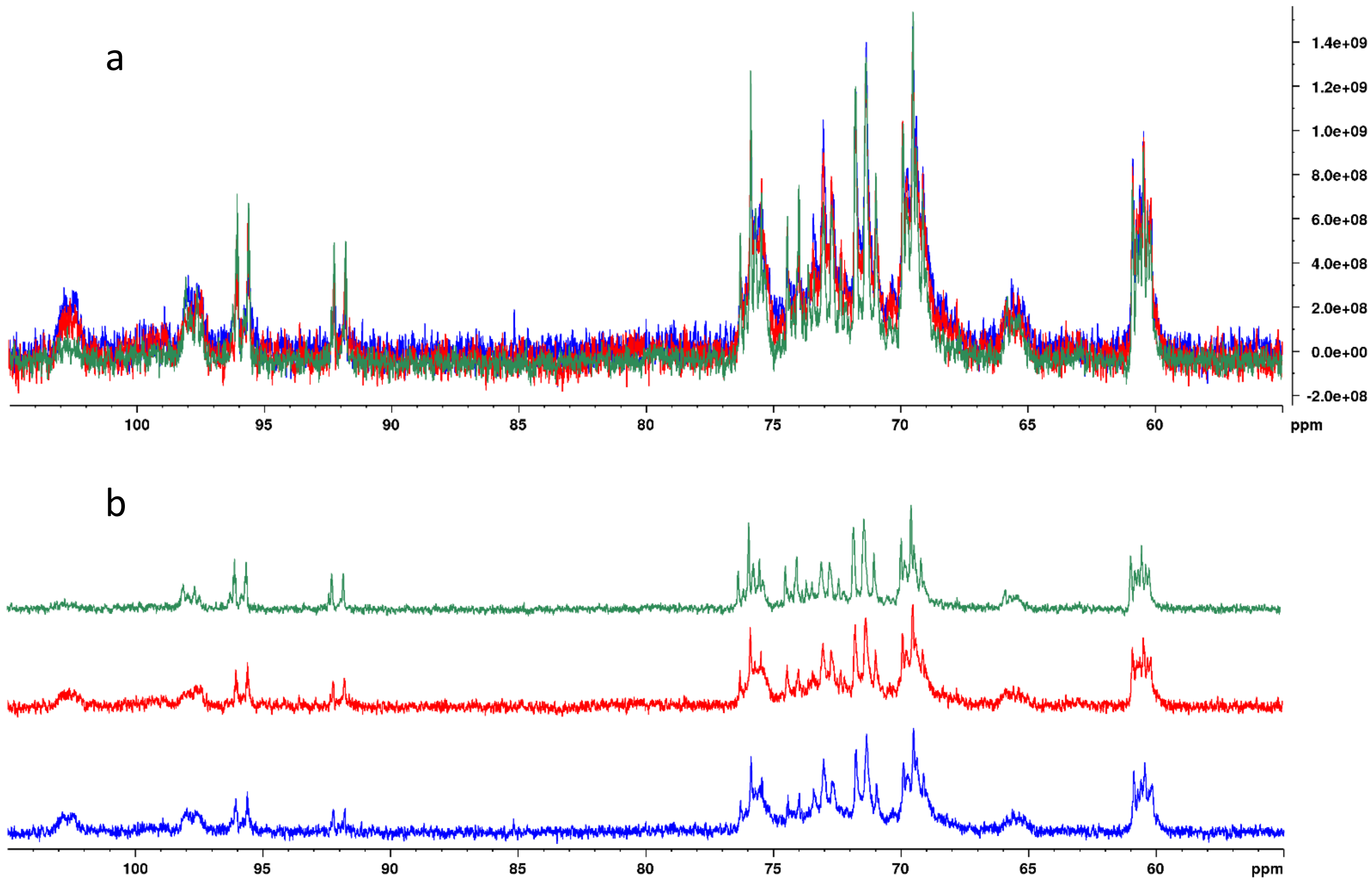

Figure S133. ^{13}C-NMR spectra of dry-down reactions of D-Glucose-$^{13}C6$ under acidified conditions. D-Glucose-$^{13}C6$ was dried either in the absence of metal ions(green) or in the presence of $ZnCl_2$ (red) or $CaCl_2$ (blue) at 50 ˚C for seven days. Overlay (a) and stack spectra (b) of the three samples are shown.

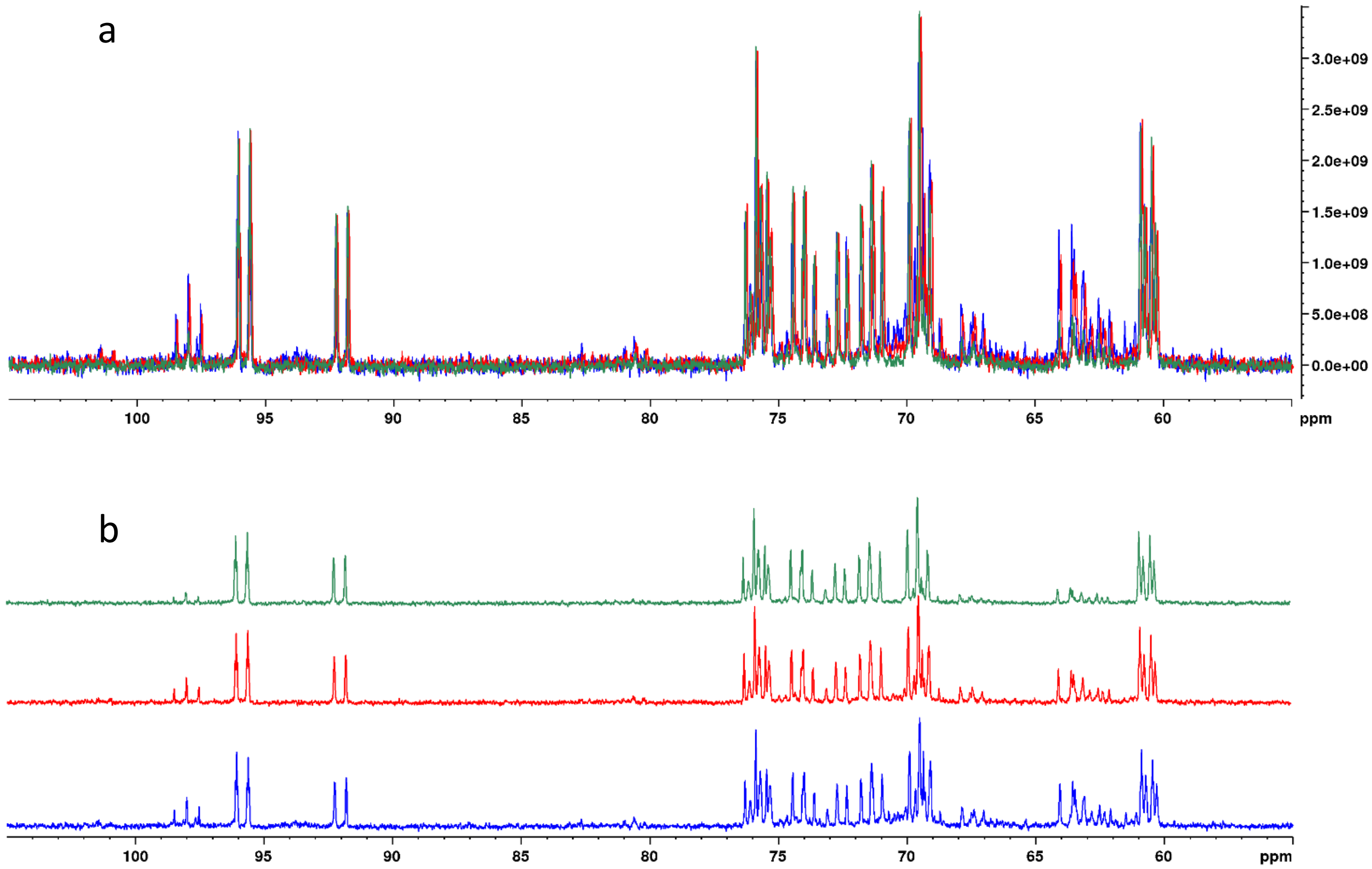

Figure S134. ^{13}C-NMR spectra of dry-down reactions of D-Glucose-^{13}C6 upon addition of NaOH. D-Glucose-^{13}C6 was dried either in the absence of metal ions(green) or in the presence of $ZnCl_2$ (red) or $CaCl_2$ (blue) at 50 ˚C for seven days. Overlay (a) and stack spectra (b) of the three samples are shown.

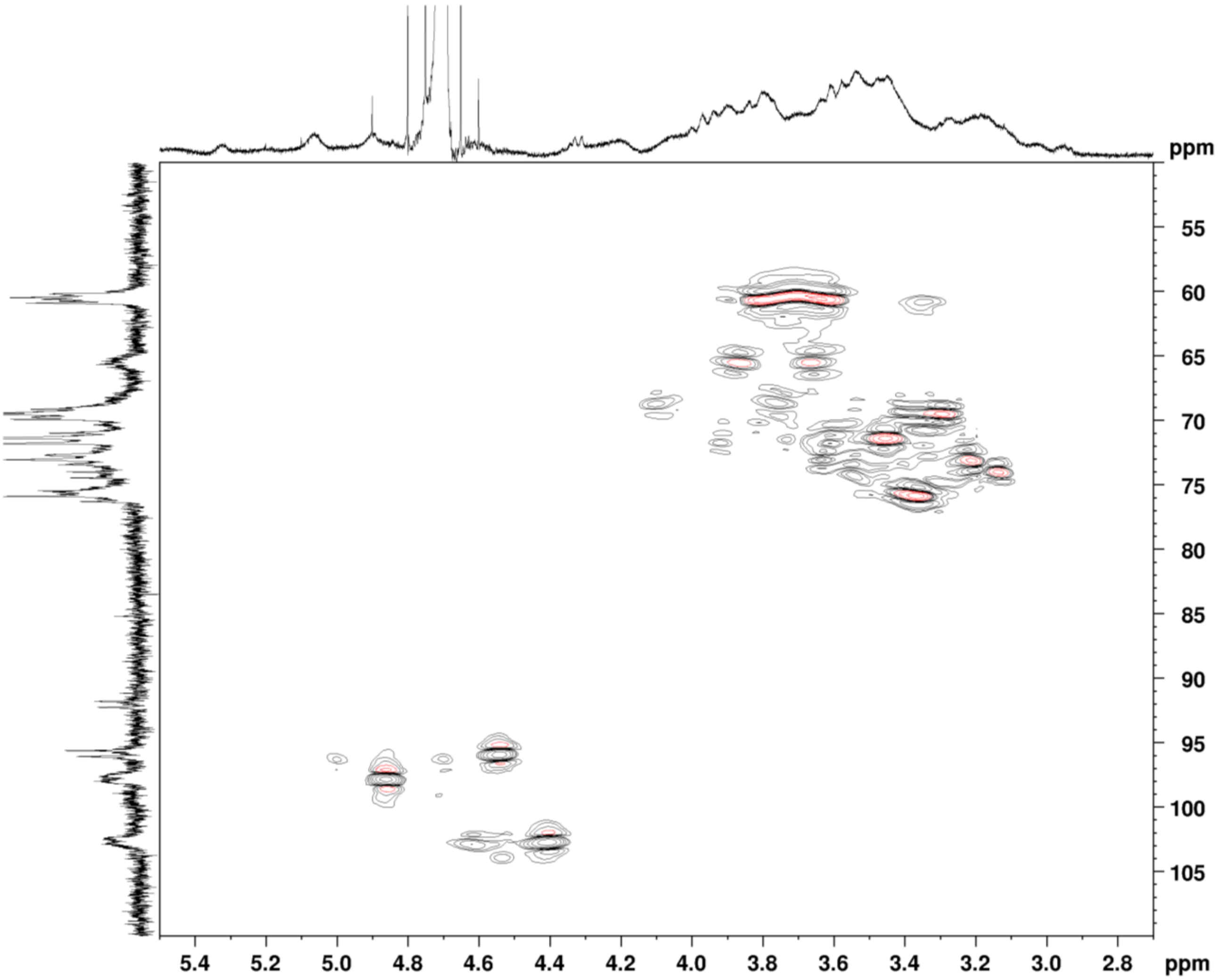

Figure S135. HSQC spectra of dry-down reaction of D-Glucose-$^{13}C6$ in the absence of metal ions under acidified conditions. Solution was prepared following drying at 50˚C for seven days. The products were dissolved in D_2O buffer (pH=2.66).

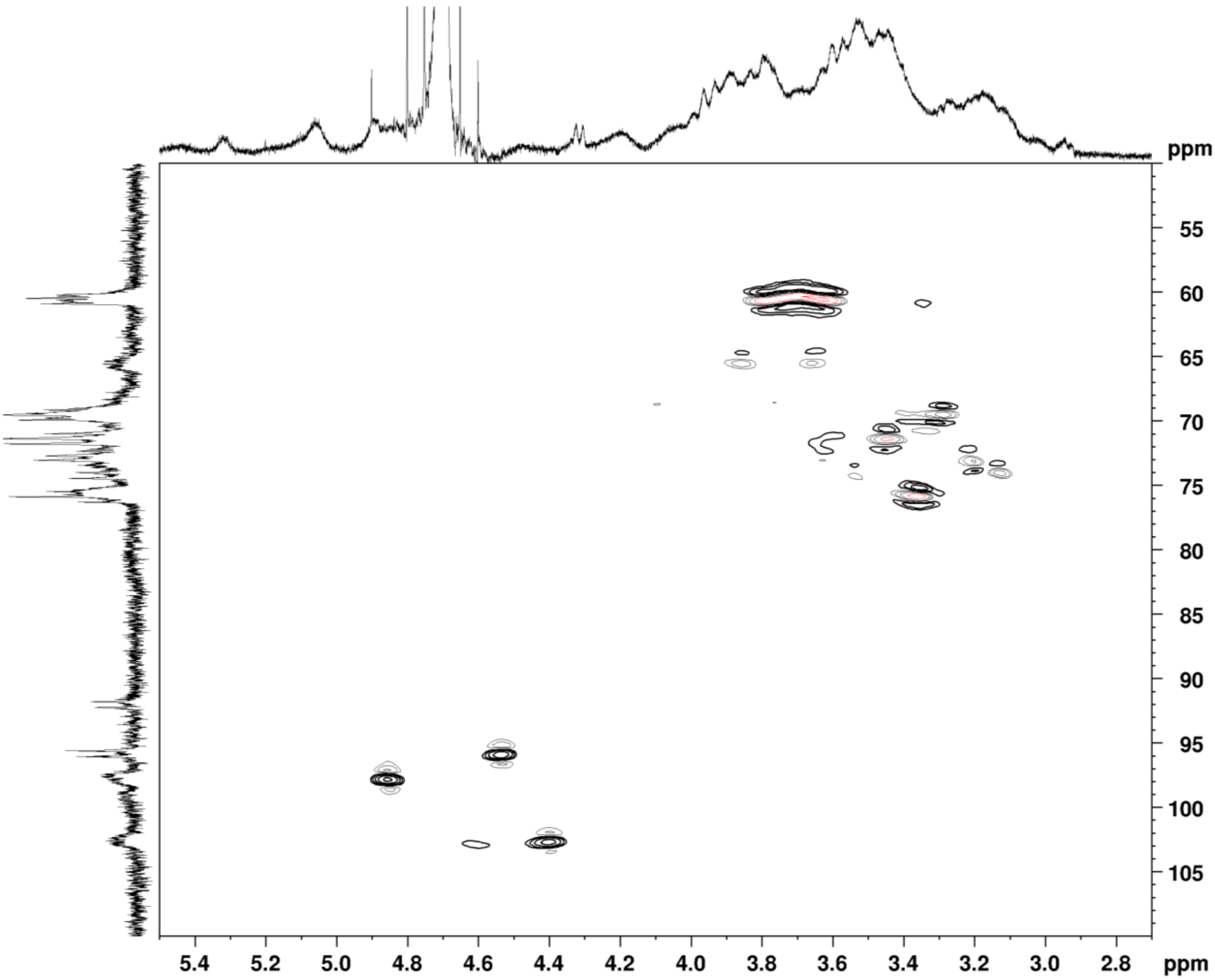

Figure S136. HSQC spectra of dry-down reaction of D-Glucose-$^{13}C6$ in the presence of $ZnCl_2$ under acidified conditions. Solution was prepared following drying at 50˚C for seven days. The products were dissolved in D_2O buffer (pH=2.66).

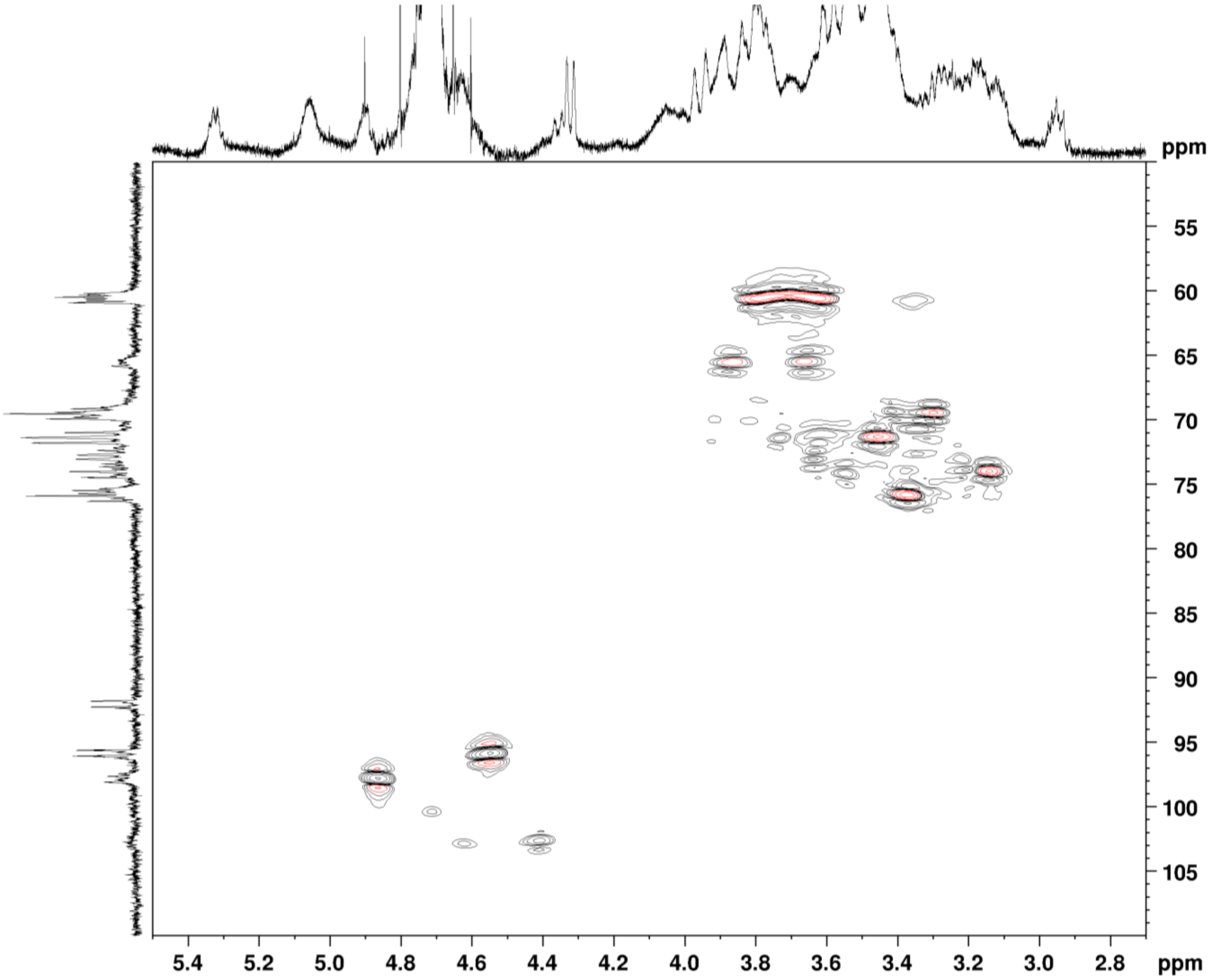

Figure S137. HSQC spectra of dry-down reaction of D-Glucose-^{13}C6 in the presence of $CaCl_2$ under acidified conditions. Solution was prepared following drying at 50˚C for seven days. The products were dissolved in D_2O buffer (pH=2.66).

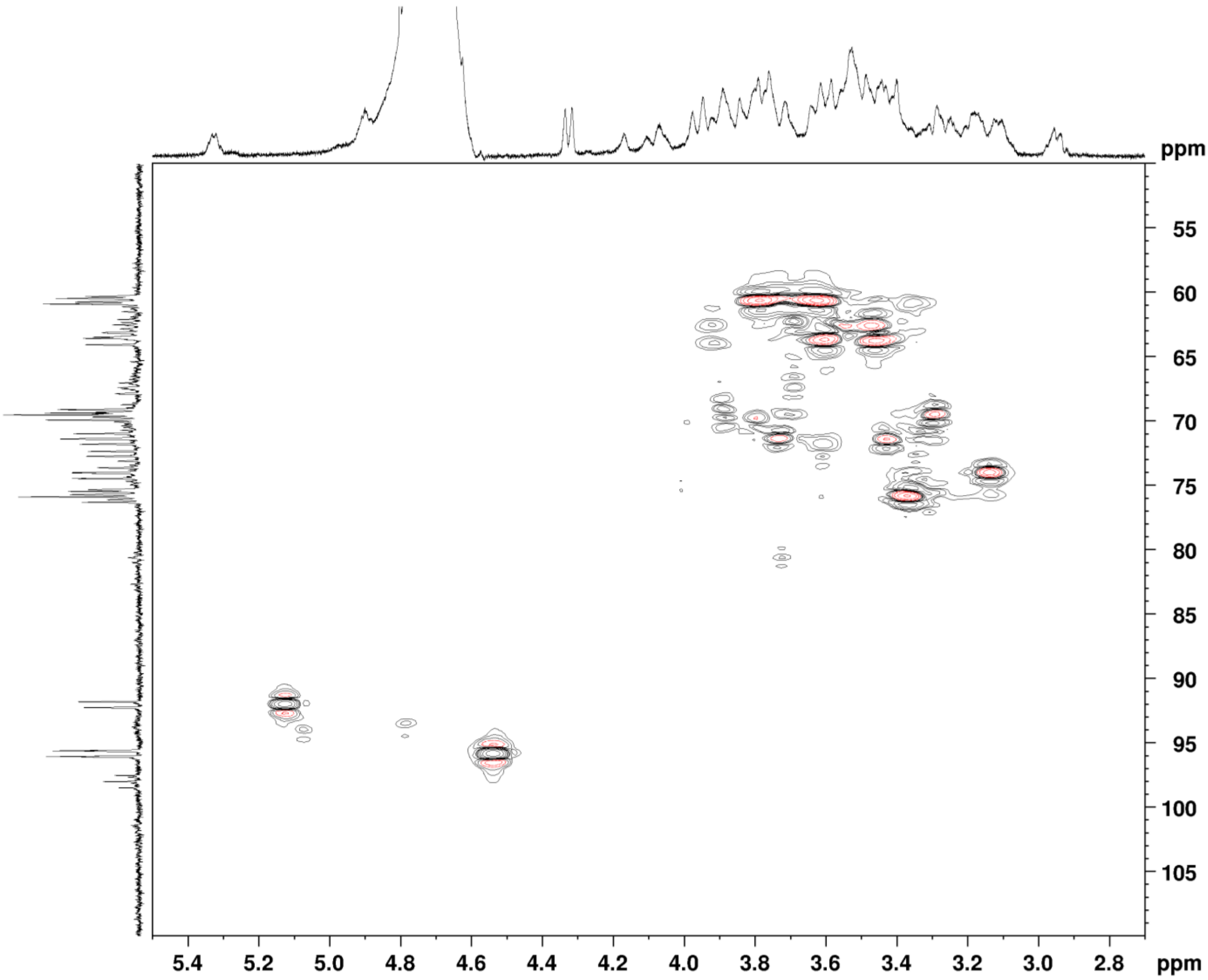

Figure S138. HSQC spectra of dry-down reaction of D-Glucose-$^{13}C6$ in the absence of metal ions upon addition of NaOH. Solution was prepared following drying at 50˚C for seven days. The products were dissolved in D_2O buffer (pH=2.66).

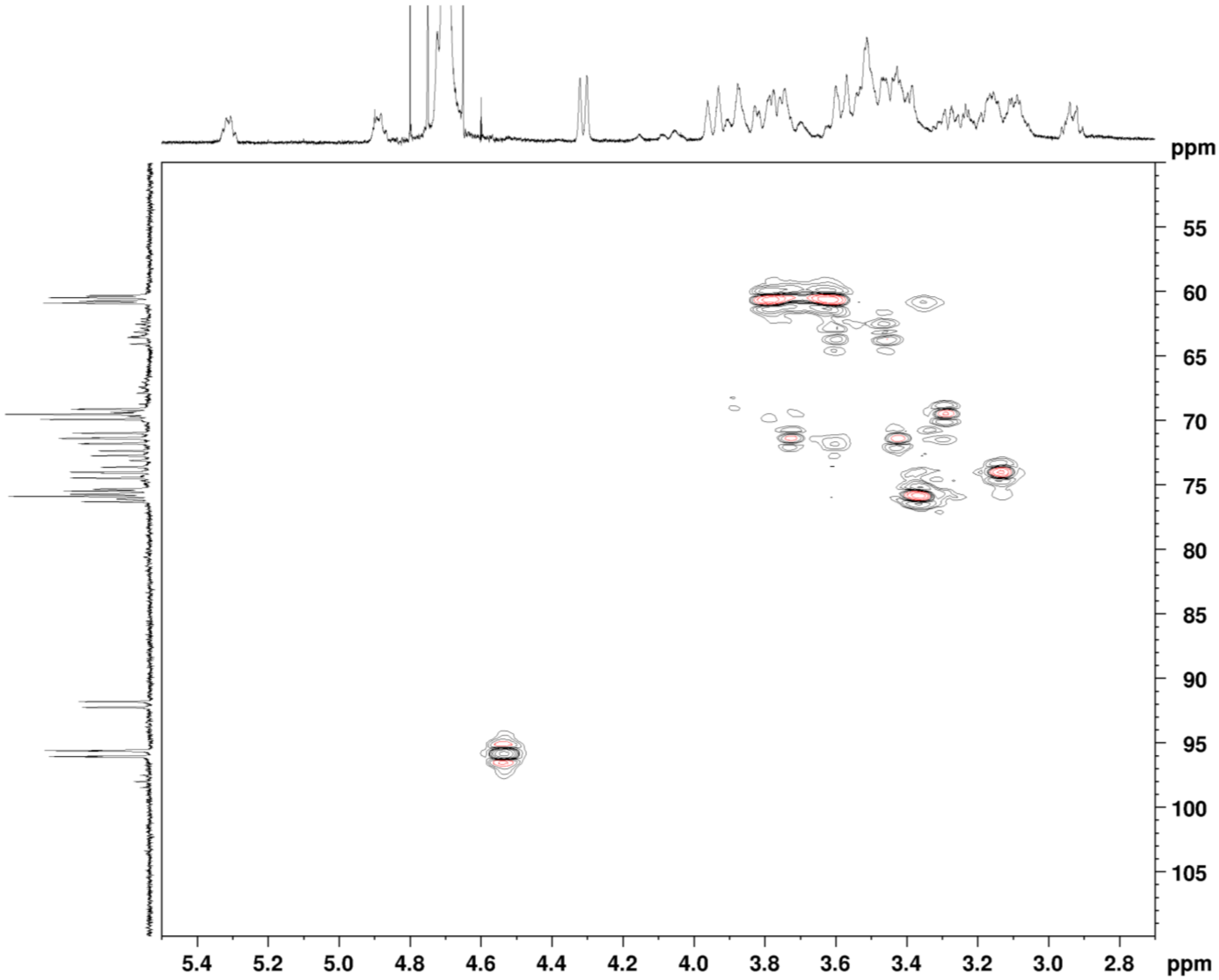

Figure S139. HSQC spectra of dry-down reaction of D-Glucose-[13]C6 in the presence of $ZnCl_2$ upon addition of NaOH. Solution was prepared following drying at 50˚C for seven days. The products were dissolved in D_2O buffer (pH=2.66).

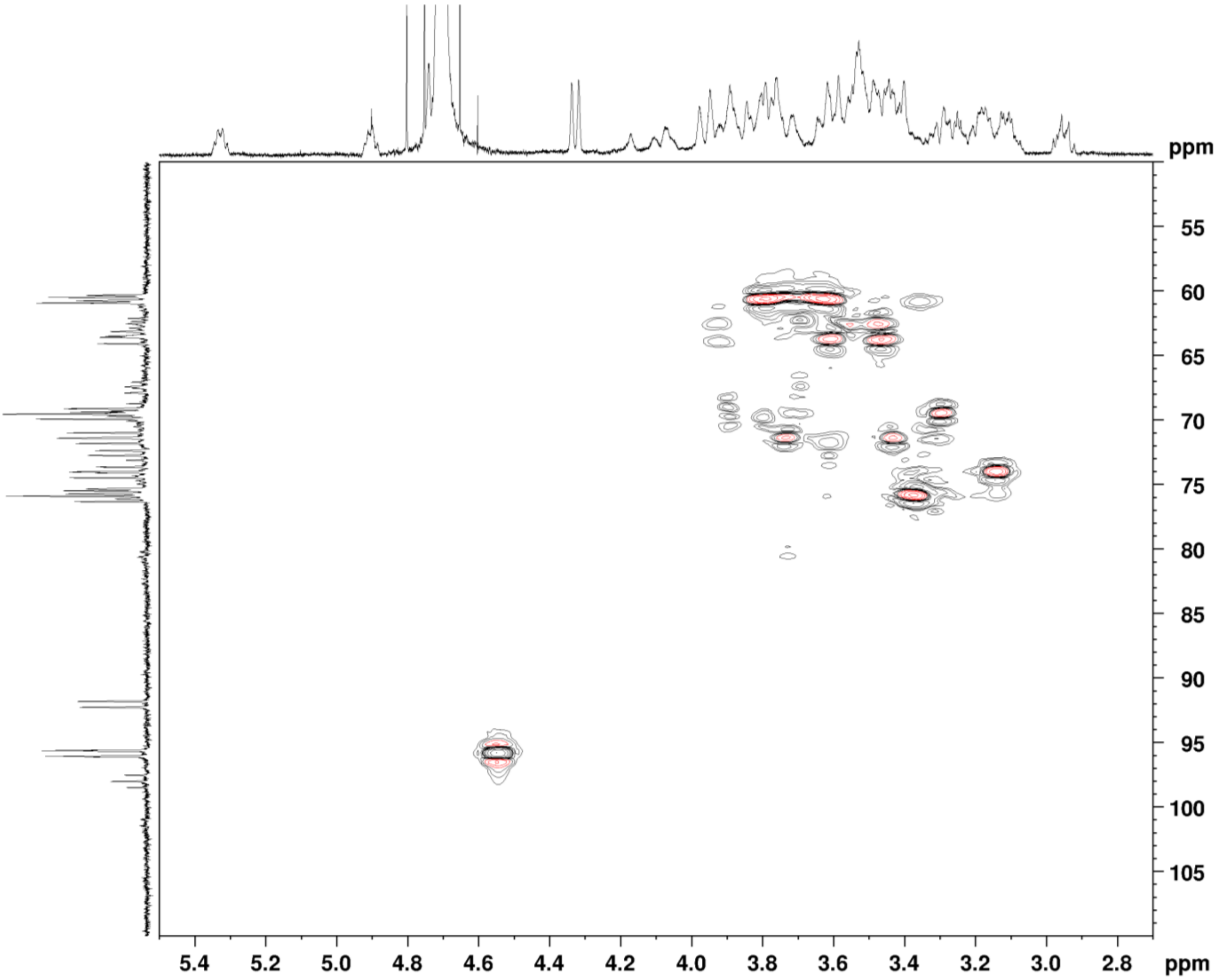

Figure S140. HSQC spectra of dry-down reaction of D-Glucose-[13]C6 in the presence of $CaCl_2$ upon addition of NaOH. Solution was prepared following drying at 50˚C for seven days. The products were dissolved in D_2O buffer (pH=2.66).

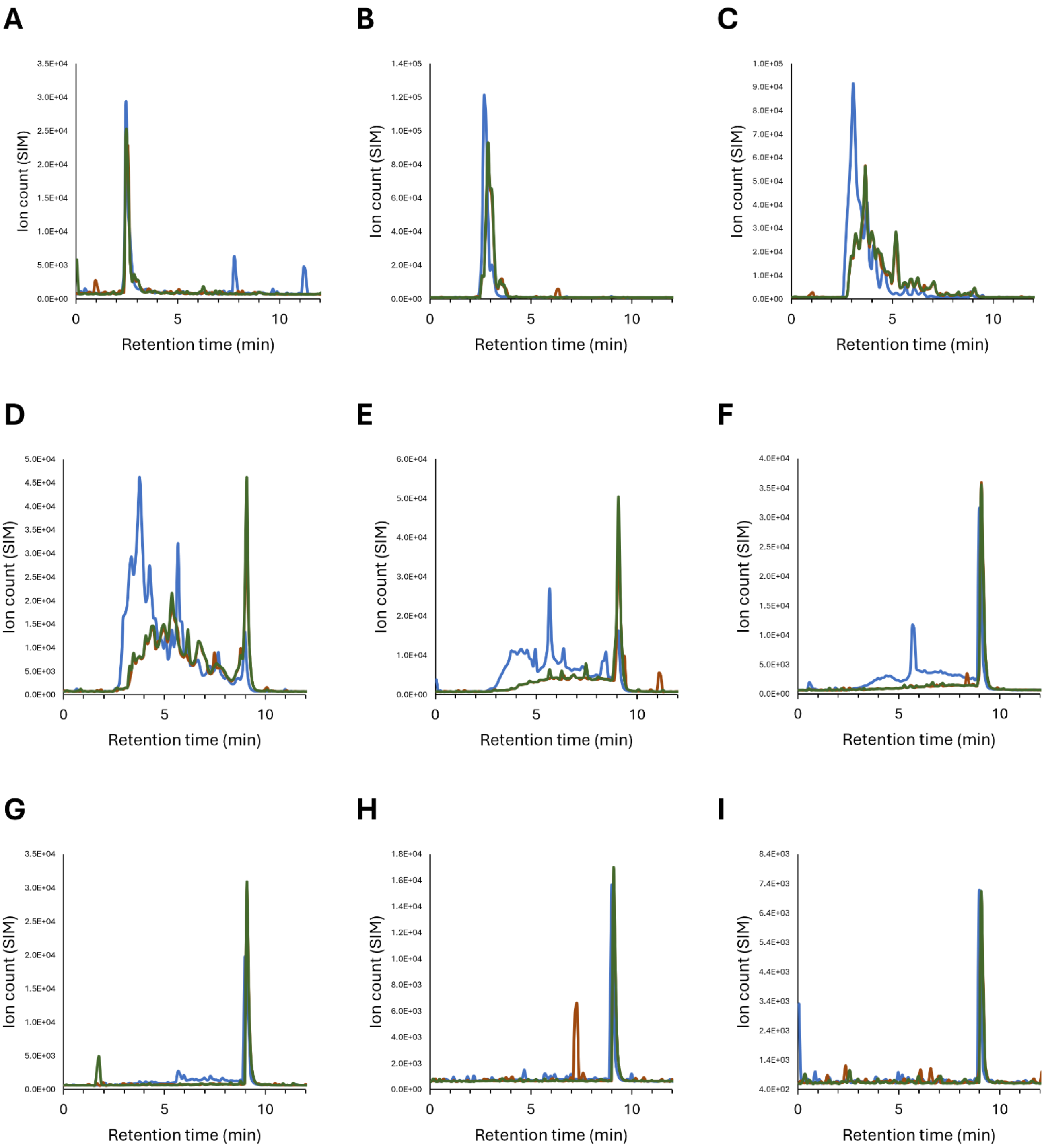

Figure S141. SIM-LC-MS of a dry down reaction of glucose (glu) under acidified conditions in the absence of metal ions at a 1mM final concentration (referring to initial glu concentration prior to the drying reaction). Glu was analyzed by negative mode SIM-LC-MS and the various species (monomer and its oligomers) were identified as [M-H]$^-$ ions, [M+formate]$^-$ ions, and [M+Cl]$^-$ ions (for monomers (A), dimers (B), trimers (C), tetramers (D), pentamers (E), hexamers (F), heptamers (G), octamers (H), and nonamers (I)).

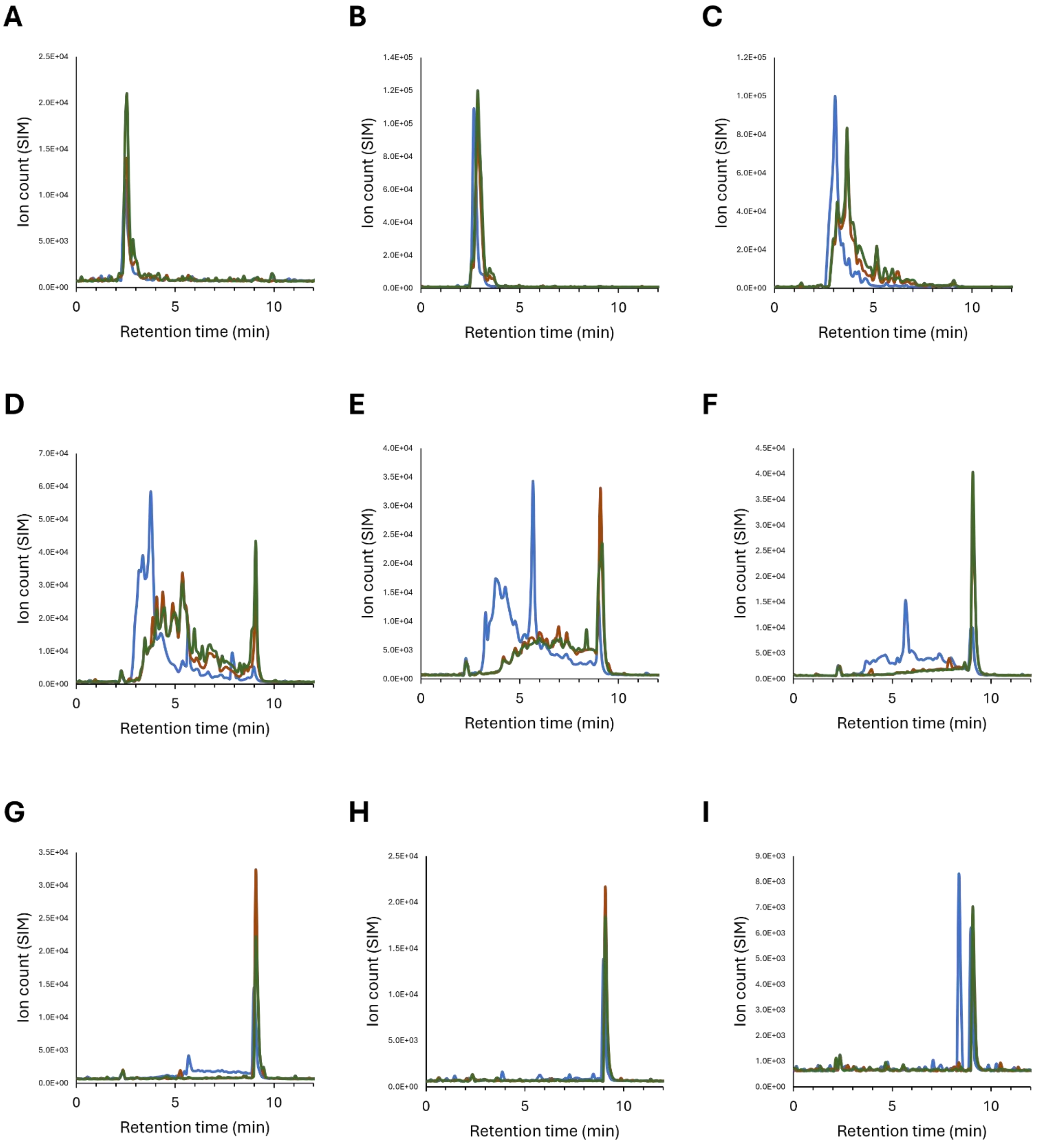

Figure S142. SIM-LC-MS of a dry down reaction of glucose (glu) under acidified conditions in the presence of LiCl at a 1mM final concentration (referring to initial glu concentration prior to the drying reaction). Glu was analyzed by negative mode SIM-LC-MS and oligomers were identified as [M-H]$^-$ ions, [M+formate]$^-$ ions, and [M+Cl]$^-$ ions (for monomers (A), dimers (B), trimers (C), tetramers (D), pentamers (E), hexamers (F), heptamers (G), octamers (H), and nonamers (I)).

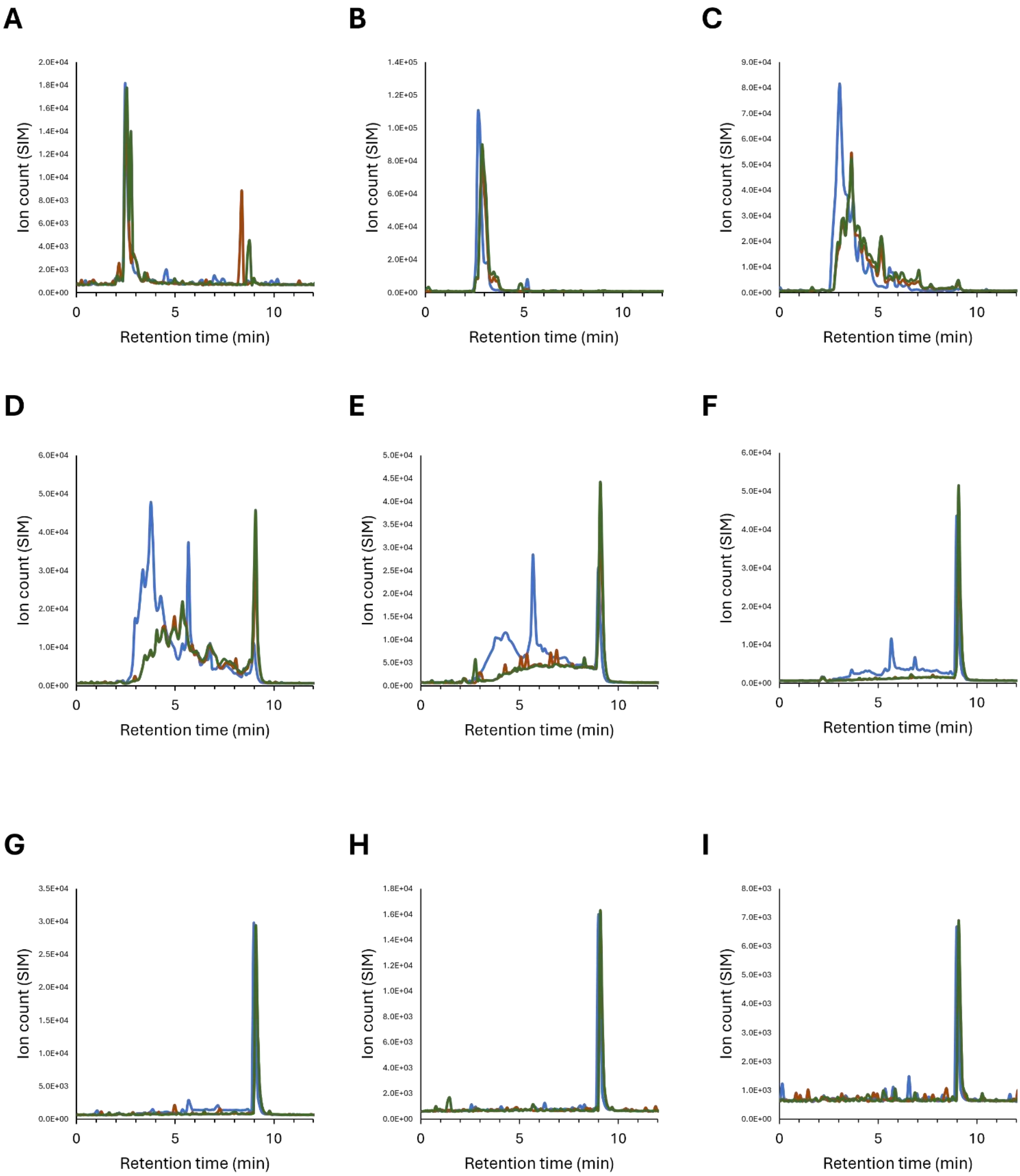

Figure S143. SIM-LC-MS of a dry down reaction of glucose (glu) under acidified conditions in the presence of $ZnCl_2$ at a 1mM final concentration (referring to initial glu concentration prior to the drying reaction). Glu was analyzed by negative mode SIM-LC-MS and oligomers were identified as $[M-H]^-$ ions, $[M+formate]^-$ ions, and $[M+Cl]^-$ ions (for monomers (A), dimers (B), trimers (C), tetramers (D), pentamers (E), hexamers (F), heptamers (G), octamers (H), and nonamers (I)).

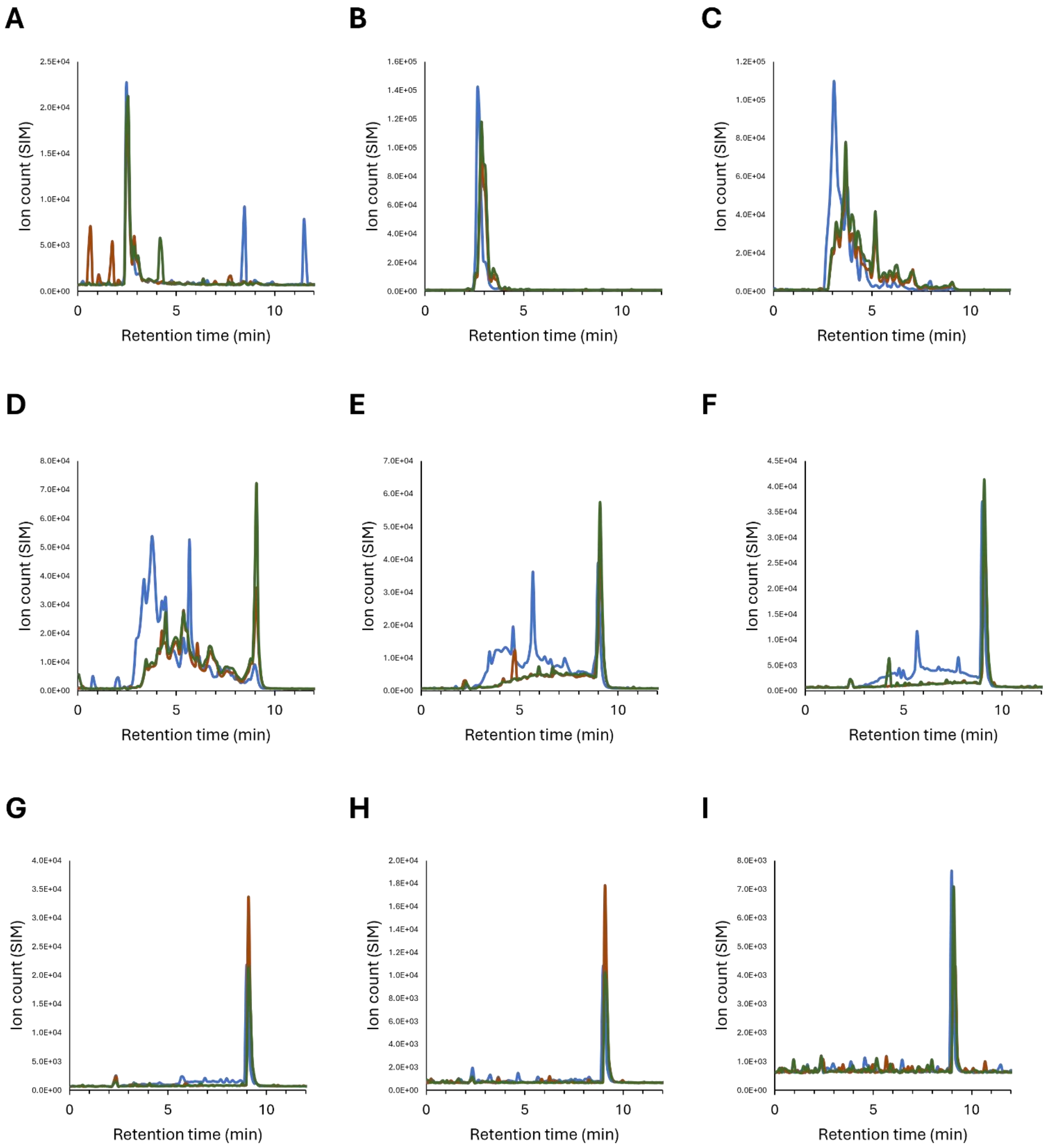

Figure S144. SIM-LC-MS of a dry down reaction of glucose (glu) under acidified conditions in the presence of NaCl at a 1mM final concentration (referring to initial glu concentration prior to the drying reaction). Glu was analyzed by negative mode SIM-LC-MS and oligomers were identified as $[M-H]^-$ ions, $[M+formate]^-$ ions, and $[M+Cl]^-$ ions (for monomers (A), dimers (B), trimers (C), tetramers (D), pentamers (E), hexamers (F), heptamers (G), octamers (H), and nonamers (I)

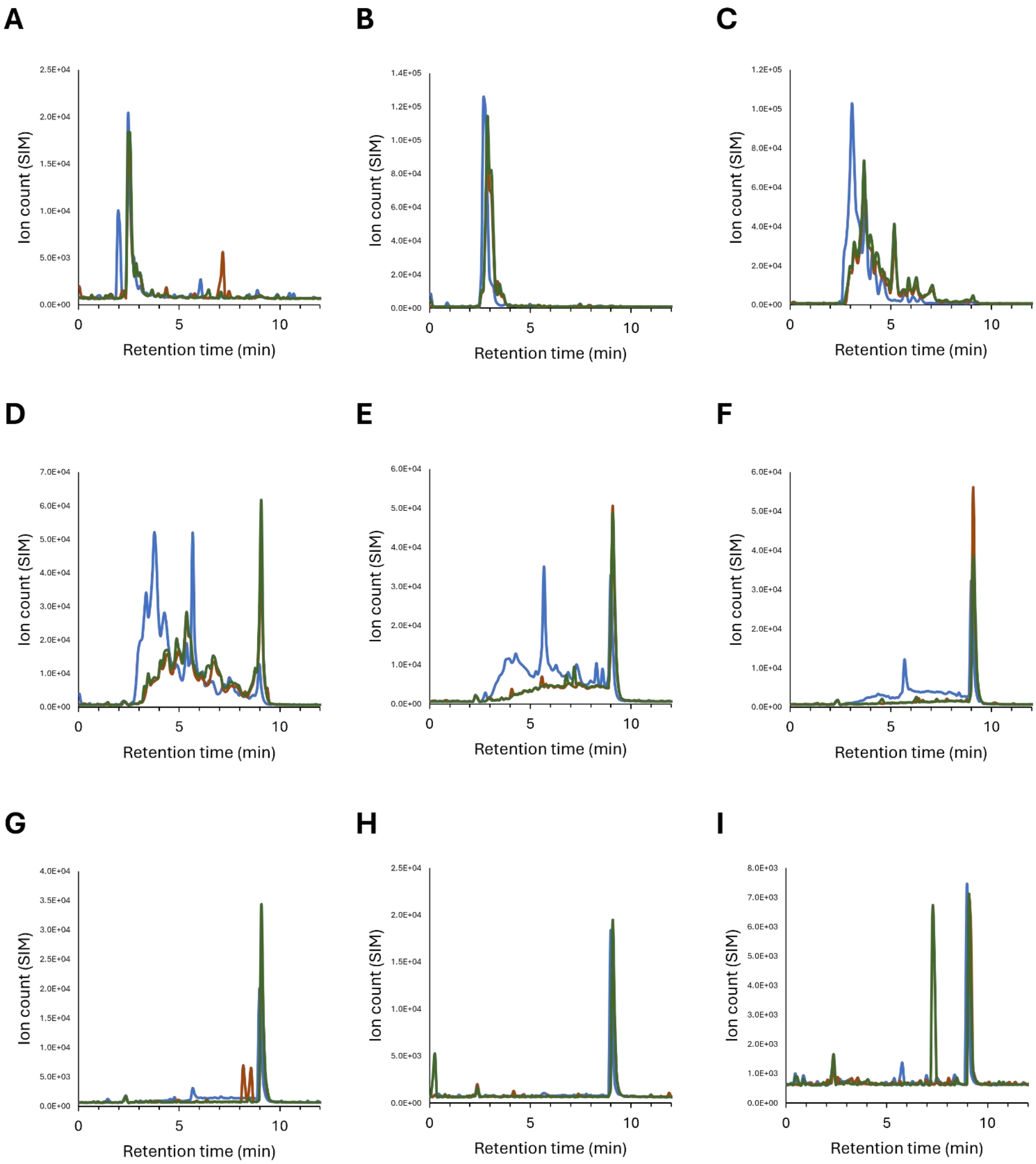

Figure S145. SIM-LC-MS of a dry down reaction of glucose (glu) under acidified conditions in the presence of KCl at a 1mM final concentration (referring to initial glu concentration prior to the drying reaction). Glu was analyzed by negative mode SIM-LC-MS and oligomers were identified as [M-H]- ions, [M+formate]- ions, and [M+Cl]- ions (for monomers (A), dimers (B), trimers (C), tetramers (D), pentamers (E), hexamers (F), heptamers (G), octamers (H), and nonamers (I)).

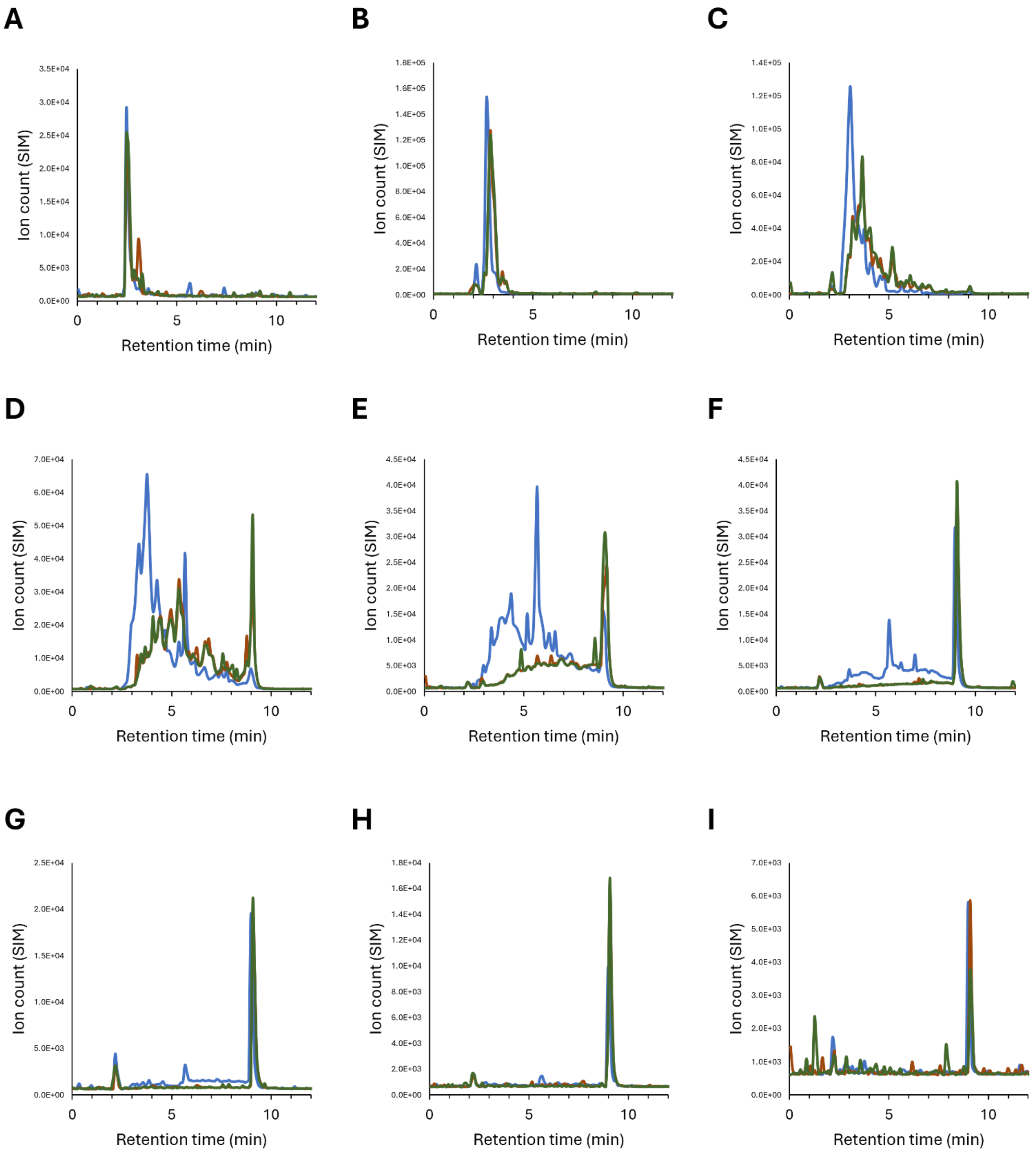

Figure S146. SIM-LC-MS of a dry down reaction of glucose (glu) under acidified conditions in the presence of $MgCl_2$ at a 1mM final concentration (referring to initial glu concentration prior to the drying reaction). Glu was analyzed by negative mode SIM-LC-MS and oligomers were identified as $[M-H]^-$ ions, $[M+formate]^-$ ions, and $[M+Cl]^-$ ions (for monomers (A), dimers (B), trimers (C), tetramers (D), pentamers (E), hexamers (F), heptamers (G), octamers (H), and nonamers (I)).

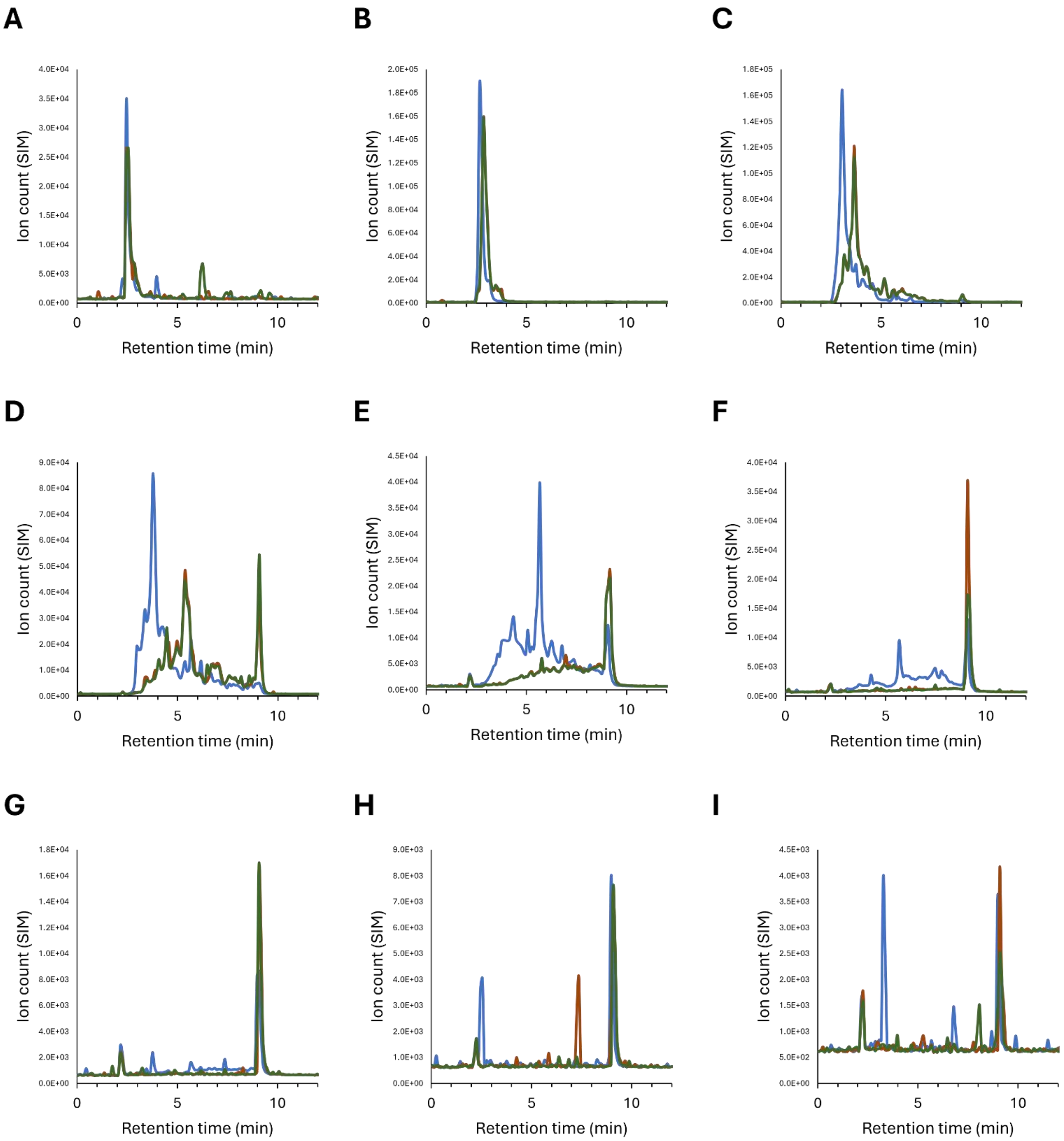

Figure S147. SIM-LC-MS of a dry down reaction of glucose (glu) under acidified conditions in the presence of $CaCl_2$ at a 1mM final concentration (referring to initial glu concentration prior to the drying reaction). Glu was analyzed by negative mode SIM-LC-MS and oligomers were identified as $[M-H]^-$ ions, $[M+formate]^-$ ions, and $[M+Cl]^-$ ions (for monomers (A), dimers (B), trimers (C), tetramers (D), pentamers (E), hexamers (F), heptamers (G), octamers (H), and nonamers (I)).

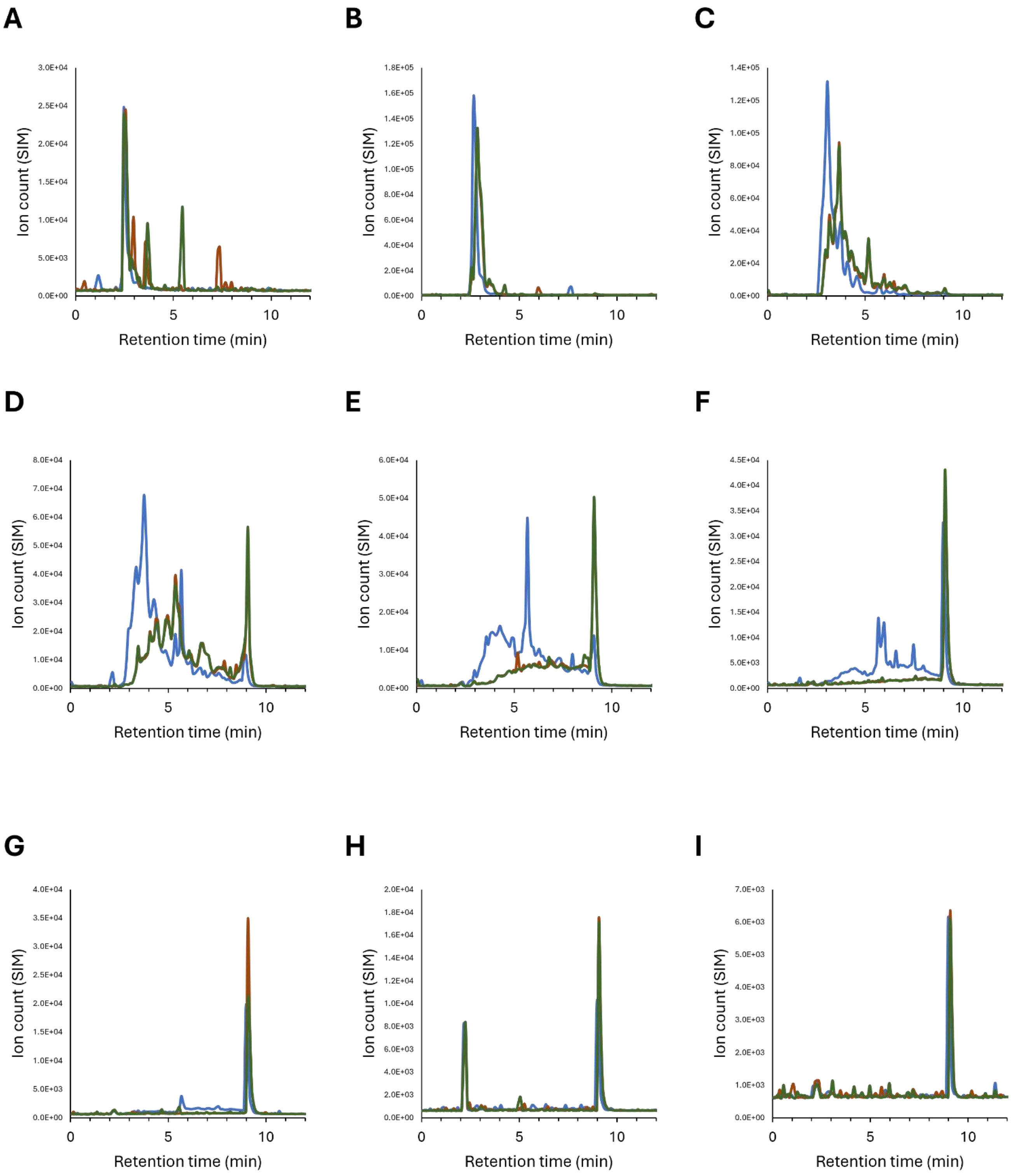

Figure S148. SIM-LC-MS of a dry down reaction of glucose (glu) under acidified conditions in the presence of $MnCl_2$ at a 1mM final concentration (referring to initial glu concentration prior to the drying reaction). Glu was analyzed by negative mode SIM-LC-MS and oligomers were identified as $[M-H]^-$ ions, $[M+formate]^-$ ions, and $[M+Cl]^-$ ions (for monomers (A), dimers (B), trimers (C), tetramers (D), pentamers (E), hexamers (F), heptamers (G), octamers (H), and nonamers (I)).

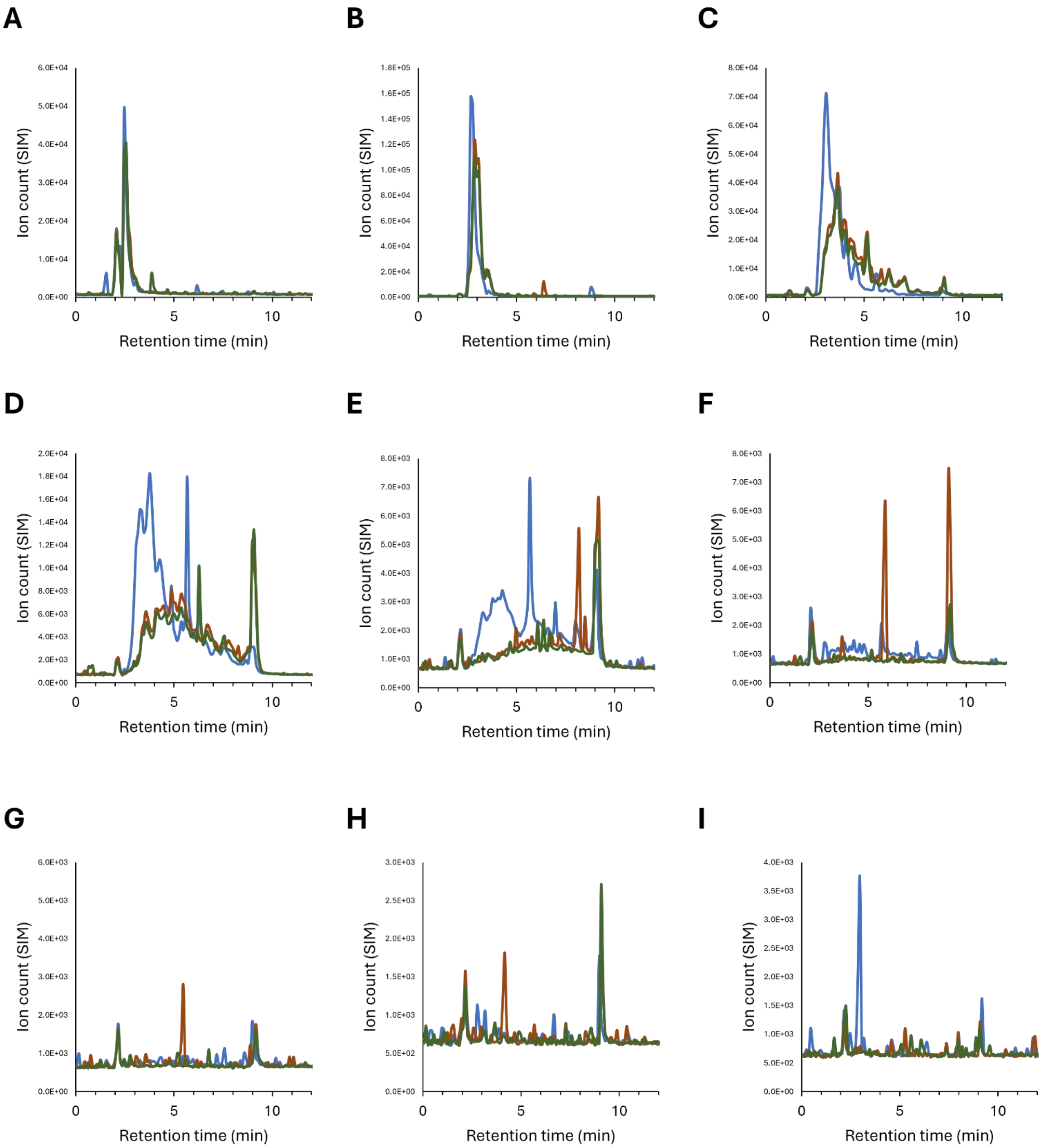

Figure S149. SIM-LC-MS of a dry down reaction of glucose (glu) under acidified conditions in the presence of $AlCl_3$ under anaerobic conditions at a 1mM final concentration (referring to initial glu concentration prior to the drying reaction). Glu was analyzed by negative mode SIM-LC-MS and oligomers were identified as $[M-H]^-$ ions, $[M+formate]^-$ ions, and $[M+Cl]^-$ ions (for monomers (A), dimers (B), trimers (C), tetramers (D), pentamers (E), hexamers (F), heptamers (G), octamers (H), and nonamers (I)).

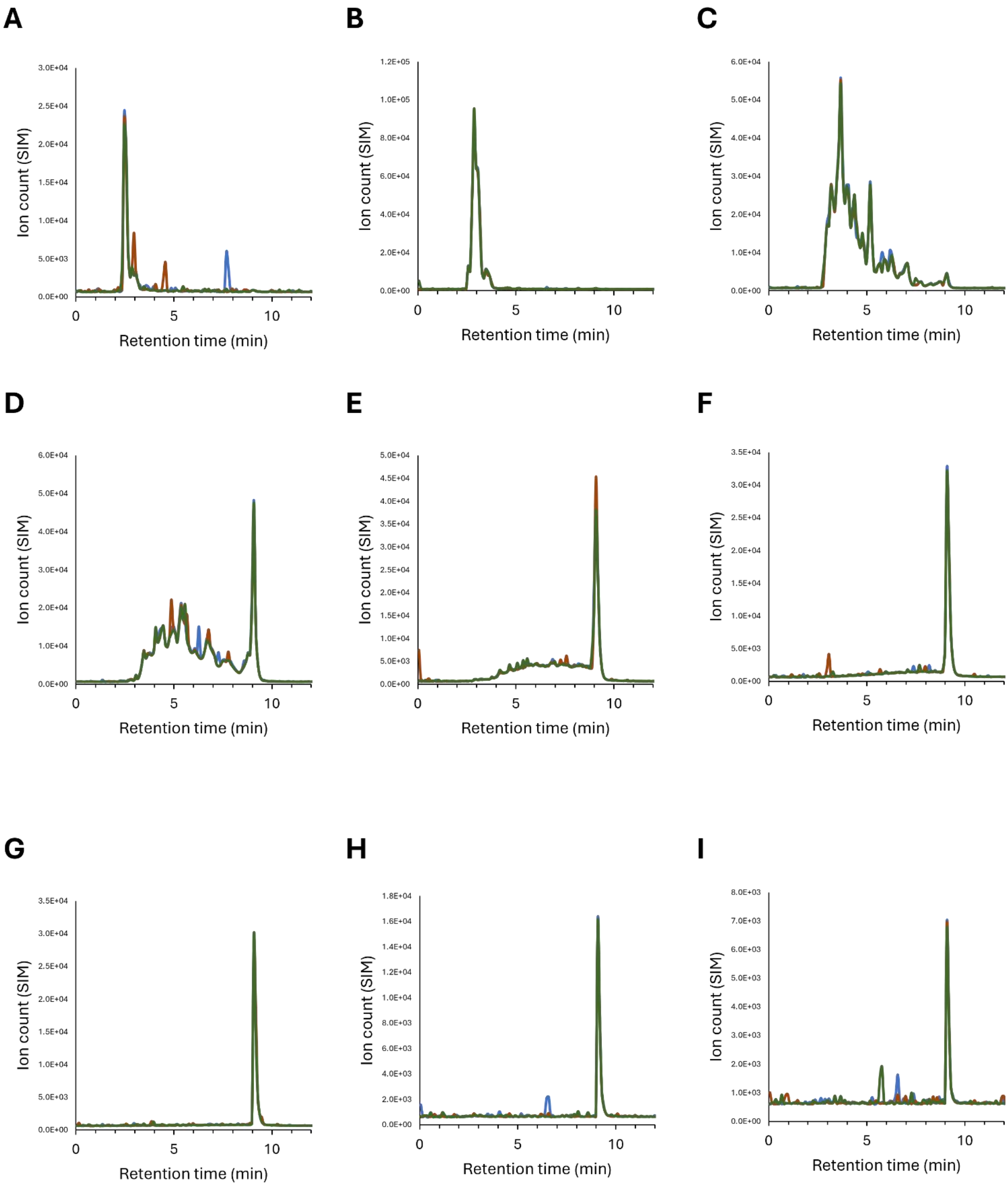

Figure S150. SIM-LC-MS of a dry down reaction of glucose (glu) under acidified conditions in the absence of metals under anaerobic conditions at a 1mM final concentration (referring to initial glu concentration prior to the drying reaction). Glu was analyzed by negative mode SIM-LC-MS and oligomers were identified as [M-H]⁻ ions, [M+formate]⁻ ions, and [M+Cl]⁻ ions (for monomers (A), dimers (B), trimers (C), tetramers (D), pentamers (E), hexamers (F), heptamers (G), octamers (H), and nonamers (I)).

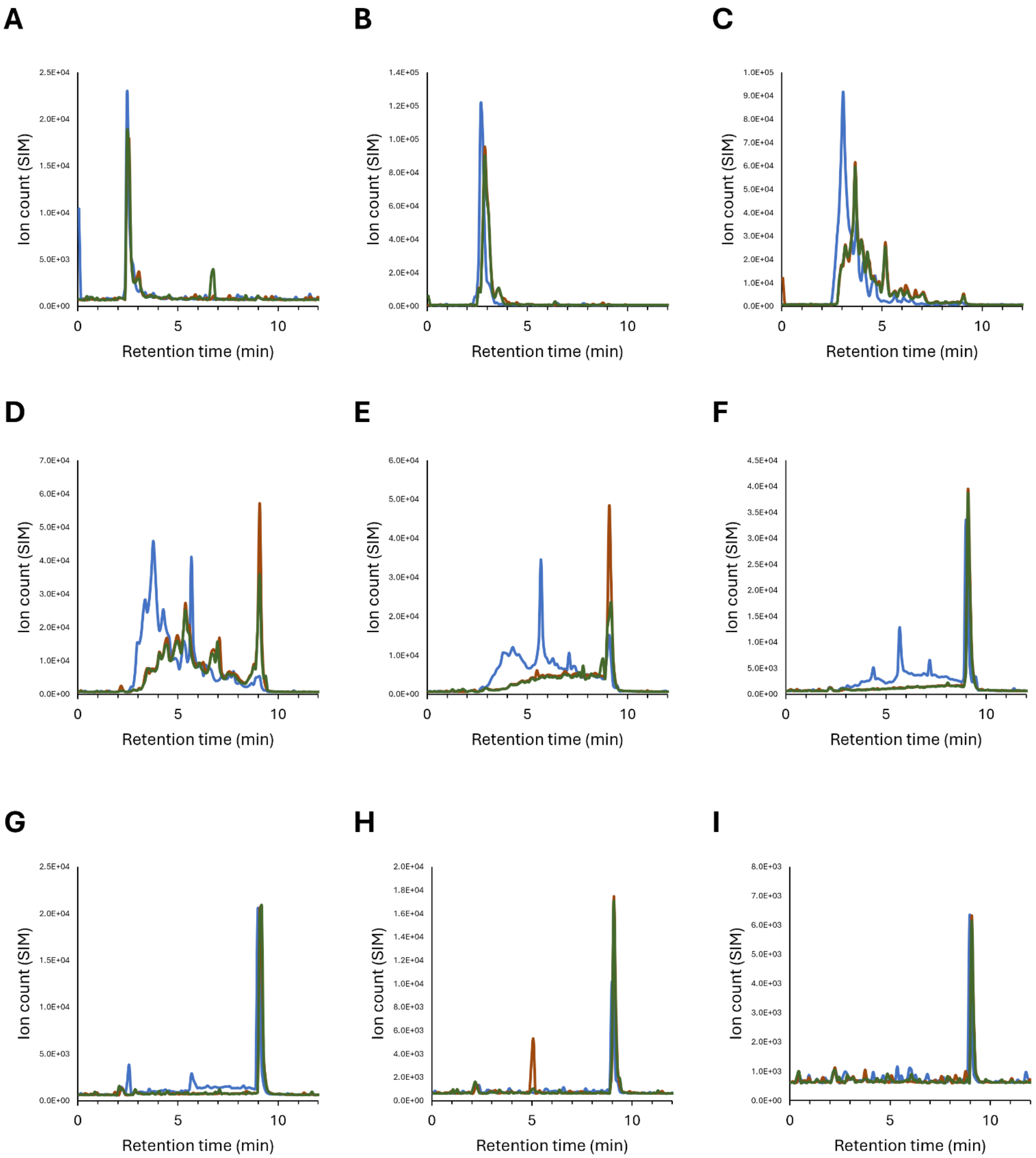

Figure S151. SIM-LC-MS of a dry down reaction of glucose (glu) under acidified conditions in the presence of $FeCl_2$ at a 1mM final concentration (referring to initial glu concentration prior to the drying reaction). Glu was analyzed by negative mode SIM-LC-MS and oligomers were identified as $[M-H]^-$ ions, $[M+formate]^-$ ions, and $[M+Cl]^-$ ions (for monomers (A), dimers (B), trimers (C), tetramers (D), pentamers (E), hexamers (F), heptamers (G), octamers (H), and nonamers (I)).

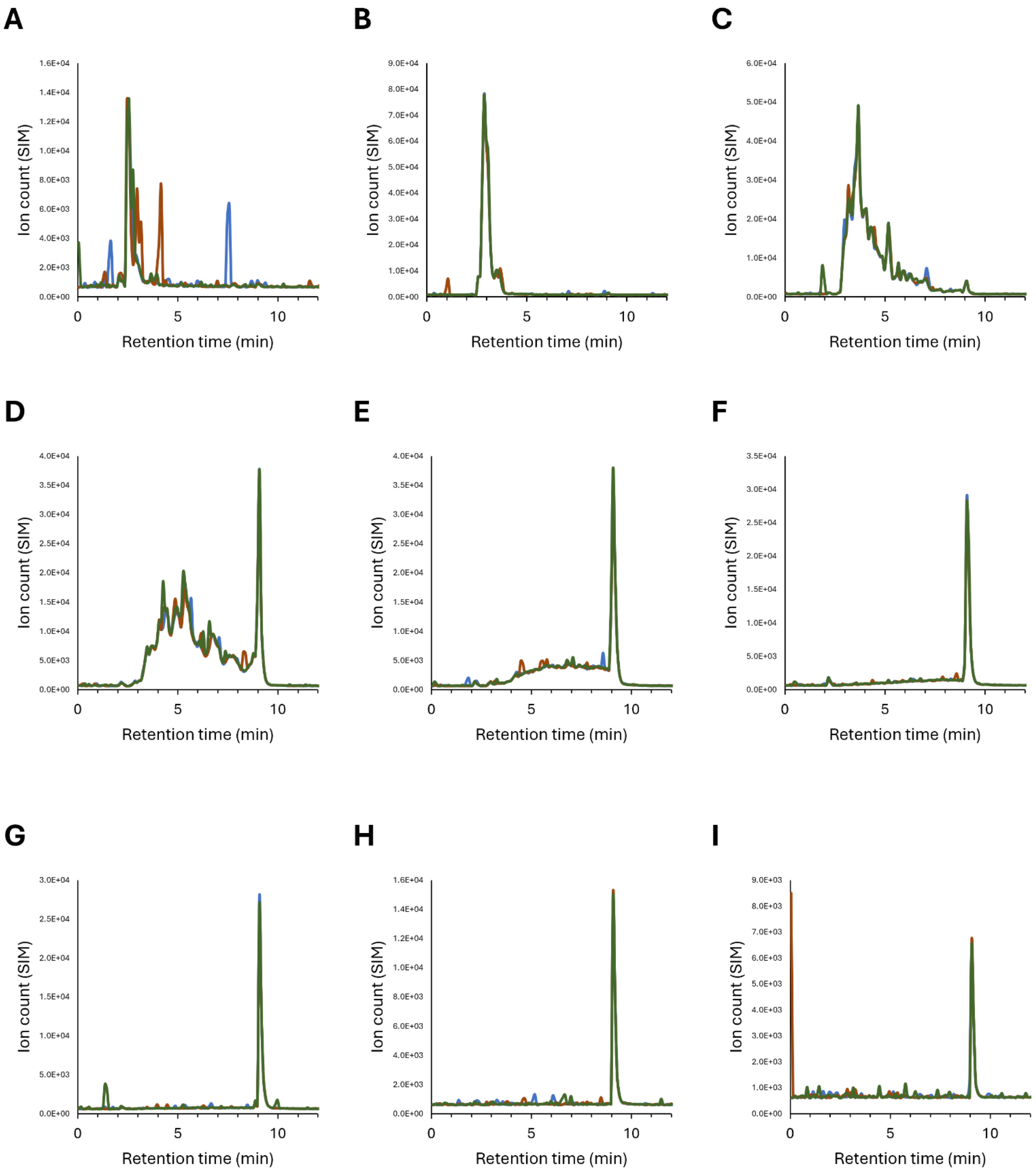

Figure S152. SIM-LC-MS of a dry down reaction of glucose (glu) under acidified conditions in the presence of $ZnCl_2$ under anaerobic conditions at a 1mM final concentration (referring to initial glu concentration prior to the drying reaction). Glu was analyzed by negative mode SIM-LC-MS and oligomers were identified as $[M-H]^-$ ions, $[M+formate]^-$ ions, and $[M+Cl]^-$ ions (for monomers (A), dimers (B), trimers (C), tetramers (D), pentamers (E), hexamers (F), heptamers (G), octamers (H), and nonamers (I)).

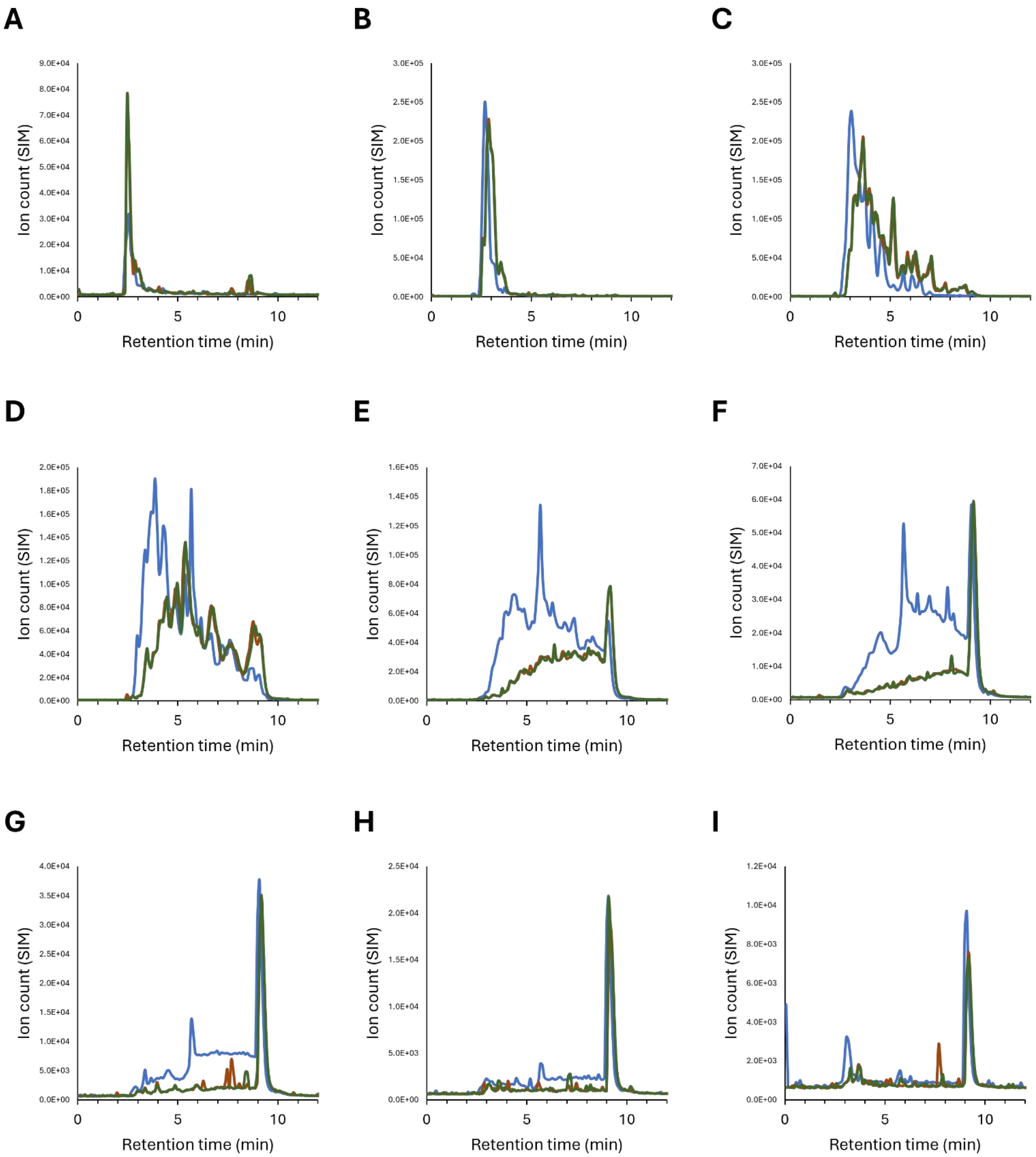

Figure S153. SIM-LC-MS of a dry down reaction of glucose (glu) under acidified conditions in the absence of metals at a 10mM final concentration (referring to initial glu concentration prior to the drying reaction). Glu was analyzed by negative mode SIM-LC-MS and oligomers were identified as [M-H]$^-$ ions, [M+formate]$^-$ ions, and [M+Cl]$^-$ ions (for monomers (A), dimers (B), trimers (C), tetramers (D), pentamers (E), hexamers (F), heptamers (G), octamers (H), and nonamers (I)).

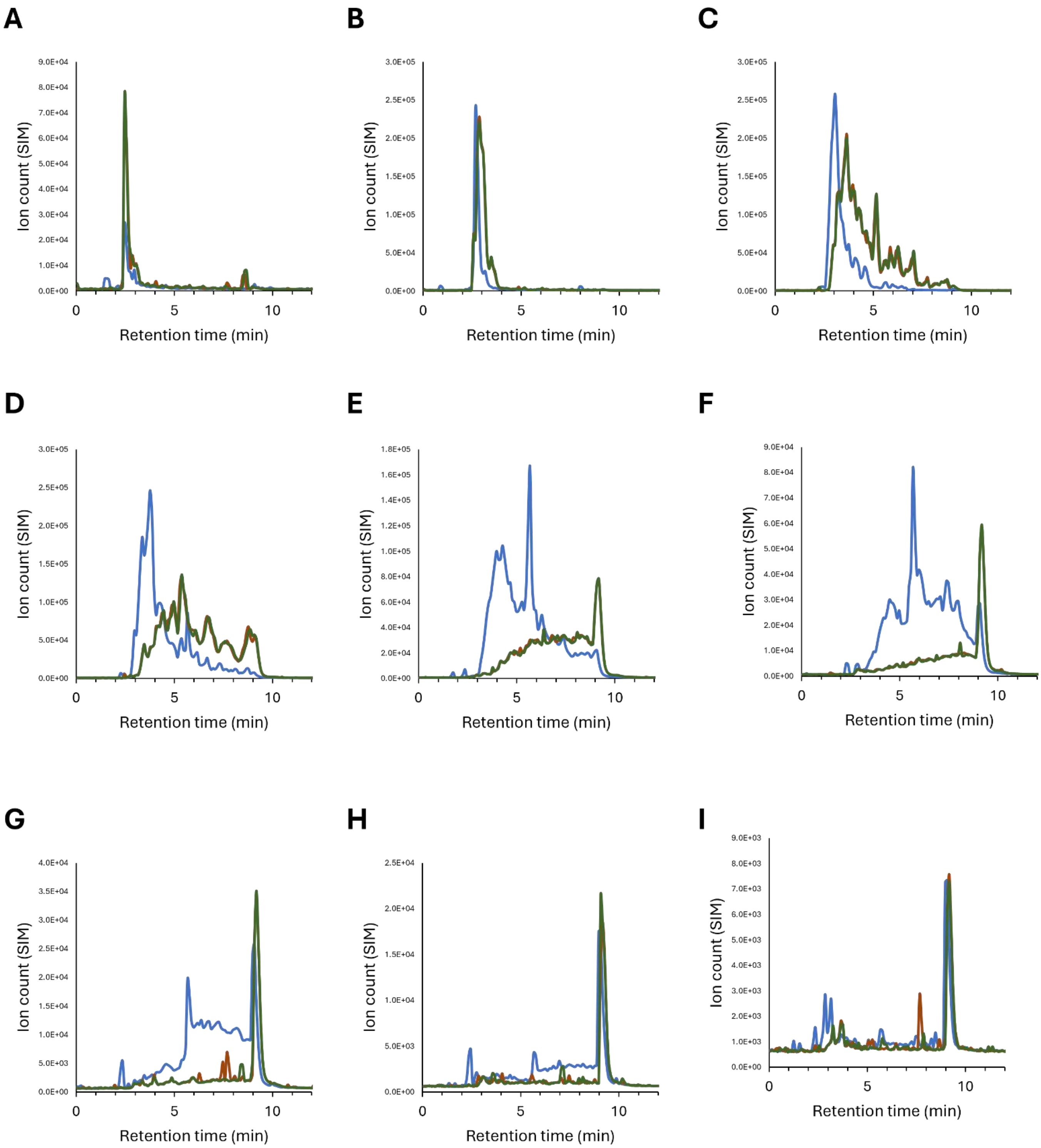

Figure S154. SIM-LC-MS of a dry down reaction of glucose (glu) under acidified conditions in the presence of LiCl at a 10mM final concentration (referring to initial glu concentration prior to the drying reaction). Glu was analyzed by negative mode SIM-LC-MS and oligomers were identified as [M-H]$^-$ ions, [M+formate]$^-$ ions, and [M+Cl]$^-$ ions (for monomers (A), dimers (B), trimers (C), tetramers (D), pentamers (E), hexamers (F), heptamers (G), octamers (H), and nonamers (I)).

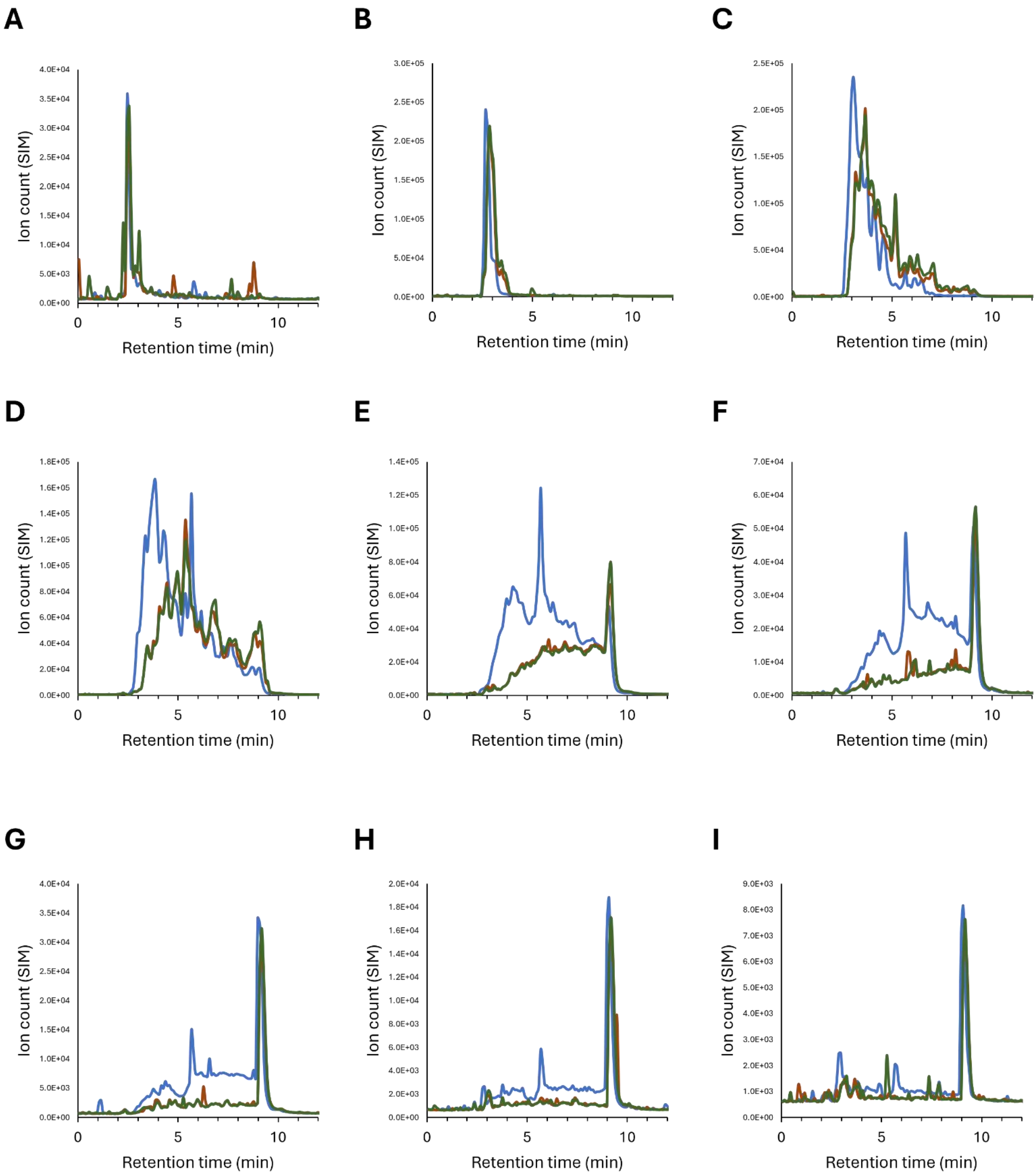

Figure S155. SIM-LC-MS of a dry down reaction of glucose (glu) under acidified conditions in the presence of $ZnCl_2$ at a 10mM final concentration (referring to initial glu concentration prior to the drying reaction). Glu was analyzed by negative mode SIM-LC-MS and oligomers were identified as $[M-H]^-$ ions, $[M+formate]^-$ ions, and $[M+Cl]^-$ ions (for monomers (A), dimers (B), trimers (C), tetramers (D), pentamers (E), hexamers (F), heptamers (G), octamers (H), and nonamers (I)).

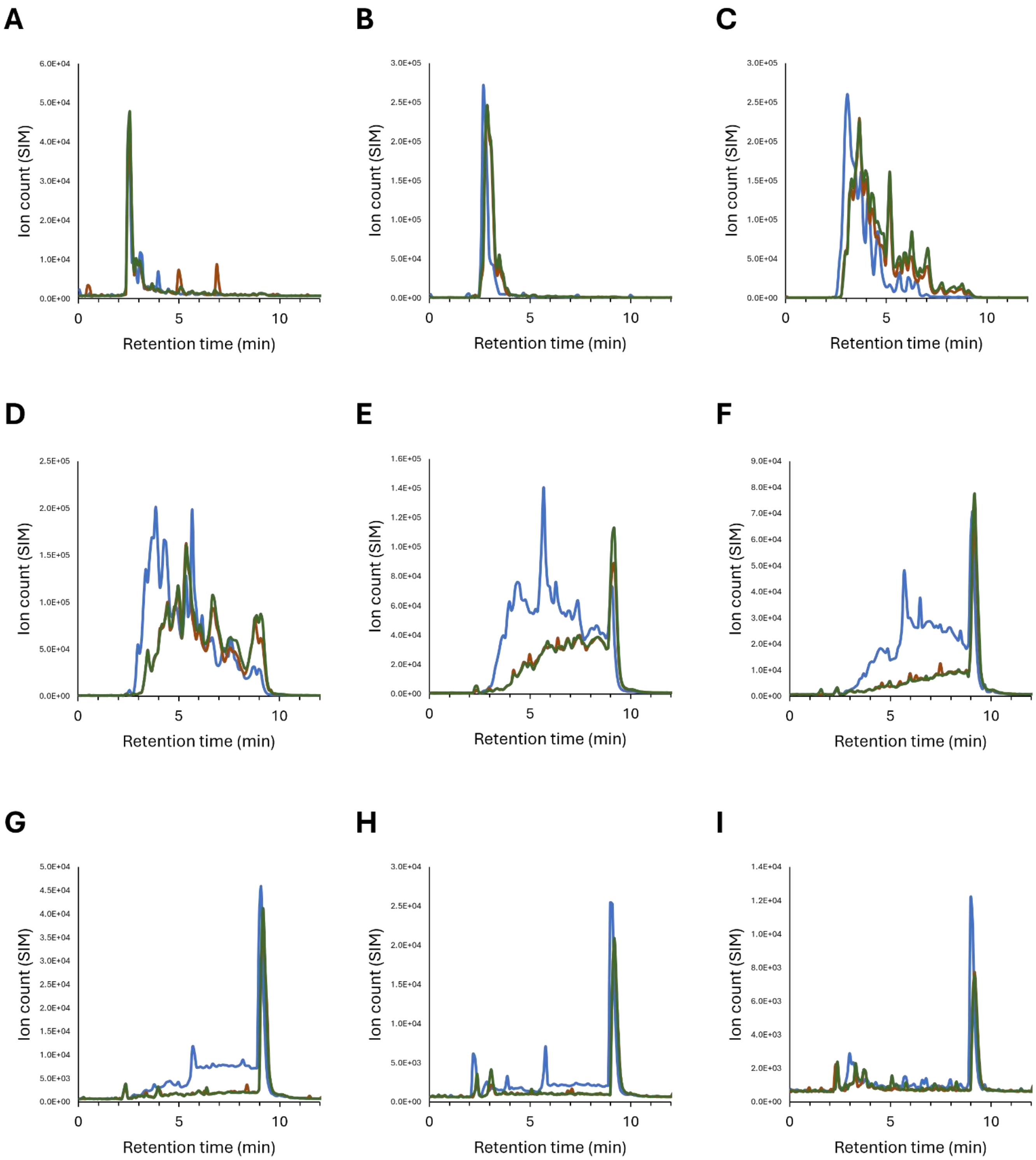

Figure S156. SIM-LC-MS of a dry down reaction of glucose (glu) under acidified conditions in the presence of NaCl at a 10mM final concentration (referring to initial glu concentration prior to the drying reaction). Glu was analyzed by negative mode SIM-LC-MS and oligomers were identified as [M-H]$^-$ ions, [M+formate]$^-$ ions, and [M+Cl]$^-$ ions (for monomers (A), dimers (B), trimers (C), tetramers (D), pentamers (E), hexamers (F), heptamers (G), octamers (H), and nonamers (I)

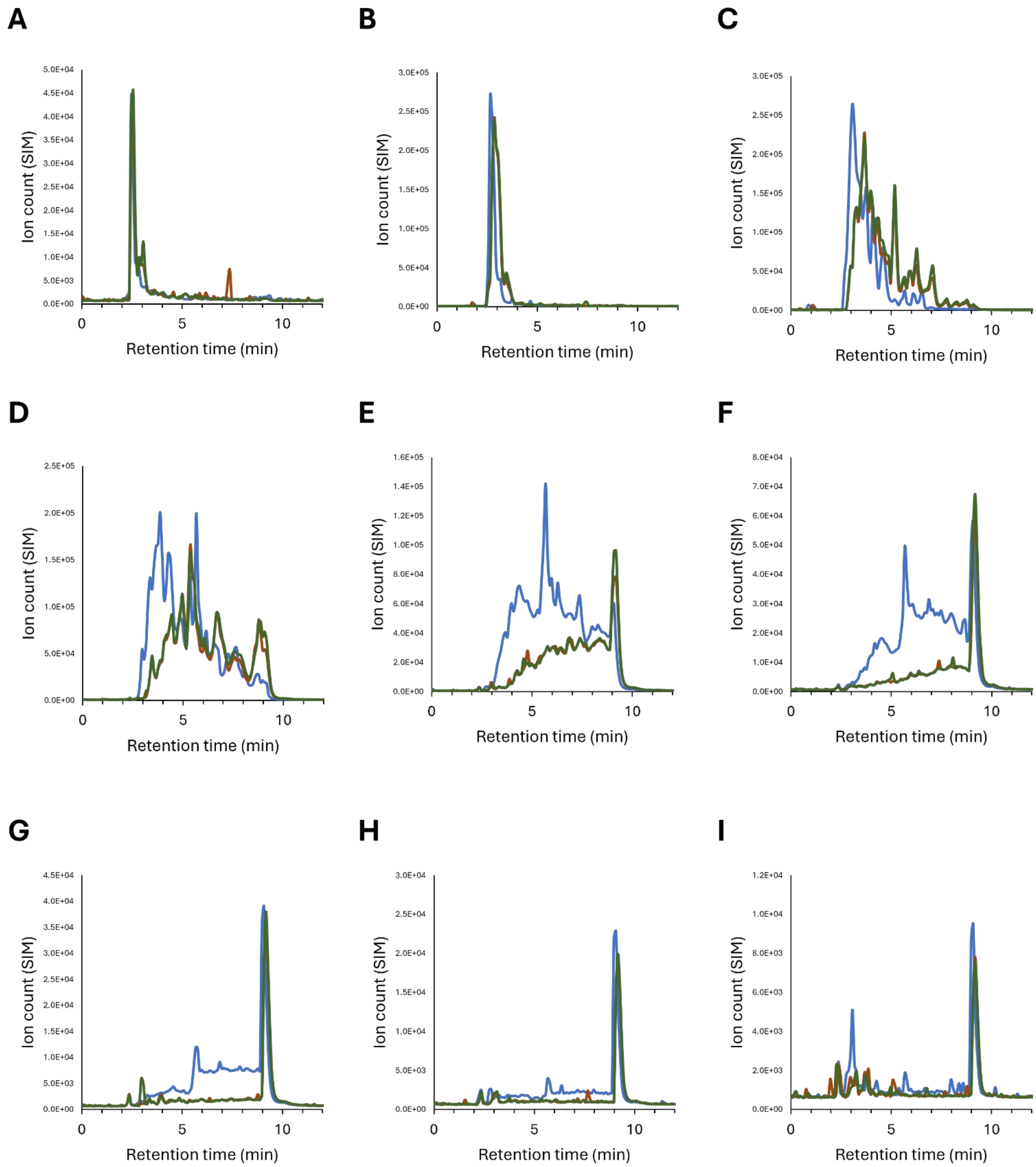

Figure S157. SIM-LC-MS of a dry down reaction of glucose (glu) under acidified conditions in the presence of KCl at a 10mM final concentration (referring to initial glu concentration prior to the drying reaction). Glu was analyzed by negative mode SIM-LC-MS and oligomers were identified as $[M-H]^-$ ions, $[M+formate]^-$ ions, and $[M+Cl]^-$ ions (for monomers (A), dimers (B), trimers (C), tetramers (D), pentamers (E), hexamers (F), heptamers (G), octamers (H), and nonamers (I)).

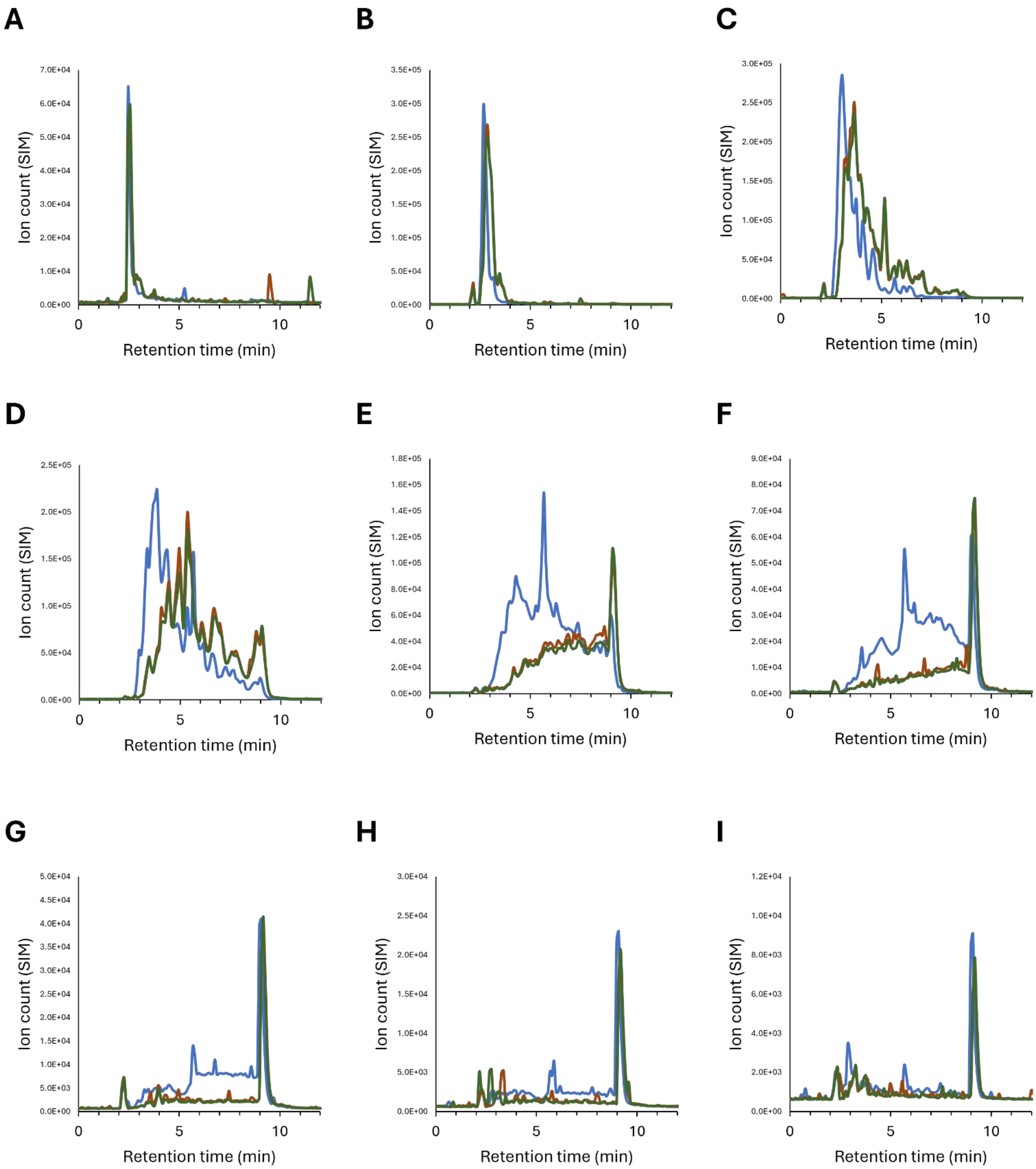

Figure S158. SIM-LC-MS of a dry down reaction of glucose (glu) under acidified conditions in the presence of $MgCl_2$ at a 10mM final concentration (referring to initial glu concentration prior to the drying reaction). Glu was analyzed by negative mode SIM-LC-MS and oligomers were identified as $[M-H]^-$ ions, $[M+formate]^-$ ions, and $[M+Cl]^-$ ions (for monomers(A), dimers (B), trimers (C), tetramers (D), pentamers (E), hexamers (F), heptamers (G), octamers (H), and nonamers (I)).

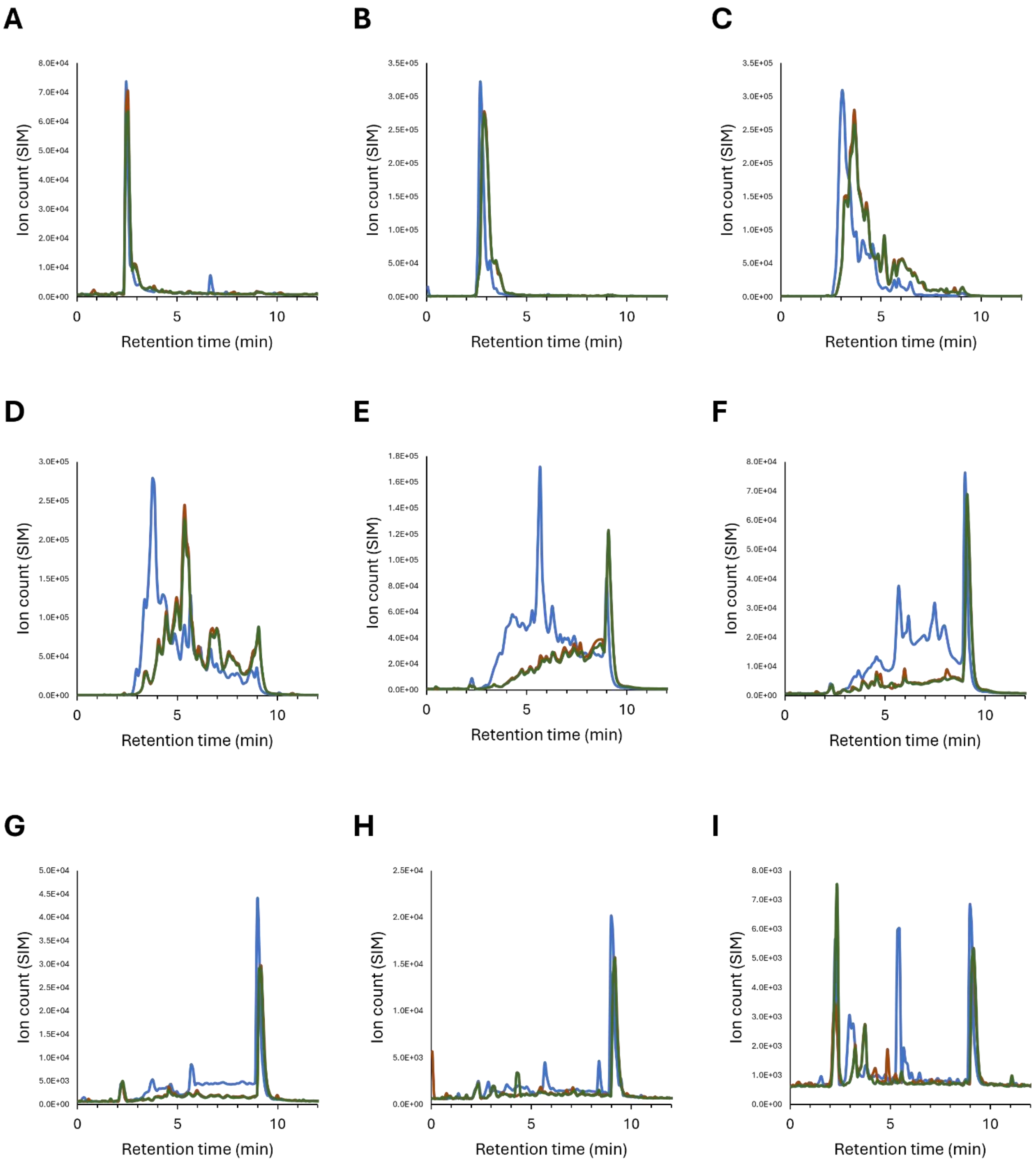

Figure S159. SIM-LC-MS of a dry down reaction of glucose (glu) under acidified conditions in the presence of $CaCl_2$ at a 10mM final concentration (referring to initial glu concentration prior to the drying reaction). Glu was analyzed by negative mode SIM-LC-MS and oligomers were identified as $[M-H]^-$ ions, $[M+formate]^-$ ions, and $[M+Cl]^-$ ions (for monomers (A), dimers (B), trimers (C), tetramers (D), pentamers (E), hexamers (F), heptamers (G), octamers (H), and nonamers (I)).

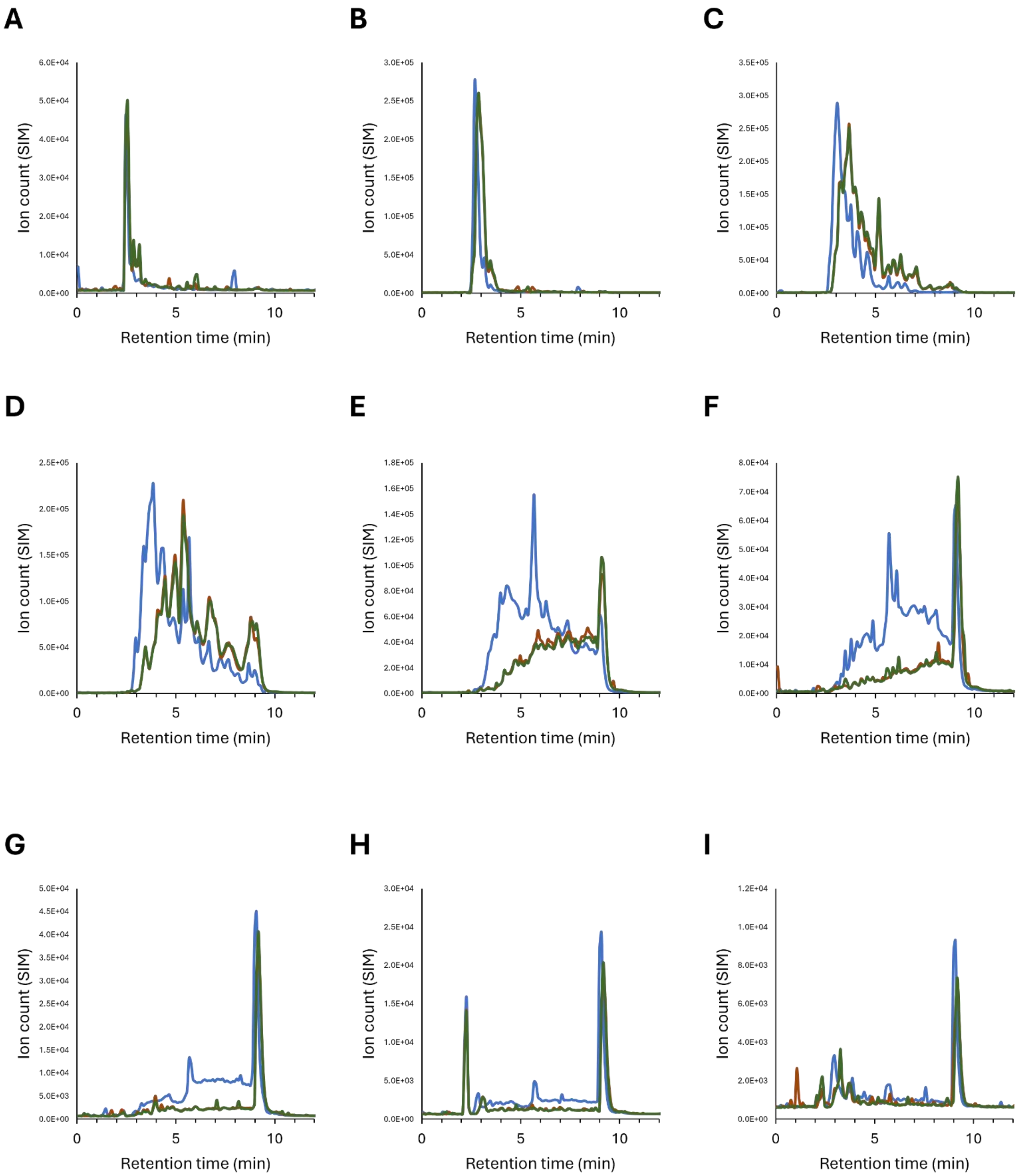

Figure S160. SIM-LC-MS of a dry down reaction of glucose (glu) under acidified conditions in the presence of $MnCl_2$ at a 10mM final concentration (referring to initial glu concentration prior to the drying reaction). Glu was analyzed by negative mode SIM-LC-MS and oligomers were identified as $[M-H]^-$ ions, $[M+formate]^-$ ions, and $[M+Cl]^-$ ions (for monomers (A), dimers (B), trimers (C), tetramers (D), pentamers (E), hexamers (F), heptamers (G), octamers (H), and nonamers (I)).

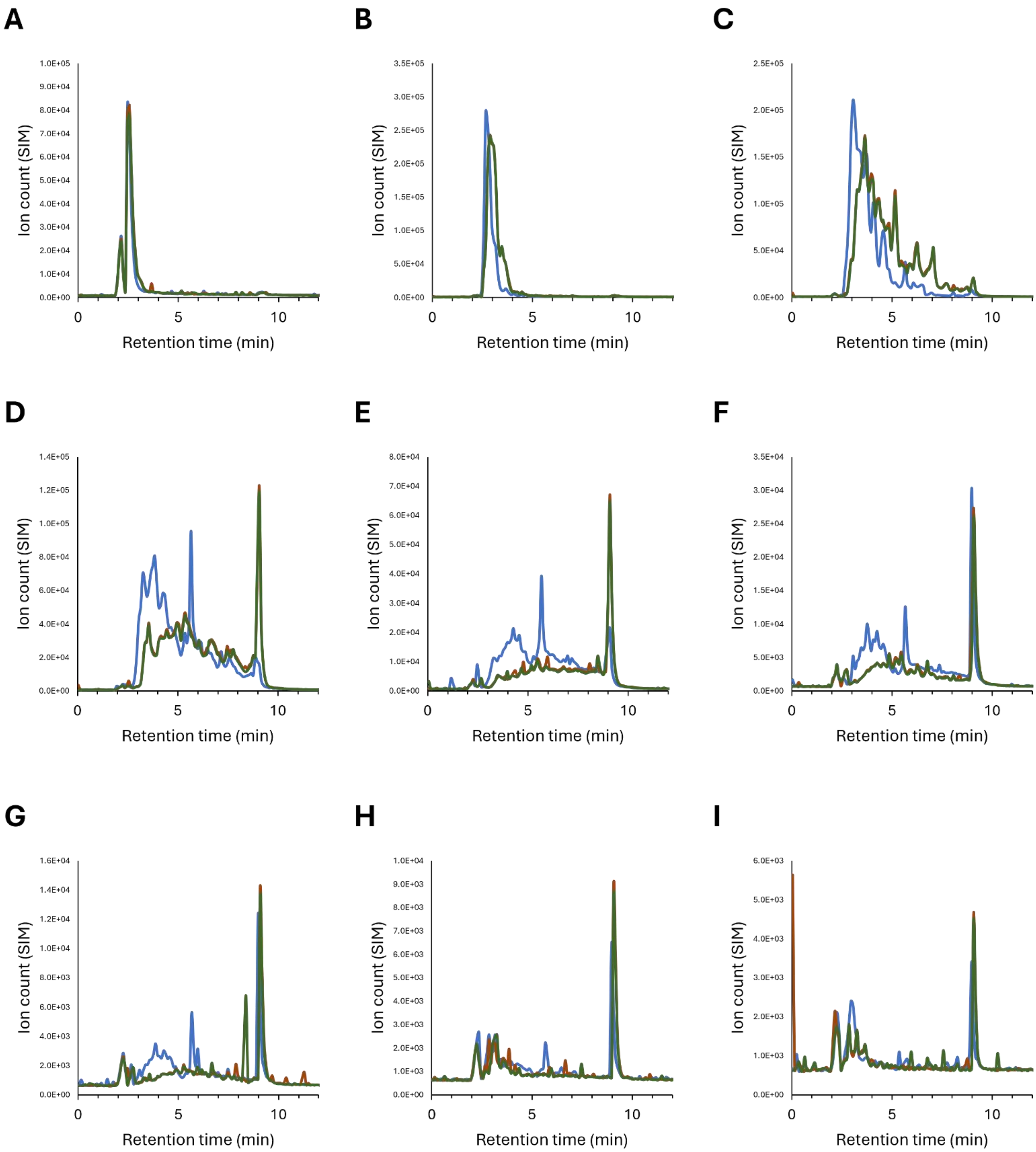

Figure S161. SIM-LC-MS of a dry down reaction of glucose (glu) under acidified conditions in the presence of $AlCl_3$ at a 10mM final concentration (referring to initial glu concentration prior to the drying reaction). Glu was analyzed by negative mode SIM-LC-MS and oligomers were identified as $[M-H]^-$ ions, $[M+formate]^-$ ions, and $[M+Cl]^-$ ions (for monomers (A), dimers (B), trimers (C), tetramers (D), pentamers (E), hexamers (F), heptamers (G), octamers (H), and nonamers (I)).

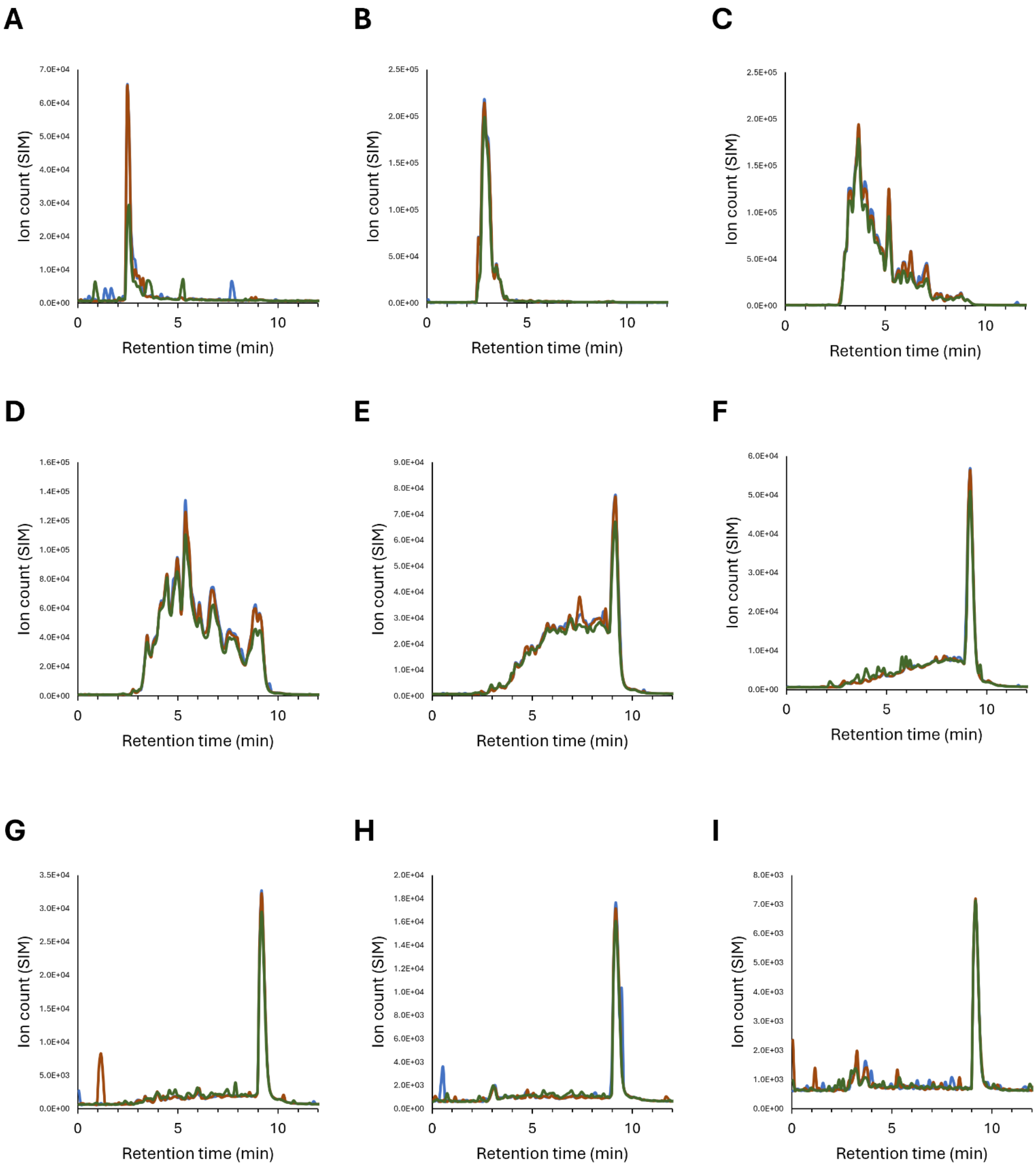

Figure S162. SIM-LC-MS of a dry down reaction of glucose (glu) under acidified conditions in the absence of metals under anaerobic conditions at a 10mM final concentration (referring to initial glu concentration prior to the drying reaction). Glu was analyzed by negative mode SIM-LC-MS and oligomers were identified as [M-H]$^-$ ions, [M+formate]$^-$ ions, and [M+Cl]$^-$ ions (for monomers (A), dimers (B), trimers (C), tetramers (D), pentamers (E), hexamers (F), heptamers (G), octamers (H), and nonamers (I)).

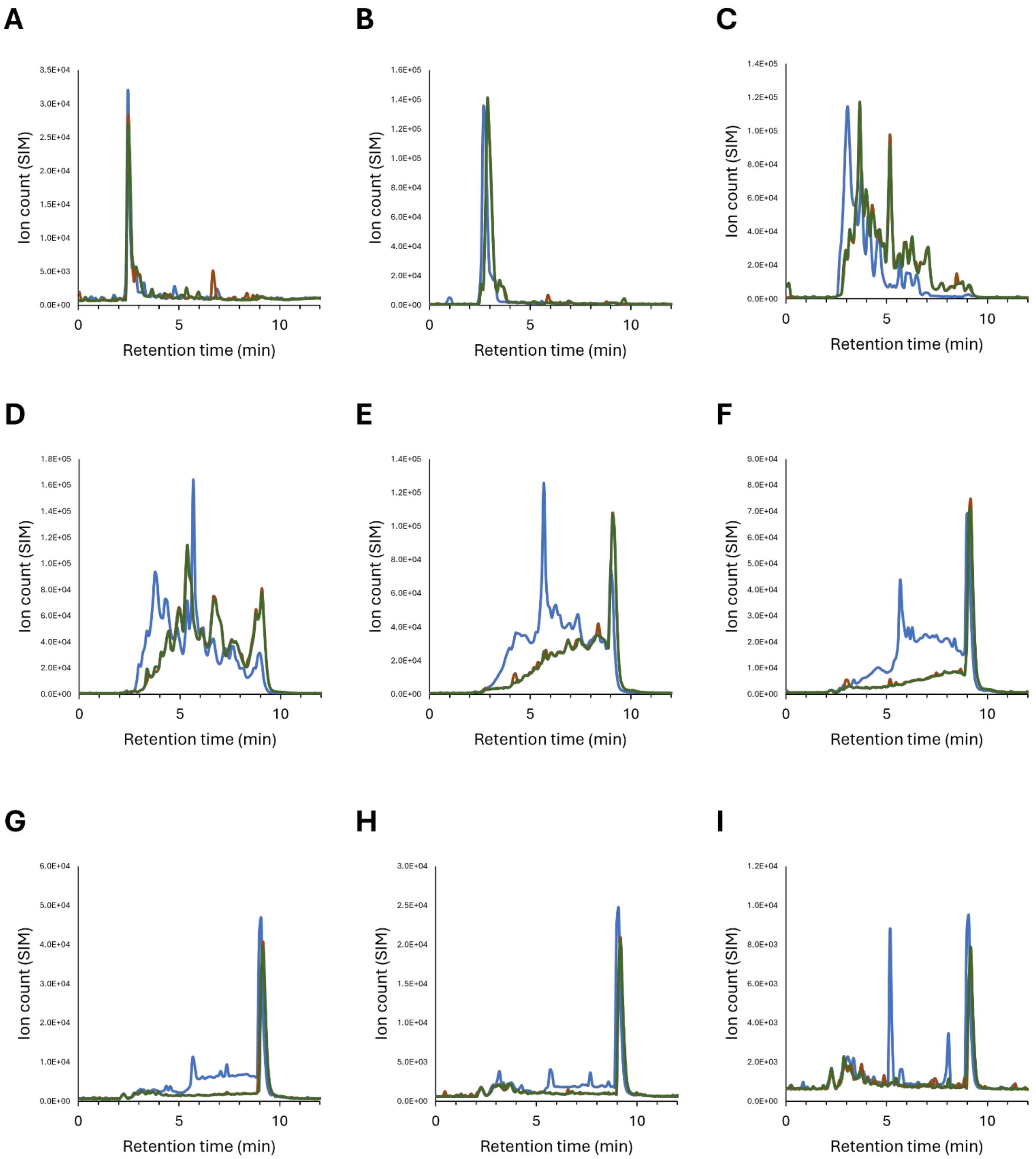

Figure S163. SIM-LC-MS of a dry down reaction of glucose (glu) under acidified conditions in the presence of $FeCl_2$ under anaerobic conditions at a 10mM final concentration (referring to initial glu concentration prior to the drying reaction). Glu was analyzed by negative mode SIM-LC-MS and oligomers were identified as $[M-H]^-$ ions, $[M+formate]^-$ ions, and $[M+Cl]^-$ ions (for monomers (A), dimers (B), trimers (C), tetramers (D), pentamers (E), hexamers (F), heptamers (G), octamers (H), and nonamers (I)).

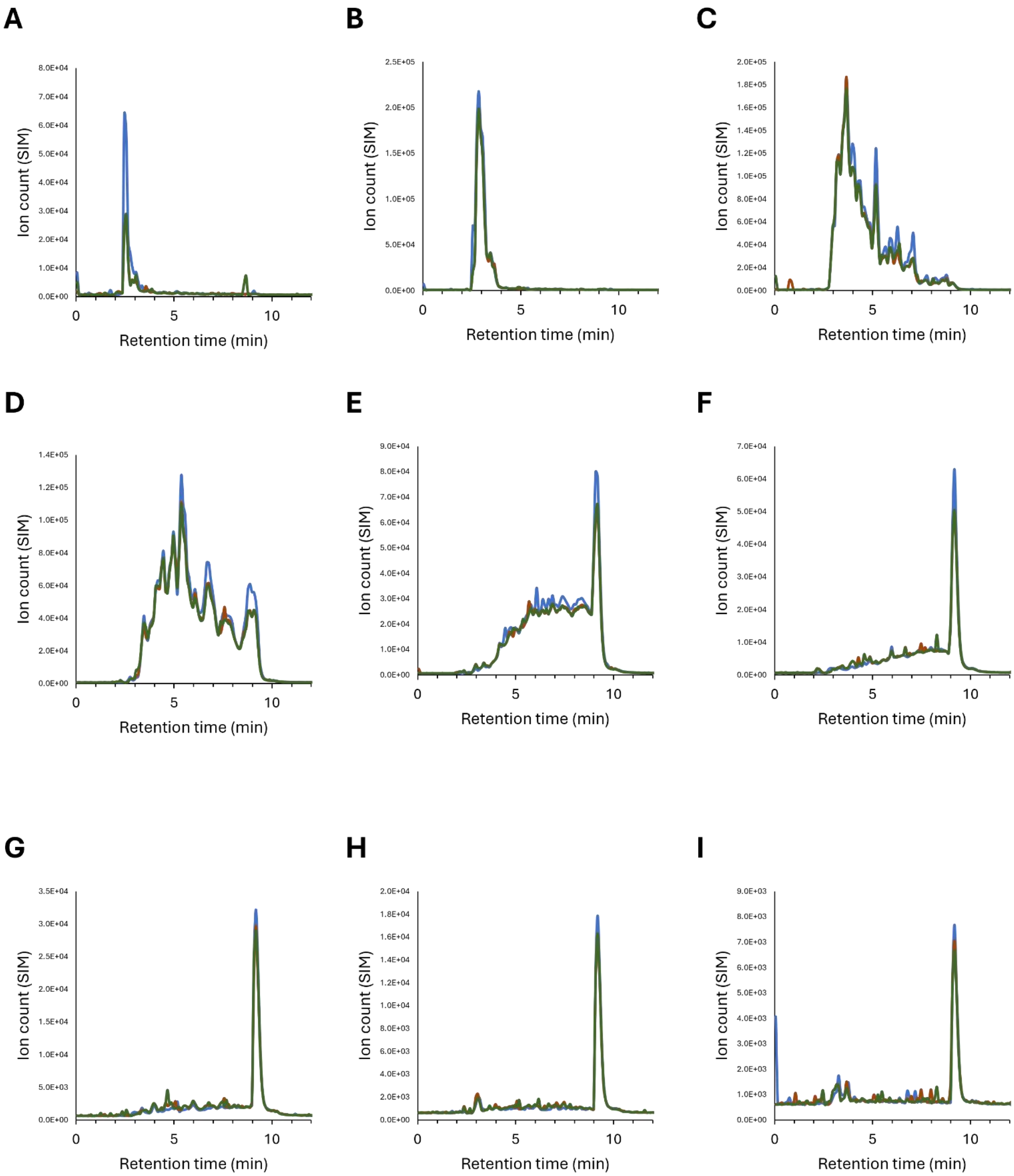

Figure S164. SIM-LC-MS of a dry down reaction of glucose (glu) under acidified conditions in the presence of $ZnCl_2$ under anaerobic conditions at a 10mM final concentration (referring to initial glu concentration prior to the drying reaction). Glu was analyzed by negative mode SIM-LC-MS and oligomers were identified as $[M-H]^-$ ions, $[M+formate]^-$ ions, and $[M+Cl]^-$ ions (for monomers (A), dimers (B), trimers (C), tetramers (D), pentamers (E), hexamers (F), heptamers (G), octamers (H), and nonamers (I)).

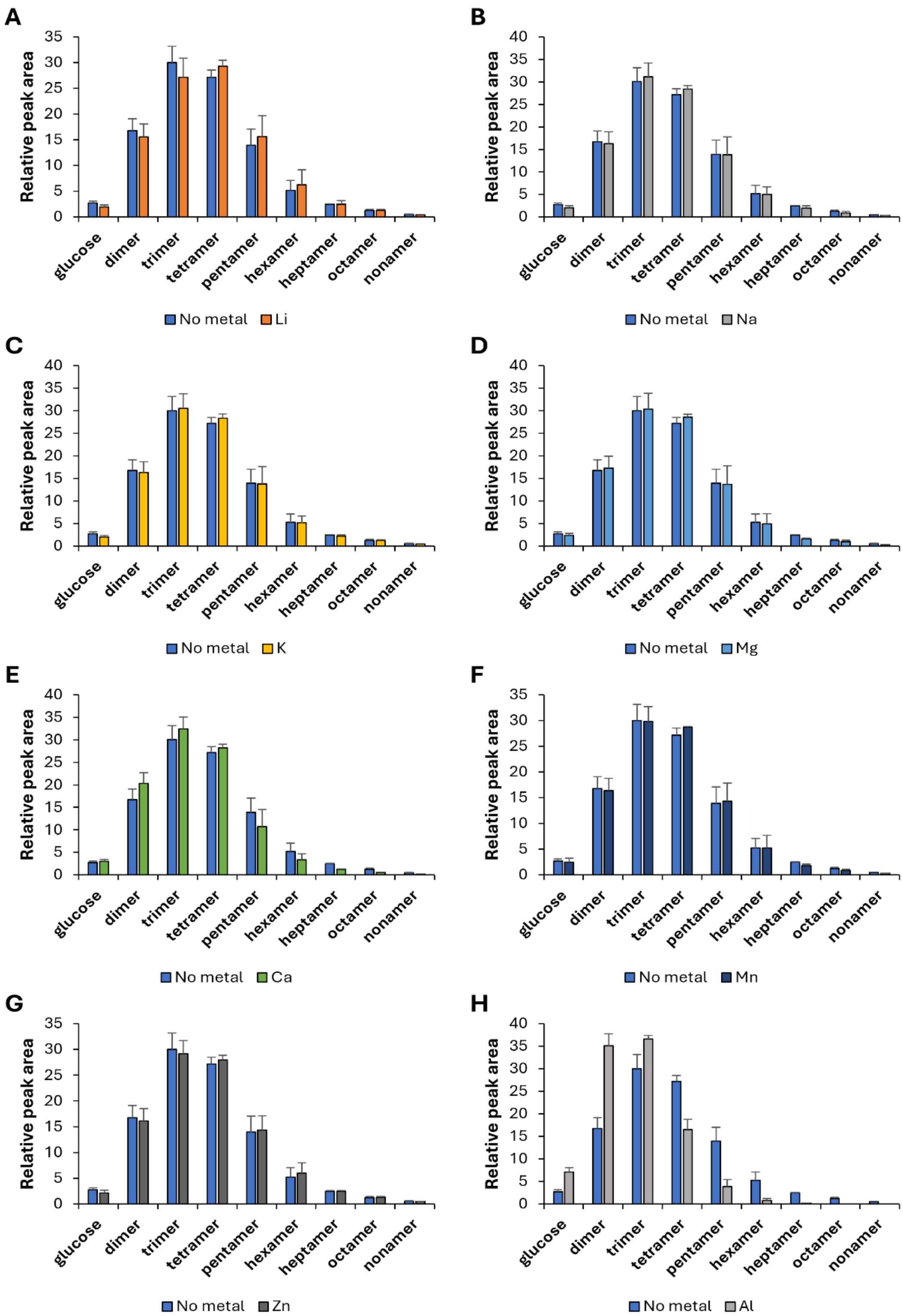

Figure S165. Relative distribution of D-Glucose oligomerization products based on SIM-LC-MS at 1 mM concentration (referring to glucose concentration prior to the drying reaction). Glucose product distribution after one week of dry down at 50 °C in the absence or presence of Li^{+} (A), Na^{+} (B), K^{+} (C), Mg^{2+} (D), Ca^{2+} (E), Mn^{2+} (F), Zn^{2+} (G), Al^{3+} (H) under acidified conditions.

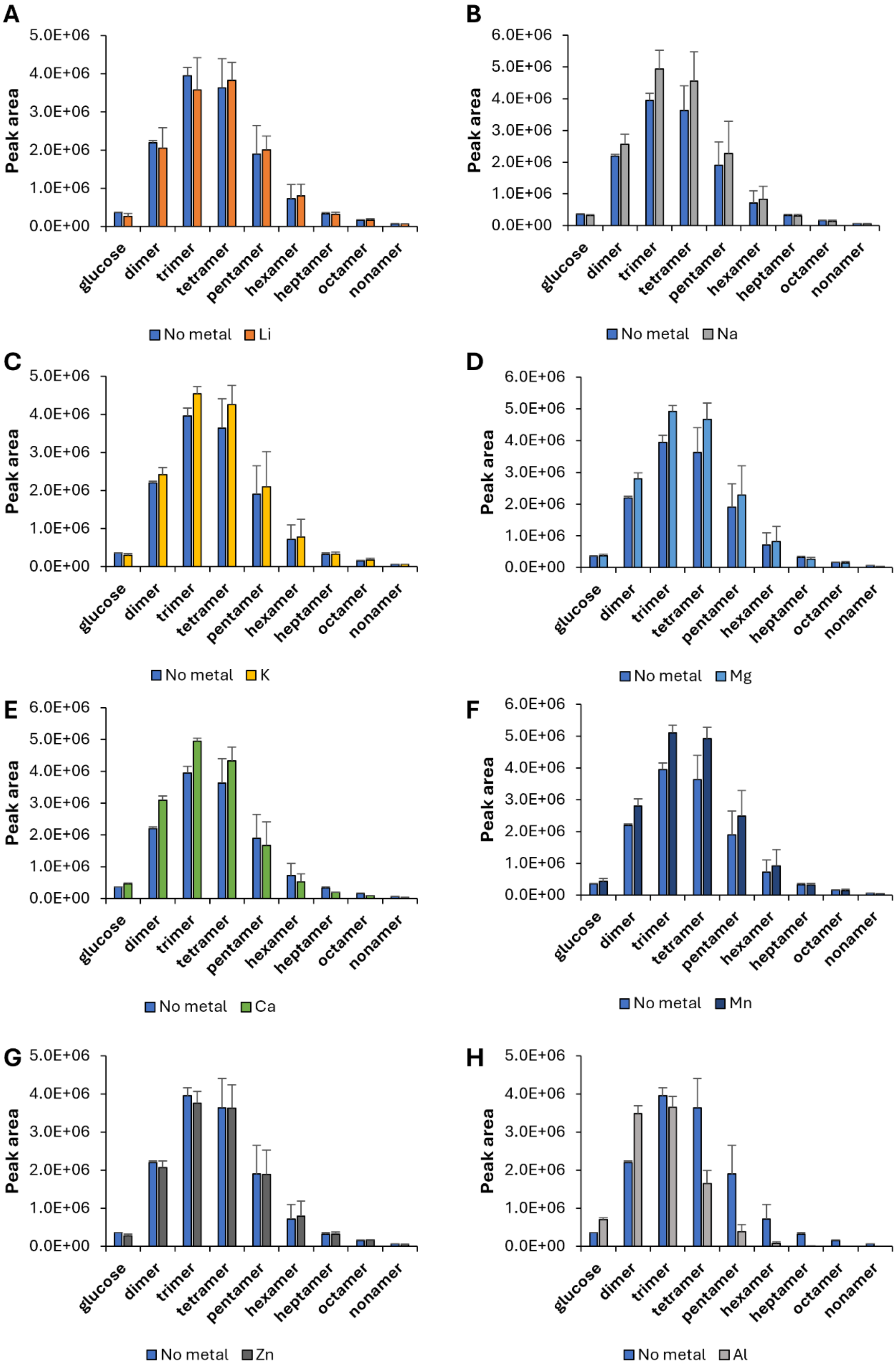

Figure S166. Absolute distribution of D-Glucose oligomerization products based on SIM-LC-MS at 1 mM concentration (referring to glucose concentration prior to the drying reaction). Glucose product distribution after one week of dry down at 50 °C in the absence or presence of Li^+ (A), Na^+ (B), K^+ (C), Mg^{2+} (D), Ca^{2+} (E), Mn^{2+} (F), Zn^{2+} (G), Al^{3+} (H) under acidified conditions.

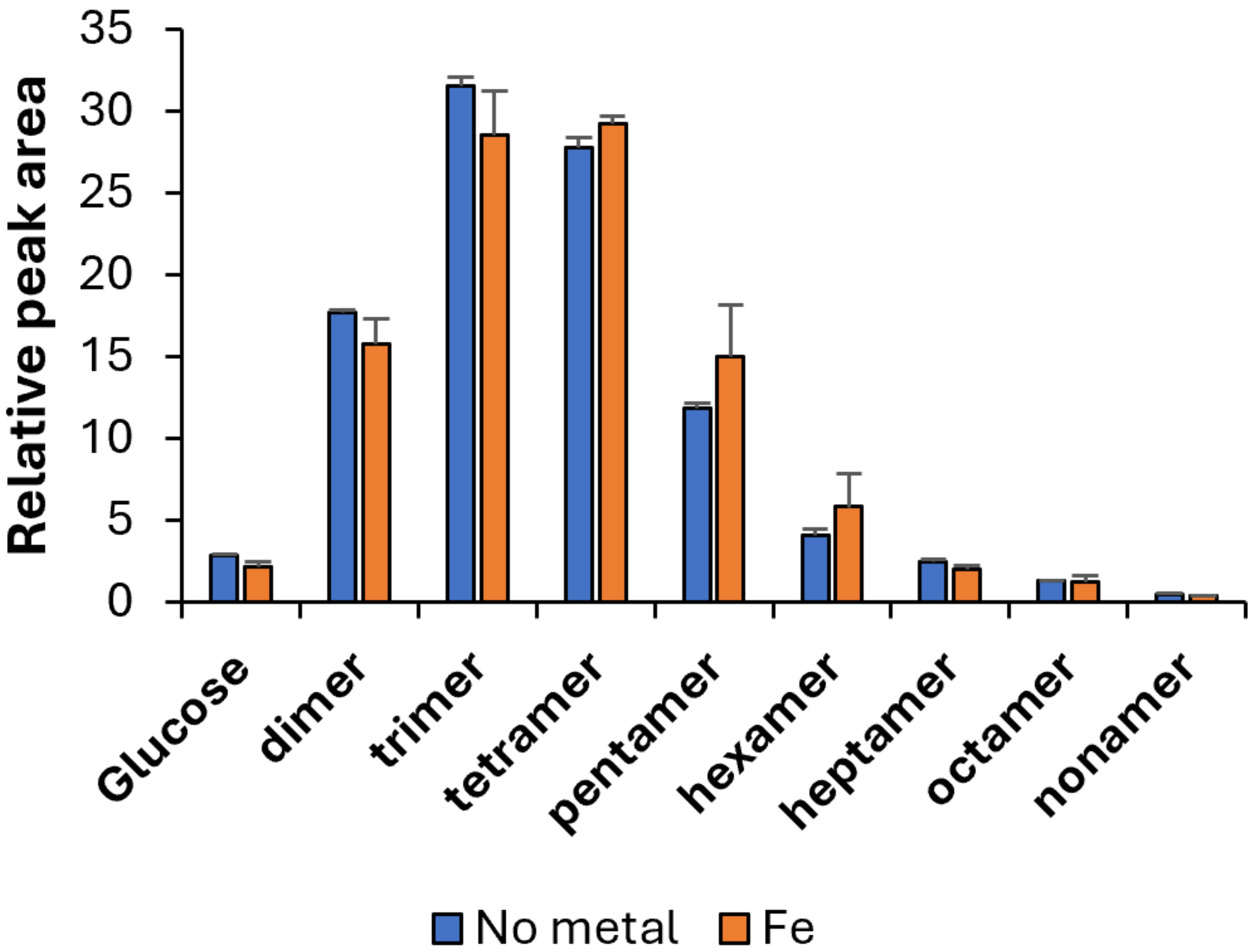

Figure S167. Relative distribution of D-Glucose oligomerization products based on SIM-LC-MS under anaerobic conditions SIM at 1 mM concentration (referring to glucose concentration prior to the drying reaction). Glucose product distribution after one week of dry down at 50 °C in the absence or presence of Fe^{2+} under acidified conditions.

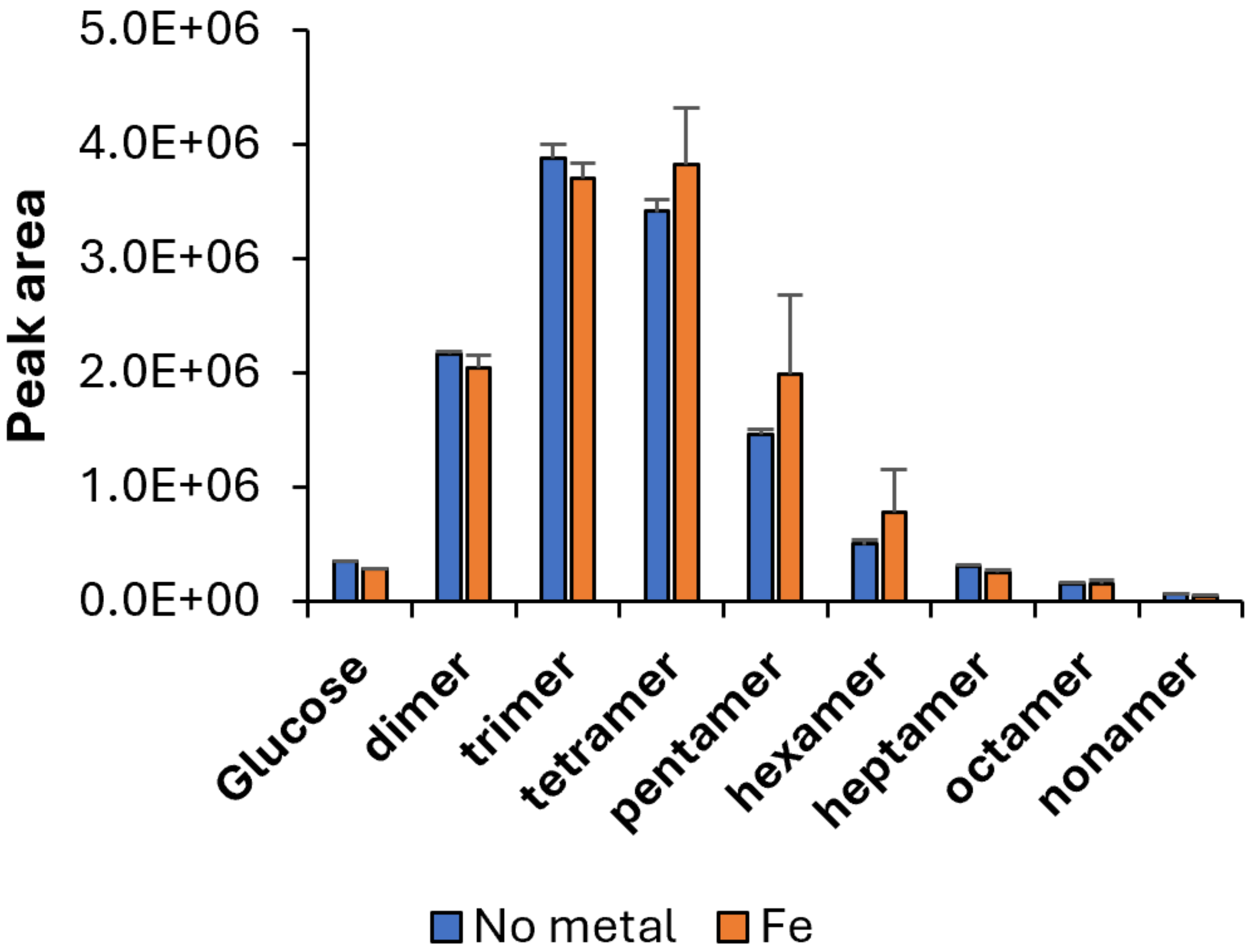

Figure S168. Absolute distribution of D-Glucose oligomerization products based on SIM-LC-MS under anaerobic conditions SIM at 1 mM concentration (referring to glucose concentration prior to the drying reaction). Glucose product distribution after one week of dry down at 50 °C in the absence or presence of Fe^{2+} under acidified conditions.

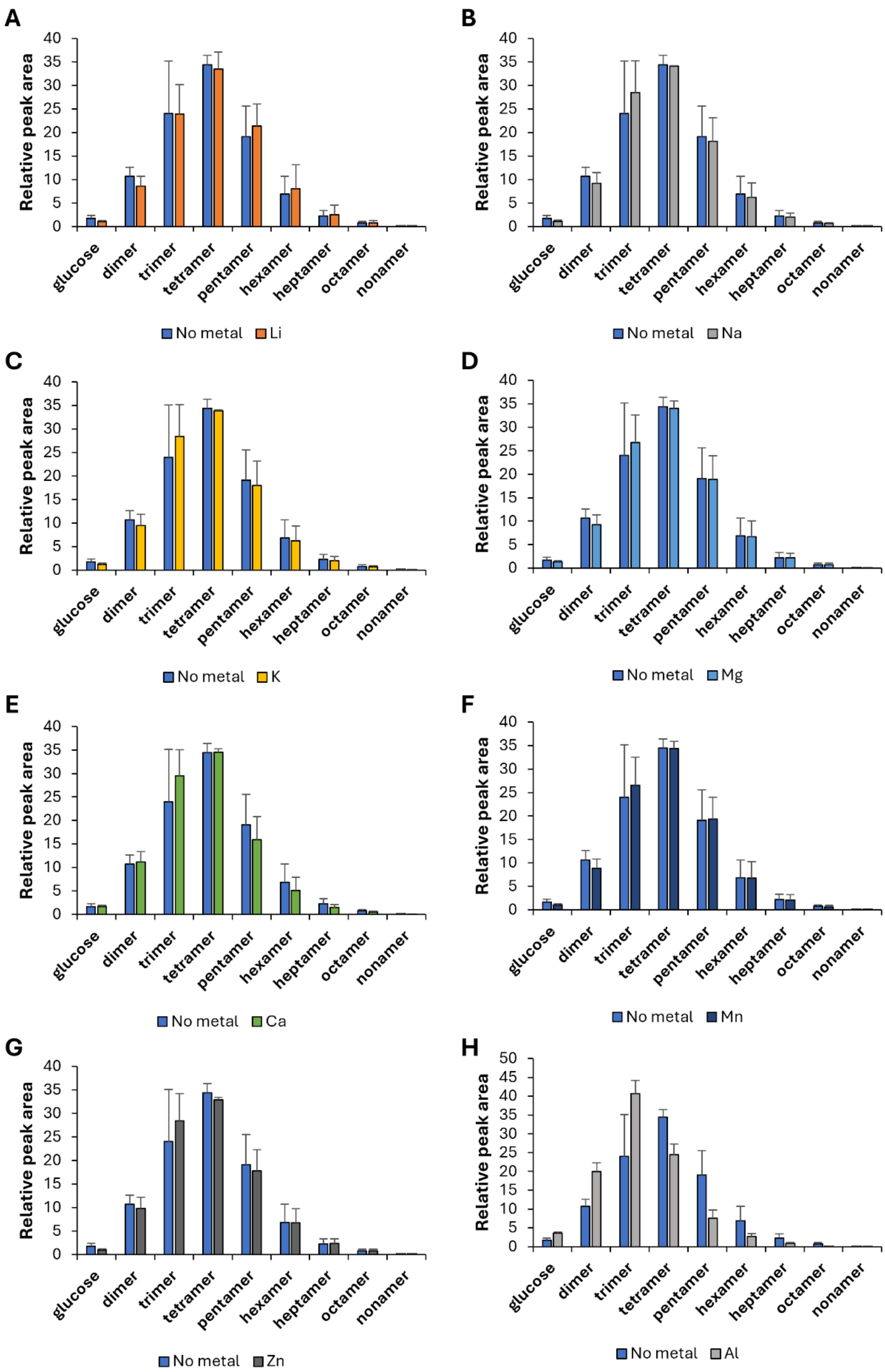

Figure S169. Relative distribution of D-Glucose oligomerization products based on SIM-LC-MS under aerobic conditions at 10 mM concentration (referring to glucose concentration prior to the drying reaction). Glucose product distribution after one week of dry down at 50 °C in the absence or presence of Li^{+} (A), Na^{+} (B), K^{+} (C), Mg^{2+} (D), Ca^{2+} (E), Mn^{2+} (F), Zn^{2+} (G), Al^{3+} (H) under acidified conditions.

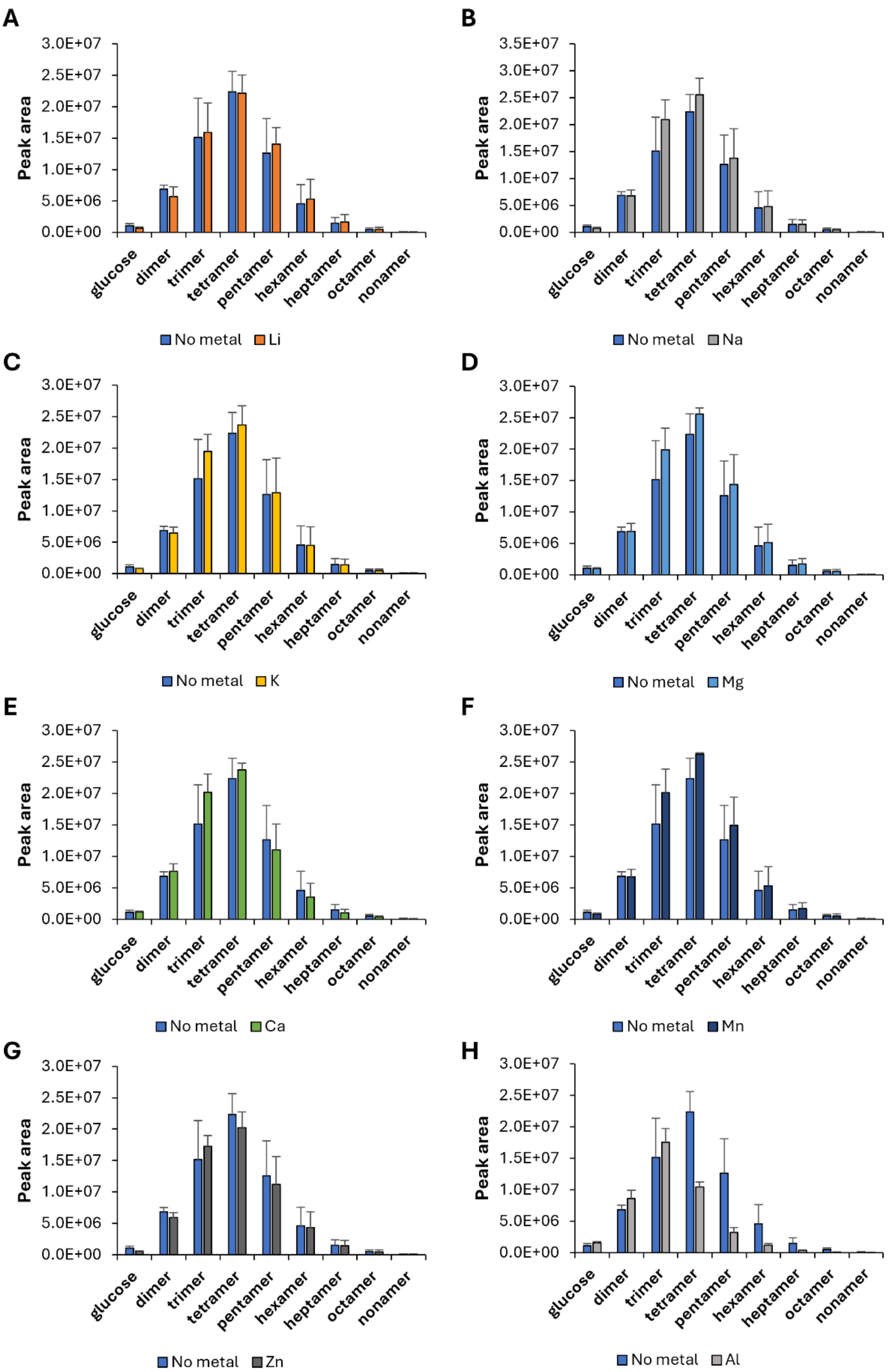

Figure S170. Absolute distribution of D-Glucose oligomerization products based on SIM-LC-MS under aerobic conditions at 10 mM concentration (referring to glucose concentration prior to the drying reaction). Glucose product distribution after one week of dry down at 50 °C in the absence or presence of Li^+ (A), Na^+ (B), K^+ (C), Mg^{2+} (D), Ca^{2+} (E), Mn^{2+} (F), Zn^{2+} (G), Al^{3+} (H) under acidified conditions.

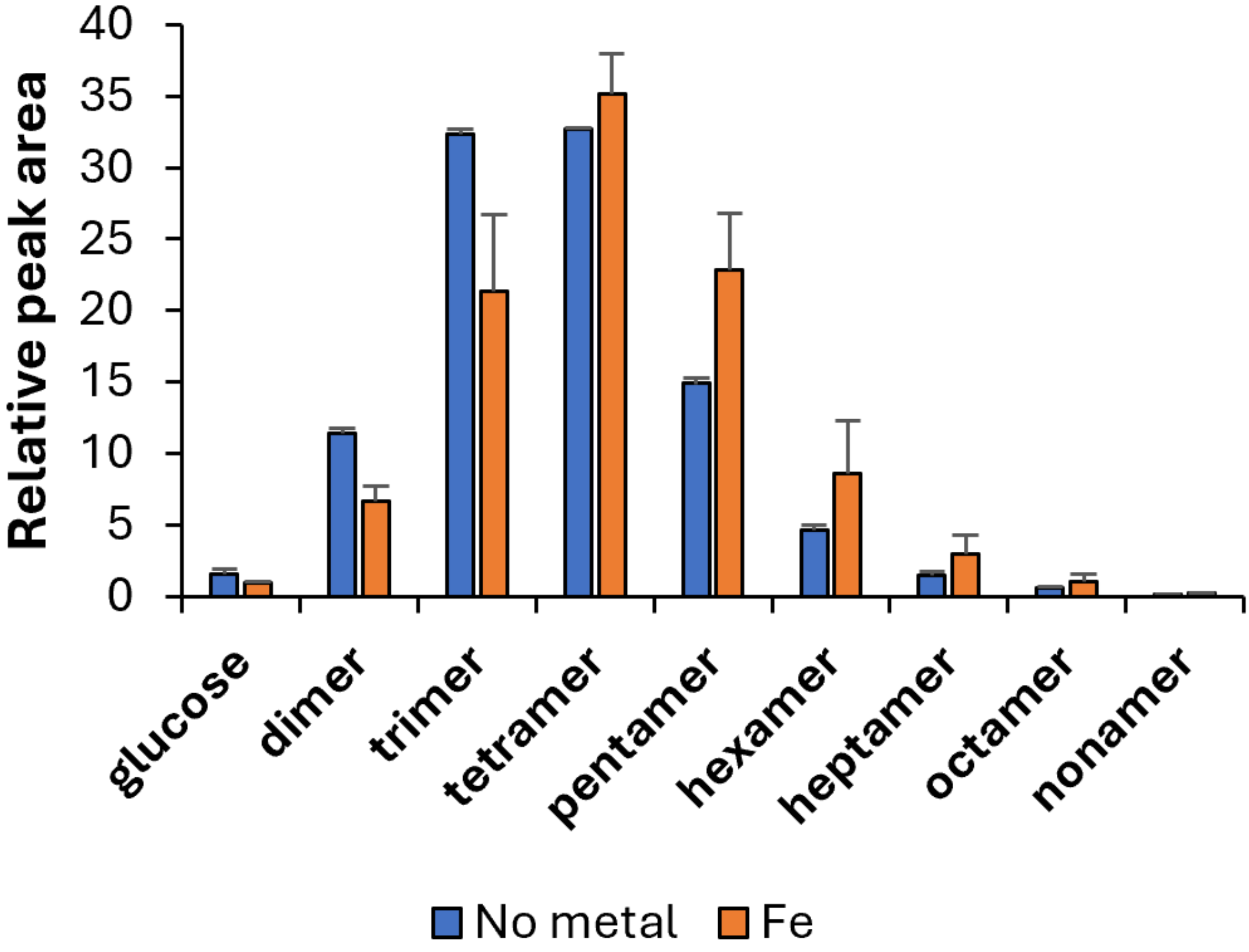

Figure S171. Relative distribution of D-Glucose oligomerization products based on SIM-LC-MS under anaerobic conditions at 10 mM concentration (referring to glucose concentration prior to the drying reaction). Glucose product distribution after one week of dry down at 50 °C in the absence or presence of Fe^{2+} under acidified conditions.

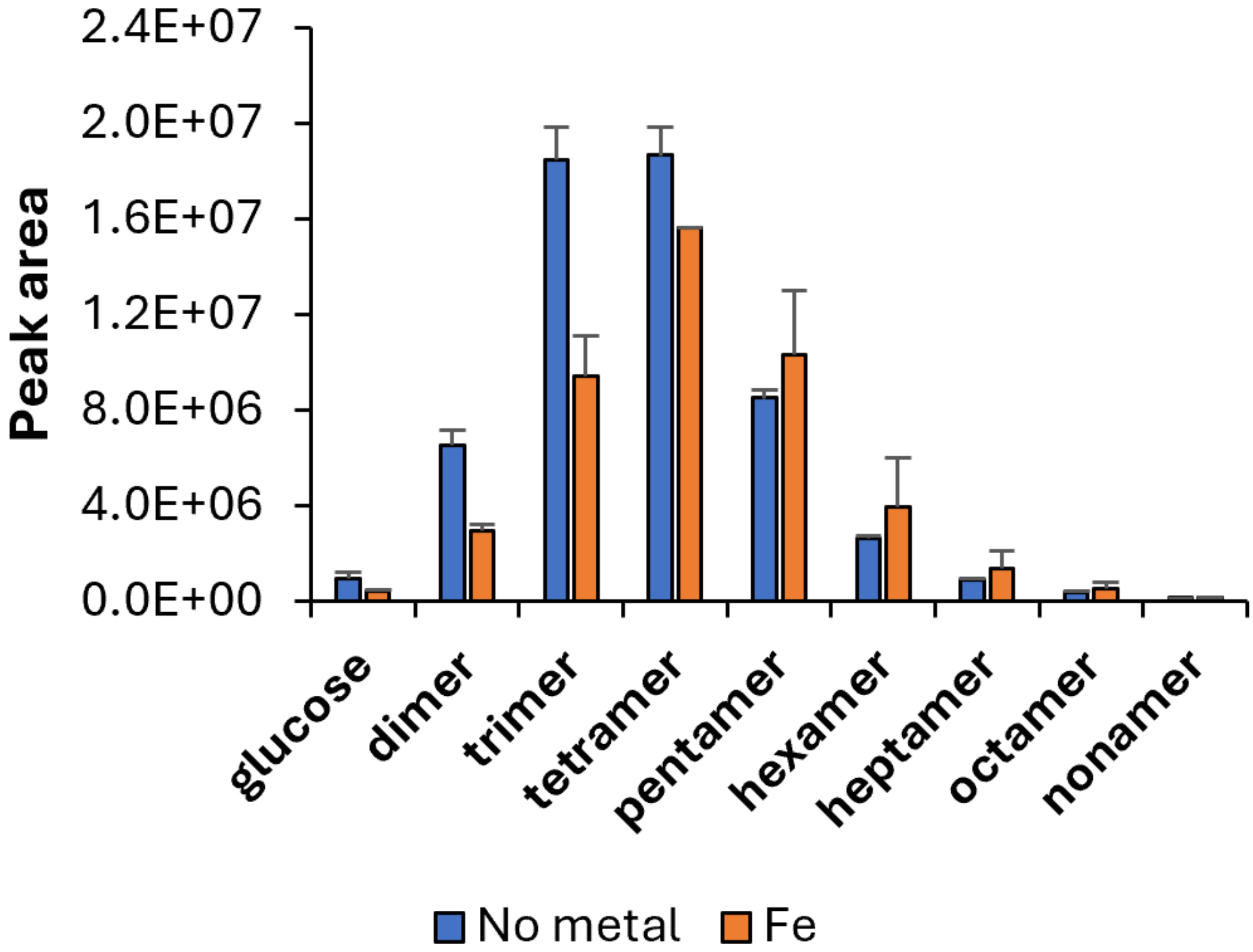

Figure S172. Absolute distribution of D-Glucose oligomerization products based on SIM-LC-MS under anaerobic conditions at 10 mM concentration (referring to glucose concentration prior to the drying reaction). Glucose product distribution after one week of dry down at 50 °C in the absence or presence of Fe^{2+} under acidified conditions.

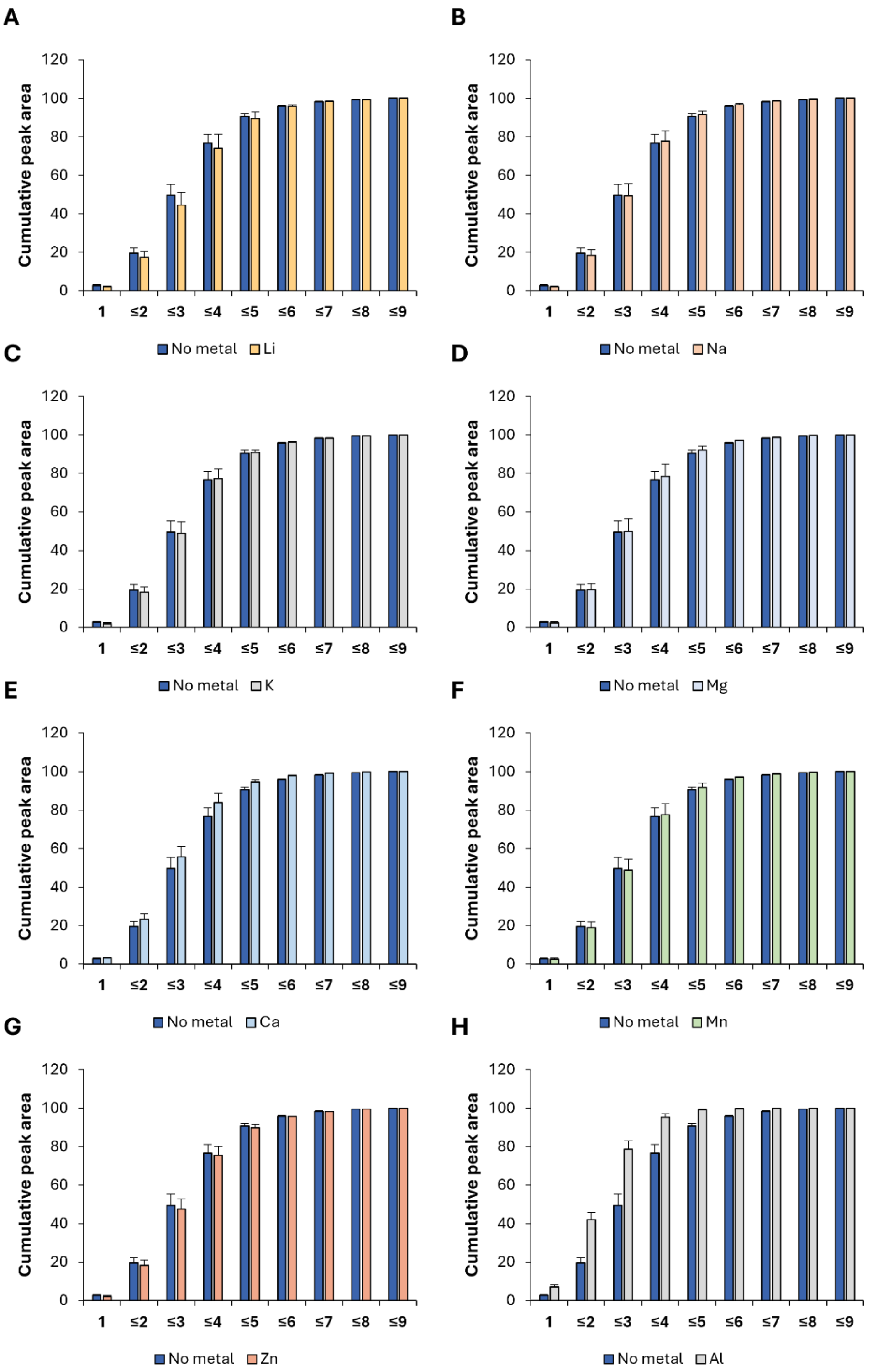

Figure S173. Cumulative distribution of D-Glucose oligomerization products based on SIM-SIM-LC-MS at 1 mM concentration (referring to glucose concentration prior to the drying reaction). Glucose product distribution after one week of dry down at 50 °C in the absence or presence of Li^{+} (A), Na^{+} (B), K^{+} (C), Mg^{2+} (D), Ca^{2+} (E), Mn^{2+} (F), Zn^{2+} (G), Al^{3+} (H) under acidified conditions.

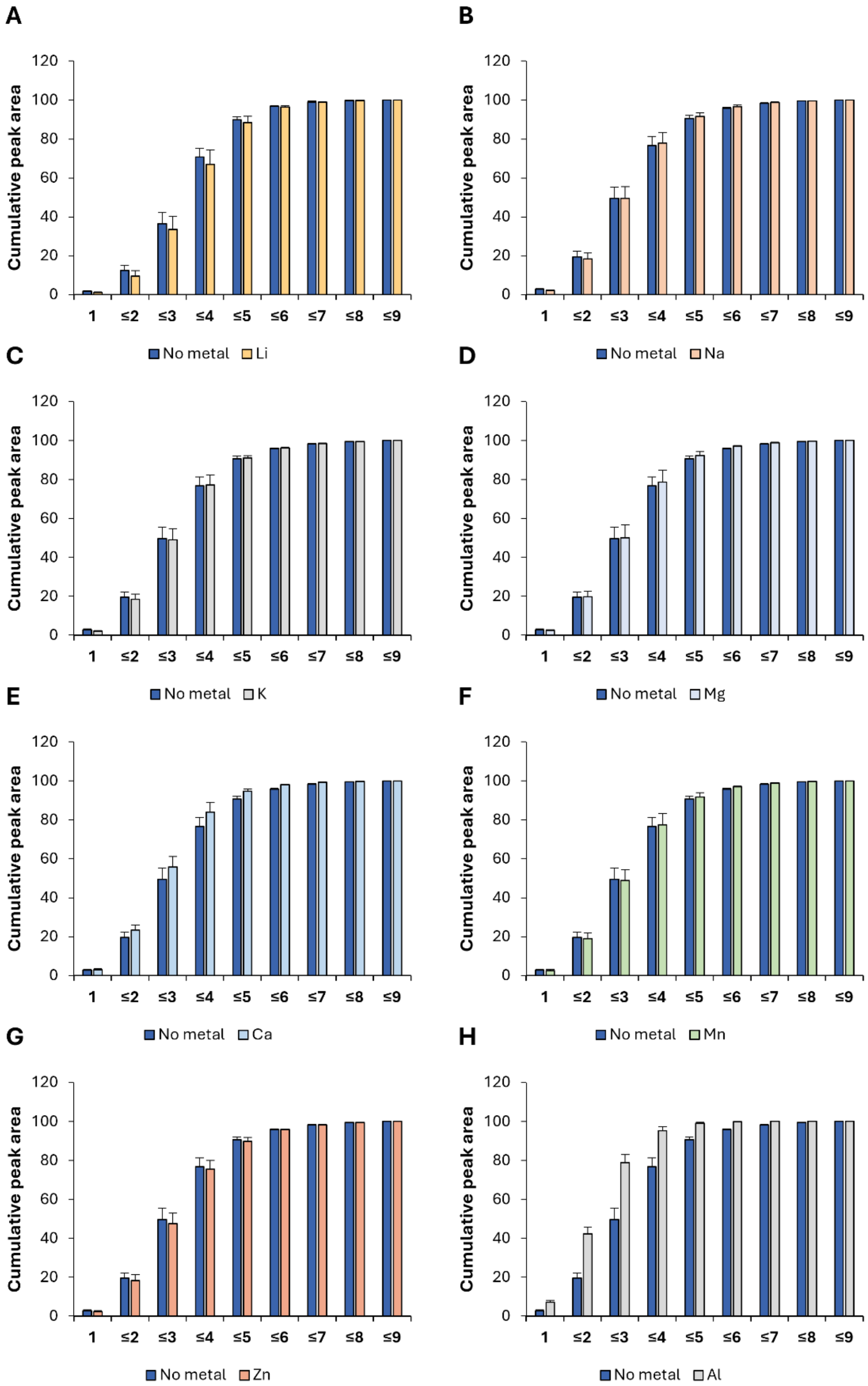

Figure S174. Cumulative distribution of D-Glucose oligomerization products based on SIM-LC-MS at 10 mM concentration (referring to glucose concentration prior to the drying reaction). Glucose product distribution after one week of dry down at 50 °C in the absence or presence of Li^{+} (A), Na^{+} (B), K^{+} (C), Mg^{2+} (D), Ca^{2+} (E), Mn^{2+} (F), Zn^{2+} (G), Al^{3+} (H) under acidified conditions.

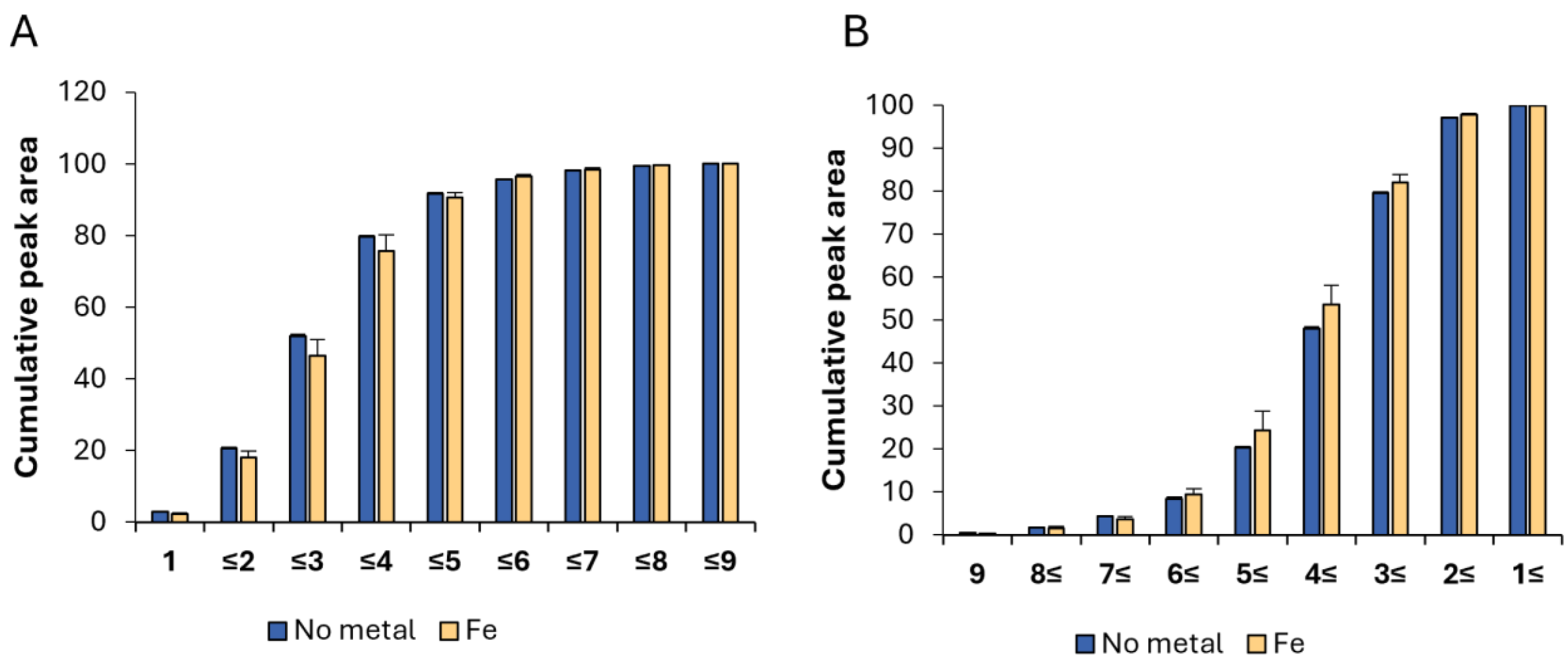

Figure S175. Cumulative distribution of D-Glucose oligomerization products based on SIM-LC-MS under anaerobic conditions at 1 mM concentration (referring to glucose concentration prior to the drying reaction). Glucose product distribution after one week of dry down at 50 °C in the absence or presence of Fe^{2+} under acidified conditions. Both the cumulative profile (A) as well as the reverse cumulative profile (B) of all species are shown.

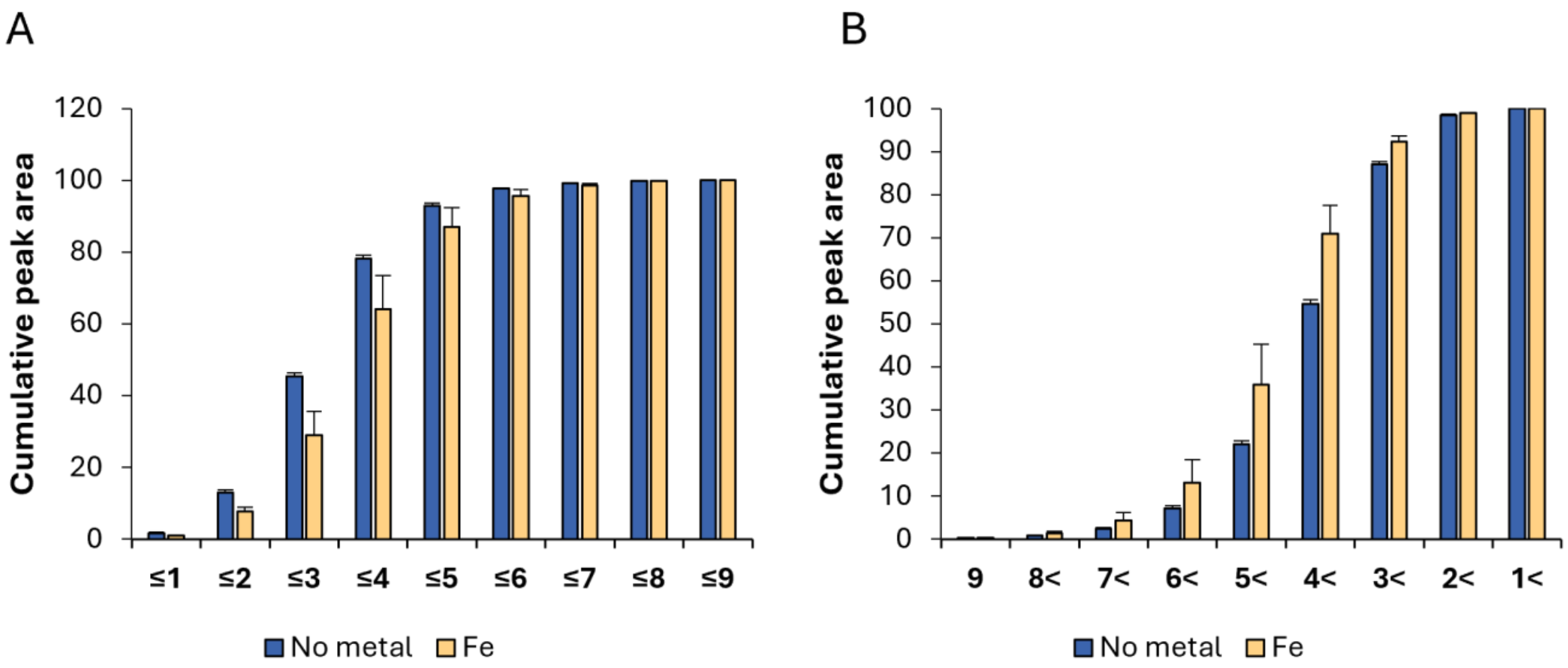

Figure S176. Cumulative distribution of D-Glucose oligomerization products based on SIM-LC-MS under anaerobic conditions at 10 mM concentration (referring to glucose concentration prior to the drying reaction). Glucose product distribution after one week of dry down at 50 °C in the absence or presence of Fe^{2+} under acidified conditions. Both the cumulative profile (A) as well as the reverse cumulative profile (B) of all species are shown.

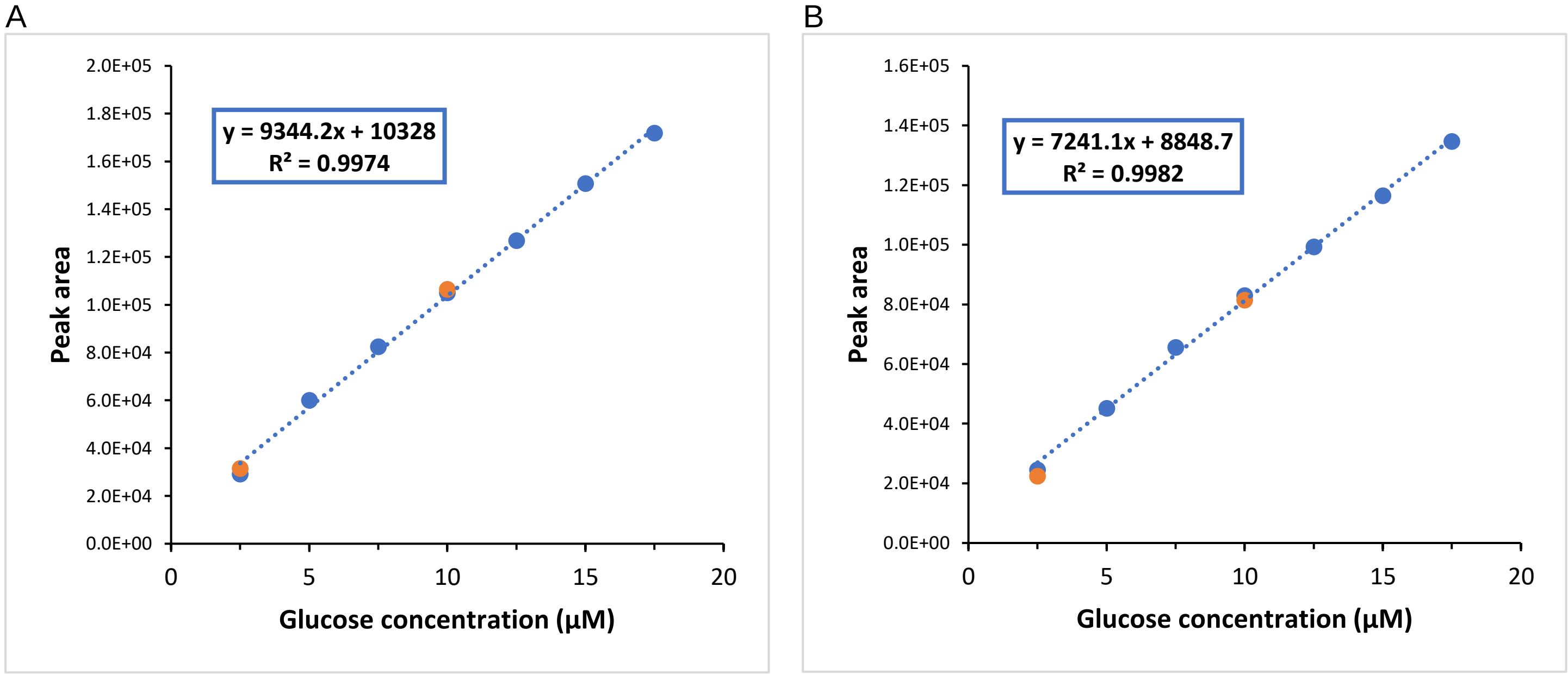

Figure S177. Calibration curves for glucose quantification by SIM-LC-MS. Glucose standards (2.5–17.5 µM) were analyzed by SIM-LC-MS in negative ionization mode using fragmentor voltages of 70 V (A) and 90 V (B). Quantification was based on the summed peak areas of the glucose ions corresponding to $[M-H]^-$ and $[M+HCOO]^-$. Blue points represent calibration standards used to generate the linear fit, and orange points represent independently prepared quality-control samples at 2.5 and 10 µM. The corresponding linear regression equations and R^2 values are shown in each panel.

Table S1. pH values for fresh and post-reaction mixtures.

sample	Acidified conditions fresh	Acidified conditions dry-down	Upon addition of NaOH fresh	Upon addition of NaOH dry-down
No metal	~2	~2	~10	~7
Li^{+}	~2	~3	~11	~9
Na^{+}	~2	~2	~11	~9
K^{+}	~2	~2	~11	~9
Mg^{2+}	~2	~3	~8	~6
Ca^{2+}	~2	~2	~8	~6
Mn^{2+}	~2	~2	~6	~6
Fe^{2+}	~2	~3	~5	~5
Zn^{2+}	~2	~2	~6	~7
Al^{3+}	~4	~4	~4	~4

Table S2. m/z values obtained by positive mode ESI-MS. Only detected species are shown.

	m/z									
Ionization species	Li^{+}	Na^{+}	K^{+}	Mg^{2+}	Ca^{2+}	Mn^{2+}	Fe^{2+}	Zn^{2+}	Al^{3+}	No metal ($M=H^{+}$)
$[Glu+M]^{n+}$	187	203	219	102	110	117.5	118	122	n.d.	181
$[2Glu+M]^{n+}$	349	365	381	183	191	198.5	199	203	n.d.	343
$[3Glu+M]^{n+}$	511	527	543	264	272	279.5	280	284	n.d.	505
$[4Glu+M]^{n+}$	673	689	705	345	353	360.5	361	365	231	667
$[5Glu+M]^{n+}$	835	851	867	426	434	441.5	442	446	n.d.	829
$[6Glu+M]^{n+}$	997	1013	1029	507	515	522.5	523	527	339	991
$[7Glu+M]^{n+}$	1159	1175	1191	588	596	603.5	604	608	n.d.	1153
$[8Glu+M]^{n+}$	1321	1337	1353	669	677	684.5	685	689	n.d.	1315
$[2\text{-}Glu+M]^{n+}$	367	383	399	192	200	207.5	208	212	n.d.	n.d.
$[3\text{-}Glu+M]^{n+}$	n.d.	n.d.	n.d.	282	290	297.5	298	302	n.d.	n.d.
$[4\text{-}Glu+M]^{n+}$	n.d.	n.d.	n.d.	372	380	387.5	388	392	n.d.	n.d.

- Hyphenated species (e.g., '2-Glu') correspond to non-covalent adducts of glucose monomers with metals ions.
- *M* symbolizes a metal ion and *n* symbolizes its charge.
- n.d. – not detected.

Table S3. m/z values of detected glucose oligomer. Species correspond to either $[M-H]^-$, $[M-Cl]^-$ or $[M+HCOO]^-$.

Oligomer	m/z ($[M-H]^-$)	m/z ($[M+Cl]^-$)	m/z ($[M+HCOO]^-$)
Monomers	179	215	225
Dimers	341	377	387
Trimers	503	539	549
Tetramers	665	701	711
Pentamers	827	863	873
Hexamers	989	1025	1035
Heptamers	1151	1187	1197
Octamers	1313	1349	1359
Nonamers	1475.5	1511.5	1521.5

Table S4. Statistical p-values for Figure 4 a-c based on three independent experiments.

Sample	P-Value		
	Acidified conditions (a)	Upon addition of NaOH (b)	Comparison between two conditions (c)
Li^+	0.253561	0.006065739	0.08591
Na^+	0.014521	0.005881685	0.01909
K^+	0.015027	0.57511144	0.080219
Mg^{2+}	0.00101	6.2337E-05	0.003403
Ca^{2+}	0.00159	0.000432998	0.005591
Mn^{2+}	9.74E-05	3.80039E-05	0.021275
Fe^{2+}	8.84E-05	4.47652E-05	0.007369
Zn^{2+}	0.283636	0.000602225	0.019816
Al^{3+}	0.00017	0.35950244	0.000801

Table S5. Statistical p-values for Figure 5 a-d based on three independent experiments.

Species	Production distribution Al^{3+} (a)	Production distribution Fe^{2+} (c)
Glucose monomer	0.0176	0.0998
dimer	0.0064	0.0124
trimer	0.1105	0.0695
tetramer	0.0112	0.2692
pentamer	0.0776	0.0720
hexamer	0.2018	0.1950
heptamer	0.1423	0.1828
octamer	0.0924	0.2651
nonamer	0.0006	0.0027

Species	Cumulative distribution Al^{3+} (b)
1	0.0176
≤2	0.0063
≤3	0.0540
≤4	0.1103
≤5	0.1721
≤6	0.1169
≤7	0.0657
≤8	0.0006
≤9	1.0000

Species	Cumulative distribution Fe^{2+} (d)
9	0.0022
≥8	0.3561
≥7	0.4487
≥6	0.5411
≥5	0.4875
≥4	0.4624
≥3	0.0644
≥2	0.1635
≥1	1.0000
