# Supplementary material for: Metal ions affect abiotic sugar oligomerization reactions
Source: Commun Chem. 2026 Jul 13;9:345. doi: 10.1038/s42004-026-02129-w (PMC13737047; doi:10.1038/s42004-026-02129-w)
Supplement: Supplementary file 3 [file 42004_2026_2129_MOESM3_ESM.pdf]

Description of Additional Supplementary Files:

File: Supplementary Data

Description: NMR spectra integrations
