# Supplementary material for: Metal ions affect abiotic sugar oligomerization reactions
Source: Commun Chem. 2026 Jul 13;9:345. doi: 10.1038/s42004-026-02129-w (PMC13737047; doi:10.1038/s42004-026-02129-w)

Supplementary Information
Full NMR Spectra Integrations

Metal Ions Affect Abiotic Sugar Oligomerization Reactions

Daniil Akulenko[a], Rotem Edri[a], and Moran Frenkel-Pinter[a,b,c*]

[a] Institute of Chemistry, The Hebrew University of Jerusalem, Jerusalem, Israel 9190401
[b] The Center for Nanoscience and Nanotechnology, The Hebrew University of Jerusalem, Jerusalem, Israel 9190401
[c] Casali Center of Applied Chemistry, The Hebrew University of Jerusalem, Jerusalem 9190401, Israel

Table of Contents

Figure 1. ^{1}H-NMR spectra of three independent dry-down reactions of glucose in the absence of metal ions under acidified conditions. 4

Figure 2. ^{1}H-NMR spectra of three independent dry-down reactions of glucose and LiCl under acidified conditions. 5

Figure 3. ^{1}H-NMR spectra of three independent dry-down reactions of glucose and $ZnCl_2$ under acidified conditions. 6

Figure 4. ^{1}H-NMR spectra of three independent dry-down reactions of glucose and NaCl under acidified conditions. 7

Figure 5. ^{1}H-NMR spectra of three independent dry-down reactions of glucose and KCl under acidified conditions. 8

Figure 6. ^{1}H-NMR spectra of three independent dry-down reactions of glucose and $MgCl_2$ under acidified conditions. 9

Figure 7. ^{1}H-NMR spectra of three independent dry-down reactions of glucose and $CaCl_2$ under acidified conditions. 10

Figure 8. ^{1}H-NMR spectra of three independent dry-down reactions of glucose and $MnCl_2$ under acidified conditions. 11

Figure 9. ^{1}H-NMR spectra of three independent dry-down reactions of glucose and $FeCl_2$ under acidified conditions. 12

Figure 10. ^{1}H-NMR spectra of three independent dry-down reactions of glucose and $AlCl_3$ under acidified conditions. 13

Figure 11. ^{1}H-NMR spectra of a fresh glucose under acidified conditions in the absence of metals. 14

Figure 12. ^{1}H-NMR spectra of a fresh glucose under acidified conditions in the presence of LiCl. 14

Figure 13. ^{1}H-NMR spectra of a fresh glucose under acidified conditions in the presence of $ZnCl_2$. 15

Figure 14. ^{1}H-NMR spectra of a fresh glucose under acidified conditions in the presence of NaCl. 15

Figure 15. ^{1}H-NMR spectra of a fresh glucose under acidified conditions in the presence of KCl. 16

Figure 16. ^{1}H-NMR spectra of a fresh glucose under acidified conditions in the presence of $MgCl_2$. 16

Figure 17. ^{1}H-NMR spectra of a fresh glucose under acidified conditions in the presence of $CaCl_2$. 17

Figure 18. ^{1}H-NMR spectra of a fresh glucose under acidified conditions in the presence of $MnCl_2$. 17

Figure 19. ^{1}H-NMR spectra of a fresh glucose under acidified conditions in the presence of $FeCl_2$. 18

Figure 20. ^{1}H-NMR spectra of a fresh glucose under acidified conditions in the presence of $AlCl_3$. 18

Figure 21. ^{1}H-NMR spectra of three independent dry-down reactions of glucose in the absence of metal ions upon addition of NaOH. 19
Figure 22. ^{1}H-NMR spectra of three independent dry-down reactions of glucose and LiCl upon addition of NaOH. 20
Figure 23. ^{1}H-NMR spectra of three independent dry-down reactions of glucose and $ZnCl_2$ upon addition of NaOH. 21
Figure 24. ^{1}H-NMR spectra of three independent dry-down reactions of glucose and NaCl upon addition of NaOH. 22
Figure 25. ^{1}H-NMR spectra of three independent dry-down reactions of glucose and KCl upon addition of NaOH. 23
Figure 26. ^{1}H-NMR spectra of three independent dry-down reactions of glucose and $MgCl_2$ upon addition of NaOH. 24
Figure 27. 1H-NMR spectra of three independent dry-down reactions of glucose and $CaCl_2$ upon addition of NaOH. 25
Figure 28. ^{1}H-NMR spectra of three independent dry-down reactions of glucose and $MnCl_2$ upon addition of NaOH. 26
Figure 29. ^{1}H-NMR spectra of three independent dry-down reactions of glucose and $FeCl_2$ upon addition of NaOH. 27
Figure 30. ^{1}H-NMR spectra of three independent dry-down reactions of glucose and $AlCl_3$ upon addition of NaOH. 28
Figure 31. ^{1}H-NMR spectra of a fresh glucose upon addition of NaOH in the absence of metals. 29
Figure 32. ^{1}H-NMR spectra of a fresh glucose upon addition of NaOH in the presence of LiCl. 29
Figure 33. ^{1}H-NMR spectra of a fresh glucose upon addition of NaOH in the presence of $ZnCl_2$. 30
Figure 34. ^{1}H-NMR spectra of a fresh glucose upon addition of NaOH in the presence of NaCl. 30
Figure 35. ^{1}H-NMR spectra of a fresh glucose upon addition of NaOH in the presence of KCl. 31
Figure 36. ^{1}H-NMR spectra of a fresh glucose upon addition of NaOH in the presence of $MgCl_2$. 31
Figure 37. ^{1}H-NMR spectra of a fresh glucose upon addition of NaOH in the presence of $CaCl_2$. 32
Figure 38. ^{1}H-NMR spectra of a fresh glucose upon addition of NaOH in the presence of $MnCl_2$. 32
Figure 39. ^{1}H-NMR spectra of a fresh glucose upon addition of NaOH in the presence of $FeCl_2$. 33
Figure 40. ^{1}H-NMR spectra of a fresh glucose upon addition of NaOH in the presence of $AlCl_3$. 33

Figure 1. ^{1}H-NMR spectra of three independent dry-down reactions of glucose in the absence of metal ions under acidified conditions. Glucose was dried at 50 °C for seven days in three replicates:

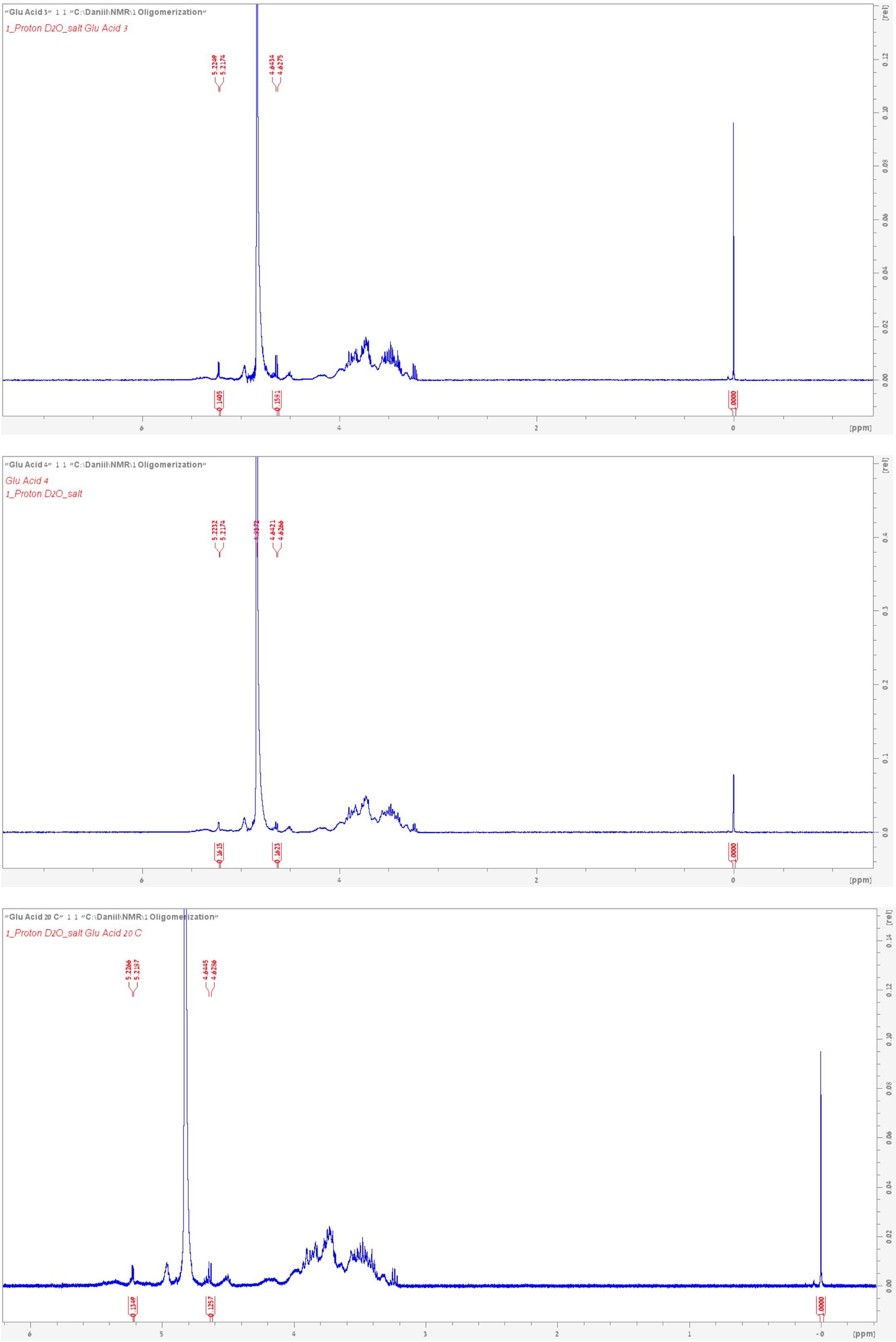

Figure 2. ^{1}H-NMR spectra of three independent dry-down reactions of glucose and LiCl under acidified conditions. Glucose was dried at 50 °C for seven days in three replicates:

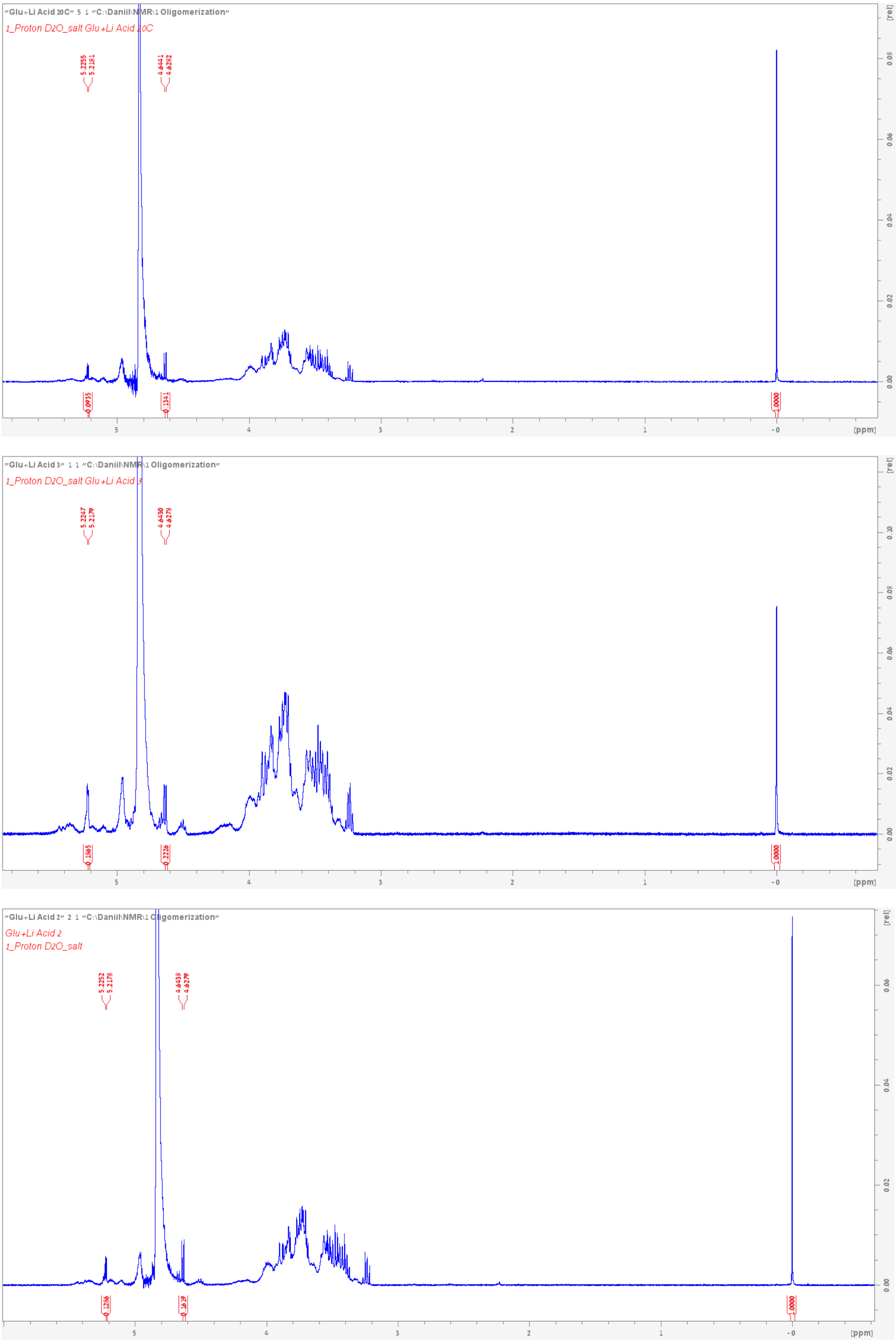

Figure 3. ^{1}H-NMR spectra of three independent dry-down reactions of glucose and $ZnCl_2$ under acidified conditions. Glucose was dried at 50 °C for seven days in three replicates:

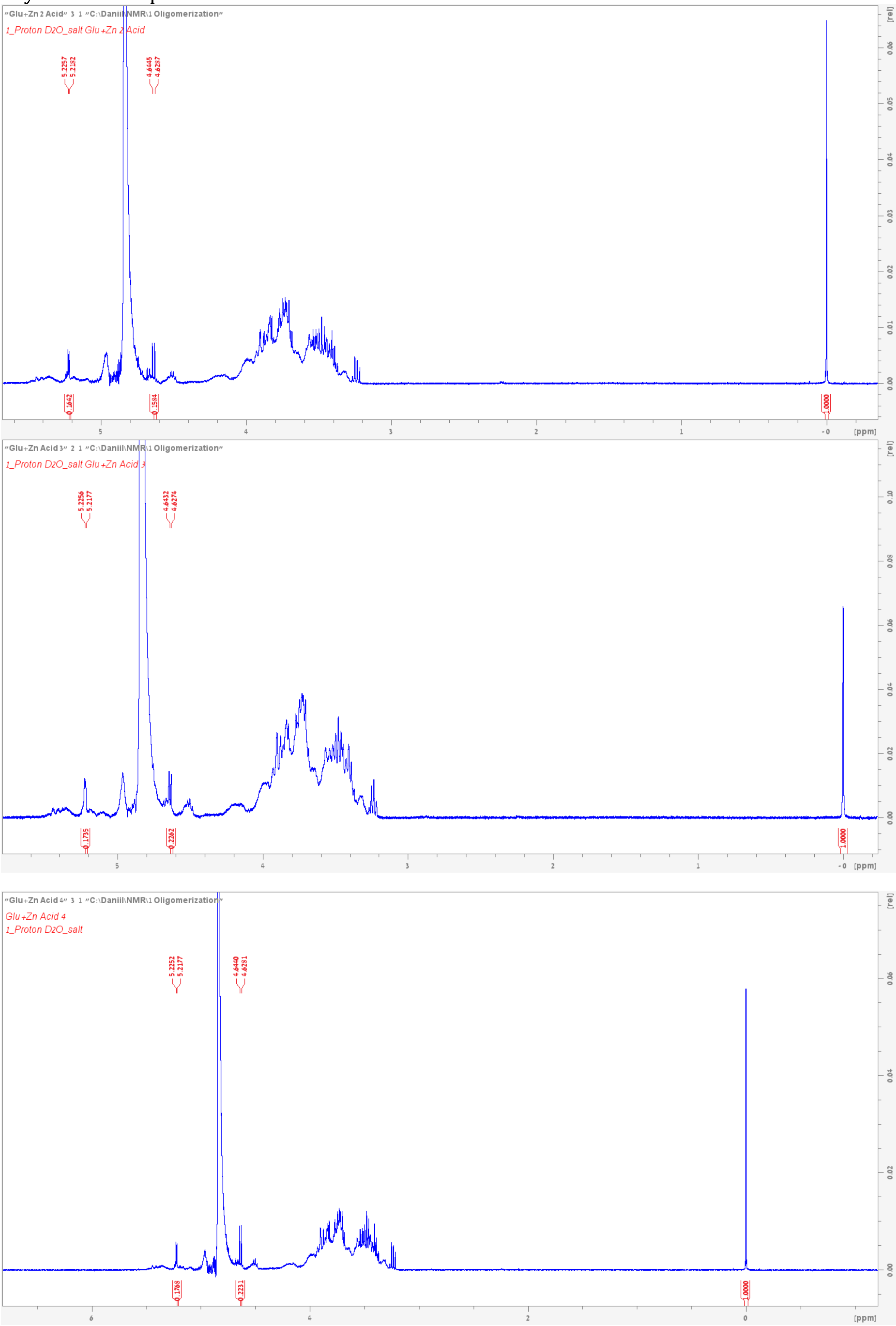

Figure 4. ^{1}H-NMR spectra of three independent dry-down reactions of glucose and NaCl under acidified conditions. Glucose was dried at 50 °C for seven days in three replicates:

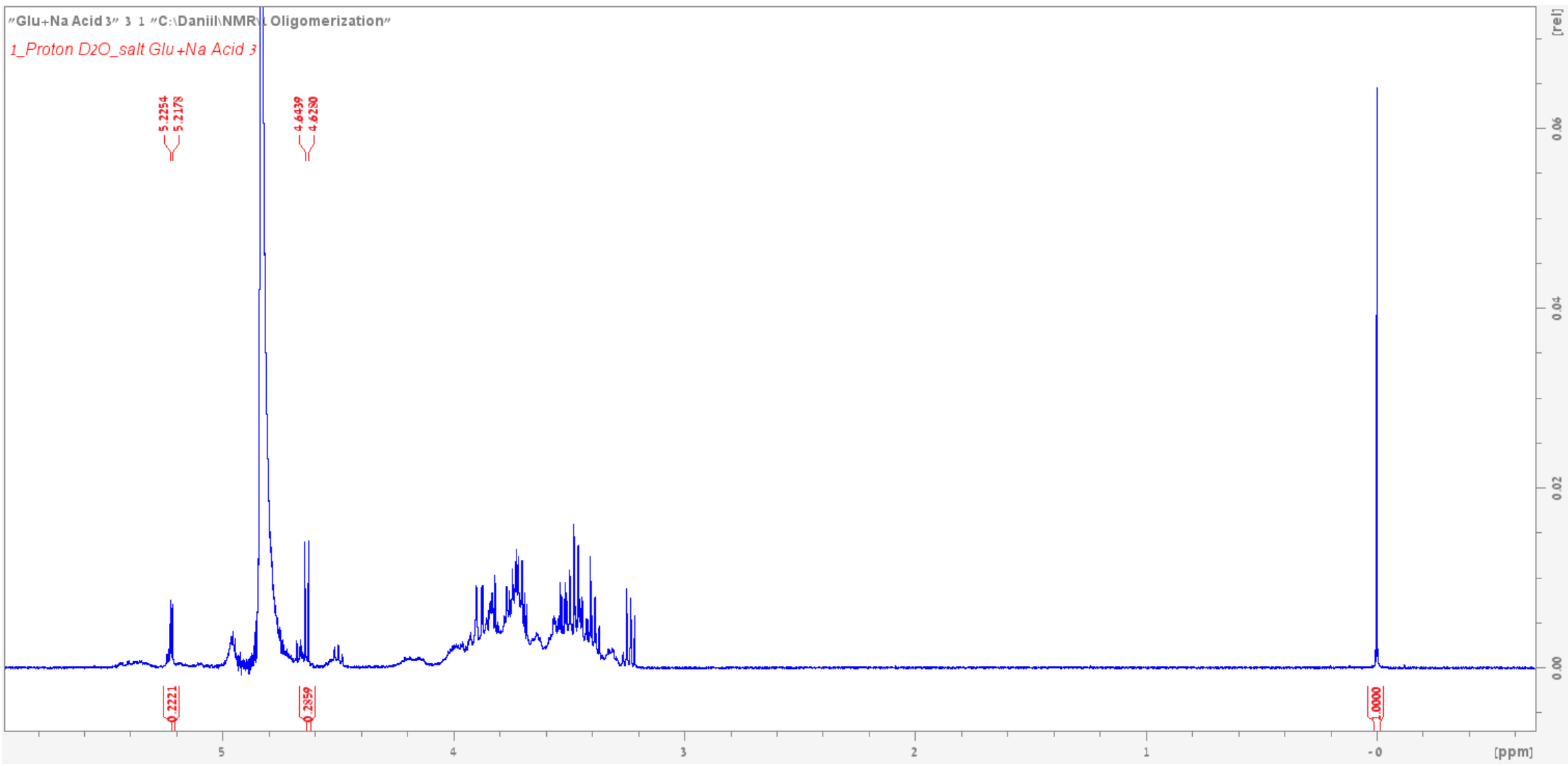

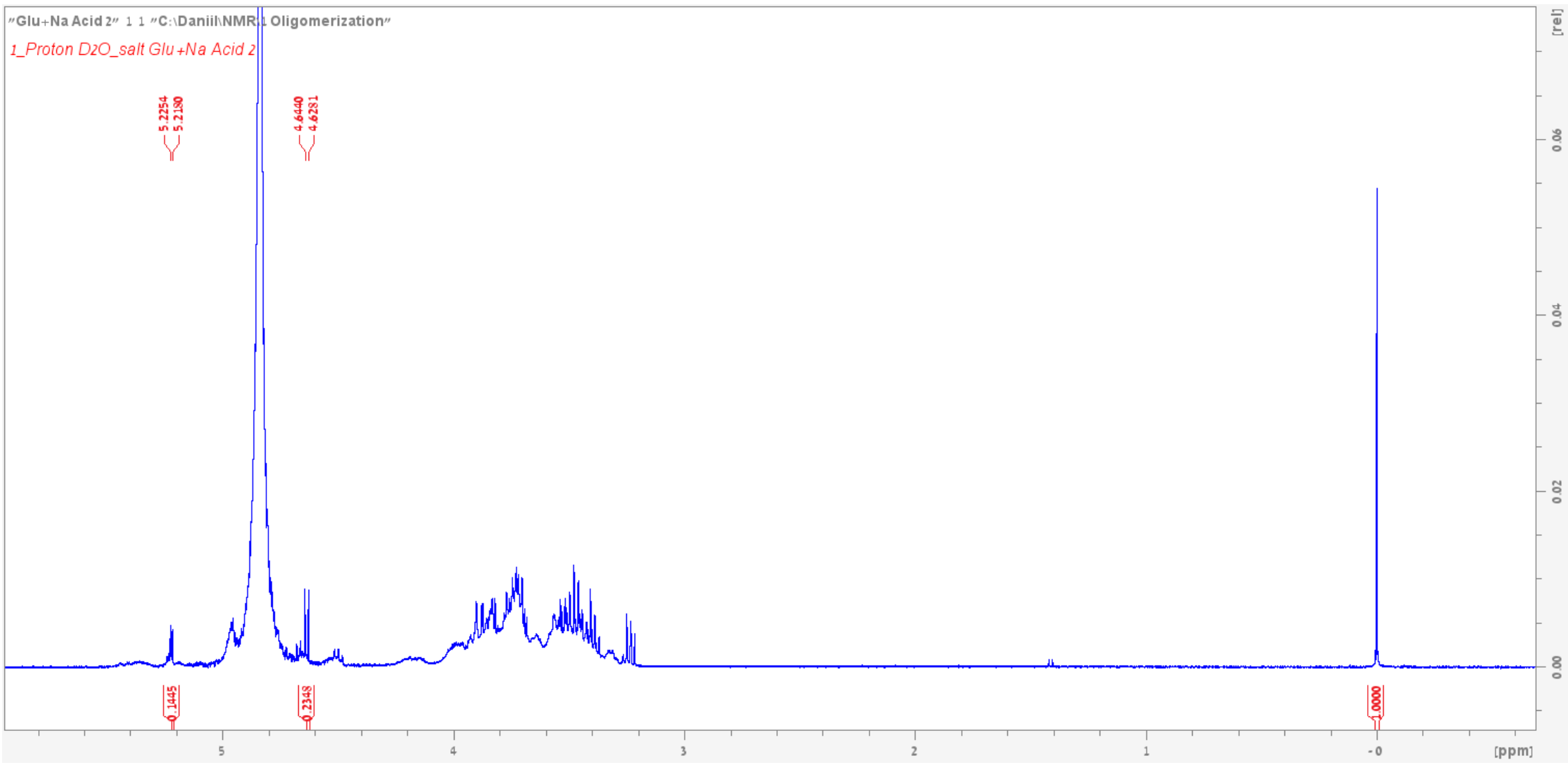

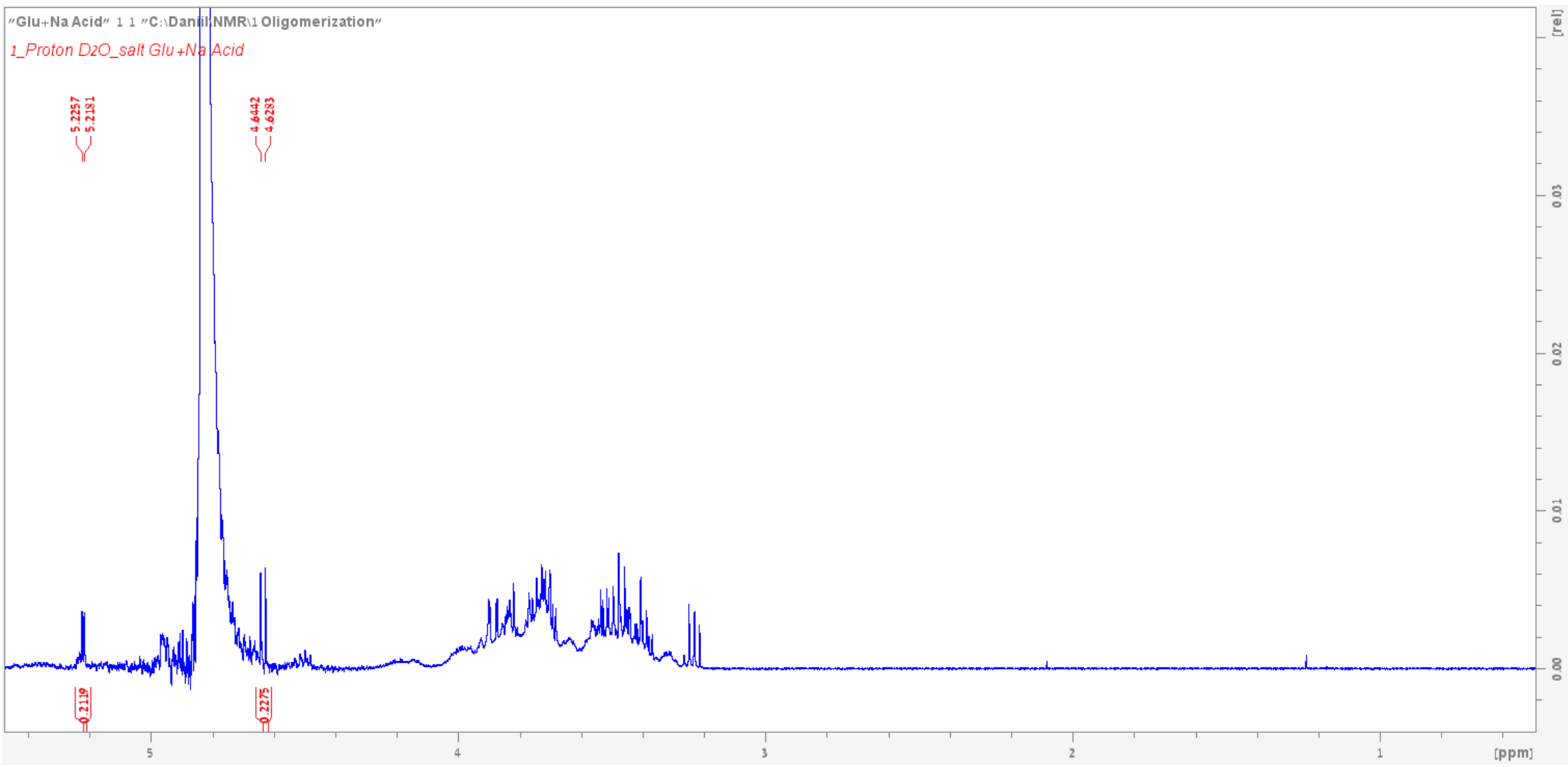

Figure 5. ^{1}H-NMR spectra of three independent dry-down reactions of glucose and KCl under acidified conditions. Glucose was dried at 50 °C for seven days in three replicates:

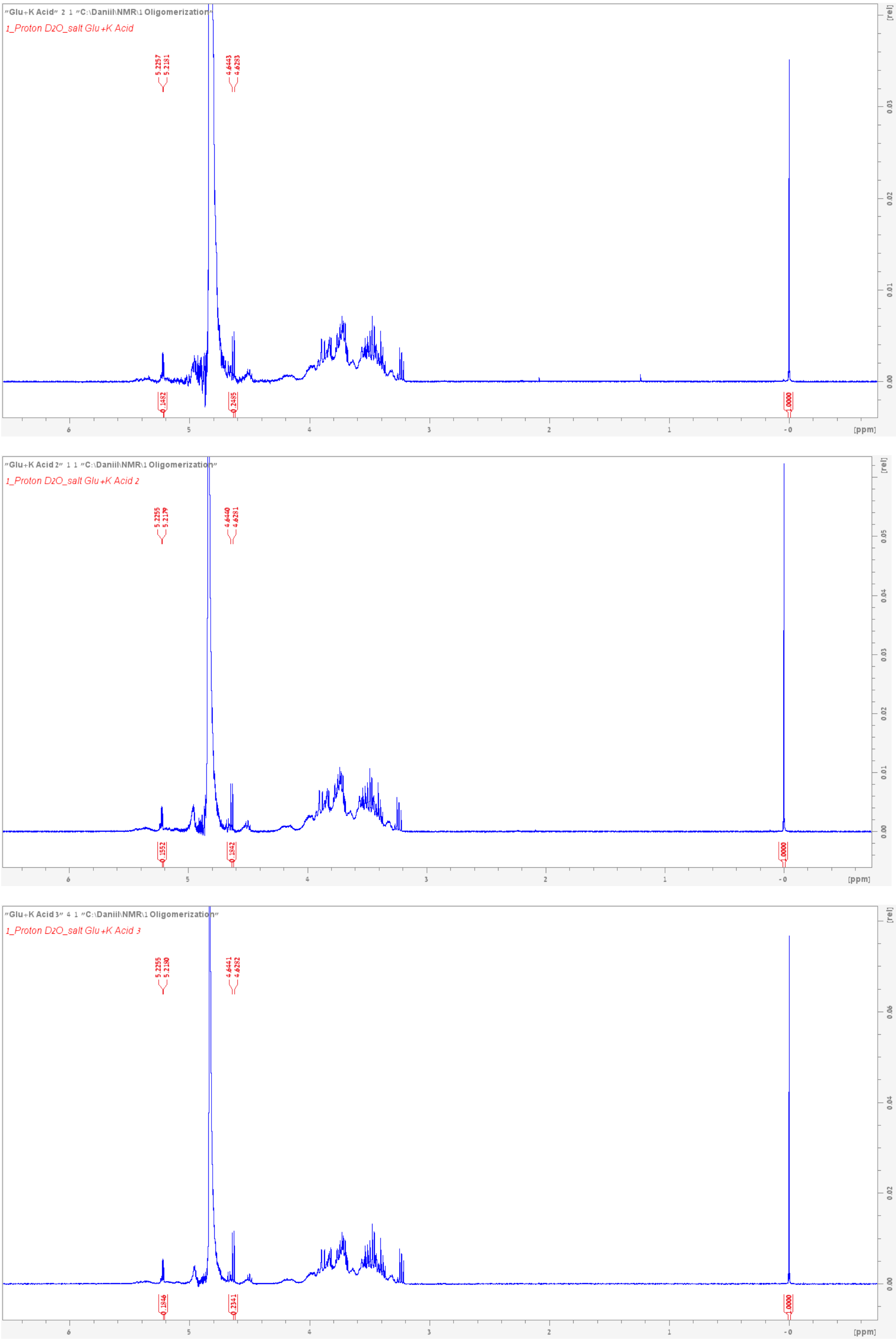

Figure 6. ^{1}H-NMR spectra of three independent dry-down reactions of glucose and $MgCl_2$ under acidified conditions. Glucose was dried at 50 °C for seven days in three replicates:

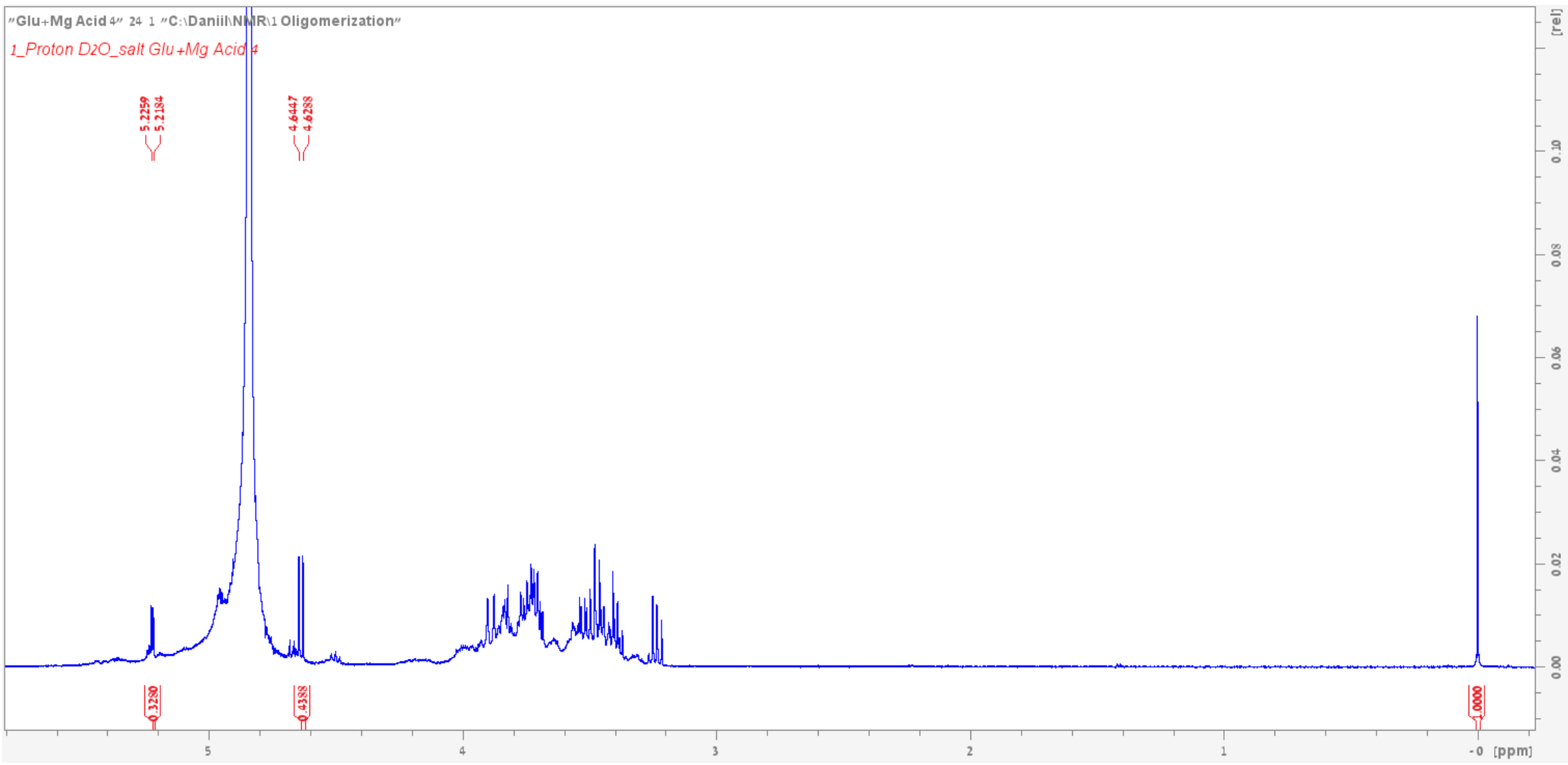

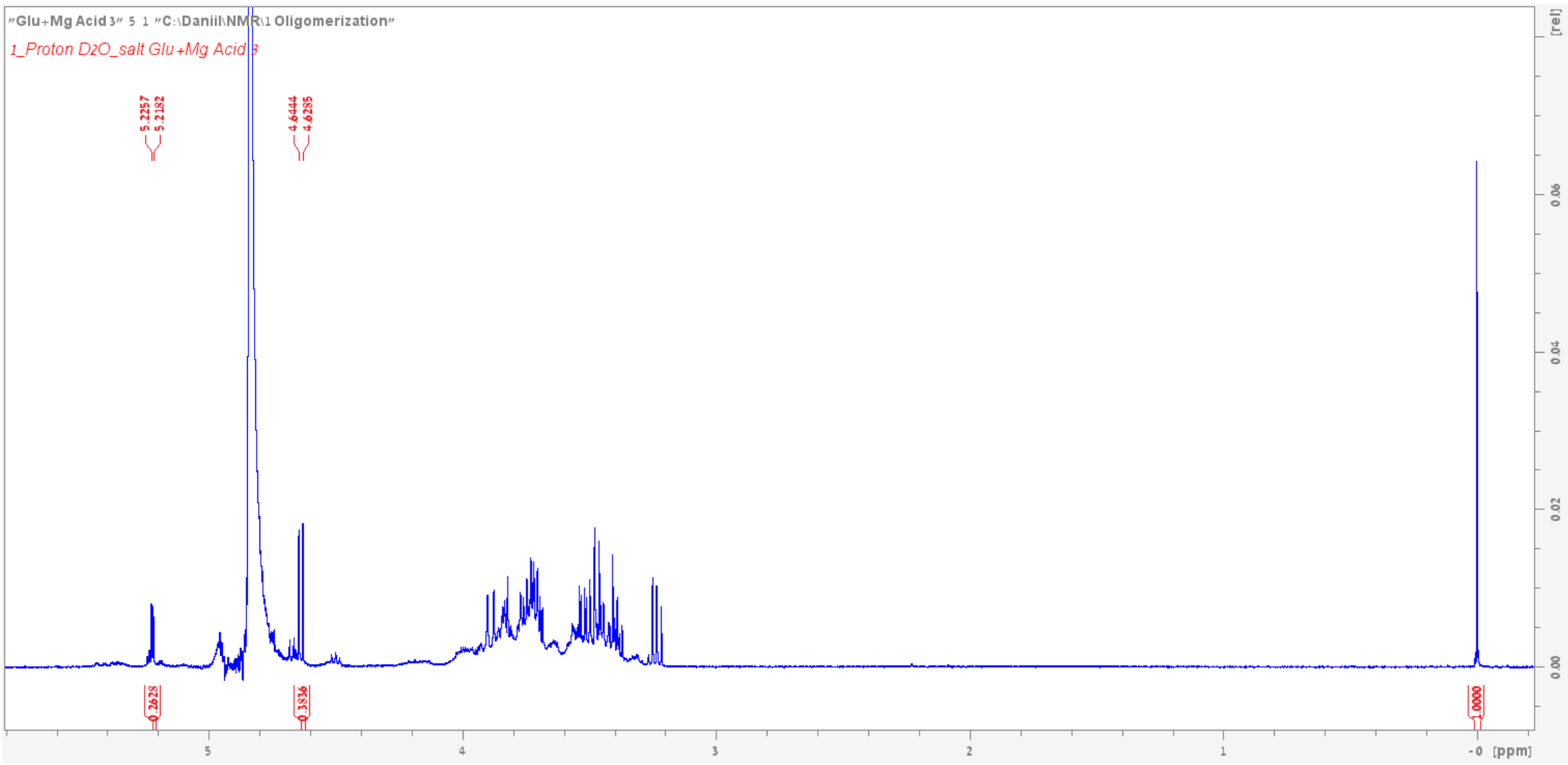

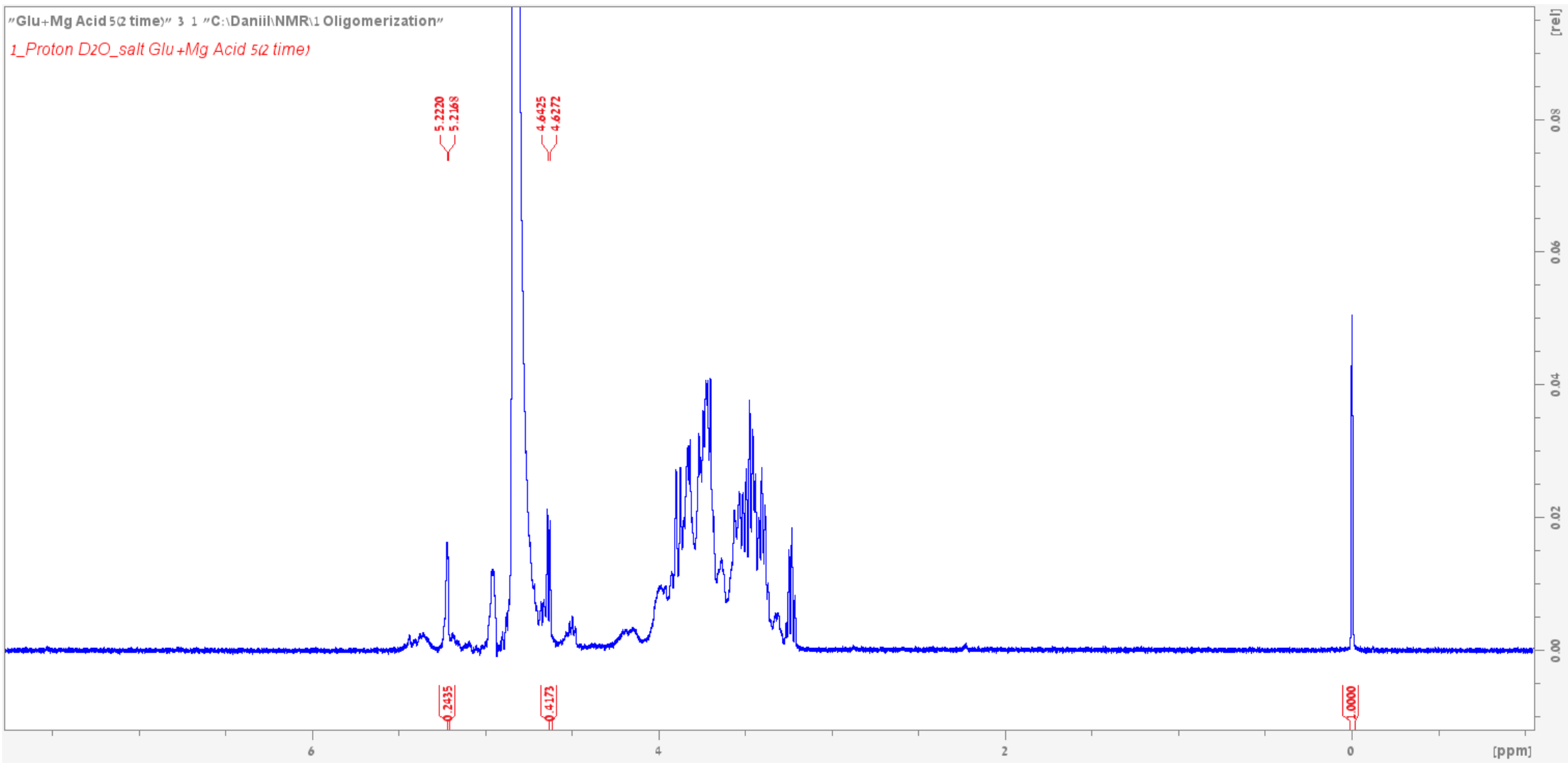

Figure 7. ^{1}H-NMR spectra of three independent dry-down reactions of glucose and $CaCl_2$ under acidified conditions. Glucose was dried at 50 °C for seven days in three replicates:

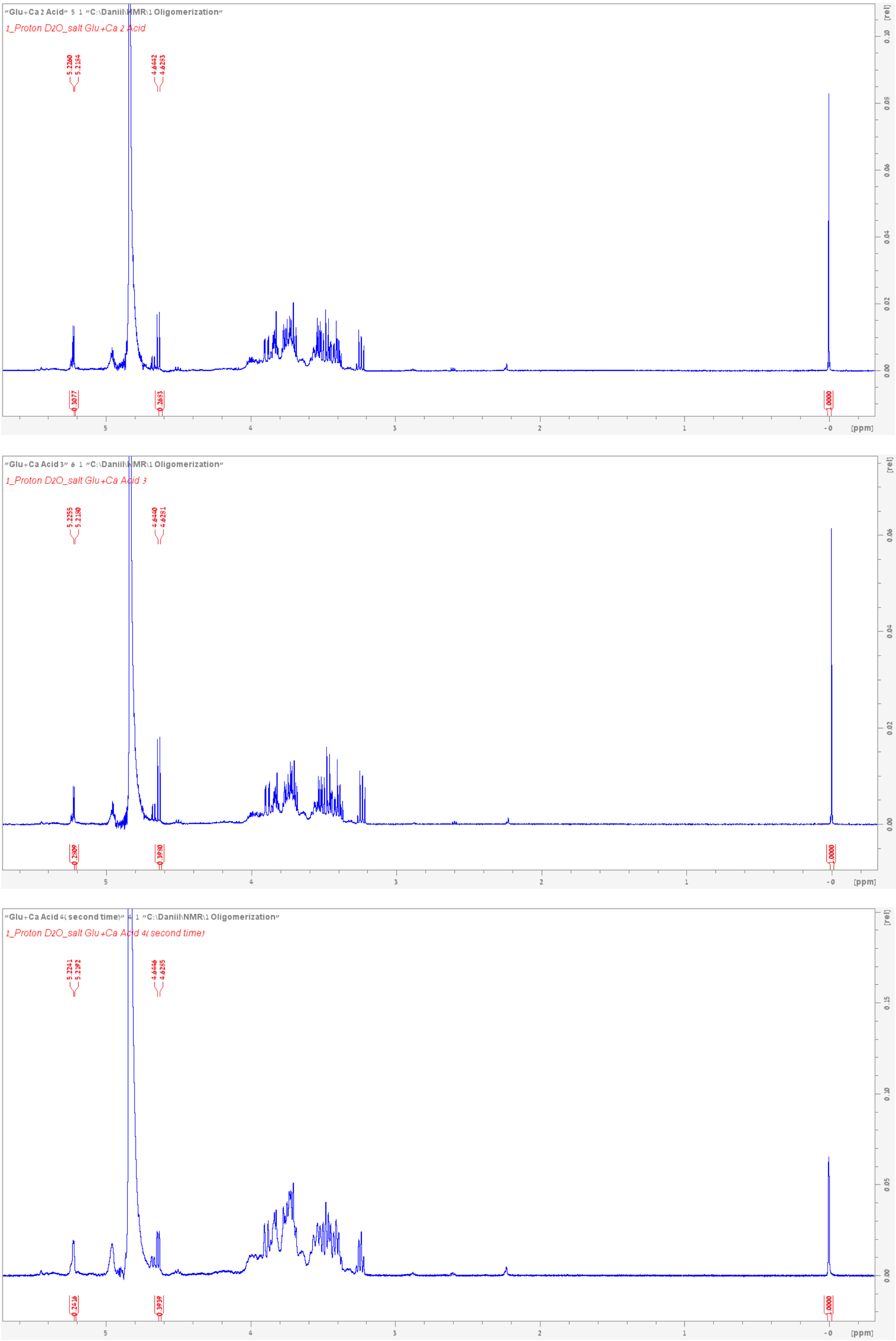

Figure 8. ^{1}H-NMR spectra of three independent dry-down reactions of glucose and $MnCl_2$ under acidified conditions. Glucose was dried at 50 °C for seven days in three replicates:

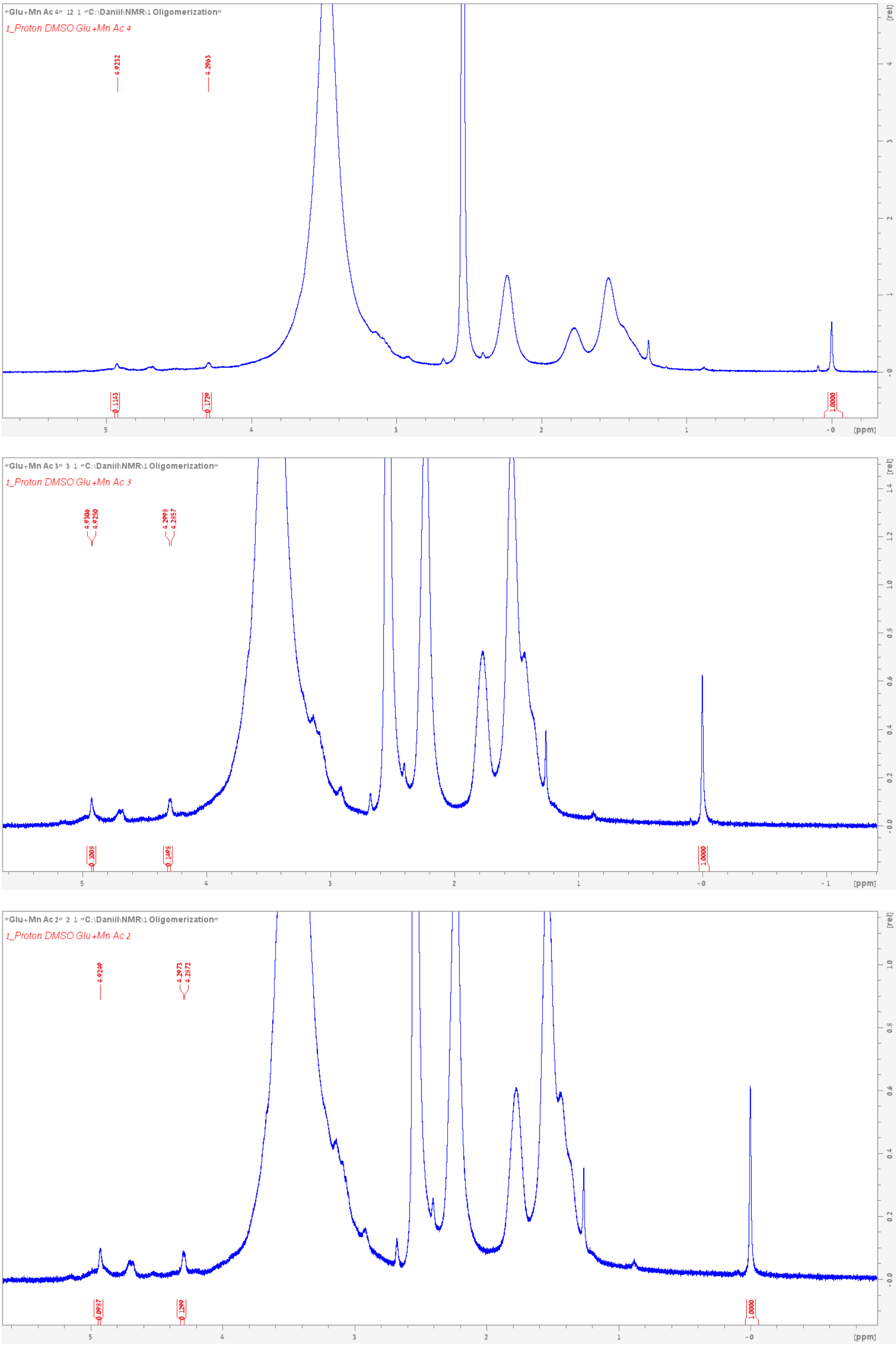

Figure 9. ^{1}H-NMR spectra of three independent dry-down reactions of glucose and $FeCl_2$ under acidified conditions. Glucose was dried at 50 °C for seven days in three replicates:

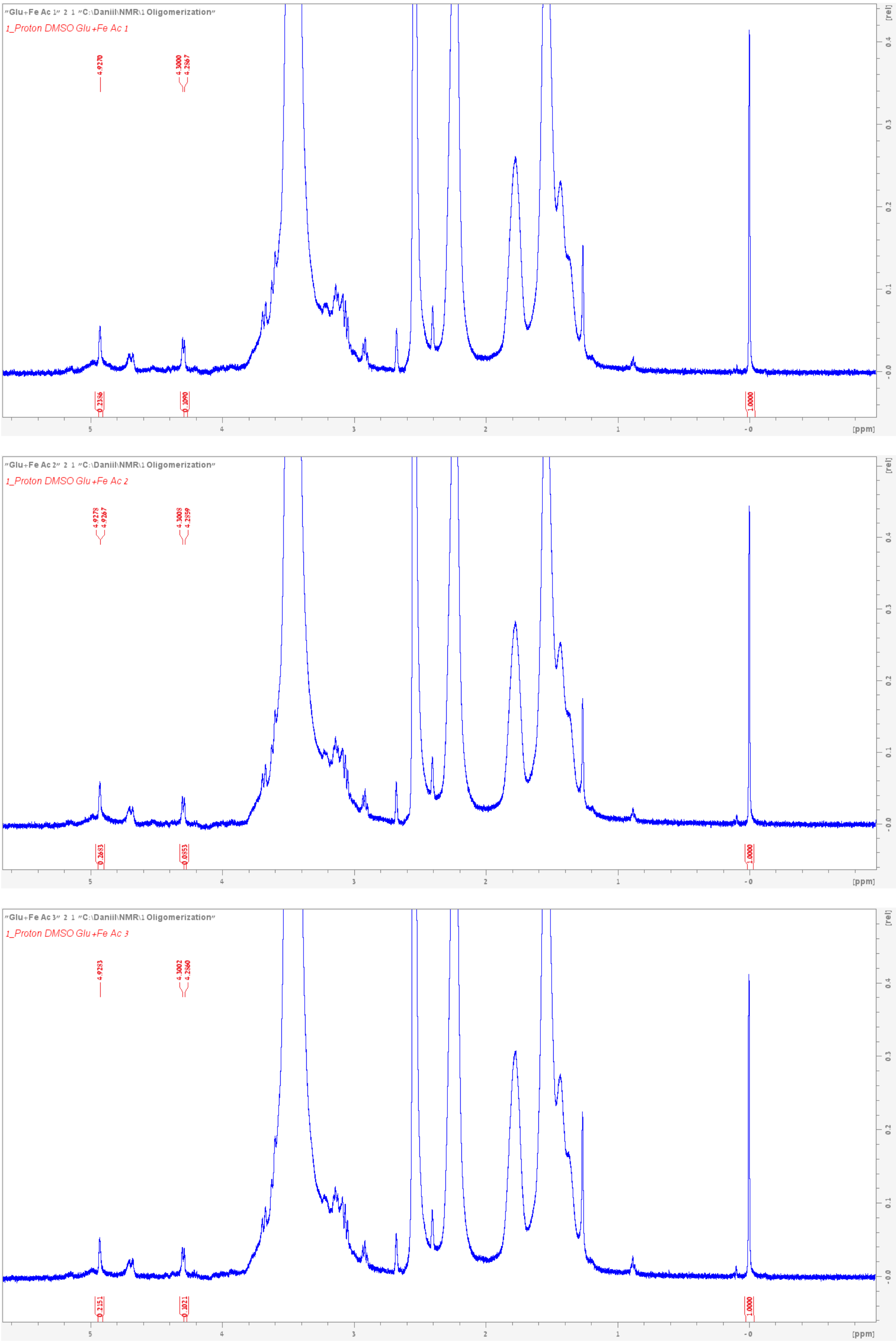

Figure 10. ^{1}H-NMR spectra of three independent dry-down reactions of glucose and $AlCl_3$ under acidified conditions. Glucose was dried at 50 °C for seven days in three replicates:

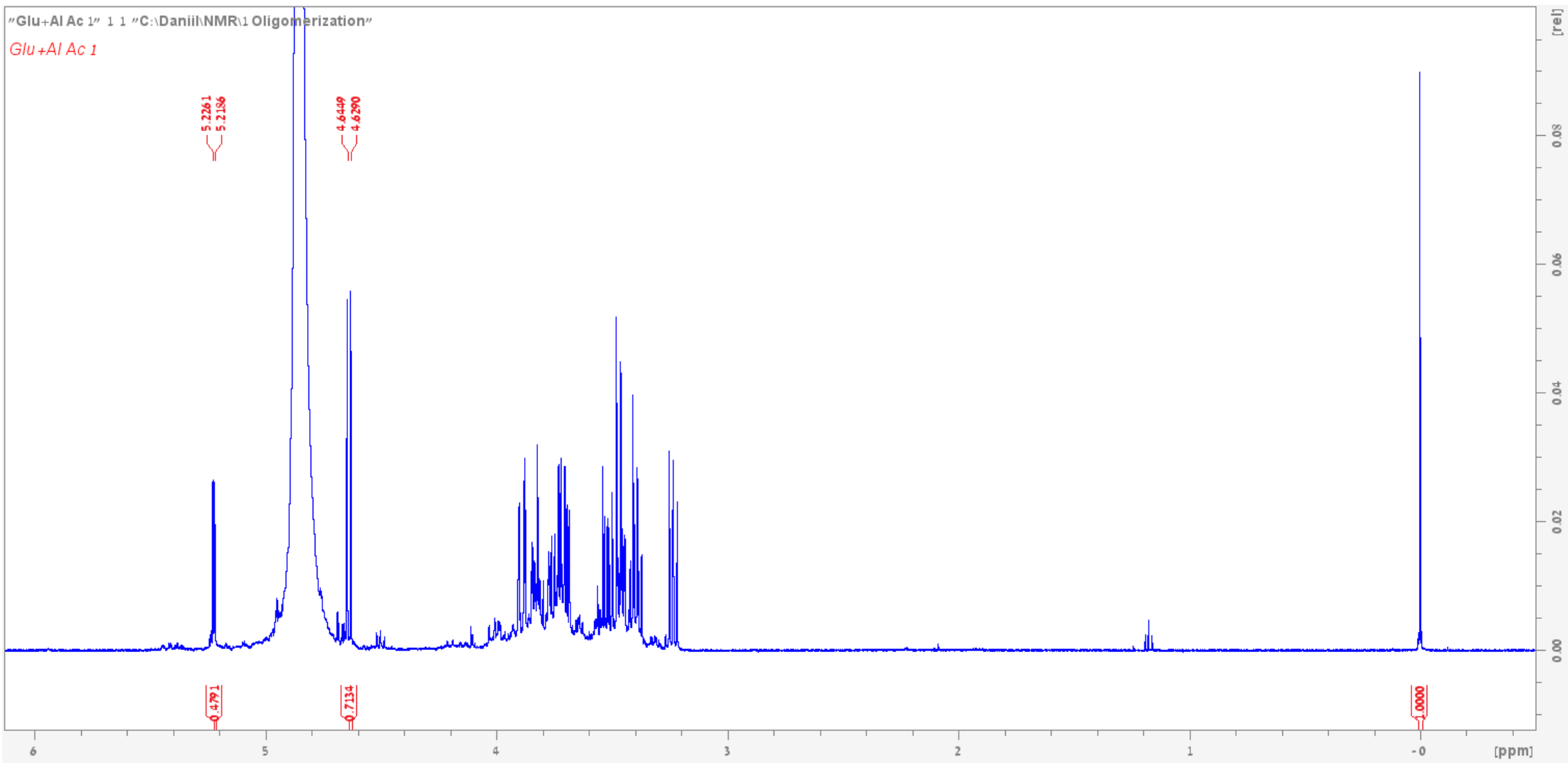

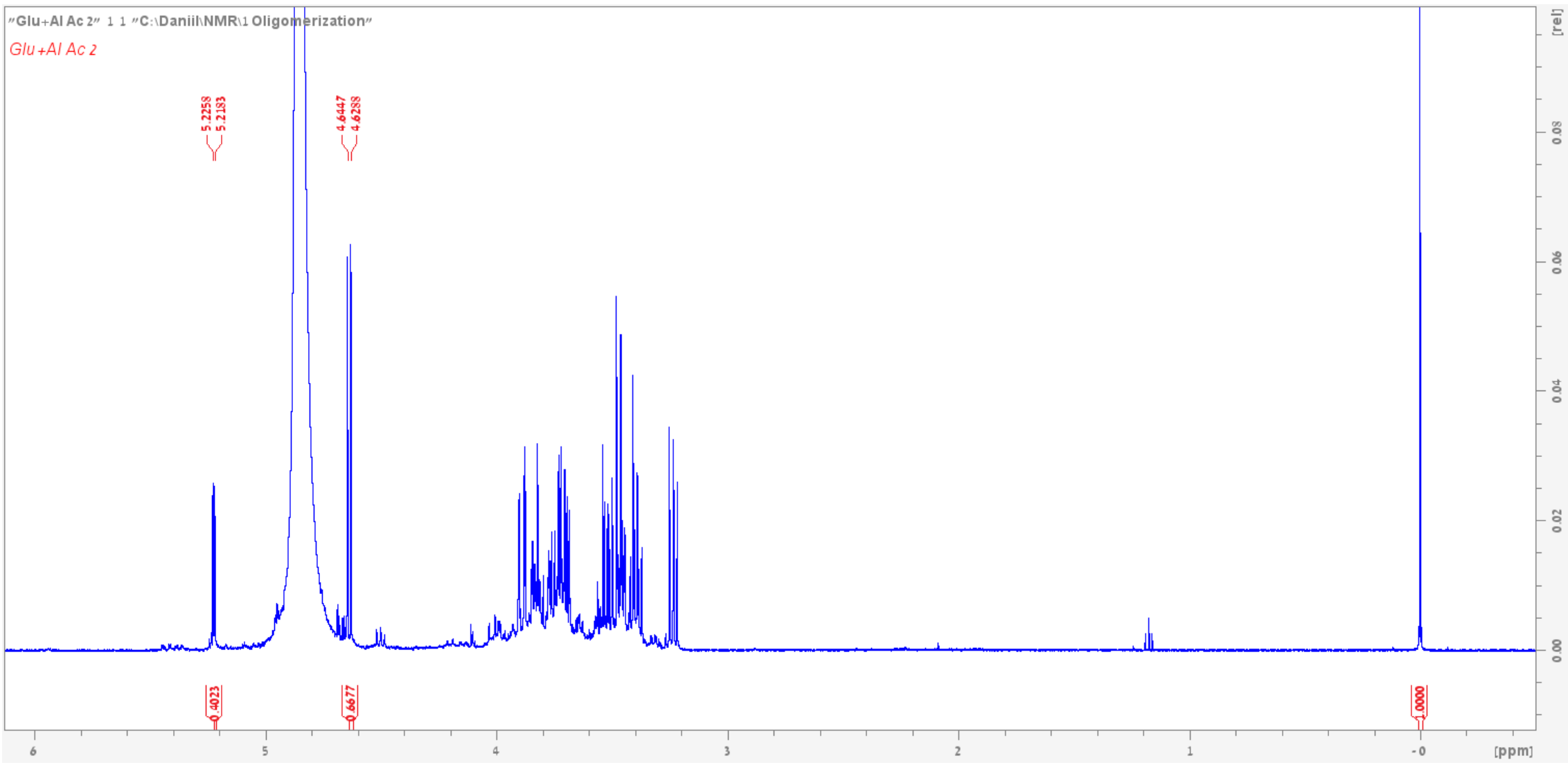

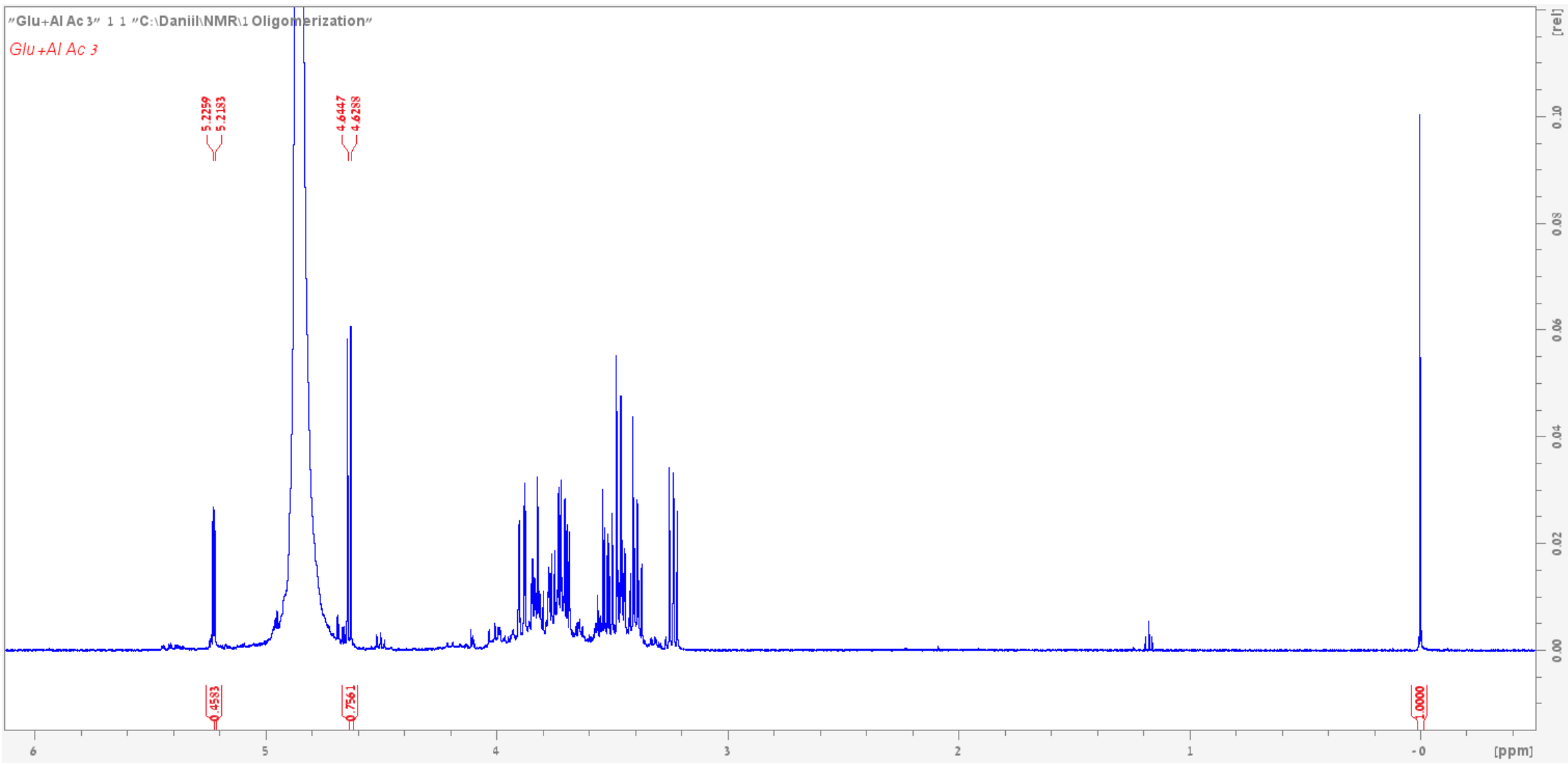

Figure 11. ^{1}H-NMR spectra of a fresh glucose under acidified conditions in the absence of metals.

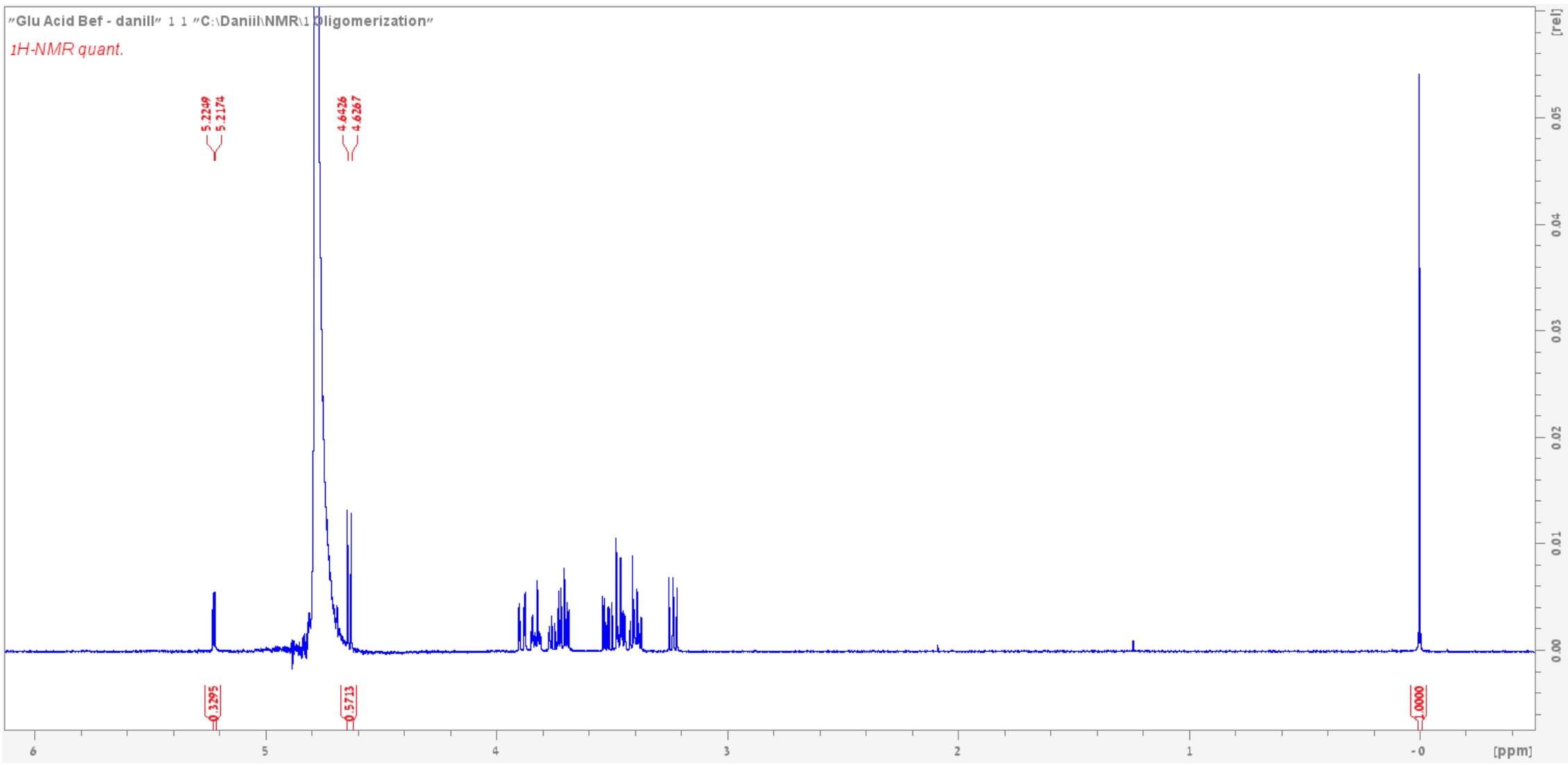

Figure 12. ^{1}H-NMR spectra of a fresh glucose under acidified conditions in the presence of LiCl.

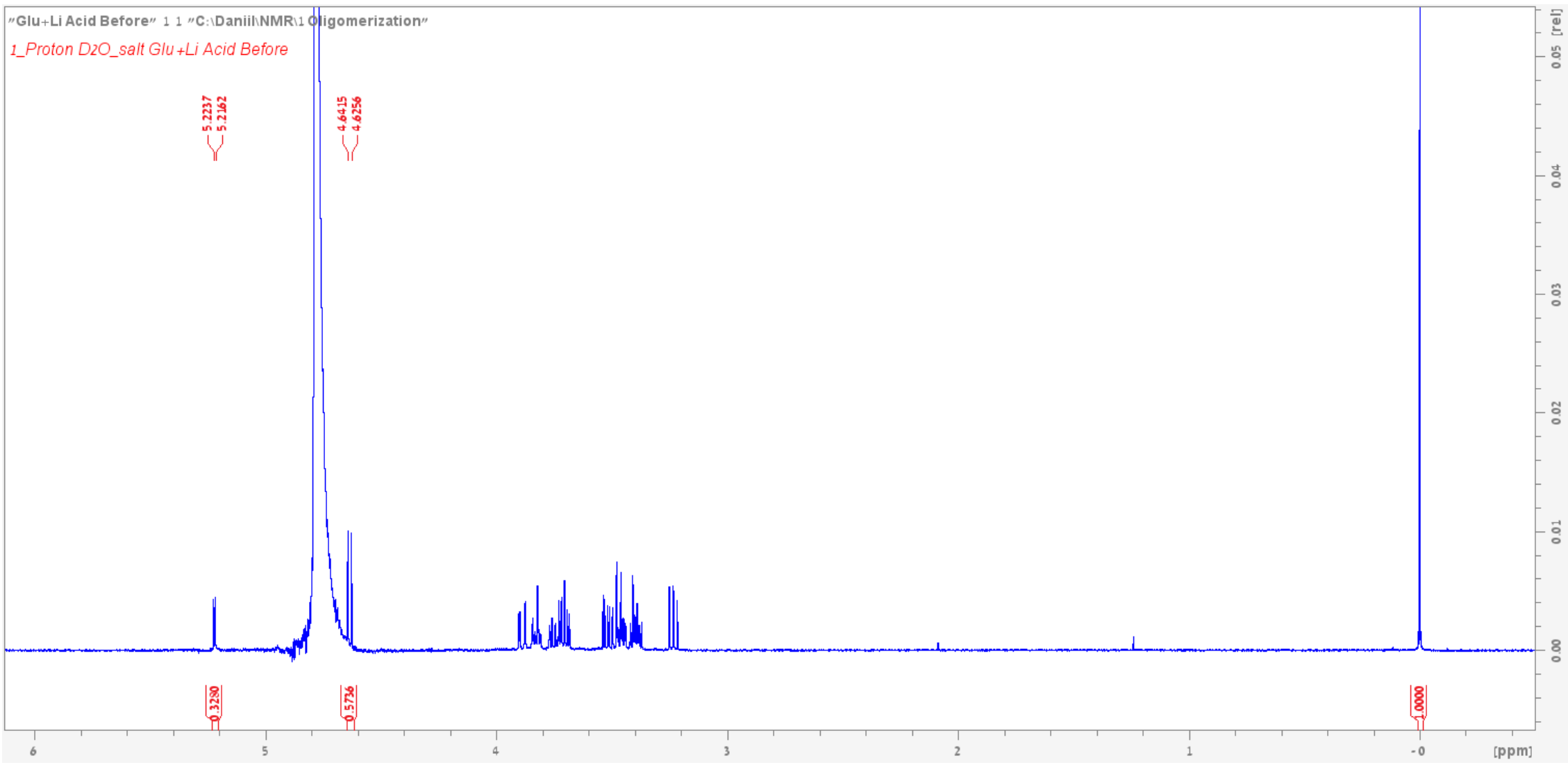

Figure 13. ^{1}**H-NMR spectra of a fresh glucose under acidified conditions in the presence of** $ZnCl_2$**.**

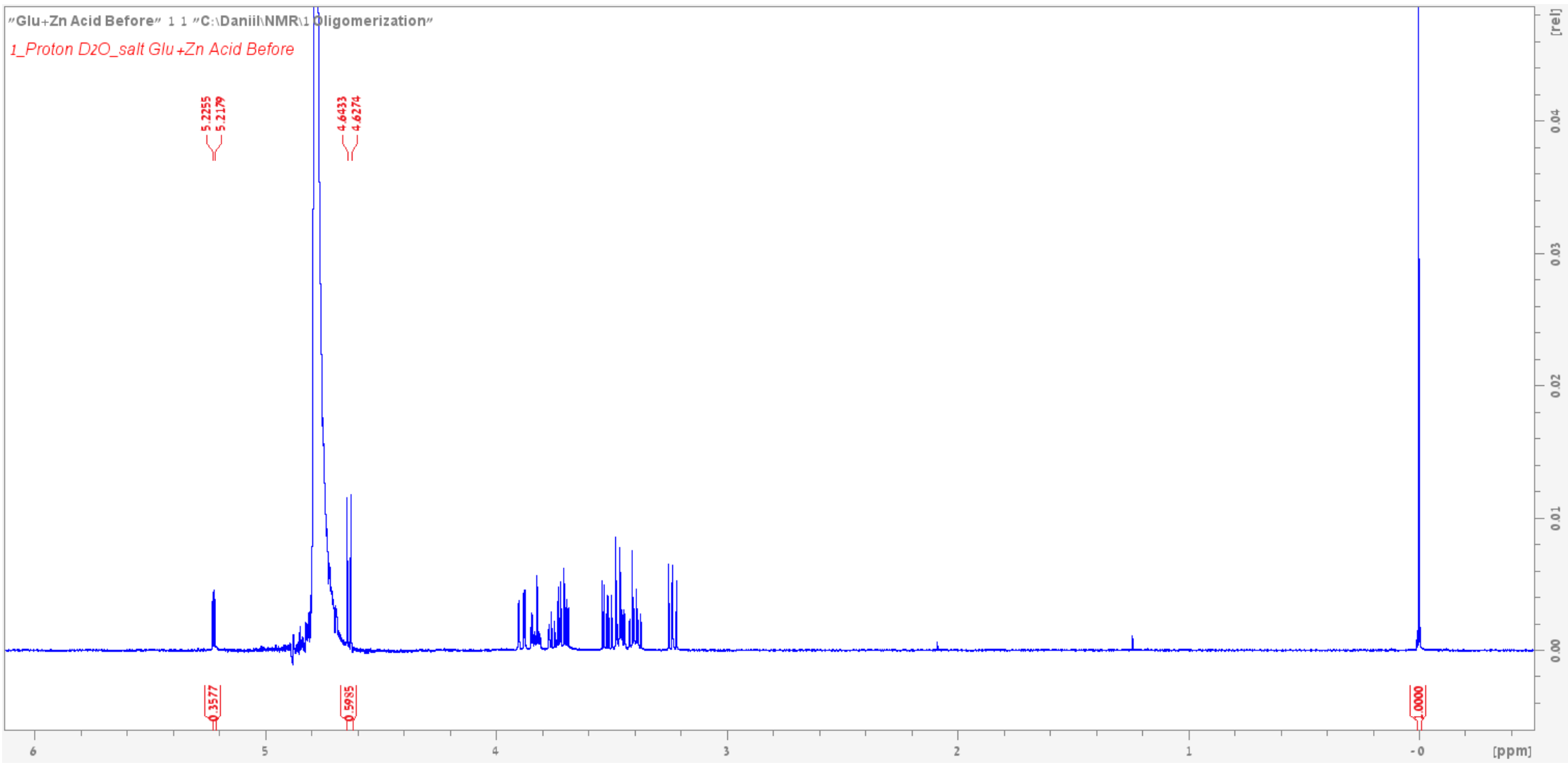

Figure 14. ^{1}**H-NMR spectra of a fresh glucose under acidified conditions in the presence of NaCl.**

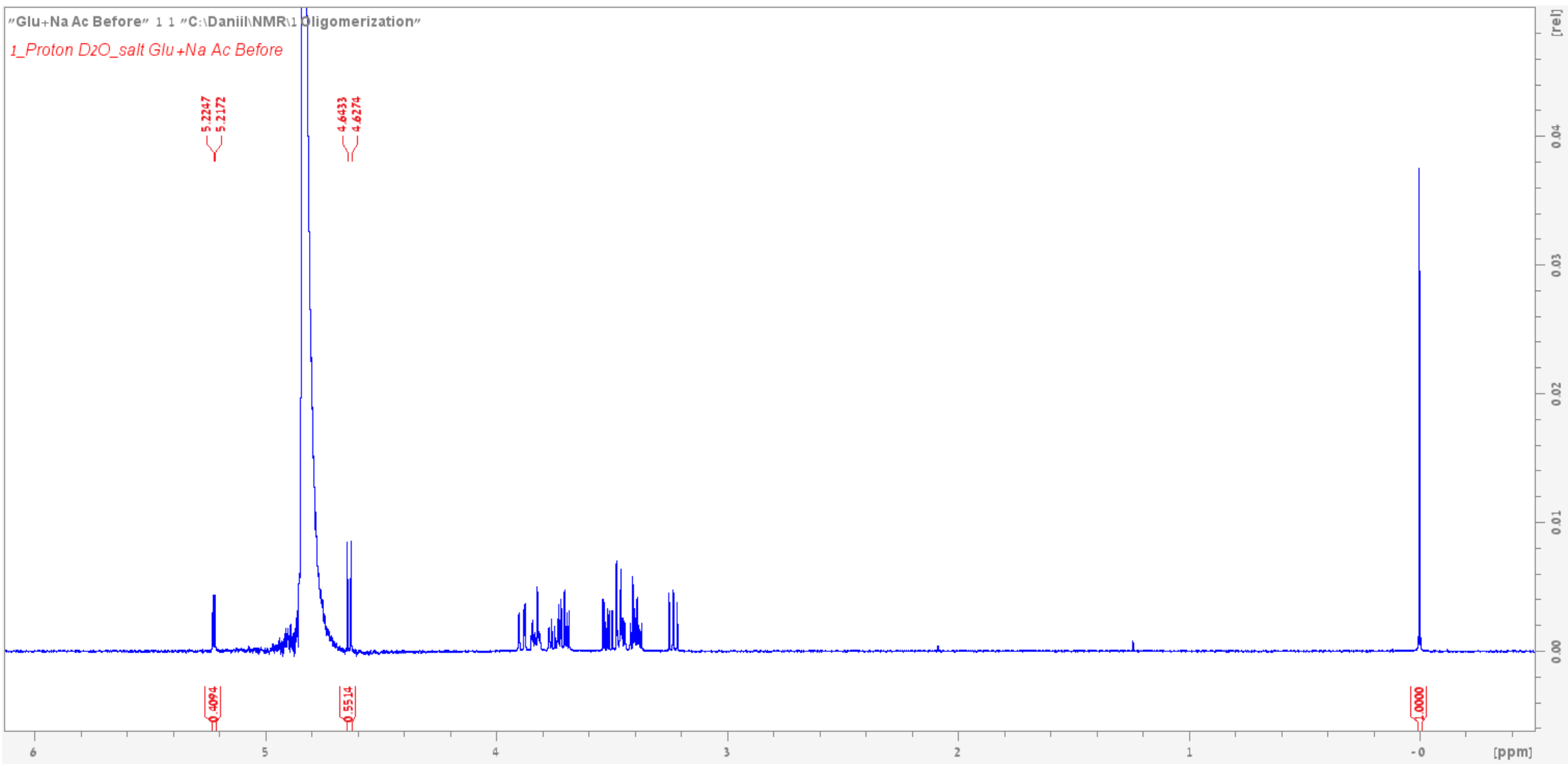

Figure 15. ^{1}H-NMR spectra of a fresh glucose under acidified conditions in the presence of KCl.

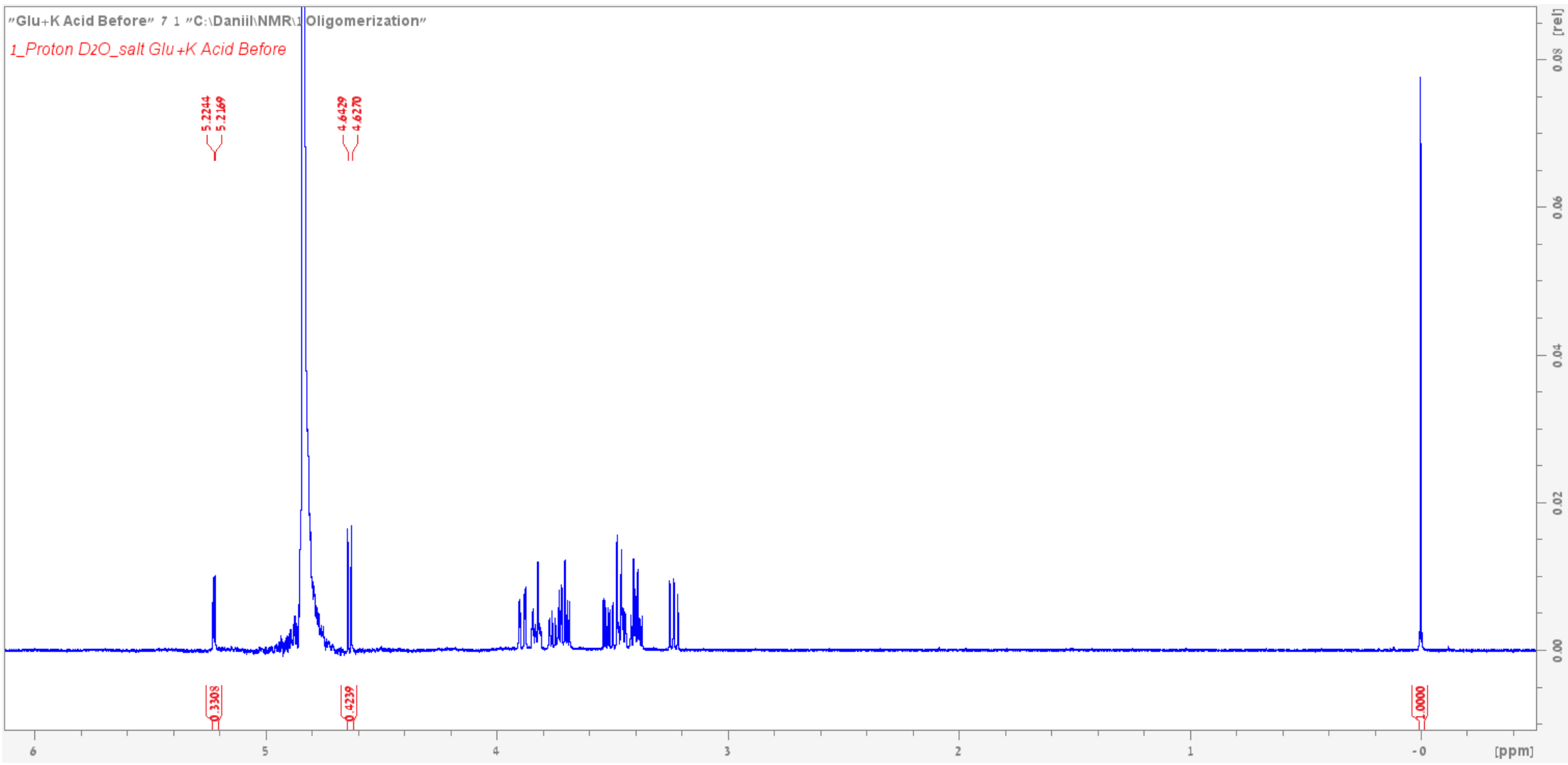

Figure 16. ^{1}H-NMR spectra of a fresh glucose under acidified conditions in the presence of $MgCl_2$.

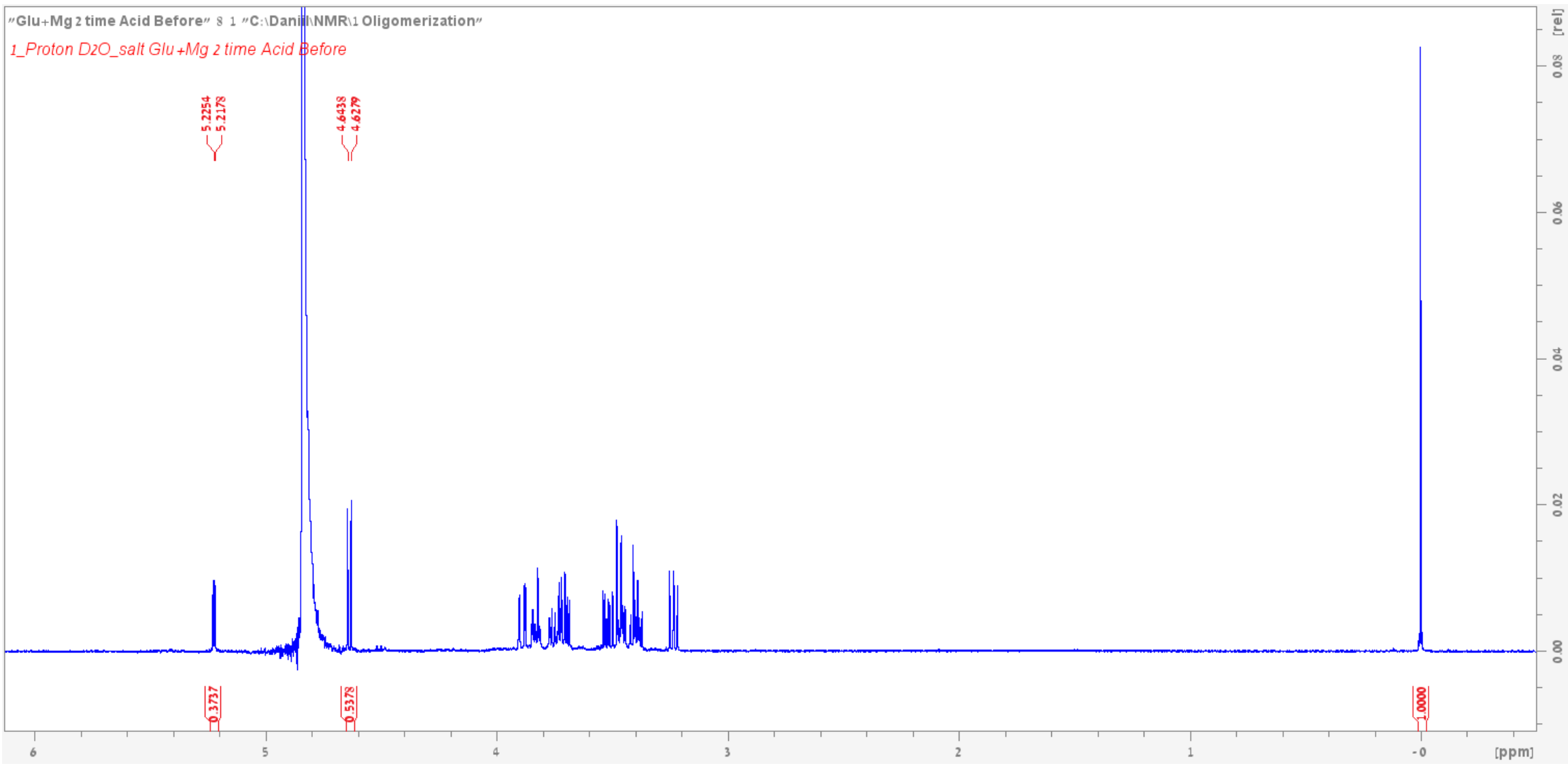

Figure 17. ^{1}H-NMR spectra of a fresh glucose under acidified conditions in the presence of $CaCl_2$.

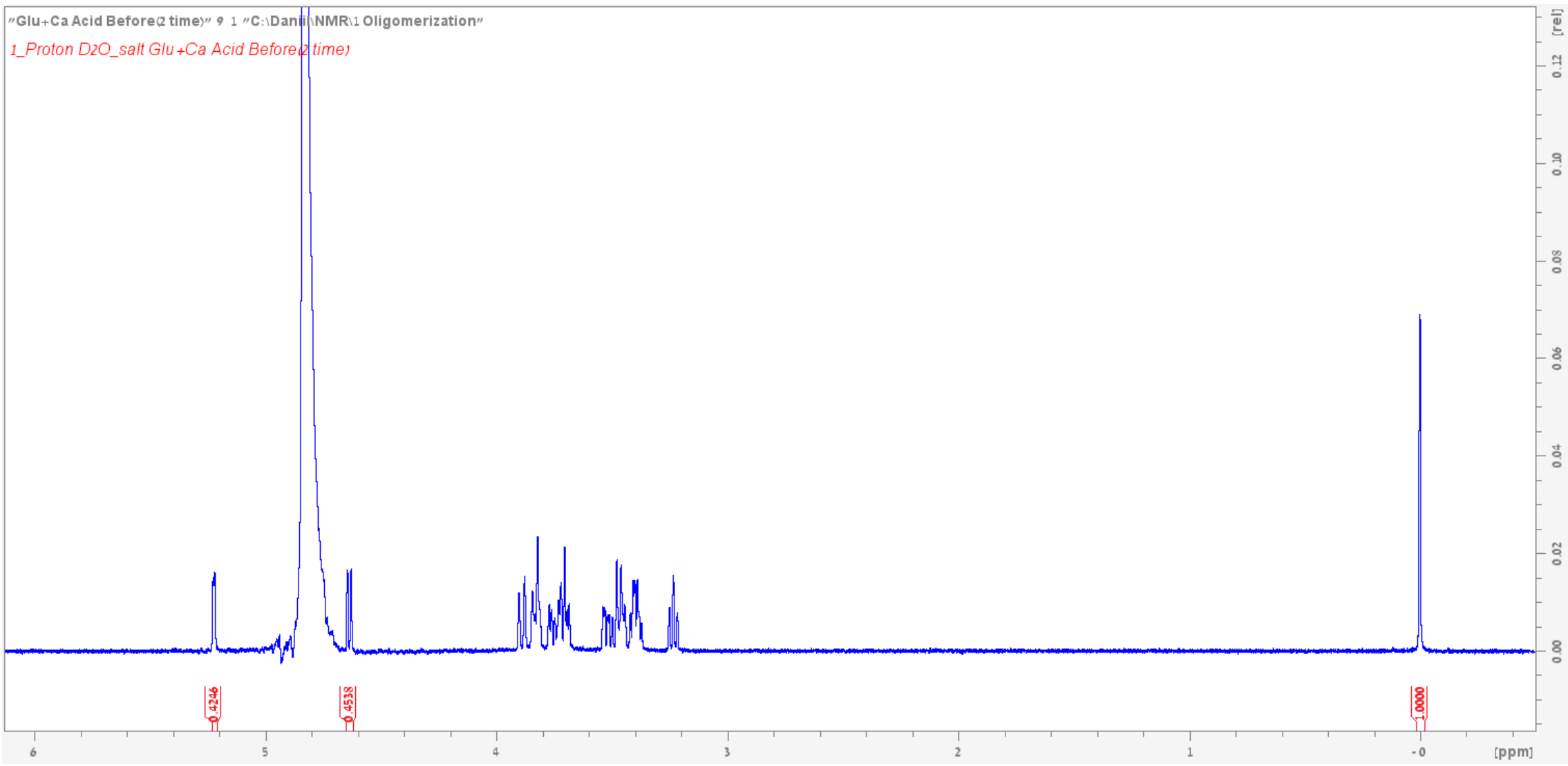

Figure 18. ^{1}H-NMR spectra of a fresh glucose under acidified conditions in the presence of $MnCl_2$.

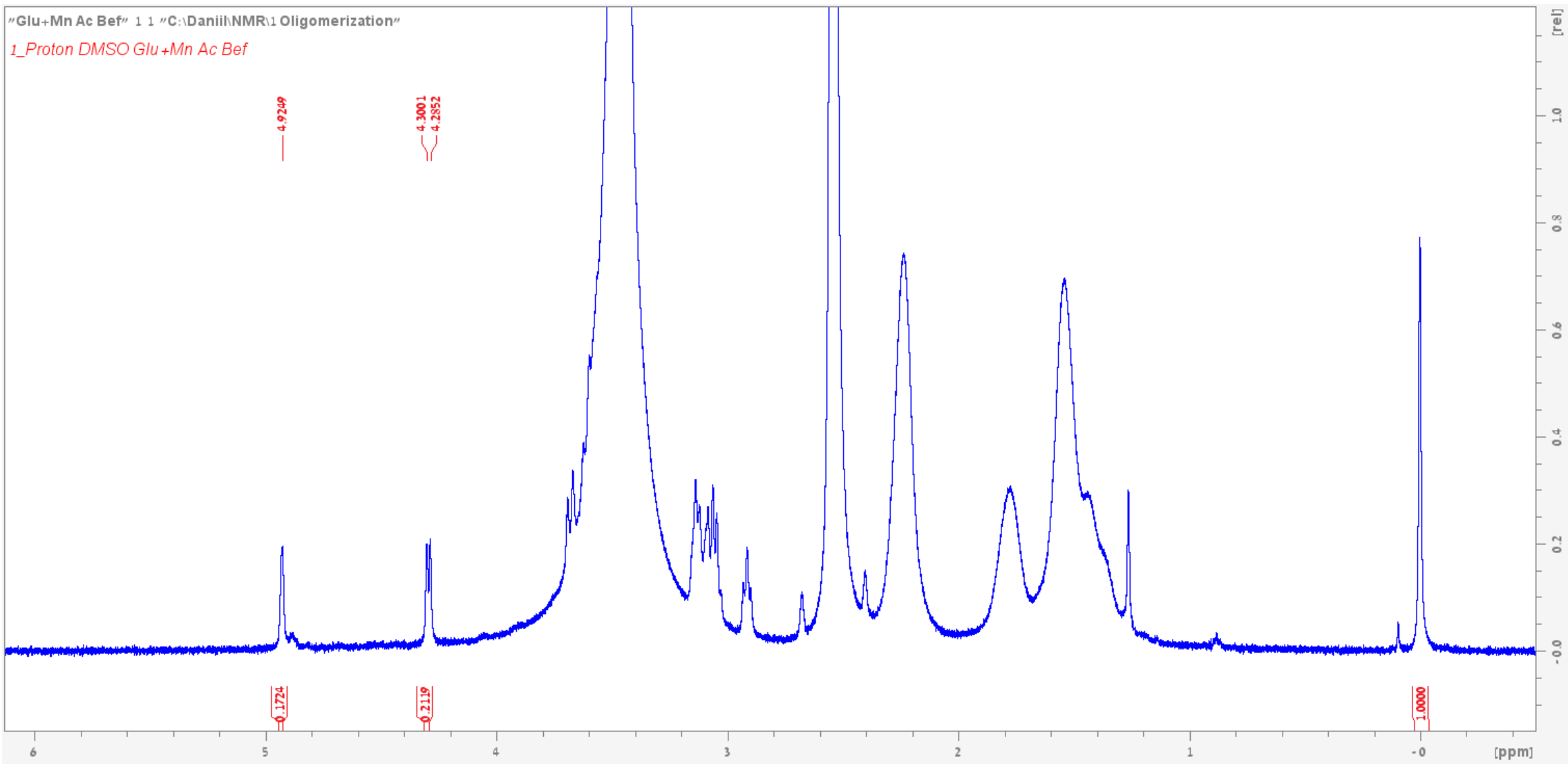

Figure 19. ^{1}**H-NMR spectra of a fresh glucose under acidified conditions in the presence of** $FeCl_2$**.**

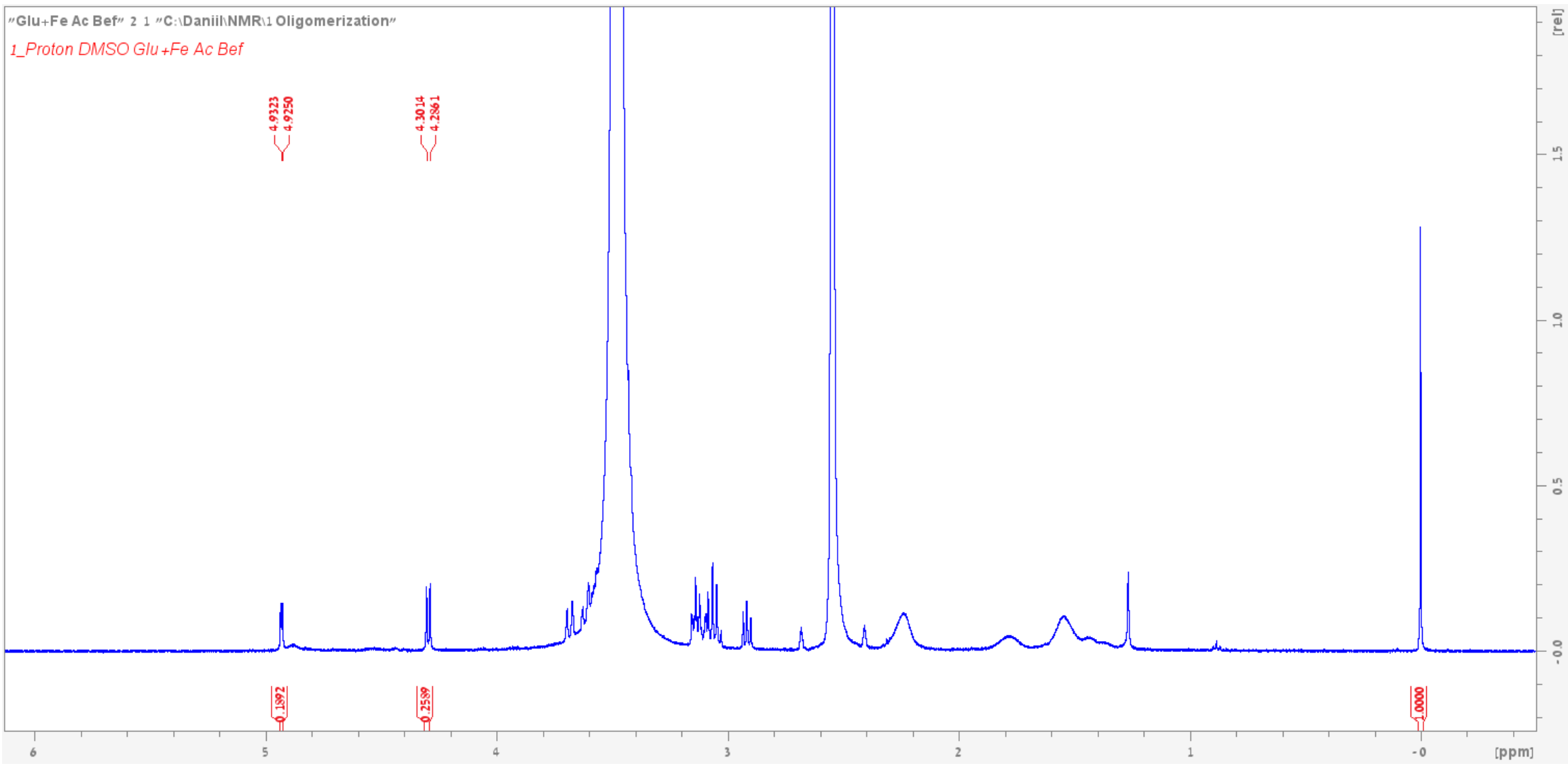

Figure 20. ^{1}**H-NMR spectra of a fresh glucose under acidified conditions in the presence of** $AlCl_3$**.**

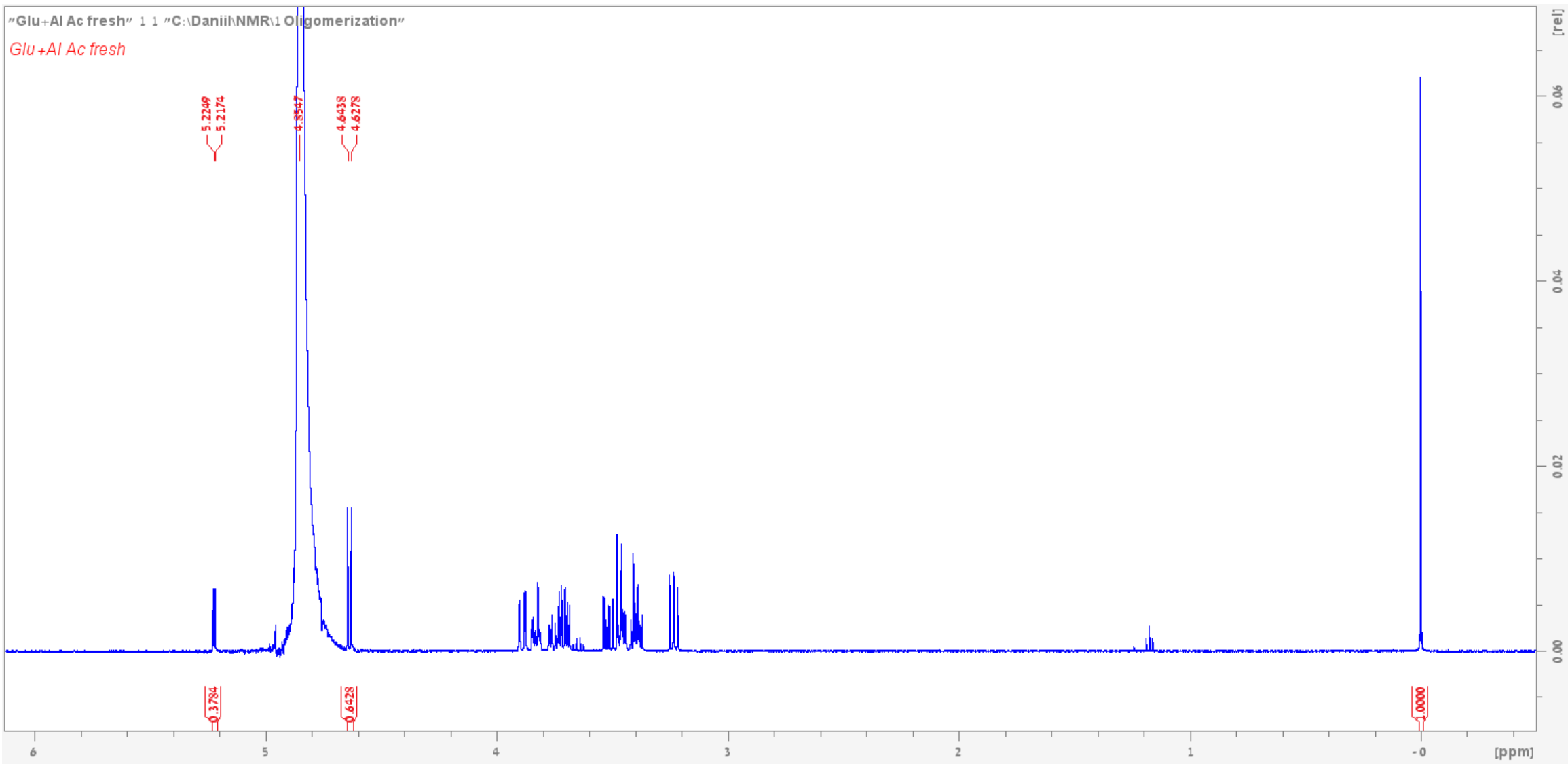

Figure 21. ¹H-NMR spectra of three independent dry-down reactions of glucose in the absence of metal ions upon addition of NaOH. Glucose was dried at 50 ˚C for seven days in three replicates:

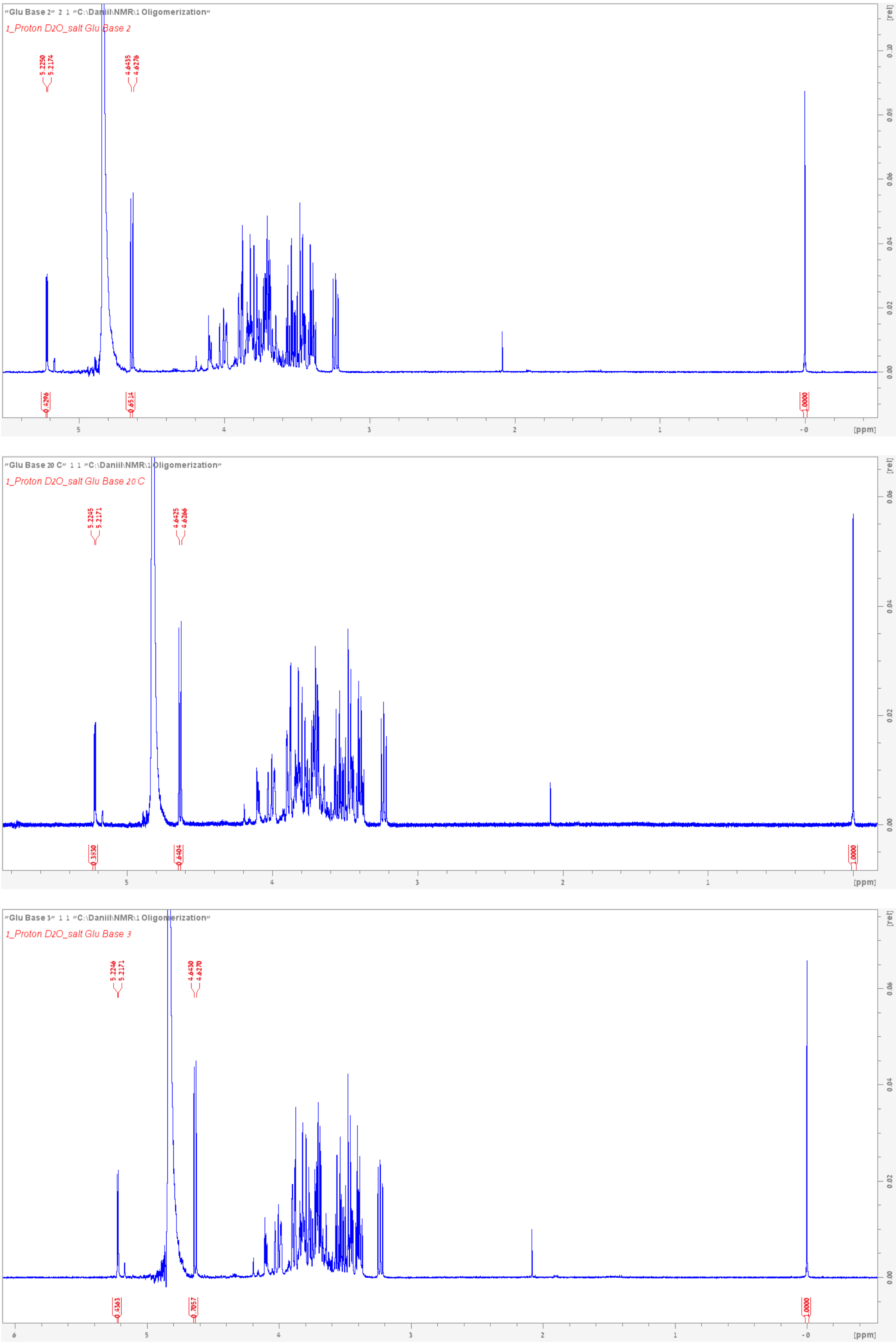

Figure 22. ^{1}H-NMR spectra of three independent dry-down reactions of glucose and LiCl upon addition of NaOH. Glucose was dried at 50 ˚C for seven days in three replicates:

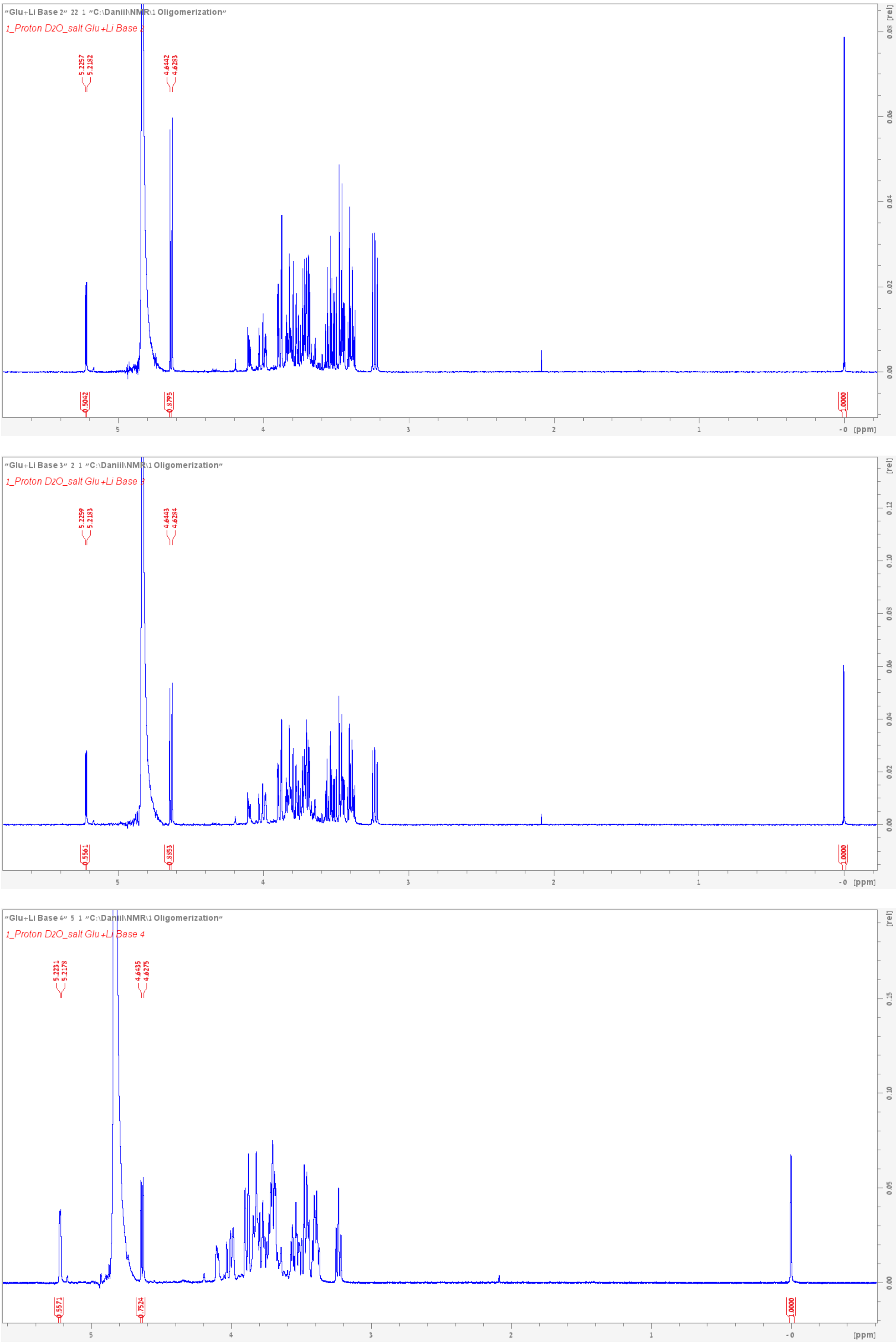

Figure 23. ^{1}H-NMR spectra of three independent dry-down reactions of glucose and $ZnCl_2$ upon addition of NaOH. Glucose was dried at 50 ˚C for seven days in three replicates:

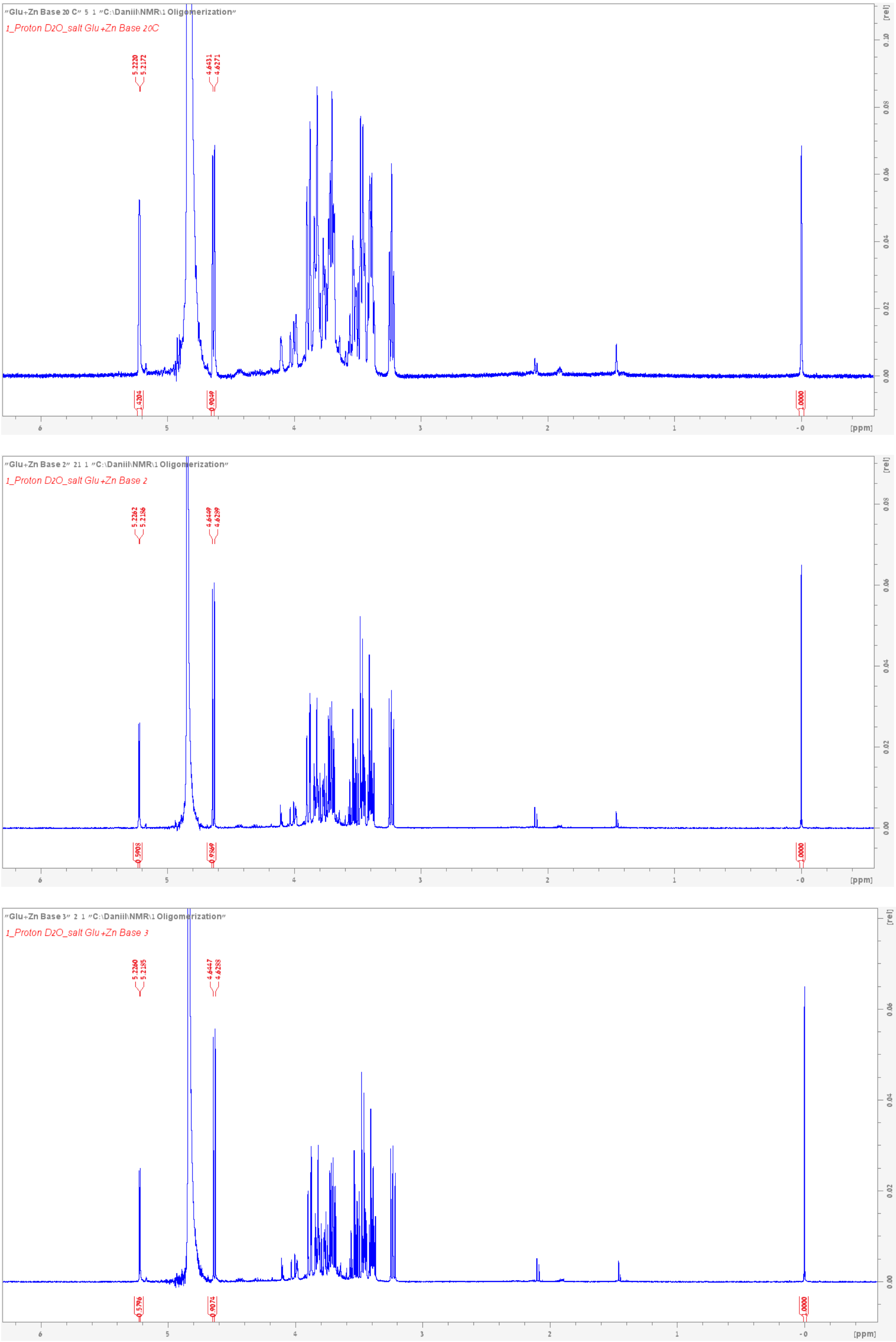

Figure 24. ^{1}H-NMR spectra of three independent dry-down reactions of glucose and NaCl upon addition of NaOH. Glucose was dried at 50 ˚C for seven days in three replicates:

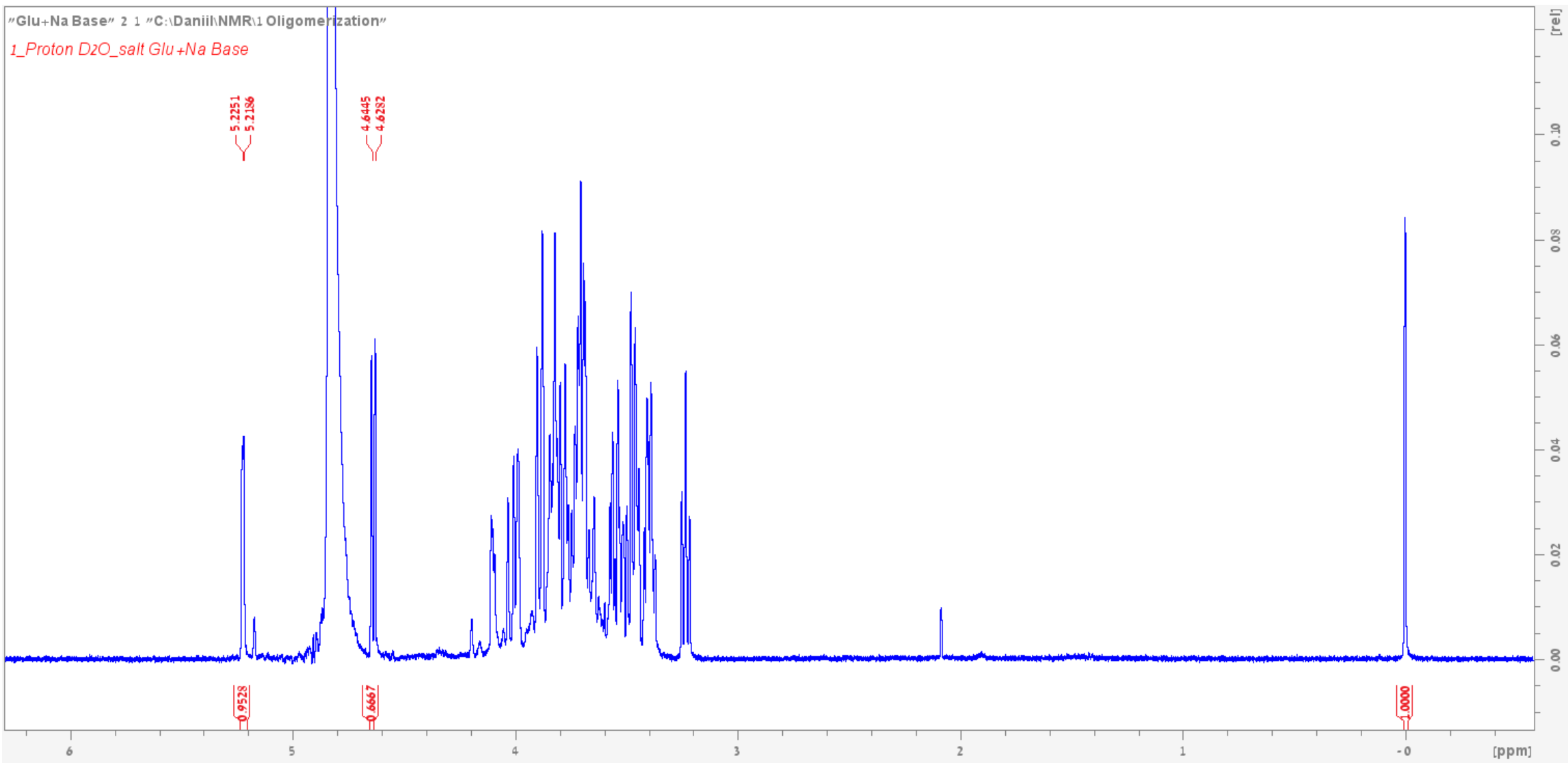

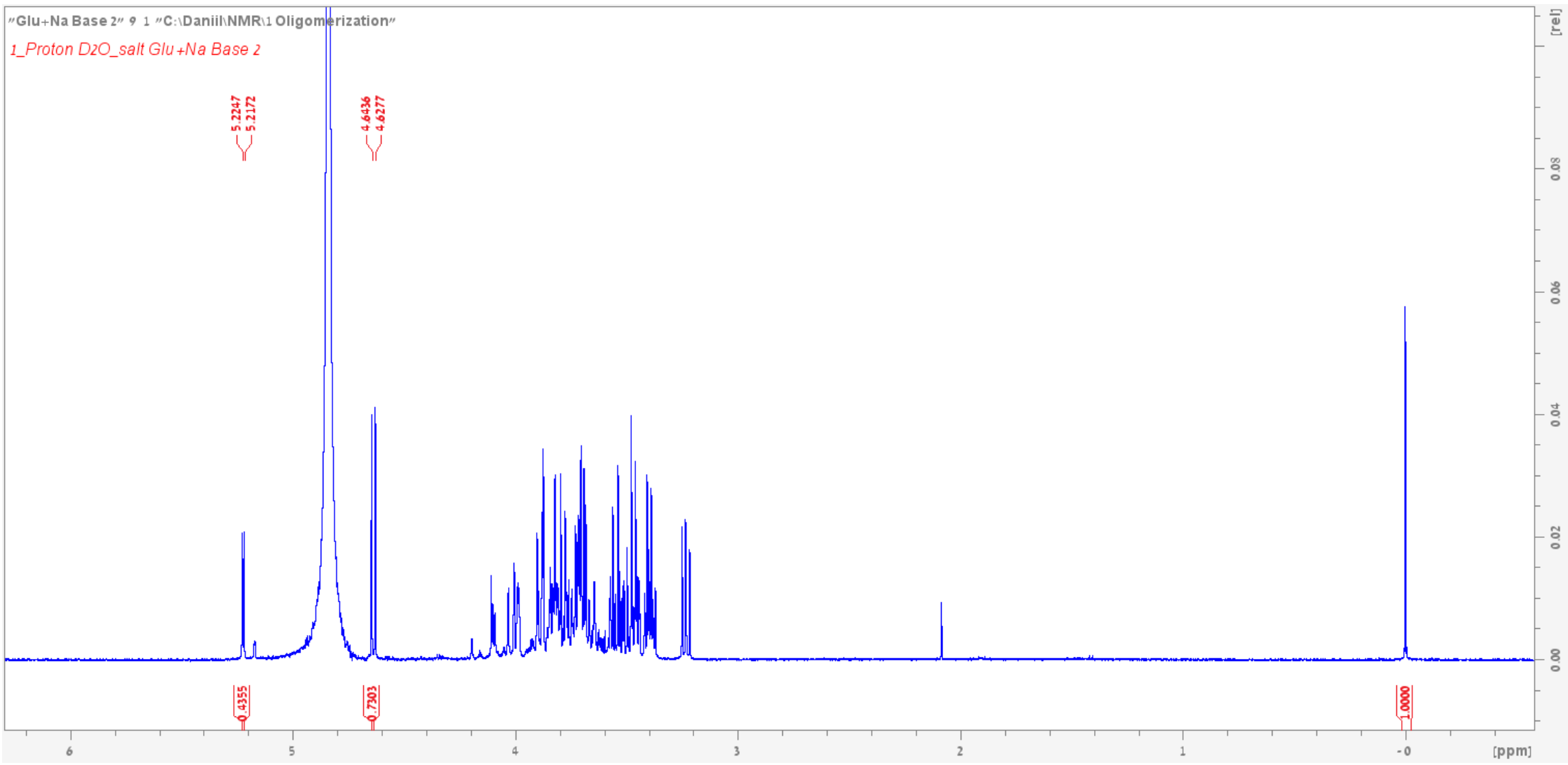

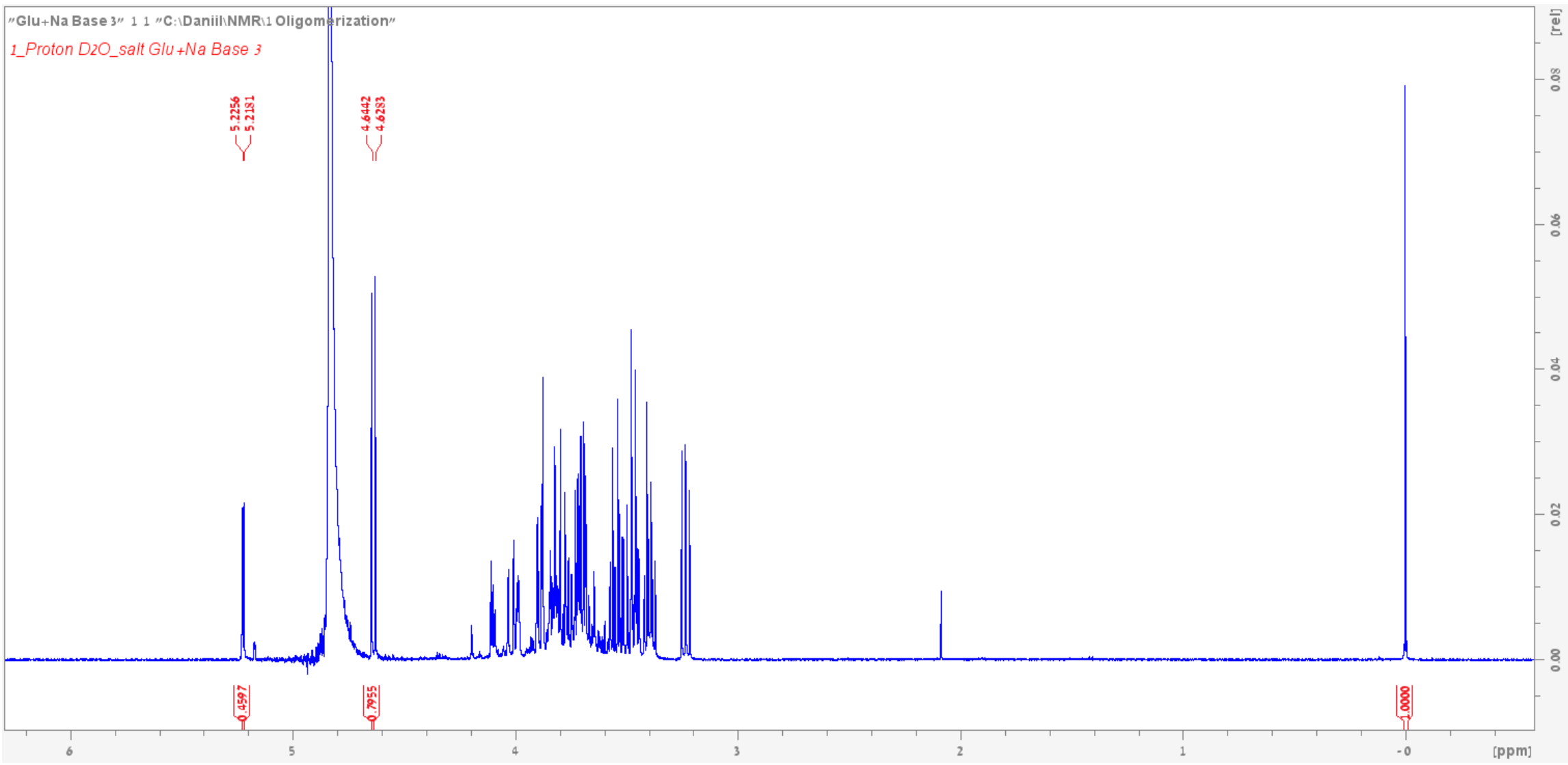

Figure 25. ^{1}H-NMR spectra of three independent dry-down reactions of glucose and KCl upon addition of NaOH. Glucose was dried at 50 ˚C for seven days in three replicates:

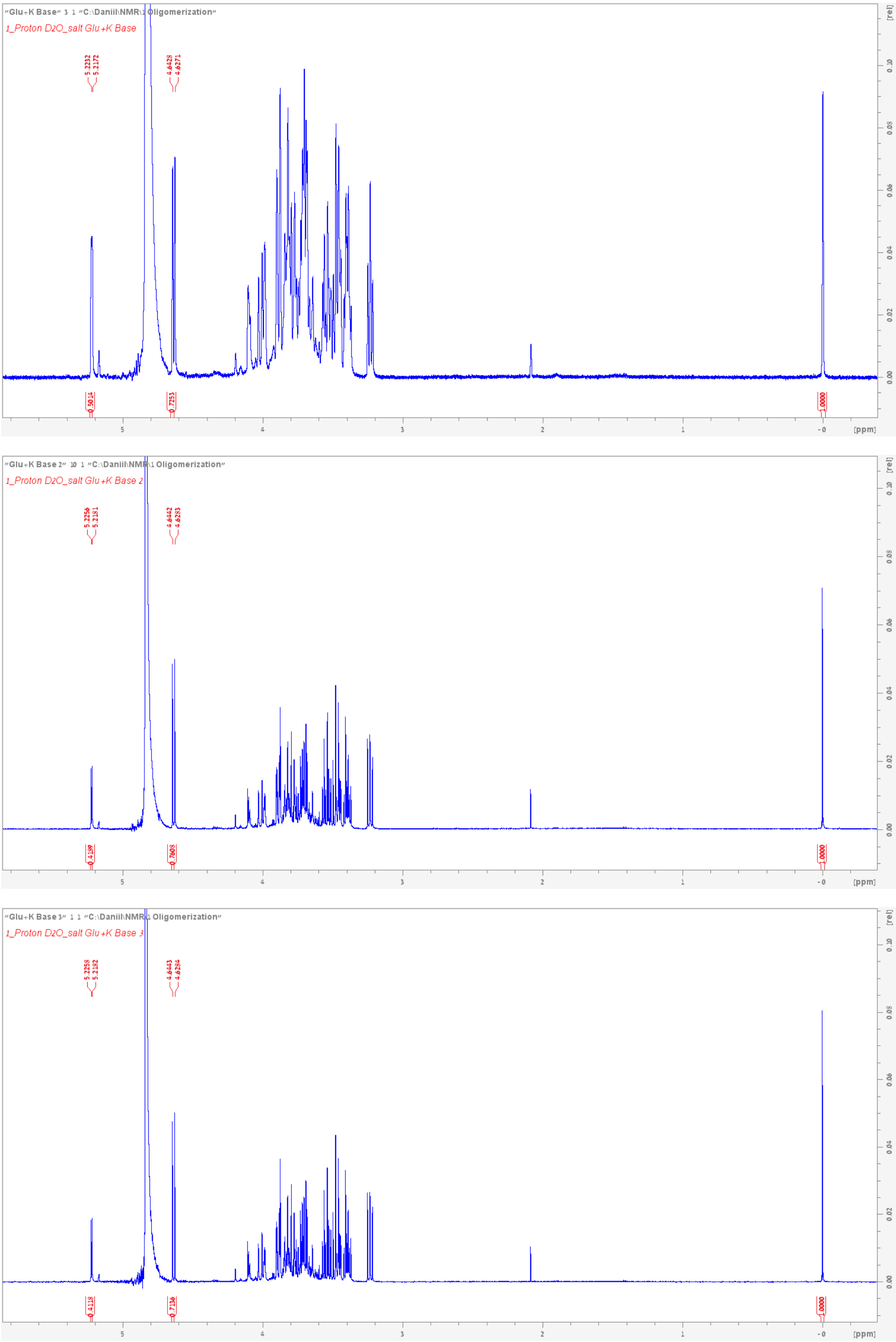

Figure 26. ^{1}H-NMR spectra of three independent dry-down reactions of glucose and $MgCl_2$ upon addition of NaOH. Glucose was dried at 50 ˚C for seven days in three replicates:

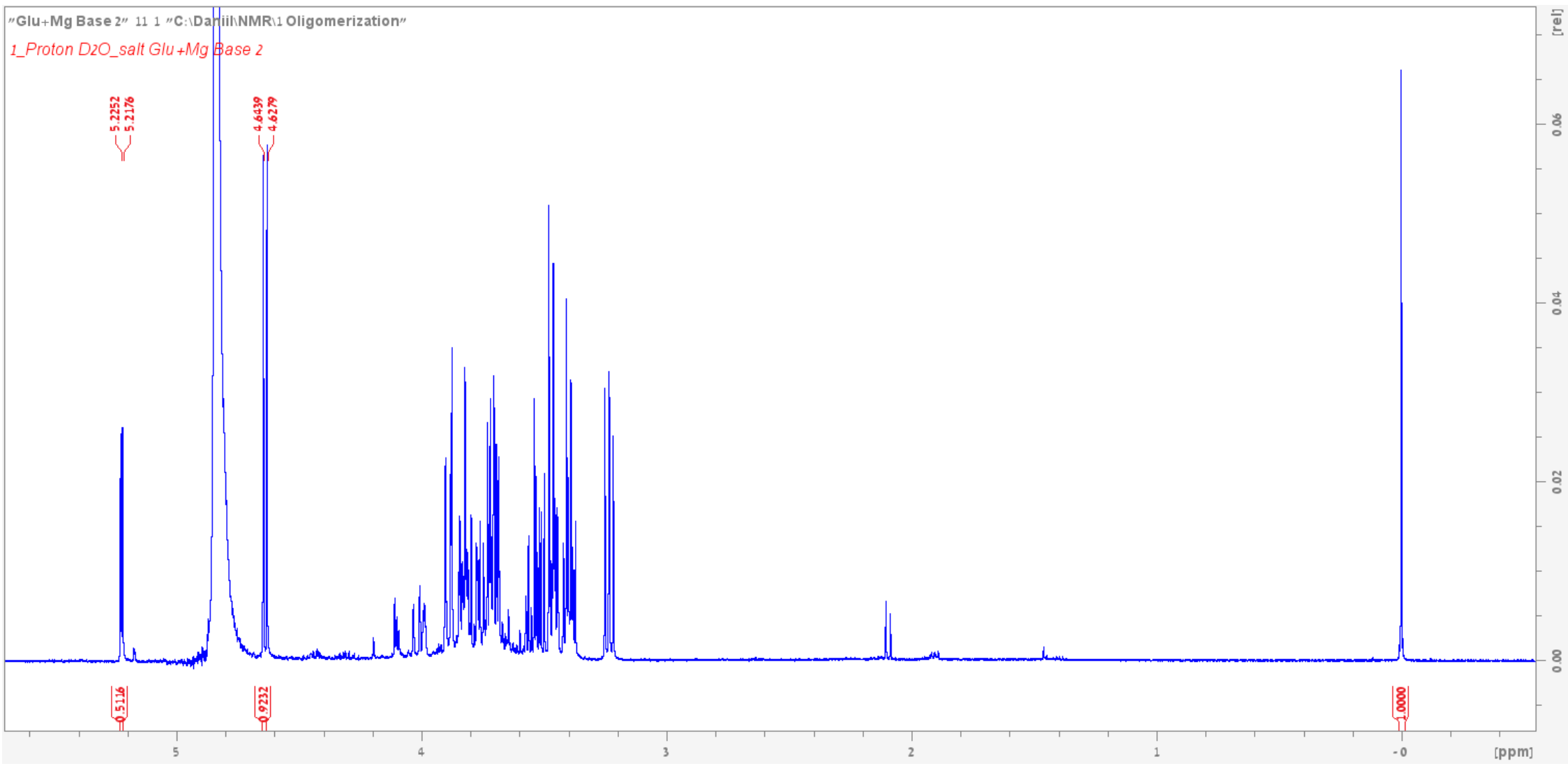

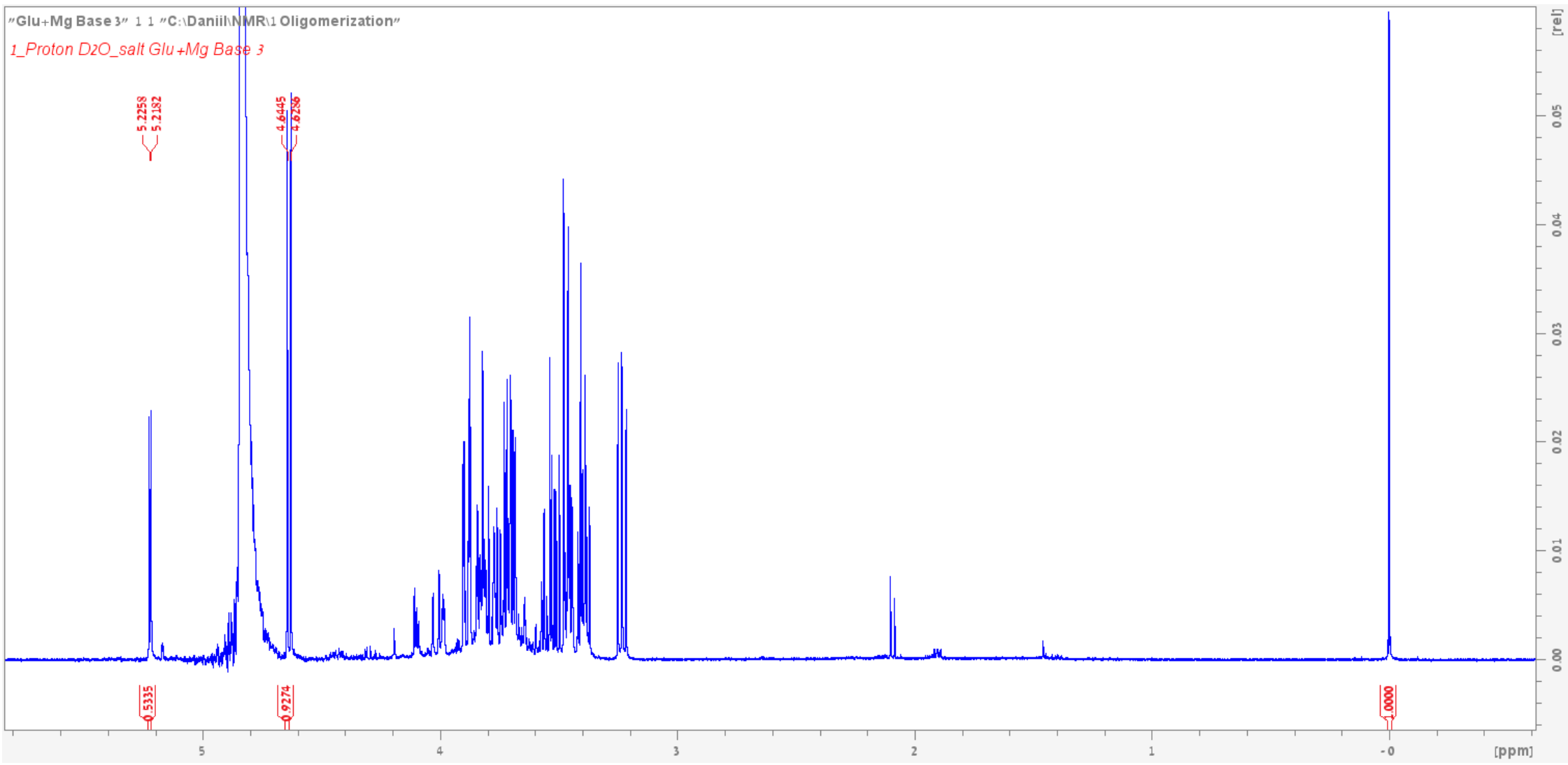

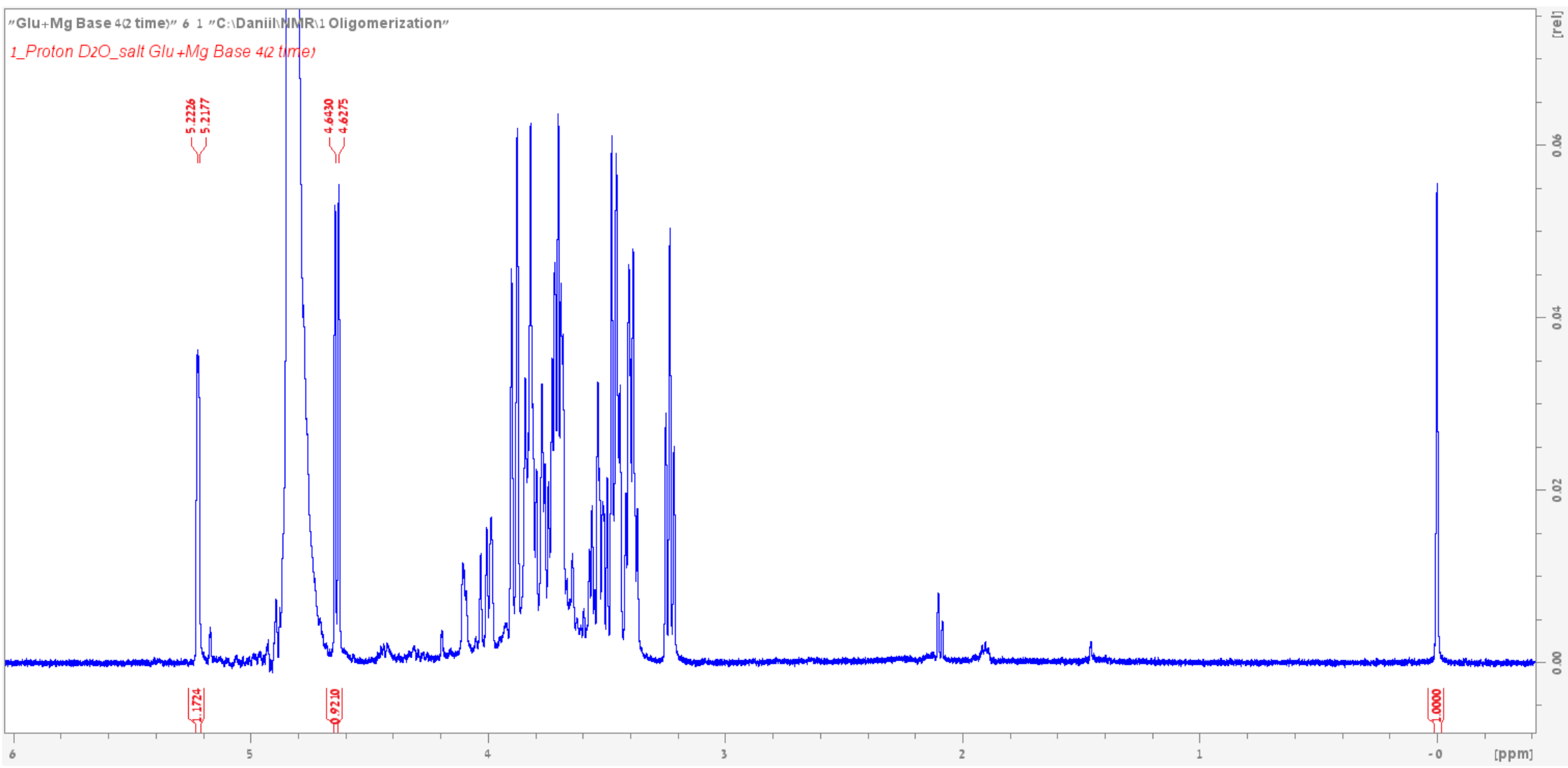

Figure 27. 1H-NMR spectra of three independent dry-down reactions of glucose and $CaCl_2$ upon addition of NaOH. Glucose was dried at 50 °C for seven days in three replicates:

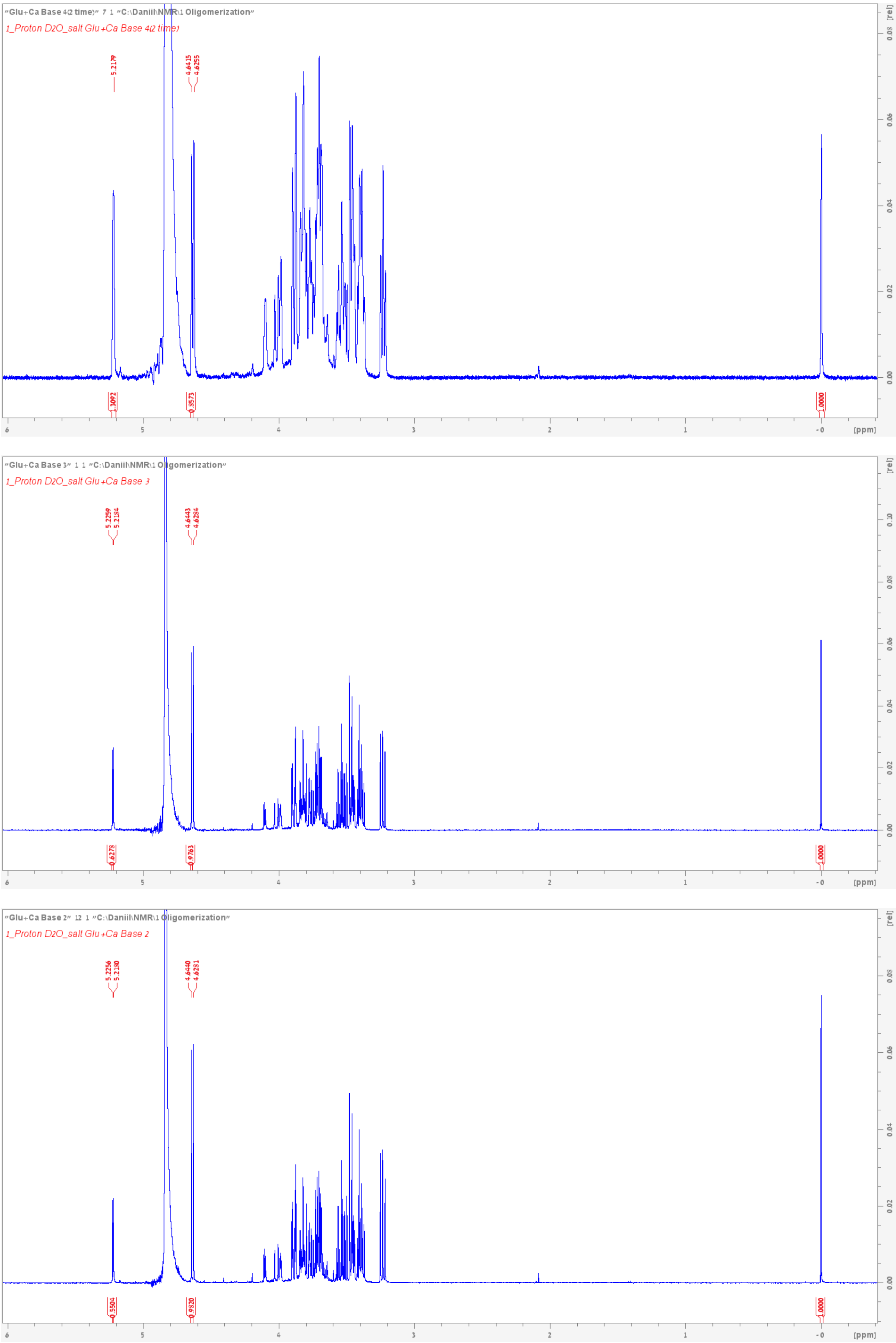

Figure 28. ^{1}H-NMR spectra of three independent dry-down reactions of glucose and $MnCl_2$ upon addition of NaOH. Glucose was dried at 50 ˚C for seven days in three replicates:

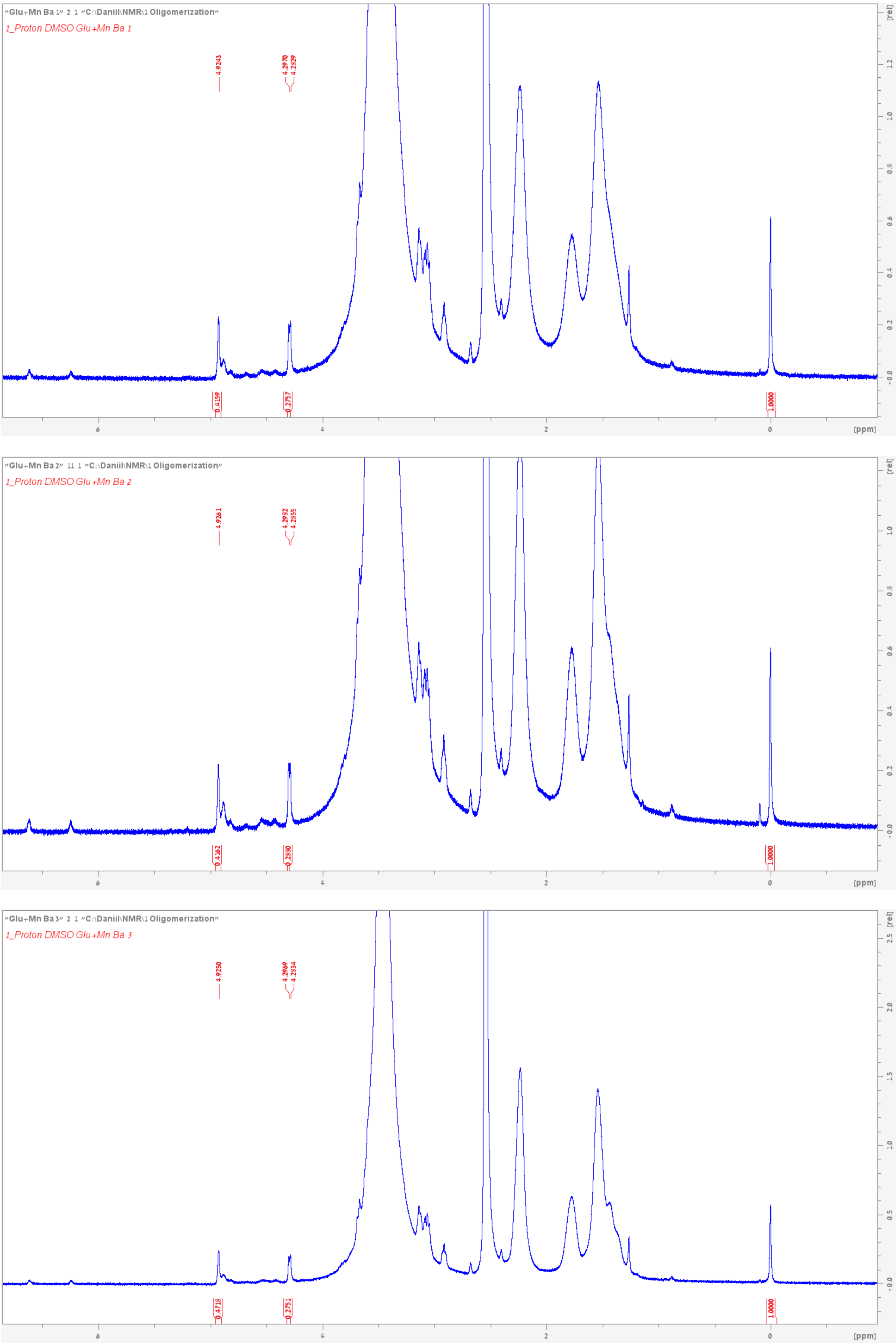

Figure 29. ^{1}H-NMR spectra of three independent dry-down reactions of glucose and $FeCl_2$ upon addition of NaOH. Glucose was dried at 50 ˚C for seven days in three replicates:

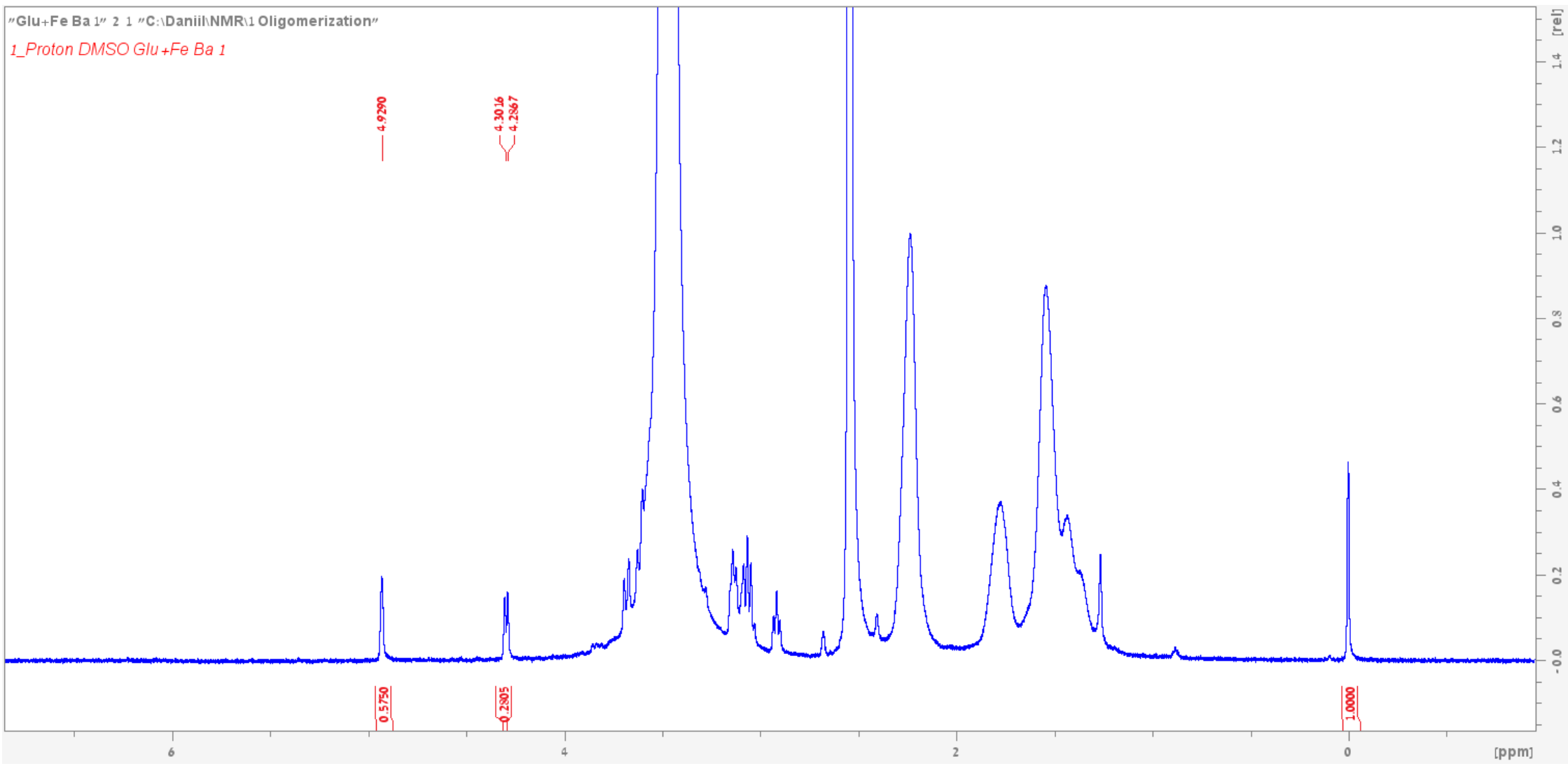

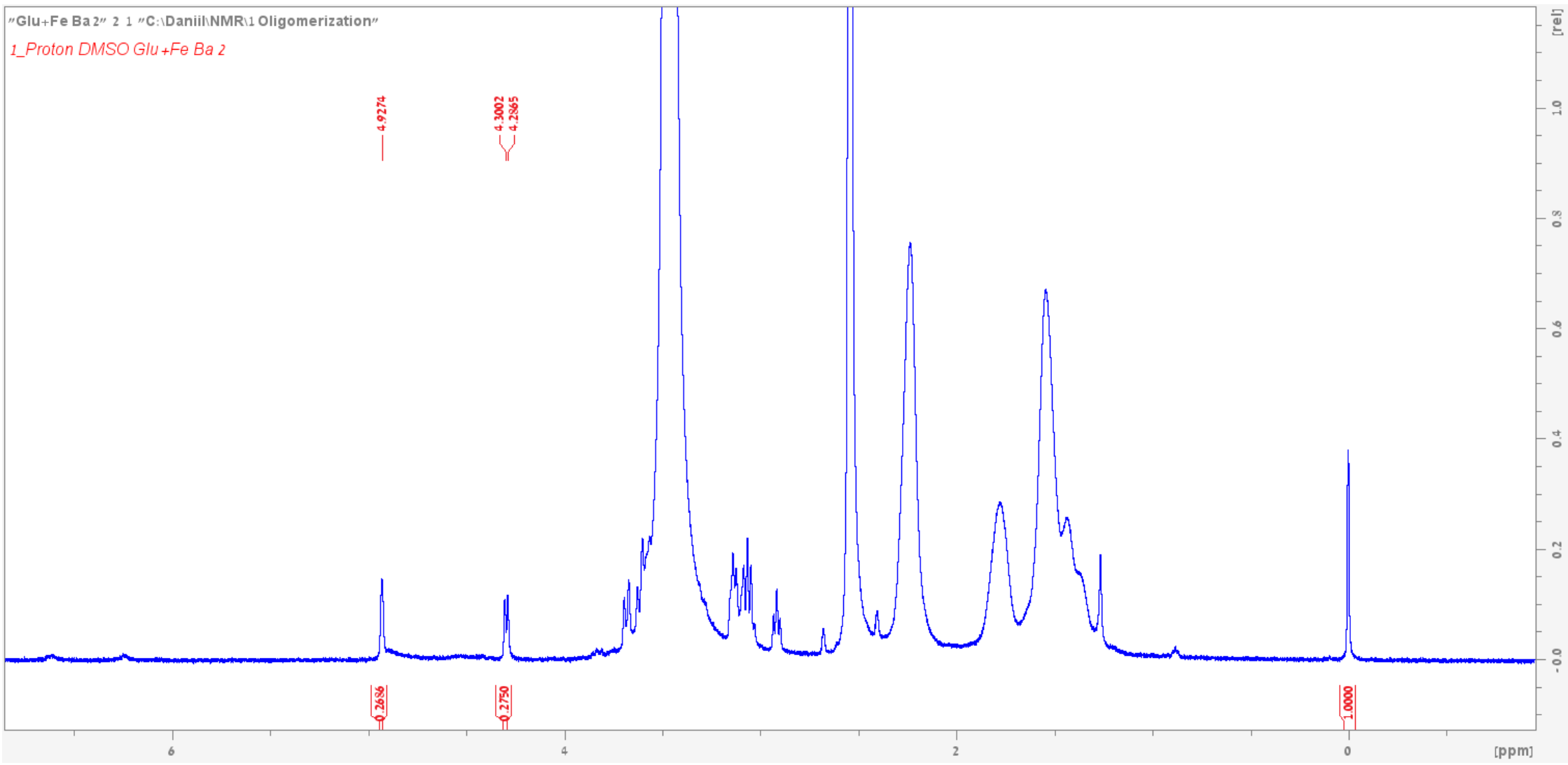

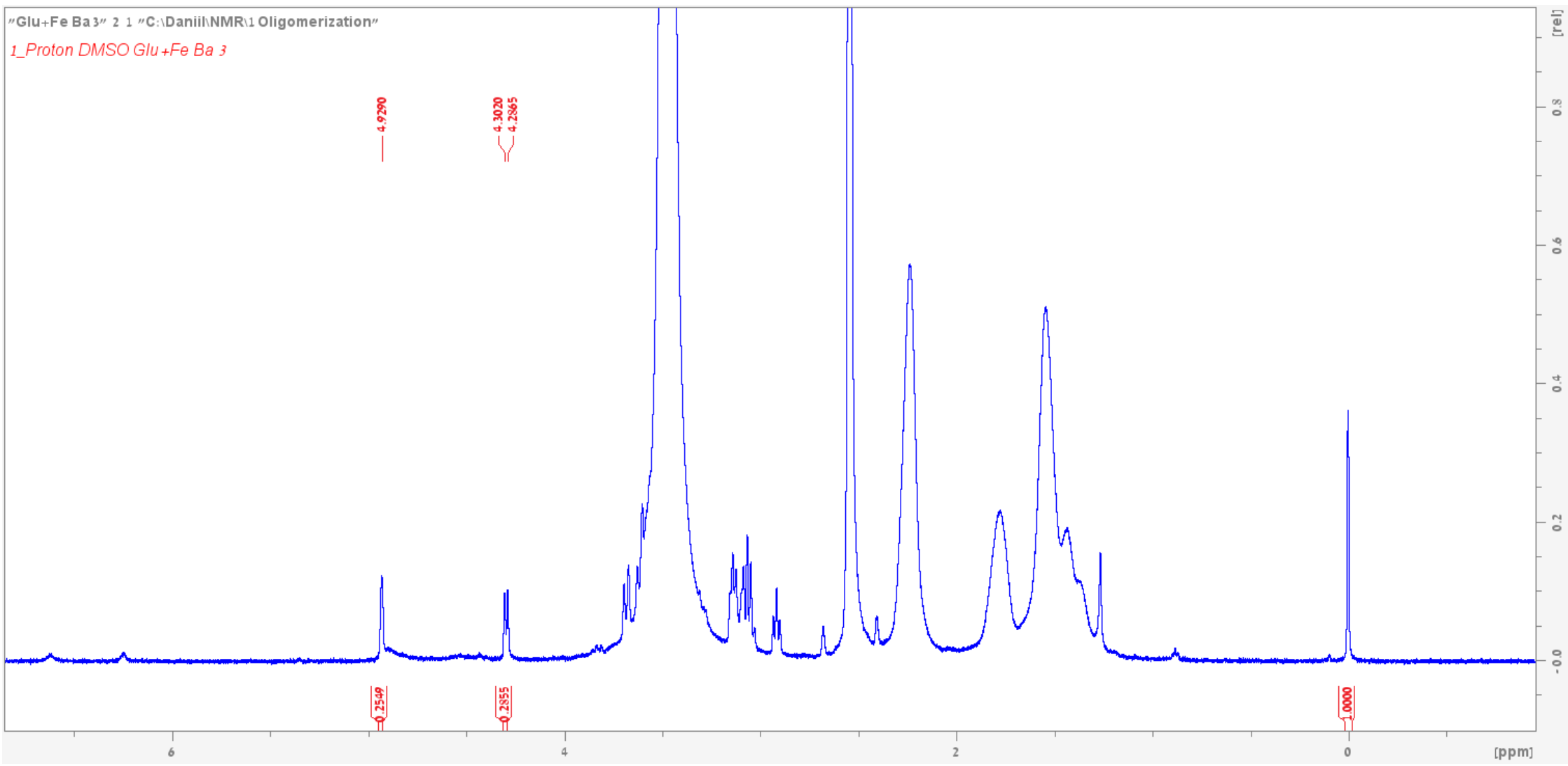

Figure 30. ^{1}H-NMR spectra of three independent dry-down reactions of glucose and $AlCl_3$ upon addition of NaOH. Glucose was dried at 50 ˚C for seven days in three replicates:

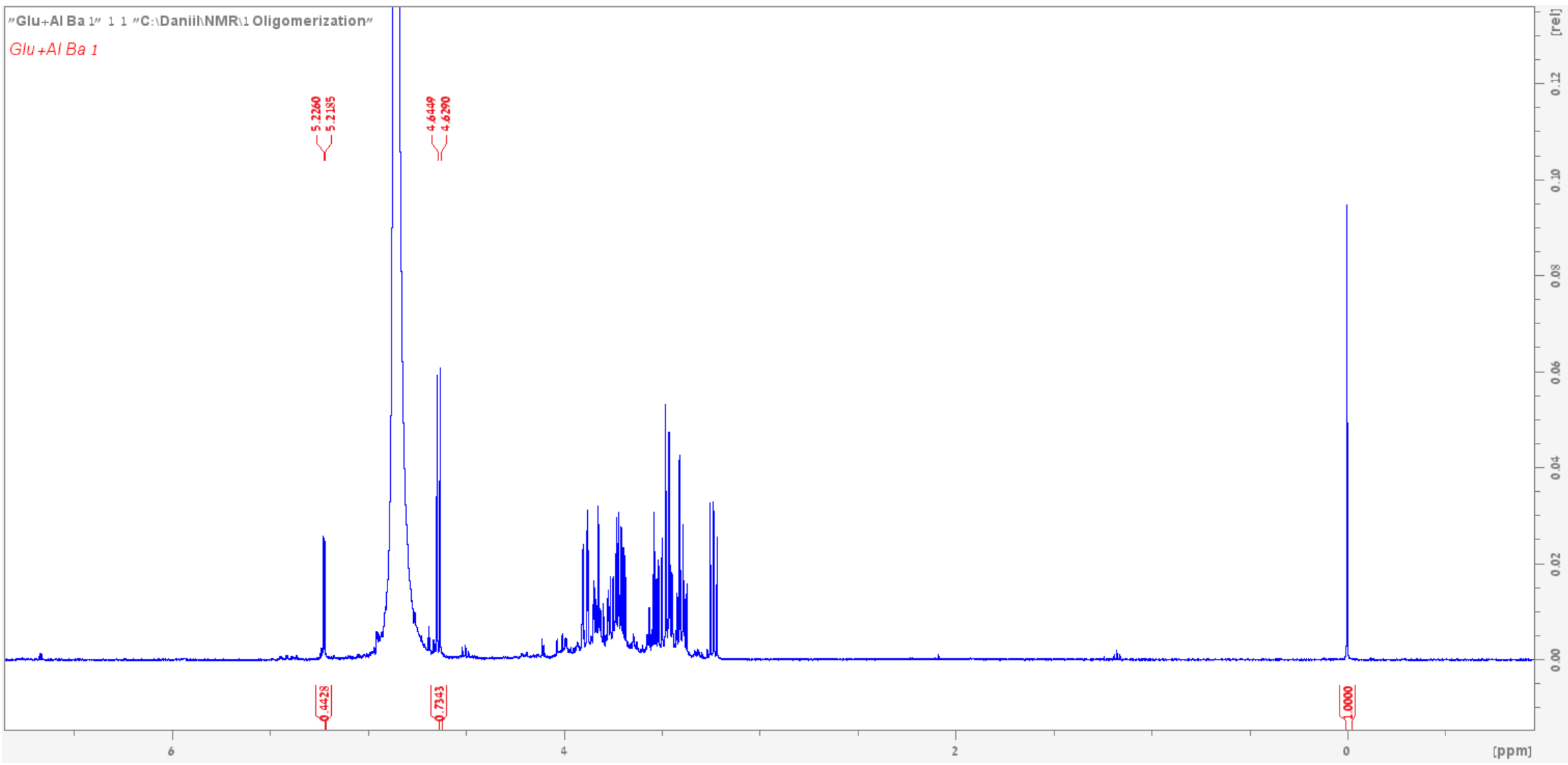

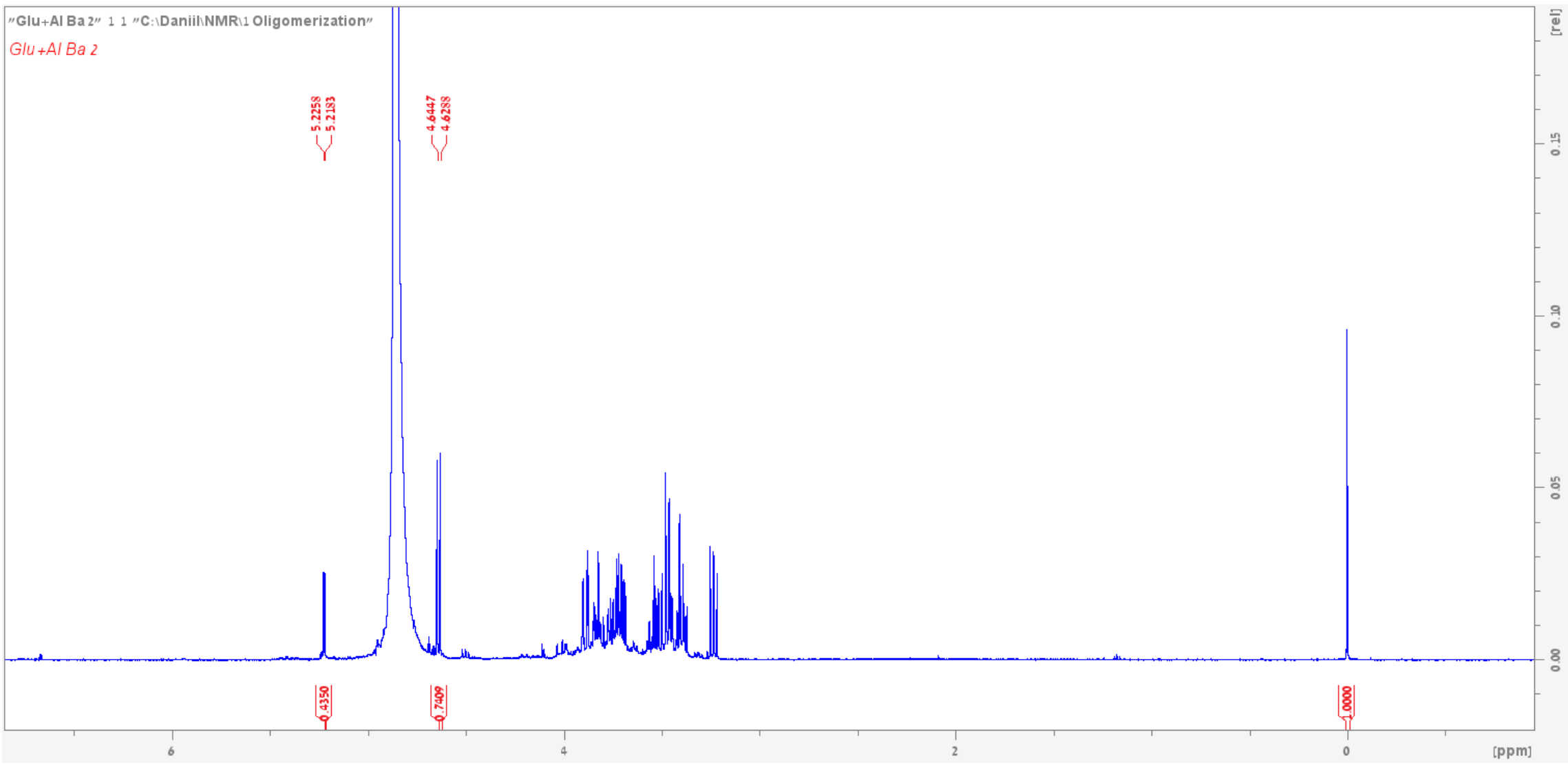

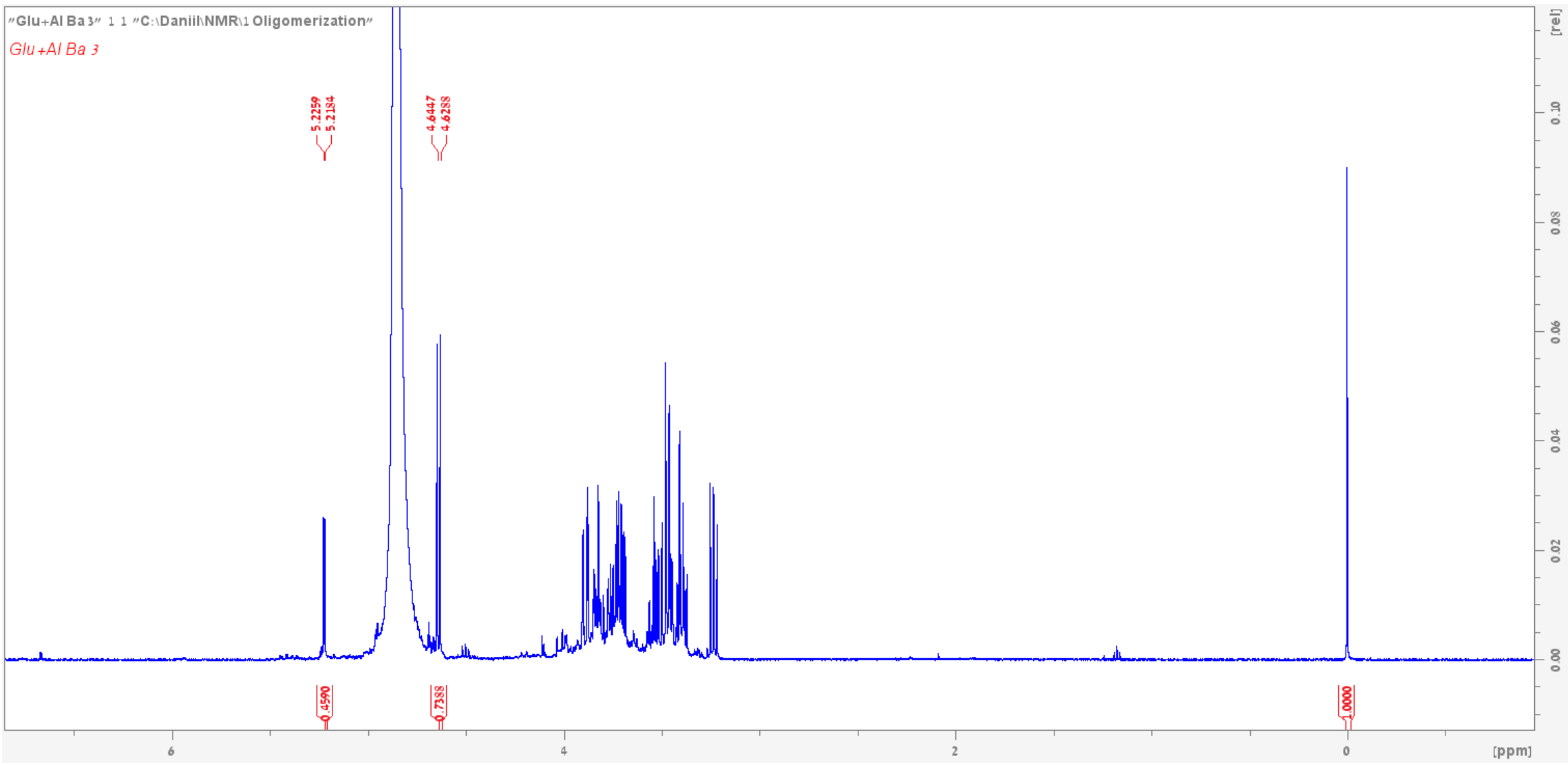

Figure 31. ^{1}H-NMR spectra of a fresh glucose upon addition of NaOH in the absence of metals.

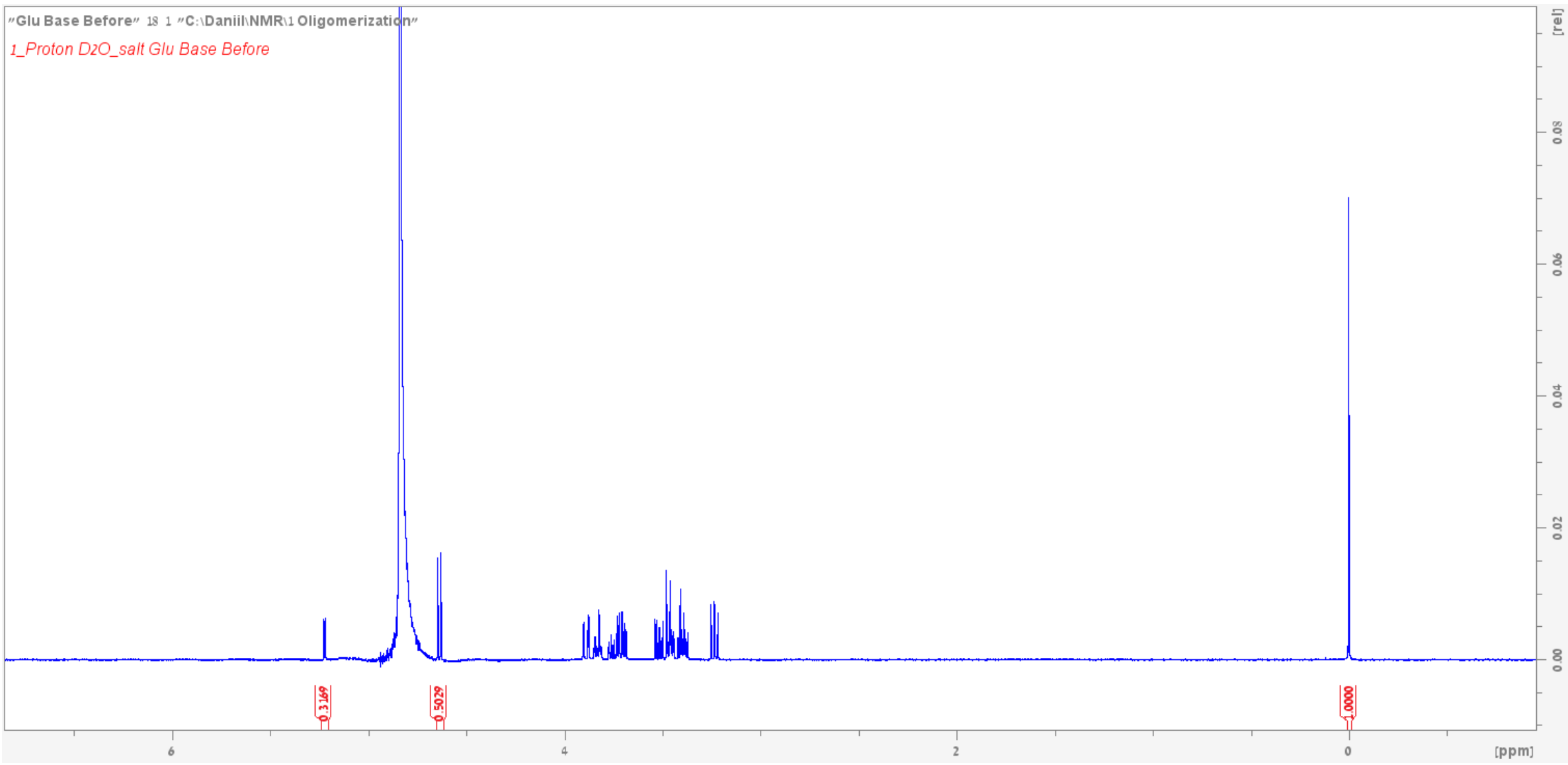

Figure 32. ^{1}H-NMR spectra of a fresh glucose upon addition of NaOH in the presence of LiCl.

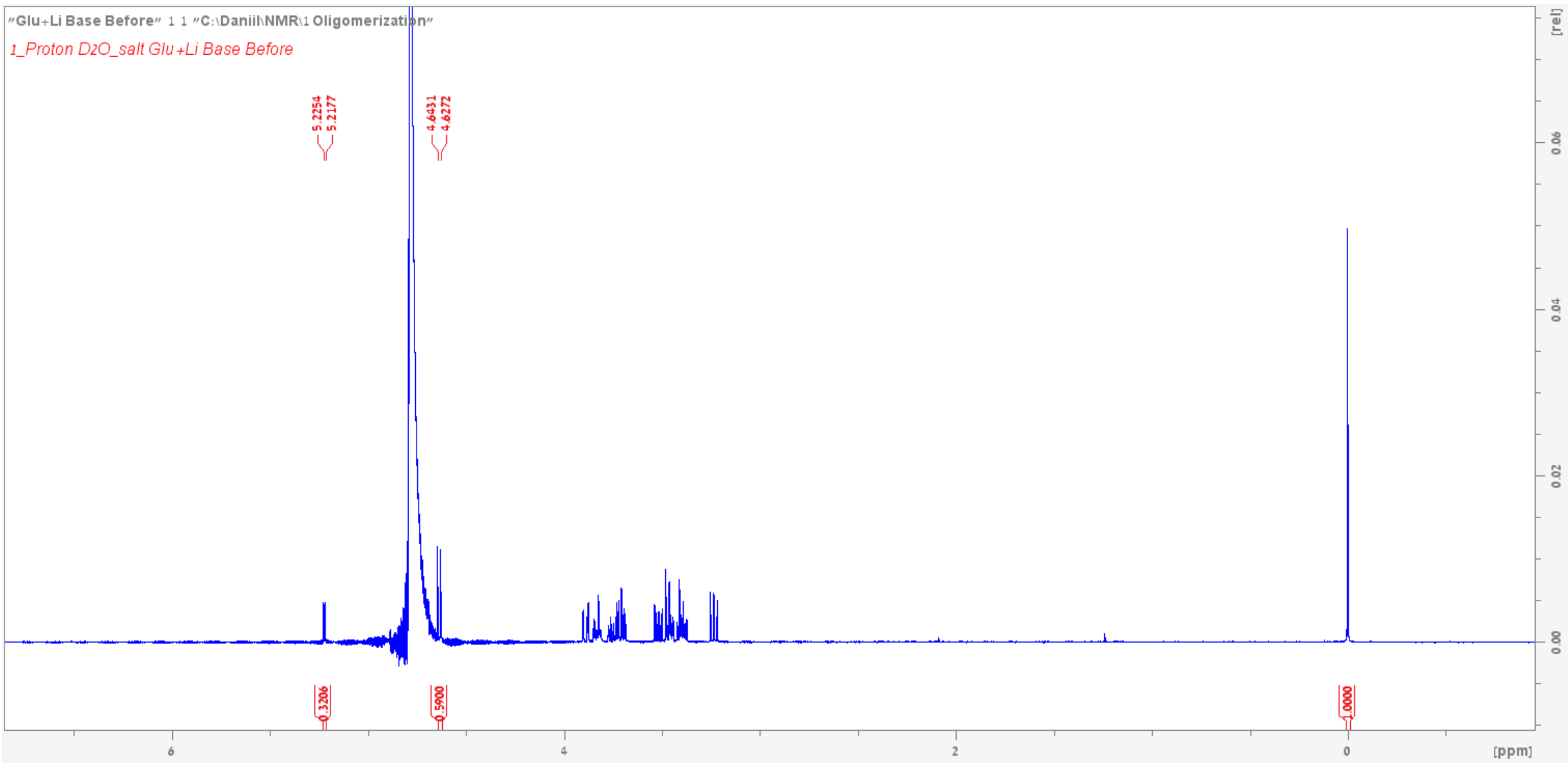

Figure 33. ^{1}H-NMR spectra of a fresh glucose upon addition of NaOH in the presence of $ZnCl_2$.

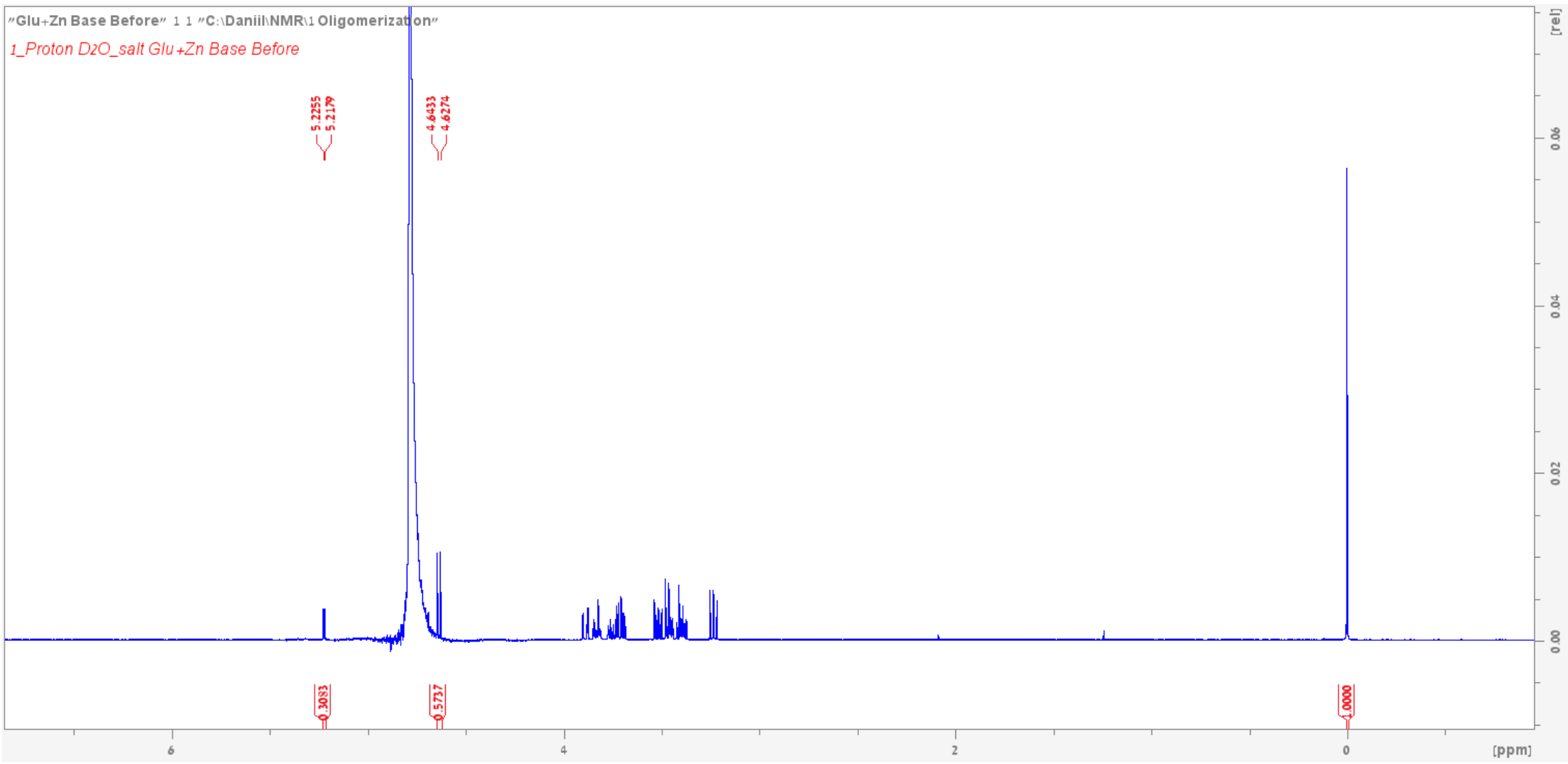

Figure 34. ^{1}H-NMR spectra of a fresh glucose upon addition of NaOH in the presence of NaCl.

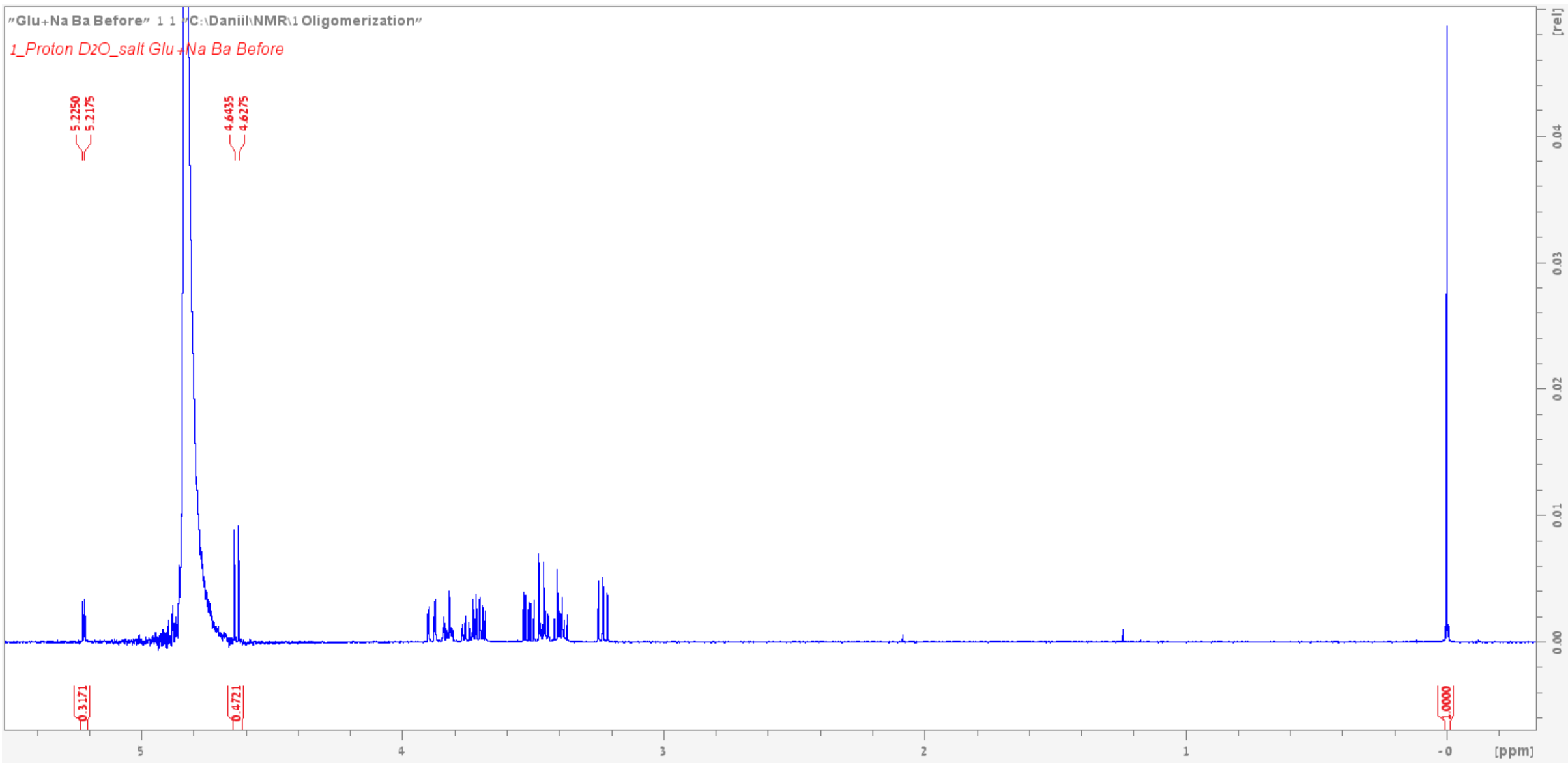

Figure 35. ^{1}H-NMR spectra of a fresh glucose upon addition of NaOH in the presence of KCl.

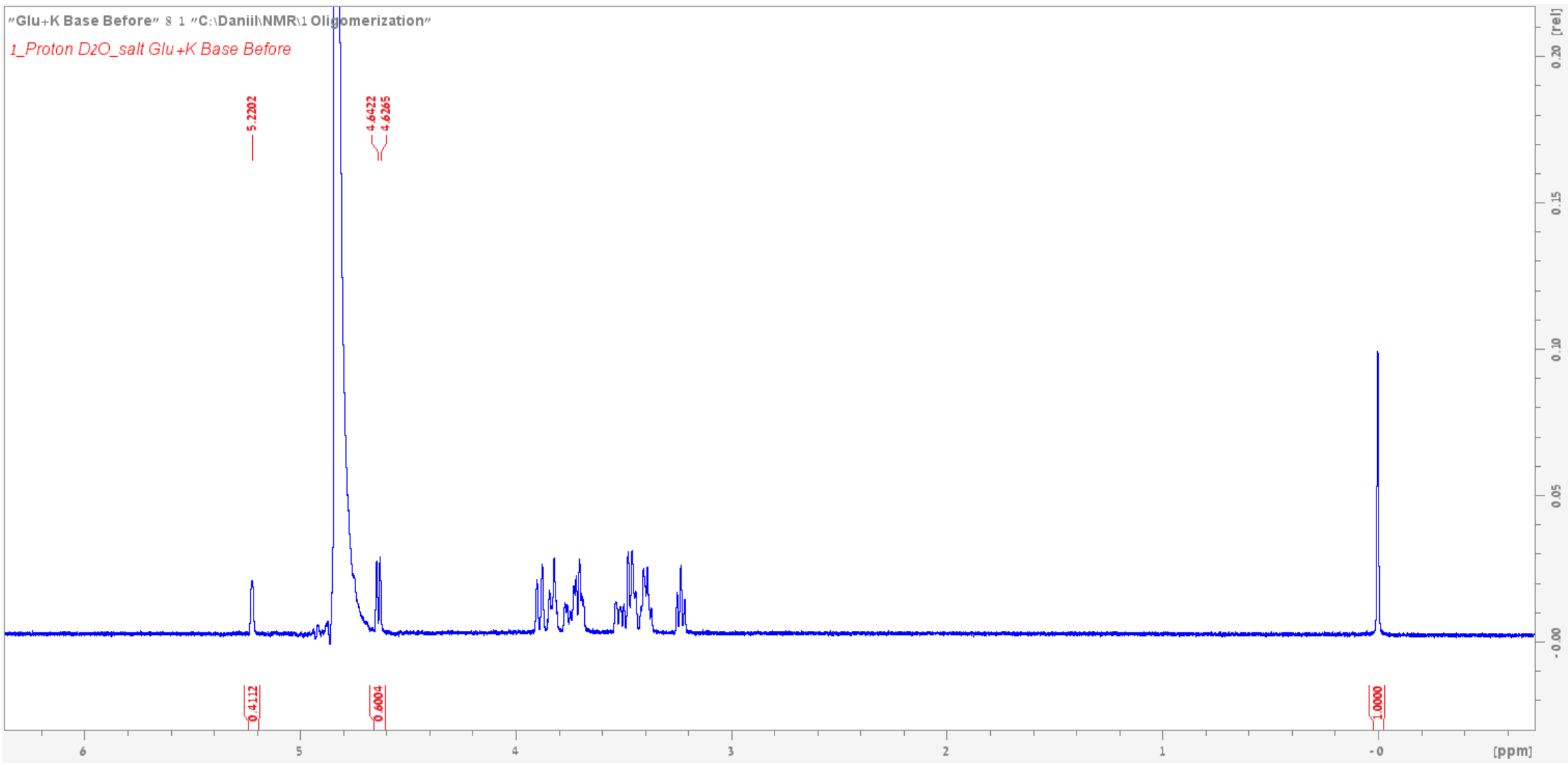

Figure 36. ^{1}H-NMR spectra of a fresh glucose upon addition of NaOH in the presence of $MgCl_2$.

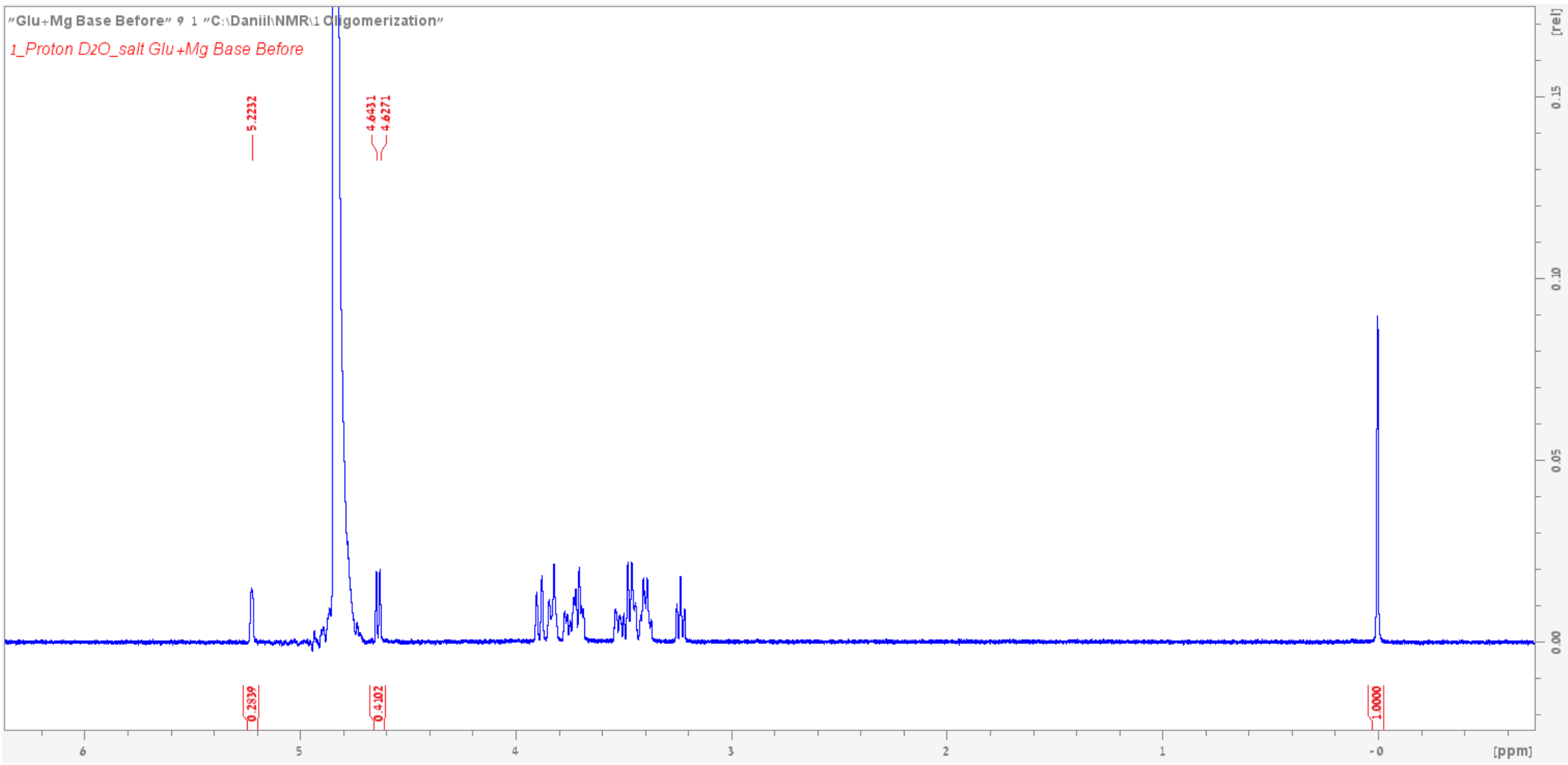

Figure 37. ^{1}H-NMR spectra of a fresh glucose upon addition of NaOH in the presence of $CaCl_2$.

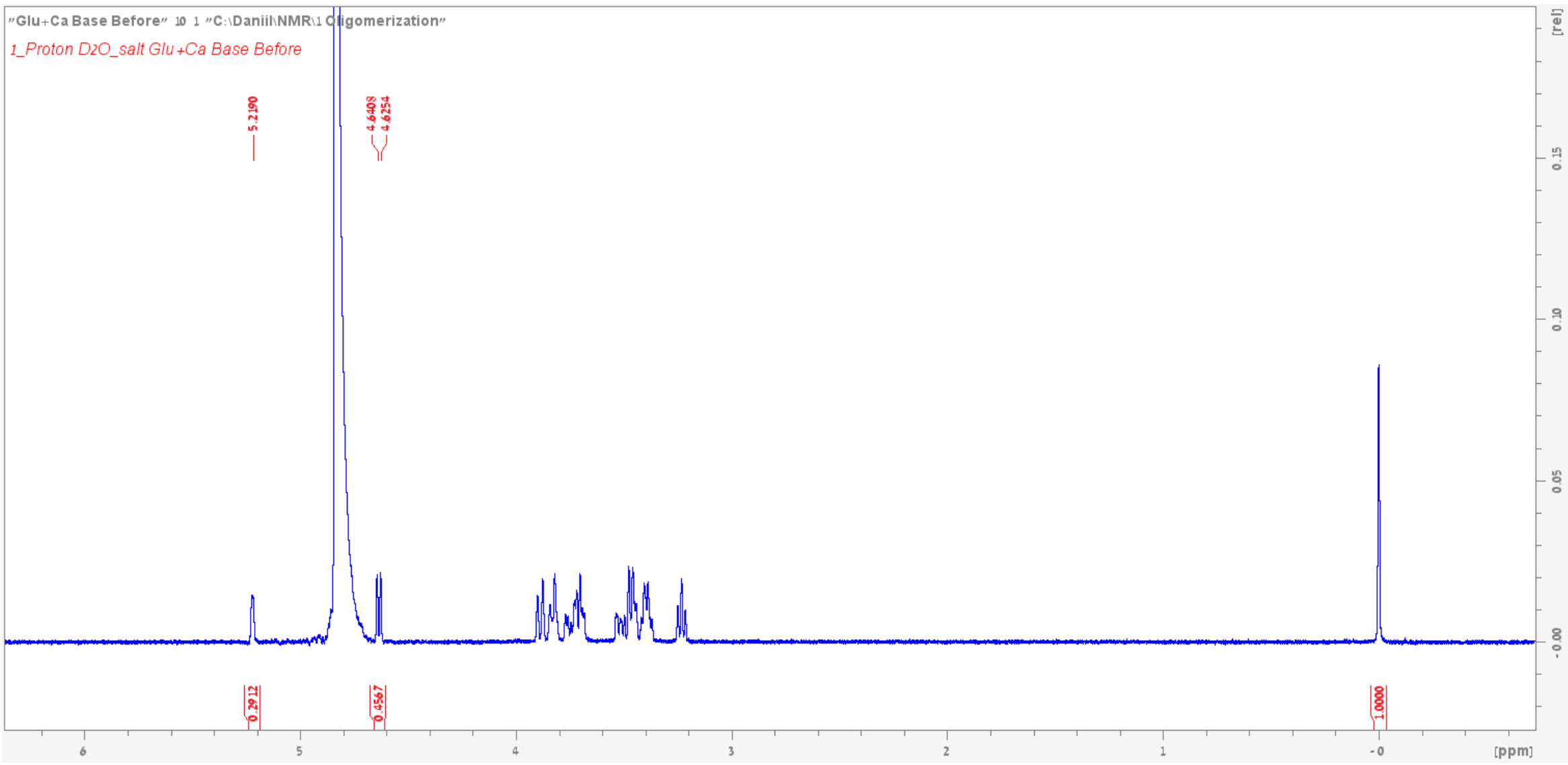

Figure 38. ^{1}H-NMR spectra of a fresh glucose upon addition of NaOH in the presence of $MnCl_2$.

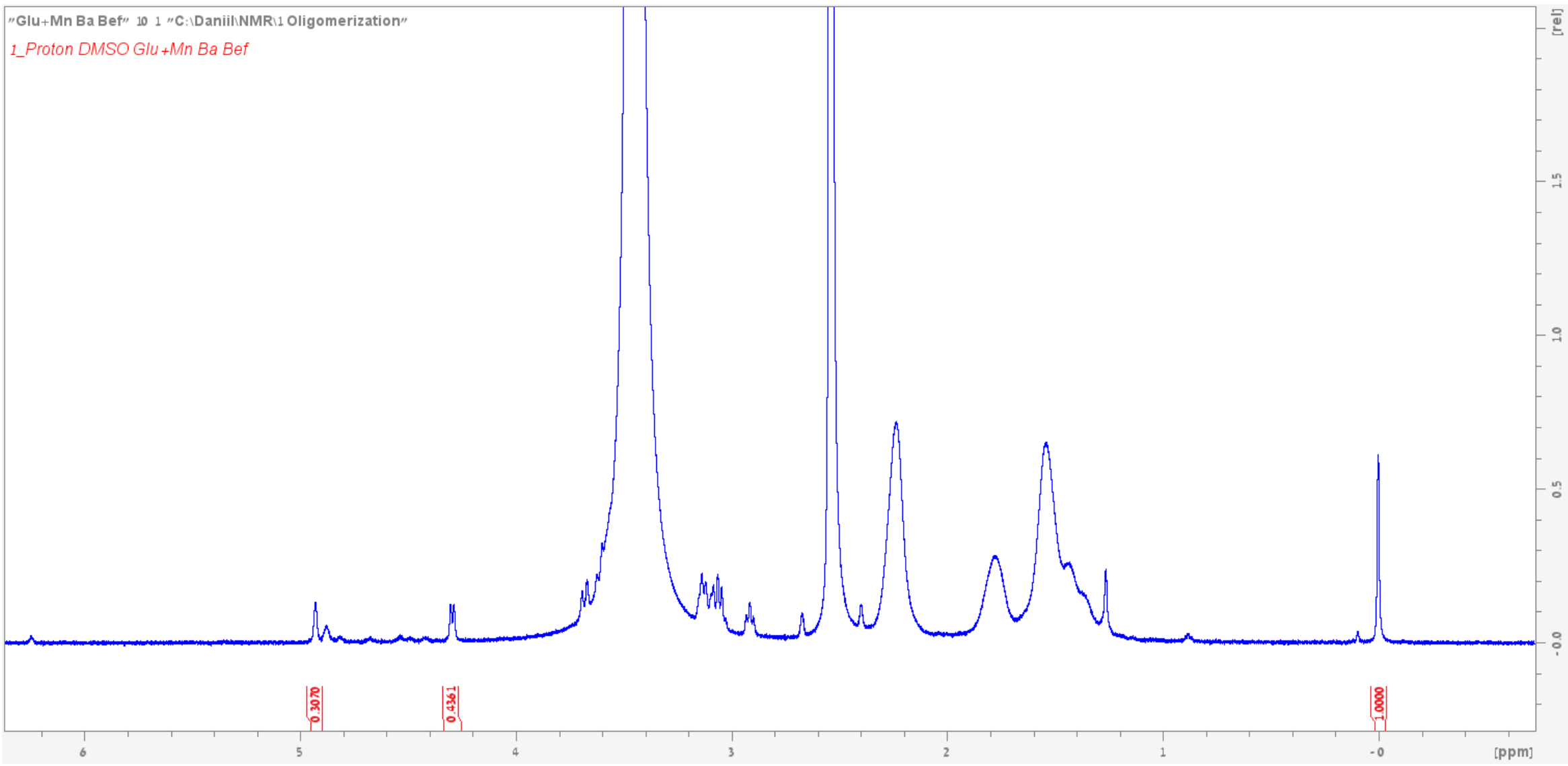

Figure 39. ¹H-NMR spectra of a fresh glucose upon addition of NaOH in the presence of $FeCl_2$.

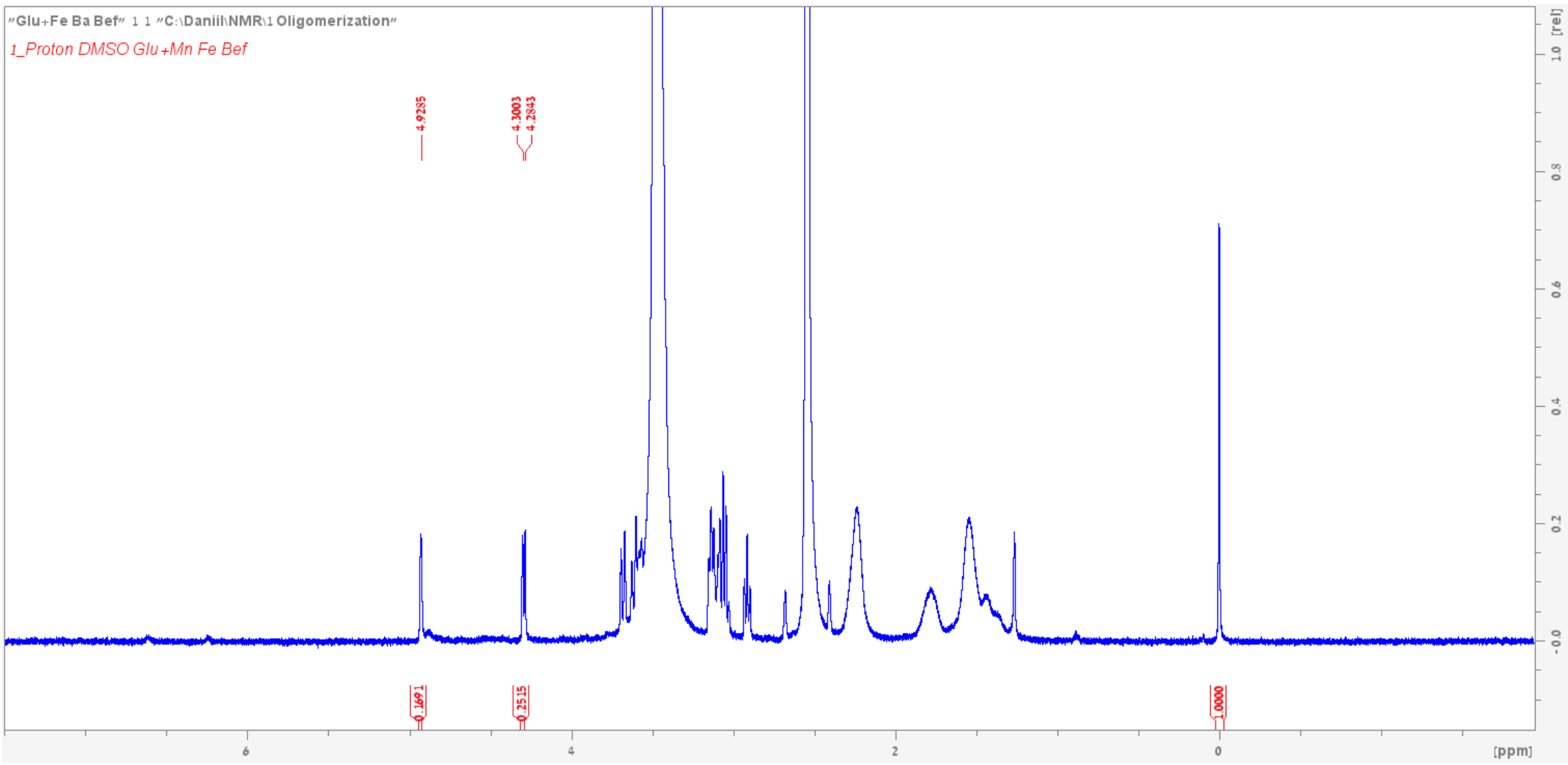

Figure 40. ¹H-NMR spectra of a fresh glucose upon addition of NaOH in the presence of $AlCl_3$.

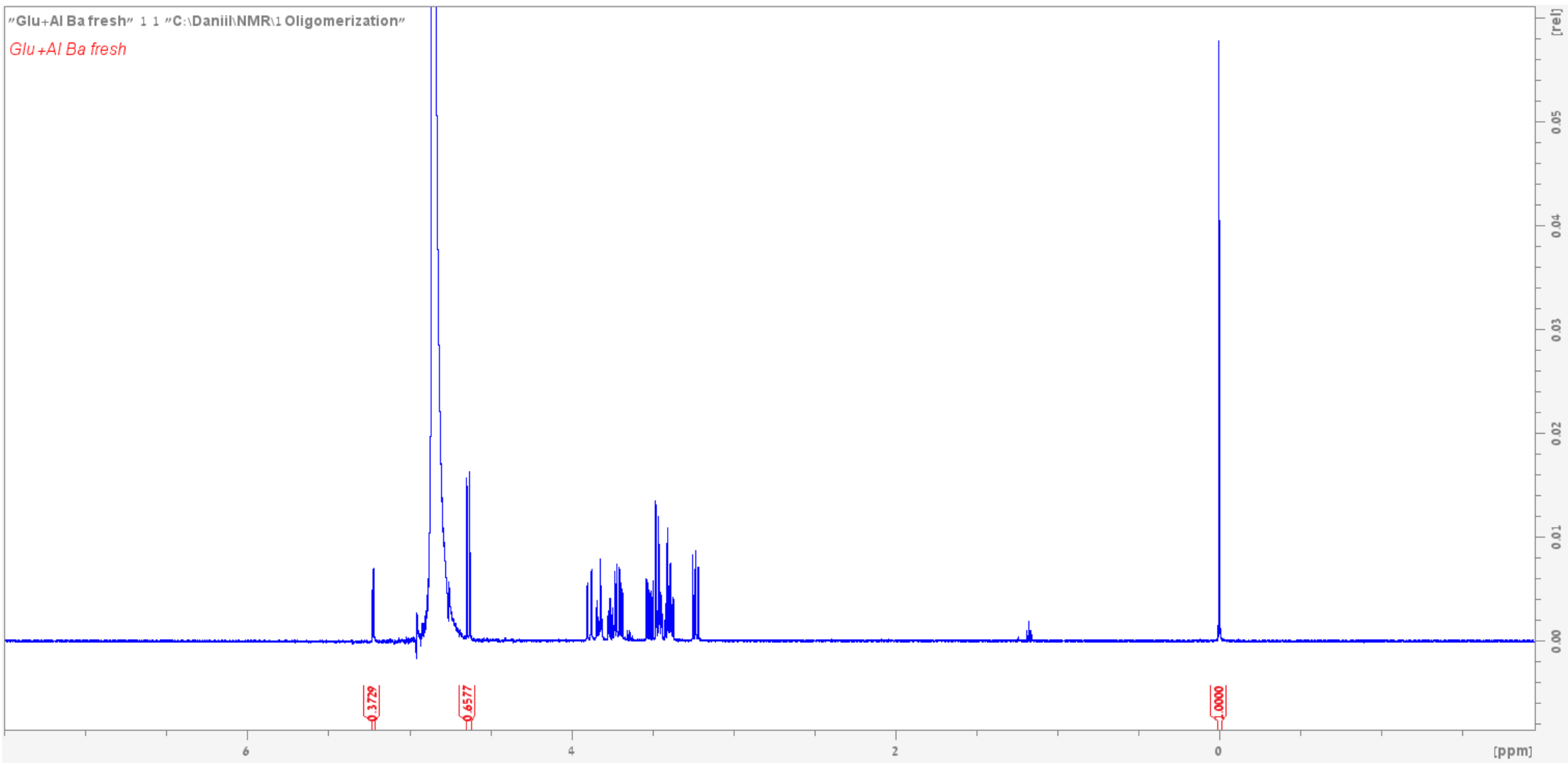

Supplement: Supplementary file 4 [file 42004_2026_2129_MOESM4_ESM.pdf]
